# The morphology and biochemistry of nanostructures provide evidence for synthesis and signaling functions in human cerebrospinal fluid

**DOI:** 10.1186/1743-8454-6-10

**Published:** 2009-09-07

**Authors:** Michael G Harrington, Alfred N Fonteh, Elena Oborina, Patricia Liao, Robert P Cowan, Gordon McComb, Jesus N Chavez, John Rush, Roger G Biringer, Andreas F Hühmer

**Affiliations:** 1 Molecular Neurology, Huntington Medical Research Institutes, Pasadena, CA, 91101 USA; 2 Neurosurgery, Children's Hospital of Los Angeles, Los Angeles, CA 90027 USA; 3 Neural Engineering, Huntington Medical Research Institutes, Pasadena, CA, 91101 USA; 4 Cell Signaling Technology, Danvers, MA 01923 USA; 5 Proteomics, Thermo Fisher Scientific, San Jose, CA 95134 USA

## Abstract

**Background:**

Cerebrospinal fluid (CSF) contacts many brain regions and may mediate humoral signaling distinct from synaptic neurotransmission. However, synthesis and transport mechanisms for such signaling are not defined. The purpose of this study was to investigate whether human CSF contains discrete structures that may enable the regulation of humoral transmission.

**Methods:**

Lumbar CSF was collected prospectively from 17 participants: with no neurological or psychiatric disease, with Alzheimer's disease, multiple sclerosis, or migraine; and ventricular CSF from two cognitively healthy participants with long-standing shunts for congenital hydrocephalus. Cell-free CSF was subjected to ultracentrifugation to yield supernatants and pellets that were examined by transmission electron microscopy, shotgun protein sequencing, electrophoresis, western blotting, lipid analysis, enzymatic activity assay, and immuno-electron microscopy.

**Results:**

Over 3,600 CSF proteins were identified from repeated shotgun sequencing of cell-free CSF from two individuals with Alzheimer's disease: 25% of these proteins are normally present in membranes. Abundant nanometer-scaled structures were observed in ultracentrifuged pellets of CSF from all 16 participants examined. The most common structures included synaptic vesicle and exosome components in 30-200 nm spheres and irregular blobs. Much less abundant nanostructures were present that derived from cellular debris. Nanostructure fractions had a unique composition compared to CSF supernatant, richer in omega-3 and phosphoinositide lipids, active prostanoid enzymes, and fibronectin.

**Conclusion:**

Unique morphology and biochemistry features of abundant and discrete membrane-bound CSF nanostructures are described. Prostaglandin H synthase activity, essential for prostanoid production and previously unknown in CSF, is localized to nanospheres. Considering CSF bulk flow and its circulatory dynamics, we propose that these nanostructures provide signaling mechanisms *via *volume transmission within the nervous system that are for slower, more diffuse, and of longer duration than synaptic transmission.

## Background

Physiological signaling *via *the CSF is not defined, but the phylogeny of CSF suggests a fundamental role in non-synaptic transmission between brain regions [1,2] and many aspects of neuroendocrine signaling in the choroid plexus and CSF have been reviewed [2]. Three lines of evidence support such communication. First, diverse structures at the CSF-contacting brain surfaces contain neurotransmitters, neuropeptides, and biosynthetic enzymes [1], suggesting components may be added to the CSF from its lining. Second, many signaling molecules (neurotransmitters, neuropeptides, and enzymes) are in contact with CSF at sites that are remote from their specific receptors [3], unlike the better-characterized spatial restriction of signaling molecules within synapses. In the absence of an enclosed synapse and facing the CSF spaces, these signaling molecules may diffuse *via *CSF to specific receptors on the cell membranes that border the fluid. Third, functional effects can be invoked *via *CSF: intracerebroventricular (icv) and intracisternal infusion of sodium or neuropeptides effect appetite [4], drinking [5], sleep [6], and pain perception [7]; and icv injection of β-amyloid dimers inhibits long-term potentiation in the hippocampus [8].

In spite of accumulating evidence for non-synaptic transmission, it is not known how the biosynthesis and transport of signals are regulated within the circulating CSF. For instance, neurotransmitters in solution would be rapidly inactivated, such as hydrolysis of acetylcholine by acetylcholinesterase in the CSF. Moreover, integral membrane proteins may not function optimally in aqueous CSF, such as the prostaglandin H synthase (PGHS) required to synthesize the sleep-inducing prostaglandin D_2 _[9]. The scope for signaling in CSF would, therefore, be enhanced if defined structures could a) protect contents from degradation; b) provide environments for hydrophobic constituents; c) localize enzyme activities; d) mediate receptor recognition for activation at specific locations; and e) be amenable for transport.

Here, we demonstrate that CSF consistently has a matrix of membrane- and protein-rich nano-scaled structures with many signal transduction components bounded by lipid membranes. These structures include features of vesicles containing acetylcholine, large dense-core vesicles (LDCVs), exosomes, and spherical structures with functional prostaglandin H synthases (PGHS) -1 & -2. People in health and disease states have these structures that contribute to CSF heterogeneity, and provide unique transport and signaling capabilities throughout CSF-contacting brain surfaces.

## Methods

### Materials

The following were purchased: Quant-iT protein assay kit (Invitrogen/Molecular Probes, Carlsbad, USA); ultrafree-MC filters and immobilon-P polyvinylidine chloride (PVDF) membrane (Millipore, Bedford, USA); 1-Step™ NBT/BCIP (Pierce, Rockford, USA); centrifuge tubes 326814 and 344057 were used in 50 ti and 50.1 sw rotors on an L8-70 ultracentrifuge (Beckman Instruments, Fullerton, USA); ammonia, ultrapure HPLC grade water, parafilm, methanol, and chloroform (VWR, West Chester, USA); luna silica normal phase HPLC column (Phenomenex, Torrance, USA), glutaraldehyde, phosphate buffered saline (PBS), sucrose, triethanolamine, phenylmethanesulphonylfluoride (PMSF), leupeptin, bovine serum albumin (BSA), glycine, Triton X-100, bovine brain phosphatidylcholine (PC), phosphatidylethanolamine (PE), and phosphatidylserine (PS), and phospholipase A_2 _(PLA_2_) from *Naja Mossambica *(Sigma, St Louis, USA); cyclooxygenase activity assay and phosphatidylinositide (PI, soybean, Cayman Chemical, Ann Arbor, USA); electrophoresis chemicals and criterion gels (BioRad, Hercules, USA); synthetic lipid standards (Avanti Polar Lipids, Alabaster, USA); uranyl acetate, Whatman #2 filters, EPON, and 200 mesh nickel formvar/carbon coated grids (Electron Microscopy Sciences, Hatfield, USA); and paraformaldehyde powder (Ted Pella, Redding, USA.)

### Antibodies and immune sera

ADP-ribosylation factor-like protein 2 (ARL2) was a generous gift from RA Kahn (Atlanta, GA, USA). 3-oxo-5-alpha-steroid 4-dehydrogenase 2 (S5A2), secretogranin-3 (SCG3), prostaglandin G/H synthase 2 (PGHS-2) were rabbit polyclonal antibodies made by MGH & John Rush (Cell Signaling Technology, Inc., Danvers, USA) from synthetic peptides; S5A2 from CAGAGHHRFYLKMFEDYPKSRKALIPFIF; SCG3 from CAGAGKEAKEKETLITIMKTLIDFV; PTGDS from APEAQVSVQPNFQQD [10]. The following antibodies were purchased: Fibronectin (FINC), prostaglandin H synthase-2 (PGHS-2), synaptotagmin, syntaxin, secondary donkey anti-goat IgG-AP, goat anti-mouse IgG-AP, goat anti-rabbit IgG-AP, and control pre-immune sera (Santa Cruz Biotechnology, Santa Cruz, USA); synaptosomal-associated protein 23 (SNAP 23) and synaptobrevin (Synaptic Systems, Goettingen, Germany); Ras-related protein Ral-A (RALA) (BD Transduction Laboratories, Franklin Lakes, USA); semaphorin 4D (Chemicon, Temecula, USA); chromogranin A & B, and acetylcholine (Abcam, Cambridge, USA); 6 and 12 nm gold-conjugated, species-specific secondary antibodies (Jackson ImmunoResearch Laboratories, Inc., West Grove, USA).

### CSF sources (Table 1)

**Table 1:** Clinical details and study assignments for CSF samples

**Sample #**	**Age** **(Yr)**	**Male/Fem**	**Diagnosis**	**Source**	**Study Series**	**Fractions** **Used**
1	65	F	Alzheimer's	Lumbar	Protein LCMS	S1
2	70	M	Alzheimer's	Lumbar	Protein LCMS	S1
3	18	F	Migraine	Lumbar	Protein LCMS	S3, P3
3'	18	F	Migraine	Lumbar	Protein LCMS	S3, P3
4	35	F	Migraine	Lumbar	TEM & WB	S1, P2, S3, P3
5	28	F	Migraine	Lumbar	TEM	S1, S3, P3
6	40	M	Migraine	Lumbar	TEM & WB	S1, P2, S3, P3
7	40	F	Migraine	Lumbar	TEM & WB	S1, P2, S3, P3
8	50	F	Normal	Lumbar	TEM & WB	S1, P2, S3, P3
9	78	F	Normal	Lumbar	TEM & WB	S1, P2, S3, P3
10	84	M	Normal	Lumbar	TEM & WB	S1, P2, S3, P3
11	80	F	Alzheimer's	Lumbar	TEM & WB	S1, P2, S3, P3
12	65	F	Multiple sclerosis	Lumbar	TEM & WB	S1, P2, S3, P3
12'	65	F	Multiple sclerosis	Lumbar	TEM (fresh fluid)	S3, P3
13	45	F	Multiple sclerosis	Lumbar	TEM	S1, S3, P3
13'	45	F	Multiple sclerosis	Lumbar	TEM (fresh fluid)	S3, P3
14	50	M	Migraine	Lumbar	TEM, WB, & ipids	S1, P2, S3, P3
15	28	F	Migraine	Lumbar	TEM, WB, & ipids	S1, P2, S3, P3
16	42	F	Migraine	Lumbar	TEM, WB, & ipids	S1, P2, S3, P3
17	41	F	Migraine	Lumbar	TEM & ipids	S1, S3, P3
18	11	M	Congenital hydroceph.	Ventricular	TEM & Enzyme	S1, S3, P3
19	5	F		Ventricular	TEM & Enzyme	S1, S3, P3

**Figure 1:**
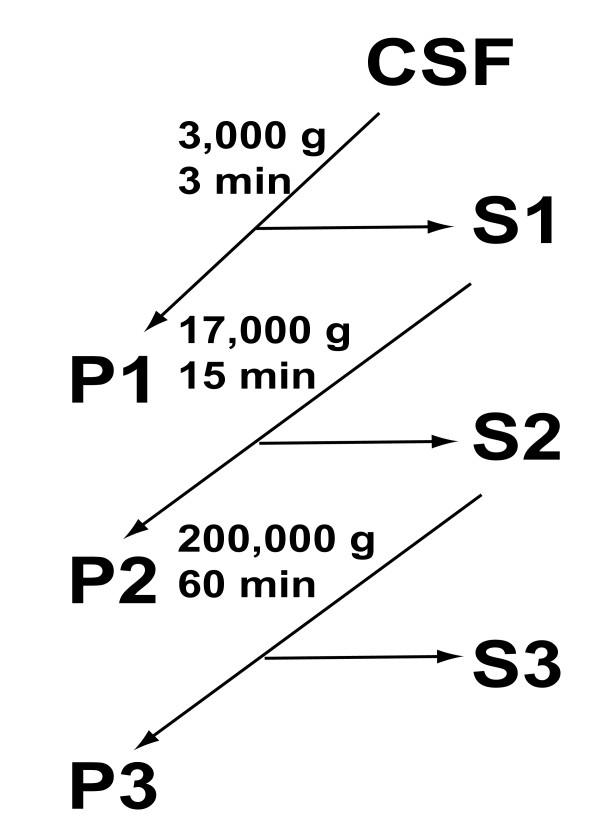
**Scheme for CSF purification**. Outline of three supernatant and pellet collections. The repetitive shotgun protein sequencing was applied to S1s, and the high-resolution sequencing compared S3 to P3 fractions. Western blots, electrophoresis, and TEMs mainly compared S1 and S3 fractions to P3s. Nanostructures were found by TEM in all 16 P3s examined. Lipids were compared between P3 and S3 fractions.

LCMS: liquid chromatography mass spectrometry; TEM: transmission electron microscopy; WB: Western blot; S1, S3, P2, P3 as designated in figure 1.

Twenty lumbar and two ventricular CSF samples were collected prospectively for compositional studies after obtaining informed consent from 19 different participants, three of whom were sampled on two separate occasions (IRB-approved consents and protocols, Huntington Hospital and Children's Hospital of Los Angeles). Twenty one mL of lumbar CSF was collected from the L2/3 or 3/4 inter-spaces, between 1 and 5 pm, with opening pressure measured in cm CSF in the recumbent position. Two ventricular CSF samples (each of approximately 250 mL) were collected by conventional external drainage (Becker EDMS, Medtronic, Goleta, USA) at room temperature over 24 h, with pressure measured continually in cm CSF, referenced to the external auditory meatus.

### Study participant selection and diagnostic criteria

Selection for lumbar samples was based on participants that represent health, migraine, inflammatory, or degenerative brain disorders. Selection for ventricular samples was based on participants that had received long-term shunts for congenital hydrocephalus and had normal cognitive function. Normal (n = 3): no classifiable neurological or psychiatric disorder after detailed structured interview and clinical assessment; Multiple sclerosis (n = 2): diagnosis of clinically definite multiple sclerosis based on national criteria [11]; Alzheimer's disease (n = 3): clinically probable Alzheimer's disease, based on the national criteria for the diagnosis of AD [12]; Migraine (n = 9): migraine with/without aura, as per the International Headache Classification [13]; Congenital hydrocephalus (n = 2): Children with normal neurological and psychiatric development for either 5 or 11 years who had ventriculo-peritoneal shunts for congenital communicating hydrocephalus; their CSF was collected after ventriculostomy with external drainage that was necessary to treat acute appendicitis.

### CSF preparation and storage

All CSF was prepared, aliquoted, and analyzed immediately or stored frozen within 1 h of collection. Table 1 indicates the clinical features of each sample and the analyses that were performed. Figure 1 illustrates the fractionation procedures: cells were first pelleted, P1, by centrifugation for 3 min at 3,000 g. Cells in P1 were counted on a chamber (Hausser Scientific Partnership, Horsham, USA). Remaining S1s were aliquoted and store in 1.1 mL aliquots at -80°C until use, or analyzed immediately (#s 12' & 13', Table 1). The CSF supernatant, S1, was further centrifuged (modified from [14], Figure 1) for 15 min at 17,000 g. This yielded a second pellet, P2, and the supernatant, S2, was centrifuged for 1 h at 200,000 g. This supernatant, S3, was collected and the final pellet, P3, was re-suspended in 50 μL isolation solution (250 mM sucrose, 10 mM triethanolamine, 0.5 mM PMSF, and 1 μM leupeptin) that had been passed through a 0.45 μm filter.

### Protein assay and trypsin digestion

Concentrations of total protein (in triplicate) were determined using a microplate-based Quant-iT protein assay kit with BSA, 0-500 μg/mL as standard, as recommended by the manufacturer. For all protein shotgun sequencing (below), CSF fractions were denatured (6 M urea), reduced, amidocarboxymethylated, washed with 100 mM ammonium bicarbonate (pH 8), filtered on Viva Spin 500 (Viva Products, Littleton, USA), digested with trypsin (Princeton Separations, Inc., Freehold, USA) overnight at 37°C, and quenched with formic acid.

### Protein shotgun sequencing of S1 fractions

Liquid chromatography and mass spectrometry (LCMS) was applied to the S1 of two different samples (#s 1 & 2, Table 1) using the following LC and MS formats, repeated 15 times, to determine as many different CSF proteins as possible.

#### A. Orthogonal 2-dimensional LC electrospray linear ion trap MS System

This was performed using the Proteome X Linear Ion-Trap System (Thermo Fisher Scientific). The system was fitted with a strong cation exchange column, SCX 320 μm ID × 100 mm (Thermo Fisher Scientific) and two C18 reversed-phase nanotrap columns: IntegraFrit Trap, 75 μm ID × 25 mm, Biobasic™ C18 followed by a PicoFritTM nanobore HPLC column with a 15 μm i.d. pulled tip, 75 μm ID × 10 cm Biobasic™ (both from New Objective, Inc., Woburn, USA). 100 μg of S1 protein digest was injected onto the SCX column and peptides were eluted onto a nanotrap column by successively injecting 20 μl of NH_4_Cl solution with concentrations: 0, 10, 20, 40, 60, 80, 120, 150, 200, 400 and 800 mM. Each of the 11 salt steps was synchronized with a linear gradient from 0 - 60% B over 180 min at a flow rate of 220 nL/min (A = 0.1% formic acid in water, B = 100% acetonitrile containing 0.1% formic acid) for separation of the peptide mixture on the 10 cm PicoFrit separation column. Peptides that eluted from the reverse phase column were analyzed by a Finnigan LTQ™ linear ion trap mass spectrometer that was equipped with a nano-electrospray ion source (both Thermo Fisher Scientific). Data-dependent mass spectral acquisition (MS/MS) was enabled and allowed for the MS/MS analysis of the most intense ion in the range of 450-1600 m/z (full scan) with the following dynamic exclusion settings: repeat count, 1; repeat duration, 0.5 min; exclusion duration, 3.0 min.

#### B. 1-dimensional nano-LC electrospray ion trap MS

A modified version of the Pepfinder Kit with a Surveyor HPLC, autosampler, and nanoflow solvent delivery (Thermo Fisher Scientific) was used to present the CSF sample to an LTQ ion trap mass spectrometer equipped with a nano-electrospray ion source (Thermo Fisher Scientific) and 30 μm PicoTip emitter (New Objective, Woburn, USA). The Pepfinder kit was modified from its original form to contain a 5 × 0.3 mm Zorbax™ C-18 peptide trap (Agilent Technologies, Santa Clara, USA) or a 0.075 × 25 mm Biobasic C18 IntegraFrit Trap combined with a 100 μm ID × 25 cm BioBasic 18 nanobore C-18 separation column (both from New Objective). 1 - 4 μg of CSF S1 protein digest was injected onto the trap, washed, and then eluted onto and through the C-18 column with a pseudo-exponential gradient profile, from 0-80% B in ~4 hr in following gradient increments; 0.1%/min in 50 min, 0.2%/min for 50 min, 0.25%/min for 40 min, 0.33%/min for 60 min, 0.44%/min for 45 min and 4%/min for 5 min (A = 0.1% formic acid, B = 0.1% formic acid in acetonitrile). The mass spectrometer was operated in a data-dependent MS/MS mode and dynamic exclusion was enabled. Gas-phase fractionation with three distinct scan ranges (450-600 m/z, 650-900 m/z, 900-1600 m/z) was used to maximize the number of peptides identified as described [15]. The number of MS/MS scans varied with the scan range: 1 MS + 10 MS/MS for 450-600 m/z; 1 MS + 8 MS/MS for 650-900 m/z; 1 MS + 4 MS/MS for 900-1600 m/z. These experiments were repeated 15 times over 18 months with CSF samples # 1 & 2 (Table 1), using increasing amounts of protein digest. Amounts injected varied from 1.3 to 400 μg for each analysis.

MS/MS spectra obtained from these LCMS analysis of the S1 protein digests of sample #s 1 & 2 (Table 1) were searched against a Swiss Prot database (release 7459) using the SEQUEST^® ^algorithm [16] implemented in BioWorks™ 3.1 (Thermo Fisher Scientific). Trypsin enzyme with potentially 2 missed cleavages was specified as a search parameter. Protein identification was dependent upon Xcorr score fit. Protein matches were identified using strict Washburn criteria, based on the charge of the precursor peptide ion and the Xcorr assigned by Bioworks software. These criteria (z = +3, Xcorr > 3.75; z = +2, Xcorr > 2.1; and z = +1, Xcorr > 1.8) [17,18] allow for the greatest confidence in correct peptide sequence assignment within a single sample run. The list of matched peptides was then further evaluated using the Request/Unified scoring in Bioworks with a value of 2400 as the cut-off filter [19]. Additionally, the data were validated with a probability-based algorithm that calculates a statistical expectation value (SE-1) of database peptide matches based on the DeNovoX™ peptide sequencing pre-integration algorithm in a pre-release version of Bioworks 3.3. Peptides with a SE-1 of > 10^-5 ^were accepted for protein analysis. Post-translational modifications were assessed using a fully automated, *de novo *sequencing software program, DeNovoX (Thermo Fisher Scientific). A comprehensive list of non-redundant proteins was generated from all analysis runs (n = 15), using Excel sorting and Pivot Table functions. Results were compared to existing identifications [20] and subdivided by the gene ontology (GO) class of components.

### High mass accuracy shotgun protein sequencing of S3s and P3s

Analysis of trypsin digests of CSF by high mass accuracy was essentially as described above, to get the best sensitivity from samples that were available in limited quantity. For these studies, instead of S1 samples, S3 and P3 from sample #s 3 & 3' (Table 1) were analyzed using an LTQ-FT mass spectrometer (Thermo Fisher Scientific). For the supernatant (S3) 60 μg of protein digest was directly injected into a 0.5 μL guard C18 cartridge (LC-Packings, Sunnyvale, USA) and separated on Biobasic C18, 10 cm × 75 μm ID with a 3 h exponential gradient (n = 2). Identical analysis conditions were used for the protein digests from pellets (P3) using 4 μg of sample (n = 3).

MS/MS spectra of the S3 & P3 protein digests of samples 3 & 3' were searched against an NCBI 25H-sapiens database (71932 entries) with Bioworks 3.2 in full tryptic and semi-tryptic search mode using the Sequest algorithm implementation of SORCERER (SAGE- N Research, San Jose, USA). For unit resolution data, a precursor tolerance of 2Da was used, while 100 ppm tolerance was applied for high mass accuracy data. Positive identifications were established using the probability calculations in Bioworks 3.2, with a statistical expectation of > 10E-5. Results from tryptic and semi-tryptic searches were combined and filtered after export into Excel. A comprehensive list of non-redundant proteins was generated from all analysis runs (n = 6) using Excel sort and Pivot Table functions. Results were compared to existing identifications [20].

### Electron microscopy of filtered CSF particles

S1 samples (1.5 mL) from #s 1, 2, 5, & 6 (Table 1) were centrifuged through 0.45 μm ultrafilters at 10,000 rpm. Gluteraldehyde (3%, 100 μL) was added for 1 hr, removed, and the filters washed with PBS, air-dried, and stained with 2% uranyl acetate followed by Reynolds' lead citrate. PBS replaced CSF for negative controls. Filters were then embedded in EPON 812 (Electron Microscopy Sciences, Hatfield, USA) and cut on an Ultramicrotome UCT (LKB Instruments, Inc., Gaithersburg, USA). Sections were viewed on a Morgagni 268D transmission electron microscope (TEM; FEI, Hillsboro, USA) and images were recorded on a digital camera, Mega View II, visualized with Soft Imaging Systems and AnalySIS 3.0 software (Soft Imaging Systems, Münster, Germany).

### TEM of S1, S3, and P3 CSF fractions from all participant sample #s 4-19

All procedures were performed in covered Petri dishes to prevent contamination. Negative controls were composed of isolation solution processed without added CSF fractions. Samples were fixed in 2% paraformaldehyde and grids were floated on the sample, washed in PBS then H_2_O, negatively stained in 0.5% uranyl acetate, dried, and examined in the Morgagni 268D TEM. To estimate the number of nanospheres in CSF P3s, nanospheres per grid were counted on 10 occasions from 1 μL of 1:1 diluted P3 suspensions.

### Electrophoresis, western blotting, and Immunostaining

Samples from at least four different participants from #s 4, 6-12, 14-16 (Table 1) were randomly evaluated for each procedure or antibody. S1, S3, P2, or P3 fractions were applied in the same amount of total protein per well on 4-20% Tris-HCl gels, transferred to PVDF, and total proteins were visualized with colloidal gold. Specific antigens were visualized after immunolabeling with primary antibodies, followed by alkaline phosphatase-conjugated secondary antibodies, and NBT/BCIP detection. Species-specific pre-immune serum was substituted in place of primary antibodies as negative controls. Dry blots were digitized and band intensities quantified on UN-SCAN-IT 6.1 software (Silk Scientific Corporation, Orem, USA).

### Immuno-TEM (iTEM)

P3 samples of at least four participants per antibody were tested, randomly selected from #s 4-19 (Table 1). P3 suspensions were mixed 1:1 with 4% paraformaldehyde on which the grid was floated, washed in PBS, 0.05 M glycine, and PBS, and exposed to primary antibody (usually diluted 100-fold). Negative controls had species-specific pre-immune sera substituted for primary antibody. Grids were washed in PBS, exposed to 1:40 dilution of 6 or 12 nm gold-conjugated secondary antibody, washed in PBS and H_2_O, stained with 0.5% uranyl acetate, air-dried, and visualized as per TEM.

### Lipid extraction and liquid chromatography of sample #s 14-17

Lipids were extracted from S3 fluids and P3 pellets using the method of Bligh Dyer [21]. After removal of the organic chloroform layer using a stream of N_2_, lipids were suspended in 100 μl sample solvent (chloroform/methanol/water, 7:3:0.5 v/v/v) then eluted through a silica column with a chloroform/methanol/water/ammonium hydroxide gradient [22]. The elution profile for lipids was in the order: ceramides (CM), cerebroside sulfates (CS), phosphatidylglycerol (PG), PE, PI, PS, phosphatidic acid (PA), PC, sphingomyelin (SPM), platelet-activating factor (PAF) and lysophosphatidylcholine (LPC).

### Mass spectrometry of lipids of sample #s 14-17

Precursor ion scans (PIS) and neutral ion loss (NIL) for different lipids were obtained using a full scan MS infusion experiment on a triple quadrupole mass spectrometer, TSQ Quantum (Thermo Fisher Scientific) operated at a spray voltage of 4500 V, sheath gas pressure of 40 units, auxiliary gas pressure of 0, capillary temperature of 225°C and collision pressure of 1.5 units. Negative ions were acquired in the profile mode with 13 different scan events after collision induced dissociation (20-24 V) of deprotonated precursor ions or the neutral loss of specific groups from lipids. Negative PIS of 196.3 (mass range 650-950), 171.14 (mass range 600-900), 240.96 (mass range 750-1200), 168.17 (mass range 600-900) were used to monitor PE, PG/PA, PI and SPM, respectively. Negative NIL of 86.99 (mass range 650-900) and 50.13 (mass range 400-1000) were used to monitor PS and PC/LPC/PAF, respectively. Lipids containing eicosanpentanoeate (EPA, m/z = 301.24), arachidonate (AA, m/z = 303.15) and docosahexaenoate (DHA, m/z = 327.22) were detected using PIS of these ions in a mass range from 600-1200. Peak intensities were integrated, processed, and mole quantities determined using ICIS and Xcalibur software (Thermo Fisher Scientific). Mole quantities were determined from standard curves obtained using known amounts of lipid standards (0-400 ng).

### PLA2 digestions of sample #s 14-17

P3 samples were incubated with PLA_2 _at 37°C overnight in reaction buffer. The same incubation was carried out with heat-denatured enzyme, controls without enzyme. Reaction products were stored at 4°C until assayed either by LCMS or TEM with negative staining as described above.

### PGHS activity assay of sample #s 18 & 19

All assay components were pre-equilibrated to the room temperature except for the PGHS-2 Standard that was kept on ice. Detection was based on measuring the peroxidase activity of cyclooxygenase by colorimetrically monitoring the appearance of oxidized N, N, N', N'-tetramethyl-p-phenylenediamine at 595 nm in 96 well plates. Either Dup-697 (PGHS-2 inhibitor) or SC-560 (PGHS-1 inhibitor) was added to inhibitor wells and both inhibitors were added into background wells. Standard, samples, and backgrounds were analyzed in duplicates. The plate was carefully shaken and after 5 min of incubation at room temperature, absorbance was read on the V*max *kinetic microplate reader at 595 nm (Molecular Devices, Sunnyvale, CA). PGHS activity (nmol/min/mg protein) was calculated as described by the kit manufacturer (Cayman Chemical, Ann Arbor, USA).

## Results

### CSF quality

Pressure was normal at all lumbar (<150 cm) and ventricular (< 15 cm) CSF collections. All fluids were clear, with < 5 white blood cells per mL, and no red blood cells were seen. All samples had total protein content within the normal range of 0.1 - 0.5 g/L.

### CSF protein composition

**Figure 2:**
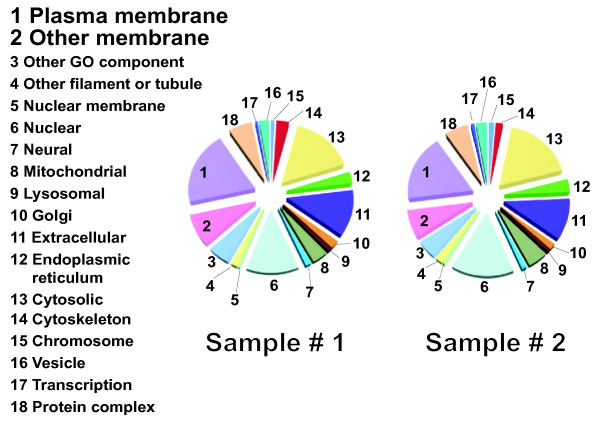
**Pie charts of total CSF proteins identified by LCMS from 15 replicates of shotgun sequencing of S1 fractions from two different participants (sample #s 1 and 2, Table 1) and charted as GO components**. There was a similarity between the two samples (complete protein lists are in Additional File 1). The principal groups of interest are the large percent of membrane proteins: Plasma (1) and Other Membranes (2) that are present in both samples.

Using sample #s 1 & 2 (Table 1), we identified with high confidence 2,390 and 3,649 proteins in the S1s from two Alzheimer's disease participants, respectively, by repeated LCMS shotgun protein sequencing (Figure 2, and additional file 1). These lists have similar overall protein categories to those already identified [23]. In this paper, we highlight the GO category of components (Figure 2) revealing that one quarter of CSF proteins we identified are normally resident in membranes.

### Morphology of CSF nanostructures

**Figure 3:**
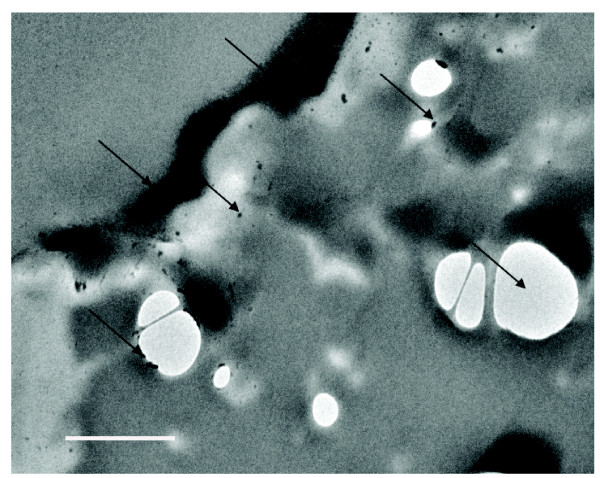
**Representative TEM of CSF structures trapped on the filter from four different S1 fractions**. Arrows point to structures (dark) and filter cavities (light). Scale bar 200 μm.

Using sample #s 4-19 (Table 1), to isolate any membranous structures in CSF, we examined the ultra-filters of the S1s from four study participants by TEM. In all cases, we found sub-cellular particles embedded in the surface of the filter (Figure 3) similar to those reported by Agnew and colleagues [24]. Since this preparation renders it difficult to assess morphology and biochemistry, we fractionated the S1s (Figure 1) by ultracentrifugation [14], collected the final supernatant (S3), and re-suspended the final P3 pellets from 16 people diagnosed as either healthy controls or people with migraine, multiple sclerosis, or Alzheimer's disease. The P3s contained 10 (+/- 10) ng of total protein per mL of CSF.

**Figure 4:**
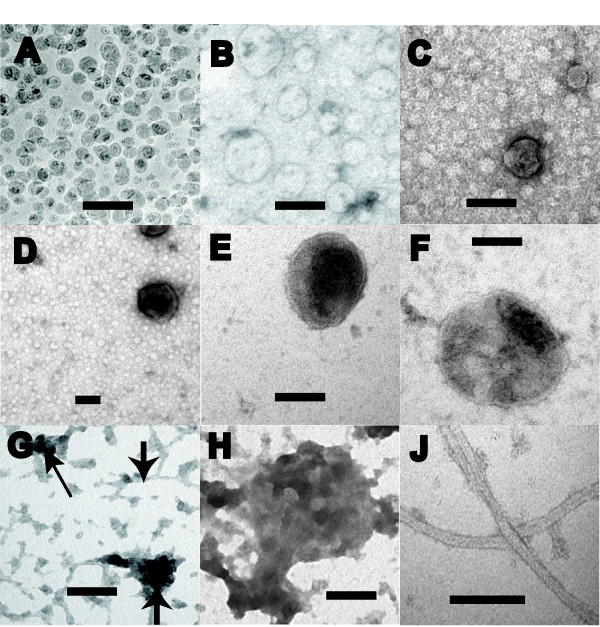
**TEM of ultrastructures from P3 fractions, representative of 16 samples**. A-F: abundant nanospheres; D-F: including large dense core vesicles; G: nanoblobs (upward arrows) and nanostrands (downward arrow); H and J: nanodebris resembles structures previously reported from ventricle walls. Scale bars 100 nm.

P3s from all 16 subjects had abundant, negatively stained structures (Figure 4) that were almost absent from S3. There was no difference in the observed structures when we prepared S3/P3 fractions freshly from two participants (sample #s 12 & 13, Table 1) as compared to preparations from their S1 samples that had been stored at -80°C for 12 months. The most common structure was roughly spherical with a mean diameter of 50 nm, ranging between 30-200 nm (Figure 4A-F). We estimated the number of nanospheres between 10^6 ^and 10^9 ^per mL of CSF, and their morphology was similar to that of synaptic vesicles and exosomes. Some spherical forms were more electron-dense (Figure 4D-F), similar in appearance to LDCVs [25].

Other frequent structure types included irregular-shaped, non-circular, electron-dense blobs (upward-pointing arrows, Figure 4G) and strands (downward-pointing arrow, Figure 4G). The irregularity of the blobs suggests they represent degraded electron-dense spheres, soluble N-ethylmaleimide-sensitive fusion attachment protein receptor (SNARE) complexes [26], or some other non-specific debris. The strands resembled long-distance membranous nanotubes [27]. Both blob- and strand-like particles were between 30 and 200 nm, and were enriched in P3 compared to S3. The frequency of the blob- and strand-like particles varied considerably between samples, but sample numbers were too small to distinguish reliable trends for specific brain conditions.

Two other structures comprised < 1% of observed structures in P3. Lobular masses on stalks (Figure 4H) had identical morphology to structures, previously reported to protrude from the ventricular floor into the CSF [1,28]. Fragments (Figure 4J) resembled cilia that have also been visualized on the ventricular floor [1,28]. The lobular and cilia-like structures are probably cellular debris, originating by fragmentation off the choroid epithelium, subarachnoid or ventricular linings.

### Protein composition of nanostructures

**Figure 5:**
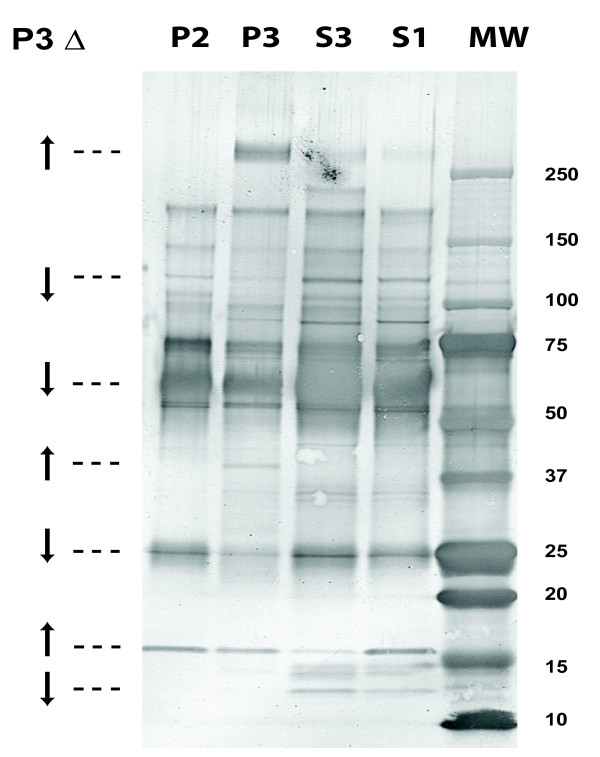
**An electrophoretic profile of P2, P3, S3, and S1 CSF fractions, representative of sample #s 4, 6-12, 14-16**. Molecular weights of standards are on the right (MW). Arrows in the P3Δ column indicate proteins that were either enriched or depleted in P3 samples compared to S3s. The fractions S1 and S3 (and to a lesser extent P2) have similar profiles.

To screen the protein composition of the nanostructures, we compared electrophoretic profiles of fractions from 11 people from sample #s 4, 6-12, 14-16 (Table 1). Figure 5 illustrates consistent changes in P3 proteins: Several protein bands were enriched (↑) while others were depleted (↓) in P3 compared with S3 fractions; in contrast, protein bands from S1 and S3 fractions had similar looking profiles. These data show that the protein composition of P3 differs from that of the supernatants S1 and S3.

**Figure 6:**
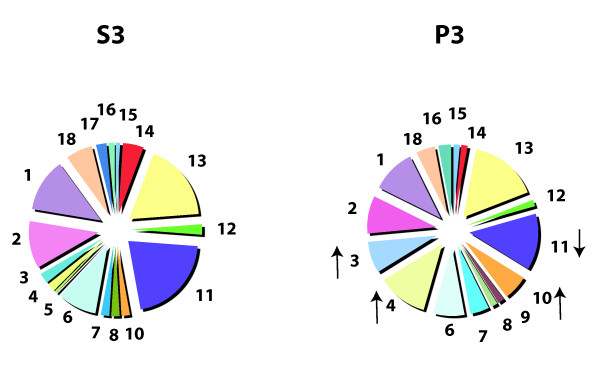
**Pie charts of total CSF proteins identified by high resolution liquid chromatography mass spectrometry from a single shotgun sequencing run of S3 and P3 fractions of two different samples #s 3 and 3' from one participant (Table 1)**. Data was pooled for both P3 and S3 fractions, and charted as S3 or P3 GO components (same code as in Figure 2). There are a number of differences between the P3 and S3 fractions (for complete protein lists see Additional File 2). The GO components that differentiate the two fractions have directional arrows, from which it is clear that there are more structural (GO #s 4 & 10) and "other" category proteins (GO # 3), and less extracellular proteins (GO # 11) in P3 fractions.

To investigate which proteins differ between P3 and S3, we performed LCMS experiments using lumbar CSF sampled on two separate occasions from the same person, diagnosed with migraine (sample #s 3 and 3', Table 1). We found that while the protein GO categorized components are consistent for each replicate of P3 and S3, we see distinct differences (Figure 6, additional file 2). More organelle-based and filament/tubule/"other" GO category proteins and fewer extracellular proteins were present in P3 than in S3. Moreover, the GO categories of S3 were similar to the extensive S1 studies: compare S3 in Figure 6 with the GO components in the larger S1 studies in Figure 2.

**Figure 7:**
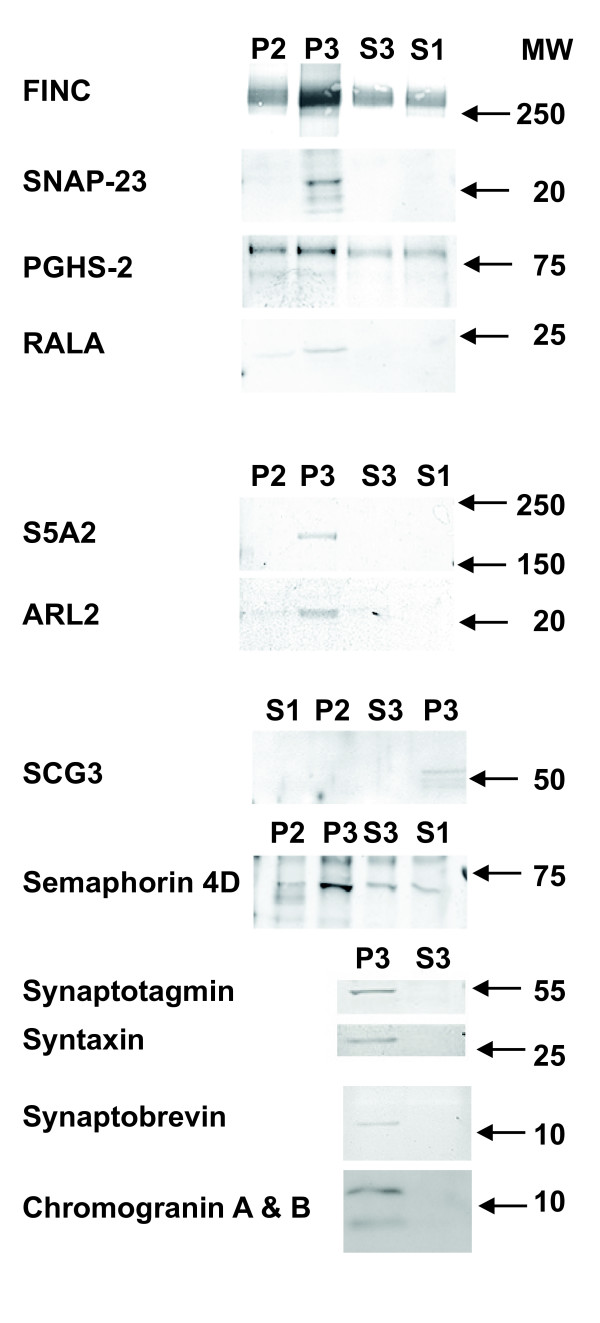
**Western blots of P2, P3, S3, and S1 fractions based on antibodies against 13 different proteins**. This blot composite is representative of four samples for the top seven images and of two samples for the remaining six proteins, all selected randomly from sample #s 4, 6-12, 14-16 (Table 1). Molecular weights from relevant standards are indicated on the right. For all 13 reactivities, the signal was enriched in P3 fractions. FINC: fibronectin, SNAP-23: synaptosomal-associated protein 23, PGHS-2: prostaglandin H synthase, RALA: ras-related protein Ral-A, S5A2: 3-oxo-5-alpha-steroid 4-dehydrogenase, ARL2: ADP-ribosylation factor-like protein 2, SCG3: secretogranin 3.

To explore the sources of these structures, we considered that the abundant spherical structures would come from the choroid plexuses and the ependymal lining of the CSF, and might be derived from similar-sized synaptic vesicles [29], LDCVs [30], or exosomes [31], while the blob- and strand-like structures may be derived from extracellular matrix proteins [32]. Nerve terminals known to border the CSF [1] could discharge synaptic vesicles and LDCVs into the CSF. Neighboring cells could also discharge exosomes. While such processes have not been defined for CSF, exosomes have been reported in urine [14], blood [33], and brain extracellular fluid [34]. Accordingly, we performed Western blot analysis on CSF fractions from sample #s 4, 6-12, 14-16, using antibodies against proteins we have identified by LCMS. We used fractions from four different participants for each antibody in 7/13 studies and two participants for the other 6 antibodies, chosen randomly from the same 11 different participants, as for electrophoresis. We found 13 proteins are enriched in P3s (Figure 7). Seven proteins are significantly enriched in P3 (n = 3 or 4, *p *< 0.05, paired analysis), based on specific staining at their predicted molecular weight: FINC, SNAP 23, PGHS-2, RALA, S5A2, ARL2, and SCG3. We also found that semaphorin 4D, chromogranin A & B, and the known SNARE complex proteins synaptotagmin, syntaxin, and synaptobrevin were enriched in P3, but with insufficient samples for statistical analysis.

### Nanostructure protein and neurotransmitter localization

**Figure 8:**
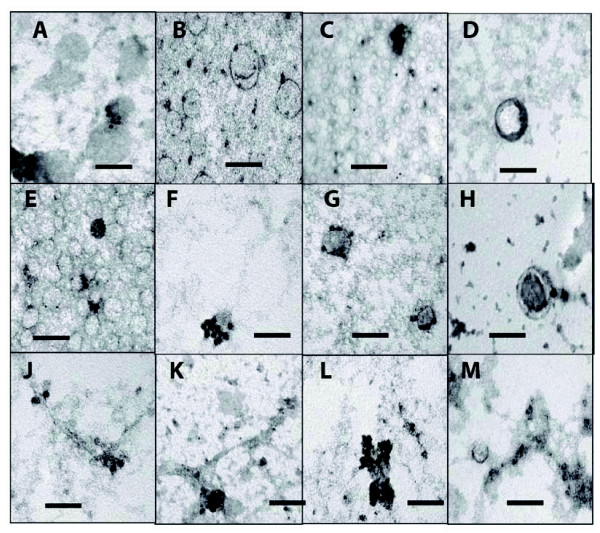
**Immuno-TEM of ultrastructures from P3 fractions of CSF, representative of sample #s 4-19**. The 12 and 18 nm gold particles are visible in all structures, specific for the eight antisera tested (negative controls with pre-immune sera only had occasional random gold particles): Nanospheres are specifically labeled with A: prostaglandin H synthase 2; B, D, H: ras-related protein Ral-A; C: ADP-ribosylation factor-like protein 2; E: synaptosomal-associated protein 23; F: secretogranin 3; and G: acetylcholine. Nanostrands and nanoblobs are labeled with J: secretogranin 3; K: 3-oxo-5-alpha-steroid 4-dehydrogenase; and L, M: fibronectin. Scale bars 100 nm.

Since nanostructures are purified in P3 fractions, we performed iTEM to find whether the proteins identified on western blots (Figure 7) localize to specific structures (Figure 8). Using P3s from sample #s 4-19 (Table 1), we tested at least four different participant samples with each antibody: against PGHS-2 (Figure 8A), RALA (8B, D, H), ARL2 (8C), SNAP 23 (8E), and SCG3 (8F) specifically bound the spherical structures. These structures did not stain when the primary antibodies were replaced with pre-immune sera. To explore whether neurotransmitters are present, we found that anti-acetylcholine specifically stained spheres 80-100 nm in diameter (Figure 8G). These iTEM results demonstrate extensive molecular heterogeneity within the spheres, since only a proportion of them on each grid region stained with each antibody. In the case of PGHS-2, we only saw staining when two spheres were beside each other (Figure 8A). In the case of acetylcholine we only saw staining of 80-100 nm diameter spheres (Figure 8G), not of smaller ones (30-50 nm), in keeping with a size-regulated subpopulation of neurotransmitter packages. Blob- and strand-like particles had specific iTEM staining for SCG3 (Figure 8J), S5A2 (8K), and FINC (8L &8M). FINC is one of the most abundant molecules in P3 by western blot (Figure 7), and by the number of peptide ions detected by mass spectrometry (data not shown).

### Lipid membrane composition of nanostructures

**Figure 9:**
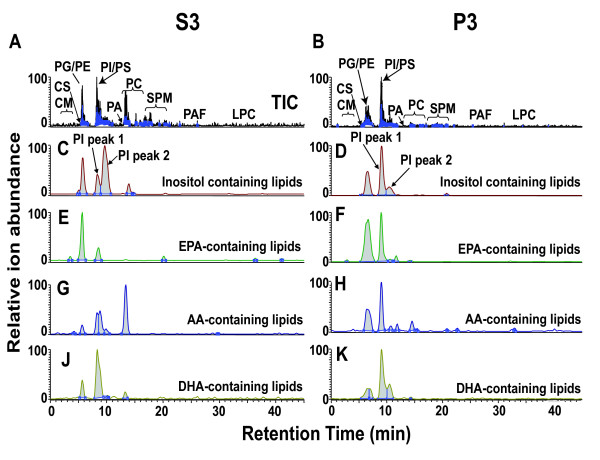
**Lipid liquid chromatography mass spectrometry data, representative of four analyses from separate CSF samples**. Unique lipid components differentiate P3 and S3 preparations. Total ion profiles (TIC) for A: S3, and B: P3 show the elution profile from the normal phase column for lipids: PG/PE: phosphatidylglycerol/phosphatidylethanolamine and PC: phosphatidylcholine were decreased in P3 fractions. CS: cerebroside; CM: ceramide; PI: phosphatidylinositide; PS: phosphatidylserine; PA: phosphatidic acid; SPM: sphingomyelin; PAF: platelet-activating factor; LPC: lysophosphatidylcholine. C and D: The inositol polyunsaturated fatty acid-containing lipids are greatly increased (Peak 1) in the P3 fractions. The omega 3 polyunsaturated fatty acids are increased in P3 fraction, E and F: EPA: eicosanpentanoeate, G and H, and DHA: docosahexaenoate J and K. The omega-6 polyunsaturated fatty acid is decreased, AA: arachidonic acid. The ratio of omega-3/omega-6 is significantly higher in P3 fractions (*p *= 0.0005).

The morphology of the abundant spheres visible by TEM (Figure 4A-F) suggests a membrane perimeter. Since membranes are composed mainly of lipids, we extracted lipids from S3 and P3 fractions from sample #s 14-17 in Table 1 with organic solvents and analyzed them by normal phase chromatography and tandem mass spectrometry. We identified several lipid classes by LCMS and these clearly differed between S3s and P3s, as illustrated by the total ion current chromatograph (TIC, Figure 9A &9B), including phospholipids (PE, PI, PS, PC, PG, PA) and sphingolipids (CE, CB, and SPM). To evaluate whether we could disrupt the apparent phospholipid membranes of the nanostructures in P3, we incubated P3 with PLA_2 _(*Naja mossambica*). This depleted the major phospholipid peaks as measured by mass spectrometry, and removed > 90% of the spherical structures as measured by TEM (data not shown).

Liquid chromatography of S3 and P3 lipids partially resolved PI into two peaks consisting of polyunsaturated fatty acids (PUFA), (PI-peak-1), and saturated fatty acids, (PI-peak-2), (Figure 9C &9D). The PI ratio of peak 1/peak 2 in S3 (0.3 ± 0.026; n = 4) was lower than in P3 (3.1 ± 0.26, n = 4) (*p *< 0.005).

The presence of eicosanoid metabolic enzymes (see additional files 1 &2) and their substrates in P3 (Figure 9A &9B) suggested that these abundant small spheres play active metabolic roles. Accordingly, we identified omega-6 PUFA (AA) and omega-3 PUFA (DHA and EPA) in the phospholipid classes (Figure 9E - K). The ratio of omega-3/omega-6 in S3 (1.17 ± 0.199, n = 4) was lower than in P3 (6.2 ± 0.815, n = 4) (*p *= 0.0005).

### Enzyme activity in nanostructures

To test whether CSF nanostructures have functional enzymes, we selected the prostanoid pathway because our protein and lipid studies showed both their enzymes and substrates present in CSF. Furthermore, Hayaishi's group demonstrated a physiological effect for prostaglandin D_2 _(PGD_2_) in the initiation of normal sleep, when it is applied *via *the CSF selectively to the ventral rostral brainstem [9], and many studies have reported roles for prostaglandins within the brain [35-37]. However, prostaglandin H_2 _(PGH_2_,) is an unstable intermediate in PGD_2_synthesis, and its local synthesis by the integral membrane protein PGHS is required for production of downstream prostaglandins. We therefore assayed PGHS activity in the CSF fractions.

**Figure 10:**
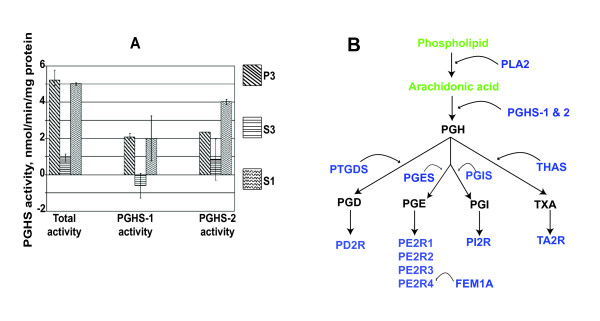
**Prostanoid regulation in CSF**. A: prostaglandin H synthase (PGHS-1 and PGHS-2) activity assays for P3, S3, and S1 from two different study participants, analyzed for a total of at least three measures per fraction, with standard error bars. Both PGHS-1 & PGHS-2 specific activities are demonstrated in P3 and S1 versus S3 fractions, compared to baseline activities without inhibitor. This demonstrates the presence of specific PGHS-1 and -2 activities in the S1 fractions that are enriched in the P3 (and decreased in the S3) fractions. B: Scheme for prostanoid enzymes, receptors, and regulators identified in CSF by shotgun liquid chromatography mass spectrometry (blue) and substrates identified by LCMS in SRM mode (green). Prostaglandins were not identified in this study (black). This diagram outlines CSF components capable of extensive prostanoid synthesis, with receptors and regulators, including the functional enzymes PGHS-1 and -2 (Figure 10A), the critical source of prostaglandin H_2 _(PGH_2_). PLA2: phospholipase A_2_; PTGDS: prostaglandin D synthase; PGES: prostaglandin E synthase; PGIS: prostaglandin I synthase; THAS: thromboxin A synthase; PGD: prostaglandin D; PGE: prostaglandin E; PGI: prostaglandin I; TXA: thromboxane A; PD2R: prostaglandin D2 receptor; PE2R1, 2, 3, 4: prostaglandin E1, 2, 3, 4 receptors; PI2R: prostaglandin I2 receptor; TA2R: thromboxane A2 receptor; FEM1A: Prostaglandin E receptor 4-associated protein.

We wanted to test PGHS activity from a large quantity of CSF because PGHS-2 staining structures were infrequent on iTEM (Figure 8A) and P3 western blots for PGHS-2 were faint (Figure 7). Accordingly, we used 90 mL of ventricular CSF from two patients with congenital hydrocephalus from sample #s 18 & 19 (both clinically stable, with normal pressure and total protein, and free from infection). We tested fractions from each person separately and in duplicate and found activity in S1 and P3 that was reduced or absent in S3; inhibitor studies reveal that the activity was from both PGHS-1 and PGHS-2 (Figure 10A).

## Discussion

A variety of experiments, as outlined in the background, support volume transmission between brain regions *via *the CSF. However, there is little information about the mechanisms by which such communications are regulated. A lack of structures to transport, protect, localize, and eventually transduce signals at an appropriate receptor poses major restrictions on transmission within the 150 mL of fluid. We now report details of the morphology and biochemistry of unique structures in CSF that have the potential to overcome many, if not all, of these limitations.

CSF proteomic studies have recently revealed many proteins that are substantially different from those found in plasma [23,38-41]. Our protein lists (see additional files 1 &2) are complementary to these in overall components, and further illuminate CSF protein composition. We view these data sets as valuable references to design experiments for the study of CSF proteins.

### CSF membrane proteins

When classes of CSF protein components from published data were compared based on GO component categories, around 40% are membrane proteins [23]. Zougman and colleagues suggest that this abundance of membrane protein identifications is the consequence of extensive protease actions that cleave fragments from proteins that are embedded in membranes, and these fragments enter the CSF [23]. An alternative explanation is that there are abundant membranous structures in CSF. In this paper, we concentrate on membrane proteins, since their large percentage suggests the possibility that there are membranes within the CSF.

### CSF lipid membrane bound nanostructures

Our TEM data demonstrates small particles on filtered CSF. The presence of these nano-sized particles from filtration provides evidence that these structures are not formed during the ultracentrifugation procedure. The presence of identical nanostructures in CSF from two participants that were collected and prepared freshly as compared to stored CSF, demonstrates that the nanostructures are not a product of sample storage.

Past reports of CSF sub-cellular structures have been interpreted as resulting from blebbing, apocrine secretion, apoptosis events, or cellular debris [1,42-45] and have limited biochemical characterization, though one extensive protein composition has been reported from CSF ultracentrifuge preparations of mouse and human embryonic CSF [40]. Our ultracentrifugation preparations confirm that CSF has abundant structures between 30-200 nm in size that are enriched in P3 in all 16 samples tested.

Morphologically, the majority are nanospheres, the next most abundant are irregular nanoblobs, and there is only occasional nanodebris that most likely represent structures that have fractured from the CSF linings. Our data are consistent with these earlier reports, but the enriched nanostructure suspensions have enabled further morphological, protein, lipid, and enzyme characterizations. Our observations that PLA_2 _caused dissolution of the TEM nanostructures, and depleted major P3 phospholipids as determined by LCMS, demonstrate that lipid membranes enclose these spherical structures. These membranes provide an appropriate environment for some of the abundant transmembrane proteins found in our shotgun sequencing experiments of CSF (Figure 2 and additional files 1 &2).

Even though the numbers examined by TEM are limited to 16 persons, the presence of these nanostructures in CSF from people without any brain disorder (healthy persons), persons with intermittent disability (migraine), and persons with serious brain pathology of either an inflammatory (multiple sclerosis) or degenerative disease (Alzheimer's disease) suggests strongly that these structures are ubiquitous in CSF. Though the unknown functions of these nanostructures require much further study, our data hints at multiple roles.

### Nanostructure composition suggests functions

The different electrophoretic and western blots of P3s in this nanostructure-rich fraction are evidence that the P3 fraction from CSF contains biochemically distinct components. The FINC immunostain is greatly enriched in P3 fractions that mainly contain the varied blobs and strands. The known functions of FINC as an adhesive extracellular molecule involved in neurite development [46] support an important connective role for these nano-sized blobs and strands within CSF. Acetylcholine and SNAP 23 are present in spheres and, along with the SNARE complex proteins synaptobrevin, synaptotagmin, and syntaxin (Figure 7), these data provide evidence that synaptic vesicles and/or LDCVs exist in CSF. TEM images of SNARE fusion proteins [47] reveal structures similar to the smaller CSF blobs but, while we found SNARE complex proteins are enriched in P3 fractions, further study is needed to evaluate their role in CSF. Some spheres contain the exosome-associated protein RALA (Figures 8B, D, H)[48], evidence that they may be exosomes. Some spheres may represent synaptic vesicles and some LDCVs. The LDCVs, with their cargoes of lipids, peptides, and proteins, may participate in receptor-mediated uptake, as is known to occur in brain with β-amyloid [49]. Overall, the functions of these different molecules suggest that structurally discrete spheres may have neurotransmission and signal transduction/regulatory activities.

Our data (Figure 9) also show that P3 has unique lipids, composed of more unsaturated PI, and more total omega-3 than omega-6 PUFAs. The known functions (receptors, neuroprotection, signaling molecules) of PUFAs in brain [50] suggest that the P3-enriched PUFAs in the membranes of the spherical CSF structures have potential transport, neuroprotection, or signaling roles [51]. Increased signaling PI lipids in spheres, along with GTPase-activating proteins such as Arl2, reflect the capacity for immediate activation of signal transduction and vesicle regulation [52]. Moreover, the greater prevalence of omega-3 PUFAs in CSF nanostructures will help to buffer inflammatory processes around the brain, since omega-3s are precursors of anti-inflammatory, pro-resolving, signaling molecules [51].

We have demonstrated that the critical enzymes (PGHS-1 & -2) for synthesis of the obligatory prostanoid intermediate, PGH_2_, are present (Figures 7 &8A) and active (Figure 10A) in CSF. PGHS 1 & 2 are the only integral membrane proteins whose presence have been confirmed in these nanostructures, thus it is premature to predict what proportion of the many other CSF membrane proteins are intact and functional versus cleaved products [23]. We have also identified structures of the prostanoid pathway (Figure 10B). These range from phospholipids (characterized by LCMS, Figure 9A-K) and lipases, synthases, and receptors (identified by LCMS, see additional files 1 &2). This is the first data to demonstrate the ability to biosynthesize prostanoids within CSF, a capability that supports a humoral role for these known lipid mediators, such as that proposed for sleep [9]. Further research is needed to establish the roles for these pathways in the CSF/brain system.

Membrane-bound nanospheres with acetylcholine provide potential protection from acetylcholinesterase in CSF, thus enabling a humoral mode for vesicle neurotransmission *via *CSF. For example, topical acetylcholine has been consistently shown to dilate cerebral vessels [53], raising the possibility that the acetylcholine we identified in spheres, if protected from premature anticholinesterase inactivation, may influence cerebral vasomotor tone.

### Nanostructure abundance

For both healthy as well as those with brain disorders we estimate, assuming production and turn over at the same rate as the fluid, between 10^9 ^and 10^12 ^nanospheres are produced per day, comprised of 5 μg total protein. These relatively small amounts of CSF nanostructures may have physiological roles since the readily releasable synaptic vesicle pool size ranges from as low as 5 to as many as 5,000 per single synapse, as reviewed by Südhof [54]. While the presence of biochemically active species in the nanostructures is clear from our data, the lack of knowledge of their absolute amounts, or their production, turnover, metabolism, and clearance necessitates further studies to determine their functions.

### Nanostructure circulation (Figure 11)

**Figure 11:**
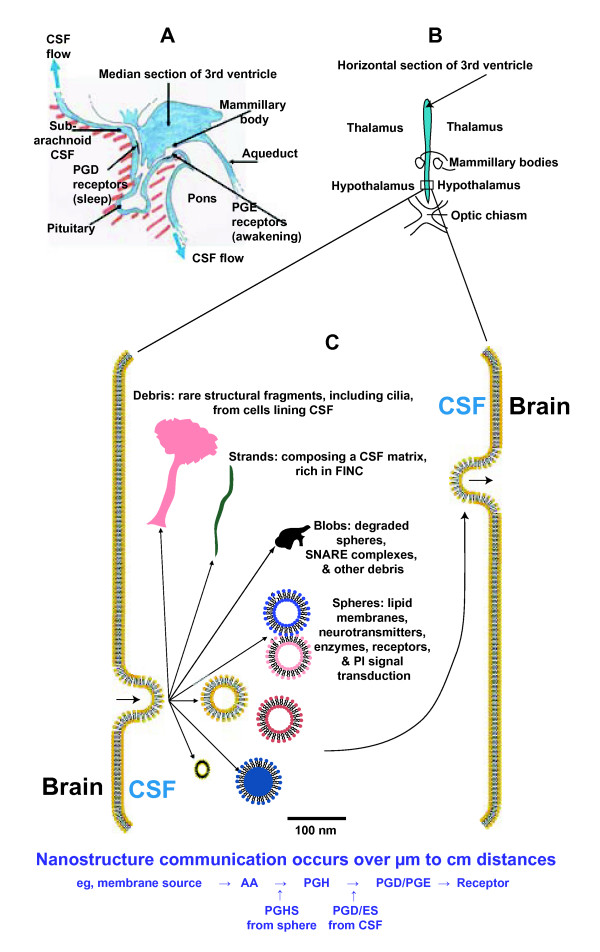
**Proposed transport model for CSF nanostructures around the 3rd ventricle, with CSF neuroanatomical relations**. A: Subarachnoid flow is directed from the pituitary fossa region, as indicated by arrows. The subarachnoid areas indicated for prostaglandin PGD and PGE receptors are quite discretely localized. CSF within the 3^rd ^ventricle is separated by a small amount of tissue from the subarachnoid CSF areas, but these fluid compartments are only contiguous through the foraminal exits of Luschka and Magendie over considerable distance *via *the cisterna magna and back over the subarachnoid spaces. The boxed region in B is enlarged in C, which illustrates our proposal that nanospheres and nanoblobs (and the much less common nanodebris) arise directly from the brain/choroid/CSF surface, circulate, and contact a remote brain region or another nanostructure for signal transmission, probably regulated with specific receptor mechanisms.

Brain extracellular fluid is known to diffuse ions, dopamine, proteins, and 35 nm particles [55,56] in spaces up to 64 nm. These extracellular spaces are large enough for the smaller nanostructures, but structures greater than 100 nm are more likely to be produced at the CSF walls, rather than from within brain tissues. Considerable variations in CSF formation and flow velocities occur, depending on locations within the neuraxis. We estimate flow for CSF nanostructures, based on the known CSF formation rate approximating 0.4 mL per minute, and maximum flow velocity in the 3^rd ^ventricle of about 5 mm/sec [57,58]. Figure 11 outlines a transport model for nanostructures formed in the 3^rd ^ventricle to move to receptors in the medial hypothalamic wall of the same ventricle. For a simple circulation of pre-synthesized signal within this small space, we estimate transmission speed will be in the order of a second. At the other extreme, such as nanostructures originating in a ventricle, migrating to a more distant subarachnoid location, and requiring signal synthesis, we estimate speeds ranging from minutes to several hours, since total CSF exchanges 3-5 times a day and is stagnant in some regions [57,58]. Bulk flow of CSF nanostructures thus generates a more dispersed signal delivery, of longer duration. These varied transmission speeds are all slower by orders of magnitude than synaptic transmission (milliseconds). Slower spatial and temporal signaling involving CSF nanostructures may regulate brain behaviors known to require slower, more gradual, and more sustained modulations, such as reported for sleep, appetite, mood, and vasomotor regulation [4-7,53,59].

## Conclusion

Human lumbar and ventricular CSF samples demonstrate abundant membranous CSF structures, 30-200 nm in size from both healthy and sick participants. Compared to the supernatant, these structures have unique protein and lipid compositions, contain acetylcholine, and have complete prostanoid pathways from membrane phospholipids to specific receptors. Variation in CSF nanostructures may be informative in both health and disease studies, but they require enrichment since they are diluted by more abundant fluid components. We anticipate that further study of these biochemically and morphologically unique CSF nanostructures will identify their roles in modulating brain functions and dysfunctions.

## Abbreviations

AA: arachidonic acid; ARL2: ADP-ribosylation factor-like protein 2; BSA: bovine serum albumin; CM: ceramide; CS: cerebroside sulfate: CSF: cerebrospinal fluid; DHA: docosahexaenoate; EPA: eicosanpentanoeate; FINC: fibronectin; GO: gene ontology; LCMS: liquid chromatography mass spectrometry; LDCV: large dense core vesicle; LPC: lysophosphatidylcholine; NIL neutral ion loss; P1: CSF 3,000 g pellet; P2: CSF 17,000 g pellet; P3: CSF 200,000 g pellet; PA: phosphatidic acid; PAF: platelet-activating factor; PBS phosphate buffered saline; PC: phosphatidylcholine; PE: phosphatidylethanolamine; PG: phosphatidylglycerol; PGD_2_: prostaglandin D_2_; PGH_2_: prostaglandin H_2_; PGHS: prostaglandin H synthase; PI: phosphatidylinositide; PIS: precursor ion scan; PLA_2_: phospholipase A_2_; PMSF: phenylmethanesulphonylfluoride; PS: phosphatidylserine; PTGDS: prostaglandin D synthase; PUFA: polyunsaturated fatty acid: PVDF: polyvinylidine difluoride; RALA: Ras-related protein ral-A; S1: CSF 3,000 g supernatant; S2: CSF 17,000 g supernatant; S3: CSF 200,000 g supernatant; S5A2: 3-oxo-5-alpha-steroid 4-dehydrogenase 2; SCG3: secretogranin 3; SE-1: statistical expectation value; SNAP-23: synaptosomal-associated protein 23; SNARE: soluble N-ethylmaleimide-sensitive fusion attachment protein receptor; SPM: sphingomyelin; TEM: transmission electron microscopy (iTEM: immuno-TEM).

## Competing interests

The authors declare that they have no competing interests.

## Authors' contributions

MGH conceived and designed both the human subjects and laboratory aspects of the project, recruited and diagnosed study participants, collected some of the CSF, carried out some of the shotgun protein sequencing experiments, performed the ultracentrifugation and sample preparations for biochemical and electron microscopy studies (TEM and iTEM), analyzed all data, and drafted the manuscript. ANF participated in design of all experiments, carried out the lipid analyses, and participated in manuscript revisions. EO carried out electrophoresis, western blots, developed and carried out the enzyme activity assays. PL carried out the early ultrafiltration of nanostructures and participated in the electron microscopy and analysis of these filters. RPC and GM recruited and diagnosed study participants, collected CSF, and participated in manuscript revisions. JNC helped design and supervise all electron microscopy experiments. RGB and AFH helped design and carry out most of the protein shotgun sequencing experiments, and participated in manuscript revisions. All authors read and approved the final manuscript.

## Supplementary Material

Additional file 1 **CSF proteins from repeated analyses of two independent samples**. CSF proteins identified by Uniprot number, name, and GO component category, from 15 shotgun sequencing analyses of sample #s 1 & 2 in Table 1. Click here for file

Additional file 2 **CSF proteins from the P3-enriched pellet and the S3 supernatant**. CSF proteins identified from a single, high resolution shotgun sequencing run of two sets of P3 and S3 samples from one participant collected on two independent occasions (sample #s 3 & 3' in Table 1). Click here for file
