# Supplementary material for: The morphology and biochemistry of nanostructures provide evidence for synthesis and signaling functions in human cerebrospinal fluid
Source: Cerebrospinal Fluid Res. 2009 Sep 7;6:10. doi: 10.1186/1743-8454-6-10 (PMC2746175; doi:10.1186/1743-8454-6-10)
Supplement: Additional file 1 — CSF proteins from repeated analyses of two independent samples. CSF proteins identified by Uniprot number, name, and GO component category, from 15 shotgun sequencing analyses of sample #s 1 & 2 in Table 1. [file 1743-8454-6-10-S1.pdf]

Supplementary Table 1. Proteins from Total CSF from repeated analyses of two independent samples (Data 1 and Data 2).

The two CSF samples were obtained from two different participants with Alzheimer's disease, as described in the Methods and Table. CSF proteins were prepared, treated with trypsin, fractionated, and analyzed using different formats of mass spectrometry in order to get as many different proteins confidently identified as possible, as described in the Methods. Proteins are ordered by Sample 1 followed by Sample 2, and within each sample, they are ordered by the protein GO component assignment in alphabetical order

Uniprot number	Data 1 Protein Name	Component_CATEGORY
Q02224	Centromeric protein E (CENP-E).	Chromosomal
Q12948	[Forkhead box protein C1 (Forkhead-related protein FKHL7) (Forkhead-, related transcription factor 3) (FREAC-3).]	Chromosomal
Q93077	Histone H2A type 1-C.	Chromosomal
P06400	Retinoblastoma-associated protein (PP110) (P105-RB) (RB).	Chromosomal
P46100	[Transcriptional regulator ATRX (EC 3.6.1.-) (ATP-dependent helicase, ATRX) (X-linked helicase II) (X-linked nuclear protein) (XNP) (Znf-, HX).]	Chromosomal
Q13112	[Chromatin assembly factor 1 subunit B (CAF-1 subunit B) (Chromatin, assembly factor I p60 subunit) (CAF-I 60 kDa subunit) (CAF-Ip60) (M-, phase phosphoprotein 7).]	Chromosomal
P03372	Estrogen receptor (ER) (Estradiol receptor) (ER-alpha).	Chromosomal
P49736	[DNA replication licensing factor MCM2 (Minichromosome maintenance, protein 2 homolog) (Nuclear protein BM28).]	Chromosomal
Q13111	[Chromatin assembly factor 1 subunit A (CAF-1 subunit A) (Chromatin, assembly factor I p150 subunit) (CAF-I 150 kDa subunit) (CAF-Ip150).]	Chromosomal
P07199	Major centromere autoantigen B (Centromere protein B) (CENP-B).	Chromosomal
O14757	Serine/threonine-protein kinase Chk1 (EC 2.7.11.1).	Chromosomal
P33993	DNA replication licensing factor MCM7 (CDC47 homolog) (P1.1-MCM3).	Chromosomal
Q92900	[Regulator of nonsense transcripts 1 (EC 3.6.1.-) (ATP-dependent, helicase RENT1) (Nonsense mRNA reducing factor 1) (NORF1) (Up-, frameshift suppressor 1 homolog) (hUpf1).]	Chromosomal
P02730	[Band 3 anion transport protein (Anion exchange protein 1) (AE 1), (Solute carrier family 4 member 1) (CD233 antigen).]	Cytoskeletal
P80723	[Brain acid soluble protein 1 (BASP1 protein) (Neuronal axonal membrane, protein NAP-22) (22 kDa neuronal tissue-enriched acidic protein).]	Cytoskeletal
O00499	[Myc box-dependent-interacting protein 1 (Bridging integrator 1), (Amphiphysin-like protein) (Amphiphysin II) (Box-dependent myc-, interacting protein 1).]	Cytoskeletal
Q13939	Calicin.	Cytoskeletal
Q9Y696	[Chloride intracellular channel protein 4 (Intracellular chloride ion, channel protein p64H1).]	Cytoskeletal
Q9UQ03	Coronin-2B (Coronin-like protein C) (Clipin-C) (Protein FC96).	Cytoskeletal
P53355	Death-associated protein kinase 1 (EC 2.7.11.1) (DAP kinase 1).	Cytoskeletal
Q16658	Fascin (Singed-like protein) (55 kDa actin-bundling protein) (p55).	Cytoskeletal
P54840	Glycogen [starch] synthase, liver (EC 2.4.1.11).	Cytoskeletal

O00291	Huntingtin-interacting protein 1 (HIP-I).	Cytoskeletal
Q9Y573	Actin-binding protein IPP (MIPP protein) (Kelch-like protein 27).	Cytoskeletal
Q9C0H6	Kelch-like protein 4.	Cytoskeletal
P29966	[Myristoylated alanine-rich C-kinase substrate (MARCKS) (Protein kinase, C substrate, 80 kDa protein, light chain) (PKCSL) (80K-L protein).]	Cytoskeletal
P11137	Microtubule-associated protein 2 (MAP 2) (MAP-2).	Cytoskeletal
Q13459	Myosin-IXb (Unconventional myosin-9b).	Cytoskeletal
P19338	Nucleolin (Protein C23).	Cytoskeletal
P07437	Tubulin beta chain (Tubulin beta-5 chain).	Cytoskeletal
P46939	Utrophin (Dystrophin-related protein 1) (DRP1) (DRP).	Cytoskeletal
O75366	Advillin (p92).	Cytoskeletal
P62330	ADP-ribosylation factor 6.	Cytoskeletal
O00423	Echinoderm microtubule-associated protein-like 1 (EMAP-1) (HuEMAP-1).	Cytoskeletal
P06396	[Gelsolin precursor (Actin-depolymerizing factor) (ADF) (Brevin), (AGEL).]	Cytoskeletal
Q13576	Ras GTPase-activating-like protein IQGAP2.	Cytoskeletal
O95198	Kelch-like protein 2 (Actin-binding protein Mayven).	Cytoskeletal
P78559	[Microtubule-associated protein 1A (MAP 1A) (Proliferation-related, protein p80) [Contains: MAP1 light chain LC2].]	Cytoskeletal
P55083	Microfibril-associated glycoprotein 4 precursor.	Cytoskeletal
P26038	Moesin (Membrane-organizing extension spike protein).	Cytoskeletal
Q9UM54	Myosin-VI (Unconventional myosin VI).	Cytoskeletal
P20929	Nebulin.	Cytoskeletal
P53814	Smoothelin.	Cytoskeletal
O95793	Double-stranded RNA-binding protein Staufen homolog 1.	Cytoskeletal
Q9UBN4	[Short transient receptor potential channel 4 (TrpC4) (Trp-related, protein 4) (hTrp-4) (hTrp4).]	Cytoskeletal
O15195	Villin-like protein.	Cytoskeletal
O43516	[WAS/WASL-interacting protein family member 1 (Wiskott-Aldrich syndrome, protein-interacting protein) (WASP-interacting protein) (PRPL-2, protein).]	Cytoskeletal
P42768	Wiskott-Aldrich syndrome protein (WASp).	Cytoskeletal
Q9Y6U3	Adseverin (Scinderin).	Cytoskeletal
Q05682	Caldesmon (CDM).	Cytoskeletal
Q14118	[Dystroglycan precursor (Dystrophin-associated glycoprotein 1), [Contains: Alpha-dystroglycan (Alpha-DG); Beta-dystroglycan (Beta-, DG)].]	Cytoskeletal
O95834	Echinoderm microtubule-associated protein-like 2 (EMAP-2) (HuEMAP-2).	Cytoskeletal
P58107	Epiplakin (450 kDa epidermal antigen).	Cytoskeletal
P46821	[Microtubule-associated protein 1B (MAP 1B) [Contains: MAP1 light chain, LC1].]	Cytoskeletal
P10916	[Myosin regulatory light chain 2, ventricular/cardiac muscle isoform, (MLC-2) (MLC-2v).]	Cytoskeletal
P35580	[Myosin-10 (Myosin heavy chain 10) (Myosin heavy chain, nonmuscle IIb), (Nonmuscle myosin heavy chain IIb) (NMMHC II-b) (NMMHC-IIB) (Cellular, myosin heavy chain, type B) (Nonmuscle myosin heavy chain-B) (NMMHC-, B).]	Cytoskeletal

Q15121	[Astrocytic phosphoprotein PEA-15 (Phosphoprotein enriched in diabetes), (PED).]	Cytoskeletal
Q16825	[Tyrosine-protein phosphatase non-receptor type 21 (EC 3.1.3.48), (Protein-tyrosine phosphatase D1).]	Cytoskeletal
Q07283	Trichohyalin.	Cytoskeletal
P50552	Vasodilator-stimulated phosphoprotein (VASP).	Cytoskeletal
Q9Y6W5	[Wiskott-Aldrich syndrome protein family member 2 (WASP-family protein, member 2) (Protein WAVE-2) (Verprolin homology domain-containing, protein 2).]	Cytoskeletal
P63104	[14-3-3 protein zeta/delta (Protein kinase C inhibitor protein 1), (KCIP-1).]	Cytosolic
P18848	[Cyclic AMP-dependent transcription factor ATF-4 (Activating, transcription factor 4) (DNA-binding protein TAXREB67) (Cyclic AMP, response element-binding protein 2) (CREB2).]	Cytosolic
P26641	Elongation factor 1-gamma (EF-1-gamma) (eEF-1B gamma).	Cytosolic
P06733	[Alpha-enolase (EC 4.2.1.11) (2-phospho-D-glycerate hydro-lyase) (Non-, neural enolase) (NNE) (Enolase 1) (Phosphopyruvate hydratase) (C-myc, promoter-binding protein) (MBP-1) (MPB-1) (Plasminogen-binding, protein).]	Cytosolic
Q14978	[Nucleolar phosphoprotein p130 (Nucleolar 130 kDa protein) (140 kDa, nucleolar phosphoprotein) (Nopp140) (Nucleolar and coiled-body, phosphoprotein 1).]	Cytosolic
P06733	[Alpha-enolase (EC 4.2.1.11) (2-phospho-D-glycerate hydro-lyase) (Non-, neural enolase) (NNE) (Enolase 1) (Phosphopyruvate hydratase) (C-myc, promoter-binding protein) (MBP-1) (MPB-1) (Plasminogen-binding, protein).]	Cytosolic
O95704	[Amyloid beta A4 precursor protein-binding family B member 3 (Fe65-like, protein 2) (Fe65L2).]	Cytosolic
P41227	[N-terminal acetyltransferase complex ARD1 subunit homolog A, (EC 2.3.1.88) (EC 2.3.1.-).]	Cytosolic
Q01814	[Plasma membrane calcium-transporting ATPase 2 (EC 3.6.3.8) (PMCA2), (Plasma membrane calcium pump isoform 2) (Plasma membrane calcium, ATPase isoform 2).]	Cytosolic
P56945	[Breast cancer anti-estrogen resistance protein 1 (CRK-associated, substrate) (p130cas).]	Cytosolic
Q14201	[Protein BTG3 (Tob5 protein) (Abundant in neuroepithelium area, protein).]	Cytosolic
P07858	[Cathepsin B precursor (EC 3.4.22.1) (Cathepsin B1) (APP secretase), (APPS) [Contains: Cathepsin B light chain; Cathepsin B heavy chain].]	Cytosolic
Q92793	CREB-binding protein (EC 2.3.1.48).	Cytosolic
Q08499	[cAMP-specific 3',5'-cyclic phosphodiesterase 4D (EC 3.1.4.17) (DPDE3), (PDE43).]	Cytosolic
P06850	[Corticoliberin precursor (Corticotropin-releasing factor) (CRF), (Corticotropin-releasing hormone).]	Cytosolic
P27797	Calreticulin precursor (CRP55) (Calregulin) (HACBP) (ERp60) (grp60).	Cytosolic
Q9Y5K3	[Choline-phosphate cytidylyltransferase B (EC 2.7.7.15), (Phosphorylcholine transferase B) (CTP:phosphocholine, cytidylyltransferase B) (CT B) (CCT B) (CCT-beta).]	Cytosolic
O00273	[DNA fragmentation factor subunit alpha (DNA fragmentation factor 45, kDa subunit) (DFF-45) (Inhibitor of CAD) (ICAD).]	Cytosolic
P00352	[Retinal dehydrogenase 1 (EC 1.2.1.36) (RalDH1) (RALDH 1) (Aldehyde, dehydrogenase family 1 member A1) (Aldehyde dehydrogenase, cytosolic), (ALHDII) (ALDH-E1).]	Cytosolic
O43237	[Cytoplasmic dynein 1 light intermediate chain 2 (Dynein light, intermediate chain 2, cytosolic) (LIC53/55) (LIC-2).]	Cytosolic
O00418	[Elongation factor 2 kinase (EC 2.7.11.20) (eEF-2 kinase) (eEF-2K), (Calcium/calmodulin-dependent eukaryotic elongation factor 2 kinase).]	Cytosolic
P14625	[Endoplasmin precursor (Heat shock protein 90 kDa beta member 1) (94, kDa glucose-regulated protein) (GRP94) (gp96 homolog) (Tumor rejection, antigen 1).]	Cytosolic
Q14289	[Protein tyrosine kinase 2 beta (EC 2.7.10.2) (Focal adhesion kinase 2), (FADK 2) (Proline-rich tyrosine kinase 2) (Cell adhesion kinase beta), (CAK beta) (Calcium-dependent tyrosine kinase) (CADTK) (Related, adhesion focal tyrosine kinase) (RAFTK).]	Cytosolic

O43524	[Forkhead box protein O3A (Forkhead in rhabdomyosarcoma-like 1) (AF6q21, protein).]	Cytosolic
P04406	Glyceraldehyde-3-phosphate dehydrogenase (EC 1.2.1.12) (GAPDH).	Cytosolic
P29992	[Guanine nucleotide-binding protein subunit alpha-11 (G alpha-11), (Guanine nucleotide-binding protein G(y) subunit alpha).]	Cytosolic
P15170	[G1 to S phase transition protein 1 homolog (GTP-binding protein GST1-, HS).]	Cytosolic
Q92769	Histone deacetylase 2 (HD2).	Cytosolic
P07900	[Heat shock protein HSP 90-alpha (HSP 86) (Renal carcinoma antigen NY-, REN-38).]	Cytosolic
Q05084	[Islet cell autoantigen 1 (69 kDa islet cell autoantigen) (ICA69) (p69), (Islet cell autoantigen p69) (ICAp69).]	Cytosolic
Q92985	Interferon regulatory factor 7 (IRF-7).	Cytosolic
P13861	cAMP-dependent protein kinase type II-alpha regulatory subunit.	Cytosolic
P19838	[Nuclear factor NF-kappa-B p105 subunit (DNA-binding factor KBF1) (EBP-, 1) [Contains: Nuclear factor NF-kappa-B p50 subunit].]	Cytosolic
Q9NR64	Kelch-like protein 1.	Cytosolic
P14618	[Pyruvate kinase isozymes M1/M2 (EC 2.7.1.40) (Pyruvate kinase muscle, isozyme) (Pyruvate kinase 2/3) (Cytosolic thyroid hormone-binding, protein) (CTHBP) (THBP1).]	Cytosolic
P07195	[L-lactate dehydrogenase B chain (EC 1.1.1.27) (LDH-B) (LDH heart, subunit) (LDH-H) (Renal carcinoma antigen NY-REN-46).]	Cytosolic
O60318	80 kDa MCM3-associated protein (GANP protein).	Cytosolic
Q9H000	Makorin-2 (RING finger protein 62).	Cytosolic
O14733	[Dual specificity mitogen-activated protein kinase kinase 7, (EC 2.7.12.2) (MAP kinase kinase 7) (MAPKK 7) (MAPK/ERK kinase 7), (JNK-activating kinase 2) (c-Jun N-terminal kinase kinase 2) (JNK, kinase 2) (JNKK 2).]	Cytosolic
Q9Y4I1	[Myosin-Va (Dilute myosin heavy chain, non-muscle) (Myosin-12) (Myosin, heavy chain 12) (Myoxin).]	Cytosolic
Q9NX02	[NACHT, LRR and PYD domains-containing protein 2 (PYRIN domain and, NACHT domain-containing protein 1) (PYRIN-containing APAF1-like, protein 2) (Nucleotide-binding site protein 1).]	Cytosolic
Q15080	[Neutrophil cytosol factor 4 (NCF-4) (Neutrophil NADPH oxidase factor, 4) (p40-phox) (p40phox) (SH3 and PX domain-containing protein 4).]	Cytosolic
Q9Y6K9	[NF-kappa-B essential modulator (NEMO) (NF-kappa-B essential modifier), (Inhibitor of nuclear factor kappa-B kinase subunit gamma) (IkB kinase, subunit gamma) (I-kappa-B kinase gamma) (IKK-gamma) (IKKG) (IkB, kinase-associated protein 1) (IKKAP1) (FIP-3).]	Cytosolic
O95757	[Heat shock 70 kDa protein 4L (Osmotic stress protein 94) (Heat shock, 70-related protein APG-1).]	Cytosolic
Q04671	[P protein (Melanocyte-specific transporter protein) (Pink-eyed, dilution protein homolog).]	Cytosolic
P51003	[Poly(A) polymerase alpha (EC 2.7.7.19) (PAP) (Polynucleotide, adenylyltransferase alpha).]	Cytosolic
Q15366	Poly(rC)-binding protein 2 (Alpha-CP2) (hnRNP-E2).	Cytosolic
P32119	[Peroxiredoxin-2 (EC 1.11.1.15) (Thioredoxin peroxidase 1), (Thioredoxin-dependent peroxide reductase 1) (Thiol-specific, antioxidant protein) (TSA) (PRP) (Natural killer cell-enhancing factor, B) (NKEF-B).]	Cytosolic
Q9NQ66	[1-phosphatidylinositol-4,5-bisphosphate phosphodiesterase beta 1, (EC 3.1.4.11) (Phosphoinositide phospholipase C) (Phospholipase C-, beta-1) (PLC-beta-1) (PLC-I) (PLC-154).]	Cytosolic
P19174	[1-phosphatidylinositol-4,5-bisphosphate phosphodiesterase gamma 1, (EC 3.1.4.11) (Phosphoinositide phospholipase C) (PLC-gamma-1), (Phospholipase C-gamma-1) (PLC-II) (PLC-148).]	Cytosolic
P29590	[Probable transcription factor PML (Tripartite motif-containing protein, 19) (RING finger protein 71).]	Cytosolic
P35998	[26S protease regulatory subunit 7 (Proteasome 26S subunit ATPase 2), (Protein MSS1).]	Cytosolic
P35236	[Tyrosine-protein phosphatase non-receptor type 7 (EC 3.1.3.48), (Protein-tyrosine phosphatase LC-PTP) (Hematopoietic protein-tyrosine, phosphatase) (HEPTP).]	Cytosolic

**O95825**	[Quinone oxidoreductase-like 1 (EC 1.-.-.-) (QOH-1) (Zeta-crystallin, homolog) (4P11).]	Cytosolic
**P82980**	Retinol-binding protein III, cellular (CRBP-III) (HRBPiso).	Cytosolic
**P62888**	60S ribosomal protein L30.	Cytosolic
**P15880**	40S ribosomal protein S2 (S4) (LLRep3 protein).	Cytosolic
**P20936**	[Ras GTPase-activating protein 1 (GTPase-activating protein) (GAP) (Ras, p21 protein activator) (p120GAP) (RasGAP).]	Cytosolic
**Q15436**	Protein transport protein Sec23A (SEC23-related protein A).	Cytosolic
**P57772**	[Selenocysteine-specific elongation factor (Elongation factor sec), (Eukaryotic elongation factor, selenocysteine-tRNA-specific).]	Cytosolic
**Q9BT40**	[Skeletal muscle and kidney-enriched inositol phosphatase, (EC 3.1.3.56).]	Cytosolic
**O60493**	Sorting nexin-3 (Protein SDP3).	Cytosolic
**P08294**	[Extracellular superoxide dismutase [Cu-Zn] precursor (EC 1.15.1.1), (EC-SOD).]	Cytosolic
**Q9NSD9**	[Phenylalanyl-tRNA synthetase beta chain (EC 6.1.1.20) (Phenylalanine--, tRNA ligase beta chain) (PheRS).]	Cytosolic
**P54136**	[Arginyl-tRNA synthetase, cytoplasmic (EC 6.1.1.19) (Arginine--tRNA, ligase) (ArgRS).]	Cytosolic
**P54577**	[Tyrosyl-tRNA synthetase, cytoplasmic (EC 6.1.1.1) (Tyrosyl--tRNA, ligase) (TyrRS).]	Cytosolic
**Q03519**	[Antigen peptide transporter 2 (APT2) (ATP-binding cassette sub-family, B member 3) (Peptide transporter TAP2) (Peptide transporter PSF2), (Peptide supply factor 2) (PSF-2) (Peptide transporter involved in, antigen processing 2).]	Cytosolic
**P78371**	T-complex protein 1 subunit beta (TCP-1-beta) (CCT-beta).	Cytosolic
**P55072**	[Transitional endoplasmic reticulum ATPase (TER ATPase) (15S Mg(2+)-, ATPase p97 subunit) (Valosin-containing protein) (VCP).]	Cytosolic
**P49815**	Tuberin (Tuberous sclerosis 2 protein).	Cytosolic
**Q13432**	Unc-119 protein homolog (Retinal protein 4) (HRG4).	Cytosolic
**P40337**	Von Hippel-Lindau disease tumor suppressor (pVHL) (Protein G7).	Cytosolic
**O75436**	[Vacuolar protein sorting-associated protein 26A (Vesicle protein, sorting 26A) (hVPS26).]	Cytosolic
**Q9NRL3**	Striatin-4 (Zinedin).	Cytosolic
**Q16537**	[Serine/threonine-protein phosphatase 2A 56 kDa regulatory subunit, epsilon isoform (PP2A, B subunit, B' epsilon isoform) (PP2A, B, subunit, B56 epsilon isoform) (PP2A, B subunit, PR61 epsilon isoform), (PP2A, B subunit, R5 epsilon isoform).]	Cytosolic
**P53582**	[Methionine aminopeptidase 1 (EC 3.4.11.18) (MetAP 1) (MAP 1), (Peptidase M 1).]	Cytosolic
**Q99873**	[Protein arginine N-methyltransferase 1 (EC 2.1.1.-) (Interferon, receptor 1-bound protein 4).]	Cytosolic
**P02649**	Apolipoprotein E precursor (Apo-E).	Cytosolic
**P25098**	[Beta-adrenergic receptor kinase 1 (EC 2.7.11.15) (Beta-ARK-1) (G-, protein coupled receptor kinase 2).]	Cytosolic
**P49407**	Beta-arrestin-1 (Arrestin beta 1).	Cytosolic
**O00192**	Armadillo repeat protein deleted in velo-cardio-facial syndrome.	Cytosolic
**O95817**	[BAG family molecular chaperone regulator 3 (Bcl-2-associated, athanogene 3) (BAG-3) (Bcl-2-binding protein Bis) (Docking protein, CAIR-1).]	Cytosolic
**P19835**	[Bile salt-activated lipase precursor (EC 3.1.1.3) (EC 3.1.1.13) (BAL), (Bile salt-stimulated lipase) (BSSL) (Carboxyl ester lipase) (Sterol, esterase) (Cholesterol esterase) (Pancreatic lysophospholipase).]	Cytosolic
**P50747**	[Biotin--protein ligase (EC 6.3.4.-) (Biotin apo-protein ligase), [Includes: Biotin--[methylmalonyl-CoA-carboxytransferase] ligase, (EC 6.3.4.9); Biotin--[propionyl-CoA-carboxylase [ATP-hydrolyzing]], ligase (EC 6.3.4.10) (Holocarboxylase synthetase) (HCS); Biotin--, [methylcrotonoyl-CoA-carboxylase] ligase (EC 6.3.4.11); Biotin--, [acetyl-CoA-carboxylase] ligase (EC 6.3.4.15)].]	Cytosolic
**P35070**	Probetacellulin precursor [Contains: Betacellulin (BTC)].	Cytosolic

**Q14511**	[Enhancer of filamentation 1 (HEF1) (CRK-associated substrate-related, protein) (CAS-L) (CasL) (p105) (Protein NEDD9) (Renal carcinoma, antigen NY-REN-12) [Contains: Enhancer of filamentation 1 p55].]	Cytosolic
**P49454**	[Centromere protein F (Kinetochore protein CENP-F) (Mitosin) (AH, antigen).]	Cytosolic
**Q16280**	[Cyclic nucleotide-gated olfactory channel (Cyclic nucleotide-gated, cation channel 2) (CNG channel 2) (CNG-2) (CNG2).]	Cytosolic
**P45877**	[Peptidyl-prolyl cis-trans isomerase C (EC 5.2.1.8) (PPIase) (Rotamase), (Cyclophilin C).]	Cytosolic
**P16989**	[DNA-binding protein A (Cold shock domain-containing protein A), (Single-strand DNA-binding protein NF-GMB).]	Cytosolic
**Q9UMR2**	[ATP-dependent RNA helicase DDX19B (EC 3.6.1.-) (DEAD box protein 19B), (DEAD box RNA helicase DEAD5).]	Cytosolic
**Q08211**	[ATP-dependent RNA helicase A (EC 3.6.1.-) (Nuclear DNA helicase II), (NDH II) (DEAH box protein 9).]	Cytosolic
**Q12882**	[Dihydropyrimidine dehydrogenase [NADP+] precursor (EC 1.3.1.2) (DPD), (DHPDHase) (Dihydrouracil dehydrogenase) (Dihydrothymine, dehydrogenase).]	Cytosolic
**P54259**	Atrophin-1 (Dentatorubral-pallidoluysian atrophy protein).	Cytosolic
**Q16828**	[Dual specificity protein phosphatase 6 (EC 3.1.3.48) (EC 3.1.3.16), (Mitogen-activated protein kinase phosphatase 3) (MAP kinase, phosphatase 3) (MKP-3) (Dual specificity protein phosphatase PYST1).]	Cytosolic
**Q14204**	[Dynein heavy chain, cytosolic (DYHC) (Cytoplasmic dynein heavy chain, 1) (DHC1) (Dynein heavy chain 1, cytoplasmic 1).]	Cytosolic
**Q14203**	[Dynactin subunit 1 (150 kDa dynein-associated polypeptide) (DP-150), (DAP-150) (p150-glued) (p135).]	Cytosolic
**Q9HBU6**	Ethanolamine kinase 1 (EC 2.7.1.82) (EKI 1).	Cytosolic
**P62495**	[Eukaryotic peptide chain release factor subunit 1 (eRF1) (Eukaryotic, release factor 1) (TB3-1) (Protein Cl1).]	Cytosolic
**O00519**	[Fatty-acid amide hydrolase 1 (EC 3.1.-.-) (Oleamide hydrolase 1), (Anandamide amidohydrolase 1).]	Cytosolic
**Q00597**	Fanconi anemia group C protein (Protein FACC).	Cytosolic
**Q92636**	[Protein FAN (Factor associated with N-SMase activation) (Factor, associated with neutral sphingomyelinase activation).]	Cytosolic
**Q13630**	[GDP-L-fucose synthetase (EC 1.1.1.271) (Protein FX) (Red cell NADP(H)-, binding protein) (GDP-4-keto-6-deoxy-D-mannose-3,5-epimerase-4-, reductase).]	Cytosolic
**P49771**	[SL cytokine precursor (Fms-related tyrosine kinase 3 ligand) (Flt3, ligand) (Flt3L).]	Cytosolic
**P04066**	[Tissue alpha-L-fucosidase precursor (EC 3.2.1.51) (Alpha-L-fucosidase, I) (Alpha-L-fucoside fucohydrolase).]	Cytosolic
**O95749**	[Geranylgeranyl pyrophosphate synthetase (GGPP synthetase) (GGPPSase), (Geranylgeranyl diphosphate synthase) [Includes:, Dimethylallyltranstransferase (EC 2.5.1.1); Geranyltranstransferase, (EC 2.5.1.10); Farnesyltranstransferase (EC 2.5.1.29)].]	Cytosolic
**P10071**	Zinc finger protein GLI3.	Cytosolic
**P09211**	Glutathione S-transferase P (EC 2.5.1.18) (GST class-pi) (GSTP1-1).	Cytosolic
**Q92598**	[Heat shock protein 105 kDa (Heat shock 110 kDa protein) (Antigen NY-, CO-25).]	Cytosolic
**Q9UQL6**	Histone deacetylase 5 (HD5) (Antigen NY-CO-9).	Cytosolic
**P51610**	[Host cell factor (HCF) (HCF-1) (C1 factor) (VP16 accessory protein), (VCAF) (CFF) [Contains: HCF N-terminal chain 1; HCF N-terminal chain, 2; HCF N-terminal chain 3; HCF N-terminal chain 4; HCF N-terminal, chain 5; HCF N-terminal chain 6; HCF C-terminal chain 1; HCF C-, terminal chain 2; HCF C-terminal chain 3; HCF C-terminal chain 4; HCF, C-terminal chain 5; HCF C-terminal chain 6].]	Cytosolic
**Q9H2X6**	Homeodomain-interacting protein kinase 2 (EC 2.7.11.1) (hHIPk2).	Cytosolic
**P34913**	[Epoxide hydrolase 2 (EC 3.3.2.10) (Soluble epoxide hydrolase) (SEH), (Epoxide hydratase) (Cytosolic epoxide hydrolase) (CEH).]	Cytosolic
**O00221**	[NF-kappa-B inhibitor epsilon (NF-kappa-BIE) (I-kappa-B-epsilon), (IkappaBepsilon) (IKB-epsilon) (IKBE).]	Cytosolic
**P52292**	[Importin subunit alpha-2 (Karyopherin subunit alpha-2) (SRP1-alpha), (RAG cohort protein 1).]	Cytosolic
**P48200**	[Iron-responsive element-binding protein 2 (IRE-BP 2) (Iron regulatory, protein 2) (IRP2).]	Cytosolic

P05783	[Keratin, type I cytoskeletal 18 (Cytokeratin-18) (CK-18) (Keratin-18), (K18) (Cell proliferation-inducing gene 46 protein).]	Cytosolic
O75912	[Diacylglycerol kinase iota (EC 2.7.1.107) (Diglyceride kinase iota), (DGK-iota) (DAG kinase iota).]	Cytosolic
Q92876	[Kallikrein-6 precursor (EC 3.4.21.-) (Protease M) (Neurosin) (Zyme), (SP59) (Serine protease 9) (Serine protease 18).]	Cytosolic
Q15139	[Serine/threonine-protein kinase D1 (EC 2.7.11.13) (nPKC-D1) (Protein, kinase D) (Protein kinase C mu type) (nPKC-mu).]	Cytosolic
P31749	[RAC-alpha serine/threonine-protein kinase (EC 2.7.11.1) (RAC-PK-alpha), (Protein kinase B) (PKB) (C-AKT).]	Cytosolic
Q05469	Hormone-sensitive lipase (EC 3.1.1.79) (HSL).	Cytosolic
P40925	[Malate dehydrogenase, cytoplasmic (EC 1.1.1.37) (Cytosolic malate, dehydrogenase).]	Cytosolic
Q9UBF1	[Melanoma-associated antigen C2 (MAGE-C2 antigen) (MAGE-E1 antigen), (Hepatocellular carcinoma-associated antigen 587) (Cancer-testis, antigen 10) (CT10).]	Cytosolic
P53778	[Mitogen-activated protein kinase 12 (EC 2.7.11.24) (Extracellular, signal-regulated kinase 6) (ERK-6) (ERK5) (Stress-activated protein, kinase 3) (Mitogen-activated protein kinase p38 gamma) (MAP kinase p38, gamma).]	Cytosolic
P58340	Myeloid leukemia factor 1 (Myelodysplasia-myeloid leukemia factor 1).	Cytosolic
P06733	[Alpha-enolase (EC 4.2.1.11) (2-phospho-D-glycerate hydro-lyase) (Non-, neural enolase) (NNE) (Enolase 1) (Phosphopyruvate hydratase) (C-myc, promoter-binding protein) (MBP-1) (MPB-1) (Plasminogen-binding, protein).]	Cytosolic
Q14764	Major vault protein (MVP) (Lung resistance-related protein).	Cytosolic
P16333	[Cytoplasmic protein NCK1 (NCK adaptor protein 1) (SH2/SH3 adaptor, protein NCK-alpha).]	Cytosolic
Q13469	[Nuclear factor of activated T-cells, cytoplasmic 2 (T-cell, transcription factor NFAT1) (NFAT pre-existing subunit) (NF-ATp).]	Cytosolic
Q13287	N-myc-interactor (Nmi) (N-myc and STAT interactor).	Cytosolic
O15130	[FMRFamide-related peptides precursor [Contains: Neuropeptide SF, (NPSF); Neuropeptide FF (NPFF); Neuropeptide AF (NPAF)].]	Cytosolic
P00973	[2'-5'-oligoadenylate synthetase 1 (EC 2.7.7.-) ((2-5')oligo(A), synthetase 1) (2-5A synthetase 1) (p46/p42 OAS) (E18/E16).]	Cytosolic
O15294	[UDP-N-acetylglucosamine--peptide N-acetylglucosaminyltransferase 110, kDa subunit (EC 2.4.1.-) (O-GlcNAc transferase subunit p110) (O-linked, N-acetylglucosamine transferase 110 kDa subunit).]	Cytosolic
P04637	[Cellular tumor antigen p53 (Tumor suppressor p53) (Phosphoprotein p53), (Antigen NY-CO-13).]	Cytosolic
Q99471	Prefoldin subunit 5 (C-myc-binding protein Mm-1) (Myc modulator 1).	Cytosolic
P62195	[26S protease regulatory subunit 8 (Proteasome 26S subunit ATPase 5), (Proteasome subunit p45) (p45/SUG) (Thyroid hormone receptor-, interacting protein 1) (TRIP1).]	Cytosolic
P55786	Puromycin-sensitive aminopeptidase (EC 3.4.11.-) (PSA).	Cytosolic
Q03431	[Parathyroid hormone/parathyroid hormone-related peptide receptor, precursor (PTH/PTHr receptor) (PTH/PTHrP type I receptor).]	Cytosolic
P15151	[Poliovirus receptor precursor (Nectin-like protein 5) (Necl-5) (CD155, antigen).]	Cytosolic
Q07020	60S ribosomal protein L18.	Cytosolic
P05388	60S acidic ribosomal protein P0 (L10E).	Cytosolic
P09651	[Heterogeneous nuclear ribonucleoprotein A1 (Helix-destabilizing, protein) (Single-strand RNA-binding protein) (hnRNP core protein A1).]	Cytosolic
Q9NQT5	[Exosome complex exonuclease RRP40 (EC 3.1.13.-) (Ribosomal RNA-, processing protein 40) (Exosome component 3) (p10).]	Cytosolic
P62841	40S ribosomal protein S15 (RIG protein).	Cytosolic
P23396	40S ribosomal protein S3.	Cytosolic
Q15019	Septin-2 (Protein NEDD5).	Cytosolic
O95219	Sorting nexin-4.	Cytosolic

Q9NRS6	Sorting nexin-15.	Cytosolic
Q9NYA1	Sphingosine kinase 1 (EC 2.7.1.-) (SK 1) (SPK 1).	Cytosolic
Q15528	[Mediator of RNA polymerase II transcription subunit 22 (Mediator, complex subunit 22) (Surfeit locus protein 5).]	Cytosolic
P14868	[Aspartyl-tRNA synthetase, cytoplasmic (EC 6.1.1.12) (Aspartate--tRNA, ligase) (AspRS) (Cell proliferation-inducing gene 40 protein).]	Cytosolic
P41250	Glycyl-tRNA synthetase (EC 6.1.1.14) (Glycine--tRNA ligase) (GlyRS).	Cytosolic
P26640	[Valyl-tRNA synthetase (EC 6.1.1.9) (Valine--tRNA ligase) (ValRS), (Protein G7a).]	Cytosolic
Q12815	Trophinin-associated protein (Tastin) (Trophinin-assisting protein).	Cytosolic
O75347	[Tubulin-specific chaperone A (Tubulin-folding cofactor A) (CFA) (TCP1-, chaperonin cofactor A).]	Cytosolic
P50990	[T-complex protein 1 subunit theta (TCP-1-theta) (CCT-theta) (Renal, carcinoma antigen NY-REN-15).]	Cytosolic
Q9Y6M0	[Testisin precursor (EC 3.4.21.-) (Serine protease 21) (Eosinophil, serine protease 1) (ESP-1).]	Cytosolic
Q04206	Transcription factor p65 (Nuclear factor NF-kappa-B p65 subunit).	Cytosolic
P29144	[Tripeptidyl-peptidase 2 (EC 3.4.14.10) (Tripeptidyl-peptidase II), (TPP-II) (Tripeptidyl aminopeptidase).]	Cytosolic
P26651	[Tristetraproline (TTP) (Zinc finger protein 36 homolog) (Zfp-36), (Protein TIS11A) (TIS11) (Growth factor-inducible nuclear protein, NUP475) (G0/G1 switch regulatory protein 24).]	Cytosolic
P40126	[L-dopachrome tautomerase precursor (EC 5.3.3.12) (DT) (DCT) (L-, dopachrome Delta-isomerase) (Tyrosinase-related protein 2) (TRP-2), (TRP2).]	Cytosolic
P52758	[Ribonuclease UK114 (EC 3.1.-.-) (14.5 kDa translational inhibitor, protein) (p14.5) (UK114 antigen homolog).]	Cytosolic
P30153	[Serine/threonine-protein phosphatase 2A 65 kDa regulatory subunit A, alpha isoform (PP2A, subunit A, PR65-alpha isoform) (PP2A, subunit A,, R1-alpha isoform) (Medium tumor antigen-associated 61 kDa protein).]	Cytosolic
Q9Y6B7	[AP-4 complex subunit beta-1 (Adapter-related protein complex 4 beta 1, subunit) (Beta subunit of AP-4) (AP-4 adapter complex subunit beta).]	Cytosolic
P61163	[Alpha-centractin (Centractin) (Centrosome-associated actin homolog), (Actin-RPV) (ARP1).]	Cytosolic
P00326	[Alcohol dehydrogenase 1C (EC 1.1.1.1) (Alcohol dehydrogenase gamma, subunit).]	Cytosolic
P50579	[Methionine aminopeptidase 2 (EC 3.4.11.18) (MetAP 2) (Peptidase M 2), (Initiation factor 2-associated 67 kDa glycoprotein) (p67) (p67eIF2).]	Cytosolic
P10275	Androgen receptor (Dihydrotestosterone receptor).	Cytosolic
Q15052	[Rho guanine nucleotide exchange factor 6 (Rac/Cdc42 guanine nucleotide, exchange factor 6) (PAK-interacting exchange factor alpha) (Alpha-Pix), (COOL-2).]	Cytosolic
O43681	[Arsenical pump-driving ATPase (EC 3.6.3.16) (Arsenite-translocating, ATPase) (Arsenical resistance ATPase) (Arsenite-transporting ATPase), (ARSA) (ASNA-I).]	Cytosolic
P00918	[Carbonic anhydrase 2 (EC 4.2.1.1) (Carbonic anhydrase II) (Carbonate, dehydratase II) (CA-II) (Carbonic anhydrase C).]	Cytosolic
P35520	[Cystathionine beta-synthase (EC 4.2.1.22) (Serine sulfhydrase) (Beta-, thionase).]	Cytosolic
P27815	[cAMP-specific 3',5'-cyclic phosphodiesterase 4A (EC 3.1.4.17) (DPDE2), (PDE46).]	Cytosolic
P02511	[Alpha-crystallin B chain (Alpha(B)-crystallin) (Rosenthal fiber, component) (Heat shock protein beta-5) (HspB5) (Renal carcinoma, antigen NY-REN-27).]	Cytosolic
P46108	Proto-oncogene C-crk (p38) (Adapter molecule crk).	Cytosolic
P41240	[Tyrosine-protein kinase CSK (EC 2.7.10.2) (C-SRC kinase) (Protein-, tyrosine kinase CYL).]	Cytosolic

**P49585**	[Choline-phosphate cytidylyltransferase A (EC 2.7.7.15), (Phosphorylcholine transferase A) (CTP:phosphocholine, cytidylyltransferase A) (CT A) (CCT A) (CCT-alpha).]	Cytosolic
**P62937**	[Peptidyl-prolyl cis-trans isomerase A (EC 5.2.1.8) (PPIase A), (Rotamase A) (Cyclophilin A) (Cyclosporin A-binding protein).]	Cytosolic
**Q99259**	[Glutamate decarboxylase 1 (EC 4.1.1.15) (Glutamate decarboxylase 67, kDa isoform) (GAD-67) (67 kDa glutamic acid decarboxylase).]	Cytosolic
**Q9UHI6**	[Probable ATP-dependent RNA helicase DDX20 (EC 3.6.1.-) (DEAD box, protein 20) (DEAD box protein DP 103) (Component of gems 3) (Gemin-3).]	Cytosolic
**O00571**	[ATP-dependent RNA helicase DDX3X (EC 3.6.1.-) (DEAD box protein 3, X-, chromosomal) (Helicase-like protein 2) (HLP2) (DEAD box, X isoform).]	Cytosolic
**P06746**	DNA polymerase beta (EC 2.7.7.7) (EC 4.2.99.-).	Cytosolic
**O14640**	[Segment polarity protein dishevelled homolog DVL-1 (Dishevelled-1), (DSH homolog 1).]	Cytosolic
**P06733**	[Alpha-enolase (EC 4.2.1.11) (2-phospho-D-glycerate hydro-lyase) (Non-, neural enolase) (NNE) (Enolase 1) (Phosphopyruvate hydratase) (C-myc, promoter-binding protein) (MBP-1) (MPB-1) (Plasminogen-binding, protein).]	Cytosolic
**Q92990**	[Glomulin (FKBP-associated protein) (FK506-binding protein-associated, protein) (FAP).]	Cytosolic
**P14314**	[Glucosidase 2 subunit beta precursor (Glucosidase II subunit beta), (Protein kinase C substrate, 60.1 kDa protein, heavy chain) (PKCSH), (80K-H protein).]	Cytosolic
**O60547**	[GDP-mannose 4,6 dehydratase (EC 4.2.1.47) (GDP-D-mannose dehydratase), (GMD).]	Cytosolic
**Q12849**	G-rich sequence factor 1 (GRSF-1).	Cytosolic
**Q9Y2T3**	[Guanine deaminase (EC 3.5.4.3) (Guanase) (Guanine aminase) (Guanine, aminohydrolase) (GAH) (p51-nedasin).]	Cytosolic
**Q30201**	Hereditary hemochromatosis protein precursor (HLA-H).	Cytosolic
**O95163**	[Elongator complex protein 1 (ELP1) (IkappaB kinase complex-associated, protein) (IKK complex-associated protein) (p150).]	Cytosolic
**Q13418**	[Integrin-linked protein kinase (EC 2.7.11.1) (ILK-1) (ILK-2) (59 kDa, serine/threonine-protein kinase) (p59ILK).]	Cytosolic
**Q14974**	[Importin subunit beta-1 (Karyopherin subunit beta-1) (Nuclear factor, P97) (Importin 90).]	Cytosolic
**Q15181**	[Inorganic pyrophosphatase (EC 3.6.1.1) (Pyrophosphate phospho-, hydrolase) (PPase).]	Cytosolic
**Q16760**	[Diacylglycerol kinase delta (EC 2.7.1.107) (Diglyceride kinase delta), (DGK-delta) (DAG kinase delta) (130 kDa diacylglycerol kinase).]	Cytosolic
**O95239**	Chromosome-associated kinesin KIF4A (Chromokinesin A).	Cytosolic
**P53667**	LIM domain kinase 1 (EC 2.7.11.1) (LIMK-1).	Cytosolic
**P48163**	NADP-dependent malic enzyme (EC 1.1.1.40) (NADP-ME) (Malic enzyme 1).	Cytosolic
**P14209**	[CD99 antigen precursor (T-cell surface glycoprotein E2) (E2 antigen), (Protein MIC2) (12E7).]	Cytosolic
**Q9NPJ1**	[McKusick-Kaufman/Bardet-Biedl syndromes putative chaperonin (Bardet-, Biedl syndrome 6 protein).]	Cytosolic
**Q13402**	Myosin-VIIa.	Cytosolic
**P14598**	[Neutrophil cytosol factor 1 (NCF-1) (Neutrophil NADPH oxidase factor, 1) (47 kDa neutrophil oxidase factor) (p47-phox) (NCF-47K) (47 kDa, autosomal chronic granulomatous disease protein) (Nox organizer 2), (Nox-organizing protein 2) (SH3 and PX domain-containing protein 1A).]	Cytosolic
**P30990**	[Neurotensin/neuromedin N precursor [Contains: Large neuromedin N (NmN-, 125); Neuromedin N (NmN) (NN); Neurotensin (NT); Tail peptide].]	Cytosolic
**P08949**	Neuromedin-B precursor [Contains: Neuromedin-B-32; Neuromedin-B].	Cytosolic
**P37198**	Nuclear pore glycoprotein p62 (62 kDa nucleoporin).	Cytosolic

**P51810**	G-protein coupled receptor 143 (Ocular albinism type 1 protein).	Cytosolic
**Q9BXW6**	[Oxysterol-binding protein-related protein 1 (OSBP-related protein 1), (ORP-1).]	Cytosolic
**Q12888**	[Tumor suppressor p53-binding protein 1 (p53-binding protein 1), (p53BP1) (53BP1).]	Cytosolic
**Q13153**	[Serine/threonine-protein kinase PAK 1 (EC 2.7.11.1) (p21-activated, kinase 1) (PAK-1) (p65-PAK) (Alpha-PAK).]	Cytosolic
**O15530**	3-phosphoinositide-dependent protein kinase 1 (EC 2.7.11.1) (hPDK1).	Cytosolic
**Q13608**	[Peroxisome assembly factor 2 (PAF-2) (Peroxisomal-type ATPase 1), (Peroxin-6) (Peroxisomal biogenesis factor 6).]	Cytosolic
**P35354**	[Prostaglandin G/H synthase 2 precursor (EC 1.14.99.1) (Cyclooxygenase-, 2) (COX-2) (Prostaglandin-endoperoxide synthase 2) (Prostaglandin H2, synthase 2) (PGH synthase 2) (PGHS-2) (PHS II).]	Cytosolic
**P51178**	[1-phosphatidylinositol-4,5-bisphosphate phosphodiesterase delta 1, (EC 3.1.4.11) (Phosphoinositide phospholipase C) (PLC-delta-1), (Phospholipase C-delta-1) (PLC-III).]	Cytosolic
**O00750**	[Phosphatidylinositol-4-phosphate 3-kinase C2 domain-containing beta, polypeptide (EC 2.7.1.154) (Phosphoinositide 3-Kinase-C2-beta), (PtdIns-3-kinase C2 beta) (PI3K-C2beta) (C2-PI3K).]	Cytosolic
**P13796**	[Plastin-2 (L-plastin) (Lymphocyte cytosolic protein 1) (LCP-1), (LC64P).]	Cytosolic
**Q05209**	[Tyrosine-protein phosphatase non-receptor type 12 (EC 3.1.3.48), (Protein-tyrosine phosphatase G1) (PTPG1) (PTP-PEST).]	Cytosolic
**P53801**	[Pituitary tumor-transforming gene 1 protein-interacting protein, precursor (Pituitary tumor-transforming gene protein-binding factor), (PTTG-binding factor) (PBF).]	Cytosolic
**O15067**	[Phosphoribosylformylglycinamidine synthase (EC 6.3.5.3) (FGAM, synthase) (FGAMS) (Formylglycinamide ribotide amidotransferase), (FGARAT) (Formylglycinamide ribotide synthetase).]	Cytosolic
**P61106**	Ras-related protein Rab-14.	Cytosolic
**Q02543**	60S ribosomal protein L18a.	Cytosolic
**P62269**	40S ribosomal protein S18 (Ke-3) (Ke3).	Cytosolic
**P62241**	40S ribosomal protein S8.	Cytosolic
**P23526**	[Adenosylhomocysteinase (EC 3.3.1.1) (S-adenosyl-L-homocysteine, hydrolase) (AdoHcyase).]	Cytosolic
**P48594**	Serpin B4 (Squamous cell carcinoma antigen 2) (SCCA-2) (Leupin).	Cytosolic
**P55822**	[SH3 domain-binding glutamic acid-rich protein (SH3BGR protein) (21-, glutamic acid-rich protein) (21-GARP).]	Cytosolic
**O14893**	[Survival of motor neuron protein-interacting protein 1 (SMN-, interacting protein 1) (Component of gems 2) (Gemin-2).]	Cytosolic
**P57059**	[Serine/threonine-protein kinase SNF1-like kinase 1 (EC 2.7.11.1), (Serine/threonine-protein kinase SNF1LK) (SIK1).]	Cytosolic
**O60749**	Sorting nexin-2 (Transformation-related gene 9 protein) (TRG-9).	Cytosolic
**P42224**	[Signal transducer and activator of transcription 1-alpha/beta, (Transcription factor ISGF-3 components p91/p84).]	Cytosolic
**P07814**	[Bifunctional aminoacyl-tRNA synthetase [Includes: Glutamyl-tRNA, synthetase (EC 6.1.1.17) (Glutamate--tRNA ligase); Prolyl-tRNA, synthetase (EC 6.1.1.15) (Proline--tRNA ligase)].]	Cytosolic
**P41252**	[Isoleucyl-tRNA synthetase, cytoplasmic (EC 6.1.1.5) (Isoleucine--tRNA, ligase) (IleRS) (IRS).]	Cytosolic
**P26639**	[Threonyl-tRNA synthetase, cytoplasmic (EC 6.1.1.3) (Threonine--tRNA, ligase) (ThrRS).]	Cytosolic
**P23381**	[Tryptophanyl-tRNA synthetase, cytoplasmic (EC 6.1.1.2) (Tryptophan--, tRNA ligase) (TrpRS) (Interferon-induced protein 53) (IFP53) (hWRS).]	Cytosolic
**Q99593**	T-box transcription factor TBX5 (T-box protein 5).	Cytosolic
**P17987**	T-complex protein 1 subunit alpha (TCP-1-alpha) (CCT-alpha).	Cytosolic
**P22105**	Tenascin-X precursor (TN-X) (Hexabrachion-like protein).	Cytosolic
**P12270**	Nucleoprotein TPR.	Cytosolic

**P08670**	Vimentin.	Cytosolic
**Q99871**	[UCHL5-interacting protein (26S proteasome-associated UCH37-interacting, protein 1) (X-linked protein STS1769).]	Cytosolic
**Q14684**	RRP1-like protein B.	Cytosolic
**Q9UBR2**	Cathepsin Z precursor (EC 3.4.22.-) (Cathepsin X) (Cathepsin P).	ER
**P20813**	Cytochrome P450 2B6 (EC 1.14.14.1) (CYPIIB6) (P450 IIB1).	ER
**P49184**	[Deoxyribonuclease I-like 1 precursor (EC 3.1.21.-) (DNase I-like 1), (Muscle-specific DNase I-like) (DNase X) (XIB).]	ER
**P33908**	[Mannosyl-oligosaccharide 1,2-alpha-mannosidase IA (EC 3.2.1.113), (Processing alpha-1,2-mannosidase IA) (Alpha-1,2-mannosidase IA), (Mannosidase alpha class 1A member 1) (Man(9)-alpha-mannosidase), (Man9-mannosidase).]	ER
**P16435**	NADPH--cytochrome P450 reductase (EC 1.6.2.4) (CPR) (P450R).	ER
**Q13087**	Protein disulfide-isomerase A2 precursor (EC 5.3.4.1) (PDIp).	ER
**P22061**	[Protein-L-isoaspartate(D-aspartate) O-methyltransferase (EC 2.1.1.77), (Protein-beta-aspartate methyltransferase) (PIMT) (Protein L-, isoaspartyl/D-aspartyl methyltransferase) (L-isoaspartyl protein, carboxyl methyltransferase).]	ER
**P04156**	[Major prion protein precursor (PrP) (PrP27-30) (PrP33-35C) (ASCR), (CD230 antigen).]	ER
**P10619**	[Lysosomal protective protein precursor (EC 3.4.16.5) (Cathepsin A), (Carboxypeptidase C) (Protective protein for beta-galactosidase), [Contains: Lysosomal protective protein 32 kDa chain; Lysosomal, protective protein 20 kDa chain].]	ER
**P04275**	[von Willebrand factor precursor (vWF) [Contains: von Willebrand, antigen 2 (von Willebrand antigen II)].]	ER
**O43264**	Centromere/kinetochore protein zw10 homolog.	ER
**Q99541**	Adipophilin (Adipose differentiation-related protein) (ADRP).	ER
**P04114**	[Apolipoprotein B-100 precursor (Apo B-100) [Contains: Apolipoprotein, B-48 (Apo B-48)].]	ER
**P02654**	Apolipoprotein C-I precursor (Apo-CI) (ApoC-I).	ER
**Q9Y2C3**	[Beta-1,3-galactosyltransferase 5 (EC 2.4.1.-) (Beta-1,3-GalTase 5), (Beta3Gal-T5) (b3Gal-T5) (UDP-galactose:beta-N-acetylglucosamine beta-, 1,3-galactosyltransferase 5) (UDP-Gal:beta-GlcNAc beta-1,3-, galactosyltransferase 5) (Beta-3-Gx-T5).]	ER
**O43852**	Calumenin precursor (Crocalbin) (IEF SSP 9302).	ER
**Q9Y6A2**	[Cytochrome P450 46A1 (EC 1.14.13.98) (Cholesterol 24-hydroxylase), (CH24H).]	ER
**Q15392**	[24-dehydrocholesterol reductase precursor (EC 1.3.1.-) (3-beta-, hydroxysterol delta-24-reductase) (Seladin-1) (Diminuto/dwarf1, homolog).]	ER
**P49257**	[Protein ERGIC-53 precursor (ER-Golgi intermediate compartment 53 kDa, protein) (Lectin mannose-binding 1) (Gp58) (Intracellular mannose-, specific lectin MR60).]	ER
**Q13724**	[Mannosyl-oligosaccharide glucosidase (EC 3.2.1.106) (Processing A-, glucosidase I).]	ER
**O60725**	[Protein-S-isoprenylcysteine O-methyltransferase (EC 2.1.1.100), (Isoprenylcysteine carboxylmethyltransferase) (Prenylcysteine carboxyl, methyltransferase) (pcCMT) (Prenylated protein carboxyl, methyltransferase) (PPMT).]	ER
**Q14643**	[Inositol 1,4,5-trisphosphate receptor type 1 (Type 1 inositol 1,4,5-, trisphosphate receptor) (Type 1 InsP3 receptor) (IP3 receptor isoform, 1) (InsP3R1) (IP3R).]	ER
**Q99828**	[Calcium and integrin-binding protein 1 (Calmyrin) (DNA-PKcs-, interacting protein) (Kinase-interacting protein) (KIP) (CIB) (SNK-, interacting protein 2-28) (SIP2-28).]	ER
**O15118**	Niemann-Pick C1 protein precursor.	ER
**P30101**	[Protein disulfide-isomerase A3 precursor (EC 5.3.4.1) (Disulfide, isomerase ER-60) (ERp60) (58 kDa microsomal protein) (p58) (ERp57) (58, kDa glucose-regulated protein).]	ER
**Q16799**	Reticulon-1 (Neuroendocrine-specific protein).	ER

**Q12770**	[Sterol regulatory element-binding protein cleavage-activating protein, (SREBP cleavage-activating protein) (SCAP).]	ER
**P43307**	[Translocon-associated protein subunit alpha precursor (TRAP-alpha), (Signal sequence receptor subunit alpha) (SSR-alpha).]	ER
**P08842**	[Steryl-sulfatase precursor (EC 3.1.6.2) (Steroid sulfatase) (Steryl-, sulfate sulfohydrolase) (Arylsulfatase C) (ASC).]	ER
**O14975**	[Very long-chain acyl-CoA synthetase (EC 6.2.1.-) (VLCS) (Very long-, chain-fatty-acid-CoA ligase) (VLACS) (THCA-CoA ligase) (Fatty-acid-, coenzyme A ligase, very long-chain 1) (Long-chain-fatty-acid--CoA, ligase) (EC 6.2.1.3) (Fatty acid transport protein 2) (FATP-2) (Solute, carrier family 27 member 2).]	ER
**P27695**	[DNA-(apurinic or apyrimidinic site) lyase (EC 4.2.99.18) (AP, endonuclease 1) (APEX nuclease) (APEN) (Protein REF-1).]	ER
**P16615**	[Sarcoplasmic/endoplasmic reticulum calcium ATPase 2 (EC 3.6.3.8), (Calcium pump 2) (SERCA2) (SR Ca(2+)-ATPase 2) (Calcium-transporting, ATPase sarcoplasmic reticulum type, slow twitch skeletal muscle, isoform) (Endoplasmic reticulum class 1/2 Ca(2+) ATPase).]	ER
**P27824**	[Calnexin precursor (Major histocompatibility complex class I antigen-, binding protein p88) (p90) (IP90).]	ER
**O75503**	Ceroid-lipofuscinosis neuronal protein 5 (Protein CLN5).	ER
**P05093**	[Cytochrome P450 17A1 (EC 1.14.99.9) (CYPXVII) (P450-C17) (P450c17), (Steroid 17-alpha-monooxygenase) (Steroid 17-alpha-hydroxylase/17,20, lyase).]	ER
**Q14571**	[Inositol 1,4,5-trisphosphate receptor type 2 (Type 2 inositol 1,4,5-, trisphosphate receptor) (Type 2 InsP3 receptor) (IP3 receptor isoform, 2) (InsP3R2).]	ER
**O43896**	Kinesin-like protein KIF1C.	ER
**Q92581**	[Sodium/hydrogen exchanger 6 (Na(+)/H(+) exchanger 6) (NHE-6) (Solute, carrier family 9 member 6).]	ER
**O60238**	[BCL2/adenovirus E1B 19 kDa protein-interacting protein 3-like (NIP3L), (NIP3-like protein X) (BCL2/adenovirus E1B 19 kDa protein-interacting, protein 3A) (Adenovirus E1B19K-binding protein B5).]	ER
**Q9Y4L1**	[Hypoxia up-regulated protein 1 precursor (150 kDa oxygen-regulated, protein) (Orp150).]	ER
**P13667**	[Protein disulfide-isomerase A4 precursor (EC 5.3.4.1) (Protein ERp-72), (ERp72).]	ER
**O00469**	[Procollagen-lysine,2-oxoglutarate 5-dioxygenase 2 precursor, (EC 1.14.11.4) (Lysyl hydroxylase 2) (LH2).]	ER
**Q9NQC3**	[Reticulon-4 (Neurite outgrowth inhibitor) (Nogo protein) (Foocen), (Neuroendocrine-specific protein) (NSP) (Neuroendocrine-specific, protein C homolog) (RTN-x) (Reticulon-5).]	ER
**Q01105**	[Protein SET (Phosphatase 2A inhibitor I2PP2A) (I-2PP2A) (Template-, activating factor I) (TAF-I) (HLA-DR-associated protein II) (PHAPII), (Inhibitor of granzyme A-activated DNase) (IGAAD).]	ER
**Q9UHB9**	Signal recognition particle 68 kDa protein (SRP68).	ER
**P57727**	[Transmembrane protease, serine 3 (EC 3.4.21.-) (Serine protease TADG-, 12) (Tumor-associated differentially-expressed gene 12 protein).]	ER
**P36222**	[Chitinase-3-like protein 1 precursor (Cartilage glycoprotein 39) (GP-, 39) (39 kDa synovial protein) (HCgp-39) (YKL-40).]	Extracellular
**P01133**	[Pro-epidermal growth factor precursor (EGF) [Contains: Epidermal, growth factor (Urogastrone)].]	Extracellular
**P36222**	[Chitinase-3-like protein 1 precursor (Cartilage glycoprotein 39) (GP-, 39) (39 kDa synovial protein) (HCgp-39) (YKL-40).]	Extracellular
**P19652**	Alpha-1-acid glycoprotein 2 precursor (AGP 2) (Orosomucoid-2) (OMD 2).	Extracellular
**P04217**	Alpha-1B-glycoprotein precursor (Alpha-1-B glycoprotein).	Extracellular
**P08697**	[Alpha-2-antiplasmin precursor (Alpha-2-plasmin inhibitor) (Alpha-2-PI), (Alpha-2-AP).]	Extracellular
**P02765**	[Alpha-2-HS-glycoprotein precursor (Fetuin-A) (Alpha-2-Z-globulin) (Ba-, alpha-2-glycoprotein) [Contains: Alpha-2-HS-glycoprotein chain A;, Alpha-2-HS-glycoprotein chain B].]	Extracellular
**P35858**	[Insulin-like growth factor-binding protein complex acid labile chain, precursor (ALS).]	Extracellular

**P02760**	[AMBP protein precursor [Contains: Alpha-1-microglobulin (Protein HC), (Complex-forming glycoprotein heterogeneous in charge) (Alpha-1, microglycoprotein); Inter-alpha-trypsin inhibitor light chain (ITI-LC), (Bikunin) (HI-30)].]	Extracellular
**P16860**	[Natriuretic peptides B precursor [Contains: Gamma-brain natriuretic, peptide; Brain natriuretic peptide 32 (BNP-32)].]	Extracellular
**P06727**	Apolipoprotein A-IV precursor (Apo-AIV) (ApoA-IV).	Extracellular
**P02656**	Apolipoprotein C-III precursor (Apo-CIII) (ApoC-III).	Extracellular
**O75882**	Attractin precursor (Mahogany homolog) (DPPT-L).	Extracellular
**Q15582**	[Transforming growth factor-beta-induced protein ig-h3 precursor (Beta, ig-h3) (Kerato-epithelin) (RGD-containing collagen-associated protein), (RGD-CAP).]	Extracellular
**P04003**	[C4b-binding protein alpha chain precursor (C4bp) (Proline-rich, protein) (PRP).]	Extracellular
**P07711**	[Cathepsin L precursor (EC 3.4.22.15) (Major excreted protein) (MEP), [Contains: Cathepsin L heavy chain; Cathepsin L light chain].]	Extracellular
**P00450**	Ceruloplasmin precursor (EC 1.16.3.1) (Ferroxidase).	Extracellular
**P08603**	Complement factor H precursor (H factor 1).	Extracellular
**Q9UBP4**	Dickkopf-related protein 3 precursor (Dkk-3) (Dickkopf-3) (hDkk-3).	Extracellular
**Q16610**	Extracellular matrix protein 1 precursor (Secretory component p85).	Extracellular
**P13727**	[Bone-marrow proteoglycan precursor (BMPG) (Proteoglycan 2) [Contains:, Eosinophil granule major basic protein (EMBP) (MBP) (Pregnancy-, associated major basic protein)].]	Extracellular
**P00488**	[Coagulation factor XIII A chain precursor (EC 2.3.2.13) (Coagulation, factor XIIIa) (Protein-glutamine gamma-glutamyltransferase A chain), (Transglutaminase A chain).]	Extracellular
**P00748**	[Coagulation factor XII precursor (EC 3.4.21.38) (Hageman factor) (HAF), [Contains: Coagulation factor XIIa heavy chain; Beta-factor XIIa part, 1; Beta-factor XIIa part 2; Coagulation factor XIIa light chain].]	Extracellular
**P00451**	[Coagulation factor VIII precursor (Procoagulant component), (Antihemophilic factor) (AHF) [Contains: Factor VIIIa heavy chain, 200, kDa isoform; Factor VIIIa heavy chain, 92 kDa isoform; Factor VIII B, chain; Factor VIIIa light chain].]	Extracellular
**Q12805**	[EGF-containing fibulin-like extracellular matrix protein 1 precursor, (Fibulin-3) (FIBL-3) (Fibrillin-like protein) (Extracellular protein, S1-5).]	Extracellular
**P35556**	Fibrillin-2 precursor.	Extracellular
**Q9GZV9**	[Fibroblast growth factor 23 precursor (FGF-23) (Tumor-derived, hypophosphatemia-inducing factor).]	Extracellular
**P13284**	[Gamma-interferon-inducible lysosomal thiol reductase precursor (Gamma-, interferon-inducible protein IP-30).]	Extracellular
**P05546**	[Heparin cofactor 2 precursor (Heparin cofactor II) (HC-II) (Protease, inhibitor leuserpin 2) (HLS2).]	Extracellular
**P06326**	Ig heavy chain V-I region Mot.	Extracellular
**P01772**	Ig heavy chain V-III region KOL.	Extracellular
**P01780**	Ig heavy chain V-III region JON.	Extracellular
**P05155**	[Plasma protease C1 inhibitor precursor (C1 Inh) (C1Inh) (C1 esterase, inhibitor) (C1-inhibiting factor).]	Extracellular
**P09529**	Inhibin beta B chain precursor (Activin beta-B chain).	Extracellular
**P19823**	[Inter-alpha-trypsin inhibitor heavy chain H2 precursor (ITI heavy, chain H2) (Inter-alpha-inhibitor heavy chain 2) (Inter-alpha-trypsin, inhibitor complex component II) (Serum-derived hyaluronan-associated, protein) (SHAP).]	Extracellular
**P23352**	[Anosmin-1 precursor (Kallmann syndrome protein) (Adhesion molecule-, like X-linked).]	Extracellular
**Q9UKQ9**	[Kallikrein-9 precursor (EC 3.4.21.-) (Kallikrein-like protein 3) (KLK-, L3).]	Extracellular
**P01607**	Ig kappa chain V-I region Rei.	Extracellular
**P04431**	Ig kappa chain V-I region Walker precursor.	Extracellular

P06310	Ig kappa chain V-II region RPMI 6410 precursor.	Extracellular
P18136	Ig kappa chain V-III region HIC precursor.	Extracellular
P04180	[Phosphatidylcholine-sterol acyltransferase precursor (EC 2.3.1.43), (Lecithin-cholesterol acyltransferase) (Phospholipid-cholesterol, acyltransferase).]	Extracellular
P06888	Ig lambda chain V-I region EPS.	Extracellular
P80748	Ig lambda chain V-III region LOI.	Extracellular
Q16820	[Meprin A subunit beta precursor (EC 3.4.24.18) (Endopeptidase-2) (N-, benzoyl-L-tyrosyl-P-amino-benzoic acid hydrolase subunit beta) (PABA, peptide hydrolase) (PPH beta).]	Extracellular
P08253	[72 kDa type IV collagenase precursor (EC 3.4.24.24) (72 kDa, gelatinase) (Matrix metalloproteinase-2) (MMP-2) (Gelatinase A) (TBE-, 1).]	Extracellular
P09238	[Stromelysin-2 precursor (EC 3.4.24.22) (Matrix metalloproteinase-10), (MMP-10) (Transin-2) (SL-2).]	Extracellular
O95460	Matrilin-4 precursor.	Extracellular
Q02505	Mucin-3A precursor (Intestinal mucin-3A).	Extracellular
P36955	[Pigment epithelium-derived factor precursor (PEDF) (Serpin-F1) (EPC-, 1).]	Extracellular
P41222	[Prostaglandin-H2 D-isomerase precursor (EC 5.3.99.2) (Lipocalin-type, prostaglandin-D synthase) (Glutathione-independent PGD synthetase), (Prostaglandin-D2 synthase) (PGD2 synthase) (PGDS2) (PGDS) (Beta-trace, protein) (Cerebrin-28).]	Extracellular
P55058	Phospholipid transfer protein precursor (Lipid transfer protein II).	Extracellular
P35542	[Serum amyloid A-4 protein precursor (Constitutively expressed serum, amyloid A protein) (C-SAA).]	Extracellular
P02743	[Serum amyloid P-component precursor (SAP) (9.5S alpha-1-glycoprotein), [Contains: Serum amyloid P-component(1-203)].]	Extracellular
P13521	[Secretogranin-2 precursor (Secretogranin II) (SgII) (Chromogranin-C), [Contains: Secretoneurin (SN)].]	Extracellular
Q13275	Semaphorin-3F precursor (Semaphorin IV) (Sema IV) (Sema III/F).	Extracellular
O00626	[Small-inducible cytokine A22 precursor (CCL22) (Macrophage-derived, chemokine) (MDC(1-69)) (Stimulated T-cell chemotactic protein 1) (CC, chemokine STCP-1) [Contains: MDC(3-69); MDC(5-69); MDC(7-69)].]	Extracellular
P13693	[Translationally-controlled tumor protein (TCTP) (p23) (Histamine-, releasing factor) (HRF) (Fortilin).]	Extracellular
P00734	[Prothrombin precursor (EC 3.4.21.5) (Coagulation factor II) [Contains:, Activation peptide fragment 1; Activation peptide fragment 2; Thrombin, light chain; Thrombin heavy chain].]	Extracellular
P01033	[Metalloproteinase inhibitor 1 precursor (TIMP-1) (Erythroid-, potentiating activity) (EPA) (Tissue inhibitor of metalloproteinases), (Fibroblast collagenase inhibitor) (Collagenase inhibitor).]	Extracellular
O14788	[Tumor necrosis factor ligand superfamily member 11 (Receptor activator, of nuclear factor kappa B ligand) (RANKL) (TNF-related activation-, induced cytokine) (TRANCE) (Osteoprotegerin ligand) (OPGL) (Osteoclast, differentiation factor) (ODF) (CD254 antigen) [Contains: Tumor, necrosis factor ligand superfamily member 11, membrane form; Tumor, necrosis factor ligand superfamily member 11, soluble form].]	Extracellular
P07996	Thrombospondin-1 precursor.	Extracellular
P02766	Transthyretin precursor (Prealbumin) (TBPA) (TTR) (ATTR).	Extracellular
P17948	[Vascular endothelial growth factor receptor 1 precursor (EC 2.7.10.1), (VEGFR-1) (Vascular permeability factor receptor) (Tyrosine-protein, kinase receptor FLT) (Flt-1) (Tyrosine-protein kinase FRT) (Fms-like, tyrosine kinase 1).]	Extracellular
P01009	[Alpha-1-antitrypsin precursor (Alpha-1 protease inhibitor) (Alpha-1-, antiproteinase).]	Extracellular
P01019	[Angiotensinogen precursor (Serpin A8) [Contains: Angiotensin-1, (Angiotensin I) (Ang I); Angiotensin-2 (Angiotensin II) (Ang II);, Angiotensin-3 (Angiotensin III) (Ang III) (Des-Asp[1]-angiotensin, II)].]	Extracellular
P02647	[Apolipoprotein A-I precursor (Apo-AI) (ApoA-I) [Contains:, Apolipoprotein A-I(1-242)].]	Extracellular

**P02749**	[Beta-2-glycoprotein 1 precursor (Beta-2-glycoprotein I), (Apolipoprotein H) (Apo-H) (B2GPI) (Beta(2)GPI) (Activated protein C-, binding protein) (APC inhibitor) (Anticardiolipin cofactor).]	Extracellular
**O15072**	[ADAMTS-3 precursor (EC 3.4.24.-) (A disintegrin and metalloproteinase, with thrombospondin motifs 3) (ADAM-TS 3) (ADAM-TS3) (Procollagen II, amino propeptide-processing enzyme) (Procollagen II N-proteinase) (PC, II-NP).]	Extracellular
**P36222**	[Chitinase-3-like protein 1 precursor (Cartilage glycoprotein 39) (GP-, 39) (39 kDa synovial protein) (HCgp-39) (YKL-40).]	Extracellular
**P07339**	[Cathepsin D precursor (EC 3.4.23.5) [Contains: Cathepsin D light, chain; Cathepsin D heavy chain].]	Extracellular
**P22792**	[Carboxypeptidase N subunit 2 precursor (Carboxypeptidase N polypeptide, 2) (Carboxypeptidase N 83 kDa chain) (Carboxypeptidase N regulatory, subunit) (Carboxypeptidase N large subunit).]	Extracellular
**P15169**	[Carboxypeptidase N catalytic chain precursor (EC 3.4.17.3) (CPN), (Carboxypeptidase N polypeptide 1) (Carboxypeptidase N small subunit), (Lysine carboxypeptidase) (Arginine carboxypeptidase) (Kininase-1), (Serum carboxypeptidase N) (SCPN) (Anaphylatoxin inactivator) (Plasma, carboxypeptidase B).]	Extracellular
**P06276**	[Cholinesterase precursor (EC 3.1.1.8) (Acylcholine acylhydrolase), (Choline esterase II) (Butyrylcholine esterase), (Pseudocholinesterase).]	Extracellular
**P10909**	[Clusterin precursor (Complement-associated protein SP-40,40), (Complement cytolysis inhibitor) (CLI) (NA1/NA2) (Apolipoprotein J), (Apo-J) (Testosterone-repressed prostate message 2) (TRPM-2), [Contains: Clusterin beta chain (ApoJalpha) (Complement cytolysis, inhibitor a chain); Clusterin alpha chain (ApoJbeta) (Complement, cytolysis inhibitor b chain)].]	Extracellular
**P01031**	[Complement C5 precursor [Contains: Complement C5 beta chain;, Complement C5 alpha chain; C5a anaphylatoxin; Complement C5 alpha', chain].]	Extracellular
**P07357**	[Complement component C8 alpha chain precursor (Complement component 8, subunit alpha).]	Extracellular
**P15502**	Elastin precursor (Tropoelastin).	Extracellular
**P05305**	[Endothelin-1 precursor (Preproendothelin-1) (PPET1) [Contains:, Endothelin-1 (ET-1); Big endothelin-1].]	Extracellular
**P05160**	[Coagulation factor XIII B chain precursor (Protein-glutamine gamma-, glutamyltransferase B chain) (Transglutaminase B chain) (Fibrin-, stabilizing factor B subunit).]	Extracellular
**P23142**	Fibulin-1 precursor.	Extracellular
**Q9UBX5**	[Fibulin-5 precursor (FIBL-5) (Developmental arteries and neural crest, EGF-like protein) (Dance) (Urine p50 protein) (UP50).]	Extracellular
**P08620**	[Fibroblast growth factor 4 precursor (FGF-4) (Heparin secretory-, transforming protein) (HST-1) (HST) (Transforming protein KS3) (HBGF-, 4).]	Extracellular
**Q02985**	[Complement factor H-related protein 3 precursor (FHR-3) (H factor-like, protein 3) (DOWN16).]	Extracellular
**Q06136**	[3-ketodihydrosphingosine reductase precursor (EC 1.1.1.102) (3-, dehydrosphinganine reductase) (KDS reductase) (Follicular variant, translocation protein 1) (FVT-1).]	Extracellular
**P01824**	Ig heavy chain V-II region WAH.	Extracellular
**P01777**	Ig heavy chain V-III region TEI.	Extracellular
**P01782**	Ig heavy chain V-III region DOB.	Extracellular
**Q06033**	[Inter-alpha-trypsin inhibitor heavy chain H3 precursor (ITI heavy, chain H3) (Inter-alpha-inhibitor heavy chain 3) (Serum-derived, hyaluronan-associated protein) (SHAP).]	Extracellular
**O43240**	[Kallikrein-10 precursor (EC 3.4.21.-) (Protease serine-like 1) (Normal, epithelial cell-specific 1).]	Extracellular
**P01042**	[Kininogen-1 precursor (Alpha-2-thiol proteinase inhibitor) [Contains:, Kininogen-1 heavy chain; Bradykinin (Kallidin I); Lysyl-bradykinin, (Kallidin II); Kininogen-1 light chain; Low molecular weight growth-, promoting factor].]	Extracellular
**P01594**	Ig kappa chain V-I region AU.	Extracellular
**P01612**	Ig kappa chain V-I region Mev.	Extracellular

**P80362**	Ig kappa chain V-I region WAT.	Extracellular
**P04207**	Ig kappa chain V-III region CLL precursor (Rheumatoid factor).	Extracellular
**P01625**	Ig kappa chain V-IV region Len.	Extracellular
**P54317**	Pancreatic lipase-related protein 2 precursor (EC 3.1.1.3).	Extracellular
**Q9Y4K0**	[Lysyl oxidase homolog 2 precursor (EC 1.4.3.-) (Lysyl oxidase-like, protein 2) (Lysyl oxidase-related protein 2) (Lysyl oxidase-related, protein WS9-14).]	Extracellular
**P01705**	Ig lambda chain V-II region NEI.	Extracellular
**P06318**	Ig lambda chain V-VI region WLT.	Extracellular
**P19875**	[Macrophage inflammatory protein 2-alpha precursor (MIP2-alpha) (CXCL2), (Growth-regulated protein beta) (Gro-beta) [Contains: GRO-beta(5-73), (GRO-beta-T) (SB-251353) (Hematopoietic synergistic factor) (HSF)].]	Extracellular
**P08254**	[Stromelysin-1 precursor (EC 3.4.24.17) (Matrix metalloproteinase-3), (MMP-3) (Transin-1) (SL-1).]	Extracellular
**Q99435**	[Protein kinase C-binding protein NELL2 precursor (NEL-like protein 2), (Nel-related protein 2).]	Extracellular
**P10451**	[Osteopontin precursor (Bone sialoprotein 1) (Secreted phosphoprotein, 1) (SPP-1) (Urinary stone protein) (Nephropontin) (Uropontin).]	Extracellular
**P13611**	[Versican core protein precursor (Large fibroblast proteoglycan), (Chondroitin sulfate proteoglycan core protein 2) (PG-M) (Glial, hyaluronate-binding protein) (GHAP).]	Extracellular
**P02776**	Platelet factor 4 precursor (PF-4) (CXCL4) (Oncostatin A) (Iroplact).	Extracellular
**P00747**	[Plasminogen precursor (EC 3.4.21.7) [Contains: Plasmin heavy chain A;, Activation peptide; Angiostatin; Plasmin heavy chain A, short form;, Plasmin light chain B].]	Extracellular
**P27169**	[Serum paraoxonase/arylesterase 1 (EC 3.1.1.2) (EC 3.1.8.1) (PON 1), (Serum aryldialkylphosphatase 1) (A-esterase 1) (Aromatic esterase 1), (K-45).]	Extracellular
**P07225**	Vitamin K-dependent protein S precursor.	Extracellular
**P02753**	[Plasma retinol-binding protein precursor (PRBP) (RBP) [Contains:, Plasma retinol-binding protein(1-182); Plasma retinol-binding, protein(1-181); Plasma retinol-binding protein(1-179); Plasma retinol-, binding protein(1-176)].]	Extracellular
**P07602**	[Proactivator polypeptide precursor [Contains: Saposin-A (Protein A);, Saposin-B-Val; Saposin-B (Sphingolipid activator protein 1) (SAP-1), (Cerebroside sulfate activator) (CSAct) (Dispersin) (Sulfatide/GM1, activator); Saposin-C (Co-beta-glucosidase) (A1 activator), (Glucosylceramidase activator) (Sphingolipid activator protein 2), (SAP-2); Saposin-D (Protein C) (Component C)].]	Extracellular
**P49908**	Selenoprotein P precursor (SeP).	Extracellular
**P09486**	[SPARC precursor (Secreted protein acidic and rich in cysteine), (Osteonectin) (ON) (Basement-membrane protein 40) (BM-40).]	Extracellular
**O43278**	[Kunitz-type protease inhibitor 1 precursor (Hepatocyte growth factor, activator inhibitor type 1) (HAI-1).]	Extracellular
**P01266**	Thyroglobulin precursor.	Extracellular
**P35443**	Thrombospondin-4 precursor.	Extracellular
**P02774**	[Vitamin D-binding protein precursor (DBP) (Group-specific component), (Gc-globulin) (VDB).]	Extracellular
**Q93097**	Protein Wnt-2b precursor (Wnt-13).	Extracellular
**P25311**	[Zinc-alpha-2-glycoprotein precursor (Zn-alpha-2-glycoprotein) (Zn-, alpha-2-GP).]	Extracellular
**P02763**	Alpha-1-acid glycoprotein 1 precursor (AGP 1) (Orosomucoid-1) (OMD 1).	Extracellular
**P02750**	Leucine-rich alpha-2-glycoprotein precursor (LRG).	Extracellular
**P01023**	Alpha-2-macroglobulin precursor (Alpha-2-M).	Extracellular

P01011	[Alpha-1-antichymotrypsin precursor (ACT) (Cell growth-inhibiting gene, 24/25 protein) [Contains: Alpha-1-antichymotrypsin His-Pro-less].]	Extracellular
P43652	Afamin precursor (Alpha-albumin) (Alpha-Alb).	Extracellular
P02768	Serum albumin precursor.	Extracellular
P01008	Antithrombin-III precursor (ATIII).	Extracellular
P02652	[Apolipoprotein A-II precursor (Apo-AII) (ApoA-II) [Contains:, Apolipoprotein A-II(1-76)].]	Extracellular
P02655	Apolipoprotein C-II precursor (Apo-CII) (ApoC-II).	Extracellular
P00736	[Complement C1r subcomponent precursor (EC 3.4.21.41) (Complement, component 1, r subcomponent) [Contains: Complement C1r subcomponent, heavy chain; Complement C1r subcomponent light chain].]	Extracellular
Q15782	[Chitinase-3-like protein 2 precursor (YKL-39) (Chondrocyte protein, 39).]	Extracellular
P02462	Collagen alpha-1(IV) chain precursor (Arresten).	Extracellular
P12110	Collagen alpha-2(VI) chain precursor.	Extracellular
P06681	[Complement C2 precursor (EC 3.4.21.43) (C3/C5 convertase) [Contains:, Complement C2b fragment; Complement C2a fragment].]	Extracellular
P49747	Cartilage oligomeric matrix protein precursor (COMP).	Extracellular
P54108	Cysteine-rich secretory protein 3 precursor (CRISP-3) (SGP28 protein).	Extracellular
O00548	[Delta-like protein 1 precursor (Drosophila Delta homolog 1) (Delta1), (H-Delta-1).]	Extracellular
P08218	Elastase-2B precursor (EC 3.4.21.71).	Extracellular
P12259	[Coagulation factor V precursor (Activated protein C cofactor), [Contains: Coagulation factor V heavy chain; Coagulation factor V, light chain].]	Extracellular
P98095	Fibulin-2 precursor.	Extracellular
O75072	[Fukutin (EC 2.-.-.-) (Fukuyama-type congenital muscular dystrophy, protein).]	Extracellular
Q9UGM5	Fetuin-B precursor (Gugu) (IRL685) (16G2).	Extracellular
Q03591	[Complement factor H-related protein 1 precursor (FHR-1) (H factor-like, protein 1) (H-factor-like 1) (H36).]	Extracellular
Q12841	Follistatin-related protein 1 precursor (Follistatin-like 1).	Extracellular
P36222	[Chitinase-3-like protein 1 precursor (Cartilage glycoprotein 39) (GP-, 39) (39 kDa synovial protein) (HCgp-39) (YKL-40).]	Extracellular
P22352	[Glutathione peroxidase 3 precursor (EC 1.11.1.9) (GSHPx-3) (GPx-3), (Plasma glutathione peroxidase) (GSHPx-P) (Extracellular glutathione, peroxidase) (GPx-P).]	Extracellular
P02790	Hemopexin precursor (Beta-1B-glycoprotein).	Extracellular
P00738	[Haptoglobin precursor [Contains: Haptoglobin alpha chain; Haptoglobin, beta chain].]	Extracellular
P04196	[Histidine-rich glycoprotein precursor (Histidine-proline-rich, glycoprotein) (HPRG).]	Extracellular
P01767	Ig heavy chain V-III region BUT.	Extracellular
P01779	Ig heavy chain V-III region TUR.	Extracellular
P80419	Ig heavy chain V-III region GAR.	Extracellular
Q14627	[Interleukin-13 receptor alpha-2 chain precursor (Interleukin-13-, binding protein) (CD213a2 antigen).]	Extracellular
P14735	[Insulin-degrading enzyme (EC 3.4.24.56) (Insulysin) (Insulinase), (Insulin protease).]	Extracellular
Q14116	[Interleukin-18 precursor (IL-18) (Interferon-gamma-inducing factor), (IFN-gamma-inducing factor) (Interleukin-1 gamma) (IL-1 gamma), (Iboctadekin).]	Extracellular
P08887	[Interleukin-6 receptor alpha chain precursor (IL-6R-alpha) (IL-6R 1), (Membrane glycoprotein 80) (gp80) (CD126 antigen).]	Extracellular

P19827	[Inter-alpha-trypsin inhibitor heavy chain H1 precursor (ITI heavy, chain H1) (Inter-alpha-inhibitor heavy chain 1) (Inter-alpha-trypsin, inhibitor complex component III) (Serum-derived hyaluronan-associated, protein) (SHAP).]	Extracellular
Q14624	[Inter-alpha-trypsin inhibitor heavy chain H4 precursor (ITI heavy, chain H4) (Inter-alpha-inhibitor heavy chain 4) (Inter-alpha-trypsin, inhibitor family heavy chain-related protein) (IHRP) (Plasma, kallikrein sensitive glycoprotein 120) (PK-120) (GP120) [Contains: 70, kDa inter-alpha-trypsin inhibitor heavy chain H4; 35 kDa inter-alpha-, trypsin inhibitor heavy chain H4].]	Extracellular
P03952	[Plasma kallikrein precursor (EC 3.4.21.34) (Plasma prekallikrein), (Kininogenin) (Fletcher factor) [Contains: Plasma kallikrein heavy, chain; Plasma kallikrein light chain].]	Extracellular
Q9P0G3	[Kallikrein-14 precursor (EC 3.4.21.-) (Kallikrein-like protein 6), (KLK-L6).]	Extracellular
P01606	Ig kappa chain V-I region OU.	Extracellular
P01613	Ig kappa chain V-I region Ni.	Extracellular
P06309	Ig kappa chain V-II region GM607 precursor (Fragment).	Extracellular
P04433	Ig kappa chain V-III region VG precursor (Fragment).	Extracellular
P18428	Lipopolysaccharide-binding protein precursor (LBP).	Extracellular
P58215	[Lysyl oxidase homolog 3 precursor (EC 1.4.3.-) (Lysyl oxidase-like, protein 3).]	Extracellular
P01700	Ig lambda chain V-I region HA.	Extracellular
P01706	Ig lambda chain V-II region BOH.	Extracellular
Q16819	[Meprin A subunit alpha precursor (EC 3.4.24.18) (Endopeptidase-2) (N-, benzoyl-L-tyrosyl-P-amino-benzoic acid hydrolase subunit alpha) (PABA, peptide hydrolase) (PPH alpha).]	Extracellular
P14780	[Matrix metalloproteinase-9 precursor (EC 3.4.24.35) (MMP-9) (92 kDa, type IV collagenase) (92 kDa gelatinase) (Gelatinase B) (GELB), [Contains: 67 kDa matrix metalloproteinase-9; 82 kDa matrix, metalloproteinase-9].]	Extracellular
Q9H239	[Matrix metalloproteinase-28 precursor (EC 3.4.24.-) (MMP-28), (Epilysin).]	Extracellular
O00339	Matrilin-2 precursor.	Extracellular
P16519	[Neuroendocrine convertase 2 precursor (EC 3.4.21.94) (NEC 2) (PC2), (Prohormone convertase 2) (Proprotein convertase 2) (KEX2-like, endoprotease 2).]	Extracellular
P56975	[Pro-neuregulin-3, membrane-bound isoform precursor (Pro-NRG3), [Contains: Neuregulin-3 (NRG-3)].]	Extracellular
P10915	[Hyaluronan and proteoglycan link protein 1 precursor (Proteoglycan, link protein) (Cartilage link protein) (LP).]	Extracellular
P20742	Pregnancy zone protein precursor.	Extracellular
Q13123	Protein Red (Protein RER) (IK factor) (Cytokine IK).	Extracellular
Q02383	Semenogelin-2 precursor (Semenogelin II) (SGII).	Extracellular
Q14563	Semaphorin-3A precursor (Semaphorin III) (Sema III).	Extracellular
P78556	[Small-inducible cytokine A20 precursor (CCL20) (Macrophage, inflammatory protein 3 alpha) (MIP-3-alpha) (Liver and activation-, regulated chemokine) (CC chemokine LARC) (Beta chemokine exodus-1), [Contains: CCL20(1-67); CCL20(1-64); CCL20(2-70)].]	Extracellular
P05452	[Tetranectin precursor (TN) (C-type lectin domain family 3 member B), (Plasminogen kringle 4-binding protein).]	Extracellular
P05543	[Thyroxine-binding globulin precursor (T4-binding globulin) (Serpin, A7).]	Extracellular
P07477	[Trypsin-1 precursor (EC 3.4.21.4) (Trypsin I) (Cationic trypsinogen), (Serine protease 1).]	Extracellular
P04004	[Vitronectin precursor (Serum-spreading factor) (S-protein) (V75), [Contains: Vitronectin V65 subunit; Vitronectin V10 subunit;, Somatomedin B].]	Extracellular
P41221	Protein Wnt-5a precursor.	Extracellular
Q9Y6F9	Protein Wnt-6 precursor.	Extracellular

O15442	[Metallophosphoesterase domain-containing protein 1 (Adult brain, protein 239) (239AB).]	Go Component not found
P30154	[Serine/threonine-protein phosphatase 2A 65 kDa regulatory subunit A, beta isoform (PP2A, subunit A, PR65-beta isoform) (PP2A, subunit A,, R1-beta isoform).]	Go Component not found
Q9UPM8	[AP-4 complex subunit epsilon-1 (Adapter-related protein complex 4, subunit epsilon-1) (Epsilon subunit of AP-4) (AP-4 adapter complex, subunit epsilon).]	Go Component not found
O60423	[Probable phospholipid-transporting ATPase IK (EC 3.6.3.1) (ATPase, class I type 8B member 3).]	Go Component not found
Q9UGI9	[5'-AMP-activated protein kinase subunit gamma-3 (AMPK gamma-3 chain), (AMPK gamma3).]	Go Component not found
P16219	[Short-chain specific acyl-CoA dehydrogenase, mitochondrial precursor, (EC 1.3.99.2) (SCAD) (Butyryl-CoA dehydrogenase).]	Go Component not found
P11766	[Alcohol dehydrogenase class-3 (EC 1.1.1.1) (Alcohol dehydrogenase, class-III) (Alcohol dehydrogenase class chi chain) (S-,(hydroxymethyl)glutathione dehydrogenase) (EC 1.1.1.284) (Glutathione-, dependent formaldehyde dehydrogenase) (FDH).]	Go Component not found
P55198	Protein AF-17.	Go Component not found
Q9Y2D5	[A-kinase anchor protein 2 (Protein kinase A-anchoring protein 2), (PRKA2) (AKAP-2) (AKAP-KL).]	Go Component not found
P01876	Ig alpha-1 chain C region.	Go Component not found
Q16671	[Anti-Muellerian hormone type-2 receptor precursor (EC 2.7.11.30), (Anti-Muellerian hormone type II receptor) (AMH type II receptor) (MIS, type II receptor) (MISRII) (MRII).]	Go Component not found
Q01484	Ankyrin-2 (Brain ankyrin) (Ankyrin-B) (Ankyrin, nonerythroid).	Go Component not found
P51164	[Potassium-transporting ATPase subunit beta (Proton pump beta chain), (Gastric H(+)/K(+) ATPase beta subunit).]	Go Component not found
O95450	[ADAMTS-2 precursor (EC 3.4.24.14) (A disintegrin and metalloproteinase, with thrombospondin motifs 2) (ADAM-TS 2) (ADAM-TS2) (Procollagen I/II, amino propeptide-processing enzyme) (Procollagen I N-proteinase) (PC, I-NP) (Procollagen N-endopeptidase) (pNPI).]	Go Component not found
Q9UP79	[ADAMTS-8 precursor (EC 3.4.24.-) (A disintegrin and metalloproteinase, with thrombospondin motifs 8) (ADAM-TS 8) (ADAM-TS8) (METH-2) (METH-, 8).]	Go Component not found
P61769	[Beta-2-microglobulin precursor [Contains: Beta-2-microglobulin form pI, 5.3].]	Go Component not found
P54132	[Bloom syndrome protein (EC 3.6.1.-) (RecQ protein-like 3) (DNA, helicase, RecQ-like type 2).]	Go Component not found
P13497	[Bone morphogenetic protein 1 precursor (EC 3.4.24.19) (BMP-1), (Procollagen C-proteinase) (PCP) (Mammalian tolloid protein) (mTld).]	Go Component not found
P25440	Bromodomain-containing protein 2 (Protein RING3) (O27.1.1).	Go Component not found
Q9ULD4	Bromodomain and PHD finger-containing protein 3.	Go Component not found

**P09871**	[Complement C1s subcomponent precursor (EC 3.4.21.42) (C1 esterase), [Contains: Complement C1s subcomponent heavy chain; Complement C1s, subcomponent light chain].]	Go Component not found
**P02458**	[Collagen alpha-1(II) chain precursor (Alpha-1 type II collagen), [Contains: Chondrocalcin].]	Go Component not found
**P29377**	[Protein S100-G (S100 calcium-binding protein G) (Vitamin D-dependent, calcium-binding protein, intestinal) (CABP) (Calbindin-D9k).]	Go Component not found
**P19022**	[Cadherin-2 precursor (Neural-cadherin) (N-cadherin) (CD325 antigen), (CDw325).]	Go Component not found
**P55290**	[Cadherin-13 precursor (Truncated-cadherin) (T-cadherin) (T-cad), (Heart-cadherin) (H-cadherin) (P105).]	Go Component not found
**P07451**	[Carbonic anhydrase 3 (EC 4.2.1.1) (Carbonic anhydrase III) (Carbonate, dehydratase III) (CA-III).]	Go Component not found
**P32004**	Neural cell adhesion molecule L1 precursor (N-CAM L1) (CD171 antigen).	Go Component not found
**Q9UMQ6**	[Calpain-11 (EC 3.4.22.-) (Calcium-activated neutral proteinase 11), (CANP 11).]	Go Component not found
**Q01518**	Adenylyl cyclase-associated protein 1 (CAP 1).	Go Component not found
**Q9UN75**	Protocadherin alpha 12 precursor (PCDH-alpha12).	Go Component not found
**P51959**	Cyclin-G1 (Cyclin-G).	Go Component not found
**Q9NSE2**	[Cytokine-inducible SH2-containing protein (CIS) (CIS-1) (Suppressor of, cytokine signaling) (SOCS) (Protein G18).]	Go Component not found
**P51788**	Chloride channel protein 2 (ClC-2).	Go Component not found
**Q14123**	[Calcium/calmodulin-dependent 3',5'-cyclic nucleotide phosphodiesterase, 1C (EC 3.1.4.17) (Cam-PDE 1C) (hCam-3).]	Go Component not found
**Q14432**	[cGMP-inhibited 3',5'-cyclic phosphodiesterase A (EC 3.1.4.17) (Cyclic, GMP-inhibited phosphodiesterase A) (CGI-PDE A).]	Go Component not found
**Q99829**	Copine-1 (Copine I).	Go Component not found
**O43924**	[Retinal rod rhodopsin-sensitive cGMP 3',5'-cyclic phosphodiesterase, subunit delta (EC 3.1.4.17) (GMP-PDE delta) (p17 protein).]	Go Component not found
**P0C0L5**	[Complement C4-B precursor (Basic complement C4) [Contains: Complement, C4 beta chain; Complement C4-B alpha chain; C4a anaphylatoxin; C4b-B;, C4d-B; Complement C4 gamma chain].]	Go Component not found
**P07360**	Complement component C8 gamma chain precursor.	Go Component not found
**O00763**	[Acetyl-CoA carboxylase 2 (EC 6.4.1.2) (ACC-beta) [Includes: Biotin, carboxylase (EC 6.3.4.14)].]	Go Component not found
**P00395**	[Cytochrome c oxidase subunit 1 (EC 1.9.3.1) (Cytochrome c oxidase, polypeptide I).]	Go Component not found

**P22680**	[Cytochrome P450 7A1 (Cholesterol 7-alpha-monooxygenase) (CYPVII), (EC 1.14.13.17) (Cholesterol 7-alpha-hydroxylase).]	Go Component not found
**P10635**	[Cytochrome P450 2D6 (EC 1.14.14.1) (CYPIID6) (P450-DB1) (Debrisoquine, 4-hydroxylase).]	Go Component not found
**P48740**	[Complement-activating component of Ra-reactive factor precursor, (EC 3.4.21.-) (Ra-reactive factor serine protease p100) (RaRF), (Mannan-binding lectin serine protease 1) (Mannose-binding protein-, associated serine protease) (MASP-1) (Serine protease 5) [Contains:, Complement-activating component of Ra-reactive factor heavy chain;, Complement-activating component of Ra-reactive factor light chain].]	Go Component not found
**P82279**	Crumbs homolog 1 precursor.	Go Component not found
**Q9Y6H8**	Gap junction alpha-3 protein (Connexin-46) (Cx46).	Go Component not found
**Q14094**	Cyclin-I.	Go Component not found
**P33402**	Guanylate cyclase soluble subunit alpha-2 (EC 4.6.1.2) (GCS-alpha-2).	Go Component not found
**Q08752**	[40 kDa peptidyl-prolyl cis-trans isomerase (EC 5.2.1.8) (PPIase), (Rotamase) (Cyclophilin-40) (CYP-40) (Cyclophilin-related protein).]	Go Component not found
**P62940**	[Peptidyl-prolyl cis-trans isomerase A (EC 5.2.1.8) (PPIase A), (Rotamase A) (Cyclophilin A) (Cyclosporin A-binding protein).]	Go Component not found
**P04080**	Cystatin-B (Stefin-B) (Liver thiol proteinase inhibitor) (CPI-B).	Go Component not found
**P19113**	Histidine decarboxylase (EC 4.1.1.22) (HDC).	Go Component not found
**Q92466**	[DNA damage-binding protein 2 (Damage-specific DNA-binding protein 2), (DDB p48 subunit) (DDBb) (UV-damaged DNA-binding protein 2) (UV-DDB, 2).]	Go Component not found
**Q01524**	Defensin-6 precursor (Defensin, alpha 6).	Go Component not found
**O75828**	[Carbonyl reductase [NADPH] 3 (EC 1.1.1.184) (NADPH-dependent carbonyl, reductase 3).]	Go Component not found
**Q9UNQ2**	[Probable dimethyladenosine transferase (EC 2.1.1.-) (S-, adenosylmethionine-6-N',N'-adenosyl(rRNA) dimethyltransferase) (18S, rRNA dimethylase) (DIM1 dimethyladenosine transferase 1-like).]	Go Component not found
**P25686**	[DnaJ homolog subfamily B member 2 (Heat shock 40 kDa protein 3) (DnaJ, protein homolog 1) (HSJ-1).]	Go Component not found
**P49917**	[DNA ligase 4 (EC 6.5.1.1) (DNA ligase IV) (Polydeoxyribonucleotide, synthase [ATP] 4).]	Go Component not found
**Q9C005**	Dpy-30-like protein.	Go Component not found
**O60673**	DNA polymerase zeta catalytic subunit (EC 2.7.7.7) (hREV3).	Go Component not found
**O14531**	[Dihydropyrimidinase-related protein 4 (DRP-4) (Collapsin response, mediator protein 3) (CRMP-3) (UNC33-like phosphoprotein 4) (ULIP4, protein).]	Go Component not found

**P55039**	Developmentally-regulated GTP-binding protein 2 (DRG 2).	Go Component not found
**Q05193**	Dynamin-1 (EC 3.6.5.5).	Go Component not found
**Q05639**	[Elongation factor 1-alpha 2 (EF-1-alpha-2) (Elongation factor 1 A-2), (eEF1A-2) (Statin S1).]	Go Component not found
**P13929**	[Beta-enolase (EC 4.2.1.11) (2-phospho-D-glycerate hydro-lyase), (Muscle-specific enolase) (MSE) (Skeletal muscle enolase) (Enolase 3).]	Go Component not found
**P53602**	[Diphosphomevalonate decarboxylase (EC 4.1.1.33) (Mevalonate, pyrophosphate decarboxylase) (Mevalonate (diphospho)decarboxylase).]	Go Component not found
**Q03468**	[DNA excision repair protein ERCC-6 (EC 3.6.1.-) (ATP-dependent, helicase ERCC6) (Cockayne syndrome protein CSB).]	Go Component not found
**P41161**	ETS translocation variant 5 (Ets-related protein ERM).	Go Component not found
**P30042**	[ES1 protein homolog, mitochondrial precursor (Protein KNP-I) (Protein, GT335).]	Go Component not found
**P41212**	[Transcription factor ETV6 (ETS-related protein Tel1) (Tel) (ETS, translocation variant 6).]	Go Component not found
**P57679**	Ellis-van Creveld syndrome protein (DWF-1).	Go Component not found
**O43909**	[Exostosin-like 3 (EC 2.4.1.223) (Glucuronyl-galactosyl-proteoglycan 4-, alpha-N-acetylglucosaminyltransferase) (Putative tumor suppressor, protein EXTL3) (Multiple exostosis-like protein 3) (Hereditary, multiple exostoses gene isolog) (EXT-related protein 1).]	Go Component not found
**O60825**	[6-phosphofructo-2-kinase/fructose-2,6-biphosphatase 2 (6PF-2-K/Fru-, 2,6-P2ASE heart-type isozyme) (PFK-2/FBPase-2) [Includes: 6-, phosphofructo-2-kinase (EC 2.7.1.105); Fructose-2,6-bisphosphatase, (EC 3.1.3.46)].]	Go Component not found
**P07332**	Proto-oncogene tyrosine-protein kinase Fes/Fps (EC 2.7.10.2) (C-Fes).	Go Component not found
**P36980**	[Complement factor H-related protein 2 precursor (FHR-2) (H factor-like, protein 2) (H factor-like 3) (DDESK59).]	Go Component not found
**Q14318**	[FK506-binding protein 8 (EC 5.2.1.8) (Peptidyl-prolyl cis-trans, isomerase) (PPIase) (Rotamase) (38 kDa FK506-binding protein), (FKBPR38) (hFKBP38).]	Go Component not found
**P30043**	[Flavin reductase (EC 1.5.1.30) (FR) (NADPH-dependent diaphorase), (NADPH-flavin reductase) (FLR) (Biliverdin reductase B) (EC 1.3.1.24), (BVR-B) (Biliverdin-IX beta-reductase) (Green heme-binding protein), (GHBP).]	Go Component not found
**P51816**	[AF4/FMR2 family member 2 (Fragile X mental retardation 2 protein), (Protein FMR-2) (FMR2P) (Protein Ox19) (Fragile X E mental retardation, syndrome protein).]	Go Component not found
**O75891**	[10-formyltetrahydrofolate dehydrogenase (EC 1.5.1.6) (10-FTHFDH), (Aldehyde dehydrogenase 1 family member L1).]	Go Component not found
**O00591**	[Gamma-aminobutyric-acid receptor subunit pi precursor (GABA(A), receptor subunit pi).]	Go Component not found
**O14976**	Cyclin G-associated kinase (EC 2.7.11.1).	Go Component not found
**P78537**	[Biogenesis of lysosome-related organelles complex-1 subunit 1 (BLOC-1, subunit 1) (GCN5-like protein 1) (RT14 protein).]	Go Component not found

Q14397	Glucokinase regulatory protein (Glucokinase regulator).	Go Component not found
Q9NR23	Growth/differentiation factor 3 precursor (GDF-3).	Go Component not found
P52306	[Rap1 GTPase-GDP dissociation stimulator 1 (SMG P21 stimulatory GDP/GTP, exchange protein) (SMG GDS protein) (Exchange factor smgGDS).]	Go Component not found
Q13070	G antigen 6 (GAGE-6).	Go Component not found
P04062	[Glucosylceramidase precursor (EC 3.2.1.45) (Beta-glucocerebrosidase), (Acid beta-glucosidase) (D-glucosyl-N-acylsphingosine glucohydrolase), (Alglucerase) (Imiglucerase).]	Go Component not found
P10070	Zinc finger protein GLI2 (Tax helper protein).	Go Component not found
Q12967	Ral guanine nucleotide dissociation stimulator (RalGEF) (RalGDS).	Go Component not found
Q9NYM4	[Probable G-protein coupled receptor 83 precursor (G-protein coupled, receptor 72).]	Go Component not found
Q14451	[Growth factor receptor-bound protein 7 (GRB7 adapter protein), (Epidermal growth factor receptor GRB-7) (B47).]	Go Component not found
Q08117	[Amino-terminal enhancer of split (Amino enhancer of split) (GRG, protein) (Protein ESP1) (Gp130-associated protein GAM).]	Go Component not found
O00178	GTP-binding protein 1 (G-protein 1) (GP-1) (GP1).	Go Component not found
P30712	Glutathione S-transferase theta-2 (EC 2.5.1.18) (GST class-theta-2).	Go Component not found
P84243	Histone H3.3.	Go Component not found
O60229	[Kalirin (Huntingtin-associated protein-interacting protein) (Protein, Duo).]	Go Component not found
P05537	HLA class II histocompatibility antigen, DQ(W3) beta chain precursor.	Go Component not found
P61921	[Hemoglobin subunit gamma-2 (Hemoglobin gamma-2 chain) (Gamma-2-globin), (Hemoglobin gamma-G chain).]	Go Component not found
P69891	[Hemoglobin subunit gamma-1 (Hemoglobin gamma-1 chain) (Gamma-1-globin), (Hemoglobin gamma-A chain) (Hb F Agamma).]	Go Component not found
Q9Y5J3	[Hairy/enhancer-of-split related with YRPW motif protein 1 (Hairy and, enhancer of split-related protein 1) (HESR-1) (Hairy-related, transcription factor 1) (hHRT1) (Cardiovascular helix-loop-helix, factor 2) (CHF-2) (HES-related repressor protein 1).]	Go Component not found
P10074	[Zinc finger and BTB domain-containing protein 48 (Krueppel-related, zinc finger protein 3) (Protein HKR3).]	Go Component not found
P09067	Homeobox protein Hox-B5 (Hox-2A) (HHO.C10) (HU-1).	Go Component not found
P30519	Heme oxygenase 2 (EC 1.14.99.3) (HO-2).	Go Component not found

**P00739**	Haptoglobin-related protein precursor.	Go Component not found
**P17066**	Heat shock 70 kDa protein 6 (Heat shock 70 kDa protein B').	Go Component not found
**Q03933**	[Heat shock factor protein 2 (HSF 2) (Heat shock transcription factor, 2) (HSTF 2).]	Go Component not found
**O43364**	Homeobox protein Hox-A2.	Go Component not found
**P31271**	Homeobox protein Hox-A13 (Hox-1J).	Go Component not found
**P31274**	Homeobox protein Hox-C9 (Hox-3B).	Go Component not found
**P31275**	Homeobox protein Hox-C12 (Hox-3F).	Go Component not found
**P19367**	[Hexokinase-1 (EC 2.7.1.1) (Hexokinase type I) (HK I) (Brain form, hexokinase).]	Go Component not found
**P07099**	[Epoxide hydrolase 1 (EC 3.3.2.9) (Microsomal epoxide hydrolase), (Epoxide hydratase).]	Go Component not found
**P29466**	[Caspase-1 precursor (EC 3.4.22.36) (CASP-1) (Interleukin-1 beta, convertase) (IL-1BC) (IL-1 beta-converting enzyme) (ICE) (Interleukin-, 1 beta-converting enzyme) (p45) [Contains: Caspase-1 subunit p20;, Caspase-1 subunit p10].]	Go Component not found
**Q01101**	Insulinoma-associated protein 1 (Zinc finger protein IA-1).	Go Component not found
**P18065**	[Insulin-like growth factor-binding protein 2 precursor (IGFBP-2) (IBP-, 2) (IGF-binding protein 2).]	Go Component not found
**P31944**	[Caspase-14 precursor (EC 3.4.22.-) (CASP-14) [Contains: Caspase-14, subunit 1; Caspase-14 subunit 2].]	Go Component not found
**O75821**	[Eukaryotic translation initiation factor 3 subunit 4 (eIF-3 delta), (eIF3 p44) (eIF-3 RNA-binding subunit) (eIF3 p42) (eIF3g).]	Go Component not found
**P01344**	[Insulin-like growth factor II precursor (IGF-II) (Somatomedin A), [Contains: Insulin-like growth factor II Ala-25 Del].]	Go Component not found
**Q15653**	[NF-kappa-B inhibitor beta (NF-kappa-BIB) (I-kappa-B-beta), (IkappaBbeta) (IKB-beta) (IKB-B) (Thyroid receptor-interacting protein, 9) (TRIP-9) (TR-interacting protein 9).]	Go Component not found
**P20839**	[Inosine-5'-monophosphate dehydrogenase 1 (EC 1.1.1.205) (IMP, dehydrogenase 1) (IMPDH-I) (IMPD 1).]	Go Component not found
**P49441**	Inositol polyphosphate 1-phosphatase (EC 3.1.3.57) (IPPase) (IPP).	Go Component not found
**P27987**	[Inositol-trisphosphate 3-kinase B (EC 2.7.1.127) (Inositol 1,4,5-, trisphosphate 3-kinase B) (IP3K B) (IP3 3-kinase B) (IP3K-B).]	Go Component not found
**P10745**	[Interphotoreceptor retinoid-binding protein precursor (IRBP), (Interstitial retinol-binding protein).]	Go Component not found
**Q14653**	Interferon regulatory factor 3 (IRF-3).	Go Component not found

**Q9Y4H2**	Insulin receptor substrate 2 (IRS-2).	Go Component not found
**Q13683**	[Integrin alpha-7 precursor [Contains: Integrin alpha-7 heavy chain;, Integrin alpha-7 light chain].]	Go Component not found
**Q08881**	[Tyrosine-protein kinase ITK/TSK (EC 2.7.10.2) (T-cell-specific kinase), (Tyrosine-protein kinase Lyk) (Kinase EMT).]	Go Component not found
**P35527**	[Keratin, type I cytoskeletal 9 (Cytokeratin-9) (CK-9) (Keratin-9), (K9).]	Go Component not found
**O76014**	Keratin, type I cuticular Ha7 (Hair keratin, type I Ha7).	Go Component not found
**P51812**	[Ribosomal protein S6 kinase alpha-3 (EC 2.7.11.1) (S6K-alpha 3) (90, kDa ribosomal protein S6 kinase 3) (p90-RSK 3) (Ribosomal S6 kinase 2), (RSK-2) (pp90RSK2) (Insulin-stimulated protein kinase 1) (ISPK-1) (MAP, kinase-activated protein kinase 1b) (MAPKAPK1B).]	Go Component not found
**P01834**	Ig kappa chain C region.	Go Component not found
**P52824**	[Diacylglycerol kinase theta (EC 2.7.1.107) (Diglyceride kinase theta), (DGK-theta) (DAG kinase theta).]	Go Component not found
**O75840**	Krueppel-like factor 7 (Ubiquitous krueppel-like factor).	Go Component not found
**P06870**	[Kallikrein-1 precursor (EC 3.4.21.35) (Tissue kallikrein), (Kidney/pancreas/salivary gland kallikrein).]	Go Component not found
**P24723**	Protein kinase C eta type (EC 2.7.11.13) (nPKC-eta) (PKC-L).	Go Component not found
**P01842**	Ig lambda chain C regions.	Go Component not found
**O60488**	[Long-chain-fatty-acid--CoA ligase 4 (EC 6.2.1.3) (Long-chain acyl-CoA, synthetase 4) (LACS 4).]	Go Component not found
**P81274**	G-protein-signaling modulator 2 (Mosaic protein LGN).	Go Component not found
**Q8WWY8**	[Lipase member H precursor (EC 3.1.1.-) (Membrane-associated, phosphatidic acid-selective phospholipase A1-alpha) (mPA-PLA1 alpha), (LPD lipase-related protein) (Phospholipase A1 member B).]	Go Component not found
**O15230**	Laminin subunit alpha-5 precursor.	Go Component not found
**Q9UQQ2**	[SH2B adapter protein 3 (Lymphocyte-specific adapter protein Lnk), (Signal transduction protein Lnk) (Lymphocyte adapter protein).]	Go Component not found
**O60711**	Leupaxin.	Go Component not found
**O00754**	[Lysosomal alpha-mannosidase precursor (EC 3.2.1.24) (Mannosidase,, alpha B) (Lysosomal acid alpha-mannosidase) (Laman) (Mannosidase alpha, class 2B member 1) [Contains: Lysosomal alpha-mannosidase A peptide;, Lysosomal alpha-mannosidase B peptide; Lysosomal alpha-mannosidase C, peptide; Lysosomal alpha-mannosidase D peptide; Lysosomal alpha-, mannosidase E peptide].]	Go Component not found

**Q13233**	[Mitogen-activated protein kinase kinase kinase 1 (EC 2.7.11.25), (MAPK/ERK kinase kinase 1) (MEK kinase 1) (MEKK 1).]	Go Component not found
**O43318**	[Mitogen-activated protein kinase kinase kinase 7 (EC 2.7.11.25), (Transforming growth factor-beta-activated kinase 1) (TGF-beta-, activated kinase 1).]	Go Component not found
**P43363**	Melanoma-associated antigen 10 (MAGE-10 antigen).	Go Component not found
**O00187**	[Mannan-binding lectin serine protease 2 precursor (EC 3.4.21.104), (Mannose-binding protein-associated serine protease 2) (MASP-2) (MBL-, associated serine protease 2) [Contains: Mannan-binding lectin serine, protease 2 A chain; Mannan-binding lectin serine protease 2 B chain].]	Go Component not found
**P61244**	Protein max (Myc-associated factor X).	Go Component not found
**Q9UI95**	[Mitotic spindle assembly checkpoint protein MAD2B (MAD2-like 2), (hREV7).]	Go Component not found
**O15320**	[Cutaneous T-cell lymphoma-associated antigen 5 (cTAGE-5 protein), (cTAGE family member 5) (Meningioma-expressed antigen 6/11).]	Go Component not found
**O00470**	Homeobox protein Meis1.	Go Component not found
**Q99707**	[Methionine synthase (EC 2.1.1.13) (5-methyltetrahydrofolate--, homocysteine methyltransferase) (Methionine synthase, vitamin-B12, dependent) (MS).]	Go Component not found
**O43451**	[Maltase-glucoamylase, intestinal [Includes: Maltase (EC 3.2.1.20), (Alpha-glucosidase); Glucoamylase (EC 3.2.1.3) (Glucan 1,4-alpha-, glucosidase)].]	Go Component not found
**P28482**	[Mitogen-activated protein kinase 1 (EC 2.7.11.24) (Extracellular, signal-regulated kinase 2) (ERK-2) (Mitogen-activated protein kinase, 2) (MAP kinase 2) (MAPK 2) (p42-MAPK) (ERT1).]	Go Component not found
**P53779**	[Mitogen-activated protein kinase 10 (EC 2.7.11.24) (Stress-activated, protein kinase JNK3) (c-Jun N-terminal kinase 3) (MAP kinase p49, 3F12).]	Go Component not found
**Q99583**	Max-binding protein MNT (Protein ROX) (Myc antagonist MNT).	Go Component not found
**P50221**	Homeobox protein MOX-1 (Mesenchyme homeobox 1).	Go Component not found
**P42898**	Methylenetetrahydrofolate reductase (EC 1.5.1.20).	Go Component not found
**Q9Y216**	Myotubularin-related protein 7 (EC 3.1.3.-).	Go Component not found
**P12882**	[Myosin-1 (Myosin heavy chain 1) (Myosin heavy chain 2x) (MyHC-2x), (Myosin heavy chain, skeletal muscle, adult 1) (Myosin heavy chain, IIx/d) (MyHC-IIx/d).]	Go Component not found
**Q9Y623**	[Myosin-4 (Myosin heavy chain 4) (Myosin heavy chain 2b) (MyHC-2b), (Myosin heavy chain, skeletal muscle, fetal) (Myosin heavy chain IIb), (MyHC-IIb).]	Go Component not found
**O14732**	[Inositol monophosphatase 2 (EC 3.1.3.25) (IMPase 2) (IMP 2) (Inositol-, 1(or 4)-monophosphatase 2) (Myo-inositol monophosphatase A2).]	Go Component not found
**Q00872**	[Myosin-binding protein C, slow-type (Slow MyBP-C) (C-protein, skeletal, muscle slow isoform).]	Go Component not found

**Q9UPR5**	[Sodium/calcium exchanger 2 precursor (Na(+)/Ca(2+)-exchange protein, 2).]	Go Component not found
**P17050**	[Alpha-N-acetylgalactosaminidase precursor (EC 3.2.1.49) (Alpha-, galactosidase B).]	Go Component not found
**Q15784**	[Neurogenic differentiation factor 2 (NeuroD2) (NeuroD-related factor), (NDRF).]	Go Component not found
**O76041**	Nebulette (Actin-binding Z-disk protein).	Go Component not found
**Q92832**	[Protein kinase C-binding protein NELL1 precursor (NEL-like protein 1), (Nel-related protein 1).]	Go Component not found
**Q92686**	Neurogranin (Ng) (RC3).	Go Component not found
**Q14938**	[Nuclear factor 1 X-type (Nuclear factor 1/X) (NF1-X) (NFI-X) (NF-I/X), (CCAAT-box-binding transcription factor) (CTF) (TGGCA-binding, protein).]	Go Component not found
**Q16236**	[Nuclear factor erythroid 2-related factor 2 (NF-E2-related factor 2), (NFE2-related factor 2) (Nuclear factor, erythroid derived 2, like 2), (HEBP1).]	Go Component not found
**Q9UJF2**	Ras GTPase-activating protein nGAP (RAS protein activator-like 1).	Go Component not found
**Q99784**	[Noelin precursor (Neuronal olfactomedin-related ER localized protein), (Olfactomedin-1).]	Go Component not found
**Q99743**	[Neuronal PAS domain-containing protein 2 (Neuronal PAS2) (Member of, PAS protein 4) (Basic-helix-loop-helix-PAS protein MOP4).]	Go Component not found
**Q9NQX5**	[Neural proliferation differentiation and control protein 1 precursor, (NPDC-1).]	Go Component not found
**Q9Y466**	[Orphan nuclear receptor NR2E1 (Nuclear receptor TLX) (Tailless, homolog) (Tll) (hTll).]	Go Component not found
**Q9GZK3**	Olfactory receptor 2B2 (Olfactory receptor 6-1) (OR6-1) (Hs6M1-10).	Go Component not found
**O95222**	[Olfactory receptor 6A2 (Olfactory receptor OR11-83) (Olfactory, receptor 11-55) (OR11-55) (hP2 olfactory receptor).]	Go Component not found
**Q9H209**	Olfactory receptor 10A4 (HP2) (Olfactory receptor-like protein JCG5).	Go Component not found
**Q02509**	Otoconin 90 precursor (Oc90) (Phospholipase A2 homolog).	Go Component not found
**Q12889**	[Oviduct-specific glycoprotein precursor (Oviductal glycoprotein), (Oviductin) (Estrogen-dependent oviduct protein) (Mucin-9).]	Go Component not found
**O95948**	[One cut domain family member 2 (Transcription factor ONECUT-2) (OC-2), (Hepatocyte nuclear factor 6-beta) (HNF-6-beta).]	Go Component not found
**Q9UBL9**	P2X purinoceptor 2 (ATP receptor) (P2X2) (Purinergic receptor).	Go Component not found

**P54886**	[Delta-1-pyrroline-5-carboxylate synthetase (P5CS) (Aldehyde, dehydrogenase 18 family member A1) [Includes: Glutamate 5-kinase, (EC 2.7.2.11) (Gamma-glutamyl kinase) (GK); Gamma-glutamyl phosphate, reductase (GPR) (EC 1.2.1.41) (Glutamate-5-semialdehyde dehydrogenase), (Glutamyl-gamma-semialdehyde dehydrogenase)].]	Go Component not found
**O00459**	[Phosphatidylinositol 3-kinase regulatory subunit beta (PI3-kinase p85, subunit beta) (PtdIns-3-kinase p85-beta).]	Go Component not found
**Q02962**	Paired box protein Pax-2.	Go Component not found
**P42785**	[Lysosomal Pro-X carboxypeptidase precursor (EC 3.4.16.2), (Prolylcarboxypeptidase) (PRCP) (Proline carboxypeptidase), (Angiotensinase C) (Lysosomal carboxypeptidase C).]	Go Component not found
**Q16342**	[Programmed cell death protein 2 (Zinc finger protein Rp-8) (Zinc, finger MYND domain-containing protein 7).]	Go Component not found
**Q9Y2J8**	[Protein-arginine deiminase type-2 (EC 3.5.3.15) (Protein-arginine, deiminase type II) (Peptidylarginine deiminase II) (PAD-H19).]	Go Component not found
**O15055**	[Period circadian protein homolog 2 (Circadian clock protein PERIOD 2), (hPER2).]	Go Component not found
**P16112**	[Aggrecan core protein precursor (Cartilage-specific proteoglycan core, protein) (CSPCP) (Chondroitin sulfate proteoglycan core protein 1), [Contains: Aggrecan core protein 2].]	Go Component not found
**P00439**	[Phenylalanine-4-hydroxylase (EC 1.14.16.1) (PAH) (Phe-4-, monooxygenase).]	Go Component not found
**Q9H0N5**	[Pterin-4-alpha-carbinolamine dehydratase 2 (EC 4.2.1.96) (PHS 2) (4-, alpha-hydroxy-tetrahydropterin dehydratase 2) (DcoH-like protein, DCoHm) (Dimerization cofactor of hepatocyte nuclear factor 1 from, muscle) (HNF1-alpha dimerization cofactor).]	Go Component not found
**P08567**	Pleckstrin (Platelet p47 protein).	Go Component not found
**Q9HCL2**	[Glycerol-3-phosphate acyltransferase, mitochondrial precursor, (EC 2.3.1.15) (GPAT).]	Go Component not found
**Q15155**	Nodal modulator 1 precursor (pM5).	Go Component not found
**P10266**	[HERV-K_5q33.3 provirus ancestral Pol protein (HERV-K10 Pol protein), (HERV-K107 Pol protein) [Includes: Reverse transcriptase (RT), (EC 2.7.7.49); Ribonuclease H (EC 3.1.26.4) (RNase H); Integrase, (IN)].]	Go Component not found
**Q9BZG2**	Testicular acid phosphatase precursor (EC 3.1.3.2).	Go Component not found
**O14830**	[Serine/threonine-protein phosphatase with EF-hands 2 (EC 3.1.3.16), (PPEF-2).]	Go Component not found
**Q92674**	[Centromere protein I (CENP-I) (Interphase centromere complex protein, 19) (Follicle-stimulating hormone primary response protein) (FSH, primary response protein 1) (Leucine-rich primary response protein 1).]	Go Component not found
**P02812**	[Basic salivary proline-rich protein 2 (Salivary proline-rich protein), (Con1 glycoprotein) [Contains: Basic peptide P-F] (Fragment).]	Go Component not found
**P20472**	Parvalbumin alpha.	Go Component not found
**Q13046**	Pregnancy-specific beta-1-glycoprotein 7 precursor (PSBG-7).	Go Component not found

**P22234**	[Multifunctional protein ADE2 [Includes: Phosphoribosylaminoimidazole-, succinocarboxamide synthase (EC 6.3.2.6) (SAICAR synthetase);, Phosphoribosylaminoimidazole carboxylase (EC 4.1.1.21) (AIR, carboxylase) (AIRC)].]	Go Component not found
**P11172**	[Uridine 5'-monophosphate synthase (UMP synthase) [Includes: Orotate, phosphoribosyltransferase (EC 2.4.2.10) (OPRTase); Orotidine 5'-, phosphate decarboxylase (EC 4.1.1.23) (OMPdecase)].]	Go Component not found
**P62820**	Ras-related protein Rab-1A (YPT1-related protein).	Go Component not found
**Q92930**	Ras-related protein Rab-8B.	Go Component not found
**P28749**	[Retinoblastoma-like protein 1 (107 kDa retinoblastoma-associated, protein) (PRB1) (P107).]	Go Component not found
**Q92781**	11-cis retinol dehydrogenase (EC 1.1.1.105) (11-cis RDH).	Go Component not found
**P04090**	Prorelaxin H2 precursor [Contains: Relaxin B chain; Relaxin A chain].	Go Component not found
**P22670**	MHC class II regulatory factor RFX1 (RFX) (Enhancer factor C) (EF-C).	Go Component not found
**O15211**	[Ral guanine nucleotide dissociation stimulator-like 2 (RalGDS-like, factor) (RAS-associated protein RAB2L).]	Go Component not found
**O43566**	Regulator of G-protein signaling 14 (RGS14).	Go Component not found
**P61587**	[Rho-related GTP-binding protein RhoE precursor (Rho family GTPase 3), (Rnd3) (Rho8) (MemB protein).]	Go Component not found
**P18621**	60S ribosomal protein L17 (L23).	Go Component not found
**P28067**	[HLA class II histocompatibility antigen, DM alpha chain precursor (MHC, class II antigen DMA).]	Go Component not found
**P51449**	[Nuclear receptor ROR-gamma (Retinoid-related orphan receptor-gamma), (Nuclear receptor RZR-gamma).]	Go Component not found
**Q9UET6**	[Putative ribosomal RNA methyltransferase 1 (EC 2.1.1.-) (rRNA, (uridine-2'-O-)-methyltransferase).]	Go Component not found
**Q9UI43**	[Putative ribosomal RNA methyltransferase 2 (EC 2.1.1.-) (rRNA, (uridine-2'-O-)-methyltransferase).]	Go Component not found
**Q96F10**	[Diamine acetyltransferase 2 (EC 2.3.1.57) (Spermidine/spermine N(1)-, acetyltransferase 2) (Polyamine N-acetyltransferase 2).]	Go Component not found
**P51172**	[Amiloride-sensitive sodium channel subunit delta (Epithelial Na(+), channel subunit delta) (Delta ENaC) (Nonvoltage-gated sodium channel 1, subunit delta) (SCNED) (Delta NaCH).]	Go Component not found
**P55809**	[Succinyl-CoA:3-ketoacid-coenzyme A transferase 1, mitochondrial, precursor (EC 2.8.3.5) (Somatic-type succinyl CoA:3-oxoacid CoA-, transferase) (Scot-S).]	Go Component not found
**Q14141**	Septin-6.	Go Component not found
**P23246**	[Splicing factor, proline- and glutamine-rich (Polypyrimidine tract-, binding protein-associated-splicing factor) (PTB-associated-splicing, factor) (PSF) (DNA-binding p52/p100 complex, 100 kDa subunit) (100 kDa, DNA-pairing protein) (hPOMp100).]	Go Component not found

**Q12872**	[Splicing factor, arginine/serine-rich 8 (Suppressor of white apricot, protein homolog).]	Go Component not found
**O95905**	SGT1 protein (hSGT1) (Ecdysoneless homolog) (Suppressor of GCR2).	Go Component not found
**Q14190**	Single-minded homolog 2.	Go Component not found
**Q9H3T3**	Semaphorin-6B precursor (Semaphorin Z) (Sema Z).	Go Component not found
**Q9BY66**	[Histone demethylase JARID1D (EC 1.14.11.-) (Jumonji/ARID domain-, containing protein 1D) (Protein SmcY) (Histocompatibility Y antigen), (H-Y).]	Go Component not found
**P54920**	[Alpha-soluble NSF attachment protein (SNAP-alpha) (N-ethylmaleimide-, sensitive factor attachment protein, alpha).]	Go Component not found
**Q9Y5W8**	[Sorting nexin-13 (RGS domain- and PHOX domain-containing protein), (RGS-PX1).]	Go Component not found
**P51688**	[N-sulphoglucosamine sulphohydrolase precursor (EC 3.10.1.1), (Sulfoglucosamine sulfamidase) (Sulphamidase).]	Go Component not found
**P52788**	[Spermine synthase (EC 2.5.1.22) (Spermidine aminopropyltransferase), (SPMSY).]	Go Component not found
**P12931**	[Proto-oncogene tyrosine-protein kinase Src (EC 2.7.10.2) (p60-Src) (c-, Src) (pp60c-src).]	Go Component not found
**P61266**	Syntaxin-1B (Syntaxin-1B1) (Syntaxin-1B2).	Go Component not found
**Q9BXU1**	[Serine/threonine-protein kinase 31 (EC 2.7.11.1) (Serine/threonine-, protein kinase NYD-SPK) (Sugen kinase 396).]	Go Component not found
**P42226**	Signal transducer and activator of transcription 6 (IL-4 Stat).	Go Component not found
**O76039**	[Cyclin-dependent kinase-like 5 (EC 2.7.11.22) (Serine/threonine-, protein kinase 9).]	Go Component not found
**Q13277**	Syntaxin-3.	Go Component not found
**P51687**	Sulfite oxidase, mitochondrial precursor (EC 1.8.3.1).	Go Component not found
**O95416**	Transcription factor SOX-14.	Go Component not found
**O43426**	[Synaptojanin-1 (EC 3.1.3.36) (Synaptic inositol-1,4,5-trisphosphate 5-, phosphatase 1).]	Go Component not found
**P17600**	Synapsin-1 (Synapsin I) (Brain protein 4.1).	Go Component not found
**Q16143**	Beta-synuclein.	Go Component not found
**Q9Y6Q2**	Stonin-1 (Stoned B-like factor).	Go Component not found

**P13984**	[Transcription initiation factor IIF subunit beta (EC 3.6.1.-) (TFIIF-, beta) (ATP-dependent helicase GTF2F2) (General transcription factor, IIF subunit 2) (Transcription initiation factor RAP30).]	Go Component not found
**P62079**	[Tetraspanin-5 (Tspan-5) (Transmembrane 4 superfamily member 9), (Tetraspan NET-4).]	Go Component not found
**Q9HD45**	[Transmembrane 9 superfamily protein member 3 precursor (SM-11044-, binding protein) (EP70-P-iso).]	Go Component not found
**P17542**	[T-cell acute lymphocytic leukemia protein 1 (TAL-1) (Stem cell, protein) (T-cell leukemia/lymphoma protein 5).]	Go Component not found
**P68368**	[Tubulin alpha-4A chain (Tubulin alpha-4 chain) (Alpha-tubulin 4), (Alpha-tubulin isotype M-alpha-4).]	Go Component not found
**Q9Y4P3**	[Transducin beta-like 2 protein (WS beta-transducin repeats protein), (WS-betaTRP) (Williams-Beuren syndrome chromosome region 13 protein).]	Go Component not found
**P37275**	[Zinc finger E-box-binding homeobox 1 (Transcription factor 8) (NIL-2-A, zinc finger protein) (Negative regulator of IL2).]	Go Component not found
**Q9BXT4**	Tudor domain-containing protein 1.	Go Component not found
**Q92664**	Transcription factor IIIA (Factor A) (TFIIIA).	Go Component not found
**Q13888**	[TFIIH basal transcription factor complex p44 subunit (Basic, transcription factor 2 44 kDa subunit) (BTF2-p44) (General, transcription factor IIH polypeptide 2).]	Go Component not found
**P31483**	[Nucleolysin TIA-1 isoform p40 (RNA-binding protein TIA-1) (p40-TIA-1), [Contains: Nucleolysin TIA-1 isoform p15 (p15-TIA-1)].]	Go Component not found
**P47974**	[Butyrate response factor 2 (Protein TIS11D) (EGF-response factor 2), (ERF-2).]	Go Component not found
**P82094**	TATA element modulatory factor (TMF).	Go Component not found
**P50616**	Protein Tob1 (Transducer of erbB-2 1).	Go Component not found
**P00750**	[Tissue-type plasminogen activator precursor (EC 3.4.21.68) (tPA) (t-, PA) (t-plasminogen activator) (Alteplase) (Reteplase) [Contains:, Tissue-type plasminogen activator chain A; Tissue-type plasminogen, activator chain B].]	Go Component not found
**Q12931**	[Heat shock protein 75 kDa, mitochondrial precursor (HSP 75) (Tumor, necrosis factor type 1 receptor-associated protein) (TRAP-1) (TNFR-, associated protein 1).]	Go Component not found
**Q9UMR3**	T-box transcription factor TBX20 (T-box protein 20).	Go Component not found
**P19971**	[Thymidine phosphorylase precursor (EC 2.4.2.4) (TdRPase) (TP), (Platelet-derived endothelial cell growth factor) (PD-ECGF), (Gliostatin).]	Go Component not found
**P51784**	[Ubiquitin carboxyl-terminal hydrolase 11 (EC 3.1.2.15) (Ubiquitin, thioesterase 11) (Ubiquitin-specific-processing protease 11), (Deubiquitinating enzyme 11).]	Go Component not found
**Q9Y2K6**	[Ubiquitin carboxyl-terminal hydrolase 20 (EC 3.1.2.15) (Ubiquitin, thioesterase 20) (Ubiquitin-specific-processing protease 20), (Deubiquitinating enzyme 20).]	Go Component not found
**O75534**	[Cold shock domain-containing protein E1 (UNR protein) (N-ras upstream, gene protein).]	Go Component not found

P00749	[Urokinase-type plasminogen activator precursor (EC 3.4.21.73) (uPA), (U-plasminogen activator) [Contains: Urokinase-type plasminogen, activator long chain A; Urokinase-type plasminogen activator short, chain A; Urokinase-type plasminogen activator chain B].]	Go Component not found
P21281	[Vacuolar ATP synthase subunit B, brain isoform (EC 3.6.3.14) (V-ATPase, subunit B 2) (Vacuolar proton pump subunit B 2) (Endomembrane proton, pump 58 kDa subunit) (HO57).]	Go Component not found
Q9UKW4	Protein vav-3.	Go Component not found
P37235	[Hippocalcin-like protein 1 (Visinin-like protein 3) (VILIP-3), (Calcium-binding protein BDR-1) (HLP2).]	Go Component not found
Q9UPY6	[Wiskott-Aldrich syndrome protein family member 3 (WASP-family protein, member 3) (Protein WAVE-3) (Verprolin homology domain-containing, protein 3).]	Go Component not found
Q9NNW5	WD repeat-containing protein 6.	Go Component not found
Q9GZT5	Protein Wnt-10a precursor.	Go Component not found
Q93098	Protein Wnt-8b precursor.	Go Component not found
Q15051	IQ calmodulin-binding motif-containing protein 1 (Nephrocystin-5).	Go Component not found
Q14156	Protein EFR3-like.	Go Component not found
Q14687	Genetic suppressor element 1.	Go Component not found
Q12768	Strumpellin.	Go Component not found
Q92618	Zinc finger protein 516.	Go Component not found
Q92561	[Phytanoyl-CoA hydroxylase-interacting protein (Phytanoyl-CoA, hydroxylase-associated protein 1) (PAHXAP1) (PAHX-AP1).]	Go Component not found
O15040	Uncharacterized protein KIAA0329/KIAA0297.	Go Component not found
O43313	Uncharacterized protein KIAA0431.	Go Component not found
O60269	G protein-regulated inducer of neurite outgrowth 2 (GRIN2).	Go Component not found
O94819	[Kelch repeat and BTB domain-containing protein 11 (Kelch domain-, containing protein 7B).]	Go Component not found
O94933	SLIT and NTRK-like protein 3 precursor.	Go Component not found
Q9Y2K5	R3H domain-containing protein 2.	Go Component not found

**Q9UPW6**	[DNA-binding protein SATB2 (Special AT-rich sequence-binding protein, 2).]	Go Component not found
**Q9P2Q2**	FERM domain-containing protein 4A.	Go Component not found
**P52740**	Zinc finger protein 132.	Go Component not found
**P52747**	Zinc finger protein 143 (SPH-binding factor).	Go Component not found
**Q9ULX5**	[Zinc finger protein 179 (Brain finger protein) (RING finger protein, 112).]	Go Component not found
**O14771**	[Zinc finger protein 213 (Putative transcription factor CR53) (Zinc, finger protein with KRAB and SCAN domains 21).]	Go Component not found
**Q9UK11**	Zinc finger protein 223.	Go Component not found
**Q14588**	Zinc finger protein 234 (Zinc finger protein HZF4).	Go Component not found
**O43296**	Zinc finger protein 264.	Go Component not found
**O94822**	Zinc finger protein 294 (RING finger protein 160).	Go Component not found
**O43716**	GatC-like protein (Protein 15E1.2).	Go Component not found
**P28335**	[5-hydroxytryptamine 2C receptor (5-HT-2C) (Serotonin receptor 2C) (5-, HT2C) (5-HTR2C) (5HT-1C).]	Go Component not found
**P13994**	Coiled-coil domain-containing protein 130 (9 kDa protein).	Go Component not found
**P63010**	[AP-2 complex subunit beta-1 (Adapter-related protein complex 2 beta-1, subunit) (Beta-adaptin) (Plasma membrane adaptor HA2/AP2 adaptin beta, subunit) (Clathrin assembly protein complex 2 beta large chain), (AP105B).]	Go Component not found
**Q96CW1**	[AP-2 complex subunit mu-1 (Adaptin mu-1) (AP-2 mu-2 chain) (Clathrin, coat assembly protein AP50) (Clathrin coat-associated protein AP50), (Plasma membrane adaptor AP-2 50 kDa protein) (HA2 50 kDa subunit), (Clathrin assembly protein complex 2 medium chain).]	Go Component not found
**P17174**	[Aspartate aminotransferase, cytoplasmic (EC 2.6.1.1) (Transaminase A), (Glutamate oxaloacetate transaminase 1).]	Go Component not found
**P07108**	[Acyl-CoA-binding protein (ACBP) (Diazepam-binding inhibitor) (DBI), (Endozepine) (EP).]	Go Component not found
**O14525**	Astrotactin-1 precursor.	Go Component not found
**Q06278**	Aldehyde oxidase (EC 1.2.3.1).	Go Component not found
**O43572**	[A kinase anchor protein 10, mitochondrial precursor (Protein kinase A-, anchoring protein 10) (PRKA10) (Dual specificity A kinase-anchoring, protein 2) (D-AKAP-2).]	Go Component not found

**O75969**	[A-kinase anchor protein 3 (Protein kinase A-anchoring protein 3), (PRKA3) (A-kinase anchor protein 110 kDa) (AKAP 110) (Sperm oocyte-, binding protein) (Fibrousheathin-1) (Fibrousheathin I) (Fibrous sheath, protein of 95 kDa) (FSP95).]	Go Component not found
**P57078**	[Receptor-interacting serine/threonine-protein kinase 4 (EC 2.7.11.1), (Ankyrin repeat domain protein 3) (PKC-delta-interacting protein, kinase).]	Go Component not found
**P28039**	[Acyloxyacyl hydrolase precursor (EC 3.1.1.77) [Contains: Acyloxyacyl, hydrolase small subunit; Acyloxyacyl hydrolase large subunit].]	Go Component not found
**Q15041**	[ARL-6-interacting protein 1 (ADP-ribosylation-like factor 6-, interacting protein 1) (Aip-1).]	Go Component not found
**O75185**	[Calcium-transporting ATPase type 2C member 2 (EC 3.6.3.8) (ATPase, 2C2).]	Go Component not found
**P17544**	[Cyclic AMP-dependent transcription factor ATF-7 (Activating, transcription factor 7) (Transcription factor ATF-A).]	Go Component not found
**Q13315**	[Serine-protein kinase ATM (EC 2.7.11.1) (Ataxia telangiectasia, mutated) (A-T, mutated).]	Go Component not found
**P17735**	[Tyrosine aminotransferase (EC 2.6.1.5) (L-tyrosine:2-oxoglutarate, aminotransferase) (TAT).]	Go Component not found
**P48634**	Large proline-rich protein BAT2 (HLA-B-associated transcript 2).	Go Component not found
**O75363**	[Breast carcinoma amplified sequence 1 (Novel amplified in breast, cancer 1) (Amplified and overexpressed in breast cancer).]	Go Component not found
**Q14457**	[Beclin-1 (Coiled-coil myosin-like BCL2-interacting protein) (Protein, GT197).]	Go Component not found
**P08236**	Beta-glucuronidase precursor (EC 3.2.1.31) (Beta-G1).	Go Component not found
**Q13075**	[Baculoviral IAP repeat-containing protein 1 (Neuronal apoptosis, inhibitory protein).]	Go Component not found
**P55107**	[Bone morphogenetic protein 3b precursor (BMP-3b), (Growth/differentiation factor 10) (GDF-10) (Bone-inducing protein), (BIP).]	Go Component not found
**P18075**	[Bone morphogenetic protein 7 precursor (BMP-7) (Osteogenic protein 1), (OP-1) (Eptotermin alfa).]	Go Component not found
**Q9H583**	HEAT repeat-containing protein 1 (Protein BAP28).	Go Component not found
**P25440**	Bromodomain-containing protein 2 (Protein RING3) (O27.1.1).	Go Component not found
**Q99622**	Protein C10.	Go Component not found
**Q13740**	CD166 antigen precursor (Activated leukocyte cell adhesion molecule).	Go Component not found
**P29377**	[Protein S100-G (S100 calcium-binding protein G) (Vitamin D-dependent, calcium-binding protein, intestinal) (CABP) (Calbindin-D9k).]	Go Component not found
**P05937**	[Calbindin (Vitamin D-dependent calcium-binding protein, avian-type), (Calbindin D28) (D-28K).]	Go Component not found

**P22223**	Cadherin-3 precursor (Placental-cadherin) (P-cadherin).	Go Component not found
**P55286**	Cadherin-8 precursor.	Go Component not found
**P00915**	[Carbonic anhydrase 1 (EC 4.2.1.1) (Carbonic anhydrase I) (Carbonate, dehydratase I) (CA-I).]	Go Component not found
**P23280**	[Carbonic anhydrase 6 precursor (EC 4.2.1.1) (Carbonic anhydrase VI), (Carbonate dehydratase VI) (CA-VI) (Secreted carbonic anhydrase), (Salivary carbonic anhydrase).]	Go Component not found
**P07384**	[Calpain-1 catalytic subunit (EC 3.4.22.52) (Calpain-1 large subunit), (Calcium-activated neutral proteinase 1) (CANP 1) (Calpain mu-type), (muCANP) (Micromolar-calpain).]	Go Component not found
**P04632**	[Calpain small subunit 1 (CSS1) (Calcium-dependent protease small, subunit 1) (Calcium-dependent protease small subunit) (CDPS) (Calpain, regulatory subunit) (Calcium-activated neutral proteinase small, subunit) (CANP small subunit).]	Go Component not found
**P40123**	Adenylyl cyclase-associated protein 2 (CAP 2).	Go Component not found
**P43234**	Cathepsin O precursor (EC 3.4.22.42).	Go Component not found
**P06307**	[Cholecystokinins precursor (CCK) [Contains: Cholecystokinin 58, (CCK58); Cholecystokinin 39 (CCK39); Cholecystokinin 33 (CCK33);, Cholecystokinin 12 (CCK12); Cholecystokinin 8 (CCK8)].]	Go Component not found
**Q13319**	[Cyclin-dependent kinase 5 activator 2 precursor (CDK5 activator 2), (Cyclin-dependent kinase 5 regulatory subunit 2) (P39) (P39I).]	Go Component not found
**Q15744**	CCAAT/enhancer-binding protein epsilon (C/EBP epsilon).	Go Component not found
**P00751**	[Complement factor B precursor (EC 3.4.21.47) (C3/C5 convertase), (Properdin factor B) (Glycine-rich beta glycoprotein) (GBG) (PBF2), [Contains: Complement factor B Ba fragment; Complement factor B Bb, fragment].]	Go Component not found
**P05156**	[Complement factor I precursor (EC 3.4.21.45) (C3B/C4B inactivator), [Contains: Complement factor I heavy chain; Complement factor I light, chain].]	Go Component not found
**Q13495**	Protein CG1 (F18).	Go Component not found
**Q16589**	Cyclin-G2.	Go Component not found
**O14646**	[Chromodomain-helicase-DNA-binding protein 1 (EC 3.6.1.-) (ATP-, dependent helicase CHD1) (CHD-1).]	Go Component not found
**Q14839**	[Chromodomain-helicase-DNA-binding protein 4 (EC 3.6.1.-) (ATP-, dependent helicase CHD4) (CHD-4) (Mi-2 autoantigen 218 kDa protein), (Mi2-beta).]	Go Component not found
**Q14524**	[Sodium channel protein type 5 subunit alpha (Sodium channel protein, type V subunit alpha) (Voltage-gated sodium channel subunit alpha, Nav1.5) (Sodium channel protein cardiac muscle subunit alpha) (HH1).]	Go Component not found
**Q9Y2U2**	Potassium channel subfamily K member 7.	Go Component not found
**P22676**	Calretinin (CR) (29 kDa calbindin).	Go Component not found
**P49759**	[Dual specificity protein kinase CLK1 (EC 2.7.12.1) (CDC-like kinase, 1).]	Go Component not found

**P10645**	[Chromogranin-A precursor (CgA) (Pituitary secretory protein I) (SP-I), [Contains: Vasostatin-1 (Vasostatin I); Vasostatin-2 (Vasostatin II);, EA-92; ES-43; Pancreastatin; SS-18; WA-8; WE-14; LF-19; AL-11; GV-19;, GR-44; ER-37].]	Go Component not found
**O00408**	[cGMP-dependent 3',5'-cyclic phosphodiesterase (EC 3.1.4.17) (Cyclic, GMP-stimulated phosphodiesterase) (CGS-PDE) (cGSPDE).]	Go Component not found
**Q13370**	[cGMP-inhibited 3',5'-cyclic phosphodiesterase B (EC 3.1.4.17) (Cyclic, GMP-inhibited phosphodiesterase B) (CGI-PDE B) (CGIPDE1) (CGIP1).]	Go Component not found
**O76074**	[cGMP-specific 3',5'-cyclic phosphodiesterase (EC 3.1.4.35) (CGB-PDE), (cGMP-binding cGMP-specific phosphodiesterase).]	Go Component not found
**P01024**	[Complement C3 precursor [Contains: Complement C3 beta chain;, Complement C3 alpha chain; C3a anaphylatoxin; Complement C3b alpha', chain; Complement C3c alpha' chain fragment 1; Complement C3dg, fragment; Complement C3g fragment; Complement C3d fragment; Complement, C3f fragment; Complement C3c alpha' chain fragment 2].]	Go Component not found
**Q9Y281**	Cofilin-2 (Cofilin, muscle isoform).	Go Component not found
**P14406**	[Cytochrome c oxidase polypeptide VIIa-liver/heart, mitochondrial, precursor (EC 1.9.3.1) (Cytochrome c oxidase subunit VIIa-L) (VIIaL).]	Go Component not found
**O75881**	[Cytochrome P450 7B1 (EC 1.14.13.100) (25-hydroxycholesterol 7-alpha-, hydroxylase) (Oxysterol 7-alpha-hydroxylase).]	Go Component not found
**P33260**	Cytochrome P450 2C18 (EC 1.14.14.1) (CYPIIC18) (P450-6B/29C).	Go Component not found
**P05181**	Cytochrome P450 2E1 (EC 1.14.14.1) (CYPIIE1) (P450-J).	Go Component not found
**P05813**	[Beta-crystallin A3 [Contains: Beta-crystallin A3, isoform A1, Delta4, form; Beta-crystallin A3, isoform A1, Delta7 form; Beta-crystallin A3,, isoform A1, Delta8 form].]	Go Component not found
**P22914**	Beta-crystallin S (Gamma-crystallin S) (Gamma-S crystallin).	Go Component not found
**P07320**	Gamma-crystallin D (Gamma-D crystallin) (Gamma-crystallin 4).	Go Component not found
**P52943**	Cysteine-rich protein 2 (CRP2) (Protein ESP1).	Go Component not found
**Q13618**	Cullin-3 (CUL-3).	Go Component not found
**P58304**	Homeobox protein CHX10 (Ceh-10 homeodomain-containing homolog).	Go Component not found
**O43306**	[Adenylate cyclase type 6 (EC 4.6.1.1) (Adenylate cyclase type VI) (ATP, pyrophosphate-lyase 6) (Adenylyl cyclase 6) (Ca(2+)-inhibitable, adenylyl cyclase).]	Go Component not found
**O75909**	Cyclin-K.	Go Component not found
**P62941**	[Peptidyl-prolyl cis-trans isomerase A (EC 5.2.1.8) (PPIase A), (Rotamase A) (Cyclophilin A) (Cyclosporin A-binding protein).]	Go Component not found
**P01034**	[Cystatin-C precursor (Cystatin-3) (Neuroendocrine basic polypeptide), (Gamma-trace) (Post-gamma-globulin).]	Go Component not found

**Q92499**	[ATP-dependent RNA helicase DDX1 (EC 3.6.1.-) (DEAD box protein 1), (DEAD box protein retinoblastoma) (DBP-RB).]	Go Component not found
**Q07507**	Dermatopontin precursor (Tyrosine-rich acidic matrix protein) (TRAMP).	Go Component not found
**O60443**	[Non-syndromic hearing impairment protein 5 (Inversely correlated with, estrogen receptor expression 1) (ICERE-1).]	Go Component not found
**O94788**	[Retinal dehydrogenase 2 (EC 1.2.1.36) (RalDH2) (RALDH 2) (RALDH(II)), (Retinaldehyde-specific dehydrogenase type 2) (Aldehyde dehydrogenase, family 1 member A2).]	Go Component not found
**P49448**	Glutamate dehydrogenase 2, mitochondrial precursor (EC 1.4.1.3) (GDH).	Go Component not found
**Q00796**	Sorbitol dehydrogenase (EC 1.1.1.14) (L-iditol 2-dehydrogenase).	Go Component not found
**Q9UBT3**	[Dickkopf-related protein 4 precursor (Dkk-4) (Dickkopf-4) (hDkk-4), [Contains: Dickkopf-related protein 4 short form].]	Go Component not found
**Q9Y5R6**	[Doublesex- and mab-3-related transcription factor 1 (DM domain, expressed in testis protein 1).]	Go Component not found
**P56282**	[DNA polymerase epsilon subunit 2 (EC 2.7.7.7) (DNA polymerase II, subunit 2) (DNA polymerase epsilon subunit B).]	Go Component not found
**P27487**	[Dipeptidyl peptidase 4 (EC 3.4.14.5) (Dipeptidyl peptidase IV) (DPP, IV) (T-cell activation antigen CD26) (TP103) (Adenosine deaminase, complexing protein 2) (ADABP) [Contains: Dipeptidyl peptidase 4, membrane form (Dipeptidyl peptidase IV membrane form); Dipeptidyl, peptidase 4 soluble form (Dipeptidyl peptidase IV soluble form)].]	Go Component not found
**P56555**	[Down syndrome critical region protein 4 (Down syndrome critical region, protein B).]	Go Component not found
**P28324**	[ETS domain-containing protein Elk-4 (Serum response factor accessory, protein 1) (SAP-1).]	Go Component not found
**P09104**	[Gamma-enolase (EC 4.2.1.11) (2-phospho-D-glycerate hydro-lyase), (Neural enolase) (Neuron-specific enolase) (NSE) (Enolase 2).]	Go Component not found
**P61550**	[HERV-T_19q13.11 provirus ancestral Env polyprotein precursor (Envelope, polyprotein) (HERV-T Env protein) [Includes: Surface protein (SU);, Transmembrane protein (TM)].]	Go Component not found
**O15197**	[Ephrin type-B receptor 6 precursor (Tyrosine-protein kinase-defective, receptor EPH-6) (HEP).]	Go Component not found
**O95718**	[Steroid hormone receptor ERR2 (Estrogen-related receptor, beta) (ERR-, beta) (Estrogen receptor-like 2) (ERR beta-2).]	Go Component not found
**P22794**	[EVI2A protein precursor (Ecotropic viral integration site 2A protein, homolog) (EVI-2A).]	Go Component not found
**Q16875**	[6-phosphofructo-2-kinase/fructose-2,6-biphosphatase 3 (6PF-2-K/Fru-, 2,6-P2ASE brain/placenta-type isozyme) (iPFK-2) (Renal carcinoma, antigen NY-REN-56) [Includes: 6-phosphofructo-2-kinase (EC 2.7.1.105);, Fructose-2,6-bisphosphatase (EC 3.1.3.46)].]	Go Component not found
**P49913**	[Cathelicidin antimicrobial peptide precursor (18 kDa cationic, antimicrobial protein) (CAP-18) (hCAP-18) [Contains: Antibacterial, protein FALL-39 (FALL-39 peptide antibiotic); Antibacterial protein, LL-37].]	Go Component not found
**Q9UKT8**	[F-box/WD repeat-containing protein 2 (F-box and WD-40 domain-, containing protein 2) (Protein MD6).]	Go Component not found

P02771	[Alpha-fetoprotein precursor (Alpha-fetoglobulin) (Alpha-1-, fetoprotein).]	Go Component not found
Q9P0K8	Forkhead box protein J2 (Fork head homologous X).	Go Component not found
P06744	[Glucose-6-phosphate isomerase (EC 5.3.1.9) (GPI) (Phosphoglucose, isomerase) (PGI) (Phosphohexose isomerase) (PHI) (Neuroleukin) (NLK), (Sperm antigen 36) (SA-36).]	Go Component not found
Q03113	Guanine nucleotide-binding protein alpha-12 subunit (G alpha-12).	Go Component not found
P32455	[Interferon-induced guanylate-binding protein 1 (GTP-binding protein 1), (Guanine nucleotide-binding protein 1) (GBP-1) (HuGBP-1).]	Go Component not found
P52951	[Homeobox protein GBX-2 (Gastrulation and brain-specific homeobox, protein 2).]	Go Component not found
P43080	[Guanylyl cyclase-activating protein 1 (GCAP 1) (Guanylate cyclase, activator 1A).]	Go Component not found
P36915	Guanine nucleotide-binding protein-like 1 (GTP-binding protein HSR1).	Go Component not found
P43304	[Glycerol-3-phosphate dehydrogenase, mitochondrial precursor, (EC 1.1.99.5) (GPD-M) (GPDH-M) (mtGPD).]	Go Component not found
Q14449	Growth factor receptor-bound protein 14 (GRB14 adapter protein).	Go Component not found
O14653	[Golgi SNAP receptor complex member 2 (27 kDa Golgi SNARE protein), (Membrin).]	Go Component not found
P07203	[Glutathione peroxidase 1 (EC 1.11.1.9) (GSHPx-1) (GPx-1) (Cellular, glutathione peroxidase).]	Go Component not found
P11169	[Solute carrier family 2, facilitated glucose transporter member 3, (Glucose transporter type 3, brain) (GLUT-3).]	Go Component not found
P49915	[GMP synthase [glutamine-hydrolyzing] (EC 6.3.5.2) (Glutamine, amidotransferase) (GMP synthetase).]	Go Component not found
P98176	Histone H2A-Bbd (H2A Barr body-deficient) (H2A.Bbd).	Go Component not found
P01916	HLA class II histocompatibility antigen, SB beta chain (Fragment).	Go Component not found
P69892	[Hemoglobin subunit gamma-2 (Hemoglobin gamma-2 chain) (Gamma-2-globin), (Hemoglobin gamma-G chain) (Hb F Ggamma).]	Go Component not found
P26927	[Hepatocyte growth factor-like protein precursor (Macrophage, stimulatory protein) (MSP) (Macrophage-stimulating protein) [Contains:, Hepatocyte growth factor-like protein alpha chain; Hepatocyte growth, factor-like protein beta chain].]	Go Component not found
P17693	[HLA class I histocompatibility antigen, alpha chain G precursor (HLA G, antigen).]	Go Component not found
Q03014	[Homeobox protein PRH (Hematopoietically-expressed homeobox) (Homeobox, protein HEX).]	Go Component not found
Q13442	[28 kDa heat- and acid-stable phosphoprotein (PDGF-associated protein), (PAP) (PDGFA-associated protein 1) (PAP1).]	Go Component not found

P83110	[Probable serine protease HTRA3 precursor (EC 3.4.21.-) (High-, temperature requirement factor A3) (Pregnancy-related serine, protease).]	Go Component not found
P17066	Heat shock 70 kDa protein 6 (Heat shock 70 kDa protein B').	Go Component not found
O14558	[Heat shock protein beta-6 (HspB6) (Heat shock 20 kDa-like protein, p20).]	Go Component not found
P57058	[Hormonally up-regulated neu tumor-associated kinase (EC 2.7.11.1), (Serine/threonine-protein kinase MAK-V) (B19).]	Go Component not found
P31267	Homeobox protein Hox-A6 (Hox-1B).	Go Component not found
P14651	Homeobox protein Hox-B3 (Hox-2G) (Hox-2.7).	Go Component not found
Q9NYD6	Homeobox protein Hox-C10.	Go Component not found
P22692	[Insulin-like growth factor-binding protein 4 precursor (IGFBP-4) (IBP-, 4) (IGF-binding protein 4).]	Go Component not found
P20810	Calpastatin (Calpain inhibitor) (Sperm BS-17 component).	Go Component not found
P27352	Gastric intrinsic factor precursor (Intrinsic factor) (IF) (INF).	Go Component not found
Q12894	Interferon-related developmental regulator 2 (SKMC15 protein).	Go Component not found
P05019	[Insulin-like growth factor IB precursor (IGF-IB) (Somatomedin C), (Mechano growth factor) (MGF).]	Go Component not found
P58166	Inhibin beta E chain precursor (Activin beta-E chain).	Go Component not found
P16871	[Interleukin-7 receptor alpha chain precursor (IL-7R-alpha) (CD127, antigen) (CDw127).]	Go Component not found
O15225	Putative inactivation escape 1 protein.	Go Component not found
Q9C010	cAMP-dependent protein kinase inhibitor beta (PKI-beta).	Go Component not found
P41236	Protein phosphatase inhibitor 2 (IPP-2).	Go Component not found
Q14500	[ATP-sensitive inward rectifier potassium channel 12 (Potassium, channel, inwardly rectifying subfamily J member 12) (Inward rectifier, K(+) channel Kir2.2) (Kir2.2v) (IRK2).]	Go Component not found
Q9BZI1	[Iroquois-class homeodomain protein IRX-2 (Iroquois homeobox protein 2), (Homeodomain protein IRXA2).]	Go Component not found
P13612	[Integrin alpha-4 precursor (Integrin alpha-IV) (VLA-4) (CD49d, antigen).]	Go Component not found
O60674	Tyrosine-protein kinase JAK2 (EC 2.7.10.2) (Janus kinase 2) (JAK-2).	Go Component not found

Q14525	Keratin, type I cuticular Ha3-II (Hair keratin, type I Ha3-II).	Go Component not found
Q9UBS0	[Ribosomal protein S6 kinase beta-2 (EC 2.7.11.1) (S6K2) (70 kDa, ribosomal protein S6 kinase 2) (p70-S6KB) (p70 ribosomal S6 kinase, beta) (p70 S6Kbeta) (p70 S6 kinase beta) (S6K-beta) (p70-beta) (S6, kinase-related kinase) (SRK) (Serine/threonine-protein kinase 14, beta).]	Go Component not found
P29622	[Kallistatin precursor (Serpin A4) (Kallikrein inhibitor) (Protease, inhibitor 4).]	Go Component not found
P31323	cAMP-dependent protein kinase type II-beta regulatory subunit.	Go Component not found
Q14012	[Calcium/calmodulin-dependent protein kinase type 1 (EC 2.7.11.17) (CaM, kinase I) (CaM-KI) (CaM kinase I alpha) (CaMKI-alpha).]	Go Component not found
Q9UGI6	[Small conductance calcium-activated potassium channel protein 3 (SK3), (SKCa3).]	Go Component not found
Q9Y6T7	[Diacylglycerol kinase beta (EC 2.7.1.107) (Diglyceride kinase beta), (DGK-beta) (DAG kinase beta) (90 kDa diacylglycerol kinase).]	Go Component not found
P46013	Antigen KI-67.	Go Component not found
Q93100	[Phosphorylase b kinase regulatory subunit beta (Phosphorylase kinase, subunit beta).]	Go Component not found
O60662	[Kelch repeat and BTB domain-containing protein 10 (Kelch-related, protein 1) (Kel-like protein 23) (Sarcosin).]	Go Component not found
P52954	Transcription factor LBX1 (Ladybird homeobox protein homolog 1).	Go Component not found
O15270	[Serine palmitoyltransferase 2 (EC 2.3.1.50) (Long chain base, biosynthesis protein 2) (LCB 2) (Serine-palmitoyl-CoA transferase 2), (SPT 2).]	Go Component not found
Q9ULC5	[Long-chain-fatty-acid--CoA ligase 5 (EC 6.2.1.3) (Long-chain acyl-CoA, synthetase 5) (LACS 5).]	Go Component not found
O43900	LIM domain only protein 6 (Triple LIM domain protein 6).	Go Component not found
P16050	[Arachidonate 15-lipoxygenase (EC 1.13.11.33) (Arachidonate omega-6, lipoxygenase) (15-LOX).]	Go Component not found
Q16706	[Alpha-mannosidase 2 (EC 3.2.1.114) (Alpha-mannosidase II) (Mannosyl-, oligosaccharide 1,3-1,6-alpha-mannosidase) (MAN II) (Golgi alpha-, mannosidase II) (Mannosidase alpha class 2A member 1).]	Go Component not found
Q9Y2E5	[Epididymis-specific alpha-mannosidase precursor (EC 3.2.1.24), (Mannosidase alpha class 2B member 2).]	Go Component not found
Q9Y6R4	[Mitogen-activated protein kinase kinase kinase 4 (EC 2.7.11.25), (MAPK/ERK kinase kinase 4) (MEK kinase 4) (MEKK 4) (MAP three kinase, 1).]	Go Component not found
P80192	[Mitogen-activated protein kinase kinase kinase 9 (EC 2.7.11.25) (Mixed, lineage kinase 1).]	Go Component not found
P20916	Myelin-associated glycoprotein precursor (Siglec-4a).	Go Component not found

**P43365**	Melanoma-associated antigen 12 (MAGE-12 antigen) (MAGE12F).	Go Component not found
**P34949**	[Mannose-6-phosphate isomerase (EC 5.3.1.8) (Phosphomannose isomerase), (PMI) (Phosphohexomutase).]	Go Component not found
**P23368**	[NAD-dependent malic enzyme, mitochondrial precursor (EC 1.1.1.38), (NAD-ME) (Malic enzyme 2).]	Go Component not found
**P36952**	Serpin B5 precursor (Maspin) (Protease inhibitor 5).	Go Component not found
**P02686**	[Myelin basic protein (MBP) (Myelin A1 protein) (Myelin membrane, encephalitogenic protein).]	Go Component not found
**Q06413**	Myocyte-specific enhancer factor 2C.	Go Component not found
**P52888**	Thimet oligopeptidase (EC 3.4.24.15) (Endopeptidase 24.15) (MP78).	Go Component not found
**P43366**	[Melanoma-associated antigen B1 (MAGE-B1 antigen) (MAGE-XP antigen), (DSS-AHC critical interval MAGE superfamily 10) (DAM10).]	Go Component not found
**P60602**	Protein MGR2 homolog.	Go Component not found
**P27361**	[Mitogen-activated protein kinase 3 (EC 2.7.11.24) (Extracellular, signal-regulated kinase 1) (ERK-1) (Insulin-stimulated MAP2 kinase), (MAP kinase 1) (MAPK 1) (p44-ERK1) (ERT2) (p44-MAPK) (Microtubule-, associated protein 2 kinase).]	Go Component not found
**O15234**	[Protein CASC3 (Cancer susceptibility candidate gene 3 protein), (Metastatic lymph node protein 51) (Protein MLN 51) (Protein barentsz), (Btz).]	Go Component not found
**Q10571**	Probable tumor suppressor protein MN1.	Go Component not found
**P50222**	[Homeobox protein MOX-2 (Mesenchyme homeobox 2) (Growth arrest-specific, homeobox).]	Go Component not found
**P47224**	Guanine nucleotide exchange factor MSS4 (Rab-interacting factor).	Go Component not found
**Q13496**	Myotubularin (EC 3.1.3.48).	Go Component not found
**Q9Y2Z2**	Protein MTO1 homolog, mitochondrial precursor.	Go Component not found
**O43795**	Myosin-Ib (Myosin I alpha) (MMI-alpha) (MMIa) (MYH-1c).	Go Component not found
**Q9NQX4**	Myosin-Vc.	Go Component not found
**P57103**	[Sodium/calcium exchanger 3 precursor (Na(+)/Ca(2+)-exchange protein, 3).]	Go Component not found
**O75376**	Nuclear receptor corepressor 1 (N-CoR1) (N-CoR).	Go Component not found
**Q92597**	[Protein NDRG1 (N-myc downstream-regulated gene 1 protein), (Differentiation-related gene 1 protein) (DRG-1) (Reducing agents and, tunicamycin-responsive protein) (RTP) (Nickel-specific induction, protein Cap43) (Rit42).]	Go Component not found

**Q99574**	Neuroserpin precursor (Serpin I1) (Protease inhibitor 12).	Go Component not found
**Q15818**	Neuronal pentraxin-1 precursor (NP1) (Neuronal pentraxin I) (NP-I).	Go Component not found
**Q16656**	[Nuclear respiratory factor 1 (NRF-1) (Alpha palindromic-binding, protein) (Alpha-pal).]	Go Component not found
**Q9Y5Y2**	Nucleotide-binding protein 2 (NBP 2).	Go Component not found
**O43869**	[Olfactory receptor 2T1 (Olfactory receptor OR1-61) (Olfactory receptor, 1-25) (OR1-25).]	Go Component not found
**Q9GZM6**	Olfactory receptor 8D2 (Olfactory receptor-like protein JCG2).	Go Component not found
**P56715**	[Oxygen-regulated protein 1 (Retinitis pigmentosa RP1 protein), (Retinitis pigmentosa 1 protein).]	Go Component not found
**P42336**	[Phosphatidylinositol-4,5-bisphosphate 3-kinase catalytic subunit alpha, isoform (EC 2.7.1.153) (PI3-kinase p110 subunit alpha) (PtdIns-3-, kinase p110) (PI3K).]	Go Component not found
**O15350**	[Tumor protein p73 (p53-like transcription factor) (p53-related, protein).]	Go Component not found
**P05120**	[Plasminogen activator inhibitor 2 precursor (PAI-2) (Placental, plasminogen activator inhibitor) (Monocyte Arg-serpin) (Urokinase, inhibitor).]	Go Component not found
**P23760**	Paired box protein Pax-3 (HUP2).	Go Component not found
**P57723**	Poly(rC)-binding protein 4 (Alpha-CP4).	Go Component not found
**Q9Y6G1**	Transmembrane protein 14A.	Go Component not found
**Q9ULW8**	[Protein-arginine deiminase type-3 (EC 3.5.3.15) (Protein-arginine, deiminase type III) (Peptidylarginine deiminase III).]	Go Component not found
**P30048**	[Thioredoxin-dependent peroxide reductase, mitochondrial precursor, (EC 1.11.1.15) (Peroxiredoxin-3) (PRX III) (Antioxidant protein 1), (AOP-1) (Protein MER5 homolog) (HBC189).]	Go Component not found
**P30086**	[Phosphatidylethanolamine-binding protein 1 (PEBP-1) (Prostatic-binding, protein) (HCNPpp) (Neuropolypeptide h3) (Raf kinase inhibitor protein), (RKIP) [Contains: Hippocampal cholinergic neurostimulating peptide, (HCNP)].]	Go Component not found
**P01210**	[Proenkephalin A precursor [Contains: Synenkephalin; Met-enkephalin, (Opioid growth factor) (OGF); Met-enkephalin-Arg-Gly-Leu; Leu-, enkephalin; Met-enkephalin-Arg-Phe].]	Go Component not found
**P28328**	[Peroxisome assembly factor 1 (PAF-1) (Peroxin-2) (Peroxisomal membrane, protein 3) (35 kDa peroxisomal membrane protein) (RING finger protein, 72).]	Go Component not found
**P80108**	[Phosphatidylinositol-glycan-specific phospholipase D precursor, (EC 3.1.4.50) (PI-G PLD) (Glycoprotein phospholipase D) (Glycosyl-, phosphatidylinositol-specific phospholipase D).]	Go Component not found
**Q15147**	[1-phosphatidylinositol-4,5-bisphosphate phosphodiesterase beta 4, (EC 3.1.4.11) (Phosphoinositide phospholipase C) (Phospholipase C-, beta-4) (PLC-beta-4).]	Go Component not found
**P16885**	[1-phosphatidylinositol-4,5-bisphosphate phosphodiesterase gamma 2, (EC 3.1.4.11) (Phosphoinositide phospholipase C) (PLC-gamma-2), (Phospholipase C-gamma-2) (PLC-IV).]	Go Component not found

Q14155	[Rho guanine nucleotide exchange factor 7 (PAK-interacting exchange, factor beta) (Beta-Pix) (COOL-1) (p85).]	Go Component not found
P51817	[Serine/threonine-protein kinase PRKX (EC 2.7.11.1) (Protein kinase, PKX1).]	Go Component not found
Q14651	Plastin-1 (I-plastin) (Intestine-specific plastin).	Go Component not found
P11086	[Phenylethanolamine N-methyltransferase (EC 2.1.1.28) (PNMTase), (Noradrenaline N-methyltransferase).]	Go Component not found
P35558	[Phosphoenolpyruvate carboxykinase, cytosolic [GTP] (EC 4.1.1.32), (Phosphoenolpyruvate carboxylase) (PEPCK-C).]	Go Component not found
Q00169	[Phosphatidylinositol transfer protein alpha isoform (PtdIns transfer, protein alpha) (PtdInsTP) (PI-TP-alpha).]	Go Component not found
Q9UKN5	PR domain zinc finger protein 4 (PR domain-containing protein 4).	Go Component not found
P78527	[DNA-dependent protein kinase catalytic subunit (EC 2.7.11.1) (DNA-PK, catalytic subunit) (DNA-PKcs) (DNPK1) (p460).]	Go Component not found
P24158	[Myeloblastin precursor (EC 3.4.21.76) (Leukocyte proteinase 3) (PR-3), (PR3) (AGP7) (Wegener autoantigen) (P29) (C-ANCA antigen) (Neutrophil, proteinase 4) (NP-4).]	Go Component not found
P81277	[Prolactin-releasing peptide precursor (PrRP) (Prolactin-releasing, hormone) [Contains: Prolactin-releasing peptide PrRP31; Prolactin-, releasing peptide PrRP20].]	Go Component not found
Q13882	[Tyrosine-protein kinase 6 (EC 2.7.10.2) (Breast tumor kinase), (Tyrosine-protein kinase BRK).]	Go Component not found
Q06124	[Tyrosine-protein phosphatase non-receptor type 11 (EC 3.1.3.48), (Protein-tyrosine phosphatase 2C) (PTP-2C) (PTP-1D) (SH-PTP3) (SH-, PTP2) (SHP-2) (Shp2).]	Go Component not found
Q12923	[Tyrosine-protein phosphatase non-receptor type 13 (EC 3.1.3.48), (Protein-tyrosine phosphatase 1E) (PTP-E1) (hPTPE1) (PTP-BAS), (Protein-tyrosine phosphatase PTPL1) (Fas-associated protein-tyrosine, phosphatase 1) (FAP-1).]	Go Component not found
Q06203	[Amidophosphoribosyltransferase precursor (EC 2.4.2.14) (Glutamine, phosphoribosylpyrophosphate amidotransferase) (ATase) (GPAT).]	Go Component not found
P11498	[Pyruvate carboxylase, mitochondrial precursor (EC 6.4.1.1) (Pyruvic, carboxylase) (PCB).]	Go Component not found
P17812	[CTP synthase 1 (EC 6.3.4.2) (UTP--ammonia ligase 1) (CTP synthetase, 1).]	Go Component not found
Q16769	[Glutaminyl-peptide cyclotransferase precursor (EC 2.3.2.5) (QC), (Glutaminyl-tRNA cyclotransferase) (Glutaminyl cyclase) (Glutamyl, cyclase) (EC).]	Go Component not found
Q9Y5P3	Retinoic acid-induced protein 2.	Go Component not found
P51157	Ras-related protein Rab-28 (Rab-26).	Go Component not found
P20338	Ras-related protein Rab-4A.	Go Component not found
Q08999	[Retinoblastoma-like protein 2 (130 kDa retinoblastoma-associated, protein) (PRB2) (P130) (RBR-2).]	Go Component not found

**P35243**	Recoverin (Cancer-associated retinopathy protein) (Protein CAR).	Go Component not found
**Q01201**	Transcription factor RelB (I-Rel).	Go Component not found
**P48378**	DNA-binding protein RFX2.	Go Component not found
**P47736**	Rap1 GTPase-activating protein 1 (Rap1GAP).	Go Component not found
**P41220**	[Regulator of G-protein signaling 2 (RGS2) (G0/G1 switch regulatory, protein 8).]	Go Component not found
**Q9UGC6**	Regulator of G-protein signaling 17 (RGS17).	Go Component not found
**P07998**	[Ribonuclease pancreatic precursor (EC 3.1.27.5) (RNase 1) (RNase A), (RNase UpI-1) (RIB-1) (HP-RNase).]	Go Component not found
**Q9H9Y6**	[DNA-directed RNA polymerase I subunit RPA2 (EC 2.7.7.6) (RNA, polymerase I subunit 2) (DNA-directed RNA polymerase I 135 kDa, polypeptide) (RPA135).]	Go Component not found
**Q9UI43**	[Putative ribosomal RNA methyltransferase 2 (EC 2.1.1.-) (rRNA, (uridine-2'-O-)-methyltransferase).]	Go Component not found
**O95294**	RasGAP-activating-like protein 1.	Go Component not found
**P48443**	Retinoic acid receptor RXR-gamma (Retinoid X receptor gamma).	Go Component not found
**Q9NZJ4**	Sacsin.	Go Component not found
**Q15431**	Synaptonemal complex protein 1 (SCP-1).	Go Component not found
**O43175**	D-3-phosphoglycerate dehydrogenase (EC 1.1.1.95) (3-PGDH).	Go Component not found
**Q13242**	[Splicing factor, arginine/serine-rich 9 (Pre-mRNA-splicing factor, SRp30C).]	Go Component not found
**Q99962**	[SH3-containing GRB2-like protein 2 (EC 2.3.1.-) (Endophilin-1), (Endophilin-A1) (SH3 domain protein 2A) (EEN-B1).]	Go Component not found
**Q15466**	[Nuclear receptor 0B2 (Orphan nuclear receptor SHP) (Small heterodimer, partner).]	Go Component not found
**Q9BPZ7**	[Stress-activated map kinase-interacting protein 1 (SAPK-interacting, protein 1) (Putative Ras inhibitor JC310).]	Go Component not found
**O95343**	Homeobox protein SIX3 (Sine oculis homeobox homolog 3).	Go Component not found
**Q92854**	[Semaphorin-4D precursor (Leukocyte activation antigen CD100) (BB18), (A8) (GR3).]	Go Component not found
**Q9H3T2**	Semaphorin-6C precursor (Semaphorin Y) (Sema Y).	Go Component not found

**O15105**	[Mothers against decapentaplegic homolog 7 (SMAD 7) (Mothers against, DPP homolog 7) (Smad7) (hSMAD7).]	Go Component not found
**P23327**	[Sarcoplasmic reticulum histidine-rich calcium-binding protein, precursor.]	Go Component not found
**O75844**	[CAAX prenyl protease 1 homolog (EC 3.4.24.84) (Prenyl protein-specific, endoprotease 1) (Farnesylated proteins-converting enzyme 1) (FACE-1), (Zinc metalloproteinase Ste24 homolog).]	Go Component not found
**P51692**	Signal transducer and activator of transcription 5B.	Go Component not found
**Q06520**	[Bile salt sulfotransferase (EC 2.8.2.14) (Hydroxysteroid, Sulfotransferase) (HST) (Dehydroepiandrosterone sulfotransferase), (DHEA-ST) (ST2) (ST2A3).]	Go Component not found
**O15467**	[Small-inducible cytokine A16 precursor (CCL16) (IL-10-inducible, chemokine) (Chemokine LEC) (Liver-expressed chemokine) (Monotactin-1), (MTN-1) (Chemokine CC-4) (HCC-4) (NCC-4) (Lymphocyte and monocyte, chemoattractant) (LMC) (LCC-1).]	Go Component not found
**O15056**	[Synaptojanin-2 (EC 3.1.3.36) (Synaptic inositol-1,4,5-trisphosphate 5-, phosphatase 2).]	Go Component not found
**P02778**	[Small-inducible cytokine B10 precursor (CXCL10) (10 kDa interferon-, gamma-induced protein) (Gamma-IP10) (IP-10) [Contains: CXCL10(1-73)].]	Go Component not found
**P48775**	[Tryptophan 2,3-dioxygenase (EC 1.13.11.11) (TO) (Tryptophan pyrrolase), (Tryptophanase) (Tryptophan oxygenase) (Tryptamin 2,3-dioxygenase), (TRPO).]	Go Component not found
**Q92917**	[G patch domain and KOW motifs-containing protein (G patch domain-, containing protein 5) (Protein MOS2 homolog) (Protein T54).]	Go Component not found
**Q01995**	[Transgelin (Smooth muscle protein 22-alpha) (SM22-alpha) (WS3-10) (22, kDa actin-binding protein).]	Go Component not found
**Q16559**	T-cell acute lymphocytic leukemia protein 2 (TAL-2).	Go Component not found
**P68366**	[Tubulin alpha-4A chain (Tubulin alpha-1 chain) (Alpha-tubulin 1), (Testis-specific alpha-tubulin) (Tubulin H2-alpha).]	Go Component not found
**Q16650**	T-brain-1 protein (T-box brain protein 1) (TBR-1) (TES-56).	Go Component not found
**O95988**	[T-cell leukemia/lymphoma protein 1B (Oncogene TCL1B), (Syncytiotrophoblast-specific protein) (SYN-1).]	Go Component not found
**P24821**	[Tenascin precursor (TN) (Tenascin-C) (TN-C) (Hexabrachion), (Cytotactin) (Neuronectin) (GMEM) (JI) (Myotendinous antigen) (Glioma-, associated-extracellular matrix antigen) (GP 150-225).]	Go Component not found
**P10646**	[Tissue factor pathway inhibitor precursor (TFPI) (Lipoprotein-, associated coagulation inhibitor) (LACI) (Extrinsic pathway inhibitor), (EPI).]	Go Component not found
**P10827**	[Thyroid hormone receptor alpha (C-erbA-alpha) (c-erbA-1) (EAR-7), (EAR7).]	Go Component not found
**P10599**	[Thioredoxin (Trx) (ATL-derived factor) (ADF) (Surface-associated, sulphydryl protein) (SASP).]	Go Component not found
**Q13009**	[T-lymphoma invasion and metastasis-inducing protein 1 (TIAM-1, protein).]	Go Component not found
**P16035**	[Metalloproteinase inhibitor 2 precursor (TIMP-2) (Tissue inhibitor of, metalloproteinases 2) (CSC-21K).]	Go Component not found

**Q06643**	[Lymphotoxin-beta (LT-beta) (Tumor necrosis factor C) (TNF-C) (Tumor, necrosis factor ligand superfamily member 3).]	Go Component not found
**P60174**	[Triosephosphate isomerase (EC 5.3.1.1) (TIM) (Triose-phosphate, isomerase).]	Go Component not found
**P02788**	[Lactotransferrin precursor (EC 3.4.21.-) (Lactoferrin), (Talalactoferrin alfa) [Contains: Kaliocin-1; Lactoferroxin A;, Lactoferroxin B; Lactoferroxin C].]	Go Component not found
**P13805**	[Troponin T, slow skeletal muscle (TnTs) (Slow skeletal muscle troponin, T) (sTnT).]	Go Component not found
**Q16881**	[Thioredoxin reductase 1, cytoplasmic precursor (EC 1.8.1.9) (TR), (TR1).]	Go Component not found
**P98066**	[Tumor necrosis factor-inducible protein TSG-6 precursor (TNF-, stimulated gene 6 protein) (Tumor necrosis factor, alpha-induced, protein 6) (Hyaluronate-binding protein).]	Go Component not found
**Q9BXU0**	Testis-expressed sequence 12 protein.	Go Component not found
**P07101**	Tyrosine 3-monooxygenase (EC 1.14.16.2) (Tyrosine 3-hydroxylase) (TH).	Go Component not found
**P22314**	Ubiquitin-activating enzyme E1 (A1S9 protein).	Go Component not found
**P40818**	[Ubiquitin carboxyl-terminal hydrolase 8 (EC 3.1.2.15) (Ubiquitin, thioesterase 8) (Ubiquitin-specific-processing protease 8), (Deubiquitinating enzyme 8) (hUBPy).]	Go Component not found
**Q9UPU5**	[Ubiquitin carboxyl-terminal hydrolase 24 (EC 3.1.2.15) (Ubiquitin, thioesterase 24) (Ubiquitin-specific-processing protease 24), (Deubiquitinating enzyme 24).]	Go Component not found
**P54855**	[UDP-glucuronosyltransferase 2B15 precursor (EC 2.4.1.17) (UDPGT), (UDPGTh-3) (HLUG4).]	Go Component not found
**Q15853**	[Upstream stimulatory factor 2 (Upstream transcription factor 2) (FOS-, interacting protein) (FIP) (Major late transcription factor 2).]	Go Component not found
**O14607**	[Ubiquitously transcribed Y chromosome tetratricopeptide repeat protein, (Ubiquitously transcribed TPR protein on the Y chromosome).]	Go Component not found
**Q93034**	[Cullin-5 (CUL-5) (Vasopressin-activated calcium-mobilizing receptor), (VACM-1).]	Go Component not found
**O95498**	[Vascular non-inflammatory molecule 2 precursor (Vanin-2), (Glycosylphosphatidyl inositol-anchored protein GPI-80) (Protein FOAP-, 4).]	Go Component not found
**Q9Y4E6**	[WD repeat-containing protein 7 (TGF-beta resistance-associated protein, TRAG) (Rabconnectin-3 beta).]	Go Component not found
**P04628**	Proto-oncogene protein Wnt-1 precursor.	Go Component not found
**Q15884**	Uncharacterized protein C9orf61 (Protein X123).	Go Component not found
**Q02040**	[Splicing factor, arginine/serine-rich 17A (Protein XE7) (B-lymphocyte, antigen) (721P).]	Go Component not found
**P28715**	[DNA-repair protein complementing XP-G cells (Xeroderma pigmentosum, group G-complementing protein) (DNA excision repair protein ERCC-5).]	Go Component not found

P42331	Rho GTPase-activating protein 25.	Go Component not found
Q14699	[Raftlin (Raft-linking protein) (Cell migration-inducing gene 2, protein).]	Go Component not found
Q14165	Uncharacterized protein KIAA0152 precursor.	Go Component not found
Q14689	Disco-interacting protein 2 homolog A.	Go Component not found
Q92599	Septin-8.	Go Component not found
Q92628	Uncharacterized protein KIAA0232.	Go Component not found
O15016	Tripartite motif-containing protein 66.	Go Component not found
O15084	Ankyrin repeat domain-containing protein 28.	Go Component not found
Q9NUA8	Zinc finger and BTB domain-containing protein 40.	Go Component not found
O94876	Transmembrane and coiled-coil domains protein 1.	Go Component not found
O94967	WD repeat-containing protein 47.	Go Component not found
O60810	PRAME family member 4.	Go Component not found
Q9ULJ7	Ankyrin repeat domain-containing protein 50.	Go Component not found
P07947	[Proto-oncogene tyrosine-protein kinase Yes (EC 2.7.10.2) (p61-Yes) (c-, Yes).]	Go Component not found
O15231	Zinc finger protein 185 (LIM domain protein ZNF185) (P1-A).	Go Component not found
Q9UJU3	Zinc finger protein 228.	Go Component not found
Q14202	Zinc finger MYM-type protein 3 (Zinc finger protein 261).	Go Component not found
Q14586	Zinc finger protein 267 (Zinc finger protein HZF2).	Go Component not found
P17097	[Zinc finger protein 7 (Zinc finger protein KOX4) (Zinc finger protein, HF.16).]	Go Component not found
P16190	[HLA class I histocompatibility antigen, A-33 alpha chain precursor, (MHC class I antigen A*33) (Aw-33) (Aw-19).]	Go Component not found
Q10567	[AP-1 complex subunit beta-1 (Adapter-related protein complex 1 beta-1, subunit) (Beta-adaptin 1) (Adaptor protein complex AP-1 beta-1, subunit) (Golgi adaptor HA1/AP1 adaptin beta subunit) (Clathrin, assembly protein complex 1 beta large chain).]	Go Component not found

P98198	[Probable phospholipid-transporting ATPase ID (EC 3.6.3.1) (ATPase, class I type 8B member 2).]	Go Component not found
P42684	[Tyrosine-protein kinase ABL2 (EC 2.7.10.2) (Abelson murine leukemia, viral oncogene homolog 2) (Abelson-related gene protein) (Tyrosine, kinase ARG).]	Go Component not found
O43506	[ADAM 20 precursor (EC 3.4.24.-) (A disintegrin and metalloproteinase, domain 20).]	Go Component not found
P55197	Protein AF-10.	Go Component not found
Q9Y4K1	Absent in melanoma 1 protein.	Go Component not found
Q9UKA4	[A-kinase anchor protein 11 (Protein kinase A-anchoring protein 11), (PRKA11) (A kinase anchor protein 220 kDa) (AKAP 220) (hAKAP220).]	Go Component not found
P09972	Fructose-bisphosphate aldolase C (EC 4.1.2.13) (Brain-type aldolase).	Go Component not found
Q01432	[AMP deaminase 3 (EC 3.5.4.6) (AMP deaminase isoform E) (Erythrocyte, AMP deaminase).]	Go Component not found
O75106	[Retina-specific copper amine oxidase precursor (EC 1.4.3.6) (RAO), (Amine oxidase [copper-containing]).]	Go Component not found
Q99767	[Amyloid beta A4 precursor protein-binding family A member 2 (Neuron-, specific X11L protein) (Neuronal Munc18-1-interacting protein 2), (Mint-2) (Adapter protein X11beta).]	Go Component not found
P35626	[Beta-adrenergic receptor kinase 2 (EC 2.7.11.15) (Beta-ARK-2) (G-, protein-coupled receptor kinase 3).]	Go Component not found
P17405	[Sphingomyelin phosphodiesterase precursor (EC 3.1.4.12) (Acid, sphingomyelinase) (aSMase).]	Go Component not found
Q99766	[ATP synthase subunit s, mitochondrial precursor (ATP synthase-coupling, factor B) (Mitochondrial ATP synthase regulatory component factor B).]	Go Component not found
Q9UHI8	[ADAMTS-1 precursor (EC 3.4.24.-) (A disintegrin and metalloproteinase, with thrombospondin motifs 1) (ADAM-TS 1) (ADAM-TS1) (METH-1).]	Go Component not found
Q9UKP4	[ADAMTS-7 precursor (EC 3.4.24.-) (A disintegrin and metalloproteinase, with thrombospondin motifs 7) (ADAM-TS 7) (ADAM-TS7).]	Go Component not found
Q9Y679	Ancient ubiquitous protein 1 precursor.	Go Component not found
P46379	[Large proline-rich protein BAT3 (HLA-B-associated transcript 3), (Protein G3).]	Go Component not found
Q9Y6D6	[Brefeldin A-inhibited guanine nucleotide-exchange protein 1 (Brefeldin, A-inhibited GEP 1) (p200 ARF-GEP1) (p200 ARF guanine nucleotide, exchange factor).]	Go Component not found
Q9NR09	[Baculoviral IAP repeat-containing protein 6 (Ubiquitin-conjugating BIR, domain enzyme apollon).]	Go Component not found
O00327	[Aryl hydrocarbon receptor nuclear translocator-like protein 1 (Brain, and muscle ARNT-like 1) (Member of PAS protein 3) (Basic-helix-loop-, helix-PAS protein MOP3) (bHLH-PAS protein JAP3).]	Go Component not found
P25440	Bromodomain-containing protein 2 (Protein RING3) (O27.1.1).	Go Component not found

O60885	Bromodomain-containing protein 4 (HUNK1 protein).	Go Component not found
Q9UMQ3	Homeobox protein BarH-like 2.	Go Component not found
P43251	Biotinidase precursor (EC 3.5.1.12).	Go Component not found
Q15332	13.2 kDa protein in chromosome 11.	Go Component not found
P08123	Collagen alpha-2(I) chain precursor (Alpha-2 type I collagen).	Go Component not found
Q9NP86	Calcium-binding protein 5 (CaBP5).	Go Component not found
O75309	Cadherin-16 precursor (Kidney-specific cadherin) (Ksp-cadherin).	Go Component not found
O75493	[Carbonic anhydrase-related protein 11 precursor (CA-XI) (CARP XI) (CA-, RP XI) (Carbonic anhydrase-related protein 2) (CARP-2) (CA-RP II).]	Go Component not found
Q9Y6Q1	Calpain-6 (Calpamodulin) (CalpM) (Calpain-like protease X-linked).	Go Component not found
P56202	Cathepsin W precursor (EC 3.4.22.-) (Lymphopain).	Go Component not found
P08185	[Corticosteroid-binding globulin precursor (CBG) (Transcortin) (Serpin, A6).]	Go Component not found
Q9NTU7	Cerebellin-4 precursor (Cerebellin-like glycoprotein 1).	Go Component not found
P00746	[Complement factor D precursor (EC 3.4.21.46) (C3 convertase activator), (Properdin factor D) (Adipsin).]	Go Component not found
O15519	[CASP8 and FADD-like apoptosis regulator precursor (Cellular FLICE-like, inhibitory protein) (c-FLIP) (Caspase-eight-related protein) (Casper), (Caspase-like apoptosis regulatory protein) (CLARP) (MACH-related, inducer of toxicity) (MRIT) (Caspase homolog) (CASH) (Inhibitor of, FLICE) (I-FLICE) (FADD-like antiapoptotic molecule 1) (FLAME-1), (Usurpin) [Contains: CASP8 and FADD-like apoptosis regulator subunit, p43; CASP8 and FADD-like apoptosis regulator subunit p12].]	Go Component not found
O96020	G1/S-specific cyclin-E2.	Go Component not found
Q16880	[2-hydroxyacylsphingosine 1-beta-galactosyltransferase precursor, (EC 2.4.1.45) (UDP-galactose-ceramide galactosyltransferase) (Ceramide, UDP-galactosyltransferase) (Cerebroside synthase).]	Go Component not found
O14647	[Chromodomain-helicase-DNA-binding protein 2 (EC 3.6.1.-) (ATP-, dependent helicase CHD2) (CHD-2).]	Go Component not found
Q9NYG8	[Potassium channel subfamily K member 4 (TWIK-related arachidonic acid-, stimulated potassium channel protein) (TRAAK) (Two pore K(+) channel, KT4.1).]	Go Component not found
P57789	[Potassium channel subfamily K member 10 (Outward rectifying potassium, channel protein TREK-2) (TREK-2 K(+) channel subunit).]	Go Component not found
P51798	Chloride channel protein 7 (ClC-7).	Go Component not found

**P53675**	Clathrin heavy chain 2 (CLH-22).	Go Component not found
**Q01064**	[Calcium/calmodulin-dependent 3',5'-cyclic nucleotide phosphodiesterase, 1B (EC 3.1.4.17) (Cam-PDE 1B) (63 kDa Cam-PDE).]	Go Component not found
**P09543**	[2',3'-cyclic-nucleotide 3'-phosphodiesterase (EC 3.1.4.37) (CNP), (CNPase).]	Go Component not found
**O95263**	[High affinity cAMP-specific and IBMX-insensitive 3',5'-cyclic, phosphodiesterase 8B (EC 3.1.4.17) (HSPDE8B).]	Go Component not found
**P35913**	[Rod cGMP-specific 3',5'-cyclic phosphodiesterase subunit beta, precursor (EC 3.1.4.35) (GMP-PDE beta).]	Go Component not found
**P0C0L4**	[Complement C4-A precursor (Acidic complement C4) [Contains: Complement, C4 beta chain; Complement C4-A alpha chain; C4a anaphylatoxin; C4b-A;, C4d-A; Complement C4 gamma chain].]	Go Component not found
**P13671**	Complement component C6 precursor.	Go Component not found
**Q13085**	[Acetyl-CoA carboxylase 1 (EC 6.4.1.2) (ACC-alpha) [Includes: Biotin, carboxylase (EC 6.3.4.14)].]	Go Component not found
**Q16850**	[Cytochrome P450 51A1 (EC 1.14.13.70) (CYPLI) (P450LI) (Sterol 14-alpha, demethylase) (Lanosterol 14-alpha demethylase) (LDM) (P450-14DM), (P45014DM).]	Go Component not found
**Q9UNU6**	[Cytochrome P450 8B1 (EC 1.14.13.95) (CYPVIIIB1) (7-alpha-, hydroxycholest-4-en-3-one 12-alpha-hydroxylase) (Sterol 12-alpha-, hydroxylase) (7-alpha-hydroxy-4-cholesten-3-one 12-alpha-hydroxylase).]	Go Component not found
**P98187**	Cytochrome P450 4F8 (EC 1.14.14.1) (CYPIVF8).	Go Component not found
**P53673**	Beta-crystallin A4 (Beta-A4 crystallin).	Go Component not found
**P23508**	Colorectal mutant cancer protein (Protein MCC).	Go Component not found
**O14578**	[Citron Rho-interacting kinase (EC 2.7.11.1) (CRIK) (Rho-interacting,, serine/threonine-protein kinase 21).]	Go Component not found
**P39880**	Homeobox protein cut-like 1 (CCAAT displacement protein) (CDP).	Go Component not found
**Q14093**	Cylicin-2 (Cylicin II) (Multiple-band polypeptide II).	Go Component not found
**P62938**	[Peptidyl-prolyl cis-trans isomerase A (EC 5.2.1.8) (PPIase A), (Rotamase A) (Cyclophilin A) (Cyclosporin A-binding protein).]	Go Component not found
**O76096**	[Cystatin-F precursor (Leukocystatin) (Cystatin-7) (Cystatin-like, metastasis-associated protein) (CMAP).]	Go Component not found
**P06132**	Uroporphyrinogen decarboxylase (EC 4.1.1.37) (URO-D) (UPD).	Go Component not found
**O15523**	[ATP-dependent RNA helicase DDX3Y (EC 3.6.1.-) (DEAD box protein 3, Y-, chromosomal).]	Go Component not found
**P30837**	[Aldehyde dehydrogenase X, mitochondrial precursor (EC 1.2.1.3), (Aldehyde dehydrogenase family 1 member B1) (ALDH class 2).]	Go Component not found

**P80365**	[Corticosteroid 11-beta-dehydrogenase isozyme 2 (EC 1.1.1.-) (11-DH2), (11-beta-hydroxysteroid dehydrogenase type 2) (11-beta-HSD2) (NAD-, dependent 11-beta-hydroxysteroid dehydrogenase).]	Go Component not found
**P31689**	[DnaJ homolog subfamily A member 1 (Heat shock 40 kDa protein 4) (DnaJ, protein homolog 2) (HDJ-2) (HSJ-2) (HSDJ).]	Go Component not found
**Q16555**	[Dihydropyrimidinase-related protein 2 (DRP-2) (Collapsin response, mediator protein 2) (CRMP-2) (N2A3).]	Go Component not found
**Q13609**	[Deoxyribonuclease gamma precursor (EC 3.1.21.-) (DNase gamma), (Deoxyribonuclease I-like 3) (DNase I homolog protein DHP2) (Liver and, spleen DNase) (LS-DNase) (LSD).]	Go Component not found
**Q02487**	[Desmocollin-2 precursor (Desmosomal glycoprotein II and III), (Desmocollin-3).]	Go Component not found
**P63172**	[Dynein light chain Tctex-type 1 (T-complex testis-specific protein 1, homolog) (Protein CW-1).]	Go Component not found
**P00374**	Dihydrofolate reductase (EC 1.5.1.3).	Go Component not found
**Q13201**	[Multimerin-1 precursor (Endothelial cell multimerin 1) (EMILIN-4), (Elastin microfibril interface located protein 4) (Elastin microfibril, interfacer 4).]	Go Component not found
**P13639**	Elongation factor 2 (EF-2).	Go Component not found
**P55199**	[RNA polymerase II elongation factor ELL (Eleven-nineteen lysine-rich, leukemia protein).]	Go Component not found
**Q12926**	[ELAV-like protein 2 (Hu-antigen B) (HuB) (ELAV-like neuronal protein, 1) (Nervous system-specific RNA-binding protein Hel-N1).]	Go Component not found
**Q05524**	[Alpha-enolase, lung specific (EC 4.2.1.11) (2-phospho-D-glycerate, hydro-lyase) (Non-neural enolase) (NNE) (Phosphopyruvate hydratase), (HLE1).]	Go Component not found
**P62508**	[Estrogen-related receptor gamma (Estrogen receptor-related protein 3), (ERR gamma-2).]	Go Component not found
**P23141**	[Liver carboxylesterase 1 precursor (EC 3.1.1.1) (Acyl coenzyme, A:cholesterol acyltransferase) (ACAT) (Monocyte/macrophage serine, esterase) (HMSE) (Serine esterase 1) (Brain carboxylesterase hBr1), (Triacylglycerol hydrolase) (TGH) (Egasyn) (Retinyl ester hydrolase), (REH).]	Go Component not found
**P49640**	Homeobox even-skipped homolog protein 1 (EVX-1).	Go Component not found
**O00757**	[Fructose-1,6-bisphosphatase isozyme 2 (EC 3.1.3.11) (D-fructose-1,6-, bisphosphate 1-phosphohydrolase 2) (FBPase 2).]	Go Component not found
**Q16877**	[6-phosphofructo-2-kinase/fructose-2,6-biphosphatase 4 (6PF-2-K/Fru-, 2,6-P2ASE testis-type isozyme) [Includes: 6-phosphofructo-2-kinase, (EC 2.7.1.105); Fructose-2,6-bisphosphatase (EC 3.1.3.46)].]	Go Component not found
**O75844**	[CAAX prenyl protease 1 homolog (EC 3.4.24.84) (Prenyl protein-specific, endoprotease 1) (Farnesylated proteins-converting enzyme 1) (FACE-1), (Zinc metalloproteinase Ste24 homolog).]	Go Component not found
**O00507**	[Probable ubiquitin carboxyl-terminal hydrolase FAF-Y (EC 3.1.2.15), (Ubiquitin thioesterase FAF-Y) (Ubiquitin-specific-processing protease, FAF-Y) (Deubiquitinating enzyme FAF-Y) (Fat facets protein-related, Y-, linked) (Ubiquitin-specific protease 9, Y chromosome).]	Go Component not found
**Q14296**	Fas-activated serine/threonine kinase (EC 2.7.11.8) (FAST kinase).	Go Component not found

P21781	[Keratinocyte growth factor precursor (KGF) (Fibroblast growth factor, 7) (FGF-7) (HBGF-7).]	Go Component not found
Q99518	[Dimethylaniline monooxygenase [N-oxide-forming] 2 (EC 1.14.13.8), (Pulmonary flavin-containing monooxygenase 2) (FMO 2) (Dimethylaniline, oxidase 2) (FMO 1B1).]	Go Component not found
Q9Y297	[F-box/WD repeat-containing protein 1A (F-box and WD repeats protein, beta-TrCP) (E3RSIkappaB) (pIkappaBalpha-E3 receptor subunit).]	Go Component not found
Q08050	[Forkhead box protein M1 (Forkhead-related protein FKHL16) (Hepatocyte, nuclear factor 3 forkhead homolog 11) (HNF-3/fork-head homolog 11), (HFH-11) (Winged-helix factor from INS-1 cells) (M-phase, phosphoprotein 2) (MPM-2 reactive phosphoprotein 2) (Transcription, factor Trident).]	Go Component not found
P34059	[N-acetylgalactosamine-6-sulfatase precursor (EC 3.1.6.4) (N-, acetylgalactosamine-6-sulfate sulfatase) (Galactose-6-sulfate, sulfatase) (GalNAc6S sulfatase) (Chondroitinsulfatase), (Chondroitinase).]	Go Component not found
P87889	[HERV-K_5q33.3 provirus ancestral Gag polyprotein (Gag polyprotein), (HERV-K10 Gag protein) (HERV-K107 Gag protein) [Contains: Matrix, protein; Capsid protein; Nucleocapsid protein].]	Go Component not found
P38405	[Guanine nucleotide-binding protein G(olf) subunit alpha (Adenylate, cyclase-stimulating G alpha protein, olfactory type).]	Go Component not found
P32456	[Interferon-induced guanylate-binding protein 2 (GTP-binding protein 2), (Guanine nucleotide-binding protein 2) (GBP-2) (HuGBP-2).]	Go Component not found
Q9UK05	[Growth/differentiation factor 2 precursor (GDF-2) (Bone morphogenetic, protein 9) (BMP-9).]	Go Component not found
P15586	[N-acetylglucosamine-6-sulfatase precursor (EC 3.1.6.14) (G6S), (Glucosamine-6-sulfatase).]	Go Component not found
Q04446	[1,4-alpha-glucan-branching enzyme (EC 2.4.1.18) (Glycogen-branching, enzyme) (Brancher enzyme).]	Go Component not found
P57057	[Glycerol-3-phosphate transporter (G-3-P transporter) (G-3-P permease), (Solute carrier family 37 member 1).]	Go Component not found
P46926	[Glucosamine-6-phosphate isomerase (EC 3.5.99.6) (Glucosamine-6-, phosphate deaminase) (GNPDA) (GlcN6P deaminase) (Oscillin).]	Go Component not found
P48506	[Glutamate--cysteine ligase catalytic subunit (EC 6.3.2.2) (Gamma-, glutamylcysteine synthetase) (Gamma-ECS) (GCS heavy chain).]	Go Component not found
P98176	Histone H2A-Bbd (H2A Barr body-deficient) (H2A.Bbd).	Go Component not found
P61920	[Hemoglobin subunit gamma-1 (Hemoglobin gamma-1 chain) (Gamma-1-globin), (Hemoglobin gamma-A chain).]	Go Component not found
Q14469	[Transcription factor HES-1 (Hairy and enhancer of split 1) (Hairy-, like) (HHL) (Hairy homolog).]	Go Component not found
P46597	[Hydroxyindole O-methyltransferase (EC 2.1.1.4) (HIOMT), (Acetylserotonin O-methyltransferase) (ASMT).]	Go Component not found
P50135	Histamine N-methyltransferase (EC 2.1.1.8) (HMT).	Go Component not found
P20823	[Hepatocyte nuclear factor 1-alpha (HNF-1A) (Liver-specific, transcription factor LF-B1) (LFB1) (Transcription factor 1) (TCF-1).]	Go Component not found

**P34931**	[Heat shock 70 kDa protein 1L (Heat shock 70 kDa protein 1-like) (Heat, shock 70 kDa protein 1-Hom) (HSP70-Hom).]	Go Component not found
**P25092**	[Heat-stable enterotoxin receptor precursor (EC 4.6.1.2) (GC-C), (Intestinal guanylate cyclase) (STA receptor) (hSTAR).]	Go Component not found
**P42357**	Histidine ammonia-lyase (EC 4.3.1.3) (Histidase).	Go Component not found
**P31260**	Homeobox protein Hox-A10 (Hox-1H) (Hox-1.8) (PL).	Go Component not found
**Q00444**	Homeobox protein Hox-C5 (Hox-3D) (CP11).	Go Component not found
**O43248**	Homeobox protein Hox-C11.	Go Component not found
**P35453**	Homeobox protein Hox-D13 (Hox-4I).	Go Component not found
**P24592**	[Insulin-like growth factor-binding protein 6 precursor (IGFBP-6) (IBP-, 6) (IGF-binding protein 6).]	Go Component not found
**P51878**	[Caspase-5 precursor (EC 3.4.22.58) (CASP-5) (ICH-3 protease) (TY, protease) (ICE(rel)-III) [Contains: Caspase-5 subunit p20; Caspase-5, subunit p10].]	Go Component not found
**O60841**	[Eukaryotic translation initiation factor 5B (eIF-5B) (Translation, initiation factor IF-2).]	Go Component not found
**P49895**	[Type I iodothyronine deiodinase (EC 1.97.1.10) (Type-I 5'-deiodinase), (DIOI) (Type 1 DI) (5DI).]	Go Component not found
**Q9Y2B9**	cAMP-dependent protein kinase inhibitor gamma (PKI-gamma).	Go Component not found
**P10914**	Interferon regulatory factor 1 (IRF-1).	Go Component not found
**O14896**	Interferon regulatory factor 6 (IRF-6).	Go Component not found
**P52333**	[Tyrosine-protein kinase JAK3 (EC 2.7.10.2) (Janus kinase 3) (JAK-3), (Leukocyte janus kinase) (L-JAK).]	Go Component not found
**P13646**	[Keratin, type I cytoskeletal 13 (Cytokeratin-13) (CK-13) (Keratin-13), (K13).]	Go Component not found
**P78368**	Casein kinase I isoform gamma-2 (EC 2.7.11.1) (CKI-gamma 2).	Go Component not found
**Q16566**	[Calcium/calmodulin-dependent protein kinase type IV (EC 2.7.11.17), (CAM kinase-GR) (CaMK IV).]	Go Component not found
**P52824**	[Diacylglycerol kinase theta (EC 2.7.1.107) (Diglyceride kinase theta), (DGK-theta) (DAG kinase theta).]	Go Component not found
**Q13237**	[cGMP-dependent protein kinase 2 (EC 2.7.11.12) (CGK 2) (cGKII) (Type, II cGMP-dependent protein kinase).]	Go Component not found
**Q9UH77**	Kelch-like protein 3.	Go Component not found

Q15726	[Metastasis-suppressor KiSS-1 precursor (Kisspeptin-1) [Contains:, Metastin (Kisspeptin-54); Kisspeptin-14; Kisspeptin-13; Kisspeptin-, 10].]	Go Component not found
Q9Y4X4	Krueppel-like factor 12 (Transcriptional repressor AP-2rep).	Go Component not found
P49862	[Kallikrein-7 precursor (EC 3.4.21.117) (hK7) (Stratum corneum, chymotryptic enzyme) (hSCCE) (Serine protease 6).]	Go Component not found
P46020	[Phosphorylase b kinase regulatory subunit alpha, skeletal muscle, isoform (Phosphorylase kinase alpha M subunit).]	Go Component not found
P11908	[Ribose-phosphate pyrophosphokinase II (EC 2.7.6.1) (Phosphoribosyl, pyrophosphate synthetase II) (PRS-II) (PPRibP).]	Go Component not found
Q13449	Limbic system-associated membrane protein precursor (LSAMP).	Go Component not found
P33121	[Long-chain-fatty-acid--CoA ligase 1 (EC 6.2.1.3) (Long-chain acyl-CoA, synthetase 1) (LACS 1) (Palmitoyl-CoA ligase 1) (Long-chain fatty acid, CoA ligase 2) (Long-chain acyl-CoA synthetase 2) (LACS 2) (Acyl-CoA, synthetase 1) (ACS1) (Palmitoyl-CoA ligase 2).]	Go Component not found
P48357	[Leptin receptor precursor (LEP-R) (OB receptor) (OB-R) (HuB219) (CD295, antigen).]	Go Component not found
P07098	[Gastric triacylglycerol lipase precursor (EC 3.1.1.3) (Gastric lipase), (GL).]	Go Component not found
P09917	Arachidonate 5-lipoxygenase (EC 1.13.11.34) (5-lipoxygenase) (5-LO).	Go Component not found
Q14596	[Next to BRCA1 gene 1 protein (Neighbor of BRCA1 gene 1 protein), (Membrane component, chromosome 17, surface marker 2) (1A1-3B).]	Go Component not found
Q99683	[Mitogen-activated protein kinase kinase kinase 5 (EC 2.7.11.25), (MAPK/ERK kinase kinase 5) (MEK kinase 5) (MEKK 5) (Apoptosis signal-, regulating kinase 1) (ASK-1).]	Go Component not found
P43360	Melanoma-associated antigen 6 (MAGE-6 antigen) (MAGE3B).	Go Component not found
P20794	[Serine/threonine-protein kinase MAK (EC 2.7.11.22) (Male germ cell-, associated kinase).]	Go Component not found
O00462	[Beta-mannosidase precursor (EC 3.2.1.25) (Lysosomal beta A, mannosidase) (Mannanase) (Mannase).]	Go Component not found
P08235	Mineralocorticoid receptor (MR).	Go Component not found
Q14814	Myocyte-specific enhancer factor 2D.	Go Component not found
Q08431	[Lactadherin precursor (Milk fat globule-EGF factor 8) (MFG-E8) (HMFG), (Breast epithelial antigen BA46) (MFGM) [Contains: Lactadherin short, form; Medin].]	Go Component not found
O15479	[Melanoma-associated antigen B2 (MAGE-B2 antigen) (DSS-AHC critical, interval MAGE superfamily 6) (DAM6) (MAGE XP-2).]	Go Component not found
Q9UJ55	MAGE-like protein 2 (Necdin-like protein 1) (Protein nM15).	Go Component not found

P31152	[Mitogen-activated protein kinase 4 (EC 2.7.11.24) (Extracellular, signal-regulated kinase 4) (ERK-4) (MAP kinase isoform p63) (p63-, MAPK).]	Go Component not found
P41218	Myeloid cell nuclear differentiation antigen.	Go Component not found
P46734	[Dual specificity mitogen-activated protein kinase kinase 3, (EC 2.7.12.2) (MAP kinase kinase 3) (MAPKK 3) (MAPK/ERK kinase 3).]	Go Component not found
Q06455	[Protein CBFA2T1 (Protein MTG8) (Protein ETO) (Eight twenty one, protein) (Cyclin-D-related protein) (Zinc finger MYND domain-, containing protein 2).]	Go Component not found
Q13613	Myotubularin-related protein 1 (EC 3.1.3.-).	Go Component not found
Q02817	Mucin-2 precursor (Intestinal mucin-2).	Go Component not found
Q9UKN7	Myosin-XV (Unconventional myosin-15).	Go Component not found
P11055	[Myosin-3 (Myosin heavy chain 3) (Myosin heavy chain, fast skeletal, muscle, embryonic) (Muscle embryonic myosin heavy chain) (SMHCE).]	Go Component not found
P54296	[Myomesin-2 (M-protein) (165 kDa titin-associated protein) (165 kDa, connectin-associated protein).]	Go Component not found
Q15274	[Nicotinate-nucleotide pyrophosphorylase [carboxylating] (EC 2.4.2.19), (Quinolinate phosphoribosyltransferase [decarboxylating]) (QAPRTase), (QPRTase).]	Go Component not found
Q9Y2A7	[Nck-associated protein 1 (NAP 1) (p125Nap1) (Membrane-associated, protein HEM-2).]	Go Component not found
P62166	[Neuronal calcium sensor 1 (NCS-1) (Frequenin homolog) (Frequenin-like, protein) (Frequenin-like ubiquitous protein).]	Go Component not found
Q9ULJ8	[Neurabin-1 (Neurabin-I) (Neural tissue-specific F-actin-binding, protein I) (Protein phosphatase 1 regulatory subunit 9A).]	Go Component not found
Q92832	[Protein kinase C-binding protein NELL1 precursor (NEL-like protein 1), (Nel-related protein 1).]	Go Component not found
P56730	[Neurotrypsin precursor (EC 3.4.21.-) (Serine protease 12) (Motopsin), (Leydin).]	Go Component not found
Q14934	[Nuclear factor of activated T-cells, cytoplasmic 4 (NF-ATc4) (NFATc4), (T-cell transcription factor NFAT3) (NF-AT3).]	Go Component not found
P08651	[Nuclear factor 1 C-type (Nuclear factor 1/C) (NF1-C) (NFI-C) (NF-I/C), (CCAAT-box-binding transcription factor) (CTF) (TGGCA-binding, protein).]	Go Component not found
Q14494	[Nuclear factor erythroid 2-related factor 1 (NF-E2-related factor 1), (NFE2-related factor 1) (Nuclear factor, erythroid derived 2, like 1), (Transcription factor 11) (Transcription factor HBZ17) (Transcription, factor LCR-F1) (Locus control region-factor 1).]	Go Component not found
Q99742	[Neuronal PAS domain-containing protein 1 (Neuronal PAS1) (Member of, PAS protein 5) (Basic-helix-loop-helix-PAS protein MOP5).]	Go Component not found
P61916	[Epididymal secretory protein E1 precursor (Niemann-Pick disease type, C2 protein) (hE1).]	Go Component not found
P47972	Neuronal pentraxin-2 precursor (NP2) (Neuronal pentraxin II) (NP-II).	Go Component not found

O14786	[Neuropilin-1 precursor (Vascular endothelial cell growth factor 165, receptor) (CD304 antigen).]	Go Component not found
Q9BS92	Protein NipSnap3B (SNAP1).	Go Component not found
Q14249	Endonuclease G, mitochondrial precursor (EC 3.1.30.-) (Endo G).	Go Component not found
P53370	[Nucleoside diphosphate-linked moiety X motif 6 (Nudix motif 6), (Protein GFG) (GFG-1) (Antisense basic fibroblast growth factor).]	Go Component not found
Q9P2S2	Neurexin-2-alpha precursor (Neurexin II-alpha).	Go Component not found
Q13606	[Olfactory receptor 5I1 (Olfactory receptor OR11-159) (Olfactory, receptor-like protein OLF1).]	Go Component not found
Q16633	[POU domain class 2-associating factor 1 (B-cell-specific coactivator, OBF-1) (OCT-binding factor 1) (BOB-1) (OCA-B).]	Go Component not found
Q02218	[2-oxoglutarate dehydrogenase E1 component, mitochondrial precursor, (EC 1.2.4.2) (Alpha-ketoglutarate dehydrogenase).]	Go Component not found
O94826	[Mitochondrial precursor proteins import receptor (Translocase of outer, membrane TOM70).]	Go Component not found
Q9NRC9	[Otoraplin precursor (Fibrocyte-derived protein) (Melanoma inhibitory, activity-like protein).]	Go Component not found
Q10472	[Polypeptide N-acetylgalactosaminyltransferase 1 (EC 2.4.1.41), (Protein-UDP acetylgalactosaminyltransferase 1) (UDP-, GalNAc:polypeptide N-acetylgalactosaminyltransferase 1) (Polypeptide, GalNAc transferase 1) (GalNAc-T1) (pp-GaNTase 1) [Contains:, Polypeptide N-acetylgalactosaminyltransferase 1 soluble form].]	Go Component not found
P15863	Paired box protein Pax-1 (HUP48).	Go Component not found
P23759	Paired box protein Pax-7 (HUP1).	Go Component not found
P40425	[Pre-B-cell leukemia transcription factor 2 (Homeobox protein PBX2), (Protein G17).]	Go Component not found
Q15113	[Procollagen C-endopeptidase enhancer 1 precursor (Procollagen COOH-, terminal proteinase enhancer 1) (Procollagen C-proteinase enhancer 1), (PCPE-1) (Type I procollagen COOH-terminal proteinase enhancer) (Type, 1 procollagen C-proteinase enhancer protein).]	Go Component not found
Q9ULC6	[Protein-arginine deiminase type-1 (EC 3.5.3.15) (Protein-arginine, deiminase type I) (Peptidylarginine deiminase I).]	Go Component not found
O15534	[Period circadian protein homolog 1 (Circadian clock protein PERIOD 1), (Circadian pacemaker protein Rigui) (hPER1).]	Go Component not found
P98160	[Basement membrane-specific heparan sulfate proteoglycan core protein, precursor (HSPG) (Perlecan) (PLC).]	Go Component not found
P07585	Decorin precursor (Bone proteoglycan II) (PG-S2) (PG40).	Go Component not found
P06737	Glycogen phosphorylase, liver form (EC 2.4.1.1).	Go Component not found

**Q9UHT4**	Putative protein PRO1854.	Go Component not found
**P07738**	[Bisphosphoglycerate mutase (EC 5.4.2.4) (2,3-bisphosphoglycerate, mutase, erythrocyte) (2,3-bisphosphoglycerate synthase) (BPGM), (EC 5.4.2.1) (EC 3.1.3.13) (BPG-dependent PGAM).]	Go Component not found
**P00491**	[Purine nucleoside phosphorylase (EC 2.4.2.1) (Inosine phosphorylase), (PNP).]	Go Component not found
**Q15165**	[Serum paraoxonase/arylesterase 2 (EC 3.1.1.2) (EC 3.1.8.1) (PON 2), (Serum aryldialkylphosphatase 2) (A-esterase 2) (Aromatic esterase 2).]	Go Component not found
**Q9NQV6**	PR domain zinc finger protein 10 (PR domain-containing protein 10).	Go Component not found
**P49643**	[DNA primase large subunit (EC 2.7.7.-) (DNA primase 58 kDa subunit), (p58).]	Go Component not found
**Q99935**	[Proline-rich protein 1 precursor (PRL1) (Basic proline-rich lacrimal, protein).]	Go Component not found
**P07737**	Profilin-1 (Profilin I).	Go Component not found
**P22891**	Vitamin K-dependent protein Z precursor.	Go Component not found
**Q6S8J3**	Prostate, ovary, testis-expressed protein on chromosome 2 (Fragment).	Go Component not found
**P26045**	[Tyrosine-protein phosphatase non-receptor type 3 (EC 3.1.3.48), (Protein-tyrosine phosphatase H1) (PTP-H1).]	Go Component not found
**P27708**	[CAD protein [Includes: Glutamine-dependent carbamoyl-phosphate, synthase (EC 6.3.5.5); Aspartate carbamoyltransferase (EC 2.1.3.2);, Dihydroorotase (EC 3.5.2.3)].]	Go Component not found
**P54725**	UV excision repair protein RAD23 homolog A (hHR23A).	Go Component not found
**P55042**	GTP-binding protein RAD (RAS associated with diabetes) (RAD1).	Go Component not found
**Q13702**	[43 kDa receptor-associated protein of the synapse (RAPsyn), (Acetylcholine receptor-associated 43 kDa protein) (43 kDa, postsynaptic protein) (RING finger protein 205).]	Go Component not found
**Q13636**	Ras-related protein Rab-31 (Rab-22B).	Go Component not found
**P51148**	Ras-related protein Rab-5C (RAB5L) (L1880).	Go Component not found
**P98179**	Putative RNA-binding protein 3 (RNA-binding motif protein 3) (RNPL).	Go Component not found
**Q9NZL6**	Ral guanine nucleotide dissociation stimulator-like 1 (RalGDS-like 1).	Go Component not found
**P49802**	Regulator of G-protein signaling 7 (RGS7).	Go Component not found
**Q15835**	[Rhodopsin kinase precursor (EC 2.7.11.14) (RK) (G protein-coupled, receptor kinase 1).]	Go Component not found

P83111	[Serine beta-lactamase-like protein LACTB, mitochondrial precursor, (EC 3.4.-.-).]	Go Component not found
P35398	[Nuclear receptor ROR-alpha (Retinoid-related orphan receptor-alpha), (Nuclear receptor RZR-alpha).]	Go Component not found
Q92766	[RAS-responsive element-binding protein 1 (RREB-1) (Raf-responsive zinc, finger protein LZ321) (Zinc finger motif-enhancer binding-protein 1), (Zep-1) (Finger protein in nuclear bodies).]	Go Component not found
Q9UET6	[Putative ribosomal RNA methyltransferase 1 (EC 2.1.1.-) (rRNA, (uridine-2'-O-)-methyltransferase).]	Go Component not found
Q92545	Transmembrane protein 131 (Protein RW1).	Go Component not found
P61619	[Protein transport protein Sec61 subunit alpha isoform 1 (Sec61 alpha-, 1).]	Go Component not found
Q13228	Selenium-binding protein 1 (56 kDa selenium-binding protein) (SP56).	Go Component not found
Q14141	Septin-6.	Go Component not found
Q13247	[Splicing factor, arginine/serine-rich 6 (Pre-mRNA-splicing factor, SRP55).]	Go Component not found
P05060	[Secretogranin-1 precursor (Secretogranin I) (SgI) (Chromogranin-B), (CgB) [Contains: GAWK peptide; CCB peptide].]	Go Component not found
P81133	Single-minded homolog 1.	Go Component not found
Q13591	Semaphorin-5A precursor (Semaphorin F) (Sema F).	Go Component not found
O75326	[Semaphorin-7A precursor (Semaphorin L) (Sema L) (Semaphorin K1) (Sema, K1) (John-Milton-Hargen human blood group Ag) (JMH blood group, antigen) (CD108 antigen) (CDw108).]	Go Component not found
P41229	[Histone demethylase JARID1C (EC 1.14.11.-) (Jumonji/ARID domain-, containing protein 1C) (Protein SmcX) (Protein Xe169).]	Go Component not found
Q9UNH6	Sorting nexin-7.	Go Component not found
Q07890	Son of sevenless homolog 2 (SOS-2).	Go Component not found
Q9BY50	[Signal peptidase complex catalytic subunit SEC11C (EC 3.4.-.-) (SEC11, homolog C) (SEC11-like protein 3) (Microsomal signal peptidase 21 kDa, subunit) (SPase 21 kDa subunit) (SPC21).]	Go Component not found
Q15513	Protein SPHAR (S-phase response protein).	Go Component not found
Q99611	[Selenide, water dikinase 2 (EC 2.7.9.3) (Selenophosphate synthetase 2), (Selenium donor protein 2).]	Go Component not found
Q9Y6E0	[Serine/threonine-protein kinase 24 (EC 2.7.11.1) (STE20-like kinase, MST3) (MST-3) (Mammalian STE20-like protein kinase 3).]	Go Component not found
Q9UN79	[SOX-13 protein (Type 1 diabetes autoantigen ICA12) (Islet cell antigen, 12).]	Go Component not found

**Q15031**	[Probable leucyl-tRNA synthetase, mitochondrial precursor (EC 6.1.1.4), (Leucine--tRNA ligase) (LeuRS).]	Go Component not found
**P47897**	[Glutaminyl-tRNA synthetase (EC 6.1.1.18) (Glutamine--tRNA ligase), (GlnRS).]	Go Component not found
**P52655**	[Transcription initiation factor IIA subunit 1 (General transcription, factor IIA1) (TFIIA-42) (TFIIAL) [Contains: Transcription initiation, factor IIA alpha chain (TFIIA p35 subunit); Transcription initiation, factor IIA beta chain (TFIIA p19 subunit)].]	Go Component not found
**O43657**	[Tetraspanin-6 (Tspan-6) (Transmembrane 4 superfamily member 6) (T245, protein) (Tetraspanin TM4-D) (A15 homolog).]	Go Component not found
**O95359**	[Transforming acidic coiled-coil-containing protein 2 (Anti Zuai-1), (AZU-1).]	Go Component not found
**P17542**	[T-cell acute lymphocytic leukemia protein 1 (TAL-1) (Stem cell, protein) (T-cell leukemia/lymphoma protein 5).]	Go Component not found
**Q92844**	[TRAF family member-associated NF-kappa-B activator (TRAF-interacting, protein) (I-TRAF).]	Go Component not found
**P68367**	[Tubulin alpha-4A chain (Tubulin alpha-1 chain) (Alpha-tubulin 1), (Testis-specific alpha-tubulin).]	Go Component not found
**Q92526**	[T-complex protein 1 subunit zeta-2 (TCP-1-zeta-2) (CCT-zeta-2) (TCP-1-, zeta-like) (CCT-zeta-like) (Testis-specific Tcp20) (Testis-specific, protein TSA303).]	Go Component not found
**P19532**	Transcription factor E3.	Go Component not found
**Q99969**	[Retinoic acid receptor responder protein 2 precursor (Tazarotene-, induced gene 2 protein) (RAR-responsive protein TIG2).]	Go Component not found
**Q07352**	[Butyrate response factor 1 (Protein TIS11B) (EGF-response factor 1), (ERF-1).]	Go Component not found
**P56847**	Protein TNG2.	Go Component not found
**P40225**	[Thrombopoietin precursor (Megakaryocyte colony-stimulating factor), (Myeloproliferative leukemia virus oncogene ligand) (C-mpl ligand), (ML) (Megakaryocyte growth and development factor) (MGDF).]	Go Component not found
**P13056**	Orphan nuclear receptor TR2 (Testicular receptor 2).	Go Component not found
**O75962**	[Triple functional domain protein (EC 2.7.11.1) (PTPRF-interacting, protein).]	Go Component not found
**P45378**	[Troponin T, fast skeletal muscle (TnTf) (Fast skeletal muscle troponin, T) (fTnT) (Beta TnTF).]	Go Component not found
**Q99598**	Translin-associated protein X (Translin-associated factor X).	Go Component not found
**P53804**	[Tetratricopeptide repeat protein 3 (TPR repeat protein 3) (TPR repeat, protein D) (RING finger protein 105).]	Go Component not found
**O60806**	[T-box transcription factor TBX19 (T-box protein 19) (T-box factor,, pituitary).]	Go Component not found
**P29597**	Non-receptor tyrosine-protein kinase TYK2 (EC 2.7.10.2).	Go Component not found

**P04818**	Thymidylate synthase (EC 2.1.1.45) (TS) (TSase).	Go Component not found
**O00762**	[Ubiquitin-conjugating enzyme E2 C (EC 6.3.2.19) (Ubiquitin-protein, ligase C) (Ubiquitin carrier protein C) (UbcH10).]	Go Component not found
**O14978**	[Zinc finger protein 263 (Zinc finger protein FPM315) (Zinc finger, protein with KRAB and SCAN domains 12).]	Go Component not found
**O15015**	Zinc finger protein 646.	Go Component not found
**O15027**	SEC16 homolog A.	Go Component not found
**O43301**	Heat shock 70 kDa protein 12A.	Go Component not found
**O60299**	Uncharacterized protein KIAA0552.	Go Component not found
**O60813**	PRAME family member 11.	Go Component not found
**O75069**	Transmembrane and coiled-coil domains protein 2 (Cerebral protein 11).	Go Component not found
**O75385**	[Serine/threonine-protein kinase ULK1 (EC 2.7.11.1) (Unc-51-like kinase, 1).]	Go Component not found
**O94889**	Kelch-like protein 18.	Go Component not found
**O95409**	Zinc finger protein ZIC 2 (Zinc finger protein of the cerebellum 2).	Go Component not found
**O95522**	PRAME family member 12.	Go Component not found
**O96014**	Protein Wnt-11 precursor.	Go Component not found
**P15498**	Proto-oncogene vav.	Go Component not found
**P78381**	[UDP-galactose translocator (UDP-galactose transporter) (UGT) (UDP-Gal-, Tr) (Solute carrier family 35 member A2).]	Go Component not found
**Q12767**	Uncharacterized protein KIAA0195 (Transmembrane protein 94).	Go Component not found
**Q14146**	Uncharacterized protein KIAA0133.	Go Component not found
**Q14694**	[Ubiquitin carboxyl-terminal hydrolase 10 (EC 3.1.2.15) (Ubiquitin, thioesterase 10) (Ubiquitin-specific-processing protease 10), (Deubiquitinating enzyme 10).]	Go Component not found
**Q15776**	[Zinc finger protein 192 (LD5-1) (Zinc finger protein with KRAB and, SCAN domains 8).]	Go Component not found
**Q15928**	Zinc finger protein 141.	Go Component not found

**Q92610**	Zinc finger protein 592.	Go Component not found
**Q93073**	Uncharacterized protein KIAA0256.	Go Component not found
**Q9HC78**	[Zinc finger and BTB domain-containing protein 20 (Zinc finger protein, 288) (Dendritic-derived BTB/POZ zinc finger protein).]	Go Component not found
**Q9NY84**	Vascular non-inflammatory molecule 3 precursor (Vanin-3).	Go Component not found
**Q9NZL3**	[Zinc finger protein 224 (Zinc finger protein 27) (Zinc finger protein, 233) (Zinc finger protein 255) (Bone marrow zinc finger 2) (BMZF-2), (Zinc finger protein KOX22).]	Go Component not found
**Q9UIE0**	Zinc finger protein 230 (Zinc finger protein FDZF2).	Go Component not found
**Q9UK13**	Zinc finger protein 221.	Go Component not found
**Q9ULK2**	Ataxin-7-like protein 1 (Fragment).	Go Component not found
**Q9Y473**	Zinc finger protein 175 (Zinc finger protein OTK18).	Go Component not found
**P98194**	[Calcium-transporting ATPase type 2C member 1 (EC 3.6.3.8) (ATPase 2C1), (ATP-dependent Ca(2+) pump PMR1).]	Golgi
**P98194**	[Calcium-transporting ATPase type 2C member 1 (EC 3.6.3.8) (ATPase 2C1), (ATP-dependent Ca(2+) pump PMR1).]	Golgi
**P45844**	[ATP-binding cassette sub-family G member 1 (White protein homolog), (ATP-binding cassette transporter 8).]	Golgi
**P98194**	[Calcium-transporting ATPase type 2C member 1 (EC 3.6.3.8) (ATPase 2C1), (ATP-dependent Ca(2+) pump PMR1).]	Golgi
**Q9UBV7**	[Beta-1,4-galactosyltransferase 7 (EC 2.4.1.-) (Beta-1,4-GalTase 7), (Beta4Gal-T7) (b4Gal-T7) (UDP-galactose:beta-N-acetylglucosamine beta-, 1,4-galactosyltransferase 7) (UDP-Gal:beta-GlcNAc beta-1,4-, galactosyltransferase 7) [Includes: Xylosylprotein 4-beta-, galactosyltransferase (EC 2.4.1.133) (UDP-galactose:beta-xylose beta-, 1,4-galactosyltransferase) (Xylosylprotein beta-1,4-, galactosyltransferase) (XGPT) (Proteoglycan UDP-galactose:beta-xylose, beta1,4-galactosyltransferase I) (XGalT-1)].]	Golgi
**P56750**	Claudin-17.	Golgi
**P98174**	[FYVE, RhoGEF and PH domain-containing protein 1 (Faciogenital, dysplasia 1 protein) (Zinc finger FYVE domain-containing protein 3), (Rho/Rac guanine nucleotide exchange factor FGD1) (Rho/Rac GEF).]	Golgi
**P19526**	[Galactoside 2-alpha-L-fucosyltransferase 1 (EC 2.4.1.69) (GDP-L-, fucose:beta-D-galactoside 2-alpha-L-fucosyltransferase 1), (Alpha(1,2)FT 1) (Fucosyltransferase 1) (Blood group H alpha 2-, fucosyltransferase).]	Golgi
**Q92538**	[Golgi-specific brefeldin A-resistance guanine nucleotide exchange, factor 1 (BFA-resistant GEF 1).]	Golgi
**Q13439**	[Golgin subfamily A member 4 (Trans-Golgi p230) (256 kDa golgin), (Golgin-245) (Protein 72.1).]	Golgi
**P31948**	[Stress-induced-phosphoprotein 1 (STI1) (Hsc70/Hsp90-organizing, protein) (Hop) (Transformation-sensitive protein IEF SSP 3521) (NY-, REN-11 antigen).]	Golgi
**Q99550**	M-phase phosphoprotein 9.	Golgi
**Q01968**	[Inositol polyphosphate 5-phosphatase OCRL-1 (EC 3.1.3.36) (Lowe, oculocerebrorenal syndrome protein).]	Golgi
**Q16549**	[Proprotein convertase subtilisin/kexin type 7 precursor (EC 3.4.21.-), (Proprotein convertase PC7) (Subtilisin/kexin-like protease PC7), (Prohormone convertase PC7) (PC8) (hPC8) (Lymphoma proprotein, convertase).]	Golgi
**Q9Y2D2**	[UDP-N-acetylglucosamine transporter (Golgi UDP-GlcNAc transporter), (Solute carrier family 35 member A3).]	Golgi

P15144	[Aminopeptidase N (EC 3.4.11.2) (hAPN) (Alanyl aminopeptidase), (Microsomal aminopeptidase) (Aminopeptidase M) (gp150) (Myeloid plasma, membrane glycoprotein CD13) (CD13 antigen).]	Golgi
Q16394	[Exostosin-1 (EC 2.4.1.224) (EC 2.4.1.225) (Glucuronosyl-N-, acetylglucosaminyl-proteoglycan/N-acetylglucosaminyl-proteoglycan 4-, alpha-N-acetylglucosaminyltransferase) (Putative tumor suppressor, protein EXT1) (Multiple exostoses protein 1).]	Golgi
Q92896	[Golgi apparatus protein 1 precursor (Golgi sialoglycoprotein MG-160), (E-selectin ligand 1) (ESL-1) (Cysteine-rich fibroblast growth factor, receptor) (CFR-1).]	Golgi
Q02818	Nucleobindin-1 precursor (CALNUC).	Golgi
P42356	[Phosphatidylinositol 4-kinase alpha (EC 2.7.1.67) (PI4-kinase alpha), (PtdIns-4-kinase alpha) (PI4K-alpha).]	Golgi
P49755	[Transmembrane emp24 domain-containing protein 10 precursor, (Transmembrane protein Tmp21) (21 kDa transmembrane-trafficking, protein) (p24delta) (S31III125) (S31I125) (Tmp-21-I).]	Golgi
O60763	[General vesicular transport factor p115 (Transcytosis-associated, protein) (TAP) (Vesicle docking protein).]	Golgi
P36406	[GTP-binding protein ARD-1 (ADP-ribosylation factor domain-containing, protein 1) (Tripartite motif-containing protein 23) (RING finger, protein 46).]	Golgi
P98194	[Calcium-transporting ATPase type 2C member 1 (EC 3.6.3.8) (ATPase 2C1), (ATP-dependent Ca(2+) pump PMR1).]	Golgi
P22460	[Potassium voltage-gated channel subfamily A member 5 (Voltage-gated, potassium channel subunit Kv1.5) (HK2) (HPCN1).]	Golgi
P02751	[Fibronectin precursor (FN) (Cold-insoluble globulin) (CIG) [Contains:, Ugl-Y1; Ugl-Y2; Ugl-Y3].]	Golgi
P09958	[Furin precursor (EC 3.4.21.75) (Paired basic amino acid residue, cleaving enzyme) (PACE) (Dibasic-processing enzyme).]	Golgi
Q9NZ52	[ADP-ribosylation factor-binding protein GGA3 (Golgi-localized, gamma, ear-containing, ARF-binding protein 3).]	Golgi
Q09328	[Alpha-1,6-mannosylglycoprotein 6-beta-N-acetylglucosaminyltransferase, A (EC 2.4.1.155) (Mannoside acetylglucosaminyltransferase 5) (Alpha-, mannoside beta-1,6-N-acetylglucosaminyltransferase) (N-, acetylglucosaminyl-transferase V) (GNT-V) (GlcNAc-T V).]	Golgi
P42858	Huntingtin (Huntington disease protein) (HD protein).	Golgi
P49641	[Alpha-mannosidase IIx (EC 3.2.1.114) (Mannosyl-oligosaccharide 1,3-, 1,6-alpha-mannosidase) (MAN IIx) (Mannosidase alpha class 2A member, 2).]	Golgi
O15258	Protein RER1.	Golgi
Q92834	X-linked retinitis pigmentosa GTPase regulator.	Golgi
Q9Y2T2	[AP-3 complex subunit mu-1 (Adapter-related protein complex 3 mu-1, subunit) (Mu-adaptin 3A) (AP-3 adapter complex mu3A subunit).]	Lysosomal
P15848	[Arylsulfatase B precursor (EC 3.1.6.12) (ASB) (N-acetylgalactosamine-, 4-sulfatase) (G4S).]	Lysosomal
P10253	[Lysosomal alpha-glucosidase precursor (EC 3.2.1.20) (Acid maltase), (Aglucosidase alfa) [Contains: 76 kDa lysosomal alpha-glucosidase; 70, kDa lysosomal alpha-glucosidase].]	Lysosomal
Q99519	[Sialidase-1 precursor (EC 3.2.1.18) (Lysosomal sialidase) (N-acetyl-, alpha-neuraminidase 1) (Acetylneuraminyl hydrolase) (G9 sialidase).]	Lysosomal
O14773	[Tripeptidyl-peptidase 1 precursor (EC 3.4.14.9) (Tripeptidyl-peptidase, I) (TPP-1) (TPP-I) (Tripeptidyl aminopeptidase) (Lysosomal pepstatin, insensitive protease) (LPIC) (Cell growth-inhibiting gene 1 protein).]	Lysosomal
P54803	[Galactocerebrosidase precursor (EC 3.2.1.46) (GALCERase), (Galactosylceramidase) (Galactosylceramide beta-galactosidase), (Galactocerebroside beta-galactosidase).]	Lysosomal
Q9UHG3	[Prenylcysteine oxidase 1 precursor (EC 1.8.3.5) (Prenylcysteine, lyase).]	Lysosomal
P05164	[Myeloperoxidase precursor (EC 1.11.1.7) (MPO) [Contains: 89 kDa, myeloperoxidase; 84 kDa myeloperoxidase; Myeloperoxidase light chain;, Myeloperoxidase heavy chain].]	Lysosomal

Q9UBX1	Cathepsin F precursor (EC 3.4.22.41) (CATSF).	Lysosomal
P98164	[Low-density lipoprotein receptor-related protein 2 precursor (Megalin), (Glycoprotein 330) (gp330).]	Lysosomal
P17900	[Ganglioside GM2 activator precursor (GM2-AP) (Cerebroside sulfate, activator protein) (Shingolipid activator protein 3) (SAP-3), [Contains: Ganglioside GM2 activator isoform short].]	Lysosomal
Q01085	Nucleolysin TIAR (TIA-1-related protein).	Lysosomal
P12236	[ADP/ATP translocase 3 (Adenine nucleotide translocator 2) (ANT 3), (ADP,ATP carrier protein 3) (Solute carrier family 25 member 6), (ADP,ATP carrier protein, isoform T2).]	Mitochondrial
P10809	[60 kDa heat shock protein, mitochondrial precursor (Hsp60) (60 kDa, chaperonin) (CPN60) (Heat shock protein 60) (HSP-60) (Mitochondrial, matrix protein P1) (P60 lymphocyte protein) (HuCHA60).]	Mitochondrial
O76031	[ATP-dependent Clp protease ATP-binding subunit clpX-like,, mitochondrial precursor.]	Mitochondrial
Q9Y6N1	Cytochrome c oxidase assembly protein COX11, mitochondrial precursor.	Mitochondrial
Q92523	[Carnitine O-palmitoyltransferase I, muscle isoform (EC 2.3.1.21) (CPT, I) (CPTI-M) (Carnitine palmitoyltransferase 1B) (Carnitine, palmitoyltransferase I-like protein).]	Mitochondrial
P21912	[Succinate dehydrogenase [ubiquinone] iron-sulfur subunit,, mitochondrial precursor (EC 1.3.5.1) (Ip) (Iron-sulfur subunit of, complex II).]	Mitochondrial
P38117	Electron transfer flavoprotein subunit beta (Beta-ETF).	Mitochondrial
P48728	[Aminomethyltransferase, mitochondrial precursor (EC 2.1.2.10) (Glycine, cleavage system T protein) (GCVT).]	Mitochondrial
Q9UI32	[Glutaminase liver isoform, mitochondrial precursor (EC 3.5.1.2) (GLS), (L-glutamine amidohydrolase) (L-glutaminase).]	Mitochondrial
P36969	[Phospholipid hydroperoxide glutathione peroxidase, mitochondrial, precursor (EC 1.11.1.12) (PHGPx) (GPX-4).]	Mitochondrial
P22557	[5-aminolevulinate synthase, erythroid-specific, mitochondrial, precursor (EC 2.3.1.37) (5-aminolevulinic acid synthase) (Delta-, aminolevulinate synthase) (Delta-ALA synthetase) (ALAS-E).]	Mitochondrial
P54868	[Hydroxymethylglutaryl-CoA synthase, mitochondrial precursor, (EC 2.3.3.10) (HMG-CoA synthase) (3-hydroxy-3-methylglutaryl coenzyme, A synthase).]	Mitochondrial
P12532	[Creatine kinase, ubiquitous mitochondrial precursor (EC 2.7.3.2) (U-, MtCK) (Mia-CK) (Acidic-type mitochondrial creatine kinase).]	Mitochondrial
Q02252	[Methylmalonate-semialdehyde dehydrogenase [acylating], mitochondrial, precursor (EC 1.2.1.27) (MMSDH) (Malonate-semialdehyde dehydrogenase, [acylating]) (EC 1.2.1.18).]	Mitochondrial
P22033	[Methylmalonyl-CoA mutase, mitochondrial precursor (EC 5.4.99.2) (MCM), (Methylmalonyl-CoA isomerase).]	Mitochondrial
P03923	[NADH-ubiquinone oxidoreductase chain 6 (EC 1.6.5.3) (NADH, dehydrogenase subunit 6).]	Mitochondrial
O00217	[NADH dehydrogenase [ubiquinone] iron-sulfur protein 8, mitochondrial, precursor (EC 1.6.5.3) (EC 1.6.99.3) (NADH-ubiquinone oxidoreductase, 23 kDa subunit) (Complex I-23kD) (CI-23kD) (TYKY subunit).]	Mitochondrial
P51970	[NADH dehydrogenase [ubiquinone] 1 alpha subcomplex subunit 8, (EC 1.6.5.3) (EC 1.6.99.3) (NADH-ubiquinone oxidoreductase 19 kDa, subunit) (Complex I-19kD) (CI-19kD) (Complex I-PGIV) (CI-PGIV).]	Mitochondrial
P49753	[Acyl-coenzyme A thioesterase 2, mitochondrial precursor (EC 3.1.2.2), (Acyl-CoA thioesterase 2) (Acyl-coenzyme A thioester hydrolase 2a), (Long-chain acyl-coA thioesterase 2) (ZAP128) (CTE-Ia).]	Mitochondrial
O00411	[DNA-directed RNA polymerase, mitochondrial precursor (EC 2.7.7.6), (MtRPOL).]	Mitochondrial
P82650	Mitochondrial 28S ribosomal protein S22 (S22mt) (MRP-S22).	Mitochondrial
P55851	Mitochondrial uncoupling protein 2 (UCP 2) (UCPH).	Mitochondrial
Q9Y4W6	AFG3-like protein 2 (EC 3.4.24.-) (Paraplegin-like protein).	Mitochondrial
P25705	ATP synthase subunit alpha, mitochondrial precursor (EC 3.6.3.14).	Mitochondrial

**P11586**	[C-1-tetrahydrofolate synthase, cytoplasmic (C1-THF synthase), [Includes: Methylenetetrahydrofolate dehydrogenase (EC 1.5.1.5);, Methenyltetrahydrofolate cyclohydrolase (EC 3.5.4.9);, Formyltetrahydrofolate synthetase (EC 6.3.4.3)].]	Mitochondrial
**P31327**	[Carbamoyl-phosphate synthase [ammonia], mitochondrial precursor, (EC 6.3.4.16) (Carbamoyl-phosphate synthetase I) (CPSase I).]	Mitochondrial
**P30084**	[Enoyl-CoA hydratase, mitochondrial precursor (EC 4.2.1.17) (Short, chain enoyl-CoA hydratase) (SCEH) (Enoyl-CoA hydratase 1).]	Mitochondrial
**P34897**	[Serine hydroxymethyltransferase, mitochondrial precursor (EC 2.1.2.1), (Serine methylase) (Glycine hydroxymethyltransferase) (SHMT).]	Mitochondrial
**Q16836**	[Hydroxyacyl-coenzyme A dehydrogenase, mitochondrial precursor, (EC 1.1.1.35) (Short chain 3-hydroxyacyl-CoA dehydrogenase) (HCDH), (Medium and short chain L-3-hydroxyacyl-coenzyme A dehydrogenase).]	Mitochondrial
**P36551**	[Coproporphyrinogen III oxidase, mitochondrial precursor (EC 1.3.3.3), (Coproporphyrinogenase) (Coprogen oxidase) (COX).]	Mitochondrial
**P52789**	[Hexokinase-2 (EC 2.7.1.1) (Hexokinase type II) (HK II) (Muscle form, hexokinase).]	Mitochondrial
**P46199**	[Translation initiation factor IF-2, mitochondrial precursor (IF-2Mt), (IF2(mt)) (IF-2(Mt)).]	Mitochondrial
**Q10713**	[Mitochondrial-processing peptidase alpha subunit, mitochondrial, precursor (EC 3.4.24.64) (Alpha-MPP) (P-55).]	Mitochondrial
**O75431**	Metaxin-2.	Mitochondrial
**O95182**	[NADH dehydrogenase [ubiquinone] 1 alpha subcomplex subunit 7, (EC 1.6.5.3) (EC 1.6.99.3) (NADH-ubiquinone oxidoreductase subunit, B14.5a) (Complex I-B14.5a) (CI-B14.5a).]	Mitochondrial
**O15239**	[NADH dehydrogenase [ubiquinone] 1 alpha subcomplex subunit 1, (EC 1.6.5.3) (EC 1.6.99.3) (NADH-ubiquinone oxidoreductase MWFE, subunit) (Complex I-MWFE) (CI-MWFE).]	Mitochondrial
**O75306**	[NADH dehydrogenase [ubiquinone] iron-sulfur protein 2, mitochondrial, precursor (EC 1.6.5.3) (EC 1.6.99.3) (NADH-ubiquinone oxidoreductase, 49 kDa subunit) (Complex I-49kD) (CI-49kD).]	Mitochondrial
**P04181**	[Ornithine aminotransferase, mitochondrial precursor (EC 2.6.1.13), (Ornithine--oxo-acid aminotransferase) [Contains: Ornithine, aminotransferase, hepatic form; Ornithine aminotransferase, renal, form].]	Mitochondrial
**Q9UQ90**	Paraplegin (EC 3.4.24.-) (Spastic paraplegia protein 7).	Mitochondrial
**P51649**	[Succinate semialdehyde dehydrogenase, mitochondrial precursor, (EC 1.2.1.24) (NAD(+)-dependent succinic semialdehyde dehydrogenase), (Aldehyde dehydrogenase family 5 member A1).]	Mitochondrial
**O95847**	[Mitochondrial uncoupling protein 4 (UCP 4) (Solute carrier family 25, member 27).]	Mitochondrial
**P45954**	[Short/branched chain specific acyl-CoA dehydrogenase, mitochondrial, precursor (EC 1.3.99.-) (SBCAD) (2-methyl branched chain acyl-CoA, dehydrogenase) (2-MEBCAD) (2-methylbutyryl-coenzyme A dehydrogenase), (2-methylbutyryl-CoA dehydrogenase).]	Mitochondrial
**Q99798**	[Aconitate hydratase, mitochondrial precursor (EC 4.2.1.3) (Citrate, hydro-lyase) (Aconitase).]	Mitochondrial
**Q9UHK6**	[Alpha-methylacyl-CoA racemase (EC 5.1.99.4) (2-methylacyl-CoA, racemase).]	Mitochondrial
**O14874**	[[3-methyl-2-oxobutanoate dehydrogenase [lipoamide]] kinase,, mitochondrial precursor (EC 2.7.11.4) (Branched-chain alpha-ketoacid, dehydrogenase kinase) (BCKDHKIN) (BCKD-kinase).]	Mitochondrial
**Q9Y305**	[Acyl-coenzyme A thioesterase 9 (EC 3.1.2.-) (Acyl-CoA thioesterase 9), (Acyl-CoA thioester hydrolase 9).]	Mitochondrial
**Q12887**	[Protoheme IX farnesyltransferase, mitochondrial precursor (EC 2.5.1.-), (Heme O synthase).]	Mitochondrial
**Q16854**	Deoxyguanosine kinase, mitochondrial precursor (EC 2.7.1.113) (dGK).	Mitochondrial
**P54098**	[DNA polymerase subunit gamma-1 (EC 2.7.7.7) (Mitochondrial DNA, polymerase catalytic subunit) (PolG-alpha).]	Mitochondrial
**O75879**	[Probable glutamyl-tRNA(Gln) amidotransferase subunit B, mitochondrial, precursor (EC 6.3.5.-) (Glu-ADT subunit B) (Cytochrome oxidase, assembly factor PET112 homolog).]	Mitochondrial
**Q92947**	[Glutaryl-CoA dehydrogenase, mitochondrial precursor (EC 1.3.99.7), (GCD).]	Mitochondrial
**P17540**	[Creatine kinase, sarcomeric mitochondrial precursor (EC 2.7.3.2) (S-, MtCK) (Mib-CK) (Basic-type mitochondrial creatine kinase).]	Mitochondrial
**P04049**	[RAF proto-oncogene serine/threonine-protein kinase (EC 2.7.11.1) (Raf-, 1) (C-RAF) (cRaf).]	Mitochondrial

O43678	[NADH dehydrogenase [ubiquinone] 1 alpha subcomplex subunit 2, (EC 1.6.5.3) (EC 1.6.99.3) (NADH-ubiquinone oxidoreductase B8 subunit), (Complex I-B8) (CI-B8).]	Mitochondrial
Q16822	[Phosphoenolpyruvate carboxykinase [GTP], mitochondrial precursor, (EC 4.1.1.32) (Phosphoenolpyruvate carboxylase) (PEPCK-M).]	Mitochondrial
O75570	Peptide chain release factor 1, mitochondrial precursor (MRF-1).	Mitochondrial
P82914	28S ribosomal protein S15, mitochondrial precursor (S15mt) (MRP-S15).	Mitochondrial
P22695	[Ubiquinol-cytochrome-c reductase complex core protein 2, mitochondrial, precursor (EC 1.10.2.2) (Core protein II) (Complex III subunit II).]	Mitochondrial
Q16352	[Alpha-internexin (Alpha-Inx) (66 kDa neurofilament protein), (Neurofilament-66) (NF-66).]	Neural
O43497	[Voltage-dependent T-type calcium channel subunit alpha-1G (Voltage-, gated calcium channel subunit alpha Cav3.1) (Cav3.1c) (NBR13).]	Neural
Q00535	[Cell division protein kinase 5 (EC 2.7.11.22) (Cyclin-dependent kinase, 5) (Tau protein kinase II catalytic subunit) (TPKII catalytic subunit), (Serine/threonine-protein kinase PSSALRE).]	Neural
Q9NYC9	Ciliary dynein heavy chain 9 (Axonemal beta dynein heavy chain 9).	Neural
P48549	[G protein-activated inward rectifier potassium channel 1 (GIRK1), (Potassium channel, inwardly rectifying subfamily J member 3) (Inward, rectifier K(+) channel Kir3.1).]	Neural
P21359	[Neurofibromin (Neurofibromatosis-related protein NF-1) [Contains:, Neurofibromin truncated].]	Neural
Q01668	[Voltage-dependent L-type calcium channel subunit alpha-1D (Voltage-, gated calcium channel subunit alpha Cav1.3) (Calcium channel, L type,, alpha-1 polypeptide, isoform 2).]	Neural
Q13698	[Voltage-dependent L-type calcium channel subunit alpha-1S (Voltage-, gated calcium channel subunit alpha Cav1.1) (Calcium channel, L type,, alpha-1 polypeptide, isoform 3, skeletal muscle).]	Neural
Q09470	[Potassium voltage-gated channel subfamily A member 1 (Voltage-gated, potassium channel subunit Kv1.1) (HUKI) (HBK1).]	Neural
P48048	[ATP-sensitive inward rectifier potassium channel 1 (Potassium channel,, inwardly rectifying subfamily J member 1) (ATP-regulated potassium, channel ROM-K) (Kir1.1).]	Neural
P12036	[Neurofilament heavy polypeptide (NF-H) (Neurofilament triplet H, protein) (200 kDa neurofilament protein).]	Neural
P07196	[Neurofilament light polypeptide (NF-L) (Neurofilament triplet L, protein) (68 kDa neurofilament protein).]	Neural
P07197	[Neurofilament medium polypeptide (NF-M) (Neurofilament triplet M, protein) (160 kDa neurofilament protein) (Neurofilament 3).]	Neural
Q00975	[Voltage-dependent N-type calcium channel subunit alpha-1B (Voltage-, gated calcium channel subunit alpha Cav2.2) (Calcium channel, L type,, alpha-1 polypeptide isoform 5) (Brain calcium channel III) (BIII).]	Neural
Q9Y698	[Voltage-dependent calcium channel gamma-2 subunit (Neuronal voltage-, gated calcium channel gamma-2 subunit).]	Neural
P54289	[Voltage-dependent calcium channel subunit alpha-2/delta-1 precursor, (Voltage-gated calcium channel subunit alpha-2/delta-1) [Contains:, Voltage-dependent calcium channel subunit alpha-2-1; Voltage-dependent, calcium channel subunit delta-1].]	Neural
O43525	[Potassium voltage-gated channel subfamily KQT member 3 (Voltage-gated, potassium channel subunit Kv7.3) (Potassium channel subunit alpha, KvLQT3) (KQT-like 3).]	Neural
Q9H2C0	Gigaxonin (Kelch-like protein 16).	Neural
O60928	[Inward rectifier potassium channel 13 (Potassium channel, inwardly, rectifying subfamily J member 13) (Inward rectifier K(+) channel, Kir7.1).]	Neural
P10636	[Microtubule-associated protein tau (Neurofibrillary tangle protein), (Paired helical filament-tau) (PHF-tau).]	Neural
P61812	[Transforming growth factor beta-2 precursor (TGF-beta-2), (Glioblastoma-derived T-cell suppressor factor) (G-TSF) (BSC-1 cell, growth inhibitor) (Polyergin) (Cetermin).]	Neural

**Q9Y6J0**	Calcineurin-binding protein Cabin 1 (Calcineurin inhibitor) (CAIN).	Nuclear
**P46087**	[Putative RNA methyltransferase NOL1 (EC 2.1.1.-) (Proliferating-cell, nucleolar antigen p120) (Proliferation-associated nucleolar protein, p120).]	Nuclear
**Q9Y6J0**	Calcineurin-binding protein Cabin 1 (Calcineurin inhibitor) (CAIN).	Nuclear
**P46087**	[Putative RNA methyltransferase NOL1 (EC 2.1.1.-) (Proliferating-cell, nucleolar antigen p120) (Proliferation-associated nucleolar protein, p120).]	Nuclear
**Q9Y2G3**	[Probable phospholipid-transporting ATPase IF (EC 3.6.3.1) (ATPase, class I type 11B) (ATPase IR).]	Nuclear
**Q09666**	[Neuroblast differentiation-associated protein AHNAK (Desmoyokin), (Fragments).]	Nuclear
**Q13023**	[A-kinase anchor protein 6 (Protein kinase A-anchoring protein 6), (PRKA6) (A-kinase anchor protein 100 kDa) (AKAP 100) (mAKAP).]	Nuclear
**P05549**	[Transcription factor AP-2 alpha (AP2-alpha) (Activating enhancer-, binding protein 2 alpha) (AP-2 transcription factor) (Activator, protein 2) (AP-2).]	Nuclear
**P56559**	[ADP-ribosylation factor-like protein 4C (ADP-ribosylation factor-like, protein 7) (ADP-ribosylation factor-like protein LAK).]	Nuclear
**Q9Y2T1**	[Axin-2 (Axis inhibition protein 2) (Conductin) (Axin-like protein), (Axil).]	Nuclear
**Q8IXQ6**	[Poly [ADP-ribose] polymerase 9 (EC 2.4.2.30) (PARP-9) (B aggressive, lymphoma protein).]	Nuclear
**Q9Y6J0**	Calcineurin-binding protein Cabin 1 (Calcineurin inhibitor) (CAIN).	Nuclear
**Q09161**	[Nuclear cap-binding protein subunit 1 (80 kDa nuclear cap-binding, protein) (NCBP 80 kDa subunit) (CBP80).]	Nuclear
**P14635**	G2/mitotic-specific cyclin-B1.	Nuclear
**Q12873**	[Chromodomain-helicase-DNA-binding protein 3 (EC 3.6.1.-) (ATP-, dependent helicase CHD3) (CHD-3) (Mi-2 autoantigen 240 kDa protein), (Mi2-alpha) (Zinc finger helicase) (hZFH).]	Nuclear
**Q9NYV4**	[Cell division cycle 2-related protein kinase 7 (EC 2.7.11.22) (CDC2-, related protein kinase 7) (Cdc2-related kinase, arginine/serine-rich), (CrkRS).]	Nuclear
**Q05048**	[Cleavage stimulation factor 50 kDa subunit (CSTF 50 kDa subunit) (CF-1, 50 kDa subunit) (CstF-50).]	Nuclear
**Q92879**	[CUG-BP- and ETR-3-like factor 1 (CELF-1) (Bruno-like protein 2) (RNA-, binding protein BRUNOL-2) (CUG triplet repeat RNA-binding protein 1), (CUG-BP1) (Deadenylation factor CUG-BP) (50 kDa nuclear polyadenylated, RNA-binding protein) (Embryo deadenylation element-binding protein, homolog) (EDEN-BP homolog).]	Nuclear
**O14529**	Homeobox protein cut-like 2 (Homeobox protein Cux-2) (Cut-like 2).	Nuclear
**O60231**	[Putative pre-mRNA-splicing factor ATP-dependent RNA helicase DHX16, (EC 3.6.1.-) (DEAH-box protein 16) (ATP-dependent RNA helicase #3).]	Nuclear
**Q14181**	DNA polymerase subunit alpha B (DNA polymerase alpha 70 kDa subunit).	Nuclear
**Q07864**	[DNA polymerase epsilon catalytic subunit A (EC 2.7.7.7) (DNA, polymerase II subunit A).]	Nuclear
**O43781**	[Dual specificity tyrosine-phosphorylation-regulated kinase 3, (EC 2.7.12.1) (Regulatory erythroid kinase) (REDK).]	Nuclear
**P19419**	ETS domain-containing protein Elk-1.	Nuclear
**P15408**	Fos-related antigen 2.	Nuclear
**Q16665**	[Hypoxia-inducible factor 1 alpha (HIF-1 alpha) (HIF1 alpha) (ARNT-, interacting protein) (Member of PAS protein 1) (Basic-helix-loop-, helix-PAS protein MOP1).]	Nuclear
**P41235**	[Hepatocyte nuclear factor 4-alpha (HNF-4-alpha) (Transcription factor, HNF-4) (Transcription factor 14).]	Nuclear
**Q03164**	Zinc finger protein HRX (ALL-1) (Trithorax-like protein).	Nuclear
**Q99081**	[Transcription factor 12 (Transcription factor HTF-4) (E-box-binding, protein) (DNA-binding protein HTF4).]	Nuclear

**P13010**	[ATP-dependent DNA helicase 2 subunit 2 (EC 3.6.1.-) (ATP-dependent DNA, helicase II 80 kDa subunit) (Lupus Ku autoantigen protein p86) (Ku86), (Ku80) (86 kDa subunit of Ku antigen) (Thyroid-lupus autoantigen), (TLAA) (CTC box-binding factor 85 kDa subunit) (CTCBF) (CTC85), (Nuclear factor IV) (DNA-repair protein XRCC5).]	Nuclear
**Q14739**	[Lamin-B receptor (Integral nuclear envelope inner membrane protein), (LMN2R).]	Nuclear
**Q9Y2U8**	Inner nuclear membrane protein Man1 (LEM domain-containing protein 3).	Nuclear
**P33991**	DNA replication licensing factor MCM4 (CDC21 homolog) (P1-CDC21).	Nuclear
**O15151**	[Mdm4 protein (p53-binding protein Mdm4) (Mdm2-like p53-binding, protein) (Mdmx protein) (Double minute 4 protein).]	Nuclear
**P49959**	[Double-strand break repair protein MRE11A (MRE11 homolog 1) (MRE11, meiotic recombination 11 homolog A).]	Nuclear
**Q9Y618**	[Nuclear receptor corepressor 2 (N-CoR2) (Silencing mediator of, retinoic acid and thyroid hormone receptor) (SMRT) (SMRTe) (Thyroid-,, retinoic-acid-receptor-associated corepressor) (T3 receptor-, associating factor) (TRAC) (CTG repeat protein 26) (SMAP270).]	Nuclear
**Q14995**	[Orphan nuclear receptor NR1D2 (Rev-erb-beta) (EAR-1R) (Orphan nuclear, hormone receptor BD73).]	Nuclear
**O43929**	Origin recognition complex subunit 4.	Nuclear
**Q09472**	[Histone acetyltransferase p300 (EC 2.3.1.48) (E1A-associated protein, p300).]	Nuclear
**P55771**	Paired box protein Pax-9.	Nuclear
**P54278**	PMS1 protein homolog 2 (DNA mismatch repair protein PMS2).	Nuclear
**P53041**	[Serine/threonine-protein phosphatase 5 (EC 3.1.3.16) (PP5) (Protein, phosphatase T) (PP-T) (PPT).]	Nuclear
**Q9HAZ2**	[PR domain zinc finger protein 16 (PR domain-containing protein 16), (Transcription factor MEL1).]	Nuclear
**Q00577**	[Transcriptional activator protein Pur-alpha (Purine-rich single-, stranded DNA-binding protein alpha).]	Nuclear
**Q13610**	[Periodic tryptophan protein 1 homolog (Keratinocyte protein IEF SSP, 9502).]	Nuclear
**P54727**	[UV excision repair protein RAD23 homolog B (hHR23B) (XP-C repair-, complementing complex 58 kDa protein) (p58).]	Nuclear
**P38159**	[Heterogeneous nuclear ribonucleoprotein G (hnRNP G) (RNA-binding motif, protein, X chromosome) (Glycoprotein p43) [Contains: Processed, heterogeneous nuclear ribonucleoprotein G].]	Nuclear
**O95602**	[DNA-directed RNA polymerase I subunit RPA1 (EC 2.7.7.6) (DNA-directed, RNA polymerase I largest subunit) (RNA polymerase I 194 kDa subunit), (RPA194) (A190).]	Nuclear
**P24928**	[DNA-directed RNA polymerase II subunit RPB1 (EC 2.7.7.6) (RNA, polymerase II subunit B1) (DNA-directed RNA polymerase II subunit A), (DNA-directed RNA polymerase III largest subunit).]	Nuclear
**P28702**	Retinoic acid receptor RXR-beta (Retinoid X receptor beta).	Nuclear
**Q16181**	Septin-7 (CDC10 protein homolog).	Nuclear
**O60315**	[Zinc finger E-box-binding homeobox 2 (Zinc finger homeobox protein 1b), (Smad-interacting protein 1) (SMADIP1).]	Nuclear
**P28370**	[Probable global transcription activator SNF2L1 (EC 3.6.1.-), (Nucleosome-remodeling factor subunit SNF2L) (ATP-dependent helicase, SMARCA1) (SWI/SNF-related matrix-associated actin-dependent regulator, of chromatin subfamily A member 1).]	Nuclear
**O00570**	SOX-1 protein.	Nuclear
**Q01082**	[Spectrin beta chain, brain 1 (Spectrin, non-erythroid beta chain 1), (Beta-II spectrin) (Fodrin beta chain).]	Nuclear
**O14981**	[TATA-binding protein-associated factor 172 (EC 3.6.1.-) (ATP-dependent, helicase BTAF1) (TBP-associated factor 172) (TAF-172) (TAF(II)170) (B-, TFIID transcription factor-associated 170 kDa subunit).]	Nuclear
**Q9UMN6**	[WW domain-binding protein 7 (Myeloid/lymphoid or mixed-lineage, leukemia protein 4) (Trithorax homolog 2).]	Nuclear
**P17480**	[Nucleolar transcription factor 1 (Upstream-binding factor 1) (UBF-1), (Autoantigen NOR-90).]	Nuclear
**P30291**	Wee1-like protein kinase (EC 2.7.10.2) (Wee1A kinase) (WEE1hu).	Nuclear

Q14191	Werner syndrome ATP-dependent helicase (EC 3.6.1.-).	Nuclear
P23025	[DNA-repair protein complementing XP-A cells (Xeroderma pigmentosum, group A-complementing protein).]	Nuclear
O14628	Zinc finger protein 195.	Nuclear
Q9Y2Q1	Zinc finger protein 257 (Bone marrow zinc finger 4) (BMZF-4).	Nuclear
Q9Y2X9	[Zinc finger protein 281 (Zinc finger DNA-binding protein 99), (Transcription factor ZBP-99) (GC-box-binding zinc finger protein 1).]	Nuclear
Q9Y5A6	[Zinc finger and SCAN domain-containing protein 21 (Zinc finger protein, 38 homolog) (Zfp-38) (Renal carcinoma antigen NY-REN-21).]	Nuclear
P17029	[Zinc finger protein with KRAB and SCAN domains 1 (Zinc finger protein, 36) (Zinc finger protein KOX18).]	Nuclear
P51523	Zinc finger protein 84 (Zinc finger protein HPF2).	Nuclear
P00519	[Proto-oncogene tyrosine-protein kinase ABL1 (EC 2.7.10.2) (p150) (c-, ABL) (Abelson murine leukemia viral oncogene homolog 1).]	Nuclear
P35869	Aryl hydrocarbon receptor precursor (Ah receptor) (AhR).	Nuclear
O95696	Bromodomain-containing protein 1 (BR140-like protein).	Nuclear
P52298	[Nuclear cap-binding protein subunit 2 (20 kDa nuclear cap-binding, protein) (NCBP 20 kDa subunit) (CBP20) (NCBP-interacting protein 1), (NIP1) (Cell proliferation-inducing gene 55 protein).]	Nuclear
O60583	Cyclin-T2 (CycT2).	Nuclear
P24864	G1/S-specific cyclin-E1.	Nuclear
P49711	[Transcriptional repressor CTCF (CCCTC-binding factor) (CTCFL paralog), (11-zinc finger protein).]	Nuclear
O60832	[H/ACA ribonucleoprotein complex subunit 4 (EC 5.4.99.-) (Dyskerin), (Nucleolar protein family A member 4) (snoRNP protein DKC1) (Nopp140-, associated protein of 57 kDa) (Nucleolar protein NAP57) (CBF5, homolog).]	Nuclear
Q92988	Homeobox protein DLX-4 (DLX-7) (DLX-8) (Beta protein 1).	Nuclear
P26358	[DNA (cytosine-5)-methyltransferase 1 (EC 2.1.1.37) (Dnmt1) (DNA, methyltransferase HsaI) (DNA MTase HsaI) (MCMT) (M.HsaI).]	Nuclear
P09884	DNA polymerase alpha catalytic subunit (EC 2.7.7.7).	Nuclear
Q9UGP5	[DNA polymerase lambda (EC 2.7.7.7) (EC 4.2.99.-) (Pol Lambda) (DNA, polymerase kappa) (DNA polymerase beta-2) (Pol beta2).]	Nuclear
Q99856	[AT-rich interactive domain-containing protein 3A (ARID domain-, containing protein 3A) (Dead ringer-like protein 1) (Bright) (E2F-, binding protein 1).]	Nuclear
Q13627	[Dual specificity tyrosine-phosphorylation-regulated kinase 1A, (EC 2.7.12.1) (Protein kinase minibrain homolog) (MNBH) (HP86) (Dual, specificity YAK1-related kinase) (hMNB).]	Nuclear
O14682	[Ectoderm-neural cortex protein 1 (ENC-1) (p53-induced gene 10 protein), (Nuclear matrix protein NRP/B).]	Nuclear
P41162	[ETS translocation variant 3 (ETS domain transcriptional repressor PE1), (PE-1) (Mitogenic Ets transcriptional suppressor).]	Nuclear
Q16676	[Forkhead box protein D1 (Forkhead-related protein FKHL8) (Forkhead-, related transcription factor 4) (FREAC-4).]	Nuclear
Q14161	[ARF GTPase-activating protein GIT2 (G protein-coupled receptor kinase-, interactor 2) (GRK-interacting protein 2) (Cool-interacting tyrosine-, phosphorylated protein 2) (CAT2) (CAT-2).]	Nuclear
Q9UGU5	High mobility group protein 2-like 1 (Protein HMGBCG).	Nuclear
Q9UBC0	Hepatocyte nuclear factor 6 (HNF-6) (One cut domain family member 1).	Nuclear
P31277	Homeobox protein Hox-D11 (Hox-4F).	Nuclear
Q15306	[Interferon regulatory factor 4 (IRF-4) (Lymphocyte-specific interferon, regulatory factor) (LSIRF) (NF-EM5) (Multiple myeloma oncogene 1).]	Nuclear
Q13118	[Krueppel-like factor 10 (Transforming growth factor-beta-inducible, early growth response protein 1) (TGFB-inducible early growth response, protein 1) (TIEG-1) (EGR-alpha).]	Nuclear

**P51826**	[AF4/FMR2 family member 3 (Protein LAF-4) (Lymphoid nuclear protein, related to AF4).]	Nuclear
**P10242**	Myb proto-oncogene protein (C-myb).	Nuclear
**Q01538**	[Myelin transcription factor 1 (MyT1) (MyTI) (Proteolipid protein-, binding protein) (PLPB1).]	Nuclear
**Q15596**	[Nuclear receptor coactivator 2 (NCoA-2) (Transcriptional intermediary, factor 2).]	Nuclear
**Q13823**	Nucleolar GTP-binding protein 2 (Autoantigen NGP-1).	Nuclear
**P46087**	[Putative RNA methyltransferase NOL1 (EC 2.1.1.-) (Proliferating-cell, nucleolar antigen p120) (Proliferation-associated nucleolar protein, p120).]	Nuclear
**O00482**	[Orphan nuclear receptor NR5A2 (Alpha-1-fetoprotein transcription, factor) (Hepatocytic transcription factor) (B1-binding factor) (hB1F), (CYP7A promoter-binding factor) (Liver receptor homolog 1) (LRH-1).]	Nuclear
**Q06518**	[Nitric oxide synthase, inducible (EC 1.14.13.39) (NOS type II), (Inducible NO synthase) (Inducible NOS) (iNOS).]	Nuclear
**P40424**	[Pre-B-cell leukemia transcription factor 1 (Homeobox protein PBX1), (Homeobox protein PRL).]	Nuclear
**Q01780**	[Exosome component 10 (Polymyositis/scleroderma autoantigen 2), (Autoantigen PM/Scl 2) (Polymyositis/scleroderma autoantigen 100 kDa), (PM/Scl-100) (P100 polymyositis-scleroderma overlap syndrome-, associated autoantigen).]	Nuclear
**Q07869**	Peroxisome proliferator-activated receptor alpha (PPAR-alpha).	Nuclear
**Q9HCU5**	[Prolactin regulatory element-binding protein (Mammalian guanine, nucleotide exchange factor mSec12).]	Nuclear
**P26599**	[Polypyrimidine tract-binding protein 1 (PTB) (Heterogeneous nuclear, ribonucleoprotein I) (hnRNP I) (57 kDa RNA-binding protein PPTB-1).]	Nuclear
**P43351**	DNA repair protein RAD52 homolog.	Nuclear
**Q9Y2P8**	RNA 3'-terminal phosphate cyclase-like protein.	Nuclear
**P48552**	[Nuclear receptor-interacting protein 1 (Nuclear factor RIP140), (Receptor-interacting protein 140).]	Nuclear
**P31943**	Heterogeneous nuclear ribonucleoprotein H (hnRNP H).	Nuclear
**Q00839**	[Heterogeneous nuclear ribonucleoprotein U (hnRNP U) (Scaffold, attachment factor A) (SAF-A) (p120) (pp120).]	Nuclear
**O14802**	[DNA-directed RNA polymerase III subunit RPC1 (EC 2.7.7.6) (RNA, polymerase III subunit C1) (DNA-directed RNA polymerase III subunit A), (DNA-directed RNA polymerase III largest subunit) (RPC155) (C160).]	Nuclear
**Q01130**	[Splicing factor, arginine/serine-rich 2 (Splicing factor SC35) (SC-35), (Splicing component, 35 kDa) (Protein PR264).]	Nuclear
**P51531**	[Probable global transcription activator SNF2L2 (EC 3.6.1.-) (ATP-, dependent helicase SMARCA2) (SNF2-alpha) (SWI/SNF-related matrix-, associated actin-dependent regulator of chromatin subfamily A member, 2) (hBRM).]	Nuclear
**P48436**	Transcription factor SOX-9.	Nuclear
**Q02447**	Transcription factor Sp3 (SPR-2).	Nuclear
**Q12962**	[Transcription initiation factor TFIID subunit 10 (Transcription, initiation factor TFIID 30 kDa subunit) (TAF(II)30) (TAFII-30), (TAFII30) (STAF28).]	Nuclear
**Q16890**	Tumor protein D53 (hD53) (Tumor protein D52-like 1).	Nuclear
**P11388**	[DNA topoisomerase 2-alpha (EC 5.99.1.3) (DNA topoisomerase II, alpha, isozyme).]	Nuclear
**Q15695**	[U2 small nuclear ribonucleoprotein auxiliary factor 35 kDa subunit-, related protein 1 (U2(RNU2) small nuclear RNA auxiliary factor 1-like, 1) (CCCH type zinc finger, RNA-binding motif and serine/arginine rich, protein 1).]	Nuclear
**P62988**	Ubiquitin.	Nuclear
**O60287**	Nucleolar preribosomal-associated protein 1 (Fragment).	Nuclear
**P52742**	Zinc finger protein 135 (Zinc finger protein 61).	Nuclear

Q05516	[Zinc finger and BTB domain-containing protein 16 (Zinc finger protein, PLZF) (Promyelocytic leukemia zinc finger protein) (Zinc finger, protein 145).]	Nuclear
P98182	Zinc finger protein 200.	Nuclear
Q9UL59	[Zinc finger protein 214 (BWSCR2-associated zinc finger protein 1) (BAZ, 1).]	Nuclear
Q9UL36	Zinc finger protein 236.	Nuclear
Q9UDV7	Zinc finger protein 282 (HTLV-I U5RE-binding protein 1) (HUB-1).	Nuclear
Q9Y2L8	[Zinc finger protein with KRAB and SCAN domains 5 (Zinc finger protein, 95 homolog) (Zfp-95).]	Nuclear
P17038	[Zinc finger protein 43 (Zinc protein HTF6) (Zinc finger protein, KOX27).]	Nuclear
P35789	[Zinc finger protein 93 (Zinc finger protein 505) (Zinc finger protein, HTF34).]	Nuclear
P98168	Zinc finger X-linked protein ZXDA.	Nuclear
O75478	Transcriptional adapter 2-like (ADA2-like protein).	Nuclear
Q01954	Zinc finger protein basonuclin-1.	Nuclear
Q9Y6J0	Calcineurin-binding protein Cabin 1 (Calcineurin inhibitor) (CAIN).	Nuclear
P11802	[Cell division protein kinase 4 (EC 2.7.11.22) (Cyclin-dependent kinase, 4) (PSK-J3).]	Nuclear
Q9ULW0	[Targeting protein for Xklp2 (Restricted expression proliferation-, associated protein 100) (p100) (Differentially expressed in cancerous, and noncancerous lung cells 2) (DIL-2) (Protein fls353), (Hepatocellular carcinoma-associated antigen 519).]	Nuclear
Q9UBC3	[DNA (cytosine-5)-methyltransferase 3B (EC 2.1.1.37) (Dnmt3b) (DNA, methyltransferase HsaIIIB) (DNA MTase HsaIIIB) (M.HsaIIIB).]	Nuclear
P49916	[DNA ligase 3 (EC 6.5.1.1) (DNA ligase III) (Polydeoxyribonucleotide, synthase [ATP] 3).]	Nuclear
O75417	DNA polymerase theta (EC 2.7.7.7) (DNA polymerase eta).	Nuclear
Q9Y463	[Dual specificity tyrosine-phosphorylation-regulated kinase 1B, (EC 2.7.12.1) (Mirk protein kinase) (Minibrain-related kinase).]	Nuclear
P43268	[ETS translocation variant 4 (Adenovirus E1A enhancer-binding protein), (E1A-F).]	Nuclear
P42685	[Tyrosine-protein kinase FRK (EC 2.7.10.2) (FYN-related kinase), (Nuclear tyrosine protein kinase RAK).]	Nuclear
O00358	[Forkhead box protein E1 (Thyroid transcription factor 2) (TTF-2), (Forkhead-related protein FKHL15).]	Nuclear
Q9UKS7	Zinc finger protein Helios (Ikaros family zinc finger protein 2).	Nuclear
P54198	Protein HIRA (TUP1-like enhancer of split protein 1).	Nuclear
Q16534	Hepatic leukemia factor.	Nuclear
Q13049	[Tripartite motif-containing protein 32 (EC 6.3.2.-) (Zinc finger, protein HT2A) (72 kDa Tat-interacting protein).]	Nuclear
Q16666	[Gamma-interferon-inducible protein Ifi-16 (Interferon-inducible, myeloid differentiation transcriptional activator) (IFI 16).]	Nuclear
Q15349	[Ribosomal protein S6 kinase alpha-2 (EC 2.7.11.1) (S6K-alpha 2) (90, kDa ribosomal protein S6 kinase 2) (p90-RSK 2) (Ribosomal S6 kinase 3), (RSK-3) (pp90RSK3) (MAP kinase-activated protein kinase 1c), (MAPKAPK1C).]	Nuclear
Q13342	[Nuclear body protein SP140 (Nuclear autoantigen Sp-140) (Speckled 140, kDa) (LYSp100 protein) (Lymphoid-restricted homolog of Sp100).]	Nuclear
P43243	Matrin-3.	Nuclear
P25205	[DNA replication licensing factor MCM3 (DNA polymerase alpha, holoenzyme-associated protein P1) (RLF subunit beta) (P102 protein), (P1-MCM3).]	Nuclear
Q00987	[E3 ubiquitin-protein ligase Mdm2 (EC 6.3.2.-) (p53-binding protein, Mdm2) (Oncoprotein Mdm2) (Double minute 2 protein) (Hdm2).]	Nuclear
Q92794	[Histone acetyltransferase MYST3 (EC 2.3.1.48) (EC 2.3.1.-) (MYST, protein 3) (MOZ, YBF2/SAS3, SAS2 and TIP60 protein 3) (Runt-related, transcription factor-binding protein 2) (Monocytic leukemia zinc, finger protein) (Zinc finger protein 220).]	Nuclear

**O15457**	MutS protein homolog 4.	Nuclear
**O60682**	Musculin (Activated B-cell factor 1) (ABF-1).	Nuclear
**P10243**	Myb-related protein A (A-Myb).	Nuclear
**O94916**	[Nuclear factor of activated T-cells 5 (T-cell transcription factor, NFAT5) (NF-AT5) (Tonicity-responsive enhancer-binding protein) (TonE-, binding protein) (TonEBP).]	Nuclear
**P46087**	[Putative RNA methyltransferase NOL1 (EC 2.1.1.-) (Proliferating-cell, nucleolar antigen p120) (Proliferation-associated nucleolar protein, p120).]	Nuclear
**Q15233**	[Non-POU domain-containing octamer-binding protein (NonO protein) (54, kDa nuclear RNA- and DNA-binding protein) (p54(nrb)) (p54nrb) (55 kDa, nuclear protein) (NMT55) (DNA-binding p52/p100 complex, 52 kDa, subunit).]	Nuclear
**Q13416**	Origin recognition complex subunit 2.	Nuclear
**P54277**	PMS1 protein homolog 1 (DNA mismatch repair protein PMS1).	Nuclear
**Q03181**	[Peroxisome proliferator-activated receptor delta (PPAR-delta) (PPAR-, beta) (Nuclear hormone receptor 1) (NUC1) (NUCI).]	Nuclear
**P09874**	[Poly [ADP-ribose] polymerase 1 (EC 2.4.2.30) (PARP-1) (ADPRT) (NAD(+), ADP-ribosyltransferase 1) (Poly[ADP-ribose] synthetase 1).]	Nuclear
**P17980**	[26S protease regulatory subunit 6A (Proteasome 26S subunit ATPase 3), (Tat-binding protein 1) (TBP-1) (Proteasome subunit P50).]	Nuclear
**P25786**	[Proteasome subunit alpha type-1 (EC 3.4.25.1) (Proteasome component, C2) (Macropain subunit C2) (Multicatalytic endopeptidase complex, subunit C2) (Proteasome nu chain) (30 kDa prosomal protein) (PROS-30).]	Nuclear
**Q06330**	[Recombining binding protein suppressor of hairless (J kappa-, recombination signal-binding protein) (RBP-J kappa) (RBP-J) (RBP-JK), (CBF-1) (Renal carcinoma antigen NY-REN-30).]	Nuclear
**P46063**	ATP-dependent DNA helicase Q1 (EC 3.6.1.-) (DNA-dependent ATPase Q1).	Nuclear
**P52597**	[Heterogeneous nuclear ribonucleoprotein F (hnRNP F) (Nucleolin-like, protein mcs94-1).]	Nuclear
**P55795**	Heterogeneous nuclear ribonucleoprotein H' (hnRNP H') (FTP-3).	Nuclear
**O15160**	[DNA-directed RNA polymerases I and III subunit RPAC1 (RNA polymerases, I and III subunit AC1) (DNA-directed RNA polymerase I subunit C) (DNA-, directed RNA polymerases I and III 40 kDa polypeptide) (RPA40) (RPA39), (RPC40) (AC40).]	Nuclear
**P12755**	Ski oncogene (C-ski).	Nuclear
**P51532**	[Probable global transcription activator SNF2L4 (EC 3.6.1.-) (ATP-, dependent helicase SMARCA4) (SNF2-beta) (BRG-1 protein) (Mitotic, growth and transcription activator) (Brahma protein homolog 1), (SWI/SNF-related matrix-associated actin-dependent regulator of, chromatin subfamily A member 4).]	Nuclear
**P08047**	Transcription factor Sp1.	Nuclear
**Q9H254**	[Spectrin beta chain, brain 3 (Spectrin, non-erythroid beta chain 3), (Beta-IV spectrin).]	Nuclear
**P11831**	Serum response factor (SRF).	Nuclear
**Q13569**	G/T mismatch-specific thymine DNA glycosylase (EC 3.2.2.-).	Nuclear
**P42166**	[Lamina-associated polypeptide 2 isoform alpha (Thymopoietin isoform, alpha) (TP alpha) (Thymopoietin-related peptide isoform alpha) (TPRP, isoform alpha) [Contains: Thymopoietin (TP) (Splenin); Thymopentin, (TP5)].]	Nuclear
**O95985**	[DNA topoisomerase 3-beta-1 (EC 5.99.1.2) (DNA topoisomerase III beta-, 1).]	Nuclear
**O75386**	Tubby-related protein 3 (Tubby-like protein 3).	Nuclear
**O94782**	[Ubiquitin carboxyl-terminal hydrolase 1 (EC 3.1.2.15) (Ubiquitin, thioesterase 1) (Ubiquitin-specific-processing protease 1), (Deubiquitinating enzyme 1) (hUBP).]	Nuclear
**Q9UMW8**	[Ubl carboxyl-terminal hydrolase 18 (EC 3.1.2.-) (Ubl thioesterase 18), (ISG15-specific-processing protease) (43 kDa ISG15-specific protease), (hUBP43).]	Nuclear

**Q9UNX4**	WD repeat-containing protein 3.	Nuclear
**Q9NSI6**	[Bromodomain and WD repeat-containing protein 1 (WD repeat-containing, protein 9).]	Nuclear
**Q15398**	[Disks large-associated protein DLG7 (Discs large homolog 7) (Hepatoma, up-regulated protein) (HURP).]	Nuclear
**P42695**	[Condensin-II complex subunit D3 (Non-SMC condensin II complex subunit, D3) (hCAP-D3).]	Nuclear
**Q9UDV6**	Zinc finger protein 212 (Zinc finger protein C2H2-150).	Nuclear
**Q9NRM2**	Zinc finger protein 277.	Nuclear
**P15822**	[Zinc finger protein 40 (Human immunodeficiency virus type I enhancer-, binding protein 1) (HIV-EP1) (Major histocompatibility complex-binding, protein 1) (MBP-1) (Positive regulatory domain II-binding factor 1), (PRDII-BF1).]	Nuclear
**P17098**	Zinc finger protein 8 (Zinc finger protein HF.18).	Nuclear
**P51815**	Zinc finger protein 75 (Zinc finger protein 82).	Nuclear
**P35658**	[Nuclear pore complex protein Nup214 (Nucleoporin Nup214) (214 kDa, nucleoporin) (CAN protein).]	Nuclear Membrane
**P49792**	[E3 SUMO-protein ligase RanBP2 (Ran-binding protein 2) (Nuclear pore, complex protein Nup358) (Nucleoporin Nup358) (358 kDa nucleoporin), (p270).]	Nuclear Membrane
**P37802**	Transgelin-2 (SM22-alpha homolog).	Nuclear Membrane
**P49790**	[Nuclear pore complex protein Nup153 (Nucleoporin Nup153) (153 kDa, nucleoporin).]	Nuclear Membrane
**P52594**	[Nucleoporin-like protein RIP (HIV-1 Rev-binding protein) (Rev-, interacting protein) (Rev/Rex activation domain-binding protein).]	Nuclear Membrane
**Q9H2T7**	Ran-binding protein 17.	Nuclear Membrane
**P35609**	[Alpha-actinin-2 (Alpha-actinin skeletal muscle isoform 2) (F-actin, cross-linking protein).]	Other Filament or Tubule
**P47756**	F-actin-capping protein subunit beta (CapZ beta).	Other Filament or Tubule
**P47755**	F-actin-capping protein subunit alpha-2 (CapZ alpha-2).	Other Filament or Tubule
**P30260**	Cell division cycle protein 27 homolog (CDC27Hs) (H-NUC).	Other Filament or Tubule
**P46940**	Ras GTPase-activating-like protein IQGAP1 (p195).	Other Filament or Tubule
**P08779**	[Keratin, type I cytoskeletal 16 (Cytokeratin-16) (CK-16) (Keratin-16), (K16).]	Other Filament or Tubule
**P04264**	[Keratin, type II cytoskeletal 1 (Cytokeratin-1) (CK-1) (Keratin-1), (K1) (67 kDa cytokeratin) (Hair alpha protein).]	Other Filament or Tubule
**P48668**	[Keratin, type II cytoskeletal 6C (Cytokeratin-6C) (CK 6C) (K6c, keratin) (Cytokeratin-6E) (CK 6E) (Keratin K6h).]	Other Filament or Tubule
**P53350**	[Serine/threonine-protein kinase PLK1 (EC 2.7.11.21) (Polo-like kinase, 1) (PLK-1) (Serine/threonine-protein kinase 13) (STPK13).]	Other Filament or Tubule
**Q08043**	[Alpha-actinin-3 (Alpha-actinin skeletal muscle isoform 3) (F-actin, cross-linking protein).]	Other Filament or Tubule
**P68133**	Actin, alpha skeletal muscle (Alpha-actin-1).	Other Filament or Tubule

Q99996	[A-kinase anchor protein 9 (Protein kinase A-anchoring protein 9), (PRKA9) (A-kinase anchor protein 450 kDa) (AKAP 450) (A-kinase anchor, protein 350 kDa) (AKAP 350) (hgAKAP 350) (AKAP 120-like protein), (Protein hyperion) (Protein yotiao) (Centrosome- and Golgi-localized, PKN-associated protein) (CG-NAP).]	Other Filament or Tubule
O43903	Growth-arrest-specific protein 2 (GAS-2).	Other Filament or Tubule
P13645	[Keratin, type I cytoskeletal 10 (Cytokeratin-10) (CK-10) (Keratin-10), (K10).]	Other Filament or Tubule
P12035	[Keratin, type II cytoskeletal 3 (Cytokeratin-3) (CK-3) (Keratin-3), (K3) (65 kDa cytokeratin).]	Other Filament or Tubule
P35908	[Keratin, type II cytoskeletal 2 epidermal (Cytokeratin-2e) (K2e) (CK, 2e) (keratin-2).]	Other Filament or Tubule
P48681	Nestin.	Other Filament or Tubule
Q13515	[Phakinin (Beaded filament structural protein 2) (Lens fiber cell, beaded filament protein CP 49) (CP49) (49 kDa cytoskeletal protein), (CP 47) (CP47) (Lens intermediate filament-like light) (LIFL-L).]	Other Filament or Tubule
P52907	F-actin-capping protein subunit alpha-1 (CapZ alpha-1).	Other Filament or Tubule
P15311	Ezrin (p81) (Cytovillin) (Villin-2).	Other Filament or Tubule
P35900	[Keratin, type I cytoskeletal 20 (Cytokeratin-20) (CK-20) (Keratin-20), (K20) (Protein IT).]	Other Filament or Tubule
Q01546	[Keratin, type II cytoskeletal 2 oral (Cytokeratin-2P) (K2P) (CK 2P), (Keratin-76).]	Other Filament or Tubule
P13647	[Keratin, type II cytoskeletal 5 (Cytokeratin-5) (CK-5) (Keratin-5), (K5) (58 kDa cytokeratin).]	Other Filament or Tubule
Q9UJT1	Tubulin delta chain (Delta-tubulin).	Other Filament or Tubule
P05408	[Neuroendocrine protein 7B2 precursor (Secretogranin-5) (Secretogranin, V) (Secretory granule endocrine protein I) (Pituitary polypeptide), [Contains: N-terminal peptide; C-terminal peptide].]	Other Go Component
P30926	Neuronal acetylcholine receptor subunit beta-4 precursor.	Other Go Component
P10323	[Acrosin precursor (EC 3.4.21.10) [Contains: Acrosin light chain;, Acrosin heavy chain].]	Other Go Component
O43684	Mitotic checkpoint protein BUB3.	Other Go Component
P20908	Collagen alpha-1(V) chain precursor.	Other Go Component
P12107	Collagen alpha-1(XI) chain precursor.	Other Go Component
Q07092	Collagen alpha-1(XVI) chain precursor.	Other Go Component
P08572	Collagen alpha-2(IV) chain precursor [Contains: Canstatin].	Other Go Component
P13942	Collagen alpha-2(XI) chain precursor.	Other Go Component
Q14031	Collagen alpha-6(IV) chain precursor.	Other Go Component
P08575	[Leukocyte common antigen precursor (EC 3.1.3.48) (L-CA) (T200) (CD45, antigen).]	Other Go Component
Q15517	Corneodesmosin precursor (S protein).	Other Go Component

O96017	Serine/threonine-protein kinase Chk2 (EC 2.7.11.1) (Cds1).	Other Go Component
P57739	Claudin-2 (SP82).	Other Go Component
P32927	[Cytokine receptor common beta chain precursor (GM-CSF/IL-3/IL-5, receptor common beta-chain) (CD131 antigen) (CDw131).]	Other Go Component
Q14562	[ATP-dependent RNA helicase DHX8 (EC 3.6.1.-) (DEAH box protein 8) (RNA, helicase HRH1).]	Other Go Component
P11532	Dystrophin.	Other Go Component
P05198	[Eukaryotic translation initiation factor 2 subunit 1 (Eukaryotic, translation initiation factor 2 subunit alpha) (eIF-2-alpha) (EIF-, 2alpha) (EIF-2A).]	Other Go Component
Q14573	[Inositol 1,4,5-trisphosphate receptor type 3 (Type 3 inositol 1,4,5-, trisphosphate receptor) (Type 3 InsP3 receptor) (IP3 receptor isoform, 3) (InsP3R3).]	Other Go Component
Q13557	[Calcium/calmodulin-dependent protein kinase type II delta chain, (EC 2.7.11.17) (CaM-kinase II delta chain) (CaM kinase II subunit, delta) (CaMK-II subunit delta).]	Other Go Component
P55268	Laminin subunit beta-2 precursor (S-laminin) (Laminin B1s chain).	Other Go Component
P23490	Loricrin.	Other Go Component
Q9HCI5	[Melanoma-associated antigen E1 (MAGE-E1 antigen) (Hepatocellular, carcinoma-associated protein 1).]	Other Go Component
P09012	[U1 small nuclear ribonucleoprotein A (U1 snRNP protein A) (U1A, protein) (U1-A).]	Other Go Component
Q12874	[Splicing factor 3A subunit 3 (Spliceosome-associated protein 61) (SAP, 61) (SF3a60).]	Other Go Component
P23497	[Nuclear autoantigen Sp-100 (Speckled 100 kDa) (Nuclear dot-associated, Sp100 protein) (Lysp100b).]	Other Go Component
P02549	Spectrin alpha chain, erythrocyte (Erythroid alpha-spectrin).	Other Go Component
Q15654	[Thyroid receptor-interacting protein 6 (TRIP-6) (OPA-interacting, protein 1) (Zyxin-related protein 1) (ZRP-1).]	Other Go Component
O75643	[U5 small nuclear ribonucleoprotein 200 kDa helicase (EC 3.6.1.-) (U5, snRNP-specific 200 kDa protein) (U5-200KD) (Activating signal, cointegrator 1 complex subunit 3-like 1) (BRR2 homolog).]	Other Go Component
P61421	[Vacuolar ATP synthase subunit d 1 (EC 3.6.3.14) (V-ATPase subunit d 1), (Vacuolar proton pump subunit d 1) (V-ATPase AC39 subunit) (V-ATPase, 40 kDa accessory protein) (P39) (32 kDa accessory protein).]	Other Go Component
P18206	Vinculin (Metavinculin).	Other Go Component
Q07001	Acetylcholine receptor subunit delta precursor.	Other Go Component
P19021	[Peptidyl-glycine alpha-amidating monooxygenase precursor (PAM), [Includes: Peptidylglycine alpha-hydroxylating monooxygenase, (EC 1.14.17.3) (PHM); Peptidyl-alpha-hydroxyglycine alpha-amidating, lyase (EC 4.3.2.5) (Peptidylamidoglycolate lyase) (PAL)].]	Other Go Component
P02461	Collagen alpha-1(III) chain precursor.	Other Go Component
P12109	Collagen alpha-1(VI) chain precursor.	Other Go Component
Q99715	Collagen alpha-1(XII) chain precursor.	Other Go Component
P39060	Collagen alpha-1(XVIII) chain precursor [Contains: Endostatin].	Other Go Component
P05997	Collagen alpha-2(V) chain precursor.	Other Go Component
Q01955	[Collagen alpha-3(IV) chain precursor (Goodpasture antigen) [Contains:, Tumstatin].]	Other Go Component
Q9NZU7	Calcium-binding protein 1 (CaBP1) (Calbrain).	Other Go Component
O00555	[Voltage-dependent P/Q-type calcium channel subunit alpha-1A (Voltage-, gated calcium channel subunit alpha Cav2.1) (Calcium channel, L type,, alpha-1 polypeptide isoform 4) (Brain calcium channel I) (BI).]	Other Go Component
P56747	Claudin-6 (Skullin 2).	Other Go Component
P56856	Claudin-18.	Other Go Component
P98082	Disabled homolog 2 (Differentially-expressed protein 2) (DOC-2).	Other Go Component

Q92817	[Envoplakin (210 kDa paraneoplastic pemphigus antigen) (p210) (210 kDa, cornified envelope precursor protein).]	Other Go Component
O75955	Flotillin-1.	Other Go Component
Q9Y2I7	[FYVE finger-containing phosphoinositide kinase (EC 2.7.1.68) (1-, phosphatidylinositol-4-phosphate 5-kinase) (Phosphatidylinositol-3-, phosphate 5-kinase type III) (PIP5K) (PtdIns(4)P-5-kinase) (PIKfyve), (p235).]	Other Go Component
P12544	[Granzyme A precursor (EC 3.4.21.78) (Cytotoxic T-lymphocyte proteinase, 1) (Hanukkah factor) (H factor) (HF) (Granzyme-1) (CTL tryptase), (Fragmentin-1).]	Other Go Component
P07476	Involucrin.	Other Go Component
P51884	[Lumican precursor (Keratan sulfate proteoglycan lumican) (KSPG, lumican).]	Other Go Component
O00255	Menin.	Other Go Component
P51153	Ras-related protein Rab-13 (Cell growth-inhibiting gene 4 protein).	Other Go Component
P09661	U2 small nuclear ribonucleoprotein A' (U2 snRNP-A').	Other Go Component
O75533	[Splicing factor 3B subunit 1 (Spliceosome-associated protein 155) (SAP, 155) (SF3b155) (Pre-mRNA-splicing factor SF3b 155 kDa subunit).]	Other Go Component
O15020	[Spectrin beta chain, brain 2 (Spectrin, non-erythroid beta chain 2), (Beta-III spectrin).]	Other Go Component
P12814	[Alpha-actinin-1 (Alpha-actinin cytoskeletal isoform) (Non-muscle, alpha-actinin-1) (F-actin cross-linking protein).]	Other Go Component
P43681	Neuronal acetylcholine receptor subunit alpha-4 precursor.	Other Go Component
Q05901	Neuronal acetylcholine receptor subunit beta-3 precursor.	Other Go Component
O43683	[Mitotic checkpoint serine/threonine-protein kinase BUB1 (EC 2.7.11.1), (hBUB1) (BUB1A).]	Other Go Component
P39059	[Collagen alpha-1(XV) chain precursor [Contains: Endostatin, (Endostatin-XV) (Restin) (Related to endostatin)].]	Other Go Component
P12111	Collagen alpha-3(VI) chain precursor.	Other Go Component
P15088	[Mast cell carboxypeptidase A precursor (EC 3.4.17.1) (MC-CPA), (Carboxypeptidase A3).]	Other Go Component
P56746	Claudin-15.	Other Go Component
P01040	Cystatin-A (Stefin-A) (Cystatin-AS).	Other Go Component
P15924	Desmoplakin (DP) (250/210 kDa paraneoplastic pemphigus antigen).	Other Go Component
P09172	[Dopamine beta-hydroxylase precursor (EC 1.14.17.1) (Dopamine beta-, monooxygenase).]	Other Go Component
Q16643	Drebrin (Developmentally-regulated brain protein).	Other Go Component
Q02413	[Desmoglein-1 precursor (Desmosomal glycoprotein 1) (DG1) (DGI), (Pemphigus foliaceus antigen).]	Other Go Component
P01880	Ig delta chain C region.	Other Go Component
Q9UI46	[Dynein intermediate chain 1, axonemal (Axonemal dynein intermediate, chain 1).]	Other Go Component
Q9P0M6	Core histone macro-H2A.2 (Histone macroH2A2) (mH2A2).	Other Go Component
P06239	[Proto-oncogene tyrosine-protein kinase LCK (EC 2.7.10.2) (p56-LCK), (Lymphocyte cell-specific protein-tyrosine kinase) (LSK) (T cell-, specific protein-tyrosine kinase).]	Other Go Component
Q16363	Laminin subunit alpha-4 precursor.	Other Go Component
Q05586	[Glutamate [NMDA] receptor subunit zeta-1 precursor (N-methyl-D-, aspartate receptor subunit NR1).]	Other Go Component
Q92730	[Rho-related GTP-binding protein Rho6 precursor (Rho family GTPase 1), (Rnd1).]	Other Go Component
Q15459	[Splicing factor 3 subunit 1 (Spliceosome-associated protein 114) (SAP, 114) (SF3a120).]	Other Go Component
P00441	Superoxide dismutase [Cu-Zn] (EC 1.15.1.1).	Other Go Component
P14410	[Sucrase-isomaltase, intestinal [Contains: Sucrase (EC 3.2.1.48);, Isomaltase (EC 3.2.1.10)].]	Other Go Component

Q15696	[U2 small nuclear ribonucleoprotein auxiliary factor 35 kDa subunit-, related protein 2 (U2(RNU2) small nuclear RNA auxiliary factor 1-like, 2) (CCCH type zinc finger, RNA-binding motif and serine/arginine rich, protein 2) (Renal carcinoma antigen NY-REN-20).]	Other Go Component
Q9ULB5	Cadherin-7 precursor.	Other Membrane
P12830	[Epithelial-cadherin precursor (E-cadherin) (Uvomorulin) (Cadherin-1), (CAM 120/80) (CD324 antigen) [Contains: E-Cad/CTF1; E-Cad/CTF2; E-, Cad/CTF3].]	Other Membrane
O15259	Nephrocystin-1 (Juvenile nephronophthisis 1 protein).	Other Membrane
P26439	[3 beta-hydroxysteroid dehydrogenase/Delta 5-->4-isomerase type II (3-, beta-HSD II) [Includes: 3-beta-hydroxy-Delta(5)-steroid dehydrogenase, (EC 1.1.1.145) (3-beta-hydroxy-5-ene steroid dehydrogenase), (Progesterone reductase); Steroid Delta-isomerase (EC 5.3.3.1) (Delta-, 5-3-ketosteroid isomerase)].]	Other Membrane
Q99758	[ATP-binding cassette sub-family A member 3 (ATP-binding cassette, transporter 3) (ATP-binding cassette 3) (ABC-C transporter).]	Other Membrane
Q9P0K1	[ADAM 22 precursor (A disintegrin and metalloproteinase domain 22), (Metalloproteinase-like, disintegrin-like, and cysteine-rich protein, 2) (Metalloproteinase-disintegrin ADAM22-3).]	Other Membrane
P51693	Amyloid-like protein 1 precursor (APLP) (APLP-1) [Contains: C30].	Other Membrane
Q10588	[ADP-ribosyl cyclase 2 precursor (EC 3.2.2.5) (Cyclic ADP-ribose, hydrolase 2) (cADPr hydrolase 2) (Bone marrow stromal antigen 1) (BST-, 1) (CD157 antigen).]	Other Membrane
Q9Y6N8	Cadherin-10 precursor (T2-cadherin).	Other Membrane
Q9H159	Cadherin-19 precursor.	Other Membrane
Q00973	[Beta-1,4 N-acetylgalactosaminyltransferase 1 (EC 2.4.1.92) ((N-, acetylneuraminyl)-galactosylglucosylceramide) (GM2/GD2 synthase), (GalNAc-T).]	Other Membrane
Q9ULX7	[Carbonic anhydrase 14 precursor (EC 4.2.1.1) (Carbonic anhydrase XIV), (Carbonate dehydratase XIV) (CA-XIV).]	Other Membrane
Q13936	[Voltage-dependent L-type calcium channel subunit alpha-1C (Voltage-, gated calcium channel subunit alpha Cav1.2) (Calcium channel, L type,, alpha-1 polypeptide, isoform 1, cardiac muscle).]	Other Membrane
P13987	[CD59 glycoprotein precursor (Membrane attack complex inhibition, factor) (MACIF) (MAC-inhibitory protein) (MAC-IP) (Protectin) (MEM43, antigen) (Membrane inhibitor of reactive lysis) (MIRL) (20 kDa, homologous restriction factor) (HRF-20) (HRF20) (1F5 antigen).]	Other Membrane
Q9Y5E1	Protocadherin beta 9 precursor (PCDH-beta9) (Protocadherin 3H).	Other Membrane
Q9Y5E9	Protocadherin beta 14 precursor (PCDH-beta14).	Other Membrane
P10643	Complement component C7 precursor.	Other Membrane
Q12860	[Contactin-1 precursor (Neural cell surface protein F3) (Glycoprotein, gp135).]	Other Membrane
Q9NYJ7	Delta-like protein 3 precursor (Drosophila Delta homolog 3).	Other Membrane
P43003	[Excitatory amino acid transporter 1 (Solute carrier family 1 member 3), (Sodium-dependent glutamate/aspartate transporter 1) (GLAST-1).]	Other Membrane
P01859	Ig gamma-2 chain C region.	Other Membrane
P04233	[HLA class II histocompatibility antigen gamma chain (HLA-DR antigens-, associated invariant chain) (Ia antigen-associated invariant chain), (Ii) (p33) (CD74 antigen).]	Other Membrane
P15814	[Immunoglobulin lambda-like polypeptide 1 precursor (Immunoglobulin-, related protein 14.1) (Immunoglobulin omega polypeptide) (Ig lambda-5), (CD179b antigen).]	Other Membrane
Q92504	[Zinc transporter SLC39A7 (Solute carrier family 39 member 7), (Histidine-rich membrane protein Ke4).]	Other Membrane
P08922	[Proto-oncogene tyrosine-protein kinase ROS precursor (EC 2.7.10.1) (c-, ros-1).]	Other Membrane
P24043	[Laminin subunit alpha-2 precursor (Laminin M chain) (Merosin heavy, chain).]	Other Membrane

**P53985**	[Monocarboxylate transporter 1 (MCT 1) (Solute carrier family 16 member, 1).]	Other Membrane
**P01871**	Ig mu chain C region.	Other Membrane
**P13591**	[Neural cell adhesion molecule 1, 140 kDa isoform precursor (N-CAM 140), (NCAM-140) (CD56 antigen).]	Other Membrane
**Q14112**	Nidogen-2 precursor (NID-2) (Osteonidogen).	Other Membrane
**P29475**	[Nitric-oxide synthase, brain (EC 1.14.13.39) (NOS type I) (Neuronal, NOS) (N-NOS) (nNOS) (Constitutive NOS) (NC-NOS) (bNOS).]	Other Membrane
**O60462**	[Neuropilin-2 precursor (Vascular endothelial cell growth factor 165, receptor 2).]	Other Membrane
**P46531**	[Neurogenic locus notch homolog protein 1 precursor (Notch 1) (hN1), (Translocation-associated notch protein TAN-1) [Contains: Notch 1, extracellular truncation; Notch 1 intracellular domain].]	Other Membrane
**P43657**	[P2Y purinoceptor 5 (P2Y5) (Purinergic receptor 5) (RB intron encoded, G-protein coupled receptor).]	Other Membrane
**O75381**	[Peroxisomal membrane protein PEX14 (Peroxin-14) (Peroxisomal membrane, anchor protein PEX14) (PTS1 receptor docking protein).]	Other Membrane
**P13686**	[Tartrate-resistant acid phosphatase type 5 precursor (EC 3.1.3.2) (TR-, AP) (Tartrate-resistant acid ATPase) (TrATPase) (Acid phosphatase 5,, tartrate resistant).]	Other Membrane
**P29350**	[Tyrosine-protein phosphatase non-receptor type 6 (EC 3.1.3.48), (Protein-tyrosine phosphatase 1C) (PTP-1C) (Hematopoietic cell, protein-tyrosine phosphatase) (SH-PTP1) (Protein-tyrosine phosphatase, SHP-1).]	Other Membrane
**Q15286**	Ras-related protein Rab-35 (Rab-1C) (GTP-binding protein RAY).	Other Membrane
**O95487**	Protein transport protein Sec24B (SEC24-related protein B).	Other Membrane
**Q9NRC6**	[Spectrin beta chain, brain 4 (Spectrin, non-erythroid beta chain 4), (Beta-V spectrin) (BSPECV).]	Other Membrane
**P08240**	[Signal recognition particle receptor subunit alpha (SR-alpha) (Docking, protein alpha) (DP-alpha).]	Other Membrane
**P56180**	[Putative tyrosine-protein phosphatase TPTE (EC 3.1.3.48), (Transmembrane phosphatase with tensin homology) (Protein BJ-HCC-5).]	Other Membrane
**Q9UPY5**	[Cystine/glutamate transporter (Amino acid transport system xc-) (xCT), (Calcium channel blocker resistance protein CCBR1).]	Other Membrane
**Q9Y2Q0**	[Probable phospholipid-transporting ATPase IA (EC 3.6.3.1) (Chromaffin, granule ATPase II) (ATPase class I type 8A member 1).]	Other Membrane
**P28288**	[ATP-binding cassette sub-family D member 3 (70 kDa peroxisomal, membrane protein) (PMP70).]	Other Membrane
**P12821**	[Angiotensin-converting enzyme, somatic isoform precursor (EC 3.4.15.1), (Dipeptidyl carboxypeptidase I) (Kininase II) (CD143 antigen), [Contains: Angiotensin-converting enzyme, somatic isoform, soluble, form].]	Other Membrane
**Q9GZZ6**	[Neuronal acetylcholine receptor subunit alpha-10 precursor (Nicotinic, acetylcholine receptor subunit alpha 10) (NACHR alpha 10).]	Other Membrane
**Q9UKF2**	[ADAM 30 precursor (EC 3.4.24.-) (A disintegrin and metalloproteinase, domain 30).]	Other Membrane
**P01877**	Ig alpha-2 chain C region.	Other Membrane
**Q06481**	[Amyloid-like protein 2 precursor (Amyloid protein homolog) (APPH), (CDEI box-binding protein) (CDEBP).]	Other Membrane
**P04920**	[Anion exchange protein 2 (Non-erythroid band 3-like protein) (AE2, anion exchanger) (Solute carrier family 4 member 2) (BND3L).]	Other Membrane
**P32247**	Bombesin receptor subtype-3 (BRS-3).	Other Membrane
**P55287**	Cadherin-11 precursor (Osteoblast-cadherin) (OB-cadherin) (OSF-4).	Other Membrane
**Q9HBT6**	Cadherin-20 precursor.	Other Membrane
**Q03135**	Caveolin-1.	Other Membrane
**Q9Y5E4**	Protocadherin beta 5 precursor (PCDH-beta5).	Other Membrane
**Q9UN67**	Protocadherin beta 10 precursor (PCDH-beta10).	Other Membrane
**O95069**	[Potassium channel subfamily K member 2 (Outward rectifying potassium, channel protein TREK-1) (TREK-1 K(+) channel subunit) (Two pore, potassium channel TPKC1) (Two pore domain potassium channel TREK-1).]	Other Membrane

P26992	Ciliary neurotrophic factor receptor alpha precursor (CNTFR alpha).	Other Membrane
P09603	[Macrophage colony-stimulating factor 1 precursor (CSF-1) (MCSF) (M-, CSF) (Lanimostim) [Contains: Processed macrophage colony-stimulating, factor 1].]	Other Membrane
O00341	[Excitatory amino acid transporter 5 (Solute carrier family 1 member 7), (Retinal glutamate transporter).]	Other Membrane
P42345	[FKBP12-rapamycin complex-associated protein (FK506-binding protein 12-, rapamycin complex-associated protein 1) (Rapamycin target protein), (RAPT1) (Mammalian target of rapamycin) (mTOR).]	Other Membrane
P23945	[Follicle-stimulating hormone receptor precursor (FSH-R) (Follitropin, receptor).]	Other Membrane
Q99928	[Gamma-aminobutyric-acid receptor subunit gamma-3 precursor (GABA(A), receptor subunit gamma-3).]	Other Membrane
P01860	Ig gamma-3 chain C region (Heavy chain disease protein) (HDC).	Other Membrane
P26572	[Alpha-1,3-mannosyl-glycoprotein 2-beta-N-acetylglucosaminyltransferase, (EC 2.4.1.101) (N-glycosyl-oligosaccharide-glycoprotein N-, acetylglucosaminyltransferase I) (GNT-I) (GlcNAc-T I).]	Other Membrane
P52849	[Bifunctional heparan sulfate N-deacetylase/N-sulfotransferase 2, (EC 2.8.2.8) (Glucosaminyl N-deacetylase/N-sulfotransferase 2) (NDST-, 2) (N-heparan sulfate sulfotransferase 2) (N-HSST 2) [Includes:, Heparan sulfate N-deacetylase 2 (EC 3.-.-.-); Heparan sulfate N-, sulfotransferase 2 (EC 2.8.2.-)].]	Other Membrane
Q16787	[Laminin subunit alpha-3 precursor (Epiligrin 170 kDa subunit) (E170), (Nicein subunit alpha).]	Other Membrane
P35410	Mas-related G-protein coupled receptor MRG (MAS-R) (MAS1-like).	Other Membrane
P04220	Ig mu heavy chain disease protein (BOT).	Other Membrane
P19634	[Sodium/hydrogen exchanger 1 (Na(+)/H(+) exchanger 1) (NHE-1) (Solute, carrier family 9 member 1) (Na(+)/H(+) antiporter, amiloride-, sensitive) (APNH).]	Other Membrane
Q9Y5P0	Olfactory receptor 51B4 (Odorant receptor HOR5'beta1).	Other Membrane
P51805	Plexin-A3 precursor (Plexin-4) (Semaphorin receptor SEX).	Other Membrane
Q15437	Protein transport protein Sec23B (SEC23-related protein B).	Other Membrane
Q02223	[Tumor necrosis factor receptor superfamily member 17 (B-cell, maturation protein) (CD269 antigen).]	Other Membrane
Q13061	Triadin.	Other Membrane
Q16864	[Vacuolar ATP synthase subunit F (EC 3.6.3.14) (V-ATPase subunit F), (Vacuolar proton pump subunit F) (V-ATPase 14 kDa subunit).]	Other Membrane
O76024	Wolframin.	Other Membrane
O14524	Uncharacterized membrane protein KIAA0286.	Other Membrane
Q9P241	Probable phospholipid-transporting ATPase VD (EC 3.6.3.1) (ATPVD).	Other Membrane
P78363	[Retinal-specific ATP-binding cassette transporter (ATP-binding, cassette sub-family A member 4) (RIM ABC transporter) (RIM protein), (RmP) (Stargardt disease protein).]	Other Membrane
O14983	[Sarcoplasmic/endoplasmic reticulum calcium ATPase 1 (EC 3.6.3.8), (Calcium pump 1) (SERCA1) (SR Ca(2+)-ATPase 1) (Calcium-transporting, ATPase sarcoplasmic reticulum type, fast twitch skeletal muscle, isoform) (Endoplasmic reticulum class 1/2 Ca(2+) ATPase).]	Other Membrane
O60242	Brain-specific angiogenesis inhibitor 3 precursor.	Other Membrane
Q03001	[Bullous pemphigoid antigen 1, isoforms 1/2/3/4/5/8 (230 kDa bullous, pemphigoid antigen) (BPA) (Hemidesmosomal plaque protein) (Dystonia, musculorum protein) (Dystonin) (Fragment).]	Other Membrane
O15165	Uncharacterized protein C18orf1.	Other Membrane
Q02388	[Collagen alpha-1(VII) chain precursor (Long-chain collagen) (LC, collagen).]	Other Membrane

**P33151**	[Cadherin-5 precursor (Vascular endothelial-cadherin) (VE-cadherin), (7B4 antigen) (CD144 antigen).]	Other Membrane
**Q9ULB4**	Cadherin-9 precursor.	Other Membrane
**P55289**	[Cadherin-12 precursor (Brain-cadherin) (BR-cadherin) (N-cadherin 2), (Neural type cadherin 2).]	Other Membrane
**Q9H251**	Cadherin-23 precursor (Otocadherin).	Other Membrane
**O60840**	[Voltage-dependent L-type calcium channel subunit alpha-1F (Voltage-, gated calcium channel subunit alpha Cav1.4).]	Other Membrane
**Q9Y5E2**	Protocadherin beta 7 precursor (PCDH-beta7).	Other Membrane
**O95674**	[Phosphatidate cytidylyltransferase 2 (EC 2.7.7.41) (CDP-diglyceride, synthetase 2) (CDP-diglyceride pyrophosphorylase 2) (CDP-, diacylglycerol synthase 2) (CDS 2) (CTP:phosphatidate, cytidylyltransferase 2) (CDP-DAG synthase 2) (CDP-DG synthetase 2).]	Other Membrane
**O75746**	[Calcium-binding mitochondrial carrier protein Aralar1 (Mitochondrial, aspartate glutamate carrier 1) (Solute carrier family 25 member 12).]	Other Membrane
**P07358**	[Complement component C8 beta chain precursor (Complement component 8, subunit beta).]	Other Membrane
**P81408**	Protein COTE1.	Other Membrane
**P51843**	[Nuclear receptor 0B1 (Nuclear receptor DAX-1) (DSS-AHC critical region, on the X chromosome protein 1).]	Other Membrane
**Q99848**	[Probable rRNA-processing protein EBP2 (EBNA1-binding protein 2), (Nucleolar protein p40).]	Other Membrane
**P35555**	Fibrillin-1 precursor.	Other Membrane
**P01857**	Ig gamma-1 chain C region.	Other Membrane
**P01861**	Ig gamma-4 chain C region.	Other Membrane
**P48146**	Neuropeptides B/W receptor type 2 (G-protein coupled receptor 8).	Other Membrane
**O95528**	[Solute carrier family 2, facilitated glucose transporter member 10, (Glucose transporter type 10) (GLUT-10).]	Other Membrane
**P11166**	[Solute carrier family 2, facilitated glucose transporter member 1, (Glucose transporter type 1, erythrocyte/brain) (GLUT-1) (HepG2, glucose transporter).]	Other Membrane
**Q9H3N8**	[Histamine H4 receptor (HH4R) (GPRv53) (G-protein coupled receptor 105), (SP9144) (AXOR35).]	Other Membrane
**P52790**	Hexokinase-3 (EC 2.7.1.1) (Hexokinase type III) (HK III).	Other Membrane
**P32019**	[Type II inositol-1,4,5-trisphosphate 5-phosphatase precursor, (EC 3.1.3.36) (Phosphoinositide 5-phosphatase) (5PTase) (75 kDa, inositol polyphosphate-5-phosphatase).]	Other Membrane
**Q14773**	[Intercellular adhesion molecule 4 precursor (ICAM-4) (Landsteiner-, Wiener blood group glycoprotein) (LW blood group protein) (CD242, antigen).]	Other Membrane
**Q14954**	[Killer cell immunoglobulin-like receptor 2DS1 precursor (MHC class I, NK cell receptor Eb6 ActI) (CD158h antigen).]	Other Membrane
**P17252**	Protein kinase C alpha type (EC 2.7.11.13) (PKC-alpha) (PKC-A).	Other Membrane
**P12956**	[ATP-dependent DNA helicase 2 subunit 1 (ATP-dependent DNA helicase II, 70 kDa subunit) (Lupus Ku autoantigen protein p70) (Ku70) (70 kDa, subunit of Ku antigen) (Thyroid-lupus autoantigen) (TLAA) (CTC box-, binding factor 75 kDa subunit) (CTCBF) (CTC75) (DNA-repair protein, XRCC6).]	Other Membrane
**Q16873**	[Leukotriene C4 synthase (EC 4.4.1.20) (Leukotriene-C(4) synthase), (LTC4 synthase).]	Other Membrane
**Q13491**	Neuronal membrane glycoprotein M6-b (M6b).	Other Membrane
**Q15049**	Membrane protein MLC1.	Other Membrane
**Q9NZW5**	MAGUK p55 subfamily member 6 (Veli-associated MAGUK 1) (VAM-1).	Other Membrane
**Q93070**	[Ecto-ADP-ribosyltransferase 4 precursor (EC 2.4.2.31) (NAD(P)(+)--, arginine ADP-ribosyltransferase 4) (Mono(ADP-ribosyl)transferase 4), (Dombrock blood group carrier molecule) (CD297 antigen).]	Other Membrane
**O15394**	Neural cell adhesion molecule 2 precursor (N-CAM 2).	Other Membrane

Q9Y585	[Olfactory receptor 1A2 (Olfactory receptor OR17-10) (Olfactory, receptor 17-6) (OR17-6).]	Other Membrane
P29728	[2'-5'-oligoadenylate synthetase 2 (EC 2.7.7.-) ((2-5')oligo(A), synthetase 2) (2-5A synthetase 2) (p69 OAS / p71 OAS) (p69OAS /, p71OAS).]	Other Membrane
P09131	P3 protein (Solute carrier family 10 member 3).	Other Membrane
Q13258	Prostaglandin D2 receptor (Prostanoid DP receptor) (PGD receptor).	Other Membrane
Q9UBV2	Sel-1 homolog precursor (Suppressor of lin-12-like protein) (Sel-1L).	Other Membrane
Q13813	[Spectrin alpha chain, brain (Spectrin, non-erythroid alpha chain), (Alpha-II spectrin) (Fodrin alpha chain).]	Other Membrane
Q9UKR8	[Tetraspanin-16 (Tspan-16) (Transmembrane 4 superfamily member 16), (Tetraspanin TM4-B).]	Other Membrane
Q9UKP6	Urotensin II receptor (UR-II-R) (G-protein coupled receptor 14).	Other Membrane
P54219	[Chromaffin granule amine transporter (Vesicular amine transporter 1), (VAT1) (Solute carrier family 18 member 1).]	Other Membrane
P55808	Glycoprotein Xg precursor (Protein PBDX).	Other Membrane
P49447	Cytochrome b561 (Cytochrome b-561).	Plasma Membrane
P21453	[Sphingosine 1-phosphate receptor Edg-1 (Sphingosine 1-phosphate, receptor 1) (S1P1).]	Plasma Membrane
Q13368	MAGUK p55 subfamily member 3 (Protein MPP3) (Discs large homolog 3).	Plasma Membrane
P20648	[Potassium-transporting ATPase alpha chain 1 (EC 3.6.3.10) (Proton, pump) (Gastric H(+)/K(+) ATPase subunit alpha).]	Plasma Membrane
P21453	[Sphingosine 1-phosphate receptor Edg-1 (Sphingosine 1-phosphate, receptor 1) (S1P1).]	Plasma Membrane
Q14542	[Equilibrative nucleoside transporter 2 (Equilibrative, nitrobenzylmercaptopurine riboside-insensitive nucleoside transporter), (Equilibrative NBMPR-insensitive nucleoside transporter) (Nucleoside, transporter, ei-type) (Solute carrier family 29 member 2) (36 kDa, nucleolar protein HNP36) (Hydrophobic nucleolar protein, 36 kDa), (Delayed-early response protein 12).]	Plasma Membrane
O15552	Free fatty acid receptor 2 (G-protein coupled receptor 43).	Plasma Membrane
P41595	5-hydroxytryptamine 2B receptor (5-HT-2B) (Serotonin receptor 2B).	Plasma Membrane
P11230	Acetylcholine receptor subunit beta precursor.	Plasma Membrane
P08172	Muscarinic acetylcholine receptor M2.	Plasma Membrane
Q99965	[ADAM 2 precursor (A disintegrin and metalloproteinase domain 2), (Fertilin subunit beta) (PH-30) (PH30).]	Plasma Membrane
Q07075	[Glutamyl aminopeptidase (EC 3.4.11.7) (EAP) (Aminopeptidase A) (APA), (Differentiation antigen gp160) (CD249 antigen).]	Plasma Membrane
P20594	[Atrial natriuretic peptide receptor B precursor (ANP-B) (ANPRB) (GC-B), (Guanylate cyclase B) (EC 4.6.1.2) (NPR-B) (Atrial natriuretic peptide, B-type receptor).]	Plasma Membrane
O76027	Annexin A9 (Annexin-9) (Annexin-31) (Annexin XXXI) (Pemphaxin).	Plasma Membrane
P35414	[Apelin receptor (G-protein coupled receptor APJ) (Angiotensin, receptor-like 1) (HG11).]	Plasma Membrane
Q04656	[Copper-transporting ATPase 1 (EC 3.6.3.4) (Copper pump 1) (Menkes, disease-associated protein).]	Plasma Membrane
Q9UN42	[X/potassium-transporting ATPase subunit beta-m (X,K-ATPase beta-m, subunit).]	Plasma Membrane
Q16515	[Amiloride-sensitive cation channel 1, neuronal (Amiloride-sensitive, brain sodium channel) (Amiloride-sensitive cation channel neuronal 1), (Acid-sensing ion channel 2) (ASIC2) (Brain sodium channel 1) (BNaC1), (BNC1) (Mammalian degenerin homolog).]	Plasma Membrane
P17213	Bactericidal permeability-increasing protein precursor (BPI) (CAP 57).	Plasma Membrane
P48509	[CD151 antigen (Platelet-endothelial tetraspan antigen 3) (PETA-3), (GP27) (Membrane glycoprotein SFA-1) (Tetraspanin-24) (Tspan-24).]	Plasma Membrane
Q16581	C3a anaphylatoxin chemotactic receptor (C3a-R) (C3AR).	Plasma Membrane

**P16870**	[Carboxypeptidase E precursor (EC 3.4.17.10) (CPE) (Carboxypeptidase H), (CPH) (Enkephalin convertase) (Prohormone-processing, carboxypeptidase).]	Plasma Membrane
**Q9UBN1**	[Voltage-dependent calcium channel gamma-4 subunit (Neuronal voltage-, gated calcium channel gamma-4 subunit).]	Plasma Membrane
**P51677**	[C-C chemokine receptor type 3 (C-C CKR-3) (CC-CKR-3) (CCR-3) (CCR3), (CKR3) (Eosinophil eotaxin receptor) (CD193 antigen).]	Plasma Membrane
**P06126**	[T-cell surface glycoprotein CD1a precursor (CD1a antigen) (T-cell, surface antigen T6/Leu-6) (hTa1 thymocyte antigen).]	Plasma Membrane
**Q01151**	[CD83 antigen precursor (Cell surface protein HB15) (B-cell activation, protein).]	Plasma Membrane
**Q9Y5H9**	Protocadherin alpha 2 precursor (PCDH-alpha2).	Plasma Membrane
**Q9Y5H7**	Protocadherin alpha 5 precursor (PCDH-alpha5).	Plasma Membrane
**P13569**	[Cystic fibrosis transmembrane conductance regulator (CFTR) (cAMP-, dependent chloride channel) (ATP-binding cassette transporter sub-, family C member 7).]	Plasma Membrane
**P51801**	Chloride channel protein ClC-Kb (Chloride channel Kb) (ClC-K2).	Plasma Membrane
**Q99250**	[Sodium channel protein type 2 subunit alpha (Sodium channel protein, type II subunit alpha) (Voltage-gated sodium channel subunit alpha, Nav1.2) (Sodium channel protein, brain II subunit alpha) (HBSC II).]	Plasma Membrane
**O95279**	[Potassium channel subfamily K member 5 (Acid-sensitive potassium, channel protein TASK-2) (TWIK-related acid-sensitive K(+) channel 2).]	Plasma Membrane
**O00590**	[Chemokine-binding protein 2 (Chemokine-binding protein D6) (C-C, chemokine receptor D6) (Chemokine receptor CCR-9) (Chemokine receptor, CCR-10).]	Plasma Membrane
**P51686**	[C-C chemokine receptor type 9 (C-C CKR-9) (CC-CKR-9) (CCR-9) (GPR-9-6), (G-protein coupled receptor 28) (CDw199 antigen).]	Plasma Membrane
**Q9UJS0**	[Calcium-binding mitochondrial carrier protein Aralar2 (Mitochondrial, aspartate glutamate carrier 2) (Solute carrier family 25 member 13), (Citrin).]	Plasma Membrane
**Q9NYX4**	D1 dopamine receptor-interacting protein calcyon.	Plasma Membrane
**P21918**	[D(1B) dopamine receptor (D(5) dopamine receptor) (D1beta dopamine, receptor).]	Plasma Membrane
**Q14574**	Desmocollin-3 precursor (Desmocollin-4) (HT-CP).	Plasma Membrane
**Q14126**	Desmoglein-2 precursor (HDGC).	Plasma Membrane
**Q16570**	[Duffy antigen/chemokine receptor (Fy glycoprotein) (GpFy), (Glycoprotein D) (Plasmodium vivax receptor) (CD234 antigen).]	Plasma Membrane
**P17813**	Endoglin precursor (CD105 antigen).	Plasma Membrane
**Q14246**	[EGF-like module-containing mucin-like hormone receptor-like 1, precursor (Cell surface glycoprotein EMR1) (EMR1 hormone receptor).]	Plasma Membrane
**P29317**	[Ephrin type-A receptor 2 precursor (EC 2.7.10.1) (Tyrosine-protein, kinase receptor ECK) (Epithelial cell kinase).]	Plasma Membrane
**P54753**	[Ephrin type-B receptor 3 precursor (EC 2.7.10.1) (Tyrosine-protein, kinase receptor HEK-2) (Tyrosine-protein kinase TYRO6).]	Plasma Membrane
**Q15303**	[Receptor tyrosine-protein kinase erbB-4 precursor (EC 2.7.10.1), (p180erbB4) (Tyrosine kinase-type cell surface receptor HER4).]	Plasma Membrane
**Q9Y256**	[CAAX prenyl protease 2 (EC 3.4.22.-) (Prenyl protein-specific, endoprotease 2) (Farnesylated proteins-converting enzyme 2) (FACE-2), (hRCE1).]	Plasma Membrane
**Q14517**	[Cadherin-related tumor suppressor homolog precursor (Protein fat, homolog).]	Plasma Membrane
**Q01362**	[High affinity immunoglobulin epsilon receptor subunit beta (FcERI), (IgE Fc receptor subunit beta) (Fc epsilon receptor I beta-chain), (Membrane-spanning 4-domains subfamily A member 2).]	Plasma Membrane
**P14867**	[Gamma-aminobutyric-acid receptor subunit alpha-1 precursor (GABA(A), receptor subunit alpha-1).]	Plasma Membrane
**P18507**	[Gamma-aminobutyric-acid receptor subunit gamma-2 precursor (GABA(A), receptor subunit gamma-2).]	Plasma Membrane
**P24046**	[Gamma-aminobutyric-acid receptor subunit rho-1 precursor (GABA(A), receptor subunit rho-1).]	Plasma Membrane

**O75899**	[Gamma-aminobutyric acid type B receptor subunit 2 precursor (GABA-B, receptor 2) (GABA-B-R2) (Gb2) (GABABR2) (G-protein coupled receptor, 51) (HG20).]	Plasma Membrane
**Q13003**	[Glutamate receptor, ionotropic kainate 3 precursor (Glutamate receptor, 7) (GluR-7) (GluR7) (Excitatory amino acid receptor 5) (EAA5).]	Plasma Membrane
**P42261**	[Glutamate receptor 1 precursor (GluR-1) (GluR-A) (GluR-K1) (Glutamate, receptor ionotropic, AMPA 1) (AMPA-selective glutamate receptor 1).]	Plasma Membrane
**Q13972**	[Guanine nucleotide-releasing protein (GNRP) (Ras-specific nucleotide, exchange factor CDC25) (Ras-specific guanine nucleotide-releasing, factor).]	Plasma Membrane
**O14842**	Free fatty acid receptor 1 (G-protein coupled receptor 40).	Plasma Membrane
**P78333**	Glypican-5 precursor.	Plasma Membrane
**P46089**	Probable G-protein coupled receptor 3 (ACCA orphan receptor).	Plasma Membrane
**P40197**	Platelet glycoprotein V precursor (GPV) (CD42D antigen).	Plasma Membrane
**P11168**	[Solute carrier family 2, facilitated glucose transporter member 2, (Glucose transporter type 2, liver) (GLUT-2).]	Plasma Membrane
**P14778**	[Interleukin-1 receptor type I precursor (IL-1R-1) (IL-1RT1) (IL-1R-, alpha) (p80) (CD121a antigen).]	Plasma Membrane
**P40189**	[Interleukin-6 receptor subunit beta precursor (IL-6R-beta), (Interleukin-6 signal transducer) (Membrane glycoprotein 130) (gp130), (Oncostatin-M receptor alpha subunit) (CD130 antigen) (CDw130).]	Plasma Membrane
**P26006**	[Integrin alpha-3 precursor (Galactoprotein B3) (GAPB3) (VLA-3 alpha, chain) (FRP-2) (CD49c antigen) [Contains: Integrin alpha-3 heavy, chain; Integrin alpha-3 light chain].]	Plasma Membrane
**P08514**	[Integrin alpha-IIb precursor (Platelet membrane glycoprotein IIb), (GPalpha IIb) (GPIIb) (CD41 antigen) [Contains: Integrin alpha-IIb, heavy chain; Integrin alpha-IIb light chain, form 1; Integrin alpha-, IIb light chain, form 2].]	Plasma Membrane
**Q9UKX5**	Integrin alpha-11 precursor.	Plasma Membrane
**P20702**	[Integrin alpha-X precursor (Leukocyte adhesion glycoprotein p150,95, alpha chain) (Leukocyte adhesion receptor p150,95) (Leu M5) (CD11c, antigen).]	Plasma Membrane
**P16144**	Integrin beta-4 precursor (GP150) (CD104 antigen).	Plasma Membrane
**P43632**	[Killer cell immunoglobulin-like receptor 2DS4 precursor (MHC class I, NK cell receptor) (Natural killer-associated transcript 8) (NKAT-8), (P58 natural killer cell receptor clone CL-39) (p58 NK receptor) (CL-, 17) (CD158i antigen).]	Plasma Membrane
**P07333**	[Macrophage colony-stimulating factor 1 receptor precursor, (EC 2.7.10.1) (CSF-1-R) (Fms proto-oncogene) (c-fms) (CD115 antigen).]	Plasma Membrane
**P29376**	[Leukocyte tyrosine kinase receptor precursor (EC 2.7.10.1) (Protein, tyrosine kinase 1).]	Plasma Membrane
**P11279**	[Lysosome-associated membrane glycoprotein 1 precursor (LAMP-1) (CD107a, antigen).]	Plasma Membrane
**P16581**	[E-selectin precursor (Endothelial leukocyte adhesion molecule 1), (ELAM-1) (Leukocyte-endothelial cell adhesion molecule 2) (LECAM2), (CD62E antigen).]	Plasma Membrane
**P22897**	Macrophage mannose receptor 1 precursor (MMR) (CD206 antigen).	Plasma Membrane
**Q13255**	Metabotropic glutamate receptor 1 precursor (mGluR1).	Plasma Membrane
**O00222**	Metabotropic glutamate receptor 8 precursor (mGluR8).	Plasma Membrane
**O15438**	[Canalicular multispecific organic anion transporter 2 (ATP-binding, cassette sub-family C member 3) (Multidrug resistance-associated, protein 3) (Multi-specific organic anion transporter-D) (MOAT-D).]	Plasma Membrane
**Q01726**	[Melanocyte-stimulating hormone receptor (MSH-R) (Melanotropin, receptor) (Melanocortin receptor 1) (MC1-R).]	Plasma Membrane
**Q9UBC5**	[Myosin-Ia (Brush border myosin I) (BBM-I) (BBMI) (Myosin I heavy, chain) (MIHC).]	Plasma Membrane

**Q9Y5X5**	[Neuropeptide FF receptor 2 (Neuropeptide G-protein coupled receptor), (G-protein coupled receptor 74) (G-protein coupled receptor HLWAR77).]	Plasma Membrane
**P26715**	[NKG2-A/NKG2-B type II integral membrane protein (NKG2-A/B-activating, NK receptor) (NK cell receptor A) (CD159a antigen).]	Plasma Membrane
**Q13224**	[Glutamate [NMDA] receptor subunit epsilon-2 precursor (N-methyl D-, aspartate receptor subtype 2B) (NR2B) (NMDAR2B) (N-methyl-D-aspartate, receptor subunit 3) (NR3) (hNR3).]	Plasma Membrane
**Q14916**	[Sodium-dependent phosphate transport protein 1 (Sodium/phosphate, cotransporter 1) (Na(+)/PI cotransporter 1) (Solute carrier family 17, member 1) (Renal sodium-dependent phosphate transport protein 1), (Renal sodium-phosphate transport protein 1) (Renal Na(+)-dependent, phosphate cotransporter 1) (Na/Pi-4).]	Plasma Membrane
**P49279**	Natural resistance-associated macrophage protein 1 (NRAMP 1).	Plasma Membrane
**Q9Y4C0**	Neurexin-3-alpha precursor (Neurexin III-alpha).	Plasma Membrane
**O43613**	Orexin receptor type 1 (Ox1r) (Hypocretin receptor type 1).	Plasma Membrane
**P43115**	[Prostaglandin E2 receptor EP3 subtype (Prostanoid EP3 receptor) (PGE, receptor, EP3 subtype) (PGE2-R).]	Plasma Membrane
**P78562**	[Phosphate-regulating neutral endopeptidase (EC 3.4.24.-), (Metalloendopeptidase homolog PEX) (X-linked hypophosphatemia protein), (HYP) (Vitamin D-resistant hypophosphatemic rickets protein).]	Plasma Membrane
**P98161**	[Polycystin-1 precursor (Autosomal dominant polycystic kidney disease, protein 1).]	Plasma Membrane
**P23467**	[Receptor-type tyrosine-protein phosphatase beta precursor, (EC 3.1.3.48) (Protein-tyrosine phosphatase beta) (R-PTP-beta).]	Plasma Membrane
**P23470**	[Receptor-type tyrosine-protein phosphatase gamma precursor, (EC 3.1.3.48) (Protein-tyrosine phosphatase gamma) (R-PTP-gamma).]	Plasma Membrane
**Q16849**	[Receptor-type tyrosine-protein phosphatase-like N precursor (R-PTP-N), (PTP IA-2) (Islet cell antigen 512) (ICA 512) (Islet cell autoantigen, 3).]	Plasma Membrane
**P23471**	[Receptor-type tyrosine-protein phosphatase zeta precursor, (EC 3.1.3.48) (R-PTP-zeta).]	Plasma Membrane
**Q08116**	[Regulator of G-protein signaling 1 (RGS1) (Early response protein, 1R20) (B-cell activation protein BL34).]	Plasma Membrane
**Q03395**	[Rod outer segment membrane protein 1 (ROSP1) (Tetraspanin-23) (Tspan-, 23).]	Plasma Membrane
**P29353**	[SHC-transforming protein 1 (SH2 domain protein C1) (Src homology 2, domain-containing-transforming protein C1).]	Plasma Membrane
**P32745**	Somatostatin receptor type 3 (SS3R) (SSR-28).	Plasma Membrane
**P36897**	[TGF-beta receptor type-1 precursor (EC 2.7.11.30) (TGF-beta receptor, type I) (TGFR-1) (TGF-beta type I receptor) (Transforming growth, factor-beta receptor type I) (TbetaR-I) (Serine/threonine-protein, kinase receptor R4) (SKR4) (Activin receptor-like kinase 5) (ALK-5).]	Plasma Membrane
**Q9UKU6**	[Thyrotropin-releasing hormone-degrading ectoenzyme (EC 3.4.19.6) (TRH-, degrading ectoenzyme) (TRH-DE) (TRH-specific aminopeptidase), (Thyroliberinase) (Pyroglutamyl-peptidase II) (PAP-II).]	Plasma Membrane
**P35590**	Tyrosine-protein kinase receptor Tie-1 precursor (EC 2.7.10.1).	Plasma Membrane
**P04629**	[High affinity nerve growth factor receptor precursor (EC 2.7.10.1), (Neurotrophic tyrosine kinase receptor type 1) (TRK1-transforming, tyrosine kinase protein) (p140-TrkA) (Trk-A).]	Plasma Membrane
**Q12816**	Trophinin (MAGE-D3 antigen).	Plasma Membrane
**Q9Y210**	Short transient receptor potential channel 6 (TrpC6).	Plasma Membrane
**P01737**	T-cell receptor alpha chain V region PY14 precursor.	Plasma Membrane
**Q05940**	[Synaptic vesicular amine transporter (Monoamine transporter), (Vesicular amine transporter 2) (VAT2) (Solute carrier family 18, member 2).]	Plasma Membrane
**O15078**	Centrosomal protein Cep290 (Nephrocystin-6) (Tumor antigen se2-2).	Plasma Membrane
**P28221**	[5-hydroxytryptamine 1D receptor (5-HT-1D) (Serotonin receptor 1D) (5-, HT-1D-alpha).]	Plasma Membrane

P25100	[Alpha-1D adrenergic receptor (Alpha 1D-adrenoceptor) (Alpha 1D-, adrenoreceptor) (Alpha-1A adrenergic receptor) (Alpha-adrenergic, receptor 1a).]	Plasma Membrane
P05067	[Amyloid beta A4 protein precursor (APP) (ABPP) (Alzheimer disease, amyloid protein) (Cerebral vascular amyloid peptide) (CVAP) (Protease, nexin-II) (PN-II) (APPI) (PreA4) [Contains: Soluble APP-alpha (S-APP-, alpha); Soluble APP-beta (S-APP-beta); C99; Beta-amyloid protein 42, (Beta-APP42); Beta-amyloid protein 40 (Beta-APP40); C83; P3(42);, P3(40); Gamma-CTF(59) (Gamma-secretase C-terminal fragment 59), (Amyloid intracellular domain 59) (AID(59)) (AICD-59); Gamma-CTF(57), (Gamma-secretase C-terminal fragment 57) (Amyloid intracellular domain, 57) (AID(57)) (AICD-57); Gamma-CTF(50) (Gamma-secretase C-terminal, fragment 50) (Amyloid intracellular domain 50) (AID(50)) (AICD-50);, C31].]	Plasma Membrane
P29275	Adenosine A2b receptor.	Plasma Membrane
O95477	[ATP-binding cassette sub-family A member 1 (ATP-binding cassette, transporter 1) (ATP-binding cassette 1) (ABC-1) (Cholesterol efflux, regulatory protein).]	Plasma Membrane
P78325	[ADAM 8 precursor (EC 3.4.24.-) (A disintegrin and metalloproteinase, domain 8) (Cell surface antigen MS2) (CD156a antigen) (CD156).]	Plasma Membrane
P35670	[Copper-transporting ATPase 2 (EC 3.6.3.4) (Copper pump 2) (Wilson, disease-associated protein) [Contains: WND/140 kDa].]	Plasma Membrane
P23634	[Plasma membrane calcium-transporting ATPase 4 (EC 3.6.3.8) (PMCA4), (Plasma membrane calcium pump isoform 4) (Plasma membrane calcium, ATPase isoform 4) (Matrix-remodeling-associated protein 1).]	Plasma Membrane
Q02246	[Contactin-2 precursor (Axonin-1) (Axonal glycoprotein TAG-1), (Transient axonal glycoprotein 1) (TAX-1).]	Plasma Membrane
P55291	Cadherin-15 precursor (Muscle-cadherin) (M-cadherin) (Cadherin-14).	Plasma Membrane
P29017	T-cell surface glycoprotein CD1c precursor (CD1c antigen).	Plasma Membrane
P06127	[T-cell surface glycoprotein CD5 precursor (Lymphocyte antigen T1/Leu-, 1).]	Plasma Membrane
P21926	[CD9 antigen (p24) (Leukocyte antigen MIC3) (Motility-related protein), (MRP-1) (Tetraspanin-29) (Tspan-29).]	Plasma Membrane
Q9Y5H8	Protocadherin alpha 3 precursor (PCDH-alpha3).	Plasma Membrane
Q9Y5H6	Protocadherin alpha 8 precursor (PCDH-alpha8).	Plasma Membrane
Q9Y5E8	Protocadherin beta 15 precursor (PCDH-beta15).	Plasma Membrane
P32246	[C-C chemokine receptor type 1 (C-C CKR-1) (CC-CKR-1) (CCR-1) (CCR1), (Macrophage inflammatory protein 1-alpha receptor) (MIP-1alpha-R), (RANTES-R) (HM145) (LD78 receptor) (CD191 antigen).]	Plasma Membrane
P51795	Chloride channel protein 5 (ClC-5).	Plasma Membrane
P02748	[Complement component C9 precursor [Contains: Complement component C9a;, Complement component C9b].]	Plasma Membrane
Q9Y5Q5	[Atrial natriuteric peptide-converting enzyme (EC 3.4.21.-) (pro-ANP-, converting enzyme) (Corin) (Heart-specific serine proteinase ATC2), (Transmembrane protease, serine 10).]	Plasma Membrane
P17927	[Complement receptor type 1 precursor (C3b/C4b receptor) (CD35, antigen).]	Plasma Membrane
P30825	[High affinity cationic amino acid transporter 1 (CAT-1) (CAT1) (System, Y+ basic amino acid transporter) (Ecotropic retroviral leukemia, receptor homolog) (ERR) (Ecotropic retrovirus receptor homolog).]	Plasma Membrane
P35212	Gap junction alpha-4 protein (Connexin-37) (Cx37).	Plasma Membrane
Q02846	[Retinal guanylyl cyclase 1 precursor (EC 4.6.1.2) (Guanylate cyclase, 2D, retinal) (RETGC-1) (Rod outer segment membrane guanylate cyclase), (ROS-GC).]	Plasma Membrane
P31785	[Cytokine receptor common gamma chain precursor (Gamma-C) (Interleukin-, 2 receptor gamma chain) (IL-2R gamma chain) (p64) (CD132 antigen).]	Plasma Membrane
O60469	Down syndrome cell adhesion molecule precursor (CHD2).	Plasma Membrane
P21453	[Sphingosine 1-phosphate receptor Edg-1 (Sphingosine 1-phosphate, receptor 1) (S1P1).]	Plasma Membrane

**P52799**	[Ephrin-B2 precursor (EPH-related receptor tyrosine kinase ligand 5), (LERK-5) (HTK ligand) (HTK-L).]	Plasma Membrane
**P29320**	[Ephrin type-A receptor 3 precursor (EC 2.7.10.1) (Tyrosine-protein, kinase receptor ETK1) (HEK) (HEK4) (Tyrosine-protein kinase TYRO4).]	Plasma Membrane
**P21802**	[Fibroblast growth factor receptor 2 precursor (EC 2.7.10.1) (FGFR-2), (Keratinocyte growth factor receptor 2) (CD332 antigen).]	Plasma Membrane
**Q04609**	[Glutamate carboxypeptidase 2 (EC 3.4.17.21) (Glutamate, carboxypeptidase II) (Membrane glutamate carboxypeptidase) (mGCP) (N-, acetylated-alpha-linked acidic dipeptidase I) (NAALADase I), (Pteroylpoly-gamma-glutamate carboxypeptidase) (Folylpoly-gamma-, glutamate carboxypeptidase) (FGCP) (Folate hydrolase 1) (Prostate-, specific membrane antigen) (PSMA) (PSM).]	Plasma Membrane
**P48169**	[Gamma-aminobutyric-acid receptor subunit alpha-4 precursor (GABA(A), receptor subunit alpha-4).]	Plasma Membrane
**Q9UN88**	[Gamma-aminobutyric-acid receptor subunit theta precursor (GABA(A), receptor subunit theta).]	Plasma Membrane
**P50395**	[Rab GDP dissociation inhibitor beta (Rab GDI beta) (Guanosine, diphosphate dissociation inhibitor 2) (GDI-2).]	Plasma Membrane
**Q16099**	[Glutamate receptor, ionotropic kainate 4 precursor (Glutamate receptor, KA-1) (KA1) (Excitatory amino acid receptor 1) (EAA1).]	Plasma Membrane
**P42262**	[Glutamate receptor 2 precursor (GluR-2) (GluR-B) (GluR-K2) (Glutamate, receptor ionotropic, AMPA 2) (AMPA-selective glutamate receptor 2).]	Plasma Membrane
**O43194**	Probable G-protein coupled receptor 39.	Plasma Membrane
**O15529**	Putative G-protein coupled receptor 42.	Plasma Membrane
**P07359**	[Platelet glycoprotein Ib alpha chain precursor (Glycoprotein Ibalpha), (GP-Ib alpha) (GPIbA) (GPIb-alpha) (Antigen CD42b-alpha) (CD42b, antigen) [Contains: Glycocalicin].]	Plasma Membrane
**P46093**	Probable G-protein coupled receptor 4 (G-protein coupled receptor 19).	Plasma Membrane
**P05536**	HLA class II histocompatibility antigen, DQ(W3) alpha chain precursor.	Plasma Membrane
**Q92839**	[Hyaluronan synthase 1 (EC 2.4.1.212) (Hyaluronate synthase 1), (Hyaluronic acid synthase 1) (HA synthase 1) (HuHAS1).]	Plasma Membrane
**P05981**	[Serine protease hepsin (EC 3.4.21.106) (Transmembrane protease, serine, 1) [Contains: Serine protease hepsin non-catalytic chain; Serine, protease hepsin catalytic chain].]	Plasma Membrane
**P11142**	Heat shock cognate 71 kDa protein (Heat shock 70 kDa protein 8).	Plasma Membrane
**P42701**	[Interleukin-12 receptor beta-1 chain precursor (IL-12R-beta1), (Interleukin-12 receptor beta) (IL-12 receptor beta component) (IL-, 12RB1) (CD212 antigen).]	Plasma Membrane
**Q9UII2**	ATPase inhibitor, mitochondrial precursor.	Plasma Membrane
**P05362**	[Intercellular adhesion molecule 1 precursor (ICAM-1) (Major group, rhinovirus receptor) (CD54 antigen).]	Plasma Membrane
**P01589**	[Interleukin-2 receptor alpha chain precursor (IL-2 receptor alpha, subunit) (IL-2-RA) (IL2-RA) (p55) (TAC antigen) (CD25 antigen).]	Plasma Membrane
**P53708**	[Integrin alpha-8 precursor [Contains: Integrin alpha-8 heavy chain;, Integrin alpha-8 light chain].]	Plasma Membrane
**Q13349**	[Integrin alpha-D precursor (Leukointegrin alpha D) (ADB2) (CD11d, antigen).]	Plasma Membrane
**P20701**	[Integrin alpha-L precursor (Leukocyte adhesion glycoprotein LFA-1, alpha chain) (LFA-1A) (Leukocyte function-associated molecule 1 alpha, chain) (CD11a antigen).]	Plasma Membrane
**P05556**	[Integrin beta-1 precursor (Fibronectin receptor subunit beta), (Integrin VLA-4 subunit beta) (CD29 antigen).]	Plasma Membrane
**P18564**	Integrin beta-6 precursor.	Plasma Membrane
**P43630**	[Killer cell immunoglobulin-like receptor 3DL2 precursor (MHC class I, NK cell receptor) (Natural killer-associated transcript 4) (NKAT-4), (p70 natural killer cell receptor clone CL-5) (CD158k antigen).]	Plasma Membrane
**Q01650**	[Large neutral amino acids transporter small subunit 1 (L-type amino, acid transporter 1) (4F2 light chain) (4F2 LC) (4F2LC) (CD98 light, chain) (Integral membrane protein E16) (hLAT1).]	Plasma Membrane
**P01130**	Low-density lipoprotein receptor precursor (LDL receptor).	Plasma Membrane

**P16109**	[P-selectin precursor (Granule membrane protein 140) (GMP-140) (PADGEM), (Leukocyte-endothelial cell adhesion molecule 3) (LECAM3) (CD62P, antigen).]	Plasma Membrane
**P42702**	[Leukemia inhibitory factor receptor precursor (LIF receptor) (LIF-R), (CD118 antigen).]	Plasma Membrane
**Q07954**	[Prolow-density lipoprotein receptor-related protein 1 precursor (LRP), (Alpha-2-macroglobulin receptor) (A2MR) (Apolipoprotein E receptor), (APOER) (CD91 antigen) [Contains: Low-density lipoprotein receptor-, related protein 1 85 kDa subunit (LRP-85); Low-density lipoprotein, receptor-related protein 1 515 kDa subunit (LRP-515); Low-density, lipoprotein receptor-related protein 1 intracellular domain (LRPICD)].]	Plasma Membrane
**P15529**	[Membrane cofactor protein precursor (Trophoblast leukocyte common, antigen) (TLX) (CD46 antigen).]	Plasma Membrane
**P08183**	[Multidrug resistance protein 1 (EC 3.6.3.44) (ATP-binding cassette, sub-family B member 1) (P-glycoprotein 1) (CD243 antigen).]	Plasma Membrane
**P51512**	[Matrix metalloproteinase-16 precursor (EC 3.4.24.-) (MMP-16), (Membrane-type matrix metalloproteinase 3) (MT-MMP 3) (MTMMP3), (Membrane-type-3 matrix metalloproteinase) (MT3-MMP) (MT3MMP) (MMP-, X2).]	Plasma Membrane
**O60669**	[Monocarboxylate transporter 2 (MCT 2) (Solute carrier family 16 member, 7).]	Plasma Membrane
**O95255**	[Multidrug resistance-associated protein 6 (ATP-binding cassette sub-, family C member 6) (Anthracycline resistance-associated protein), (Multi-specific organic anion transporter-E) (MOAT-E).]	Plasma Membrane
**P15941**	[Mucin-1 precursor (MUC-1) (Polymorphic epithelial mucin) (PEM) (PEMT), (Episialin) (Tumor-associated mucin) (Carcinoma-associated mucin), (Tumor-associated epithelial membrane antigen) (EMA) (H23AG) (Peanut-, reactive urinary mucin) (PUM) (Breast carcinoma-associated antigen, DF3) (CD227 antigen).]	Plasma Membrane
**Q13508**	[Ecto-ADP-ribosyltransferase 3 precursor (EC 2.4.2.31) (NAD(P)(+)--, arginine ADP-ribosyltransferase 3) (Mono(ADP-ribosyl)transferase 3).]	Plasma Membrane
**Q92859**	Neogenin precursor.	Plasma Membrane
**P49281**	[Natural resistance-associated macrophage protein 2 (NRAMP 2) (Divalent, metal transporter 1) (DMT1).]	Plasma Membrane
**O95665**	[Neurotensin receptor type 2 (NT-R-2) (Levocabastine-sensitive, neurotensin receptor) (NTR2 receptor).]	Plasma Membrane
**P49757**	Protein numb homolog (h-Numb) (Protein S171).	Plasma Membrane
**Q9ULB1**	Neurexin-1-alpha precursor (Neurexin I-alpha).	Plasma Membrane
**P50391**	[Neuropeptide Y receptor type 4 (NPY4-R) (Pancreatic polypeptide, receptor 1) (PP1).]	Plasma Membrane
**Q16625**	Occludin.	Plasma Membrane
**Q14982**	[Opioid-binding protein/cell adhesion molecule precursor (OBCAM), (Opioid-binding cell adhesion molecule) (OPCML).]	Plasma Membrane
**Q14444**	[Caprin-1 (Cytoplasmic activation- and proliferation-associated protein, 1) (GPI-anchored membrane protein 1) (GPI-anchored protein p137), (p137GPI) (Membrane component chromosome 11 surface marker 1).]	Plasma Membrane
**O15547**	[P2X purinoceptor 6 (ATP receptor) (P2X6) (Purinergic receptor) (P2XM), (Purinergic receptor P2X-like 1).]	Plasma Membrane
**Q15077**	P2Y purinoceptor 6 (P2Y6).	Plasma Membrane
**P41586**	[Pituitary adenylate cyclase-activating polypeptide type I receptor, precursor (PACAP type I receptor) (PACAP-R-1).]	Plasma Membrane
**O00254**	[Proteinase-activated receptor 3 precursor (PAR-3) (Thrombin receptor-, like 2) (Coagulation factor II receptor-like 2).]	Plasma Membrane
**P07237**	[Protein disulfide-isomerase precursor (EC 5.3.4.1) (PDI) (Prolyl 4-, hydroxylase subunit beta) (Cellular thyroid hormone-binding protein), (p55).]	Plasma Membrane
**O43490**	[Prominin-1 precursor (Prominin-like protein 1) (Antigen AC133) (CD133, antigen).]	Plasma Membrane
**P23468**	[Receptor-type tyrosine-protein phosphatase delta precursor, (EC 3.1.3.48) (Protein-tyrosine phosphatase delta) (R-PTP-delta).]	Plasma Membrane
**Q15262**	[Receptor-type tyrosine-protein phosphatase kappa precursor, (EC 3.1.3.48) (Protein-tyrosine phosphatase kappa) (R-PTP-kappa).]	Plasma Membrane

**Q92729**	[Receptor-type tyrosine-protein phosphatase U precursor (EC 3.1.3.48), (R-PTP-U) (Protein-tyrosine phosphatase J) (PTP-J) (Pancreatic, carcinoma phosphatase 2) (PCP-2).]	Plasma Membrane
**Q13671**	[Ras and Rab interactor 1 (Ras interaction/interference protein 1) (Ras, inhibitor JC99).]	Plasma Membrane
**Q04912**	[Macrophage-stimulating protein receptor precursor (EC 2.7.10.1) (MSP, receptor) (p185-Ron) (CD136 antigen) (CDw136) [Contains: Macrophage-, stimulating protein receptor alpha chain; Macrophage-stimulating, protein receptor beta chain].]	Plasma Membrane
**P43007**	[Neutral amino acid transporter A (SATT) (Solute carrier family 1, member 4) (Alanine/serine/cysteine/ threonine transporter) (ASCT1).]	Plasma Membrane
**O43556**	Epsilon-sarcoglycan precursor (Epsilon-SG).	Plasma Membrane
**Q9Y289**	[Sodium-dependent multivitamin transporter (Na(+)-dependent, multivitamin transporter).]	Plasma Membrane
**Q92673**	[Sortilin-related receptor precursor (Sorting protein-related receptor, containing LDLR class A repeats) (SorLA) (SorLA-1) (Low-density, lipoprotein receptor relative with 11 ligand-binding repeats) (LDLR, relative with 11 ligand-binding repeats) (LR11).]	Plasma Membrane
**P48230**	[Transmembrane 4 L6 family member 4 (Intestine and liver tetraspan, membrane protein) (IL-TMP).]	Plasma Membrane
**P21731**	Thromboxane A2 receptor (TXA2-R) (Prostanoid TP receptor).	Plasma Membrane
**Q02763**	[Angiopoietin-1 receptor precursor (EC 2.7.10.1) (Tyrosine-protein, kinase receptor TIE-2) (hTIE2) (Tyrosine-protein kinase receptor TEK), (p140 TEK) (Tunica interna endothelial cell kinase) (CD202b antigen).]	Plasma Membrane
**O15393**	[Transmembrane protease, serine 2 precursor (EC 3.4.21.-) (Serine, protease 10) [Contains: Transmembrane protease, serine 2 non-catalytic, chain; Transmembrane protease, serine 2 catalytic chain].]	Plasma Membrane
**Q16620**	[BDNF/NT-3 growth factors receptor precursor (EC 2.7.10.1), (Neurotrophic tyrosine kinase receptor type 2) (TrkB tyrosine kinase), (GP145-TrkB) (Trk-B).]	Plasma Membrane
**Q03405**	[Urokinase plasminogen activator surface receptor precursor (uPAR) (U-, PAR) (Monocyte activation antigen Mo3) (CD87 antigen).]	Plasma Membrane
**P38606**	[Vacuolar ATP synthase catalytic subunit A (EC 3.6.3.14) (V-ATPase, subunit A) (Vacuolar proton pump subunit alpha) (V-ATPase 69 kDa, subunit) (Vacuolar ATPase isoform VA68).]	Plasma Membrane
**P35968**	[Vascular endothelial growth factor receptor 2 precursor (EC 2.7.10.1), (VEGFR-2) (Kinase insert domain receptor) (Protein-tyrosine kinase, receptor Flk-1) (CD309 antigen).]	Plasma Membrane
**P32241**	[Vasoactive intestinal polypeptide receptor 1 precursor (VIP-R-1), (Pituitary adenylate cyclase-activating polypeptide type II receptor), (PACAP type II receptor) (PACAP-R-2).]	Plasma Membrane
**P30939**	5-hydroxytryptamine 1F receptor (5-HT-1F) (Serotonin receptor 1F).	Plasma Membrane
**P46098**	[5-hydroxytryptamine 3 receptor precursor (5-HT-3) (Serotonin-gated ion, channel receptor) (5-HT3R).]	Plasma Membrane
**O95342**	Bile salt export pump (ATP-binding cassette sub-family B member 11).	Plasma Membrane
**P24588**	[A-kinase anchor protein 5 (A-kinase anchor protein 79 kDa) (AKAP 79), (cAMP-dependent protein kinase regulatory subunit II high affinity-, binding protein) (H21).]	Plasma Membrane
**P16157**	Ankyrin-1 (Erythrocyte ankyrin) (Ankyrin-R).	Plasma Membrane
**P16066**	[Atrial natriuretic peptide receptor A precursor (ANP-A) (ANPRA) (GC-A), (Guanylate cyclase) (EC 4.6.1.2) (NPR-A) (Atrial natriuretic peptide, A-type receptor).]	Plasma Membrane
**O95445**	Apolipoprotein M (Apo-M) (ApoM) (Protein G3a).	Plasma Membrane
**Q13796**	Protein Shroom2 (Apical-like protein) (Protein APXL).	Plasma Membrane
**P48751**	[Anion exchange protein 3 (Neuronal band 3-like protein) (Solute, carrier family 4 member 3) (Cardiac/brain band 3-like protein), (CAE3/BAE3).]	Plasma Membrane

**Q9Y2A9**	[UDP-GlcNAc:betaGal beta-1,3-N-acetylglucosaminyltransferase 3, (EC 2.4.1.-) (Beta3Gn-T3) (BGnT-3) (Core 1 extending beta-1,3-N-, acetylglucosaminyltransferase) (Core1-beta3GlcNAcT) (Beta-1,3-, galactosyltransferase 8) (Beta-1,3-GalTase 8) (Beta3Gal-T8) (b3Gal-T8), (UDP galactose:beta-N-acetylglucosamine beta-1,3-galactosyltransferase, 8) (UDP-Gal:beta-GlcNAc beta-1,3-galactosyltransferase 8) (Beta-3-Gx-, T8).]	Plasma Membrane
**O00238**	[Bone morphogenetic protein receptor type IB precursor (EC 2.7.11.30), (CDw293 antigen).]	Plasma Membrane
**P04839**	[Cytochrome b-245 heavy chain (p22 phagocyte B-cytochrome) (Neutrophil, cytochrome b 91 kDa polypeptide) (CGD91-phox) (gp91-phox) (gp91-1), (Heme-binding membrane glycoprotein gp91phox) (Cytochrome b(558), subunit beta) (Cytochrome b558 subunit beta) (Superoxide-generating, NADPH oxidase heavy chain subunit) (NADPH oxidase 2).]	Plasma Membrane
**P41180**	[Extracellular calcium-sensing receptor precursor (CaSR) (Parathyroid, Cell calcium-sensing receptor).]	Plasma Membrane
**P34972**	Cannabinoid receptor 2 (CB2) (CB-2) (CX5).	Plasma Membrane
**P32238**	[Cholecystokinin type A receptor (CCK-A receptor) (CCK-AR), (Cholecystokinin-1 receptor) (CCK1-R).]	Plasma Membrane
**P08571**	[Monocyte differentiation antigen CD14 precursor (Myeloid cell-specific, leucine-rich glycoprotein) [Contains: Monocyte differentiation antigen, CD14, urinary form; Monocyte differentiation antigen CD14, membrane-, bound form].]	Plasma Membrane
**P16070**	[CD44 antigen precursor (Phagocytic glycoprotein I) (PGP-1) (HUTCH-I), (Extracellular matrix receptor-III) (ECMR-III) (GP90 lymphocyte, homing/adhesion receptor) (Hermes antigen) (Hyaluronate receptor), (Heparan sulfate proteoglycan) (Epican) (CDw44).]	Plasma Membrane
**P31358**	[CAMPATH-1 antigen precursor (Cambridge pathology 1 antigen), (Epididymal secretory protein E5) (CD52 antigen) (CDw52).]	Plasma Membrane
**Q9Y5I3**	Protocadherin alpha 1 precursor (PCDH-alpha1).	Plasma Membrane
**Q9UN74**	Protocadherin alpha 4 precursor (PCDH-alpha4).	Plasma Membrane
**Q9Y5I1**	Protocadherin alpha 11 precursor (PCDH-alpha11).	Plasma Membrane
**Q9Y5F1**	Protocadherin beta 12 precursor (PCDH-beta12).	Plasma Membrane
**P51684**	[C-C chemokine receptor type 6 (C-C CKR-6) (CC-CKR-6) (CCR-6) (LARC, receptor) (GPR-CY4) (GPRCY4) (Chemokine receptor-like 3) (CKR-L3), (DRY6) (G-protein coupled receptor 29) (CD196 antigen).]	Plasma Membrane
**P35523**	[Chloride channel protein, skeletal muscle (Chloride channel protein 1), (ClC-1).]	Plasma Membrane
**O60503**	[Adenylate cyclase type 9 (EC 4.6.1.1) (Adenylate cyclase type IX) (ATP, pyrophosphate-lyase 9) (Adenylyl cyclase 9).]	Plasma Membrane
**O94907**	[Dickkopf-related protein 1 precursor (Dkk-1) (Dickkopf-1) (hDkk-1), (SK).]	Plasma Membrane
**P21453**	[Sphingosine 1-phosphate receptor Edg-1 (Sphingosine 1-phosphate, receptor 1) (S1P1).]	Plasma Membrane
**P54762**	[Ephrin type-B receptor 1 precursor (EC 2.7.10.1) (Tyrosine-protein, kinase receptor EPH-2) (NET) (HEK6) (ELK).]	Plasma Membrane
**P21860**	[Receptor tyrosine-protein kinase erbB-3 precursor (EC 2.7.10.1) (c-, erbB3) (Tyrosine kinase-type cell surface receptor HER3).]	Plasma Membrane
**P25101**	[Endothelin-1 receptor precursor (Endothelin A receptor) (ET-A) (hET-, AR) (ETA-R).]	Plasma Membrane
**P34910**	[EVI2B protein precursor (Ecotropic viral integration site 2B protein, homolog) (EVI-2B).]	Plasma Membrane
**P24071**	[Immunoglobulin alpha Fc receptor precursor (IgA Fc receptor) (CD89, antigen).]	Plasma Membrane
**P41440**	[Folate transporter 1 (Solute carrier family 19 member 1) (Placental, folate transporter) (FOLT) (Reduced folate carrier protein) (RFC), (Intestinal folate carrier) (IFC-1).]	Plasma Membrane
**P31644**	[Gamma-aminobutyric-acid receptor subunit alpha-5 precursor (GABA(A), receptor subunit alpha-5).]	Plasma Membrane
**Q99062**	[Granulocyte colony-stimulating factor receptor precursor (G-CSF-R), (CD114 antigen).]	Plasma Membrane
**P10912**	[Growth hormone receptor precursor (GH receptor) (Somatotropin, receptor) [Contains: Growth hormone-binding protein (GH-binding, protein) (GHBP) (Serum-binding protein)].]	Plasma Membrane

Q13002	[Glutamate receptor, ionotropic kainate 2 precursor (Glutamate receptor, 6) (GluR-6) (GluR6) (Excitatory amino acid receptor 4) (EAA4).]	Plasma Membrane
P48058	[Glutamate receptor 4 precursor (GluR-4) (GluR4) (GluR-D) (Glutamate, receptor ionotropic, AMPA 4) (AMPA-selective glutamate receptor 4).]	Plasma Membrane
P51654	Glypican-3 precursor (Intestinal protein OCI-5) (GTR2-2) (MXR7).	Plasma Membrane
P46091	Probable G-protein coupled receptor 1.	Plasma Membrane
P48167	[Glycine receptor subunit beta precursor (Glycine receptor 58 kDa, subunit).]	Plasma Membrane
P14672	[Solute carrier family 2, facilitated glucose transporter member 4, (Glucose transporter type 4, insulin-responsive) (GLUT-4).]	Plasma Membrane
O00219	[Hyaluronan synthase 3 (EC 2.4.1.212) (Hyaluronate synthase 3), (Hyaluronic acid synthase 3) (HA synthase 3).]	Plasma Membrane
P49019	[Nicotinic acid receptor 2 (G-protein coupled receptor 109B) (G-protein, coupled receptor HM74) (G-protein coupled receptor HM74B).]	Plasma Membrane
P54652	Heat shock-related 70 kDa protein 2 (Heat shock 70 kDa protein 2).	Plasma Membrane
Q13651	[Interleukin-10 receptor alpha chain precursor (IL-10R-A) (IL-10R1), (CDw210a antigen).]	Plasma Membrane
P08069	[Insulin-like growth factor 1 receptor precursor (EC 2.7.10.1), (Insulin-like growth factor I receptor) (IGF-I receptor) (CD221, antigen) [Contains: Insulin-like growth factor 1 receptor alpha chain;, Insulin-like growth factor 1 receptor beta chain].]	Plasma Membrane
P15260	[Interferon-gamma receptor alpha chain precursor (IFN-gamma-R1) (CD119, antigen) (CDw119).]	Plasma Membrane
O43187	Interleukin-1 receptor-associated kinase-like 2 (IRAK-2).	Plasma Membrane
P63252	[Inward rectifier potassium channel 2 (Potassium channel, inwardly, rectifying subfamily J member 2) (Inward rectifier K(+) channel, Kir2.1) (Cardiac inward rectifier potassium channel) (IRK1).]	Plasma Membrane
P56199	[Integrin alpha-1 precursor (Laminin and collagen receptor) (VLA-1), (CD49a antigen).]	Plasma Membrane
P08648	[Integrin alpha-5 precursor (Fibronectin receptor subunit alpha), (Integrin alpha-F) (VLA-5) (CD49e antigen) [Contains: Integrin alpha-5, heavy chain; Integrin alpha-5 light chain].]	Plasma Membrane
Q13797	Integrin alpha-9 precursor (Integrin alpha-RLC).	Plasma Membrane
P38570	[Integrin alpha-E precursor (Mucosal lymphocyte 1 antigen) (HML-1, antigen) (Integrin alpha-IEL) (CD103 antigen) [Contains: Integrin, alpha-E light chain; Integrin alpha-E heavy chain].]	Plasma Membrane
P11215	[Integrin alpha-M precursor (Cell surface glycoprotein MAC-1 alpha, subunit) (CR-3 alpha chain) (Leukocyte adhesion receptor MO1), (Neutrophil adherence receptor) (CD11b antigen).]	Plasma Membrane
P05107	[Integrin beta-2 precursor (Cell surface adhesion glycoproteins LFA-, 1/CR3/p150,95 subunit beta) (Complement receptor C3 subunit beta), (CD18 antigen).]	Plasma Membrane
P14151	[L-selectin precursor (Lymph node homing receptor) (Leukocyte adhesion, molecule 1) (LAM-1) (Leukocyte surface antigen Leu-8) (TQ1) (gp90-MEL), (Leukocyte-endothelial cell adhesion molecule 1) (LECAM1) (CD62L, antigen).]	Plasma Membrane
Q02978	[Mitochondrial 2-oxoglutarate/malate carrier protein (OGCP) (Solute, carrier family 25 member 11).]	Plasma Membrane
P21439	[Multidrug resistance protein 3 (EC 3.6.3.44) (ATP-binding cassette, sub-family B member 4) (P-glycoprotein 3).]	Plasma Membrane
Q12866	[Proto-oncogene tyrosine-protein kinase MER precursor (EC 2.7.10.1) (C-, mer) (Receptor tyrosine kinase MerTK).]	Plasma Membrane
Q14831	Metabotropic glutamate receptor 7 precursor (mGluR7).	Plasma Membrane
Q16653	Myelin-oligodendrocyte glycoprotein precursor.	Plasma Membrane
O15374	[Monocarboxylate transporter 5 (MCT 5) (MCT 4) (Solute carrier family, 16 member 4).]	Plasma Membrane
P33527	[Multidrug resistance-associated protein 1 (ATP-binding cassette sub-, family C member 1) (Leukotriene C(4) transporter) (LTC4 transporter).]	Plasma Membrane

P43121	[Cell surface glycoprotein MUC18 precursor (Melanoma-associated antigen, MUC18) (Melanoma cell adhesion molecule) (Melanoma-associated antigen, A32) (S-endo 1 endothelial-associated antigen) (Cell surface, glycoprotein P1H12) (CD146 antigen).]	Plasma Membrane
P25189	[Myelin P0 protein precursor (Myelin protein zero) (Myelin peripheral, protein) (MPP).]	Plasma Membrane
P32418	[Sodium/calcium exchanger 1 precursor (Na(+)/Ca(2+)-exchange protein, 1).]	Plasma Membrane
P08473	[Neprilysin (EC 3.4.24.11) (Neutral endopeptidase) (NEP), (Enkephalinase) (Neutral endopeptidase 24.11) (Atriopeptidase) (Common, acute lymphocytic leukemia antigen) (CALLA) (CD10 antigen).]	Plasma Membrane
O76082	[Organic cation/carnitine transporter 2 (Solute carrier family 22, member 5) (High-affinity sodium-dependent carnitine cotransporter).]	Plasma Membrane
Q99572	[P2X purinoceptor 7 (ATP receptor) (P2X7) (Purinergic receptor) (P2Z, receptor).]	Plasma Membrane
P16284	[Platelet endothelial cell adhesion molecule precursor (PECAM-1), (EndoCAM) (GPIIA') (CD31 antigen).]	Plasma Membrane
P46059	[Oligopeptide transporter, small intestine isoform (Peptide transporter, 1) (Intestinal H(+)/peptide cotransporter) (Solute carrier family 15, member 1).]	Plasma Membrane
P01833	[Polymeric-immunoglobulin receptor precursor (Poly-Ig receptor) (PIGR), (Hepatocellular carcinoma-associated protein TB6) [Contains: Secretory, component].]	Plasma Membrane
Q15149	Plectin-1 (PLTN) (PCN) (Hemidesmosomal protein 1) (HD1) (Plectin-11).	Plasma Membrane
P40967	[Melanocyte protein Pmel 17 precursor (Silver locus protein homolog), (Melanocyte lineage-specific antigen GP100) (Melanoma-associated ME20, antigen) (ME20M) (ME20-M) [Contains: ME20-S (Secreted melanoma-, associated ME20 antigen) (ME20S) (95 kDa melanocyte-specific secreted, glycoprotein)].]	Plasma Membrane
P10586	[Receptor-type tyrosine-protein phosphatase F precursor (EC 3.1.3.48), (LAR protein) (Leukocyte antigen related).]	Plasma Membrane
P28827	[Receptor-type tyrosine-protein phosphatase mu precursor (EC 3.1.3.48), (Protein-tyrosine phosphatase mu) (R-PTP-mu).]	Plasma Membrane
Q92692	[Poliovirus receptor-related protein 2 precursor (Herpes virus entry, mediator B) (HveB) (Nectin-2) (CD112 antigen).]	Plasma Membrane
P47804	RPE-retinal G protein-coupled receptor.	Plasma Membrane
Q92736	[Ryanodine receptor 2 (Cardiac muscle-type ryanodine receptor) (RyR2), (RYR-2) (Cardiac muscle ryanodine receptor-calcium release channel), (hRYR-2).]	Plasma Membrane
P30874	Somatostatin receptor type 2 (SS2R) (SRIF-1).	Plasma Membrane
Q9Y5Y6	[Suppressor of tumorigenicity protein 14 (EC 3.4.21.109) (Serine, protease 14) (Matriptase) (Membrane-type serine protease 1) (MT-SP1), (Prostamin) (Serine protease TADG-15) (Tumor-associated, differentially-expressed gene 15 protein).]	Plasma Membrane
O14798	[Tumor necrosis factor receptor superfamily member 10C precursor (Decoy, receptor 1) (DcR1) (Decoy TRAIL receptor without death domain) (TNF-, related apoptosis-inducing ligand receptor 3) (TRAIL receptor 3), (TRAIL-R3) (Trail receptor without an intracellular domain), (Lymphocyte inhibitor of TRAIL) (Antagonist decoy receptor for, TRAIL/Apo-2L) (CD263 antigen).]	Plasma Membrane
O43280	[Trehalase precursor (EC 3.2.1.28) (Alpha,alpha-trehalase), (Alpha,alpha-trehalose glucohydrolase).]	Plasma Membrane
Q16288	[NT-3 growth factor receptor precursor (EC 2.7.10.1) (Neurotrophic, tyrosine kinase receptor type 3) (TrkC tyrosine kinase) (GP145-TrkC), (Trk-C).]	Plasma Membrane
Q9UL62	Short transient receptor potential channel 5 (TrpC5) (Htrp-5) (Htrp5).	Plasma Membrane
Q13336	Urea transporter, erythrocyte.	Plasma Membrane
Q00341	[Vigilin (High density lipoprotein-binding protein) (HDL-binding, protein).]	Plasma Membrane
P35916	[Vascular endothelial growth factor receptor 3 precursor (EC 2.7.10.1), (VEGFR-3) (Tyrosine-protein kinase receptor FLT4).]	Plasma Membrane
P41587	[Vasoactive intestinal polypeptide receptor 2 precursor (VIP-R-2), (Pituitary adenylate cyclase-activating polypeptide type III receptor), (PACAP type III receptor) (PACAP-R-3) (Helodermin-preferring VIP, receptor).]	Plasma Membrane
Q07157	[Tight junction protein ZO-1 (Zonula occludens 1 protein) (Zona, occludens 1 protein) (Tight junction protein 1).]	Plasma Membrane
Q9UBJ2	[ATP-binding cassette sub-family D member 2 (Adrenoleukodystrophy-, related protein) (hALDR) (Adrenoleukodystrophy-like 1).]	Protein Complex

**P25054**	Adenomatous polyposis coli protein (Protein APC).	Protein Complex
**O15145**	[Actin-related protein 2/3 complex subunit 3 (ARP2/3 complex 21 kDa, subunit) (p21-ARC).]	Protein Complex
**Q9Y4X5**	[Protein ariadne-1 homolog (ARI-1) (Ubiquitin-conjugating enzyme E2-, binding protein 1) (UbcH7-binding protein) (UbcM4-interacting protein), (HHARI) (H7-AP2) (Monocyte protein 6) (MOP-6).]	Protein Complex
**P20749**	B-cell lymphoma 3-encoded protein (Protein Bcl-3).	Protein Complex
**P51587**	[Breast cancer type 2 susceptibility protein (Fanconi anemia group D1, protein).]	Protein Complex
**P02745**	Complement C1q subcomponent subunit A precursor.	Protein Complex
**P29033**	Gap junction beta-2 protein (Connexin-26) (Cx26).	Protein Complex
**Q14232**	[Translation initiation factor eIF-2B subunit alpha (eIF-2B GDP-GTP, exchange factor subunit alpha).]	Protein Complex
**P02675**	Fibrinogen beta chain precursor [Contains: Fibrinopeptide B].	Protein Complex
**P69905**	Hemoglobin subunit alpha (Hemoglobin alpha chain) (Alpha-globin).	Protein Complex
**Q08334**	[Interleukin-10 receptor beta chain precursor (IL-10R-B) (IL-10R2), (Cytokine receptor family 2 member 4) (Cytokine receptor class-II, member 4) (CRF2-4) (CDw210b antigen).]	Protein Complex
**Q04637**	[Eukaryotic translation initiation factor 4 gamma 1 (eIF-4-gamma 1), (eIF-4G1) (eIF-4G 1) (p220).]	Protein Complex
**P67870**	Casein kinase II subunit beta (CK II beta) (Phosvitin) (G5a).	Protein Complex
**O15066**	[Kinesin-like protein KIF3B (Microtubule plus end-directed kinesin, motor 3B) (HH0048).]	Protein Complex
**Q12840**	[Kinesin heavy chain isoform 5A (Neuronal kinesin heavy chain) (NKHC), (Kinesin heavy chain neuron-specific 1).]	Protein Complex
**P46019**	[Phosphorylase b kinase regulatory subunit alpha, liver isoform, (Phosphorylase kinase alpha L subunit).]	Protein Complex
**P08590**	[Myosin light polypeptide 3 (Myosin light chain 1, slow-twitch muscle, B/ventricular isoform) (MLC1SB) (Ventricular/slow twitch myosin alkali, light chain) (Cardiac myosin light chain-1) (CMLC1).]	Protein Complex
**O00566**	[U3 small nucleolar ribonucleoprotein protein MPP10 (M phase, phosphoprotein 10).]	Protein Complex
**P35749**	[Myosin-11 (Myosin heavy chain 11) (Myosin heavy chain, smooth muscle, isoform) (SMMHC).]	Protein Complex
**O00329**	[Phosphatidylinositol-4,5-bisphosphate 3-kinase catalytic subunit delta, isoform (EC 2.7.1.153) (PI3-kinase p110 subunit delta) (PtdIns-3-, kinase p110) (PI3K) (p110delta).]	Protein Complex
**P28065**	[Proteasome subunit beta type-9 precursor (EC 3.4.25.1) (Proteasome, subunit beta-1i) (Proteasome chain 7) (Macropain chain 7), (Multicatalytic endopeptidase complex chain 7) (RING12 protein) (Low, molecular mass protein 2).]	Protein Complex
**P24386**	[Rab proteins geranylgeranyltransferase component A 1 (Rab escort, protein 1) (REP-1) (Choroideraemia protein) (TCD protein).]	Protein Complex
**P04844**	[Dolichyl-diphosphooligosaccharide--protein glycosyltransferase 63 kDa, subunit precursor (EC 2.4.1.119) (Ribophorin II) (RPN-II) (RIBIIR).]	Protein Complex
**P10155**	[60 kDa SS-A/Ro ribonucleoprotein (60 kDa Ro protein) (60 kDa, ribonucleoprotein Ro) (RoRNP) (Ro 60 kDa autoantigen) (TROVE domain, family member 2) (Sjoegren syndrome type A antigen) (SS-A) (Sjoegren, syndrome antigen A2).]	Protein Complex
**P21283**	[Vacuolar ATP synthase subunit C 1 (EC 3.6.3.14) (V-ATPase subunit C 1), (Vacuolar proton pump subunit C 1).]	Protein Complex
**Q14999**	Cullin-7 (CUL-7).	Protein Complex
**Q9Y2T4**	[Serine/threonine-protein phosphatase 2A 55 kDa regulatory subunit B, gamma isoform (PP2A, subunit B, B-gamma isoform) (PP2A, subunit B,, B55-gamma isoform) (PP2A, subunit B, PR55-gamma isoform) (PP2A,, subunit B, R2-gamma isoform) (IMYPNO1).]	Protein Complex
**O95782**	[AP-2 complex subunit alpha-1 (Adapter-related protein complex 2 alpha-, 1 subunit) (Alpha-adaptin A) (Adaptor protein complex AP-2 alpha-1, subunit) (Clathrin assembly protein complex 2 alpha-A large chain), (100 kDa coated vesicle protein A) (Plasma membrane adaptor HA2/AP2, adaptin alpha A subunit).]	Protein Complex

**Q9NP58**	[Mitochondrial ATP-binding cassette sub-family B member 6, (Mitochondrial ABC transporter 3) (Mt-ABC transporter 3), (Ubiquitously-expressed mammalian ABC half transporter) (P-, glycoprotein-related protein).]	Protein Complex
**O60566**	[Mitotic checkpoint serine/threonine-protein kinase BUB1 beta, (EC 2.7.11.1) (hBUBR1) (MAD3/BUB1-related protein kinase) (Mitotic, checkpoint kinase MAD3L) (SSK1).]	Protein Complex
**P02746**	Complement C1q subcomponent subunit B precursor.	Protein Complex
**P63098**	[Calcineurin subunit B isoform 1 (Protein phosphatase 2B regulatory, subunit 1) (Protein phosphatase 3 regulatory subunit B alpha isoform, 1).]	Protein Complex
**P23511**	[Nuclear transcription factor Y subunit alpha (Nuclear transcription, factor Y subunit A) (NF-YA) (CAAT-box DNA-binding protein subunit A).]	Protein Complex
**P49336**	[Cell division protein kinase 8 (EC 2.7.11.22) (EC 2.7.11.23) (Protein, kinase K35).]	Protein Complex
**P49770**	[Translation initiation factor eIF-2B subunit beta (eIF-2B GDP-GTP, exchange factor subunit beta) (S20I15) (S20III15).]	Protein Complex
**P07992**	DNA excision repair protein ERCC-1.	Protein Complex
**P02679**	Fibrinogen gamma chain precursor.	Protein Complex
**P35573**	[Glycogen debranching enzyme (Glycogen debrancher) [Includes: 4-alpha-, glucanotransferase (EC 2.4.1.25) (Oligo-1,4-1,4-glucantransferase);, Amylo-alpha-1,6-glucosidase (EC 3.2.1.33) (Amylo-1,6-glucosidase), (Dextrin 6-alpha-D-glucosidase)].]	Protein Complex
**P68871**	[Hemoglobin subunit beta (Hemoglobin beta chain) (Beta-globin), [Contains: LVV-hemorphin-7].]	Protein Complex
**Q9UL18**	[Eukaryotic translation initiation factor 2C 1 (eIF2C 1) (eIF-2C 1), (Argonaute-1) (Putative RNA-binding protein Q99).]	Protein Complex
**Q99613**	[Eukaryotic translation initiation factor 3 subunit 8 (eIF3 p110), (eIF3c).]	Protein Complex
**P10644**	[cAMP-dependent protein kinase type I-alpha regulatory subunit (Tissue-, specific extinguisher 1) (TSE1).]	Protein Complex
**Q00653**	[Nuclear factor NF-kappa-B p100 subunit (DNA-binding factor KBF2), (H2TF1) (Lymphocyte translocation chromosome 10) (Oncogene Lyt-10), (Lyt10) [Contains: Nuclear factor NF-kappa-B p52 subunit].]	Protein Complex
**O14782**	Kinesin-like protein KIF3C.	Protein Complex
**P49841**	Glycogen synthase kinase-3 beta (EC 2.7.11.26) (GSK-3 beta).	Protein Complex
**P07942**	Laminin subunit beta-1 precursor (Laminin B1 chain).	Protein Complex
**Q9UKX2**	[Myosin-2 (Myosin heavy chain 2) (Myosin heavy chain 2a) (MyHC-2a), (Myosin heavy chain, skeletal muscle, adult 2) (Myosin heavy chain, IIa) (MyHC-IIa).]	Protein Complex
**P12883**	[Myosin-7 (Myosin heavy chain 7) (Myosin heavy chain, cardiac muscle, beta isoform) (MyHC-beta) (Myosin heavy chain slow isoform) (MyHC-, slow).]	Protein Complex
**Q9UKX3**	[Myosin-13 (Myosin heavy chain 13) (Myosin heavy chain, skeletal, muscle, extraocular) (MyHC-eo).]	Protein Complex
**Q13200**	[26S proteasome non-ATPase regulatory subunit 2 (26S proteasome, regulatory subunit RPN1) (26S proteasome regulatory subunit S2) (26S, proteasome subunit p97) (Tumor necrosis factor type 1 receptor-, associated protein 2) (55.11 protein).]	Protein Complex
**Q12824**	[SWI/SNF-related matrix-associated actin-dependent regulator of, chromatin subfamily B member 1 (Integrase interactor 1 protein), (hSNF5) (BAF47).]	Protein Complex
**P11277**	Spectrin beta chain, erythrocyte (Beta-I spectrin).	Protein Complex
**P19429**	Troponin I, cardiac muscle (Cardiac troponin I).	Protein Complex
**Q06190**	[Serine/threonine-protein phosphatase 2A regulatory subunit B'' subunit, alpha (Serine/threonine-protein phosphatase 2A 72/130 kDa regulatory, subunit B) (PP2A, subunit B, B''-PR72/PR130) (PP2A, subunit B,, B72/B130 isoforms) (PP2A, subunit B, PR72/PR130 isoforms) (PP2A,, subunit B, R3 isoform).]	Protein Complex

**O94973**	[AP-2 complex subunit alpha-2 (Adapter-related protein complex 2 alpha-, 2 subunit) (Alpha-adaptin C) (Adaptor protein complex AP-2 alpha-2, subunit) (Clathrin assembly protein complex 2 alpha-C large chain), (100 kDa coated vesicle protein C) (Plasma membrane adaptor HA2/AP2, adaptin alpha C subunit) (Huntingtin-interacting protein HYPJ).]	Protein Complex
**Q9BZC7**	[ATP-binding cassette sub-family A member 2 (ATP-binding cassette, transporter 2) (ATP-binding cassette 2).]	Protein Complex
**Q9UG63**	[ATP-binding cassette sub-family F member 2 (Iron-inhibited ABC, transporter 2).]	Protein Complex
**P53396**	[ATP-citrate synthase (EC 2.3.3.8) (ATP-citrate (pro-S-)-lyase), (Citrate cleavage enzyme).]	Protein Complex
**P54709**	[Sodium/potassium-transporting ATPase subunit beta-3 (Sodium/potassium-, dependent ATPase beta-3 subunit) (ATPB-3) (CD298 antigen).]	Protein Complex
**P38398**	Breast cancer type 1 susceptibility protein (RING finger protein 53).	Protein Complex
**P11912**	[B-cell antigen receptor complex-associated protein alpha-chain, precursor (Ig-alpha) (MB-1 membrane glycoprotein) (Surface IgM-, associated protein) (Membrane-bound immunoglobulin-associated protein), (CD79a antigen).]	Protein Complex
**P06730**	[Eukaryotic translation initiation factor 4E (eIF4E) (eIF-4E) (mRNA, cap-binding protein) (eIF-4F 25 kDa subunit).]	Protein Complex
**P17302**	[Gap junction alpha-1 protein (Connexin-43) (Cx43) (Gap junction 43 kDa, heart protein).]	Protein Complex
**P48165**	[Gap junction alpha-8 protein (Connexin-50) (Cx50) (Lens fiber protein, MP70).]	Protein Complex
**Q02108**	[Guanylate cyclase soluble subunit alpha-3 (EC 4.6.1.2) (GCS-alpha-3), (Soluble guanylate cyclase large subunit) (GCS-alpha-1).]	Protein Complex
**Q13144**	[Translation initiation factor eIF-2B subunit epsilon (eIF-2B GDP-GTP, exchange factor subunit epsilon).]	Protein Complex
**Q8IUD2**	[ELKS/RAB6-interacting/CAST family member 1 (RAB6-interacting protein, 2) (ERC protein 1).]	Protein Complex
**P02671**	Fibrinogen alpha chain precursor [Contains: Fibrinopeptide A].	Protein Complex
**P80404**	[4-aminobutyrate aminotransferase, mitochondrial precursor, (EC 2.6.1.19) ((S)-3-amino-2-methylpropionate transaminase), (EC 2.6.1.22) (Gamma-amino-N-butyrate transaminase) (GABA, transaminase) (GABA aminotransferase) (GABA-AT) (GABA-T) (L-AIBAT).]	Protein Complex
**P05538**	HLA class II histocompatibility antigen, DX beta chain precursor.	Protein Complex
**P02042**	Hemoglobin subunit delta (Hemoglobin delta chain) (Delta-globin).	Protein Complex
**Q14240**	[Eukaryotic initiation factor 4A-II (EC 3.6.1.-) (ATP-dependent RNA, helicase eIF4A-2) (eIF4A-II) (eIF-4A-II).]	Protein Complex
**P08476**	[Inhibin beta A chain precursor (Activin beta-A chain) (Erythroid, differentiation protein) (EDF).]	Protein Complex
**Q01813**	[6-phosphofructokinase type C (EC 2.7.1.11) (Phosphofructokinase 1), (Phosphohexokinase) (Phosphofructo-1-kinase isozyme C) (PFK-C) (6-, phosphofructokinase, platelet type).]	Protein Complex
**P31321**	cAMP-dependent protein kinase type I-beta regulatory subunit.	Protein Complex
**P22694**	[cAMP-dependent protein kinase, beta-catalytic subunit (EC 2.7.11.11), (PKA C-beta).]	Protein Complex
**P33176**	Kinesin heavy chain (Ubiquitous kinesin heavy chain) (UKHC).	Protein Complex
**P05455**	[Lupus La protein (Sjoegren syndrome type B antigen) (SS-B) (La, ribonucleoprotein) (La autoantigen).]	Protein Complex
**P25391**	Laminin subunit alpha-1 precursor (Laminin A chain).	Protein Complex
**P11047**	Laminin subunit gamma-1 precursor (Laminin B2 chain).	Protein Complex
**O00160**	Myosin-If (Myosin-Ie).	Protein Complex
**P62714**	[Serine/threonine-protein phosphatase 2A catalytic subunit beta isoform, (EC 3.1.3.16) (PP2A-beta).]	Protein Complex
**P27986**	[Phosphatidylinositol 3-kinase regulatory subunit alpha (PI3-kinase p85, subunit alpha) (PtdIns-3-kinase p85-alpha) (PI3K).]	Protein Complex
**O94913**	[Pre-mRNA cleavage complex 2 protein Pcf11 (Pre-mRNA cleavage complex, II protein Pcf11) (Fragment).]	Protein Complex
**P43686**	[26S protease regulatory subunit 6B (Proteasome 26S subunit ATPase 4), (MIP224) (MB67-interacting protein) (TAT-binding protein 7) (TBP-7).]	Protein Complex

**Q16401**	[26S proteasome non-ATPase regulatory subunit 5 (26S proteasome subunit, S5B) (26S protease subunit S5 basic).]	Protein Complex
**P04843**	[Dolichyl-diphosphooligosaccharide--protein glycosyltransferase 67 kDa, subunit precursor (EC 2.4.1.119) (Ribophorin I) (RPN-I).]	Protein Complex
**Q13326**	[Gamma-sarcoglycan (Gamma-SG) (35 kDa dystrophin-associated, glycoprotein) (35DAG).]	Protein Complex
**P46977**	[Dolichyl-diphosphooligosaccharide--protein glycosyltransferase subunit, STT3A (EC 2.4.1.119) (Oligosaccharyl transferase subunit STT3A) (STT3-, A) (B5) (Integral membrane protein 1) (TMC).]	Protein Complex
**Q92993**	[Histone acetyltransferase HTATIP (EC 2.3.1.48) (EC 2.3.1.-) (60 kDa, Tat interactive protein) (Tip60) (HIV-1 Tat interactive protein), (cPLA(2)-interacting protein).]	Protein Complex
**O00472**	RNA polymerase II elongation factor ELL2.	Transcription
**Q12947**	[Forkhead box protein F2 (Forkhead-related protein FKHL6) (Forkhead-, related transcription factor 2) (FREAC-2) (Forkhead-related activator, 2).]	Transcription
**Q15796**	[Mothers against decapentaplegic homolog 2 (SMAD 2) (Mothers against, DPP homolog 2) (Mad-related protein 2) (hMAD-2) (JV18-1) (hSMAD2).]	Transcription
**P49848**	[Transcription initiation factor TFIID subunit 6 (Transcription, initiation factor TFIID 70 kDa subunit) (TAF(II)70) (TAFII-70) (TAFII-, 80) (TAFII80).]	Transcription
**P29374**	[AT-rich interactive domain-containing protein 4A (ARID domain-, containing protein 4A) (Retinoblastoma-binding protein 1) (RBBP-1).]	Transcription
**P21675**	[Transcription initiation factor TFIID subunit 1 (EC 2.7.11.1), (Transcription initiation factor TFIID 250 kDa subunit) (TAF(II)250), (TAFII-250) (TAFII250) (TBP-associated factor 250 kDa) (p250) (Cell, cycle gene 1 protein).]	Transcription
**Q15542**	[Transcription initiation factor TFIID subunit 5 (Transcription, initiation factor TFIID 100 kDa subunit) (TAF(II)100) (TAFII-100), (TAFII100).]	Transcription
**O00268**	[Transcription initiation factor TFIID subunit 4 (TBP-associated factor, 4) (Transcription initiation factor TFIID 135 kDa subunit), (TAF(II)135) (TAFII-135) (TAFII135) (TAFII-130) (TAFII130).]	Transcription
**Q16594**	[Transcription initiation factor TFIID subunit 9 (Transcription, initiation factor TFIID 31 kDa subunit) (TAFII-31) (TAFII-32), (TAFII32) (STAF31/32).]	Transcription
**Q16514**	[Transcription initiation factor TFIID subunit 12 (Transcription, initiation factor TFIID 20/15 kDa subunits) (TAFII-20/TAFII-15), (TAFII20/TAFII15).]	Transcription
**P19801**	[Amiloride-sensitive amine oxidase [copper-containing] precursor, (EC 1.4.3.6) (Diamine oxidase) (DAO) (Amiloride-binding protein) (ABP), (Histaminase) (Kidney amine oxidase) (KAO).]	Vesicle
**O00116**	[Alkyldihydroxyacetonephosphate synthase, peroxisomal precursor, (EC 2.5.1.26) (Alkyl-DHAP synthase) (Alkylglycerone-phosphate, synthase) (Aging-associated gene 5 protein).]	Vesicle
**P35606**	Coatomer subunit beta' (Beta'-coat protein) (Beta'-COP) (p102).	Vesicle
**Q9UBF2**	Coatomer subunit gamma-2 (Gamma-2 coat protein) (Gamma-2 COP).	Vesicle
**O60344**	Endothelin-converting enzyme 2 (EC 3.4.24.71) (ECE-2).	Vesicle
**P10768**	S-formylglutathione hydrolase (EC 3.1.2.12) (FGH) (Esterase D).	Vesicle
**P22888**	[Lutropin-choriogonadotropic hormone receptor precursor (LH/CG-R) (LSH-, R) (Luteinizing hormone receptor) (LHR).]	Vesicle
**Q9Y6K5**	[2'-5'-oligoadenylate synthetase 3 (EC 2.7.7.-) ((2-5')oligo(A), synthetase 3) (2-5A synthetase 3) (p100 OAS) (p100OAS).]	Vesicle
**O14832**	[Phytanoyl-CoA dioxygenase, peroxisomal precursor (EC 1.14.11.18), (Phytanoyl-CoA alpha-hydroxylase) (PhyH) (Phytanic acid oxidase).]	Vesicle
**P02787**	[Serotransferrin precursor (Transferrin) (Siderophilin) (Beta-1-metal-, binding globulin).]	Vesicle

P19224	[UDP-glucuronosyltransferase 1-6 precursor (EC 2.4.1.17) (UDP-, glucuronosyltransferase 1A6) (UDPGT) (UGT1*6) (UGT1-06) (UGT1.6) (UGT-, 1F) (UGT1F) (Phenol-metabolizing UDP-glucuronosyltransferase).]	Vesicle
P53618	Coatomer subunit beta (Beta-coat protein) (Beta-COP).	Vesicle
O15228	[Dihydroxyacetone phosphate acyltransferase (EC 2.3.1.42) (DHAP-AT), (DAP-AT) (Glycerone-phosphate O-acyltransferase) (Acyl-, CoA:dihydroxyacetonephosphateacyltransferase).]	Vesicle
O95822	Malonyl-CoA decarboxylase, mitochondrial precursor (EC 4.1.1.9) (MCD).	Vesicle
O14880	[Microsomal glutathione S-transferase 3 (EC 2.5.1.18) (Microsomal GST-, 3) (Microsomal GST-III).]	Vesicle
O75146	[Huntingtin-interacting protein 1-related protein (Hip1-related) (Hip, 12).]	Vesicle
O60333	Kinesin-like protein KIF1B (Klp).	Vesicle
Q99698	Lysosomal-trafficking regulator (Beige homolog).	Vesicle
P22307	[Nonspecific lipid-transfer protein (EC 2.3.1.176) (Propanoyl-CoA C-, acyltransferase) (NSL-TP) (Sterol carrier protein 2) (SCP-2) (Sterol, carrier protein X) (SCP-X) (SCP-chi) (SCPX).]	Vesicle
Q9UKG9	Peroxisomal carnitine O-octanoyltransferase (EC 2.3.1.137) (COT).	Vesicle
O94855	Protein transport protein Sec24D (SEC24-related protein D).	Vesicle
O14994	Synapsin-3 (Synapsin III).	Vesicle
P21579	Synaptotagmin-1 (Synaptotagmin I) (SytI) (p65).	Vesicle
P07550	[Beta-2 adrenergic receptor (Beta-2 adrenoceptor) (Beta-2, adrenoreceptor).]	Vesicle
O15254	[Acyl-coenzyme A oxidase 3, peroxisomal (EC 1.3.3.6) (Pristanoyl-CoA, oxidase) (Branched-chain acyl-CoA oxidase) (BRCACox).]	Vesicle
P08962	[CD63 antigen (Melanoma-associated antigen ME491) (Ocular melanoma-, associated antigen) (OMA81H) (Granulophysin) (Tetraspanin-30) (Tspan-, 30).]	Vesicle
P48444	Coatomer subunit delta (Delta-coat protein) (Delta-COP) (Archain).	Vesicle
P33261	[Cytochrome P450 2C19 (EC 1.14.13.80) ((R)-limonene 6-monooxygenase), (EC 1.14.13.48) ((S)-limonene 6-monooxygenase) (EC 1.14.13.49) ((S)-, limonene 7-monooxygenase) (CYPIIC19) (P450-11A) (Mephenytoin 4-, hydroxylase) (CYPIIC17) (P450-254C).]	Vesicle
Q14534	Squalene monooxygenase (EC 1.14.99.7) (Squalene epoxidase) (SE).	Vesicle
P52566	Rho GDP-dissociation inhibitor 2 (Rho GDI 2) (Rho-GDI beta) (Ly-GDI).	Vesicle
Q9UJM8	Hydroxyacid oxidase 1 (EC 1.1.3.15) (HAOX1) (Glycolate oxidase) (GOX).	Vesicle
Q14656	Transmembrane protein 187 (Protein ITBA1).	Vesicle
P13473	[Lysosome-associated membrane glycoprotein 2 precursor (LAMP-2) (CD107b, antigen).]	Vesicle
P11717	[Cation-independent mannose-6-phosphate receptor precursor (CI Man-6-P, receptor) (CI-MPR) (M6PR) (Insulin-like growth factor 2 receptor), (Insulin-like growth factor II receptor) (IGF-II receptor) (M6P/IGF2, receptor) (M6P/IGF2R) (300 kDa mannose 6-phosphate receptor) (MPR 300), (MPR300) (CD222 antigen).]	Vesicle
O95486	Protein transport protein Sec24A (SEC24-related protein A) (Fragment).	Vesicle
P48723	[Stress 70 protein chaperone microsome-associated 60 kDa protein, precursor (Microsomal stress 70 protein ATPase core).]	Vesicle
Q99805	Transmembrane 9 superfamily protein member 2 precursor (p76).	Vesicle
P47901	[Vasopressin V1b receptor (V1bR) (AVPR V1b) (Vasopressin V3 receptor), (AVPR V3) (Antidiuretic hormone receptor 1b).]	Vesicle

Uniprot Number	Data 2 Protein Name	Component_CATEGORY
P16401	Histone H1.5 (Histone H1a).	Chromosomal
P46100	[Transcriptional regulator ATRX (EC 3.6.1.-) (ATP-dependent helicase, ATRX) (X-linked helicase II) (X-linked nuclear protein) (XNP) (Znf-, HX).]	Chromosomal
P49736	[DNA replication licensing factor MCM2 (Minichromosome maintenance, protein 2 homolog) (Nuclear protein BM28).]	Chromosomal
P55209	Nucleosome assembly protein 1-like 1 (NAP-1-related protein) (hNRP).	Chromosomal
Q92900	[Regulator of nonsense transcripts 1 (EC 3.6.1.-) (ATP-dependent, helicase RENT1) (Nonsense mRNA reducing factor 1) (NORF1) (Up-, frameshift suppressor 1 homolog) (hUpf1).]	Chromosomal
P03372	Estrogen receptor (ER) (Estradiol receptor) (ER-alpha).	Chromosomal
P33993	DNA replication licensing factor MCM7 (CDC47 homolog) (P1.1-MCM3).	Chromosomal
Q02224	Centromeric protein E (CENP-E).	Chromosomal
Q99811	[Paired mesoderm homeobox protein 2 (PRX-2) (Paired-related homeobox, protein 2).]	Chromosomal
O14757	Serine/threonine-protein kinase Chk1 (EC 2.7.11.1).	Chromosomal
O14924	Regulator of G-protein signaling 12 (RGS12).	Chromosomal
P16403	Histone H1.2 (Histone H1d).	Chromosomal
P18754	[Regulator of chromosome condensation (Chromosome condensation protein, 1) (Cell cycle regulatory protein).]	Chromosomal
P42704	[Leucine-rich PPR motif-containing protein, mitochondrial precursor, (130 kDa leucine-rich protein) (LRP 130) (GP130).]	Chromosomal
P04198	N-myc proto-oncogene protein.	Chromosomal
P62807	[Histone H2B type 1-C/E/F/G/I (H2B.a/g/h/k/l) (H2B.1 A) (H2B/a) (H2B/g), (H2B/h) (H2B/k) (H2B/l).]	Chromosomal
P83916	[Chromobox protein homolog 1 (Heterochromatin protein 1 homolog beta), (HP1 beta) (Modifier 1 protein) (M31) (Heterochromatin protein p25), (HP1Hsbeta) (p25beta).]	Chromosomal
Q13111	[Chromatin assembly factor 1 subunit A (CAF-1 subunit A) (Chromatin, assembly factor I p150 subunit) (CAF-I 150 kDa subunit) (CAF-Ip150).]	Chromosomal
Q99457	Nucleosome assembly protein 1-like 3.	Chromosomal
Q99878	Histone H2A type 1-J.	Chromosomal
Q9UHN1	[DNA polymerase subunit gamma-2, mitochondrial precursor (EC 2.7.7.7), (Mitochondrial DNA polymerase accessory subunit) (PolG-beta) (MtPolB), (DNA polymerase gamma accessory 55 kDa subunit) (p55).]	Chromosomal
P06400	Retinoblastoma-associated protein (PP110) (P105-RB) (RB).	Chromosomal
O14746	[Telomerase reverse transcriptase (EC 2.7.7.49) (Telomerase catalytic, subunit) (HEST2) (Telomerase-associated protein 2) (TP2).]	Chromosomal
P09430	Spermatid nuclear transition protein 1 (STP-1) (TP-1).	Chromosomal
P07199	Major centromere autoantigen B (Centromere protein B) (CENP-B).	Chromosomal
P35452	Homeobox protein Hox-D12 (Hox-4H).	Chromosomal
Q12948	[Forkhead box protein C1 (Forkhead-related protein FKHL7) (Forkhead-, related transcription factor 3) (FREAC-3).]	Chromosomal
Q13112	[Chromatin assembly factor 1 subunit B (CAF-1 subunit B) (Chromatin, assembly factor I p60 subunit) (CAF-I 60 kDa subunit) (CAF-Ip60) (M-, phase phosphoprotein 7).]	Chromosomal
Q9UNS1	Protein timeless homolog (hTIM).	Chromosomal
O00291	Huntingtin-interacting protein 1 (HIP-I).	Cytoskeletal

O43602	[Neuronal migration protein doublecortin (Lissencephalin-X) (Lis-X), (Doublin).]	Cytoskeletal
O75366	Advillin (p92).	Cytoskeletal
P11137	Microtubule-associated protein 2 (MAP 2) (MAP-2).	Cytoskeletal
P20929	Nebulin.	Cytoskeletal
P46821	[Microtubule-associated protein 1B (MAP 1B) [Contains: MAP1 light chain, LC1].]	Cytoskeletal
P49418	Amphiphysin.	Cytoskeletal
P52565	Rho GDP-dissociation inhibitor 1 (Rho GDI 1) (Rho-GDI alpha).	Cytoskeletal
P53814	Smoothelin.	Cytoskeletal
Q14790	[Caspase-8 precursor (EC 3.4.22.61) (CASP-8) (ICE-like apoptotic, protease 5) (MORT1-associated CED-3 homolog) (MACH) (FADD-homologous, ICE/CED-3-like protease) (FADD-like ICE) (FLICE) (Apoptotic cysteine, protease) (Apoptotic protease Mch-5) (CAP4) [Contains: Caspase-8, subunit p18; Caspase-8 subunit p10].]	Cytoskeletal
Q15814	Tubulin-specific chaperone C (Tubulin-folding cofactor C) (CFC).	Cytoskeletal
Q16825	[Tyrosine-protein phosphatase non-receptor type 21 (EC 3.1.3.48), (Protein-tyrosine phosphatase D1).]	Cytoskeletal
Q92574	Hamartin (Tuberous sclerosis 1 protein).	Cytoskeletal
Q92838	[Ectodysplasin-A (Ectodermal dysplasia protein) (EDA protein), [Contains: Ectodysplasin-A, membrane form; Ectodysplasin-A, secreted, form].]	Cytoskeletal
P02730	[Band 3 anion transport protein (Anion exchange protein 1) (AE 1), (Solute carrier family 4 member 1) (CD233 antigen).]	Cytoskeletal
P06396	[Gelsolin precursor (Actin-depolymerizing factor) (ADF) (Brevin), (AGEL).]	Cytoskeletal
P27105	[Erythrocyte band 7 integral membrane protein (Stomatin) (Protein, 7.2b).]	Cytoskeletal
P27816	Microtubule-associated protein 4 (MAP 4).	Cytoskeletal
P35240	[Merlin (Moesin-ezrin-radixin-like protein) (Neurofibromin-2), (Schwannomin) (Schwannomerlin).]	Cytoskeletal
P58107	Epiplakin (450 kDa epidermal antigen).	Cytoskeletal
P60981	Destrin (Actin-depolymerizing factor) (ADF).	Cytoskeletal
Q05682	Caldesmon (CDM).	Cytoskeletal
Q07283	Trichohyalin.	Cytoskeletal
Q9C0H6	Kelch-like protein 4.	Cytoskeletal
O14936	[Peripheral plasma membrane protein CASK (EC 2.7.11.1) (hCASK), (Calcium/calmodulin-dependent serine protein kinase) (Lin-2 homolog).]	Cytoskeletal
O15195	Villin-like protein.	Cytoskeletal
P19338	Nucleolin (Protein C23).	Cytoskeletal
P49368	T-complex protein 1 subunit gamma (TCP-1-gamma) (CCT-gamma) (hTRiC5).	Cytoskeletal
P50552	Vasodilator-stimulated phosphoprotein (VASP).	Cytoskeletal
P53355	Death-associated protein kinase 1 (EC 2.7.11.1) (DAP kinase 1).	Cytoskeletal
Q13459	Myosin-IXb (Unconventional myosin-9b).	Cytoskeletal
Q14247	Src substrate cortactin (Amplaxin) (Oncogene EMS1).	Cytoskeletal
Q16658	Fascin (Singed-like protein) (55 kDa actin-bundling protein) (p55).	Cytoskeletal
Q92747	Actin-related protein 2/3 complex subunit 1A (SOP2-like protein).	Cytoskeletal
P07437	Tubulin beta chain (Tubulin beta-5 chain).	Cytoskeletal

P26232	Catenin alpha-2 (Alpha-catenin-related protein) (Alpha N-catenin).	Cytoskeletal
P62330	ADP-ribosylation factor 6.	Cytoskeletal
P78559	[Microtubule-associated protein 1A (MAP 1A) (Proliferation-related, protein p80) [Contains: MAP1 light chain LC2].]	Cytoskeletal
Q92974	[Rho/Rac guanine nucleotide exchange factor 2 (GEF-H1 protein), (Proliferating cell nucleolar antigen p40).]	Cytoskeletal
Q9UBN4	[Short transient receptor potential channel 4 (TrpC4) (Trp-related, protein 4) (hTrp-4) (hTrp4).]	Cytoskeletal
Q9ULV4	Coronin-1C (Coronin-3) (hCRNN4).	Cytoskeletal
Q9Y2M5	[Kelch-like protein 20 (Kelch-like ECT2-interacting protein) (Kelch-, like protein X).]	Cytoskeletal
O00499	[Myc box-dependent-interacting protein 1 (Bridging integrator 1), (Amphiphysin-like protein) (Amphiphysin II) (Box-dependent myc-, interacting protein 1).]	Cytoskeletal
O14926	Fascin-2 (Retinal fascin).	Cytoskeletal
O15553	Pyrin (Marenostrin).	Cytoskeletal
O60437	[Periplakin (195 kDa cornified envelope precursor protein) (190 kDa, paraneoplastic pemphigus antigen).]	Cytoskeletal
P10916	[Myosin regulatory light chain 2, ventricular/cardiac muscle isoform, (MLC-2) (MLC-2v).]	Cytoskeletal
P21333	[Filamin-A (Alpha-filamin) (Filamin-1) (Endothelial actin-binding, protein) (Actin-binding protein 280) (ABP-280) (Non-muscle filamin).]	Cytoskeletal
P46939	Utrophin (Dystrophin-related protein 1) (DRP1) (DRP).	Cytoskeletal
P50748	[Kinetochore-associated protein 1 (Rough deal homolog) (hRod) (HsROD), (Rod).]	Cytoskeletal
P54840	Glycogen [starch] synthase, liver (EC 2.4.1.11).	Cytoskeletal
Q13563	[Polycystin-2 (Polycystic kidney disease 2 protein homolog) (Autosomal, dominant polycystic kidney disease type II protein) (Polycystwin), (R48321).]	Cytoskeletal
Q13576	Ras GTPase-activating-like protein IQGAP2.	Cytoskeletal
O95198	Kelch-like protein 2 (Actin-binding protein Mayven).	Cytoskeletal
O95793	Double-stranded RNA-binding protein Staufen homolog 1.	Cytoskeletal
P80723	[Brain acid soluble protein 1 (BASP1 protein) (Neuronal axonal membrane, protein NAP-22) (22 kDa neuronal tissue-enriched acidic protein).]	Cytoskeletal
P35080	Profilin-2 (Profilin II).	Cytoskeletal
P78352	[Disks large homolog 4 (Postsynaptic density protein 95) (PSD-95), (Synapse-associated protein 90) (SAP90).]	Cytoskeletal
Q05397	[Focal adhesion kinase 1 (EC 2.7.10.2) (FADK 1) (pp125FAK) (Protein-, tyrosine kinase 2).]	Cytoskeletal
Q08495	Dematin (Erythrocyte membrane protein band 4.9).	Cytoskeletal
Q99426	[Tubulin folding cofactor B (Tubulin-specific chaperone B), (Cytoskeleton-associated protein 1) (Cytoskeleton-associated protein, CKAPI).]	Cytoskeletal
Q99439	Calponin-2 (Calponin H2, smooth muscle) (Neutral calponin).	Cytoskeletal
Q99867	Tubulin beta-4q chain.	Cytoskeletal
Q9NQ38	[Serine protease inhibitor Kazal-type 5 precursor (Lympho-epithelial, Kazal-type-related inhibitor) (LEKTI) [Contains: Hemofiltrate peptide, HF6478; Hemofiltrate peptide HF7665].]	Cytoskeletal
Q9UM54	Myosin-VI (Unconventional myosin VI).	Cytoskeletal
O00410	Importin beta-3 (Karyopherin beta-3) (Ran-binding protein 5) (RanBP5).	Cytosolic
O00571	[ATP-dependent RNA helicase DDX3X (EC 3.6.1.-) (DEAD box protein 3, X-, chromosomal) (Helicase-like protein 2) (HLP2) (DEAD box, X isoform).]	Cytosolic

O00750	[Phosphatidylinositol-4-phosphate 3-kinase C2 domain-containing beta, polypeptide (EC 2.7.1.154) (Phosphoinositide 3-Kinase-C2-beta), (PtdIns-3-kinase C2 beta) (PI3K-C2beta) (C2-PI3K).]	Cytosolic
O14733	[Dual specificity mitogen-activated protein kinase kinase 7, (EC 2.7.12.2) (MAP kinase kinase 7) (MAPKK 7) (MAPK/ERK kinase 7), (JNK-activating kinase 2) (c-Jun N-terminal kinase kinase 2) (JNK, kinase 2) (JNKK 2).]	Cytosolic
O14787	Transportin-2 (Karyopherin beta-2b).	Cytosolic
O15111	[Inhibitor of nuclear factor kappa-B kinase subunit alpha, (EC 2.7.11.10) (I kappa-B kinase alpha) (IkBKA) (IKK-alpha) (IKK-A), (IkappaB kinase) (I-kappa-B kinase 1) (IKK1) (Conserved helix-loop-, helix ubiquitous kinase) (Nuclear factor NF-kappa-B inhibitor kinase, alpha) (NFKBIKA).]	Cytosolic
O15296	[Arachidonate 15-lipoxygenase type II (EC 1.13.11.33) (15-LOX-2) (15-, lipoxygenase 2).]	Cytosolic
O15360	Fanconi anemia group A protein (Protein FACA).	Cytosolic
O15530	3-phosphoinositide-dependent protein kinase 1 (EC 2.7.11.1) (hPDK1).	Cytosolic
O43237	[Cytoplasmic dynein 1 light intermediate chain 2 (Dynein light, intermediate chain 2, cytosolic) (LIC53/55) (LIC-2).]	Cytosolic
O43291	[Kunitz-type protease inhibitor 2 precursor (Hepatocyte growth factor, activator inhibitor type 2) (HAI-2) (Placental bikunin).]	Cytosolic
O43765	[Small glutamine-rich tetratricopeptide repeat-containing protein A, (Vpu-binding protein) (UBP).]	Cytosolic
O60318	80 kDa MCM3-associated protein (Protein GANP).	Cytosolic
O60716	[Catenin delta-1 (p120 catenin) (p120(ctn)) (Cadherin-associated Src, substrate) (CAS) (p120(cas)).]	Cytosolic
O75312	Zinc finger protein ZPR1 (Zinc finger protein 259).	Cytosolic
P07900	[Heat shock protein HSP 90-alpha (HSP 86) (Renal carcinoma antigen NY-, REN-38).]	Cytosolic
P08238	Heat shock protein HSP 90-beta (HSP 84) (HSP 90).	Cytosolic
P08670	Vimentin.	Cytosolic
P09651	[Heterogeneous nuclear ribonucleoprotein A1 (Helix-destabilizing, protein) (Single-strand RNA-binding protein) (hnRNP core protein A1).]	Cytosolic
P10071	Zinc finger protein GLI3.	Cytosolic
P10275	Androgen receptor (Dihydrotestosterone receptor).	Cytosolic
P12272	[Parathyroid hormone-related protein precursor (PTH-rP) (PTHrP), [Contains: PTHrP[1-36]; PTHrP[38-94]; Osteostatin (PTHrP[107-139])].]	Cytosolic
P14598	[Neutrophil cytosol factor 1 (NCF-1) (Neutrophil NADPH oxidase factor, 1) (47 kDa neutrophil oxidase factor) (p47-phox) (NCF-47K) (47 kDa, autosomal chronic granulomatous disease protein) (Nox organizer 2), (Nox-organizing protein 2) (SH3 and PX domain-containing protein 1A).]	Cytosolic
P15428	[15-hydroxyprostaglandin dehydrogenase [NAD+] (EC 1.1.1.141) (PGDH), (Prostaglandin dehydrogenase 1).]	Cytosolic
P16989	[DNA-binding protein A (Cold shock domain-containing protein A), (Single-strand DNA-binding protein NF-GMB).]	Cytosolic
P17931	[Galectin-3 (Galactose-specific lectin 3) (Mac-2 antigen) (IgE-binding, protein) (35 kDa lectin) (Carbohydrate-binding protein 35) (CBP 35), (Laminin-binding protein) (Lectin L-29) (L-31) (Galactoside-binding, protein) (GALBP).]	Cytosolic
P19835	[Bile salt-activated lipase precursor (EC 3.1.1.3) (EC 3.1.1.13) (BAL), (Bile salt-stimulated lipase) (BSSL) (Carboxyl ester lipase) (Sterol, esterase) (Cholesterol esterase) (Pancreatic lysophospholipase).]	Cytosolic
P20396	[Thyroliberin precursor [Contains: Prothyroliberin; Thyroliberin, (Thyrotropin-releasing hormone) (TRH) (Thyrotropin-releasing factor), (TRF) (TSH-releasing factor) (Protirelin)].]	Cytosolic
P42677	40S ribosomal protein S27 (Metallopan-stimulin 1) (MPS-1).	Cytosolic
P42771	[Cyclin-dependent kinase inhibitor 2A, isoforms 1/2/3 (Cyclin-dependent, kinase 4 inhibitor A) (CDK4I) (p16-INK4) (p16-INK4a) (p16INK4A), (Multiple tumor suppressor 1) (MTS1).]	Cytosolic

P43487	[Ran-specific GTPase-activating protein (Ran-binding protein 1), (RanBP1).]	Cytosolic
P45877	[Peptidyl-prolyl cis-trans isomerase C (EC 5.2.1.8) (PPIase) (Rotamase), (Cyclophilin C).]	Cytosolic
P47712	[Cytosolic phospholipase A2 (cPLA2) (Phospholipase A2 group IVA), [Includes: Phospholipase A2 (EC 3.1.1.4) (Phosphatidylcholine 2-, acylhydrolase); Lysophospholipase (EC 3.1.1.5)].]	Cytosolic
P48147	[Prolyl endopeptidase (EC 3.4.21.26) (Post-proline cleaving enzyme), (PE).]	Cytosolic
P49585	[Choline-phosphate cytidylyltransferase A (EC 2.7.7.15), (Phosphorylcholine transferase A) (CTP:phosphocholine, cytidylyltransferase A) (CT A) (CCT A) (CCT-alpha).]	Cytosolic
P49590	[Probable histidyl-tRNA synthetase, mitochondrial precursor, (EC 6.1.1.21) (Histidine--tRNA ligase) (HisRS) (Histidine--tRNA, ligase-like).]	Cytosolic
P50452	[Serpin B8 (Cytoplasmic antiproteinase 2) (CAP-2) (CAP2) (Proteinase, inhibitor 8).]	Cytosolic
P50579	[Methionine aminopeptidase 2 (EC 3.4.11.18) (MetAP 2) (Peptidase M 2), (Initiation factor 2-associated 67 kDa glycoprotein) (p67) (p67eIF2).]	Cytosolic
P50914	60S ribosomal protein L14 (CAG-ISL 7).	Cytosolic
P51003	[Poly(A) polymerase alpha (EC 2.7.7.19) (PAP) (Polynucleotide, adenylyltransferase alpha).]	Cytosolic
P51610	[Host cell factor (HCF) (HCF-1) (C1 factor) (VP16 accessory protein), (VCAF) (CFF) [Contains: HCF N-terminal chain 1; HCF N-terminal chain, 2; HCF N-terminal chain 3; HCF N-terminal chain 4; HCF N-terminal, chain 5; HCF N-terminal chain 6; HCF C-terminal chain 1; HCF C-, terminal chain 2; HCF C-terminal chain 3; HCF C-terminal chain 4; HCF, C-terminal chain 5; HCF C-terminal chain 6].]	Cytosolic
P51858	[Hepatoma-derived growth factor (HDGF) (High-mobility group protein 1-, like 2) (HMG-1L2).]	Cytosolic
P54252	[Ataxin-3 (EC 3.4.22.-) (Machado-Joseph disease protein 1), (Spinocerebellar ataxia type 3 protein).]	Cytosolic
P55060	[Exportin-2 (Exp2) (Importin-alpha re-exporter) (Chromosome segregation, 1-like protein) (Cellular apoptosis susceptibility protein).]	Cytosolic
P55265	[Double-stranded RNA-specific adenosine deaminase (EC 3.5.4.-) (DRADA), (136 kDa double-stranded RNA-binding protein) (P136) (K88DSRBP), (Interferon-inducible protein 4) (IFI-4).]	Cytosolic
P55786	Puromycin-sensitive aminopeptidase (EC 3.4.11.-) (PSA).	Cytosolic
P57678	Component of gems 4 (Gemin-4) (p97).	Cytosolic
Q13469	[Nuclear factor of activated T-cells, cytoplasmic 2 (T-cell, transcription factor NFAT1) (NFAT pre-existing subunit) (NF-ATp).]	Cytosolic
Q13588	GRB2-related adapter protein.	Cytosolic
Q13596	Sorting nexin-1.	Cytosolic
Q13625	[Apoptosis-stimulating of p53 protein 2 (Tumor suppressor p53-binding, protein 2) (p53-binding protein 2) (p53BP2) (53BP2) (Bcl2-binding, protein) (Bbp) (Renal carcinoma antigen NY-REN-51).]	Cytosolic
Q13867	Bleomycin hydrolase (EC 3.4.22.40) (BLM hydrolase) (BMH) (BH).	Cytosolic
Q13895	Bystin.	Cytosolic
Q14028	[Cyclic nucleotide-gated cation channel 4 (CNG channel 4) (CNG-4), (CNG4) (Cyclic nucleotide-gated cation channel modulatory subunit).]	Cytosolic
Q14203	[Dynactin subunit 1 (150 kDa dynein-associated polypeptide) (DP-150), (DAP-150) (p150-glued) (p135).]	Cytosolic
Q14691	DNA replication complex GINS protein PSF1.	Cytosolic
Q14914	[NADP-dependent leukotriene B4 12-hydroxydehydrogenase (EC 1.3.1.74), (15-oxoprostaglandin 13-reductase) (EC 1.3.1.48).]	Cytosolic
Q14978	[Nucleolar phosphoprotein p130 (Nucleolar 130 kDa protein) (140 kDa, nucleolar phosphoprotein) (Nopp140) (Nucleolar and coiled-body, phosphoprotein 1).]	Cytosolic
Q15013	MAD2L1-binding protein (Caught by MAD2 protein).	Cytosolic

Q15052	[Rho guanine nucleotide exchange factor 6 (Rac/Cdc42 guanine nucleotide, exchange factor 6) (PAK-interacting exchange factor alpha) (Alpha-Pix), (COOL-2).]	Cytosolic
Q15080	[Neutrophil cytosol factor 4 (NCF-4) (Neutrophil NADPH oxidase factor, 4) (p40-phox) (p40phox) (SH3 and PX domain-containing protein 4).]	Cytosolic
Q15366	Poly(rC)-binding protein 2 (Alpha-CP2) (hnRNP-E2).	Cytosolic
Q15628	[Tumor necrosis factor receptor type 1-associated DEATH domain protein, (TNFR1-associated DEATH domain protein) (TNFRSF1A-associated via death, domain).]	Cytosolic
Q15646	[59 kDa 2'-5'-oligoadenylate synthetase-like protein (p59 OASL), (p59OASL) (Thyroid receptor-interacting protein 14) (TRIP-14).]	Cytosolic
Q92540	Protein SMG7 (SMG-7 homolog) (EST1-like protein C).	Cytosolic
Q92604	Acyl-CoA:lysophosphatidylglycerol acyltransferase 1 (EC 2.3.1.-).	Cytosolic
Q92793	CREB-binding protein (EC 2.3.1.48).	Cytosolic
O76038	Secretagogin.	Cytosolic
O94833	[Bullous pemphigoid antigen 1, isoforms 6/9/10 (Trabeculin-beta), (Bullous pemphigoid antigen) (BPA) (Hemidesmosomal plaque protein), (Dystonia musculorum protein) (Dystonin).]	Cytosolic
O94903	Proline synthetase co-transcribed bacterial homolog protein.	Cytosolic
O95544	NAD kinase (EC 2.7.1.23) (Poly(P)/ATP NAD kinase).	Cytosolic
O95757	[Heat shock 70 kDa protein 4L (Osmotic stress protein 94) (Heat shock, 70-related protein APG-1).]	Cytosolic
P25815	Protein S100-P (S100 calcium-binding protein P).	Cytosolic
P01229	[Lutropin subunit beta precursor (Luteinizing hormone subunit beta), (LSH-beta) (LSH-B) (LH-B) (Lutropin beta chain).]	Cytosolic
P04066	[Tissue alpha-L-fucosidase precursor (EC 3.2.1.51) (Alpha-L-fucosidase, I) (Alpha-L-fucoside fucohydrolase).]	Cytosolic
P04424	Argininosuccinate lyase (EC 4.3.2.1) (Arginosuccinase) (ASAL).	Cytosolic
P04637	[Cellular tumor antigen p53 (Tumor suppressor p53) (Phosphoprotein p53), (Antigen NY-CO-13).]	Cytosolic
P06732	Creatine kinase M-type (EC 2.7.3.2) (Creatine kinase M chain) (M-CK).	Cytosolic
P07741	Adenine phosphoribosyltransferase (EC 2.4.2.7) (APRT).	Cytosolic
P22105	Tenascin-X precursor (TN-X) (Hexabrachion-like protein).	Cytosolic
P23219	[Prostaglandin G/H synthase 1 precursor (EC 1.14.99.1) (Cyclooxygenase-, 1) (COX-1) (Prostaglandin-endoperoxide synthase 1) (Prostaglandin H2, synthase 1) (PGH synthase 1) (PGHS-1) (PHS 1).]	Cytosolic
P23528	Cofilin-1 (Cofilin, non-muscle isoform) (18 kDa phosphoprotein) (p18).	Cytosolic
P26639	[Threonyl-tRNA synthetase, cytoplasmic (EC 6.1.1.3) (Threonine--tRNA, ligase) (ThrRS).]	Cytosolic
P28329	[Choline O-acetyltransferase (EC 2.3.1.6) (CHOACTase) (Choline, acetylase) (ChAT).]	Cytosolic
P29074	[Tyrosine-protein phosphatase non-receptor type 4 (EC 3.1.3.48), (Protein-tyrosine phosphatase MEG1) (PTPase-MEG1) (MEG).]	Cytosolic
P30626	Sorcin (22 kDa protein) (CP-22) (V19).	Cytosolic
P30740	[Leukocyte elastase inhibitor (LEI) (Serpin B1) (Monocyte/neutrophil, elastase inhibitor) (M/NEI) (EI).]	Cytosolic
P31350	[Ribonucleoside-diphosphate reductase subunit M2 (EC 1.17.4.1), (Ribonucleotide reductase small subunit) (Ribonucleotide reductase, small chain).]	Cytosolic
P31947	14-3-3 protein sigma (Stratifin) (Epithelial cell marker protein 1).	Cytosolic
P32121	Beta-arrestin-2 (Arrestin beta 2).	Cytosolic
P34947	[G protein-coupled receptor kinase 5 (EC 2.7.11.16) (G protein-coupled, receptor kinase GRK5).]	Cytosolic

P35354	[Prostaglandin G/H synthase 2 precursor (EC 1.14.99.1) (Cyclooxygenase-, 2) (COX-2) (Prostaglandin-endoperoxide synthase 2) (Prostaglandin H2, synthase 2) (PGH synthase 2) (PGHS-2) (PHS II).]	Cytosolic
P35754	Glutaredoxin-1 (Thioltransferase-1) (TTase-1).	Cytosolic
P36871	Phosphoglucomutase-1 (EC 5.4.2.2) (Glucose phosphomutase 1) (PGM 1).	Cytosolic
P39023	60S ribosomal protein L3 (HIV-1 TAR RNA-binding protein B) (TARBP-B).	Cytosolic
P40222	Alpha-taxilin.	Cytosolic
P41214	Ligatin (Hepatocellular carcinoma-associated antigen 56).	Cytosolic
P41240	[Tyrosine-protein kinase CSK (EC 2.7.10.2) (C-SRC kinase) (Protein-, tyrosine kinase CYL).]	Cytosolic
P41279	[Mitogen-activated protein kinase kinase kinase 8 (EC 2.7.11.25) (COT, proto-oncogene serine/threonine-protein kinase) (C-COT) (Cancer Osaka, thyroid oncogene).]	Cytosolic
P61202	[COP9 signalosome complex subunit 2 (Signalosome subunit 2) (SGN2), (JAB1-containing signalosome subunit 2) (Thyroid receptor-interacting, protein 15) (TRIP-15) (Alien homolog).]	Cytosolic
P61247	40S ribosomal protein S3a.	Cytosolic
P62081	40S ribosomal protein S7.	Cytosolic
P62195	[26S protease regulatory subunit 8 (Proteasome 26S subunit ATPase 5), (Proteasome subunit p45) (p45/SUG) (Thyroid hormone receptor-, interacting protein 1) (TRIP1).]	Cytosolic
P62280	40S ribosomal protein S11.	Cytosolic
P62324	Protein BTG1 (B-cell translocation gene 1 protein).	Cytosolic
P62495	[Eukaryotic peptide chain release factor subunit 1 (eRF1) (Eukaryotic, release factor 1) (TB3-1) (Protein Cl1).]	Cytosolic
P62854	40S ribosomal protein S26.	Cytosolic
P78371	T-complex protein 1 subunit beta (TCP-1-beta) (CCT-beta).	Cytosolic
P78417	Glutathione transferase omega-1 (EC 2.5.1.18) (GSTO 1-1).	Cytosolic
Q01581	[Hydroxymethylglutaryl-CoA synthase, cytoplasmic (EC 2.3.3.10) (HMG-CoA, synthase) (3-hydroxy-3-methylglutaryl coenzyme A synthase).]	Cytosolic
Q02878	[60S ribosomal protein L6 (TAX-responsive enhancer element-binding, protein 107) (TAXREB107) (Neoplasm-related protein C140).]	Cytosolic
Q02952	[A-kinase anchor protein 12 (A-kinase anchor protein 250 kDa) (AKAP, 250) (Gravin) (Myasthenia gravis autoantigen).]	Cytosolic
Q04760	[Lactoylglutathione lyase (EC 4.4.1.5) (Methylglyoxalase), (Aldoketomutase) (Glyoxalase I) (Glx I) (Ketone-aldehyde mutase) (S-D-, lactoylglutathione methylglyoxal lyase).]	Cytosolic
Q05209	[Tyrosine-protein phosphatase non-receptor type 12 (EC 3.1.3.48), (Protein-tyrosine phosphatase G1) (PTPG1) (PTP-PEST).]	Cytosolic
Q05469	Hormone-sensitive lipase (EC 3.1.1.79) (HSL).	Cytosolic
Q06141	[Regenerating islet-derived protein 3 alpha precursor (Reg III-alpha), (Pancreatitis-associated protein 1).]	Cytosolic
Q07866	Kinesin light chain 1 (KLC 1).	Cytosolic
Q08499	[cAMP-specific 3',5'-cyclic phosphodiesterase 4D (EC 3.1.4.17) (DPDE3), (PDE43).]	Cytosolic
Q92973	[Transportin-1 (Importin beta-2) (Karyopherin beta-2) (M9 region, interaction protein) (MIP).]	Cytosolic
Q99259	[Glutamate decarboxylase 1 (EC 4.1.1.15) (Glutamate decarboxylase 67, kDa isoform) (GAD-67) (67 kDa glutamic acid decarboxylase).]	Cytosolic
Q99469	[SH3 and cysteine-rich domain-containing protein (SRC homology 3 and, cysteine-rich domain protein).]	Cytosolic
Q99549	M-phase phosphoprotein 8.	Cytosolic

Q99873	[Protein arginine N-methyltransferase 1 (EC 2.1.1.-) (Interferon, receptor 1-bound protein 4).]	Cytosolic
Q99956	[Dual specificity protein phosphatase 9 (EC 3.1.3.48) (EC 3.1.3.16), (Mitogen-activated protein kinase phosphatase 4) (MAP kinase, phosphatase 4) (MKP-4).]	Cytosolic
Q9NQ66	[1-phosphatidylinositol-4,5-bisphosphate phosphodiesterase beta-1, (EC 3.1.4.11) (Phosphoinositide phospholipase C) (Phospholipase C-, beta-1) (PLC-beta-1) (PLC-I) (PLC-154).]	Cytosolic
Q9NRA0	Sphingosine kinase 2 (EC 2.7.1.-) (SK 2) (SPK 2).	Cytosolic
Q9NSD9	[Phenylalanyl-tRNA synthetase beta chain (EC 6.1.1.20) (Phenylalanine--, tRNA ligase beta chain) (PheRS).]	Cytosolic
Q9UHI6	[Probable ATP-dependent RNA helicase DDX20 (EC 3.6.1.-) (DEAD box, protein 20) (DEAD box protein DP 103) (Component of gems 3) (Gemin-3).]	Cytosolic
Q9UKL6	[Phosphatidylcholine transfer protein (PC-TP) (StAR-related lipid, transfer protein 2) (StARD2) (START domain-containing protein 2).]	Cytosolic
Q9UN86	[Ras GTPase-activating protein-binding protein 2 (G3BP-2) (GAP SH3, domain-binding protein 2).]	Cytosolic
Q9UPN3	[Microtubule-actin cross-linking factor 1, isoforms 1/2/3/5 (Actin, cross-linking family protein 7) (Macrophin-1) (Trabeculin-alpha) (620, kDa actin-binding protein) (ABP620).]	Cytosolic
Q9UQ13	Leucine-rich repeat protein SHOC-2 (Ras-binding protein Sur-8).	Cytosolic
Q9Y217	Myotubularin-related protein 6 (EC 3.1.3.-).	Cytosolic
Q9Y275	[Tumor necrosis factor ligand superfamily member 13B (TNF- and APOL-, related leukocyte expressed ligand 1) (TALL-1) (B lymphocyte, stimulator) (BLyS) (B cell-activating factor) (BAFF) (Dendritic cell-, derived TNF-like molecule) (CD257 antigen) [Contains: Tumor necrosis, factor ligand superfamily member 13b, membrane form; Tumor necrosis, factor ligand superfamily member 13b, soluble form].]	Cytosolic
Q9Y2Q3	[Glutathione S-transferase kappa 1 (EC 2.5.1.18) (GST 13-13), (Glutathione S-transferase subunit 13) (GST class-kappa) (GSTK1-1), (hGSTK1).]	Cytosolic
Q04671	[P protein (Melanocyte-specific transporter protein) (Pink-eyed, dilution protein homolog).]	Cytosolic
O00170	[AH receptor-interacting protein (AIP) (Aryl-hydrocarbon receptor-, interacting protein) (Immunophilin homolog ARA9) (HBV X-associated, protein 2) (XAP-2).]	Cytosolic
O00244	Copper transport protein ATOX1 (Metal transport protein ATX1).	Cytosolic
O00743	Serine/threonine-protein phosphatase 6 (EC 3.1.3.16) (PP6).	Cytosolic
O14576	[Cytoplasmic dynein 1 intermediate chain 1 (Dynein intermediate chain, 1, cytosolic) (DH IC-1) (Cytoplasmic dynein intermediate chain 1).]	Cytosolic
O43776	[Asparaginyl-tRNA synthetase, cytoplasmic (EC 6.1.1.22) (Asparagine--, tRNA ligase) (AsnRS).]	Cytosolic
O60493	Sorting nexin-3 (Protein SDP3).	Cytosolic
O60547	[GDP-mannose 4,6 dehydratase (EC 4.2.1.47) (GDP-D-mannose dehydratase), (GMD).]	Cytosolic
O60880	[SH2 domain-containing protein 1A (Signaling lymphocytic activation, molecule-associated protein) (SLAM-associated protein) (T-cell signal, transduction molecule SAP) (Duncan disease SH2-protein).]	Cytosolic
O75031	Heat shock factor 2-binding protein.	Cytosolic
P07814	[Bifunctional aminoacyl-tRNA synthetase [Includes: Glutamyl-tRNA, synthetase (EC 6.1.1.17) (Glutamate--tRNA ligase); Prolyl-tRNA, synthetase (EC 6.1.1.15) (Proline--tRNA ligase)].]	Cytosolic
P08294	[Extracellular superoxide dismutase [Cu-Zn] precursor (EC 1.15.1.1), (EC-SOD).]	Cytosolic
P09758	[Tumor-associated calcium signal transducer 2 precursor (Pancreatic, carcinoma marker protein GA733-1) (Cell surface glycoprotein Trop-2).]	Cytosolic

P11413	Glucose-6-phosphate 1-dehydrogenase (EC 1.1.1.49) (G6PD).	Cytosolic
P12270	Nucleoprotein TPR.	Cytosolic
P12429	[Annexin A3 (Annexin-3) (Annexin III) (Lipocortin III) (Placental, anticoagulant protein III) (PAP-III) (35-alpha calcimedin) (Inositol, 1,2-cyclic phosphate 2-phosphohydrolase).]	Cytosolic
P14209	[CD99 antigen precursor (T-cell surface glycoprotein E2) (E2 antigen), (Protein MIC2) (12E7).]	Cytosolic
P14625	[Endoplasmin precursor (Heat shock protein 90 kDa beta member 1) (94, kDa glucose-regulated protein) (GRP94) (gp96 homolog) (Tumor rejection, antigen 1).]	Cytosolic
P15090	[Fatty acid-binding protein, adipocyte (AFABP) (Adipocyte lipid-binding, protein) (ALBP) (A-FABP).]	Cytosolic
P16333	[Cytoplasmic protein NCK1 (NCK adaptor protein 1) (SH2/SH3 adaptor, protein NCK-alpha).]	Cytosolic
P19174	[1-phosphatidylinositol-4,5-bisphosphate phosphodiesterase gamma-1, (EC 3.1.4.11) (Phosphoinositide phospholipase C) (PLC-gamma-1), (Phospholipase C-gamma-1) (PLC-II) (PLC-148).]	Cytosolic
P19838	[Nuclear factor NF-kappa-B p105 subunit (DNA-binding factor KBF1) (EBP-, 1) [Contains: Nuclear factor NF-kappa-B p50 subunit].]	Cytosolic
P41743	[Protein kinase C iota type (EC 2.7.11.13) (nPKC-iota) (Atypical, protein kinase C-lambda/iota) (aPKC-lambda/iota) (PRKC-lambda/iota).]	Cytosolic
P46108	Proto-oncogene C-crk (p38) (Adapter molecule crk).	Cytosolic
P46778	60S ribosomal protein L21.	Cytosolic
P48163	NADP-dependent malic enzyme (EC 1.1.1.40) (NADP-ME) (Malic enzyme 1).	Cytosolic
P48200	[Iron-responsive element-binding protein 2 (IRE-BP 2) (Iron regulatory, protein 2) (IRP2).]	Cytosolic
P49189	[4-trimethylaminobutyraldehyde dehydrogenase (EC 1.2.1.47) (TMABADH), (Aldehyde dehydrogenase family 9 member A1) (EC 1.2.1.3) (Aldehyde, dehydrogenase E3 isozyme) (Gamma-aminobutyraldehyde dehydrogenase), (EC 1.2.1.19) (R-aminobutyraldehyde dehydrogenase).]	Cytosolic
P49454	[Centromere protein F (Kinetochore protein CENP-F) (Mitosin) (AH, antigen).]	Cytosolic
P49588	[Alanyl-tRNA synthetase, cytoplasmic (EC 6.1.1.7) (Alanine--tRNA, ligase) (AlaRS) (Renal carcinoma antigen NY-REN-42).]	Cytosolic
P50148	[Guanine nucleotide-binding protein G(q) subunit alpha (Guanine, nucleotide-binding protein alpha-q).]	Cytosolic
P50453	[Serpin B9 (Cytoplasmic antiproteinase 3) (CAP-3) (CAP3) (Proteinase, inhibitor 9).]	Cytosolic
P50747	[Biotin--protein ligase (EC 6.3.4.-) (Biotin apo-protein ligase), [Includes: Biotin--[methylmalonyl-CoA-carboxytransferase] ligase, (EC 6.3.4.9); Biotin--[propionyl-CoA-carboxylase [ATP-hydrolyzing]], ligase (EC 6.3.4.10) (Holocarboxylase synthetase) (HCS); Biotin--, [methylcrotonoyl-CoA-carboxylase] ligase (EC 6.3.4.11); Biotin--, [acetyl-CoA-carboxylase] ligase (EC 6.3.4.15)].]	Cytosolic
P50991	[T-complex protein 1 subunit delta (TCP-1-delta) (CCT-delta), (Stimulator of TAR RNA-binding).]	Cytosolic
P51114	Fragile X mental retardation syndrome-related protein 1 (hFXR1p).	Cytosolic
P51178	[1-phosphatidylinositol-4,5-bisphosphate phosphodiesterase delta-1, (EC 3.1.4.11) (Phosphoinositide phospholipase C) (PLC-delta-1), (Phospholipase C-delta-1) (PLC-III).]	Cytosolic
P53582	[Methionine aminopeptidase 1 (EC 3.4.11.18) (MetAP 1) (MAP 1), (Peptidase M 1).]	Cytosolic
P53667	LIM domain kinase 1 (EC 2.7.11.1) (LIMK-1).	Cytosolic
P54253	Ataxin-1 (Spinocerebellar ataxia type 1 protein).	Cytosolic
P54577	[Tyrosyl-tRNA synthetase, cytoplasmic (EC 6.1.1.1) (Tyrosyl--tRNA, ligase) (TyrRS).]	Cytosolic
P55055	[Oxysterols receptor LXR-beta (Liver X receptor beta) (Nuclear orphan, receptor LXR-beta) (Ubiquitously-expressed nuclear receptor) (Nuclear, receptor NER).]	Cytosolic
Q13402	Myosin-VIIa.	Cytosolic

Q13438	Protein OS-9 precursor (Amplified in osteosarcoma 9).	Cytosolic
Q13489	[Baculoviral IAP repeat-containing protein 3 (Inhibitor of apoptosis, protein 1) (HIAP1) (HIAP-1) (C-IAP2) (TNFR2-TRAF-signaling complex, protein 1) (IAP homolog C) (Apoptosis inhibitor 2) (API2) (RING finger, protein 49).]	Cytosolic
Q13608	[Peroxisome assembly factor 2 (PAF-2) (Peroxisomal-type ATPase 1), (Peroxin-6) (Peroxisomal biogenesis factor 6).]	Cytosolic
Q14164	[Inhibitor of nuclear factor kappa-B kinase subunit epsilon, (EC 2.7.11.10) (I kappa-B kinase epsilon) (IkBKE) (IKK-epsilon) (IKK-, E) (Inducible I kappa-B kinase) (IKK-i).]	Cytosolic
Q14204	[Dynein heavy chain, cytosolic (DYHC) (Cytoplasmic dynein heavy chain, 1) (DHC1) (Dynein heavy chain 1, cytoplasmic 1).]	Cytosolic
Q14289	[Protein tyrosine kinase 2 beta (EC 2.7.10.2) (Focal adhesion kinase 2), (FADK 2) (Proline-rich tyrosine kinase 2) (Cell adhesion kinase beta), (CAK beta) (Calcium-dependent tyrosine kinase) (CADTK) (Related, adhesion focal tyrosine kinase) (RAFTK).]	Cytosolic
Q14764	Major vault protein (MVP) (Lung resistance-related protein).	Cytosolic
Q15181	[Inorganic pyrophosphatase (EC 3.6.1.1) (Pyrophosphate phospho-, hydrolase) (PPase).]	Cytosolic
Q15438	[Cytohesin-1 (PH, SEC7 and coiled-coil domain-containing protein 1), (SEC7 homolog B2-1).]	Cytosolic
Q15669	[Rho-related GTP-binding protein RhoH precursor (GTP-binding protein, TTF).]	Cytosolic
Q16539	[Mitogen-activated protein kinase 14 (EC 2.7.11.24) (Mitogen-activated, protein kinase p38 alpha) (MAP kinase p38 alpha) (Cytokine suppressive, anti-inflammatory drug-binding protein) (CSAID-binding protein) (CSBP), (MAX-interacting protein 2) (MAP kinase MXI2) (SAPK2A).]	Cytosolic
Q86W56	Poly(ADP-ribose) glycohydrolase (EC 3.2.1.143).	Cytosolic
Q8WUI4	Histone deacetylase 7a (HD7a).	Cytosolic
Q92556	Engulfment and cell motility protein 1 (CED-12 homolog).	Cytosolic
Q92598	[Heat shock protein 105 kDa (Heat shock 110 kDa protein) (Antigen NY-, CO-25).]	Cytosolic
Q92624	[Amyloid protein-binding protein 2 (Amyloid beta precursor protein-, binding protein 2) (APP-BP2) (Protein interacting with APP tail 1).]	Cytosolic
Q92876	[Kallikrein-6 precursor (EC 3.4.21.-) (Protease M) (Neurosin) (Zyme), (SP59) (Serine protease 9) (Serine protease 18).]	Cytosolic
O95163	[Elongator complex protein 1 (ELP1) (IkappaB kinase complex-associated, protein) (IKK complex-associated protein) (p150).]	Cytosolic
O95219	Sorting nexin-4.	Cytosolic
O95239	Chromosome-associated kinesin KIF4A (Chromokinesin-A).	Cytosolic
O95817	[BAG family molecular chaperone regulator 3 (Bcl-2-associated, athanogene 3) (BAG-3) (Bcl-2-binding protein Bis) (Docking protein, CAIR-1).]	Cytosolic
O95833	Chloride intracellular channel protein 3.	Cytosolic
P00326	[Alcohol dehydrogenase 1C (EC 1.1.1.1) (Alcohol dehydrogenase subunit, gamma).]	Cytosolic
P02489	[Alpha-crystallin A chain (Heat shock protein beta-4) (HspB4), [Contains: Alpha-crystallin A chain, short form].]	Cytosolic
P02649	Apolipoprotein E precursor (Apo-E).	Cytosolic
P04216	[Thy-1 membrane glycoprotein precursor (Thy-1 antigen) (CD90 antigen), (CDw90).]	Cytosolic
P04731	Metallothionein-1A (MT-1A) (Metallothionein-IA) (MT-IA).	Cytosolic
P05062	Fructose-bisphosphate aldolase B (EC 4.1.2.13) (Liver-type aldolase).	Cytosolic
P05388	60S acidic ribosomal protein P0 (L10E).	Cytosolic
P06733	[Alpha-enolase (EC 4.2.1.11) (2-phospho-D-glycerate hydro-lyase) (Non-, neural enolase) (NNE) (Enolase 1) (Phosphopyruvate hydratase) (C-myc, promoter-binding protein) (MBP-1) (MPB-1) (Plasminogen-binding, protein).]	Cytosolic
P06746	DNA polymerase beta (EC 2.7.7.7) (EC 4.2.99.-).	Cytosolic

P06850	[Corticoliberin precursor (Corticotropin-releasing factor) (CRF), (Corticotropin-releasing hormone).]	Cytosolic
P23396	40S ribosomal protein S3.	Cytosolic
P24941	Cell division protein kinase 2 (EC 2.7.11.22) (p33 protein kinase).	Cytosolic
P26640	[Valyl-tRNA synthetase (EC 6.1.1.9) (Valine--tRNA ligase) (ValRS), (Protein G7a).]	Cytosolic
P28838	[Cytosol aminopeptidase (EC 3.4.11.1) (Leucine aminopeptidase) (LAP), (Leucyl aminopeptidase) (Leucine aminopeptidase 3) (Proline, aminopeptidase) (EC 3.4.11.5) (Prolyl aminopeptidase) (Peptidase S).]	Cytosolic
P29590	[Probable transcription factor PML (Tripartite motif-containing protein, 19) (RING finger protein 71).]	Cytosolic
P30838	[Aldehyde dehydrogenase, dimeric NADP-preferring (EC 1.2.1.5) (ALDH, class 3) (ALDHIII).]	Cytosolic
P30990	[Neurotensin/neuromedin N precursor [Contains: Large neuromedin N (NmN-, 125); Neuromedin N (NmN) (NN); Neurotensin (NT); Tail peptide].]	Cytosolic
P35268	[60S ribosomal protein L22 (Epstein-Barr virus small RNA-associated, protein) (EBER-associated protein) (EAP) (Heparin-binding protein, HBp15).]	Cytosolic
P36575	[Arrestin-C (Cone arrestin) (cArr) (Retinal cone arrestin-3) (X-, arrestin) (C-arrestin).]	Cytosolic
P40126	[L-dopachrome tautomerase precursor (EC 5.3.3.12) (DT) (DCT) (L-, dopachrome Delta-isomerase) (Tyrosinase-related protein 2) (TRP-2), (TRP2).]	Cytosolic
P40227	[T-complex protein 1 subunit zeta (TCP-1-zeta) (CCT-zeta) (CCT-zeta-1), (Tcp20) (HTR3) (Acute morphine dependence-related protein 2).]	Cytosolic
P41250	Glycyl-tRNA synthetase (EC 6.1.1.14) (Glycine--tRNA ligase) (GlyRS).	Cytosolic
P61163	[Alpha-centractin (Centractin) (Centrosome-associated actin homolog), (Actin-RPV) (ARP1).]	Cytosolic
P61224	Ras-related protein Rap-1b precursor (GTP-binding protein smg p21B).	Cytosolic
P62241	40S ribosomal protein S8.	Cytosolic
P62263	40S ribosomal protein S14.	Cytosolic
P62861	40S ribosomal protein S30.	Cytosolic
P62913	60S ribosomal protein L11 (CLL-associated antigen KW-12).	Cytosolic
Q9BXW6	[Oxysterol-binding protein-related protein 1 (OSBP-related protein 1), (ORP-1).]	Cytosolic
P84098	60S ribosomal protein L19.	Cytosolic
P98170	[Baculoviral IAP repeat-containing protein 4 (Inhibitor of apoptosis, protein 3) (X-linked inhibitor of apoptosis protein) (X-linked IAP), (IAP-like protein) (HILP).]	Cytosolic
P99999	Cytochrome c.	Cytosolic
Q00597	Fanconi anemia group C protein (Protein FACC).	Cytosolic
Q02543	60S ribosomal protein L18a.	Cytosolic
Q02790	[FK506-binding protein 4 (EC 5.2.1.8) (Peptidyl-prolyl cis-trans, isomerase) (PPIase) (Rotamase) (p59 protein) (HSP-binding, immunophilin) (HBI) (FKBP52 protein) (52 kDa FK506-binding protein), (FKBP59).]	Cytosolic
Q02880	[DNA topoisomerase 2-beta (EC 5.99.1.3) (DNA topoisomerase II, beta, isozyme).]	Cytosolic
Q04206	Transcription factor p65 (Nuclear factor NF-kappa-B p65 subunit).	Cytosolic
Q06187	[Tyrosine-protein kinase BTK (EC 2.7.10.2) (Bruton tyrosine kinase), (Agammaglobulinaemia tyrosine kinase) (ATK) (B-cell progenitor kinase), (BPK).]	Cytosolic

Q08188	[Protein-glutamine gamma-glutamyltransferase E precursor (EC 2.3.2.13), (TGase E) (TGE) (TG(E)) (Transglutaminase-3) [Contains: Protein-, glutamine gamma-glutamyltransferase E 50 kDa non-catalytic chain;, Protein-glutamine gamma-glutamyltransferase E 27 kDa catalytic chain].]	Cytosolic
Q12815	Trophinin-associated protein (Tastin) (Trophinin-assisting protein).	Cytosolic
Q12882	[Dihydropyrimidine dehydrogenase [NADP+] precursor (EC 1.3.1.2) (DPD), (DHPDHase) (Dihydrouracil dehydrogenase) (Dihydrothymine, dehydrogenase).]	Cytosolic
Q93038	[Tumor necrosis factor receptor superfamily member 25 precursor (WSL-1, protein) (Apoptosis-mediating receptor DR3) (Apoptosis-mediating, receptor TRAMP) (Death domain receptor 3) (WSL protein) (Apoptosis-, inducing receptor AIR) (Apo-3) (Lymphocyte-associated receptor of, death) (LARD).]	Cytosolic
Q99593	T-box transcription factor TBX5 (T-box protein 5).	Cytosolic
Q99832	[T-complex protein 1 subunit eta (TCP-1-eta) (CCT-eta) (HIV-1 Nef-, interacting protein).]	Cytosolic
Q9H000	Makorin-2 (RING finger protein 62).	Cytosolic
Q9NP31	[SH2 domain-containing protein 2A (T cell-specific adapter protein), (TSAd) (VEGF receptor-associated protein) (SH2 domain-containing, adapter protein).]	Cytosolic
Q9NR64	Kelch-like protein 1.	Cytosolic
Q9NRS6	Sorting nexin-15.	Cytosolic
Q9NX02	[NACHT, LRR and PYD domains-containing protein 2 (PYRIN domain and, NACHT domain-containing protein 1) (PYRIN-containing APAF1-like, protein 2) (Nucleotide-binding site protein 1).]	Cytosolic
Q9UGI0	Zinc finger Ran-binding domain-containing protein 1 (Protein TRABID).	Cytosolic
Q9UHD4	[Cell death activator CIDE-B (Cell death-inducing DFFA-like effector, B).]	Cytosolic
Q9UIA9	Exportin-7 (Exp7) (Ran-binding protein 16).	Cytosolic
Q9UJW3	DNA (cytosine-5)-methyltransferase 3-like.	Cytosolic
Q9UK41	Vacuolar protein sorting-associated protein 28 homolog (H-Vps28).	Cytosolic
Q9UKV0	[Histone deacetylase 9 (HD9) (HD7B) (HD7) (Histone deacetylase-related, protein) (MEF2-interacting transcription repressor MITR).]	Cytosolic
Q9UMR2	[ATP-dependent RNA helicase DDX19B (EC 3.6.1.-) (DEAD box protein 19B), (DEAD box RNA helicase DEAD5).]	Cytosolic
Q9UQL6	Histone deacetylase 5 (HD5) (Antigen NY-CO-9).	Cytosolic
Q9Y2G2	[Caspase recruitment domain-containing protein 8 (Apoptotic protein, NDPP1) (DACAR) (CARD-inhibitor of NF-kappa-B-activating ligand), (CARDINAL) (Tumor up-regulated CARD-containing antagonist of CASP9), (TUCAN).]	Cytosolic
O00154	[Cytosolic acyl coenzyme A thioester hydrolase (EC 3.1.2.2) (Long chain, acyl-CoA thioester hydrolase) (CTE-II) (CTE-IIa) (Brain acyl-CoA, hydrolase) (Acyl-CoA thioesterase 7).]	Cytosolic
O00192	Armadillo repeat protein deleted in velo-cardio-facial syndrome.	Cytosolic
O00311	[Cell division cycle 7-related protein kinase (EC 2.7.11.1) (CDC7-, related kinase) (HsCdc7) (huCdc7).]	Cytosolic
O00418	[Elongation factor 2 kinase (EC 2.7.11.20) (eEF-2 kinase) (eEF-2K), (Calcium/calmodulin-dependent eukaryotic elongation factor 2 kinase).]	Cytosolic
O14641	[Segment polarity protein dishevelled homolog DVL-2 (Dishevelled-2), (DSH homolog 2).]	Cytosolic
O14893	[Survival of motor neuron protein-interacting protein 1 (SMN-, interacting protein 1) (Component of gems 2) (Gemin-2).]	Cytosolic
O15294	[UDP-N-acetylglucosamine--peptide N-acetylglucosaminyltransferase 110, kDa subunit (EC 2.4.1.-) (O-GlcNAc transferase subunit p110) (O-linked, N-acetylglucosamine transferase 110 kDa subunit).]	Cytosolic
O43281	Embryonal Fyn-associated substrate (HEFS).	Cytosolic

O43927	[Small-inducible cytokine B13 precursor (CXCL13) (B lymphocyte, chemoattractant) (CXC chemokine BLC) (B cell-attracting chemokine 1), (BCA-1) (ANGIE).]	Cytosolic
O60760	[Glutathione-requiring prostaglandin D synthase (EC 5.3.99.2), (Glutathione-dependent PGD synthetase) (Prostaglandin-H2 D-isomerase), (Hematopoietic prostaglandin D synthase) (H-PGDS).]	Cytosolic
O75791	[GRB2-related adapter protein 2 (GADS protein) (Growth factor receptor-, binding protein) (GRBLG) (Grf40 adapter protein) (Grf-40) (GRB-2-like, protein) (GRB2L) (GRBX) (P38) (Hematopoietic cell-associated adapter, protein GrpL) (Adapter protein GRID) (SH3-SH2-SH3 adapter Mona).]	Cytosolic
O75912	[Diacylglycerol kinase iota (EC 2.7.1.107) (Diglyceride kinase iota), (DGK-iota) (DAG kinase iota).]	Cytosolic
P07948	Tyrosine-protein kinase Lyn (EC 2.7.10.2).	Cytosolic
P08151	Zinc finger protein GLI1 (Glioma-associated oncogene) (Oncogene GLI).	Cytosolic
P08758	[Annexin A5 (Annexin-5) (Annexin V) (Lipocortin V) (Endonexin II), (Calphobindin I) (CBP-I) (Placental anticoagulant protein I) (PAP-I), (PP4) (Thromboplastin inhibitor) (Vascular anticoagulant-alpha) (VAC-, alpha) (Anchorin CII).]	Cytosolic
P08949	Neuromedin-B precursor [Contains: Neuromedin-B-32; Neuromedin-B].	Cytosolic
P09525	[Annexin A4 (Annexin-4) (Annexin IV) (Lipocortin IV) (Endonexin I), (Chromobindin-4) (Protein II) (P32.5) (Placental anticoagulant protein, II) (PAP-II) (PP4-X) (35-beta calcimedin) (Carbohydrate-binding, protein P33/P41) (P33/41).]	Cytosolic
P11940	Polyadenylate-binding protein 1 (Poly(A)-binding protein 1) (PABP 1).	Cytosolic
P12271	[Retinaldehyde-binding protein 1 (Cellular retinaldehyde-binding, protein).]	Cytosolic
P13796	[Plastin-2 (L-plastin) (Lymphocyte cytosolic protein 1) (LCP-1), (LC64P).]	Cytosolic
P13861	cAMP-dependent protein kinase type II-alpha regulatory subunit.	Cytosolic
P14618	[Pyruvate kinase isozymes M1/M2 (EC 2.7.1.40) (Pyruvate kinase muscle, isozyme) (Pyruvate kinase 2/3) (Cytosolic thyroid hormone-binding, protein) (CTHBP) (THBP1).]	Cytosolic
P14923	[Junction plakoglobin (Desmoplakin-3) (Desmoplakin III) (Catenin, gamma).]	Cytosolic
P15170	[G1 to S phase transition protein 1 homolog (GTP-binding protein GST1-, HS).]	Cytosolic
P15559	[NAD(P)H dehydrogenase [quinone] 1 (EC 1.6.5.2) (Quinone reductase 1), (NAD(P)H:quinone oxidoreductase 1) (QR1) (DT-diaphorase) (DTD), (Azoreductase) (Phylloquinone reductase) (Menadione reductase).]	Cytosolic
P18124	60S ribosomal protein L7.	Cytosolic
P18509	[Pituitary adenylate cyclase-activating polypeptide precursor (PACAP), [Contains: PACAP-related peptide (PRP-48); Pituitary adenylate, cyclase-activating polypeptide 27 (PACAP-27) (PACAP27); Pituitary, adenylate cyclase-activating polypeptide 38 (PACAP-38) (PACAP38)].]	Cytosolic
P20591	[Interferon-induced GTP-binding protein Mx1 (Interferon-regulated, resistance GTP-binding protein MxA) (Interferon-induced protein p78), (IFI-78K).]	Cytosolic
P20936	[Ras GTPase-activating protein 1 (GTPase-activating protein) (GAP) (Ras, p21 protein activator) (p120GAP) (RasGAP).]	Cytosolic
P21399	[Iron-responsive element-binding protein 1 (IRE-BP 1) (Iron regulatory, protein 1) (IRP1) (Ferritin repressor protein) (Aconitate hydratase), (EC 4.2.1.3) (Citrate hydro-lyase) (Aconitase).]	Cytosolic
P43403	[Tyrosine-protein kinase ZAP-70 (EC 2.7.10.2) (70 kDa zeta-associated, protein) (Syk-related tyrosine kinase).]	Cytosolic
P46060	Ran GTPase-activating protein 1.	Cytosolic
P46782	40S ribosomal protein S5.	Cytosolic
P49589	[Cysteinyl-tRNA synthetase, cytoplasmic (EC 6.1.1.16) (Cysteine--tRNA, ligase) (CysRS).]	Cytosolic
P49815	Tuberin (Tuberous sclerosis 2 protein).	Cytosolic

P49914	[5-formyltetrahydrofolate cyclo-ligase (EC 6.3.3.2) (5,10-methenyl-, tetrahydrofolate synthetase) (Methenyl-THF synthetase) (MTHFS).]	Cytosolic
P50502	[Hsc70-interacting protein (Hip) (Suppression of tumorigenicity protein, 13) (Putative tumor suppressor ST13) (Protein FAM10A1) (Progesterone, receptor-associated p48 protein) (Renal carcinoma antigen NY-REN-33).]	Cytosolic
P50570	Dynamin-2 (EC 3.6.5.5).	Cytosolic
P50995	[Annexin A11 (Annexin-11) (Annexin XI) (Calcyclin-associated annexin, 50) (CAP-50) (56 kDa autoantigen).]	Cytosolic
P51570	Galactokinase (EC 2.7.1.6) (Galactose kinase).	Cytosolic
P51606	[N-acylglucosamine 2-epimerase (EC 5.1.3.8) (GlcNAc 2-epimerase) (N-, acetyl-D-glucosamine 2-epimerase) (AGE) (Renin-binding protein), (RnBP).]	Cytosolic
P51854	[Transketolase-like protein 1 (EC 2.2.1.1) (Transketolase 2) (TK 2), (Transketolase-related protein).]	Cytosolic
P53367	Arfaptin-1 (ADP-ribosylation factor-interacting protein 1).	Cytosolic
P53671	LIM domain kinase 2 (EC 2.7.11.1) (LIMK-2).	Cytosolic
P53778	[Mitogen-activated protein kinase 12 (EC 2.7.11.24) (Extracellular, signal-regulated kinase 6) (ERK-6) (ERK5) (Stress-activated protein, kinase 3) (Mitogen-activated protein kinase p38 gamma) (MAP kinase p38, gamma).]	Cytosolic
P55072	[Transitional endoplasmic reticulum ATPase (TER ATPase) (15S Mg(2+)-, ATPase p97 subunit) (Valosin-containing protein) (VCP).]	Cytosolic
P56524	Histone deacetylase 4 (HD4).	Cytosolic
P57075	[Suppressor of T-cell receptor signaling 2 (STS-2) (Cbl-interacting, protein 4) (CLIP4) (T-cell ubiquitin ligand) (TULA).]	Cytosolic
Q13418	[Integrin-linked protein kinase (EC 2.7.11.1) (ILK-1) (ILK-2) (59 kDa, serine/threonine-protein kinase) (p59ILK).]	Cytosolic
Q13547	Histone deacetylase 1 (HD1).	Cytosolic
Q13614	Myotubularin-related protein 2 (EC 3.1.3.-).	Cytosolic
Q14142	Tripartite motif-containing protein 14.	Cytosolic
Q14974	[Importin subunit beta-1 (Karyopherin subunit beta-1) (Nuclear factor, P97) (Importin 90).]	Cytosolic
Q15019	Septin-2 (Protein NEDD5).	Cytosolic
Q15046	Lysyl-tRNA synthetase (EC 6.1.1.6) (Lysine--tRNA ligase) (LysRS).	Cytosolic
Q15139	[Serine/threonine-protein kinase D1 (EC 2.7.11.13) (nPKC-D1) (Protein, kinase D) (Protein kinase C mu type) (nPKC-mu).]	Cytosolic
Q16760	[Diacylglycerol kinase delta (EC 2.7.1.107) (Diglyceride kinase delta), (DGK-delta) (DAG kinase delta) (130 kDa diacylglycerol kinase).]	Cytosolic
O76083	[High affinity cGMP-specific 3',5'-cyclic phosphodiesterase 9A, (EC 3.1.4.35).]	Cytosolic
O95164	[Ubiquitin-like protein 3 precursor (Membrane-anchored ubiquitin-fold, protein) (MUB) (HsMUB) (Protein HCG-1).]	Cytosolic
O95433	Activator of 90 kDa heat shock protein ATPase homolog 1 (AHA1) (p38).	Cytosolic
O95644	[Nuclear factor of activated T-cells, cytoplasmic 1 (NFAT transcription, complex cytosolic component) (NF-ATc1) (NF-ATc).]	Cytosolic
O95954	[Formimidoyltransferase-cyclodeaminase (Formiminotransferase-, cyclodeaminase) (FTCD) (LCHC1) [Includes: Glutamate, formimidoyltransferase (EC 2.1.2.5) (Glutamate formiminotransferase), (Glutamate formyltransferase); Formimidoyltetrahydrofolate, cyclodeaminase (EC 4.3.1.4) (Formiminotetrahydrofolate, cyclodeaminase)].]	Cytosolic
P25963	[NF-kappa-B inhibitor alpha (Major histocompatibility complex enhancer-, binding protein MAD3) (I-kappa-B-alpha) (IkappaBalpha) (IkB-alpha).]	Cytosolic
P00338	[L-lactate dehydrogenase A chain (EC 1.1.1.27) (LDH-A) (LDH muscle, subunit) (LDH-M) (Renal carcinoma antigen NY-REN-59) (Cell, proliferation-inducing gene 19 protein).]	Cytosolic

P01189	[Corticotropin-lipotropin precursor (Pro-opiomelanocortin) (POMC), [Contains: NPP; Melanotropin gamma (Gamma-MSH); Potential peptide;, Corticotropin (Adrenocorticotropic hormone) (ACTH); Melanotropin alpha, (Alpha-MSH); Corticotropin-like intermediary peptide (CLIP);, Lipotropin beta (Beta-LPH); Lipotropin gamma (Gamma-LPH); Melanotropin, beta (Beta-MSH); Beta-endorphin; Met-enkephalin].]	Cytosolic
P01215	[Glycoprotein hormones alpha chain precursor (Anterior pituitary, glycoprotein hormones common subunit alpha) (Follitropin alpha chain), (Follicle-stimulating hormone alpha chain) (FSH-alpha) (Lutropin alpha, chain) (Luteinizing hormone alpha chain) (LSH-alpha) (Thyrotropin, alpha chain) (Thyroid-stimulating hormone alpha chain) (TSH-alpha), (Choriogonadotropin alpha chain) (Chorionic gonadotrophin alpha, subunit) (CG-alpha).]	Cytosolic
P01375	[Tumor necrosis factor precursor (TNF-alpha) (Tumor necrosis factor, ligand superfamily member 2) (TNF-a) (Cachectin) [Contains: Tumor, necrosis factor, membrane form; Tumor necrosis factor, soluble form].]	Cytosolic
P02511	[Alpha-crystallin B chain (Alpha(B)-crystallin) (Rosenthal fiber, component) (Heat shock protein beta-5) (HspB5) (Renal carcinoma, antigen NY-REN-27).]	Cytosolic
P05089	Arginase-1 (EC 3.5.3.1) (Type I arginase) (Liver-type arginase).	Cytosolic
P07195	[L-lactate dehydrogenase B chain (EC 1.1.1.27) (LDH-B) (LDH heart, subunit) (LDH-H) (Renal carcinoma antigen NY-REN-46).]	Cytosolic
P07205	[Phosphoglycerate kinase 2 (EC 2.7.2.3) (Phosphoglycerate kinase,, testis specific).]	Cytosolic
P21673	[Diamine acetyltransferase 1 (EC 2.3.1.57) (Spermidine/spermine N(1)-, acetyltransferase 1) (SSAT) (SSAT-1) (Putrescine acetyltransferase), (Polyamine N-acetyltransferase 1).]	Cytosolic
P23526	[Adenosylhomocysteinase (EC 3.3.1.1) (S-adenosyl-L-homocysteine, hydrolase) (AdoHcyase).]	Cytosolic
P26373	60S ribosomal protein L13 (Breast basic conserved protein 1).	Cytosolic
P26651	[Tristetraproline (TTP) (Zinc finger protein 36 homolog) (Zfp-36), (Protein TIS11A) (TIS11) (Growth factor-inducible nuclear protein, NUP475) (G0/G1 switch regulatory protein 24).]	Cytosolic
P27037	[Activin receptor type-2A precursor (EC 2.7.11.30) (Activin receptor, type IIA) (ACTR-IIA) (ACTRIIA).]	Cytosolic
P27797	Calreticulin precursor (CRP55) (Calregulin) (HACBP) (ERp60) (grp60).	Cytosolic
P29992	[Guanine nucleotide-binding protein subunit alpha-11 (G alpha-11), (Guanine nucleotide-binding protein G(y) subunit alpha).]	Cytosolic
P30305	[M-phase inducer phosphatase 2 (EC 3.1.3.48) (Dual specificity, phosphatase Cdc25B).]	Cytosolic
P31749	[RAC-alpha serine/threonine-protein kinase (EC 2.7.11.1) (RAC-PK-alpha), (Protein kinase B) (PKB) (C-AKT).]	Cytosolic
P31949	[Protein S100-A11 (S100 calcium-binding protein A11) (Protein S100C), (Calgizzarin) (MLN 70).]	Cytosolic
P32119	[Peroxiredoxin-2 (EC 1.11.1.15) (Thioredoxin peroxidase 1), (Thioredoxin-dependent peroxide reductase 1) (Thiol-specific, antioxidant protein) (TSA) (PRP) (Natural killer cell-enhancing factor, B) (NKEF-B).]	Cytosolic
P35236	[Tyrosine-protein phosphatase non-receptor type 7 (EC 3.1.3.48), (Protein-tyrosine phosphatase LC-PTP) (Hematopoietic protein-tyrosine, phosphatase) (HEPTP).]	Cytosolic
P35568	Insulin receptor substrate 1 (IRS-1).	Cytosolic
P35998	[26S protease regulatory subunit 7 (Proteasome 26S subunit ATPase 2), (Protein MSS1).]	Cytosolic
P39019	40S ribosomal protein S19.	Cytosolic
P40763	[Signal transducer and activator of transcription 3 (Acute-phase, response factor).]	Cytosolic
P41252	[Isoleucyl-tRNA synthetase, cytoplasmic (EC 6.1.1.5) (Isoleucine--tRNA, ligase) (IleRS) (IRS).]	Cytosolic
P60484	[Phosphatidylinositol-3,4,5-trisphosphate 3-phosphatase and dual-, specificity protein phosphatase PTEN (EC 3.1.3.67) (EC 3.1.3.16), (EC 3.1.3.48) (Phosphatase and tensin homolog) (Mutated in multiple, advanced cancers 1).]	Cytosolic
P61201	[COP9 signalosome complex subunit 2 (Signalosome subunit 2) (SGN2), (JAB1-containing signalosome subunit 2) (Thyroid receptor-interacting, protein 15) (TRIP-15) (Alien homolog).]	Cytosolic

P61927	60S ribosomal protein L37 (G1.16).	Cytosolic
P62249	40S ribosomal protein S16.	Cytosolic
P62266	40S ribosomal protein S23.	Cytosolic
P62424	[60S ribosomal protein L7a (Surfeit locus protein 3) (PLA-X, polypeptide).]	Cytosolic
P62753	40S ribosomal protein S6 (Phosphoprotein NP33).	Cytosolic
P62826	[GTP-binding nuclear protein Ran (GTPase Ran) (Ras-like protein TC4), (Androgen receptor-associated protein 24).]	Cytosolic
P62841	40S ribosomal protein S15 (RIG protein).	Cytosolic
P62917	60S ribosomal protein L8.	Cytosolic
P63104	[14-3-3 protein zeta/delta (Protein kinase C inhibitor protein 1), (KCIP-1).]	Cytosolic
P68402	[Platelet-activating factor acetylhydrolase IB subunit beta, (EC 3.1.1.47) (PAF acetylhydrolase 30 kDa subunit) (PAF-AH 30 kDa, subunit) (PAF-AH subunit beta) (PAFAH subunit beta).]	Cytosolic
Q9C000	[NACHT, LRR and PYD domains-containing protein 1 (Death effector, filament-forming ced-4-like apoptosis protein) (Nucleotide-binding, domain and caspase recruitment domain) (Caspase recruitment domain-, containing protein 7).]	Cytosolic
P98171	[Rho GTPase-activating protein 4 (Rho-GAP hematopoietic protein C1), (p115).]	Cytosolic
Q01814	[Plasma membrane calcium-transporting ATPase 2 (EC 3.6.3.8) (PMCA2), (Plasma membrane calcium pump isoform 2) (Plasma membrane calcium, ATPase isoform 2).]	Cytosolic
Q04759	Protein kinase C theta type (EC 2.7.11.13) (nPKC-theta).	Cytosolic
Q05513	Protein kinase C zeta type (EC 2.7.11.13) (nPKC-zeta).	Cytosolic
Q05655	Protein kinase C delta type (EC 2.7.11.13) (nPKC-delta).	Cytosolic
Q07020	60S ribosomal protein L18.	Cytosolic
Q07343	[cAMP-specific 3',5'-cyclic phosphodiesterase 4B (EC 3.1.4.17) (DPDE4), (PDE32).]	Cytosolic
Q08211	[ATP-dependent RNA helicase A (EC 3.6.1.-) (Nuclear DNA helicase II), (NDH II) (DEAH box protein 9).]	Cytosolic
Q12888	[Tumor suppressor p53-binding protein 1 (p53-binding protein 1), (p53BP1) (53BP1).]	Cytosolic
Q13202	[Dual specificity protein phosphatase 8 (EC 3.1.3.48) (EC 3.1.3.16), (Dual specificity protein phosphatase hVH-5).]	Cytosolic
Q13287	N-myc-interactor (Nmi) (N-myc and STAT interactor).	Cytosolic
Q13322	[Growth factor receptor-bound protein 10 (GRB10 adaptor protein), (Insulin receptor-binding protein GRB-IR).]	Cytosolic
Q92985	Interferon regulatory factor 7 (IRF-7).	Cytosolic
Q92990	[Glomulin (FKBP-associated protein) (FK506-binding protein-associated, protein) (FAP).]	Cytosolic
Q9BQI3	[Eukaryotic translation initiation factor 2-alpha kinase 1, (EC 2.7.11.1) (Heme-regulated eukaryotic initiation factor eIF-2-alpha, kinase) (Hemin-sensitive initiation factor 2-alpha kinase) (Heme-, regulated inhibitor) (Heme-controlled repressor) (HCR).]	Cytosolic
Q9BT40	[Skeletal muscle and kidney-enriched inositol phosphatase, (EC 3.1.3.56).]	Cytosolic
Q9H0B6	Kinesin light chain 2 (KLC 2).	Cytosolic
Q9NQT4	[Exosome complex exonuclease RRP46 (EC 3.1.13.-) (Ribosomal RNA-, processing protein 46) (Exosome component 5) (p12B) (Chronic, myelogenous leukemia tumor antigen 28).]	Cytosolic
Q9NR56	Muscleblind-like protein 1 (Triplet-expansion RNA-binding protein).	Cytosolic
Q9NZN9	Aryl-hydrocarbon-interacting protein-like 1.	Cytosolic
Q9ULA0	Aspartyl aminopeptidase (EC 3.4.11.21).	Cytosolic
Q9UNH7	Sorting nexin-6 (TRAF4-associated factor 2).	Cytosolic

Q9Y316	[Protein MEMO1 (Mediator of ErbB2-driven cell motility 1) (Protein, memo) (C21orf19-like protein) (Hepatitis C virus NS5A-transactivated, protein 7) (HCV NS5A-transactivated protein 7).]	Cytosolic
O60568	[Procollagen-lysine,2-oxoglutarate 5-dioxygenase 3 precursor, (EC 1.14.11.4) (Lysyl hydroxylase 3) (LH3).]	ER
P10632	[Cytochrome P450 2C8 (EC 1.14.14.1) (CYPIIC8) (P450 form 1) (P450 MP-, 12/MP-20) (P450 IIC2) (S-mephenytoin 4-hydroxylase).]	ER
P13674	[Prolyl 4-hydroxylase subunit alpha-1 precursor (EC 1.14.11.2) (4-PH, alpha-1) (Procollagen-proline,2-oxoglutarate-4-dioxygenase alpha-1, subunit).]	ER
P16435	NADPH--cytochrome P450 reductase (EC 1.6.2.4) (CPR) (P450R).	ER
P18850	[Cyclic AMP-dependent transcription factor ATF-6 alpha (Activating, transcription factor 6 alpha) (ATF6-alpha) [Contains: Processed cyclic, AMP-dependent transcription factor ATF-6 alpha].]	ER
P49257	[Protein ERGIC-53 precursor (ER-Golgi intermediate compartment 53 kDa, protein) (Lectin mannose-binding 1) (Gp58) (Intracellular mannose-, specific lectin MR60).]	ER
Q13724	[Mannosyl-oligosaccharide glucosidase (EC 3.2.1.106) (Processing A-, glucosidase I).]	ER
Q14703	[Membrane-bound transcription factor site-1 protease precursor, (EC 3.4.21.112) (S1P endopeptidase) (Site-1 protease), (Subtilisin/kexin-isozyme 1) (SKI-1).]	ER
O95169	[NADH dehydrogenase [ubiquinone] 1 beta subcomplex subunit 8,, mitochondrial precursor (EC 1.6.5.3) (EC 1.6.99.3) (NADH-ubiquinone, oxidoreductase ASHI subunit) (Complex I-ASHI) (CI-ASHI).]	ER
P02654	Apolipoprotein C-I precursor (Apo-CI) (ApoC-I).	ER
P04156	[Major prion protein precursor (PrP) (PrP27-30) (PrP33-35C) (ASCR), (CD230 antigen).]	ER
P30040	Endoplasmic reticulum protein ERp29 precursor (ERp31) (ERp28).	ER
P30101	[Protein disulfide-isomerase A3 precursor (EC 5.3.4.1) (Disulfide, isomerase ER-60) (ERp60) (58 kDa microsomal protein) (p58) (ERp57) (58, kDa glucose-regulated protein).]	ER
P30989	[Neurotensin receptor type 1 (NT-R-1) (High-affinity levocabastine-, insensitive neurotensin receptor) (NTRH).]	ER
P37108	[Signal recognition particle 14 kDa protein (SRP14) (18 kDa Alu RNA-, binding protein).]	ER
P37287	[Phosphatidylinositol N-acetylglucosaminyltransferase subunit A, (EC 2.4.1.198) (GlcNAc-PI synthesis protein) (Phosphatidylinositol-, glycan biosynthesis class A protein) (PIG-A).]	ER
P38484	[Interferon-gamma receptor beta chain precursor (Interferon-gamma, receptor accessory factor 1) (AF-1) (Interferon-gamma transducer 1).]	ER
Q93063	[Exostosin-2 (EC 2.4.1.224) (EC 2.4.1.225) (Glucuronosyl-N-, acetylglucosaminyl-proteoglycan/N-acetylglucosaminyl-proteoglycan 4-, alpha-N-acetylglucosaminyltransferase) (Putative tumor suppressor, protein EXT2) (Multiple exostoses protein 2).]	ER
Q99828	[Calcium and integrin-binding protein 1 (Calmyrin) (DNA-PKcs-, interacting protein) (Kinase-interacting protein) (KIP) (CIB) (SNK-, interacting protein 2-28) (SIP2-28).]	ER
Q9UBR2	Cathepsin Z precursor (EC 3.4.22.-) (Cathepsin X) (Cathepsin P).	ER
O00469	[Procollagen-lysine,2-oxoglutarate 5-dioxygenase 2 precursor, (EC 1.14.11.4) (Lysyl hydroxylase 2) (LH2).]	ER
O14967	Calmegin precursor.	ER
O15533	[Tapasin precursor (TPSN) (TPN) (TAP-binding protein) (TAP-associated, protein) (NGS-17).]	ER
O43264	Centromere/kinetochore protein zw10 homolog.	ER
O43896	Kinesin-like protein KIF1C.	ER
O60704	[Protein-tyrosine sulfotransferase 2 (EC 2.8.2.20) (Tyrosylprotein, sulfotransferase-2) (TPST-2).]	ER
P08842	[Steryl-sulfatase precursor (EC 3.1.6.2) (Steroid sulfatase) (Steryl-, sulfate sulfohydrolase) (Arylsulfatase C) (ASC).]	ER
P13667	[Protein disulfide-isomerase A4 precursor (EC 5.3.4.1) (Protein ERp-72), (ERp72).]	ER

P16615	[Sarcoplasmic/endoplasmic reticulum calcium ATPase 2 (EC 3.6.3.8), (Calcium pump 2) (SERCA2) (SR Ca(2+)-ATPase 2) (Calcium-transporting, ATPase sarcoplasmic reticulum type, slow twitch skeletal muscle, isoform) (Endoplasmic reticulum class 1/2 Ca(2+) ATPase).]	ER
P19086	[Guanine nucleotide-binding protein G(z) subunit alpha (G(x) alpha, chain) (Gz-alpha).]	ER
P49810	[Presenilin-2 (EC 3.4.23.-) (PS-2) (STM-2) (E5-1) (AD3LP) (AD5), [Contains: Presenilin-2 NTF subunit; Presenilin-2 CTF subunit].]	ER
P55210	[Caspase-7 precursor (EC 3.4.22.60) (CASP-7) (ICE-like apoptotic, protease 3) (ICE-LAP3) (Apoptotic protease Mch-3) (CMH-1) [Contains:, Caspase-7 subunit p20; Caspase-7 subunit p11].]	ER
P55327	Tumor protein D52 (Protein N8).	ER
Q15006	Tetratricopeptide repeat protein 35 (TPR repeat protein 35).	ER
Q15084	[Protein disulfide-isomerase A6 precursor (EC 5.3.4.1) (Protein, disulfide isomerase P5) (Thioredoxin domain-containing protein 7).]	ER
Q15293	Reticulocalbin-1 precursor.	ER
Q8TAT6	Nuclear protein localization protein 4 homolog (Protein NPL4).	ER
P04035	[3-hydroxy-3-methylglutaryl-coenzyme A reductase (EC 1.1.1.34) (HMG-CoA, reductase).]	ER
P04114	[Apolipoprotein B-100 precursor (Apo B-100) [Contains: Apolipoprotein, B-48 (Apo B-48)].]	ER
P05093	[Cytochrome P450 17A1 (EC 1.14.99.9) (CYPXVII) (P450-C17) (P450c17), (Steroid 17-alpha-monooxygenase) (Steroid 17-alpha-hydroxylase/17,20, lyase).]	ER
P22760	Arylacetamide deacetylase (EC 3.1.1.-) (AADAC).	ER
P23284	[Peptidyl-prolyl cis-trans isomerase B precursor (EC 5.2.1.8) (PPIase), (Rotamase) (Cyclophilin B) (S-cyclophilin) (SCYLP) (CYP-S1).]	ER
P27824	[Calnexin precursor (Major histocompatibility complex class I antigen-, binding protein p88) (p90) (IP90).]	ER
Q12770	[Sterol regulatory element-binding protein cleavage-activating protein, (SREBP cleavage-activating protein) (SCAP).]	ER
Q12797	[Aspartyl/asparaginyl beta-hydroxylase (EC 1.14.11.16) (Aspartate beta-, hydroxylase) (ASP beta-hydroxylase) (Peptide-aspartate beta-, dioxygenase).]	ER
Q99541	Adipophilin (Adipose differentiation-related protein) (ADRP).	ER
Q9NQC3	[Reticulon-4 (Neurite outgrowth inhibitor) (Nogo protein) (Foocen), (Neuroendocrine-specific protein) (NSP) (Neuroendocrine-specific, protein C homolog) (RTN-x) (Reticulon-5).]	ER
Q9NRZ7	[1-acyl-sn-glycerol-3-phosphate acyltransferase gamma (EC 2.3.1.51) (1-, AGP acyltransferase 3) (1-AGPAT 3) (Lysophosphatidic acid, acyltransferase gamma) (LPAAT-gamma) (1-acylglycerol-3-phosphate O-, acyltransferase 3).]	ER
Q9P2W9	Syntaxin-18 (Cell growth-inhibiting gene 9 protein).	ER
Q9UNK0	Syntaxin-8.	ER
O15118	Niemann-Pick C1 protein precursor.	ER
P08107	Heat shock 70 kDa protein 1 (HSP70.1) (HSP70-1/HSP70-2).	ER
P11511	[Cytochrome P450 19A1 (EC 1.14.14.1) (Aromatase) (CYPXIX) (Estrogen, synthetase) (P-450AROM).]	ER
P11712	[Cytochrome P450 2C9 (EC 1.14.13.80) ((R)-limonene 6-monooxygenase), (EC 1.14.13.48) ((S)-limonene 6-monooxygenase) (EC 1.14.13.49) ((S)-, limonene 7-monooxygenase) (CYPIIC9) (P450 PB-1) (P450 MP-4/MP-8) (S-, mephenytoin 4-hydroxylase) (P-450MP).]	ER
P13637	[Sodium/potassium-transporting ATPase subunit alpha-3 (EC 3.6.3.9), (Sodium pump subunit alpha-3) (Na(+)/K(+) ATPase alpha-3 subunit), (Na(+)/K(+) ATPase alpha(III) subunit).]	ER
P20774	Mimecan precursor (Osteoglycin) (Osteoinductive factor) (OIF).	ER

P20813	Cytochrome P450 2B6 (EC 1.14.14.1) (CYPIIB6) (P450 IIB1).	ER
P21246	[Pleiotrophin precursor (PTN) (Heparin-binding growth-associated, molecule) (HB-GAM) (Heparin-binding growth factor 8) (HBGF-8), (Osteoblast-specific factor 1) (OSF-1) (Heparin-binding neurite, outgrowth-promoting factor 1) (HBNF-1) (Heparin-binding brain mitogen), (HBBM).]	ER
P43155	[Carnitine O-acetyltransferase (EC 2.3.1.7) (Carnitine acetylase) (CAT), (Carnitine acetyltransferase) (CrAT).]	ER
P43308	[Translocon-associated protein subunit beta precursor (TRAP-beta), (Signal sequence receptor subunit beta) (SSR-beta).]	ER
P46663	B1 bradykinin receptor (BK-1 receptor) (B1R).	ER
P50454	[Serpin H1 precursor (Collagen-binding protein) (Colligin) (47 kDa heat, shock protein) (Rheumatoid arthritis-related antigen RA-A47) (Arsenic-, transactivated protein 3) (AsTP3) (Cell proliferation-inducing gene 14, protein).]	ER
Q14554	[Protein disulfide-isomerase A5 precursor (EC 5.3.4.1) (Protein, disulfide isomerase-related protein).]	ER
Q15397	[Pumilio domain-containing protein KIAA0020 (HBV X-transactivated gene, 5 protein) (Minor histocompatibility antigen HA-8) (HLA-HA8).]	ER
Q15738	[Sterol-4-alpha-carboxylate 3-dehydrogenase, decarboxylating, (EC 1.1.1.170) (Protein H105e3).]	ER
Q16799	Reticulon-1 (Neuroendocrine-specific protein).	ER
Q92535	[Phosphatidylinositol N-acetylglucosaminyltransferase subunit C, (EC 2.4.1.198) (Phosphatidylinositol-glycan biosynthesis class C, protein) (PIG-C).]	ER
P04275	[von Willebrand factor precursor (vWF) [Contains: von Willebrand, antigen 2 (von Willebrand antigen II)].]	ER
P35610	[Sterol O-acyltransferase 1 (EC 2.3.1.26) (Cholesterol acyltransferase, 1) (Acyl coenzyme A:cholesterol acyltransferase 1) (ACAT-1).]	ER
P37059	[Estradiol 17-beta-dehydrogenase 2 (EC 1.1.1.62) (Testosterone 17-beta-, dehydrogenase) (EC 1.1.1.63) (17-beta-HSD 2) (Microsomal 17-beta-, hydroxysteroid dehydrogenase) (20 alpha-hydroxysteroid dehydrogenase), (20-alpha-HSD) (E2DH).]	ER
Q93084	[Sarcoplasmic/endoplasmic reticulum calcium ATPase 3 (EC 3.6.3.8), (Calcium pump 3) (SERCA3) (SR Ca(2+)-ATPase 3).]	ER
Q99943	[1-acyl-sn-glycerol-3-phosphate acyltransferase alpha (EC 2.3.1.51) (1-, AGP acyltransferase 1) (1-AGPAT 1) (Lysophosphatidic acid, acyltransferase alpha) (LPAAT-alpha) (1-acylglycerol-3-phosphate O-, acyltransferase 1) (Protein G15).]	ER
Q9UHB9	Signal recognition particle 68 kDa protein (SRP68).	ER
Q9Y2B2	[N-acetylglucosaminyl-phosphatidylinositol de-N-acetylase (EC 3.5.1.89), (Phosphatidylinositol-glycan biosynthesis class L protein) (PIG-L).]	ER
O15537	Retinoschisin precursor (X-linked juvenile retinoschisis protein).	Extracellular
O43915	[Vascular endothelial growth factor D precursor (VEGF-D) (c-fos-induced, growth factor) (FIGF).]	Extracellular
O75636	[Ficolin-3 precursor (Collagen/fibrinogen domain-containing protein 3), (Collagen/fibrinogen domain-containing lectin 3 p35) (Hakata antigen).]	Extracellular
O75882	Attractin precursor (Mahogany homolog) (DPPT-L).	Extracellular
P08254	[Stromelysin-1 precursor (EC 3.4.24.17) (Matrix metalloproteinase-3), (MMP-3) (Transin-1) (SL-1).]	Extracellular
P08519	Apolipoprotein(a) precursor (EC 3.4.21.-) (Apo(a)) (Lp(a)).	Extracellular
P09486	[SPARC precursor (Secreted protein acidic and rich in cysteine), (Osteonectin) (ON) (Basement-membrane protein 40) (BM-40).]	Extracellular
P10451	[Osteopontin precursor (Bone sialoprotein 1) (Secreted phosphoprotein, 1) (SPP-1) (Urinary stone protein) (Nephropontin) (Uropontin).]	Extracellular
P11487	[INT-2 proto-oncogene protein precursor (Fibroblast growth factor 3), (FGF-3) (HBGF-3).]	Extracellular
P12018	[Immunoglobulin iota chain precursor (V(pre)B protein) (VpreB protein), (CD179a antigen).]	Extracellular

P12259	[Coagulation factor V precursor (Activated protein C cofactor), [Contains: Coagulation factor V heavy chain; Coagulation factor V, light chain].]	Extracellular
P13521	[Secretogranin-2 precursor (Secretogranin II) (SgII) (Chromogranin-C), [Contains: Secretoneurin (SN)].]	Extracellular
P13611	[Versican core protein precursor (Large fibroblast proteoglycan), (Chondroitin sulfate proteoglycan core protein 2) (PG-M) (Glial, hyaluronate-binding protein) (GHAP).]	Extracellular
P13725	Oncostatin-M precursor (OSM).	Extracellular
P14780	[Matrix metalloproteinase-9 precursor (EC 3.4.24.35) (MMP-9) (92 kDa, type IV collagenase) (92 kDa gelatinase) (Gelatinase B) (GELB), [Contains: 67 kDa matrix metalloproteinase-9; 82 kDa matrix, metalloproteinase-9].]	Extracellular
P15692	[Vascular endothelial growth factor A precursor (VEGF-A) (Vascular, permeability factor) (VPF).]	Extracellular
P17948	[Vascular endothelial growth factor receptor 1 precursor (EC 2.7.10.1), (VEGFR-1) (Vascular permeability factor receptor) (Tyrosine-protein, kinase receptor FLT) (Flt-1) (Tyrosine-protein kinase FRT) (Fms-like, tyrosine kinase 1).]	Extracellular
P18136	Ig kappa chain V-III region HIC precursor.	Extracellular
P18510	[Interleukin-1 receptor antagonist protein precursor (IL-1ra) (IRAP), (IL1 inhibitor) (IL-1RN) (ICIL-1RA) (Anakinra).]	Extracellular
P19883	Follistatin precursor (FS) (Activin-binding protein).	Extracellular
P49908	Selenoprotein P precursor (SeP).	Extracellular
P54317	Pancreatic lipase-related protein 2 precursor (EC 3.1.1.3).	Extracellular
P56703	Proto-oncogene protein Wnt-3 precursor.	Extracellular
Q14116	[Interleukin-18 precursor (IL-18) (Interferon-gamma-inducing factor), (IFN-gamma-inducing factor) (Interleukin-1 gamma) (IL-1 gamma), (Iboctadekin).]	Extracellular
Q14624	[Inter-alpha-trypsin inhibitor heavy chain H4 precursor (ITI heavy, chain H4) (Inter-alpha-inhibitor heavy chain 4) (Inter-alpha-trypsin, inhibitor family heavy chain-related protein) (IHRP) (Plasma, kallikrein sensitive glycoprotein 120) (PK-120) (GP120) [Contains: 70, kDa inter-alpha-trypsin inhibitor heavy chain H4; 35 kDa inter-alpha-, trypsin inhibitor heavy chain H4].]	Extracellular
Q15166	Serum paraoxonase/lactonase 3 (EC 3.1.1.-).	Extracellular
Q16610	Extracellular matrix protein 1 precursor (Secretory component p85).	Extracellular
Q16674	[Melanoma-derived growth regulatory protein precursor (Melanoma, inhibitory activity).]	Extracellular
Q92824	[Proprotein convertase subtilisin/kexin type 5 precursor (EC 3.4.21.-), (Proprotein convertase PC5) (Subtilisin/kexin-like protease PC5) (PC6), (hPC6).]	Extracellular
O95158	Neurexophilin-4 precursor.	Extracellular
P00738	[Haptoglobin precursor [Contains: Haptoglobin alpha chain; Haptoglobin, beta chain].]	Extracellular
P00742	[Coagulation factor X precursor (EC 3.4.21.6) (Stuart factor) (Stuart-, Prower factor) [Contains: Factor X light chain; Factor X heavy chain;, Activated factor Xa heavy chain].]	Extracellular
P00748	[Coagulation factor XII precursor (EC 3.4.21.38) (Hageman factor) (HAF), [Contains: Coagulation factor XIIa heavy chain; Beta-factor XIIa part, 1; Beta-factor XIIa part 2; Coagulation factor XIIa light chain].]	Extracellular
P00797	Renin precursor (EC 3.4.23.15) (Angiotensinogenase).	Extracellular
P01009	[Alpha-1-antitrypsin precursor (Alpha-1 protease inhibitor) (Alpha-1-, antiproteinase).]	Extracellular
P01023	Alpha-2-macroglobulin precursor (Alpha-2-M).	Extracellular
P01137	[Transforming growth factor beta-1 precursor (TGF-beta-1) [Contains:, Latency-associated peptide (LAP)].]	Extracellular
P01567	[Interferon alpha-7 precursor (Interferon alpha-J1) (IFN-alpha-J1), (Interferon alpha-J) (LeIF J).]	Extracellular
P01709	Ig lambda chain V-II region MGC.	Extracellular

P01769	Ig heavy chain V-III region GA.	Extracellular
P01779	Ig heavy chain V-III region TUR.	Extracellular
P02647	[Apolipoprotein A-I precursor (Apo-AI) (ApoA-I) [Contains:, Apolipoprotein A-I(1-242)].]	Extracellular
P02747	Complement C1q subcomponent subunit C precursor.	Extracellular
P02750	Leucine-rich alpha-2-glycoprotein precursor (LRG).	Extracellular
P02760	[AMBP protein precursor [Contains: Alpha-1-microglobulin (Protein HC), (Complex-forming glycoprotein heterogeneous in charge) (Alpha-1, microglycoprotein); Inter-alpha-trypsin inhibitor light chain (ITI-LC), (Bikunin) (HI-30)].]	Extracellular
P02766	Transthyretin precursor (Prealbumin) (TBPA) (TTR) (ATTR).	Extracellular
P02774	[Vitamin D-binding protein precursor (DBP) (Group-specific component), (Gc-globulin) (VDB).]	Extracellular
P03951	[Coagulation factor XI precursor (EC 3.4.21.27) (Plasma thromboplastin, antecedent) (PTA) (FXI) [Contains: Coagulation factor XIa heavy chain;, Coagulation factor XIa light chain].]	Extracellular
P04004	[Vitronectin precursor (Serum-spreading factor) (S-protein) (V75), [Contains: Vitronectin V65 subunit; Vitronectin V10 subunit;, Somatomedin-B].]	Extracellular
P04196	[Histidine-rich glycoprotein precursor (Histidine-proline-rich, glycoprotein) (HPRG).]	Extracellular
P04207	Ig kappa chain V-III region CLL precursor (Rheumatoid factor).	Extracellular
P04278	[Sex hormone-binding globulin precursor (SHBG) (Sex steroid-binding, protein) (SBP) (Testis-specific androgen-binding protein) (ABP), (Testosterone-estrogen-binding globulin) (Testosterone-estradiol-, binding globulin) (TeBG).]	Extracellular
P05090	Apolipoprotein D precursor (Apo-D) (ApoD).	Extracellular
P06280	[Alpha-galactosidase A precursor (EC 3.2.1.22) (Melibiase) (Alpha-D-, galactoside galactohydrolase) (Alpha-D-galactosidase A) (Agalsidase, alfa).]	Extracellular
P06314	Ig kappa chain V-IV region B17 precursor.	Extracellular
P07093	[Glia-derived nexin precursor (GDN) (Protease nexin I) (PN-1) (Protease, inhibitor 7).]	Extracellular
P07225	Vitamin K-dependent protein S precursor.	Extracellular
P07477	[Trypsin-1 precursor (EC 3.4.21.4) (Trypsin I) (Cationic trypsinogen), (Serine protease 1).]	Extracellular
P07602	[Proactivator polypeptide precursor [Contains: Saposin-A (Protein A);, Saposin-B-Val; Saposin-B (Sphingolipid activator protein 1) (SAP-1), (Cerebroside sulfate activator) (CSAct) (Dispersin) (Sulfatide/GM1, activator); Saposin-C (Co-beta-glucosidase) (A1 activator), (Glucosylceramidase activator) (Sphingolipid activator protein 2), (SAP-2); Saposin-D (Protein C) (Component C)].]	Extracellular
P21810	Biglycan precursor (Bone/cartilage proteoglycan I) (PG-S1).	Extracellular
P22362	[Small-inducible cytokine A1 precursor (CCL1) (T lymphocyte-secreted, protein I-309).]	Extracellular
P22614	Putative serum amyloid A-3 protein.	Extracellular
P24347	[Stromelysin-3 precursor (EC 3.4.24.-) (ST3) (SL-3) (Matrix, metalloproteinase-11) (MMP-11).]	Extracellular
P80421	Ig heavy chain V-I region DOT.	Extracellular
P80748	Ig lambda chain V-III region LOI.	Extracellular
P35030	[Trypsin-3 precursor (EC 3.4.21.4) (Trypsin III) (Brain trypsinogen), (Mesotrypsinogen) (Trypsin IV) (Serine protease 3) (Serine protease, 4).]	Extracellular
Q9BZM1	[Group XIIA secretory phospholipase A2 precursor (EC 3.1.1.4), (Phosphatidylcholine 2-acylhydrolase GXII) (GXII sPLA2).]	Extracellular
P98095	Fibulin-2 precursor.	Extracellular

Q02297	[Pro-neuregulin-1, membrane-bound isoform precursor (Pro-NRG1), [Contains: Neuregulin-1 (Neu differentiation factor) (Heregulin) (HRG), (Breast cancer cell differentiation factor p45) (Acetylcholine, receptor-inducing activity) (ARIA) (Sensory and motor neuron-derived, factor) (Glial growth factor)].]	Extracellular
Q02747	[Guanylin precursor (Guanylate cyclase activator 2A) (Guanylate, cyclase-activating protein 1) (Gap-I) [Contains: HMW-guanylin;, Guanylin].]	Extracellular
Q02985	[Complement factor H-related protein 3 precursor (FHR-3) (H factor-like, protein 3) (DOWN16).]	Extracellular
Q03169	[Tumor necrosis factor, alpha-induced protein 2 (Primary response gene, B94 protein).]	Extracellular
Q06828	[Fibromodulin precursor (FM) (Collagen-binding 59 kDa protein) (Keratan, sulfate proteoglycan fibromodulin) (KSPG fibromodulin).]	Extracellular
Q99435	[Protein kinase C-binding protein NELL2 precursor (NEL-like protein 2), (Nel-related protein 2).]	Extracellular
Q9H239	[Matrix metalloproteinase-28 precursor (EC 3.4.24.-) (MMP-28), (Epilysin).]	Extracellular
Q9UBP4	Dickkopf-related protein 3 precursor (Dkk-3) (Dickkopf-3) (hDkk-3).	Extracellular
Q9UGM5	Fetuin-B precursor (Gugu) (IRL685) (16G2).	Extracellular
Q9UKU9	Angiopoietin-related protein 2 precursor (Angiopoietin-like 2).	Extracellular
O14791	[Apolipoprotein-L1 precursor (Apolipoprotein L-I) (Apolipoprotein L), (ApoL-I) (Apo-L) (ApoL).]	Extracellular
O60258	Fibroblast growth factor 17 precursor (FGF-17).	Extracellular
P08493	[Matrix Gla protein precursor (MGP) (Cell growth-inhibiting gene 36, protein).]	Extracellular
P08603	Complement factor H precursor (H factor 1).	Extracellular
P09038	[Heparin-binding growth factor 2 precursor (HBGF-2) (Basic fibroblast, growth factor) (BFGF) (Prostatropin).]	Extracellular
P09238	[Stromelysin-2 precursor (EC 3.4.24.22) (Matrix metalloproteinase-10), (MMP-10) (Transin-2) (SL-2).]	Extracellular
P09341	[Growth-regulated protein alpha precursor (CXCL1) (Melanoma growth, stimulatory activity) (MGSA) (Neutrophil-activating protein 3) (NAP-3), (GRO-alpha(1-73)) [Contains: GRO-alpha(4-73); GRO-alpha(5-73); GRO-, alpha(6-73)].]	Extracellular
P09544	Protein Wnt-2 precursor (IRP protein) (Int-1-related protein).	Extracellular
P10153	[Non-secretory ribonuclease precursor (EC 3.1.27.5) (Ribonuclease US), (Eosinophil-derived neurotoxin) (RNase UpI-2) (Ribonuclease 2) (RNase, 2).]	Extracellular
P10909	[Clusterin precursor (Complement-associated protein SP-40,40), (Complement cytolysis inhibitor) (CLI) (NA1/NA2) (Apolipoprotein J), (Apo-J) (Testosterone-repressed prostate message 2) (TRPM-2), [Contains: Clusterin beta chain (ApoJalpha) (Complement cytolysis, inhibitor a chain); Clusterin alpha chain (ApoJbeta) (Complement, cytolysis inhibitor b chain)].]	Extracellular
P10915	[Hyaluronan and proteoglycan link protein 1 precursor (Proteoglycan, link protein) (Cartilage link protein) (LP).]	Extracellular
P11226	[Mannose-binding protein C precursor (MBP-C) (MBP1) (Mannan-binding, protein) (Mannose-binding lectin).]	Extracellular
P12034	Fibroblast growth factor 5 precursor (FGF-5) (HBGF-5) (Smag-82).	Extracellular
P12110	Collagen alpha-2(VI) chain precursor.	Extracellular
P13727	[Bone marrow proteoglycan precursor (BMPG) (Proteoglycan 2) [Contains:, Eosinophil granule major basic protein (EMBP) (MBP) (Pregnancy-, associated major basic protein)].]	Extracellular
P14735	[Insulin-degrading enzyme (EC 3.4.24.56) (Insulysin) (Insulinase), (Insulin protease).]	Extracellular
P15169	[Carboxypeptidase N catalytic chain precursor (EC 3.4.17.3) (CPN), (Carboxypeptidase N polypeptide 1) (Carboxypeptidase N small subunit), (Lysine carboxypeptidase) (Arginine carboxypeptidase) (Kininase-1), (Serum carboxypeptidase N) (SCPN) (Anaphylatoxin inactivator) (Plasma, carboxypeptidase B).]	Extracellular

P16442	[Histo-blood group ABO system transferase (NAGAT) [Includes:, Glycoprotein-fucosylgalactoside alpha-N-, acetylgalactosaminyltransferase (EC 2.4.1.40) (Fucosylglycoprotein, alpha-N-acetylgalactosaminyltransferase) (Histo-blood group A, transferase) (A transferase); Glycoprotein-fucosylgalactoside alpha-, galactosyltransferase (EC 2.4.1.37) (Fucosylglycoprotein 3-alpha-, galactosyltransferase) (Histo-blood group B transferase) (B, transferase)] [Contains: Fucosylglycoprotein alpha-N-, acetylgalactosaminyltransferase; Fucosylglycoprotein alpha-N-, acetylgalactosaminyltransferase soluble form].]	Extracellular
P17936	[Insulin-like growth factor-binding protein 3 precursor (IGFBP-3) (IBP-, 3) (IGF-binding protein 3).]	Extracellular
P19823	[Inter-alpha-trypsin inhibitor heavy chain H2 precursor (ITI heavy, chain H2) (Inter-alpha-inhibitor heavy chain 2) (Inter-alpha-trypsin, inhibitor complex component II) (Serum-derived hyaluronan-associated, protein) (SHAP).]	Extracellular
P20366	[Protachykinin 1 precursor (PPT) [Contains: Substance P; Neurokinin A, (NKA) (Substance K) (Neuromedin L); Neuropeptide K (NPK); Neuropeptide, gamma; C-terminal-flanking peptide].]	Extracellular
P20742	Pregnancy zone protein precursor.	Extracellular
P56706	Protein Wnt-7b precursor.	Extracellular
P56975	[Pro-neuregulin-3, membrane-bound isoform precursor (Pro-NRG3), [Contains: Neuregulin-3 (NRG-3)].]	Extracellular
Q14563	Semaphorin-3A precursor (Semaphorin III) (Sema III).	Extracellular
Q15582	[Transforming growth factor-beta-induced protein ig-h3 precursor (Beta, ig-h3) (Kerato-epithelin) (RGD-containing collagen-associated protein), (RGD-CAP).]	Extracellular
Q16619	Cardiotrophin-1 (CT-1).	Extracellular
Q16651	[Prostasin precursor (EC 3.4.21.-) (Serine protease 8) [Contains:, Prostasin light chain; Prostasin heavy chain].]	Extracellular
Q16819	[Meprin A subunit alpha precursor (EC 3.4.24.18) (Endopeptidase-2) (N-, benzoyl-L-tyrosyl-P-amino-benzoic acid hydrolase subunit alpha) (PABA, peptide hydrolase) (PPH alpha).]	Extracellular
O76093	Fibroblast growth factor 18 precursor (FGF-18) (zFGF5).	Extracellular
O95399	Urotensin-2 precursor (Urotensin-II) (U-II) (UII).	Extracellular
P00450	Ceruloplasmin precursor (EC 1.16.3.1) (Ferroxidase).	Extracellular
P00734	[Prothrombin precursor (EC 3.4.21.5) (Coagulation factor II) [Contains:, Activation peptide fragment 1; Activation peptide fragment 2; Thrombin, light chain; Thrombin heavy chain].]	Extracellular
P01011	[Alpha-1-antichymotrypsin precursor (ACT) (Cell growth-inhibiting gene, 24/25 protein) [Contains: Alpha-1-antichymotrypsin His-Pro-less].]	Extracellular
P01042	[Kininogen-1 precursor (Alpha-2-thiol proteinase inhibitor) [Contains:, Kininogen-1 heavy chain; Bradykinin (Kallidin I); Lysyl-bradykinin, (Kallidin II); Kininogen-1 light chain; Low molecular weight growth-, promoting factor].]	Extracellular
P01266	Thyroglobulin precursor.	Extracellular
P01574	Interferon beta precursor (IFN-beta) (Fibroblast interferon).	Extracellular
P01591	Immunoglobulin J chain.	Extracellular
P01624	Ig kappa chain V-III region POM.	Extracellular
P01717	Ig lambda chain V-IV region Hil.	Extracellular
P01771	Ig heavy chain V-III region HIL.	Extracellular
P01814	Ig heavy chain V-II region OU.	Extracellular
P02655	Apolipoprotein C-II precursor (Apo-CII) (ApoC-II).	Extracellular

P02735	[Serum amyloid A protein precursor (SAA) [Contains: Amyloid protein A, (Amyloid fibril protein AA); Serum amyloid protein A(2-104); Serum, amyloid protein A(3-104); Serum amyloid protein A(2-103); Serum, amyloid protein A(2-102); Serum amyloid protein A(4-101)].]	Extracellular
P02763	Alpha-1-acid glycoprotein 1 precursor (AGP 1) (Orosomucoid-1) (OMD 1).	Extracellular
P02768	Serum albumin precursor.	Extracellular
P02790	Hemopexin precursor (Beta-1B-glycoprotein).	Extracellular
P04070	[Vitamin K-dependent protein C precursor (EC 3.4.21.69), (Autoprothrombin IIA) (Anticoagulant protein C) (Blood coagulation, factor XIV) [Contains: Vitamin K-dependent protein C light chain;, Vitamin K-dependent protein C heavy chain; Activation peptide].]	Extracellular
P04180	[Phosphatidylcholine-sterol acyltransferase precursor (EC 2.3.1.43), (Lecithin-cholesterol acyltransferase) (Phospholipid-cholesterol, acyltransferase).]	Extracellular
P04279	[Semenogelin-1 precursor (Semenogelin I) (SGI) [Contains: Alpha-, inhibin-92; Alpha-inhibin-31; Seminal basic protein].]	Extracellular
P04434	Ig kappa chain V-III region VH precursor (Fragment).	Extracellular
P05112	[Interleukin-4 precursor (IL-4) (B-cell stimulatory factor 1) (BSF-1), (Lymphocyte stimulatory factor 1) (Binetrakin) (Pitrakinra).]	Extracellular
P05154	[Plasma serine protease inhibitor precursor (PCI) (Protein C inhibitor), (Serpin A5) (Plasminogen activator inhibitor 3) (PAI-3) (PAI3), (Acrosomal serine protease inhibitor).]	Extracellular
P05160	[Coagulation factor XIII B chain precursor (Protein-glutamine gamma-, glutamyltransferase B chain) (Transglutaminase B chain) (Fibrin-, stabilizing factor B subunit).]	Extracellular
P05452	[Tetranectin precursor (TN) (C-type lectin domain family 3 member B), (Plasminogen kringle 4-binding protein).]	Extracellular
P05543	[Thyroxine-binding globulin precursor (T4-binding globulin) (Serpin, A7).]	Extracellular
P06309	Ig kappa chain V-II region GM607 precursor (Fragment).	Extracellular
P06326	Ig heavy chain V-I region Mot.	Extracellular
P06727	Apolipoprotein A-IV precursor (Apo-AIV) (ApoA-IV).	Extracellular
P07288	[Prostate-specific antigen precursor (EC 3.4.21.77) (PSA) (Kallikrein-, 3) (Semenogelase) (Gamma-seminoprotein) (Seminin) (P-30 antigen).]	Extracellular
P07492	[Gastrin-releasing peptide precursor (GRP) [Contains: Neuromedin-C, (GRP-10)].]	Extracellular
P22894	[Neutrophil collagenase precursor (EC 3.4.24.34) (Matrix, metalloproteinase-8) (MMP-8) (PMNL collagenase) (PMNL-CL).]	Extracellular
P23142	Fibulin-1 precursor.	Extracellular
P24001	[Interleukin-32 precursor (IL-32) (Natural killer cells protein 4), (Tumor necrosis factor alpha-inducing factor).]	Extracellular
P80362	Ig kappa chain V-I region WAT.	Extracellular
P80697	Synovial stimulatory protein p205 (Fragments).	Extracellular
P29460	[Interleukin-12 subunit beta precursor (IL-12B) (IL-12 subunit p40), (Cytotoxic lymphocyte maturation factor 40 kDa subunit) (CLMF p40) (NK, cell stimulatory factor chain 2) (NKSF2).]	Extracellular
P31371	[Glia-activating factor precursor (GAF) (Fibroblast growth factor 9), (FGF-9) (HBGF-9).]	Extracellular
P35052	Glypican-1 precursor.	Extracellular
P35858	[Insulin-like growth factor-binding protein complex acid labile chain, precursor (ALS).]	Extracellular
P36222	[Chitinase-3-like protein 1 precursor (Cartilage glycoprotein 39) (GP-, 39) (39 kDa synovial protein) (HCgp-39) (YKL-40).]	Extracellular
P36955	[Pigment epithelium-derived factor precursor (PEDF) (Serpin-F1) (EPC-, 1).]	Extracellular
P41221	Protein Wnt-5a precursor.	Extracellular
Q01113	Interleukin-9 receptor precursor (IL-9R) (CD129 antigen).	Extracellular

Q04756	[Hepatocyte growth factor activator precursor (EC 3.4.21.-) (HGF, activator) (HGFA) [Contains: Hepatocyte growth factor activator short, chain; Hepatocyte growth factor activator long chain].]	Extracellular
Q04762	Very putative protein CMAR.	Extracellular
Q06033	[Inter-alpha-trypsin inhibitor heavy chain H3 precursor (ITI heavy, chain H3) (Inter-alpha-inhibitor heavy chain 3) (Serum-derived, hyaluronan-associated protein) (SHAP).]	Extracellular
Q07325	[Small-inducible cytokine B9 precursor (CXCL9) (Gamma-interferon-, induced monokine) (MIG).]	Extracellular
Q13253	Noggin precursor.	Extracellular
Q13316	[Dentin matrix acidic phosphoprotein 1 precursor (Dentin matrix protein, 1) (DMP-1).]	Extracellular
Q99972	[Myocilin precursor (Trabecular meshwork-induced glucocorticoid, response protein).]	Extracellular
Q9GZV9	[Fibroblast growth factor 23 precursor (FGF-23) (Tumor-derived, hypophosphatemia-inducing factor).]	Extracellular
O00339	Matrilin-2 precursor.	Extracellular
O00548	[Delta-like protein 1 precursor (Drosophila Delta homolog 1) (Delta1), (H-Delta-1).]	Extracellular
O15041	Semaphorin-3E precursor.	Extracellular
O60235	[Transmembrane protease, serine 11D precursor (EC 3.4.21.-) (Airway, trypsin-like protease) [Contains: Transmembrane protease, serine 11D, non-catalytic chain; Transmembrane protease, serine 11D catalytic, chain].]	Extracellular
P07996	Thrombospondin-1 precursor.	Extracellular
P08253	[72 kDa type IV collagenase precursor (EC 3.4.24.24) (72 kDa, gelatinase) (Matrix metalloproteinase-2) (MMP-2) (Gelatinase A) (TBE-, 1).]	Extracellular
P08620	[Fibroblast growth factor 4 precursor (FGF-4) (Heparin secretory-, transforming protein) (HST-1) (HST) (Transforming protein KS3) (HBGF-, 4).]	Extracellular
P08697	[Alpha-2-antiplasmin precursor (Alpha-2-plasmin inhibitor) (Alpha-2-PI), (Alpha-2-AP).]	Extracellular
P08887	[Interleukin-6 receptor alpha chain precursor (IL-6R-alpha) (IL-6R 1), (Membrane glycoprotein 80) (gp80) (CD126 antigen).]	Extracellular
P12724	[Eosinophil cationic protein precursor (EC 3.1.27.-) (ECP), (Ribonuclease 3) (RNase 3).]	Extracellular
P15514	[Amphiregulin precursor (AR) (Colorectum cell-derived growth factor), (CRDGF).]	Extracellular
P18428	Lipopolysaccharide-binding protein precursor (LBP).	Extracellular
P19827	[Inter-alpha-trypsin inhibitor heavy chain H1 precursor (ITI heavy, chain H1) (Inter-alpha-inhibitor heavy chain 1) (Inter-alpha-trypsin, inhibitor complex component III) (Serum-derived hyaluronan-associated, protein) (SHAP).]	Extracellular
P20382	[Pro-MCH precursor [Contains: Neuropeptide-glycine-glutamic acid (NGE), (Neuropeptide G-E); Neuropeptide-glutamic acid-isoleucine (NEI), (Neuropeptide E-I); Melanin-concentrating hormone (MCH)].]	Extracellular
P43652	Afamin precursor (Alpha-albumin) (Alpha-Alb).	Extracellular
P45452	[Collagenase 3 precursor (EC 3.4.24.-) (Matrix metalloproteinase-13), (MMP-13).]	Extracellular
P55058	Phospholipid transfer protein precursor (Lipid transfer protein II).	Extracellular
Q14129	Protein DGCR6 (DiGeorge syndrome critical region 6).	Extracellular
Q15782	[Chitinase-3-like protein 2 precursor (YKL-39) (Chondrocyte protein, 39).]	Extracellular
Q8IZU9	[Kin of IRRE-like protein 3 precursor (Kin of irregular chiasm-like, protein 3) (Nephrin-like 2).]	Extracellular
Q92520	Protein FAM3C precursor (Protein GS3786).	Extracellular
P26022	[Pentraxin-related protein PTX3 precursor (Pentaxin-related protein, PTX3) (Tumor necrosis factor-inducible protein TSG-14).]	Extracellular
P00451	[Coagulation factor VIII precursor (Procoagulant component), (Antihemophilic factor) (AHF) [Contains: Factor VIIIa heavy chain, 200, kDa isoform; Factor VIIIa heavy chain, 92 kDa isoform; Factor VIII B, chain; Factor VIIIa light chain].]	Extracellular

P00736	[Complement C1r subcomponent precursor (EC 3.4.21.41) (Complement, component 1, r subcomponent) [Contains: Complement C1r subcomponent, heavy chain; Complement C1r subcomponent light chain].]	Extracellular
P00740	[Coagulation factor IX precursor (EC 3.4.21.22) (Christmas factor), (Plasma thromboplastin component) (PTC) [Contains: Coagulation factor, IXa light chain; Coagulation factor IXa heavy chain].]	Extracellular
P00747	[Plasminogen precursor (EC 3.4.21.7) [Contains: Plasmin heavy chain A;, Activation peptide; Angiostatin; Plasmin heavy chain A, short form;, Plasmin light chain B].]	Extracellular
P01008	Antithrombin-III precursor (ATIII).	Extracellular
P01019	[Angiotensinogen precursor (Serpin A8) [Contains: Angiotensin-1, (Angiotensin I) (Ang I); Angiotensin-2 (Angiotensin II) (Ang II);, Angiotensin-3 (Angiotensin III) (Ang III) (Des-Asp[1]-angiotensin, II)].]	Extracellular
P01031	[Complement C5 precursor [Contains: Complement C5 beta chain;, Complement C5 alpha chain; C5a anaphylatoxin; Complement C5 alpha', chain].]	Extracellular
P01270	Parathyroid hormone precursor (Parathyrin) (PTH) (Parathormone).	Extracellular
P01603	Ig kappa chain V-I region Ka.	Extracellular
P01625	Ig kappa chain V-IV region Len.	Extracellular
P01718	Ig lambda chain V-IV region Kern.	Extracellular
P01760	Ig heavy chain V-I region WOL.	Extracellular
P01777	Ig heavy chain V-III region TEI.	Extracellular
P01825	Ig heavy chain V-II region NEWM.	Extracellular
P02652	[Apolipoprotein A-II precursor (Apo-AII) (ApoA-II) [Contains:, Apolipoprotein A-II(1-76)].]	Extracellular
P02749	[Beta-2-glycoprotein 1 precursor (Beta-2-glycoprotein I), (Apolipoprotein H) (Apo-H) (B2GPI) (Beta(2)GPI) (Activated protein C-, binding protein) (APC inhibitor) (Anticardiolipin cofactor).]	Extracellular
P02753	[Plasma retinol-binding protein precursor (PRBP) (RBP) [Contains:, Plasma retinol-binding protein(1-182); Plasma retinol-binding, protein(1-181); Plasma retinol-binding protein(1-179); Plasma retinol-, binding protein(1-176)].]	Extracellular
P02765	[Alpha-2-HS-glycoprotein precursor (Fetuin-A) (Alpha-2-Z-globulin) (Ba-, alpha-2-glycoprotein) [Contains: Alpha-2-HS-glycoprotein chain A;, Alpha-2-HS-glycoprotein chain B].]	Extracellular
P04003	[C4b-binding protein alpha chain precursor (C4bp) (Proline-rich, protein) (PRP).]	Extracellular
P04206	Ig kappa chain V-III region GOL (Rheumatoid factor).	Extracellular
P04217	Alpha-1B-glycoprotein precursor (Alpha-1-B glycoprotein).	Extracellular
P05155	[Plasma protease C1 inhibitor precursor (C1 Inh) (C1Inh) (C1 esterase, inhibitor) (C1-inhibiting factor).]	Extracellular
P05305	[Endothelin-1 precursor (Preproendothelin-1) (PPET1) [Contains:, Endothelin-1 (ET-1); Big endothelin-1].]	Extracellular
P05546	[Heparin cofactor 2 precursor (Heparin cofactor II) (HC-II) (Protease, inhibitor leuserpin 2) (HLS2).]	Extracellular
P06276	[Cholinesterase precursor (EC 3.1.1.8) (Acylcholine acylhydrolase), (Choline esterase II) (Butyrylcholine esterase), (Pseudocholinesterase).]	Extracellular
P06310	Ig kappa chain V-II region RPMI 6410 precursor.	Extracellular
P06681	[Complement C2 precursor (EC 3.4.21.43) (C3/C5 convertase) [Contains:, Complement C2b fragment; Complement C2a fragment].]	Extracellular
P06858	Lipoprotein lipase precursor (EC 3.1.1.34) (LPL).	Extracellular
P07357	[Complement component C8 alpha chain precursor (Complement component 8, subunit alpha).]	Extracellular
P21941	Cartilage matrix protein precursor (Matrilin-1).	Extracellular
P22301	[Interleukin-10 precursor (IL-10) (Cytokine synthesis inhibitory, factor) (CSIF).]	Extracellular

P22352	[Glutathione peroxidase 3 precursor (EC 1.11.1.9) (GSHPx-3) (GPx-3), (Plasma glutathione peroxidase) (GSHPx-P) (Extracellular glutathione, peroxidase) (GPx-P).]	Extracellular
P22792	[Carboxypeptidase N subunit 2 precursor (Carboxypeptidase N polypeptide, 2) (Carboxypeptidase N 83 kDa chain) (Carboxypeptidase N regulatory, subunit) (Carboxypeptidase N large subunit).]	Extracellular
P23352	[Anosmin-1 precursor (Kallmann syndrome protein) (Adhesion molecule-, like X-linked).]	Extracellular
P24593	[Insulin-like growth factor-binding protein 5 precursor (IGFBP-5) (IBP-, 5) (IGF-binding protein 5).]	Extracellular
P25311	[Zinc-alpha-2-glycoprotein precursor (Zn-alpha-2-glycoprotein) (Zn-, alpha-2-GP).]	Extracellular
P80370	[Delta-like protein precursor (DLK) (pG2) [Contains: Fetal antigen 1, (FA1)].]	Extracellular
P27169	[Serum paraoxonase/arylesterase 1 (EC 3.1.1.2) (EC 3.1.8.1) (PON 1), (Serum aryldialkylphosphatase 1) (A-esterase 1) (Aromatic esterase 1), (K-45).]	Extracellular
P27918	Properdin precursor (Complement factor P).	Extracellular
P35556	Fibrillin-2 precursor.	Extracellular
P41222	[Prostaglandin-H2 D-isomerase precursor (EC 5.3.99.2) (Lipocalin-type, prostaglandin-D synthase) (Glutathione-independent PGD synthetase), (Prostaglandin-D2 synthase) (PGD2 synthase) (PGDS2) (PGDS) (Beta-trace, protein) (Cerebrin-28).]	Extracellular
P81605	[Dermcidin precursor (Preproteolysin) [Contains: Survival-promoting, peptide; DCD-1].]	Extracellular
Q02383	Semenogelin-2 precursor (Semenogelin II) (SGII).	Extracellular
Q13123	Protein Red (Protein RER) (IK factor) (Cytokine IK).	Extracellular
Q93091	Ribonuclease K6 precursor (EC 3.1.27.-) (RNase K6).	Extracellular
Q9NP55	[Protein Plunc precursor (Palate lung and nasal epithelium clone, protein) (Lung-specific protein X) (Nasopharyngeal carcinoma-related, protein) (Tracheal epithelium-enriched protein) (Secretory protein in, upper respiratory tracts) (Von Ebner protein Hl).]	Extracellular
Q9NYY1	Interleukin-20 precursor (IL-20) (Four alpha helix cytokine Zcyto10).	Extracellular
Q9NZV5	Selenoprotein N precursor.	Extracellular
Q9P2N4	[ADAMTS-9 precursor (EC 3.4.24.-) (A disintegrin and metalloproteinase, with thrombospondin motifs 9) (ADAM-TS 9) (ADAM-TS9).]	Extracellular
Q9UBU2	Dickkopf-related protein 2 precursor (Dkk-2) (Dickkopf-2) (hDkk-2).	Extracellular
Q15835	[Rhodopsin kinase precursor (EC 2.7.11.14) (RK) (G protein-coupled, receptor kinase 1).]	Go Component not found
O00141	[Serine/threonine-protein kinase Sgk1 (EC 2.7.11.1), (Serum/glucocorticoid-regulated kinase 1).]	Go Component not found
O00167	Eyes absent homolog 2 (EC 3.1.3.48).	Go Component not found
O00186	[Syntaxin-binding protein 3 (Unc-18 homolog 3) (Unc-18C) (Unc-18-3), (Platelet Sec1 protein) (PSP).]	Go Component not found
O00194	Ras-related protein Rab-27B (C25KG).	Go Component not found
O00321	ETS translocation variant 2 (Ets-related protein 71).	Go Component not found
O00330	[Pyruvate dehydrogenase protein X component, mitochondrial precursor, (Dihydrolipoamide dehydrogenase-binding protein of pyruvate, dehydrogenase complex) (Lipoyl-containing pyruvate dehydrogenase, complex component X) (E3-binding protein) (E3BP) (proX).]	Go Component not found
O00462	[Beta-mannosidase precursor (EC 3.2.1.25) (Lysosomal beta A, mannosidase) (Mannanase) (Mannase).]	Go Component not found
O00522	[Krev interaction trapped protein 1 (Krev interaction trapped 1), (Cerebral cavernous malformations 1 protein).]	Go Component not found
O00716	Transcription factor E2F3 (E2F-3).	Go Component not found
O00762	[Ubiquitin-conjugating enzyme E2 C (EC 6.3.2.19) (Ubiquitin-protein, ligase C) (Ubiquitin carrier protein C) (UbcH10).]	Go Component not found

O14511	[Pro-neuregulin-2, membrane-bound isoform precursor (Pro-NRG2), [Contains: Neuregulin-2 (NRG-2) (Neural- and thymus-derived activator, for ERBB kinases) (NTAK) (Divergent of neuregulin-1) (DON-1)].]	Go Component not found
O14548	[Cytochrome c oxidase subunit VIIa-related protein, mitochondrial, precursor (COX7a-related protein) (EB1).]	Go Component not found
O14607	[Ubiquitously transcribed Y chromosome tetratricopeptide repeat protein, (Ubiquitously transcribed TPR protein on the Y chromosome).]	Go Component not found
O14627	Homeobox protein CDX-4 (Caudal-type homeobox protein 4).	Go Component not found
O14653	[Golgi SNAP receptor complex member 2 (27 kDa Golgi SNARE protein), (Membrin).]	Go Component not found
O14709	[Zinc finger protein 197 (ZnF20) (Zinc finger protein with KRAB and, SCAN domains 9).]	Go Component not found
O14771	[Zinc finger protein 213 (Putative transcription factor CR53) (Zinc, finger protein with KRAB and SCAN domains 21).]	Go Component not found
O14830	[Serine/threonine-protein phosphatase with EF-hands 2 (EC 3.1.3.16), (PPEF-2).]	Go Component not found
O14867	[Transcription regulator protein BACH1 (BTB and CNC homolog 1), (HA2303).]	Go Component not found
O14917	Protocadherin-17 precursor (Protocadherin-68).	Go Component not found
O14933	[Ubiquitin/ISG15-conjugating enzyme E2 L6 (EC 6.3.2.19) (Ubiquitin-, protein ligase L6) (Ubiquitin carrier protein L6) (UbcH8) (Retinoic, acid-induced gene B protein) (RIG-B).]	Go Component not found
O14966	Ras-related protein Rab-7L1 (Rab-7-like protein 1).	Go Component not found
O14978	[Zinc finger protein 263 (Zinc finger protein FPM315) (Zinc finger, protein with KRAB and SCAN domains 12).]	Go Component not found
O15027	SEC16 homolog A.	Go Component not found
O15049	NEDD4-binding protein 3 (N4BP3).	Go Component not found
O15068	[Guanine nucleotide exchange factor DBS (DBL's big sister) (MCF2-, transforming sequence-like protein).]	Go Component not found
O15084	[Ankyrin repeat domain-containing protein 28 (Phosphatase interactor, targeting protein hnRNP K) (PITK).]	Go Component not found
O15211	[Ral guanine nucleotide dissociation stimulator-like 2 (RalGDS-like, factor) (RAS-associated protein RAB2L).]	Go Component not found
O15234	[Protein CASC3 (Cancer susceptibility candidate gene 3 protein), (Metastatic lymph node protein 51) (Protein MLN 51) (Protein barentsz), (Btz).]	Go Component not found
O15270	[Serine palmitoyltransferase 2 (EC 2.3.1.50) (Long chain base, biosynthesis protein 2) (LCB 2) (Serine-palmitoyl-CoA transferase 2), (SPT 2).]	Go Component not found
O15305	Phosphomannomutase 2 (EC 5.4.2.8) (PMM 2).	Go Component not found
O15467	[Small-inducible cytokine A16 precursor (CCL16) (IL-10-inducible, chemokine) (Chemokine LEC) (Liver-expressed chemokine) (Monotactin-1), (MTN-1) (Chemokine CC-4) (HCC-4) (NCC-4) (Lymphocyte and monocyte, chemoattractant) (LMC) (LCC-1).]	Go Component not found
O15523	[ATP-dependent RNA helicase DDX3Y (EC 3.6.1.-) (DEAD box protein 3, Y-, chromosomal).]	Go Component not found
O43167	[Zinc finger and BTB domain-containing protein 24 (Zinc finger protein, 450).]	Go Component not found
O43252	[Bifunctional 3'-phosphoadenosine 5'-phosphosulfate synthetase 1 (PAPS, synthetase 1) (PAPSS 1) (Sulfurylase kinase 1) (SK1) (SK 1) [Includes:, Sulfate adenylyltransferase (EC 2.7.7.4) (Sulfate adenylate, transferase) (SAT) (ATP-sulfurylase); Adenylyl-sulfate kinase, (EC 2.7.1.25) (Adenylylsulfate 3'-phosphotransferase) (APS kinase), (Adenosine-5'-phosphosulfate 3'-phosphotransferase) (3'-, phosphoadenosine-5'-phosphosulfate synthetase)].]	Go Component not found
O43310	Uncharacterized protein KIAA0427.	Go Component not found
O43318	[Mitogen-activated protein kinase kinase kinase 7 (EC 2.7.11.25), (Transforming growth factor-beta-activated kinase 1) (TGF-beta-, activated kinase 1).]	Go Component not found
O43365	Homeobox protein Hox-A3 (Hox-1E).	Go Component not found

O43422	[52 kDa repressor of the inhibitor of the protein kinase (p58IPK-, interacting protein) (58 kDa interferon-induced protein kinase-, interacting protein) (P52rIPK) (Death-associated protein 4) (THAP, domain-containing protein 0).]	Go Component not found
O43506	[ADAM 20 precursor (EC 3.4.24.-) (A disintegrin and metalloproteinase, domain 20).]	Go Component not found
O43572	[A kinase anchor protein 10, mitochondrial precursor (Protein kinase A-, anchoring protein 10) (PRKA10) (Dual specificity A kinase-anchoring, protein 2) (D-AKAP-2).]	Go Component not found
O43731	[ER lumen protein retaining receptor 3 (KDEL receptor 3) (KDEL, endoplasmic reticulum protein retention receptor 3).]	Go Component not found
O43815	Striatin.	Go Component not found
O60268	Uncharacterized protein KIAA0513.	Go Component not found
O60443	[Non-syndromic hearing impairment protein 5 (Inversely correlated with, estrogen receptor expression 1) (ICERE-1).]	Go Component not found
O60488	[Long-chain-fatty-acid--CoA ligase 4 (EC 6.2.1.3) (Long-chain acyl-CoA, synthetase 4) (LACS 4).]	Go Component not found
O60610	Protein diaphanous homolog 1 (Diaphanous-related formin-1) (DRF1).	Go Component not found
O60674	Tyrosine-protein kinase JAK2 (EC 2.7.10.2) (Janus kinase 2) (JAK-2).	Go Component not found
O60701	[UDP-glucose 6-dehydrogenase (EC 1.1.1.22) (UDP-Glc dehydrogenase), (UDP-GlcDH) (UDPGDH).]	Go Component not found
O60809	PRAME family member 10.	Go Component not found
O60879	Protein diaphanous homolog 2 (Diaphanous-related formin-2) (DRF2).	Go Component not found
O60911	Cathepsin L2 precursor (EC 3.4.22.43) (Cathepsin V) (Cathepsin U).	Go Component not found
O75015	[Low affinity immunoglobulin gamma Fc region receptor III-B precursor, (IgG Fc receptor III-1) (Fc-gamma RIII-beta) (Fc-gamma RIIIb), (FcRIIIb) (Fc-gamma RIII) (FcRIII) (FcR-10) (CD16b antigen).]	Go Component not found
O75069	Transmembrane and coiled-coil domains protein 2 (Cerebral protein 11).	Go Component not found
O75153	Putative eukaryotic translation initiation factor 3 subunit (eIF-3).	Go Component not found
O75293	[Growth arrest and DNA-damage-inducible protein GADD45 beta (Negative, growth regulatory protein MyD118) (Myeloid differentiation primary, response protein MyD118).]	Go Component not found
O75326	[Semaphorin-7A precursor (Semaphorin L) (Sema L) (Semaphorin K1) (Sema, K1) (John-Milton-Hargen human blood group Ag) (JMH blood group, antigen) (CD108 antigen) (CDw108).]	Go Component not found
O75343	Guanylate cyclase soluble subunit beta-2 (EC 4.6.1.2) (GCS-beta-2).	Go Component not found
O75362	Zinc finger protein 217.	Go Component not found
O75414	[Nucleoside diphosphate kinase 6 (EC 2.7.4.6) (NDK 6) (NDP kinase 6), (nm23-H6) (Inhibitor of p53-induced apoptosis-alpha) (IPIA-alpha).]	Go Component not found
O75461	Transcription factor E2F6 (E2F-6).	Go Component not found
O75534	[Cold shock domain-containing protein E1 (UNR protein) (N-ras upstream, gene protein).]	Go Component not found
O75610	[Left-right determination factor 1 precursor (Protein lefty-1) (Left-, right determination factor B) (Protein lefty-B).]	Go Component not found
O75665	Oral-facial-digital syndrome 1 protein (Protein 71-7A).	Go Component not found
O75694	[Nuclear pore complex protein Nup155 (Nucleoporin Nup155) (155 kDa, nucleoporin).]	Go Component not found
O75747	[Phosphatidylinositol-4-phosphate 3-kinase C2 domain-containing gamma, polypeptide (EC 2.7.1.154) (Phosphoinositide 3-Kinase-C2-gamma), (PtdIns-3-kinase C2 gamma) (PI3K-C2gamma).]	Go Component not found
O75840	Krueppel-like factor 7 (Ubiquitous krueppel-like factor).	Go Component not found
O75916	Regulator of G-protein signaling 9 (RGS9).	Go Component not found
P07988	[Pulmonary surfactant-associated protein B precursor (SP-B) (6 kDa, protein) (Pulmonary surfactant-associated proteolipid SPL(Phe)) (18, kDa pulmonary-surfactant protein).]	Go Component not found

P08048	Zinc finger Y-chromosomal protein.	Go Component not found
P08123	Collagen alpha-2(I) chain precursor (Alpha-2 type I collagen).	Go Component not found
P08185	[Corticosteroid-binding globulin precursor (CBG) (Transcortin) (Serpin, A6).]	Go Component not found
P09210	[Glutathione S-transferase A2 (EC 2.5.1.18) (GTH2) (HA subunit 2) (GST-, gamma) (GSTA2-2) (GST class-alpha member 2).]	Go Component not found
P09327	Villin-1.	Go Component not found
P09543	[2',3'-cyclic-nucleotide 3'-phosphodiesterase (EC 3.1.4.37) (CNP), (CNPase).]	Go Component not found
P09619	[Beta-type platelet-derived growth factor receptor precursor, (EC 2.7.10.1) (PDGF-R-beta) (CD140b antigen).]	Go Component not found
P0C0L4	[Complement C4-A precursor (Acidic complement C4) [Contains: Complement, C4 beta chain; Complement C4-A alpha chain; C4a anaphylatoxin; C4b-A;, C4d-A; Complement C4 gamma chain].]	Go Component not found
P10914	Interferon regulatory factor 1 (IRF-1).	Go Component not found
P11177	[Pyruvate dehydrogenase E1 component subunit beta, mitochondrial, precursor (EC 1.2.4.1) (PDHE1-B).]	Go Component not found
P11216	Glycogen phosphorylase, brain form (EC 2.4.1.1).	Go Component not found
P11274	[Breakpoint cluster region protein (EC 2.7.11.1) (Renal carcinoma, antigen NY-REN-26).]	Go Component not found
P11464	[Pregnancy-specific beta-1-glycoprotein 1 precursor (PSBG-1), (Pregnancy-specific beta-1 glycoprotein C/D) (PS-beta-C/D) (Fetal, liver non-specific cross-reactive antigen 1/2) (FL-NCA-1/2) (PSG95), (CD66f antigen).]	Go Component not found
P11678	[Eosinophil peroxidase precursor (EC 1.11.1.7) (EPO) [Contains:, Eosinophil peroxidase light chain; Eosinophil peroxidase heavy chain].]	Go Component not found
P12645	Bone morphogenetic protein 3 precursor (BMP-3) (Osteogenin) (BMP-3A).	Go Component not found
P12757	Ski-like protein (Ski-related protein) (Ski-related oncogene).	Go Component not found
P12931	[Proto-oncogene tyrosine-protein kinase Src (EC 2.7.10.2) (p60-Src) (c-, Src) (pp60c-src).]	Go Component not found
P13584	Cytochrome P450 4B1 (EC 1.14.14.1) (CYPIVB1) (P450-HP).	Go Component not found
P13639	Elongation factor 2 (EF-2).	Go Component not found
P13797	Plastin-3 (T-plastin).	Go Component not found
P14619	[cGMP-dependent protein kinase 1, beta isozyme (EC 2.7.11.12) (cGK 1, beta) (cGKI-beta).]	Go Component not found
P15157	Tryptase alpha-1 precursor (EC 3.4.21.59) (Tryptase-1).	Go Component not found
P15248	[Interleukin-9 precursor (IL-9) (T-cell growth factor P40) (P40, cytokine).]	Go Component not found
P15586	[N-acetylglucosamine-6-sulfatase precursor (EC 3.1.6.14) (G6S), (Glucosamine-6-sulfatase).]	Go Component not found
P16035	[Metalloproteinase inhibitor 2 precursor (TIMP-2) (Tissue inhibitor of, metalloproteinases 2) (CSC-21K).]	Go Component not found
P16152	[Carbonyl reductase [NADPH] 1 (EC 1.1.1.184) (NADPH-dependent carbonyl, reductase 1) (Prostaglandin-E(2) 9-reductase) (EC 1.1.1.189), (Prostaglandin 9-ketoreductase) (15-hydroxyprostaglandin dehydrogenase, [NADP+]) (EC 1.1.1.197).]	Go Component not found
P16591	[Proto-oncogene tyrosine-protein kinase FER (EC 2.7.10.2) (p94-FER) (c-, FER) (Tyrosine kinase 3).]	Go Component not found
P17024	Zinc finger protein 20 (Zinc finger protein KOX13).	Go Component not found
P17030	Zinc finger protein 25 (Zinc finger protein KOX19).	Go Component not found
P17039	Zinc finger protein 30 (Zinc finger protein KOX28).	Go Component not found
P17066	Heat shock 70 kDa protein 6 (Heat shock 70 kDa protein B').	Go Component not found
P17735	[Tyrosine aminotransferase (EC 2.6.1.5) (L-tyrosine:2-oxoglutarate, aminotransferase) (TAT).]	Go Component not found
P18084	Integrin beta-5 precursor.	Go Component not found
P18621	60S ribosomal protein L17 (L23).	Go Component not found

P19022	[Cadherin-2 precursor (Neural cadherin) (N-cadherin) (CD325 antigen), (CDw325).]	Go Component not found
P19113	Histidine decarboxylase (EC 4.1.1.22) (HDC).	Go Component not found
P19320	[Vascular cell adhesion protein 1 precursor (V-CAM 1) (CD106 antigen), (INCAM-100).]	Go Component not found
P19532	Transcription factor E3.	Go Component not found
P20155	[Serine protease inhibitor Kazal-type 2 precursor (Acrosin-trypsin, inhibitor) (HUSI-II).]	Go Component not found
P20290	Transcription factor BTF3 (RNA polymerase B transcription factor 3).	Go Component not found
P20333	[Tumor necrosis factor receptor superfamily member 1B precursor (Tumor, necrosis factor receptor 2) (TNF-R2) (Tumor necrosis factor receptor, type II) (p75) (p80 TNF-alpha receptor) (CD120b antigen) (Etanercept), [Contains: Tumor necrosis factor receptor superfamily member 1b,, membrane form; Tumor necrosis factor-binding protein 2 (TBPII) (TBP-, 2)].]	Go Component not found
P20794	[Serine/threonine-protein kinase MAK (EC 2.7.11.22) (Male germ cell-, associated kinase).]	Go Component not found
P20823	[Hepatocyte nuclear factor 1-alpha (HNF-1A) (Liver-specific, transcription factor LF-B1) (LFB1) (Transcription factor 1) (TCF-1).]	Go Component not found
P21108	[Ribose-phosphate pyrophosphokinase 3 (EC 2.7.6.1) (Phosphoribosyl, pyrophosphate synthetase III) (PRS-III) (Phosphoribosyl pyrophosphate, synthetase 1-like 1).]	Go Component not found
P21266	[Glutathione S-transferase Mu 3 (EC 2.5.1.18) (GSTM3-3) (GST class-mu, 3) (hGSTM3-3).]	Go Component not found
P42336	[Phosphatidylinositol-4,5-bisphosphate 3-kinase catalytic subunit alpha, isoform (EC 2.7.1.153) (PI3-kinase p110 subunit alpha) (PtdIns-3-, kinase p110) (PI3K).]	Go Component not found
P42681	Tyrosine-protein kinase TXK (EC 2.7.10.2).	Go Component not found
P42898	Methylenetetrahydrofolate reductase (EC 1.5.1.20).	Go Component not found
P43166	[Carbonic anhydrase 7 (EC 4.2.1.1) (Carbonic anhydrase VII) (Carbonate, dehydratase VII) (CA-VII).]	Go Component not found
P43359	[Melanoma-associated antigen 5 (MAGE-5 antigen) (Cancer/testis antigen, 1.5) (CT1.5).]	Go Component not found
P43365	[Melanoma-associated antigen 12 (MAGE-12 antigen) (MAGE12F), (Cancer/testis antigen 1.12) (CT1.12).]	Go Component not found
P45378	[Troponin T, fast skeletal muscle (TnTf) (Fast skeletal muscle troponin, T) (fTnT) (Beta TnTF).]	Go Component not found
P45984	[Mitogen-activated protein kinase 9 (EC 2.7.11.24) (Stress-activated, protein kinase JNK2) (c-Jun N-terminal kinase 2) (JNK-55).]	Go Component not found
P47897	[Glutaminyl-tRNA synthetase (EC 6.1.1.18) (Glutamine--tRNA ligase), (GlnRS).]	Go Component not found
P48059	[LIM and senescent cell antigen-like-containing domain protein 1, (Particularly interesting new Cys-His protein 1) (PINCH-1) (Renal, carcinoma antigen NY-REN-48).]	Go Component not found
P48443	Retinoic acid receptor RXR-gamma (Retinoid X receptor gamma).	Go Component not found
P48595	Serpin B10 (Bomapin) (Proteinase inhibitor 10).	Go Component not found
P48643	T-complex protein 1 subunit epsilon (TCP-1-epsilon) (CCT-epsilon).	Go Component not found
P48740	[Complement-activating component of Ra-reactive factor precursor, (EC 3.4.21.-) (Ra-reactive factor serine protease p100) (RaRF), (Mannan-binding lectin serine protease 1) (Mannose-binding protein-, associated serine protease) (MASP-1) (Serine protease 5) [Contains:, Complement-activating component of Ra-reactive factor heavy chain;, Complement-activating component of Ra-reactive factor light chain].]	Go Component not found
P49327	[Fatty acid synthase (EC 2.3.1.85) [Includes: [Acyl-carrier-protein] S-, acetyltransferase (EC 2.3.1.38); [Acyl-carrier-protein] S-, malonyltransferase (EC 2.3.1.39); 3-oxoacyl-[acyl-carrier-protein], synthase (EC 2.3.1.41); 3-oxoacyl-[acyl-carrier-protein] reductase, (EC 1.1.1.100); 3-hydroxypalmitoyl-[acyl-carrier-protein] dehydratase, (EC 4.2.1.61); Enoyl-[acyl-carrier-protein] reductase (EC 1.3.1.10);, Oleoyl-[acyl-carrier-protein] hydrolase (EC 3.1.2.14)].]	Go Component not found
P49619	[Diacylglycerol kinase gamma (EC 2.7.1.107) (Diglyceride kinase gamma), (DGK-gamma) (DAG kinase gamma).]	Go Component not found
P49756	[Probable RNA-binding protein 25 (RNA-binding motif protein 25) (RNA-, binding region-containing protein 7) (Protein S164).]	Go Component not found

P49759	[Dual specificity protein kinase CLK1 (EC 2.7.12.1) (CDC-like kinase, 1).]	Go Component not found
P49767	[Vascular endothelial growth factor C precursor (VEGF-C) (Vascular, endothelial growth factor-related protein) (VRP) (Flt4 ligand) (Flt4-, L).]	Go Component not found
P49802	Regulator of G-protein signaling 7 (RGS7).	Go Component not found
P49863	[Granzyme K precursor (EC 3.4.21.-) (Granzyme-3) (NK-tryptase-2) (NK-, TRYP-2) (Fragmentin-3).]	Go Component not found
P49915	[GMP synthase [glutamine-hydrolyzing] (EC 6.3.5.2) (Glutamine, amidotransferase) (GMP synthetase).]	Go Component not found
P50135	Histamine N-methyltransferase (EC 2.1.1.8) (HMT).	Go Component not found
P50458	[LIM/homeobox protein Lhx2 (LIM homeobox protein 2) (Homeobox protein, LH-2).]	Go Component not found
P50749	Ras association domain-containing protein 2.	Go Component not found
P51160	[Cone cGMP-specific 3',5'-cyclic phosphodiesterase subunit alpha', (EC 3.1.4.35) (cGMP phosphodiesterase 6C).]	Go Component not found
P51172	[Amiloride-sensitive sodium channel subunit delta (Epithelial Na(+), channel subunit delta) (Delta-ENaC) (Nonvoltage-gated sodium channel 1, subunit delta) (SCNED) (Delta-NaCH).]	Go Component not found
P51452	[Dual specificity protein phosphatase 3 (EC 3.1.3.48) (EC 3.1.3.16), (Dual specificity protein phosphatase VHR).]	Go Component not found
P51513	[RNA-binding protein Nova-1 (Neuro-oncological ventral antigen 1), (Onconeural ventral antigen 1) (Paraneoplastic Ri antigen) (Ventral, neuron-specific protein 1).]	Go Component not found
P51784	[Ubiquitin carboxyl-terminal hydrolase 11 (EC 3.1.2.15) (Ubiquitin, thioesterase 11) (Ubiquitin-specific-processing protease 11), (Deubiquitinating enzyme 11).]	Go Component not found
P51812	[Ribosomal protein S6 kinase alpha-3 (EC 2.7.11.1) (S6K-alpha 3) (90, kDa ribosomal protein S6 kinase 3) (p90-RSK 3) (Ribosomal S6 kinase 2), (RSK-2) (pp90RSK2) (Insulin-stimulated protein kinase 1) (ISPK-1) (MAP, kinase-activated protein kinase 1b) (MAPKAPK1B).]	Go Component not found
P51825	[AF4/FMR2 family member 1 (Protein AF-4) (Proto-oncogene AF4) (Protein, FEL).]	Go Component not found
P51957	[Serine/threonine-protein kinase Nek4 (EC 2.7.11.1) (NimA-related, protein kinase 4) (Serine/threonine-protein kinase 2), (Serine/threonine-protein kinase NRK2).]	Go Component not found
P52209	6-phosphogluconate dehydrogenase, decarboxylating (EC 1.1.1.44).	Go Component not found
P52333	[Tyrosine-protein kinase JAK3 (EC 2.7.10.2) (Janus kinase 3) (JAK-3), (Leukocyte janus kinase) (L-JAK).]	Go Component not found
P52630	Signal transducer and activator of transcription 2 (p113).	Go Component not found
P52735	Protein vav-2.	Go Component not found
P52757	Beta-chimaerin (Beta-chimerin) (Rho GTPase-activating protein 3).	Go Component not found
P52823	Stanniocalcin-1 precursor (STC-1).	Go Component not found
P52888	Thimet oligopeptidase (EC 3.4.24.15) (Endopeptidase 24.15) (MP78).	Go Component not found
P52954	Transcription factor LBX1 (Ladybird homeobox protein homolog 1).	Go Component not found
P53370	[Nucleoside diphosphate-linked moiety X motif 6 (Nudix motif 6), (Protein GFG) (GFG-1) (Antisense basic fibroblast growth factor).]	Go Component not found
P53675	Clathrin heavy chain 2 (CLH-22).	Go Component not found
P53779	[Mitogen-activated protein kinase 10 (EC 2.7.11.24) (Stress-activated, protein kinase JNK3) (c-Jun N-terminal kinase 3) (MAP kinase p49, 3F12).]	Go Component not found
P54646	[5'-AMP-activated protein kinase catalytic subunit alpha-2, (EC 2.7.11.1) (AMPK alpha-2 chain).]	Go Component not found
P54886	[Delta-1-pyrroline-5-carboxylate synthetase (P5CS) (Aldehyde, dehydrogenase family 18 member A1) [Includes: Glutamate 5-kinase, (EC 2.7.2.11) (Gamma-glutamyl kinase) (GK); Gamma-glutamyl phosphate, reductase (GPR) (EC 1.2.1.41) (Glutamate-5-semialdehyde dehydrogenase), (Glutamyl-gamma-semialdehyde dehydrogenase)].]	Go Component not found

P55042	GTP-binding protein RAD (RAS associated with diabetes) (RAD1).	Go Component not found
P55075	[Fibroblast growth factor 8 precursor (FGF-8) (HBGF-8) (Androgen-, induced growth factor) (AIGF).]	Go Component not found
P55198	Protein AF-17.	Go Component not found
P55286	Cadherin-8 precursor.	Go Component not found
P55316	[Forkhead box protein G1 (Forkhead box protein G1A) (Forkhead box, protein G1B) (Forkhead box protein G1C) (Forkhead-related protein, FKHL1) (HFK1) (Forkhead-related protein FKHL2) (HFK2) (Forkhead-, related protein FKHL3) (HFK3) (Transcription factor BF-1) (Brain, factor 1) (BF1) (Transcription factor BF-2) (Brain factor 2) (BF2), (hBF-2).]	Go Component not found
P55347	[Homeobox protein PKNOX1 (PBX/knotted homeobox 1) (Homeobox protein, PREP-1).]	Go Component not found
P56270	[Myc-associated zinc finger protein (MAZI) (Purine-binding, transcription factor) (Pur-1) (ZF87) (ZIF87).]	Go Component not found
P56645	[Period circadian protein homolog 3 (Circadian clock protein PERIOD 3), (hPER3) (Cell growth-inhibiting gene 13 protein).]	Go Component not found
P56846	T-cell leukemia/lymphoma protein 6 (Protein TNG1).	Go Component not found
P56962	Syntaxin-17 (KAT01814).	Go Component not found
P57055	Down syndrome critical region protein 6.	Go Component not found
P57078	[Receptor-interacting serine/threonine-protein kinase 4 (EC 2.7.11.1), (Ankyrin repeat domain-containing protein 3) (PKC-delta-interacting, protein kinase).]	Go Component not found
P57730	Caspase-1 inhibitor Iceberg.	Go Component not found
Q13442	[28 kDa heat- and acid-stable phosphoprotein (PDGF-associated protein), (PAP) (PDGFA-associated protein 1) (PAP1).]	Go Component not found
Q13496	Myotubularin (EC 3.1.3.48).	Go Component not found
Q13523	[Serine/threonine-protein kinase PRP4 homolog (EC 2.7.11.1) (PRP4 pre-, mRNA-processing factor 4 homolog) (PRP4 kinase).]	Go Component not found
Q13555	[Calcium/calmodulin-dependent protein kinase type II gamma chain, (EC 2.7.11.17) (CaM-kinase II gamma chain) (CaM kinase II subunit, gamma) (CaMK-II subunit gamma).]	Go Component not found
Q13616	Cullin-1 (CUL-1).	Go Component not found
Q13619	Cullin-4A (CUL-4A).	Go Component not found
Q13887	[Krueppel-like factor 5 (Intestinal-enriched krueppel-like factor), (Colon krueppel-like factor) (Transcription factor BTEB2) (Basic, transcription element-binding protein 2) (BTE-binding protein 2) (GC-, box-binding protein 2).]	Go Component not found
Q14093	Cylicin-2 (Cylicin II) (Multiple-band polypeptide II).	Go Component not found
Q14137	Ribosome biogenesis protein BOP1 (Block of proliferation 1 protein).	Go Component not found
Q14146	Uncharacterized protein KIAA0133.	Go Component not found
Q14166	Tubulin--tyrosine ligase-like protein 12.	Go Component not found
Q14209	Transcription factor E2F2 (E2F-2).	Go Component not found
Q14432	[cGMP-inhibited 3',5'-cyclic phosphodiesterase A (EC 3.1.4.17) (Cyclic, GMP-inhibited phosphodiesterase A) (CGI-PDE A).]	Go Component not found
Q14457	[Beclin-1 (Coiled-coil myosin-like BCL2-interacting protein) (Protein, GT197).]	Go Component not found
Q14524	[Sodium channel protein type 5 subunit alpha (Sodium channel protein, type V subunit alpha) (Voltage-gated sodium channel subunit alpha, Nav1.5) (Sodium channel protein cardiac muscle subunit alpha) (HH1).]	Go Component not found
Q14576	[ELAV-like protein 3 (Hu-antigen C) (HuC) (Paraneoplastic cerebellar, degeneration-associated antigen) (Paraneoplastic limbic encephalitis, antigen 21).]	Go Component not found
Q14585	Zinc finger protein 345 (Zinc finger protein 10) (HZF10).	Go Component not found
Q14653	Interferon regulatory factor 3 (IRF-3).	Go Component not found

Q14687	Genetic suppressor element 1.	Go Component not found
Q14749	Glycine N-methyltransferase (EC 2.1.1.20).	Go Component not found
Q15027	[Centaurin-beta 1 (Cnt-b1) (ARFGAP with coiled coil, ANK repeat and PH, domain-1) (ACAP1).]	Go Component not found
Q15048	Leucine-rich repeat-containing protein 14.	Go Component not found
Q15113	[Procollagen C-endopeptidase enhancer 1 precursor (Procollagen COOH-, terminal proteinase enhancer 1) (Procollagen C-proteinase enhancer 1), (PCPE-1) (Type I procollagen COOH-terminal proteinase enhancer) (Type, 1 procollagen C-proteinase enhancer protein).]	Go Component not found
Q15119	[[Pyruvate dehydrogenase [lipoamide]] kinase isozyme 2, mitochondrial, precursor (EC 2.7.11.2) (Pyruvate dehydrogenase kinase isoform 2).]	Go Component not found
Q15147	[1-phosphatidylinositol-4,5-bisphosphate phosphodiesterase beta-4, (EC 3.1.4.11) (Phosphoinositide phospholipase C) (Phospholipase C-, beta-4) (PLC-beta-4).]	Go Component not found
Q15329	Transcription factor E2F5 (E2F-5).	Go Component not found
Q15431	[Synaptonemal complex protein 1 (SCP-1) (Cancer/testis antigen 8), (CT8).]	Go Component not found
Q15485	[Ficolin-2 precursor (Ficolin-B) (Ficolin-beta) (L-ficolin), (Collagen/fibrinogen domain-containing protein 2) (Serum lectin p35), (EBP-37) (Hucolin).]	Go Component not found
Q15527	Surfeit locus protein 2 (Surf-2).	Go Component not found
Q15569	[Dual specificity testis-specific protein kinase 1 (EC 2.7.12.1), (Testicular protein kinase 1).]	Go Component not found
Q15699	Cartilage homeoprotein 1 (CART-1).	Go Component not found
Q15717	ELAV-like protein 1 (Hu-antigen R) (HuR).	Go Component not found
Q15742	[NGFI-A-binding protein 2 (EGR-1-binding protein 2) (Melanoma-, associated delayed early response protein) (Protein MADER).]	Go Component not found
Q15750	[Mitogen-activated protein kinase kinase kinase 7-interacting protein 1, (TGF-beta-activated kinase 1-binding protein 1) (TAK1-binding protein, 1).]	Go Component not found
Q15773	Myeloid leukemia factor 2 (Myelodysplasia-myeloid leukemia factor 2).	Go Component not found
Q15784	[Neurogenic differentiation factor 2 (NeuroD2) (NeuroD-related factor), (NDRF).]	Go Component not found
Q16281	[Cyclic nucleotide-gated cation channel alpha 3 (CNG channel alpha 3), (CNG-3) (CNG3) (Cyclic nucleotide-gated channel alpha 3) (Cone, photoreceptor cGMP-gated channel subunit alpha).]	Go Component not found
Q16517	Neuronatin.	Go Component not found
Q16566	[Calcium/calmodulin-dependent protein kinase type IV (EC 2.7.11.17), (CAM kinase-GR) (CaMK IV).]	Go Component not found
Q16626	Male-enhanced antigen 1 (MEA-1).	Go Component not found
Q16650	T-brain-1 protein (T-box brain protein 1) (TBR-1) (TES-56).	Go Component not found
Q16696	Cytochrome P450 2A13 (EC 1.14.14.1) (CYPIIA13).	Go Component not found
Q16816	[Phosphorylase b kinase gamma catalytic chain, skeletal muscle isoform, (EC 2.7.11.19) (Phosphorylase kinase subunit gamma 1).]	Go Component not found
Q53GD3	[Choline transporter-like protein 4 (Solute carrier family 44 member, 4).]	Go Component not found
Q86UA6	RPA-interacting protein (hRIP).	Go Component not found
Q8IUE1	[Homeobox protein TGIF2LX (TGFB-induced factor 2-like protein, X-, linked) (TGF(beta)induced transcription factor 2-like protein) (TGIF-, like on the X).]	Go Component not found
Q8N6M3	Uncharacterized protein C20orf142 precursor.	Go Component not found
Q8WUD1	Ras-related protein Rab-2B.	Go Component not found
Q92186	[Alpha-2,8-sialyltransferase 8B (EC 2.4.99.-) (ST8Sia II), (Sialyltransferase X) (STX).]	Go Component not found

Q92499	[ATP-dependent RNA helicase DDX1 (EC 3.6.1.-) (DEAD box protein 1), (DEAD box protein retinoblastoma) (DBP-RB).]	Go Component not found
Q92537	Uncharacterized protein KIAA0247 precursor.	Go Component not found
Q92545	Transmembrane protein 131 (Protein RW1).	Go Component not found
Q92562	SAC domain-containing protein 3 (FIG4 homolog).	Go Component not found
Q92617	Uncharacterized protein KIAA0220.	Go Component not found
Q92622	Uncharacterized protein KIAA0226.	Go Component not found
Q92777	Synapsin-2 (Synapsin II).	Go Component not found
Q92785	[Zinc finger protein ubi-d4 (Requiem) (Apoptosis response zinc finger, protein) (D4, zinc and double PHD fingers family 2).]	Go Component not found
Q92804	[TATA-binding protein-associated factor 2N (RNA-binding protein 56), (TAFII68) (TAF(II)68).]	Go Component not found
Q92832	[Protein kinase C-binding protein NELL1 precursor (NEL-like protein 1), (Nel-related protein 1).]	Go Component not found
Q92844	[TRAF family member-associated NF-kappa-B activator (TRAF-interacting, protein) (I-TRAF).]	Go Component not found
Q92903	[Phosphatidate cytidylyltransferase 1 (EC 2.7.7.41) (CDP-diglyceride, synthetase 1) (CDP-diglyceride pyrophosphorylase 1) (CDP-, diacylglycerol synthase 1) (CDS 1) (CTP:phosphatidate, cytidylyltransferase 1) (CDP-DAG synthase 1) (CDP-DG synthetase 1).]	Go Component not found
Q92930	Ras-related protein Rab-8B.	Go Component not found
Q92935	[Exostosin-like 1 (EC 2.4.1.224) (Glucuronosyl-N-acetylglucosaminyl-, proteoglycan 4-alpha-N-acetylglucosaminyltransferase) (Exostosin-L), (Multiple exostosis-like protein).]	Go Component not found
O76061	[Stanniocalcin-2 precursor (STC-2) (Stanniocalcin-related protein), (STCRP) (STC-related protein).]	Go Component not found
O94804	[Serine/threonine-protein kinase 10 (EC 2.7.11.1) (Lymphocyte-oriented, kinase).]	Go Component not found
O94819	[Kelch repeat and BTB domain-containing protein 11 (Kelch domain-, containing protein 7B).]	Go Component not found
O94966	[Ubiquitin carboxyl-terminal hydrolase 19 (EC 3.1.2.15) (Ubiquitin, thioesterase 19) (Ubiquitin-specific-processing protease 19), (Deubiquitinating enzyme 19) (Zinc finger MYND domain-containing, protein 9).]	Go Component not found
O94991	[SLIT and NTRK-like protein 5 precursor (Leucine-rich repeat-containing, protein 11).]	Go Component not found
O95013	Olfactory receptor 4F3.	Go Component not found
O95050	[Indolethylamine N-methyltransferase (EC 2.1.1.49) (Aromatic alkylamine, N-methyltransferase) (Indolamine N-methyltransferase) (Arylamine N-, methyltransferase) (Amine N-methyltransferase).]	Go Component not found
O95236	[Apolipoprotein-L3 (Apolipoprotein L-III) (ApoL-III) (TNF-inducible, protein CG12-1) (CG12_1).]	Go Component not found
O95249	[Golgi SNAP receptor complex member 1 (28 kDa Golgi SNARE protein) (28, kDa cis-Golgi SNARE p28) (GOS-28).]	Go Component not found
O95336	6-phosphogluconolactonase (EC 3.1.1.31) (6PGL).	Go Component not found
O95382	[Mitogen-activated protein kinase kinase kinase 6 (EC 2.7.11.25), (Apoptosis signal-regulating kinase 2).]	Go Component not found
O95394	[Phosphoacetylglucosamine mutase (EC 5.4.2.3) (PAGM) (Acetylglucosamine, phosphomutase) (N-acetylglucosamine-phosphate mutase), (Phosphoglucomutase 3).]	Go Component not found
O95409	Zinc finger protein ZIC 2 (Zinc finger protein of the cerebellum 2).	Go Component not found
O95447	Uncharacterized protein C21orf13.	Go Component not found
O95613	Pericentrin (Pericentrin B) (Kendrin).	Go Component not found
O95661	[GTP-binding protein Di-Ras3 (Distinct subgroup of the Ras family, member 3) (Rho-related GTP-binding protein RhoI).]	Go Component not found
O95718	[Steroid hormone receptor ERR2 (Estrogen-related receptor, beta) (ERR-, beta) (Estrogen receptor-like 2) (ERR beta-2).]	Go Component not found
O95816	[BAG family molecular chaperone regulator 2 (Bcl-2-associated, athanogene 2) (BAG-2).]	Go Component not found
O95863	Zinc finger protein SNAI1 (Protein snail homolog) (Protein sna).	Go Component not found

O95905	SGT1 protein (hSGT1) (Ecdysoneless homolog) (Suppressor of GCR2).	Go Component not found
O95947	T-box transcription factor TBX6 (T-box protein 6).	Go Component not found
P25685	[DnaJ homolog subfamily B member 1 (Heat shock 40 kDa protein 1) (Heat, shock protein 40) (HSP40) (DnaJ protein homolog 1) (HDJ-1).]	Go Component not found
P00167	Cytochrome b5.	Go Component not found
P00374	Dihydrofolate reductase (EC 1.5.1.3).	Go Component not found
P00568	[Adenylate kinase isoenzyme 1 (EC 2.7.4.3) (ATP-AMP transphosphorylase), (AK1) (Myokinase).]	Go Component not found
P01034	[Cystatin-C precursor (Cystatin-3) (Neuroendocrine basic polypeptide), (Gamma-trace) (Post-gamma-globulin).]	Go Component not found
P01210	[Proenkephalin A precursor [Contains: Synenkephalin; Met-enkephalin, (Opioid growth factor) (OGF); Met-enkephalin-Arg-Gly-Leu; Leu-, enkephalin; Met-enkephalin-Arg-Phe].]	Go Component not found
P01282	[VIP peptides precursor [Contains: Intestinal peptide PHV-42;, Intestinal peptide PHM-27 (Peptide histidine methioninamide 27);, Vasoactive intestinal peptide (VIP) (Vasoactive intestinal, polypeptide)].]	Go Component not found
P01344	[Insulin-like growth factor II precursor (IGF-II) (Somatomedin-A), [Contains: Insulin-like growth factor II Ala-25 Del].]	Go Component not found
P01571	[Interferon alpha-17 precursor (Interferon alpha-I') (Interferon alpha-, T) (Interferon alpha-88).]	Go Component not found
P01834	Ig kappa chain C region.	Go Component not found
P01854	Ig epsilon chain C region.	Go Component not found
P01876	Ig alpha-1 chain C region.	Go Component not found
P01918	[HLA class II histocompatibility antigen, DQ(1) beta chain precursor, (DC-3 beta chain).]	Go Component not found
P02144	Myoglobin.	Go Component not found
P02788	[Lactotransferrin precursor (EC 3.4.21.-) (Lactoferrin), (Talalactoferrin alfa) [Contains: Kaliocin-1; Lactoferroxin A;, Lactoferroxin B; Lactoferroxin C].]	Go Component not found
P04080	Cystatin-B (Stefin-B) (Liver thiol proteinase inhibitor) (CPI-B).	Go Component not found
P04090	Prorelaxin H2 precursor [Contains: Relaxin B chain; Relaxin A chain].	Go Component not found
P05060	[Secretogranin-1 precursor (Secretogranin I) (SgI) (Chromogranin-B), (CgB) [Contains: GAWK peptide; CCB peptide].]	Go Component not found
P05108	[Cytochrome P450 11A1, mitochondrial precursor (EC 1.14.15.6) (CYPXIA1), (P450(scc)) (Cholesterol side-chain cleavage enzyme) (Cholesterol, desmolase).]	Go Component not found
P05129	Protein kinase C gamma type (EC 2.7.11.13) (PKC-gamma).	Go Component not found
P05156	[Complement factor I precursor (EC 3.4.21.45) (C3B/C4B inactivator), [Contains: Complement factor I heavy chain; Complement factor I light, chain].]	Go Component not found
P05423	[DNA-directed RNA polymerase III subunit RPC4 (RNA polymerase III, subunit C4) (DNA-directed RNA polymerase III subunit D) (DNA-directed, RNA polymerase III 47 kDa polypeptide) (RPC53) (Protein BN51).]	Go Component not found
P06702	[Protein S100-A9 (S100 calcium-binding protein A9) (Calgranulin-B), (Migration inhibitory factor-related protein 14) (MRP-14) (P14), (Leukocyte L1 complex heavy chain) (Calprotectin L1H subunit).]	Go Component not found
P06744	[Glucose-6-phosphate isomerase (EC 5.3.1.9) (GPI) (Phosphoglucose, isomerase) (PGI) (Phosphohexose isomerase) (PHI) (Neuroleukin) (NLK), (Sperm antigen 36) (SA-36).]	Go Component not found
P07332	Proto-oncogene tyrosine-protein kinase Fes/Fps (EC 2.7.10.2) (C-Fes).	Go Component not found
P07384	[Calpain-1 catalytic subunit (EC 3.4.22.52) (Calpain-1 large subunit), (Calcium-activated neutral proteinase 1) (CANP 1) (Calpain mu-type), (muCANP) (Micromolar-calpain).]	Go Component not found
P21980	[Protein-glutamine gamma-glutamyltransferase 2 (EC 2.3.2.13) (Tissue, transglutaminase) (TGase C) (TGC) (TG(C)) (Transglutaminase-2) (TGase-, H).]	Go Component not found

P22059	Oxysterol-binding protein 1.	Go Component not found
P22309	[UDP-glucuronosyltransferase 1-1 precursor (EC 2.4.1.17) (UDP-, glucuronosyltransferase 1A1) (UDPGT) (UGT1*1) (UGT1-01) (UGT1.1) (UGT-, 1A) (UGT1A) (Bilirubin-specific UDPGT isozyme 1) (HUG-BR1).]	Go Component not found
P22676	Calretinin (CR) (29 kDa calbindin).	Go Component not found
P22794	[EVI2A protein precursor (Ecotropic viral integration site 2A protein, homolog) (EVI-2A).]	Go Component not found
P22914	Beta-crystallin S (Gamma-crystallin S) (Gamma-S-crystallin).	Go Component not found
P23141	[Liver carboxylesterase 1 precursor (EC 3.1.1.1) (Acyl coenzyme, A:cholesterol acyltransferase) (ACAT) (Monocyte/macrophage serine, esterase) (HMSE) (Serine esterase 1) (Brain carboxylesterase hBr1), (Triacylglycerol hydrolase) (TGH) (Egasyn) (Retinyl ester hydrolase), (REH).]	Go Component not found
P23378	[Glycine dehydrogenase [decarboxylating], mitochondrial precursor, (EC 1.4.4.2) (Glycine decarboxylase) (Glycine cleavage system P-, protein).]	Go Component not found
P23458	Tyrosine-protein kinase JAK1 (EC 2.7.10.2) (Janus kinase 1) (JAK-1).	Go Component not found
P23508	Colorectal mutant cancer protein (Protein MCC).	Go Component not found
P23743	[Diacylglycerol kinase alpha (EC 2.7.1.107) (Diglyceride kinase alpha), (DGK-alpha) (DAG kinase alpha) (80 kDa diacylglycerol kinase).]	Go Component not found
P24723	Protein kinase C eta type (EC 2.7.11.13) (nPKC-eta) (PKC-L).	Go Component not found
P25092	[Heat-stable enterotoxin receptor precursor (EC 4.6.1.2) (GC-C), (Intestinal guanylate cyclase) (STA receptor) (hSTAR).]	Go Component not found
P25325	3-mercaptopyruvate sulfurtransferase (EC 2.8.1.2) (MST).	Go Component not found
P80294	[Metallothionein-1H (MT-1H) (Metallothionein-IH) (MT-IH), (Metallothionein-0) (MT-0).]	Go Component not found
P26196	[Probable ATP-dependent RNA helicase DDX6 (EC 3.6.1.-) (DEAD box, protein 6) (ATP-dependent RNA helicase p54) (Oncogene RCK).]	Go Component not found
P26367	Paired box protein Pax-6 (Oculorhombin) (Aniridia type II protein).	Go Component not found
P26436	Acrosomal protein SP-10 precursor (Acrosomal vesicle protein 1).	Go Component not found
P26441	Ciliary neurotrophic factor (CNTF).	Go Component not found
P26678	Cardiac phospholamban (PLB).	Go Component not found
P27487	[Dipeptidyl peptidase 4 (EC 3.4.14.5) (Dipeptidyl peptidase IV) (DPP, IV) (T-cell activation antigen CD26) (TP103) (Adenosine deaminase, complexing protein 2) (ADABP) [Contains: Dipeptidyl peptidase 4, membrane form (Dipeptidyl peptidase IV membrane form); Dipeptidyl, peptidase 4 soluble form (Dipeptidyl peptidase IV soluble form)].]	Go Component not found
P27930	[Interleukin-1 receptor type II precursor (IL-1R-2) (IL-1R-beta), (CD121b antigen) (CDw121b).]	Go Component not found
P28698	[Myeloid zinc finger 1 (MZF-1) (Zinc finger protein 42) (Zinc finger, and SCAN domain-containing protein 6).]	Go Component not found
P29122	[Proprotein convertase subtilisin/kexin type 6 precursor (EC 3.4.21.-), (Paired basic amino acid cleaving enzyme 4) (Subtilisin/kexin-like, protease PACE4) (Subtilisin-like proprotein convertase 4) (SPC4).]	Go Component not found
P29377	[Protein S100-G (S100 calcium-binding protein G) (Vitamin D-dependent, calcium-binding protein, intestinal) (CABP) (Calbindin-D9k).]	Go Component not found
P29508	Serpin B3 (Squamous cell carcinoma antigen 1) (SCCA-1) (Protein T4-A).	Go Component not found
P29622	[Kallistatin precursor (Serpin A4) (Kallikrein inhibitor) (Protease, inhibitor 4).]	Go Component not found
P29777	Guanine nucleotide-binding protein G(o) subunit alpha 2.	Go Component not found
P30307	[M-phase inducer phosphatase 3 (EC 3.1.3.48) (Dual specificity, phosphatase Cdc25C).]	Go Component not found
P30453	[HLA class I histocompatibility antigen, A-34 alpha chain precursor, (MHC class I antigen A*34) (Aw-34) (A-10).]	Go Component not found

P30485	[HLA class I histocompatibility antigen, B-47 alpha chain precursor, (MHC class I antigen B*47) (Bw-47).]	Go Component not found
P31153	[S-adenosylmethionine synthetase isoform type-2 (EC 2.5.1.6), (Methionine adenosyltransferase 2) (AdoMet synthetase 2) (Methionine, adenosyltransferase II) (MAT-II).]	Go Component not found
P31270	Homeobox protein Hox-A11 (Hox-1I).	Go Component not found
P31483	[Nucleolysin TIA-1 isoform p40 (RNA-binding protein TIA-1) (p40-TIA-1), [Contains: Nucleolysin TIA-1 isoform p15 (p15-TIA-1)].]	Go Component not found
P32455	[Interferon-induced guanylate-binding protein 1 (GTP-binding protein 1), (Guanine nucleotide-binding protein 1) (GBP-1) (HuGBP-1).]	Go Component not found
P32754	[4-hydroxyphenylpyruvate dioxygenase (EC 1.13.11.27) (4HPPD) (HPD), (HPPDase) (4-hydroxyphenylpyruvic acid oxidase).]	Go Component not found
P33121	[Long-chain-fatty-acid--CoA ligase 1 (EC 6.2.1.3) (Long-chain acyl-CoA, synthetase 1) (LACS 1) (Palmitoyl-CoA ligase 1) (Long-chain fatty, acid-CoA ligase 2) (Long-chain acyl-CoA synthetase 2) (LACS 2) (Acyl-, CoA synthetase 1) (ACS1) (Palmitoyl-CoA ligase 2).]	Go Component not found
P33260	Cytochrome P450 2C18 (EC 1.14.14.1) (CYPIIC18) (P450-6B/29C).	Go Component not found
P33552	Cyclin-dependent kinases regulatory subunit 2 (CKS-2).	Go Component not found
P35270	Sepiapterin reductase (EC 1.1.1.153) (SPR).	Go Component not found
P35453	Homeobox protein Hox-D13 (Hox-4I).	Go Component not found
P35557	[Glucokinase (EC 2.7.1.2) (Hexokinase-4) (Hexokinase type IV) (HK IV), (HK4) (Hexokinase-D).]	Go Component not found
P35611	Alpha-adducin (Erythrocyte adducin subunit alpha).	Go Component not found
P35680	[Hepatocyte nuclear factor 1-beta (HNF-1-beta) (HNF-1B) (Variant, hepatic nuclear factor 1) (VHNF1) (Homeoprotein LFB3) (Transcription, factor 2) (TCF-2).]	Go Component not found
P36508	Zinc finger protein 76.	Go Component not found
P36952	Serpin B5 precursor (Maspin) (Protease inhibitor 5).	Go Component not found
P36959	[GMP reductase 1 (EC 1.7.1.7) (Guanosine 5'-monophosphate, oxidoreductase 1) (Guanosine monophosphate reductase 1).]	Go Component not found
P40394	[Alcohol dehydrogenase class 4 mu/sigma chain (EC 1.1.1.1) (Alcohol, dehydrogenase class IV mu/sigma chain) (Retinol dehydrogenase), (Gastric alcohol dehydrogenase).]	Go Component not found
P40818	[Ubiquitin carboxyl-terminal hydrolase 8 (EC 3.1.2.15) (Ubiquitin, thioesterase 8) (Ubiquitin-specific-processing protease 8), (Deubiquitinating enzyme 8) (hUBPy).]	Go Component not found
P41002	G2/mitotic-specific cyclin-F.	Go Component not found
P41161	ETS translocation variant 5 (Ets-related protein ERM).	Go Component not found
P41226	Ubiquitin-activating enzyme E1 homolog (D8).	Go Component not found
P58304	[Visual system homeobox 2 (Homeobox protein CHX10) (Ceh-10 homeodomain-, containing homolog).]	Go Component not found
P60059	Protein transport protein SEC61 subunit gamma.	Go Component not found
P61019	Ras-related protein Rab-2A.	Go Component not found
P61105	Ras-related protein Rab-2A.	Go Component not found
P61619	[Protein transport protein Sec61 subunit alpha isoform 1 (Sec61 alpha-, 1).]	Go Component not found
P61920	[Hemoglobin subunit gamma-1 (Hemoglobin gamma-1 chain) (Gamma-1-globin), (Hemoglobin gamma-A chain).]	Go Component not found
P62253	[Ubiquitin-conjugating enzyme E2 G1 (EC 6.3.2.19) (Ubiquitin-protein, ligase G1) (Ubiquitin carrier protein G1) (E217K) (UBC7).]	Go Component not found
P62760	Visinin-like protein 1 (VILIP) (Hippocalcin-like protein 3) (HLP3).	Go Component not found
P62910	60S ribosomal protein L32.	Go Component not found

P62987	60S ribosomal protein L40 (CEP52).	Go Component not found
P63244	[Guanine nucleotide-binding protein subunit beta-2-like 1 (Guanine, nucleotide-binding protein subunit beta-like protein 12.3) (Receptor, of activated protein kinase C 1) (RACK1) (Receptor for activated C, kinase).]	Go Component not found
P78367	Homeobox protein Nkx-3.2 (Bagpipe homeobox protein homolog 1).	Go Component not found
P80108	[Phosphatidylinositol-glycan-specific phospholipase D precursor, (EC 3.1.4.50) (PI-G PLD) (Glycoprotein phospholipase D) (Glycosyl-, phosphatidylinositol-specific phospholipase D).]	Go Component not found
P80108	[Phosphatidylinositol-glycan-specific phospholipase D precursor, (EC 3.1.4.50) (PI-G PLD) (Glycoprotein phospholipase D) (Glycosyl-, phosphatidylinositol-specific phospholipase D).]	Go Component not found
P80108	[Phosphatidylinositol-glycan-specific phospholipase D precursor, (EC 3.1.4.50) (PI-G PLD) (Glycoprotein phospholipase D) (Glycosyl-, phosphatidylinositol-specific phospholipase D).]	Go Component not found
P80108	[Phosphatidylinositol-glycan-specific phospholipase D precursor, (EC 3.1.4.50) (PI-G PLD) (Glycoprotein phospholipase D) (Glycosyl-, phosphatidylinositol-specific phospholipase D).]	Go Component not found
P80108	[Phosphatidylinositol-glycan-specific phospholipase D precursor, (EC 3.1.4.50) (PI-G PLD) (Glycoprotein phospholipase D) (Glycosyl-, phosphatidylinositol-specific phospholipase D).]	Go Component not found
P80108	[Phosphatidylinositol-glycan-specific phospholipase D precursor, (EC 3.1.4.50) (PI-G PLD) (Glycoprotein phospholipase D) (Glycosyl-, phosphatidylinositol-specific phospholipase D).]	Go Component not found
P80108	[Phosphatidylinositol-glycan-specific phospholipase D precursor, (EC 3.1.4.50) (PI-G PLD) (Glycoprotein phospholipase D) (Glycosyl-, phosphatidylinositol-specific phospholipase D).]	Go Component not found
P80108	[Phosphatidylinositol-glycan-specific phospholipase D precursor, (EC 3.1.4.50) (PI-G PLD) (Glycoprotein phospholipase D) (Glycosyl-, phosphatidylinositol-specific phospholipase D).]	Go Component not found
Q9BY66	[Histone demethylase JARID1D (EC 1.14.11.-) (Jumonji/ARID domain-, containing protein 1D) (Protein SmcY) (Histocompatibility Y antigen), (H-Y).]	Go Component not found
Q9C004	Portein sprouty homolog 4 (Spry-4).	Go Component not found
P82094	TATA element modulatory factor (TMF).	Go Component not found
P84095	Rho-related GTP-binding protein RhoG precursor.	Go Component not found
P98187	Cytochrome P450 4F8 (EC 1.14.14.1) (CYPIVF8).	Go Component not found
P98198	[Probable phospholipid-transporting ATPase ID (EC 3.6.3.1) (ATPase, class I type 8B member 2).]	Go Component not found
Q00266	[S-adenosylmethionine synthetase isoform type-1 (EC 2.5.1.6), (Methionine adenosyltransferase 1) (AdoMet synthetase 1) (Methionine, adenosyltransferase I/III) (MAT-I/III).]	Go Component not found
Q00532	[Cyclin-dependent kinase-like 1 (EC 2.7.11.22) (Serine/threonine-, protein kinase KKIALRE) (Protein kinase p42 KKIALRE).]	Go Component not found
Q00537	[Serine/threonine-protein kinase PCTAIRE-2 (EC 2.7.11.22) (PCTAIRE-, motif protein kinase 2).]	Go Component not found
Q00613	[Heat shock factor protein 1 (HSF 1) (Heat shock transcription factor, 1) (HSTF 1).]	Go Component not found
Q00796	Sorbitol dehydrogenase (EC 1.1.1.14) (L-iditol 2-dehydrogenase).	Go Component not found
Q01101	Insulinoma-associated protein 1 (Zinc finger protein IA-1).	Go Component not found
Q01484	Ankyrin-2 (Brain ankyrin) (Ankyrin-B) (Non-erythroid ankyrin).	Go Component not found
Q01534	Testis-specific Y-encoded protein 1 (Cancer/testis antigen 78) (CT78).	Go Component not found
Q01831	[DNA-repair protein complementing XP-C cells (Xeroderma pigmentosum, group C-complementing protein) (p125).]	Go Component not found
Q01970	[1-phosphatidylinositol-4,5-bisphosphate phosphodiesterase beta-3, (EC 3.1.4.11) (Phosphoinositide phospholipase C) (Phospholipase C-, beta-3) (PLC-beta-3).]	Go Component not found
Q02086	Transcription factor Sp2.	Go Component not found

Q02386	[Zinc finger protein 45 (BRC1744) (Zinc finger protein 13) (Zinc finger, protein KOX5).]	Go Component not found
Q02509	Otoconin 90 precursor (Oc90) (Phospholipase A2 homolog).	Go Component not found
Q02577	[Helix-loop-helix protein 2 (HEN2) (Nescient helix loop helix 2) (NSCL-, 2).]	Go Component not found
Q02779	[Mitogen-activated protein kinase kinase kinase 10 (EC 2.7.11.25), (Mixed lineage kinase 2) (Protein kinase MST).]	Go Component not found
Q02817	Mucin-2 precursor (Intestinal mucin-2).	Go Component not found
Q03014	[Homeobox protein PRH (Hematopoietically-expressed homeobox) (Homeobox, protein HEX).]	Go Component not found
Q03933	[Heat shock factor protein 2 (HSF 2) (Heat shock transcription factor, 2) (HSTF 2).]	Go Component not found
Q04864	C-Rel proto-oncogene protein (C-Rel protein).	Go Component not found
Q06210	[Glucosamine--fructose-6-phosphate aminotransferase [isomerizing] 1, (EC 2.6.1.16) (Glutamine:fructose 6 phosphate amidotransferase 1), (Hexosephosphate aminotransferase 1) (D-fructose-6-phosphate, amidotransferase 1) (GFAT 1) (GFAT1).]	Go Component not found
Q06413	Myocyte-specific enhancer factor 2C.	Go Component not found
Q06455	[Protein CBFA2T1 (Protein MTG8) (Protein ETO) (Eight twenty one, protein) (Cyclin-D-related protein) (Zinc finger MYND domain-, containing protein 2).]	Go Component not found
Q06643	[Lymphotoxin-beta (LT-beta) (Tumor necrosis factor C) (TNF-C) (Tumor, necrosis factor ligand superfamily member 3).]	Go Component not found
Q07000	[HLA class I histocompatibility antigen, Cw-15 alpha chain precursor, (MHC class I antigen Cw*15).]	Go Component not found
Q07444	[NKG2-E type II integral membrane protein (NKG2-E-activating NK, receptor) (NK cell receptor E).]	Go Component not found
Q07890	Son of sevenless homolog 2 (SOS-2).	Go Component not found
Q08050	[Forkhead box protein M1 (Forkhead-related protein FKHL16) (Hepatocyte, nuclear factor 3 forkhead homolog 11) (HNF-3/fork-head homolog 11), (HFH-11) (Winged-helix factor from INS-1 cells) (M-phase, phosphoprotein 2) (MPM-2 reactive phosphoprotein 2) (Transcription, factor Trident).]	Go Component not found
Q08257	[Quinone oxidoreductase (EC 1.6.5.5) (NADPH:quinone reductase) (Zeta-, crystallin).]	Go Component not found
Q08477	[Cytochrome P450 4F3 (EC 1.14.13.30) (CYPIVF3) (Leukotriene-B(4) omega-, hydroxylase) (Leukotriene-B(4) 20-monooxygenase) (Cytochrome P450-LTB-, omega).]	Go Component not found
Q08752	[40 kDa peptidyl-prolyl cis-trans isomerase (EC 5.2.1.8) (PPIase), (Rotamase) (Cyclophilin-40) (CYP-40) (Cyclophilin-related protein).]	Go Component not found
Q08999	[Retinoblastoma-like protein 2 (130 kDa retinoblastoma-associated, protein) (p130) (PRB2) (RBR-2).]	Go Component not found
Q09428	[ATP-binding cassette transporter sub-family C member 8 (Sulfonylurea, receptor 1).]	Go Component not found
Q12766	SMF protein (Fragment).	Go Component not found
Q12788	WD repeat-containing protein SAZD (Transducin beta-like 3 protein).	Go Component not found
Q12864	[Cadherin-17 precursor (Liver-intestine cadherin) (LI-cadherin), (Intestinal peptide-associated transporter HPT-1).]	Go Component not found
Q12889	[Oviduct-specific glycoprotein precursor (Oviductal glycoprotein), (Oviductin) (Estrogen-dependent oviduct protein) (Mucin-9).]	Go Component not found
Q12931	[Heat shock protein 75 kDa, mitochondrial precursor (HSP 75) (Tumor, necrosis factor type 1 receptor-associated protein) (TRAP-1) (TNFR-, associated protein 1).]	Go Component not found
Q12967	Ral guanine nucleotide dissociation stimulator (RalGEF) (RalGDS).	Go Component not found
Q13043	[Serine/threonine-protein kinase 4 (EC 2.7.11.1) (STE20-like kinase, MST1) (MST-1) (Mammalian STE20-like protein kinase 1), (Serine/threonine-protein kinase Krs-2).]	Go Component not found
Q13094	[Lymphocyte cytosolic protein 2 (SH2 domain-containing leukocyte, protein of 76 kDa) (SLP-76 tyrosine phosphoprotein) (SLP76).]	Go Component not found
Q13114	[TNF receptor-associated factor 3 (CD40 receptor-associated factor 1), (CRAF1) (CD40-binding protein) (CD40BP) (LMP1-associated protein), (LAP1) (CAP-1).]	Go Component not found

Q13162	[Peroxiredoxin-4 (EC 1.11.1.15) (Prx-IV) (Thioredoxin peroxidase AO372), (Thioredoxin-dependent peroxide reductase A0372) (Antioxidant enzyme, AOE372) (AOE37-2).]	Go Component not found
Q13188	[Serine/threonine-protein kinase 3 (EC 2.7.11.1) (STE20-like kinase, MST2) (MST-2) (Mammalian STE20-like protein kinase 2), (Serine/threonine-protein kinase Krs-1).]	Go Component not found
Q13203	Myosin-binding protein H (MyBP-H) (H-protein).	Go Component not found
Q13233	[Mitogen-activated protein kinase kinase kinase 1 (EC 2.7.11.25), (MAPK/ERK kinase kinase 1) (MEK kinase 1) (MEKK 1).]	Go Component not found
Q13247	[Splicing factor, arginine/serine-rich 6 (Pre-mRNA-splicing factor, SRP55).]	Go Component not found
Q13315	[Serine-protein kinase ATM (EC 2.7.11.1) (Ataxia telangiectasia, mutated) (A-T, mutated).]	Go Component not found
Q93034	[Cullin-5 (CUL-5) (Vasopressin-activated calcium-mobilizing receptor), (VACM-1).]	Go Component not found
Q93100	[Phosphorylase b kinase regulatory subunit beta (Phosphorylase kinase, subunit beta).]	Go Component not found
Q96MY1	Uncharacterized protein C20orf112.	Go Component not found
Q99453	[Paired mesoderm homeobox protein 2B (Paired-like homeobox 2B) (PHOX2B, homeodomain protein) (Neuroblastoma Phox) (NBPhox).]	Go Component not found
Q99504	Eyes absent homolog 3 (EC 3.1.3.48).	Go Component not found
Q99538	[Legumain precursor (EC 3.4.22.34) (Asparaginyl endopeptidase), (Protease, cysteine 1).]	Go Component not found
Q99558	[Mitogen-activated protein kinase kinase kinase 14 (EC 2.7.11.25) (NF-, kappa beta-inducing kinase) (Serine/threonine-protein kinase NIK), (HsNIK).]	Go Component not found
Q99584	Protein S100-A13 (S100 calcium-binding protein A13).	Go Component not found
Q99608	Necdin.	Go Component not found
Q99683	[Mitogen-activated protein kinase kinase kinase 5 (EC 2.7.11.25), (MAPK/ERK kinase kinase 5) (MEK kinase 5) (MEKK 5) (Apoptosis signal-, regulating kinase 1) (ASK-1).]	Go Component not found
Q99689	Fasciculation and elongation protein zeta 1 (Zygin-1) (Zygin I).	Go Component not found
Q99707	[Methionine synthase (EC 2.1.1.13) (5-methyltetrahydrofolate--, homocysteine methyltransferase) (Methionine synthase, vitamin-B12, dependent) (MS).]	Go Component not found
Q99909	Protein SSX3 (Cancer/testis antigen 5.3) (CT5.3).	Go Component not found
Q9BS92	Protein NipSnap3B (SNAP1).	Go Component not found
Q9BXT4	[Tudor domain-containing protein 1 (Cancer/testis antigen 41.1), (CT41.1).]	Go Component not found
Q9GZV8	PR domain zinc finger protein 14 (PR domain-containing protein 14).	Go Component not found
Q9H0N5	[Pterin-4-alpha-carbinolamine dehydratase 2 (EC 4.2.1.96) (PHS 2) (4-, alpha-hydroxy-tetrahydropterin dehydratase 2) (DcoH-like protein, DCoHm) (Dimerization cofactor of hepatocyte nuclear factor 1 from, muscle) (HNF-1-alpha dimerization cofactor).]	Go Component not found
Q9H169	Stathmin-4 (Stathmin-like protein B3) (RB3).	Go Component not found
Q9H4B4	[Serine/threonine-protein kinase PLK3 (EC 2.7.11.21) (Polo-like kinase, 3) (PLK-3) (Cytokine-inducible serine/threonine-protein kinase) (FGF-, inducible kinase) (Proliferation-related kinase).]	Go Component not found
Q9H4Q3	PR domain zinc finger protein 13 (PR domain-containing protein 13).	Go Component not found
Q9H9D4	Zinc finger protein 408 (PR domain zinc finger protein 17).	Go Component not found
Q9HAS0	Protein Njmu-R1.	Go Component not found
Q9HCH3	Copine-5 (Copine V).	Go Component not found
Q9HCS5	Band 4.1-like protein 4A (Protein NBL4).	Go Component not found
Q9NP87	DNA polymerase mu (EC 2.7.7.7) (Pol Mu).	Go Component not found

Q9NPR2	Semaphorin-4B precursor.	Go Component not found
Q9NQV6	PR domain zinc finger protein 10 (PR domain-containing protein 10).	Go Component not found
Q9NQX1	PR domain zinc finger protein 5 (PR domain-containing protein 5).	Go Component not found
Q9NR61	Delta-like protein 4 precursor (Drosophila Delta homolog 4).	Go Component not found
Q9NRI5	Disrupted in schizophrenia 1 protein.	Go Component not found
Q9NRZ5	[1-acyl-sn-glycerol-3-phosphate acyltransferase delta (EC 2.3.1.51) (1-, AGP acyltransferase 4) (1-AGPAT 4) (Lysophosphatidic acid, acyltransferase delta) (LPAAT-delta) (1-acylglycerol-3-phosphate O-, acyltransferase 4).]	Go Component not found
Q9NSI8	[SAM domain-containing protein SAMSN-1 (SAM domain, SH3 domain and, nuclear localization signals protein 1) (SH3-SAM adaptor protein), (Hematopoietic adaptor containing SH3 and SAM domains 1).]	Go Component not found
Q9NTN9	Semaphorin-4G precursor.	Go Component not found
Q9NVF9	[Ethanolamine kinase 2 (EC 2.7.1.82) (EKI 2) (Ethanolamine kinase-like, protein).]	Go Component not found
Q9NY12	[H/ACA ribonucleoprotein complex subunit 1 (Nucleolar protein family A, member 1) (snoRNP protein GAR1).]	Go Component not found
Q9NYI0	[PH and SEC7 domain-containing protein 3 (Pleckstrin homology and SEC7, domain-containing protein 3) (Exchange factor for ADP-ribosylation, factor guanine nucleotide factor 6) (Hepatocellular carcinoma-, associated antigen 67).]	Go Component not found
Q9NYP9	Uncharacterized protein C21orf45 (FAPP1-associated protein 1).	Go Component not found
Q9NYT6	Zinc finger protein 226.	Go Component not found
Q9NZ50	Gamma-synuclein (Synoretin) (SR).	Go Component not found
Q9NZJ4	Sacsin.	Go Component not found
Q9NZU6	Calcium-binding protein 3 (CaBP3).	Go Component not found
Q9P286	[Serine/threonine-protein kinase PAK 7 (EC 2.7.11.1) (p21-activated, kinase 7) (PAK-7) (PAK-5).]	Go Component not found
Q9P2J3	Kelch-like protein 9.	Go Component not found
Q9P2N7	Kelch-like protein 13 (BTB and kelch domain-containing protein 2).	Go Component not found
Q9P2S5	WD repeat-containing protein 8.	Go Component not found
Q9UBX7	[Kallikrein-11 precursor (EC 3.4.21.-) (hK11) (Hippostasin) (Trypsin-, like protease) (Serine protease 20) [Contains: Kallikrein-11 inactive, chain 1; Kallikrein-11 inactive chain 2].]	Go Component not found
Q9UDY4	[DnaJ homolog subfamily B member 4 (Heat shock 40 kDa protein 1, homolog) (Heat shock protein 40 homolog) (HSP40 homolog).]	Go Component not found
Q9UEU0	[Vesicle transport through interaction with t-SNAREs homolog 1B, (Vesicle transport v-SNARE protein Vti1-like 1) (Vti1-rp1).]	Go Component not found
Q9UGF7	Olfactory receptor 12D3 (Olfactory receptor OR6-27) (Hs6M1-27).	Go Component not found
Q9UGJ0	[5'-AMP-activated protein kinase subunit gamma-2 (AMPK gamma-2 chain), (AMPK gamma2) (H91620p).]	Go Component not found
Q9UH17	[Probable DNA dC->dU-editing enzyme APOBEC-3B (EC 3.5.4.-) (Phorbolin-, 1-related protein) (Phorbolin-2/3).]	Go Component not found
Q9UHL4	[Dipeptidyl-peptidase 2 precursor (EC 3.4.14.2) (Dipeptidyl-peptidase, II) (DPP II) (Dipeptidyl aminopeptidase II) (Quiescent cell proline, dipeptidase) (Dipeptidyl peptidase 7).]	Go Component not found
Q9UHP9	Small muscular protein (Stretch-responsive skeletal muscle protein).	Go Component not found
Q9UIA0	Cytohesin-4 (PH, SEC7 and coiled-coil domain-containing protein 4).	Go Component not found
Q9UIE0	Zinc finger protein 230 (Zinc finger protein FDZF2).	Go Component not found
Q9UIU6	Homeobox protein SIX4 (Sine oculis homeobox homolog 4).	Go Component not found
Q9UJ72	Annexin A10 (Annexin-10) (Annexin-14).	Go Component not found
Q9UJV9	[Probable ATP-dependent RNA helicase DDX41 (EC 3.6.1.-) (DEAD box, protein 41) (DEAD box protein abstrakt homolog).]	Go Component not found

Q9UK05	[Growth/differentiation factor 2 precursor (GDF-2) (Bone morphogenetic, protein 9) (BMP-9).]	Go Component not found
Q9UK12	Zinc finger protein 222.	Go Component not found
Q9UK32	[Ribosomal protein S6 kinase alpha-6 (EC 2.7.11.1) (S6K-alpha 6) (90, kDa ribosomal protein S6 kinase 6) (p90-RSK 6) (Ribosomal S6 kinase 4), (RSK-4) (pp90RSK4).]	Go Component not found
Q9UKJ3	G patch domain-containing protein 8.	Go Component not found
Q9UKP4	[ADAMTS-7 precursor (EC 3.4.24.-) (A disintegrin and metalloproteinase, with thrombospondin motifs 7) (ADAM-TS 7) (ADAM-TS7).]	Go Component not found
Q9ULC5	[Long-chain-fatty-acid--CoA ligase 5 (EC 6.2.1.3) (Long-chain acyl-CoA, synthetase 5) (LACS 5).]	Go Component not found
Q9ULI0	ATPase family AAA domain-containing protein 2B.	Go Component not found
Q9ULK2	Ataxin-7-like protein 1 (Fragment).	Go Component not found
Q9UM63	[Zinc finger protein PLAGL1 (Pleiomorphic adenoma-like protein 1), (Tumor supressor ZAC) (Lost on transformation 1) (LOT-1).]	Go Component not found
Q9UMQ6	[Calpain-11 (EC 3.4.22.-) (Calcium-activated neutral proteinase 11), (CANP 11).]	Go Component not found
Q9UMY4	Sorting nexin-12.	Go Component not found
Q9UNI6	[Dual specificity protein phosphatase 12 (EC 3.1.3.48) (EC 3.1.3.16), (Dual specificity tyrosine phosphatase YVH1).]	Go Component not found
Q9UNT1	Rab-like protein 2B.	Go Component not found
Q9UNY5	[Zinc finger protein 232 (Zinc finger and SCAN domain-containing, protein 11).]	Go Component not found
Q9UPE1	[Serine/threonine-protein kinase SRPK3 (EC 2.7.11.1) (Serine/arginine-, rich protein specific kinase 3) (Serine/threonine-protein kinase 23), (Muscle-specific serine kinase 1) (MSSK-1).]	Go Component not found
Q9UPU5	[Ubiquitin carboxyl-terminal hydrolase 24 (EC 3.1.2.15) (Ubiquitin, thioesterase 24) (Ubiquitin-specific-processing protease 24), (Deubiquitinating enzyme 24).]	Go Component not found
Q9UQ74	Pregnancy-specific beta-1-glycoprotein 8 precursor (PSBG-8).	Go Component not found
Q9Y252	RING finger protein 6 (RING-H2 protein).	Go Component not found
Q9Y295	[Developmentally-regulated GTP-binding protein 1 (DRG 1) (Protein, NEDD3) (Neural precursor cell expressed developmentally down-regulated, protein 3).]	Go Component not found
Q9Y2E5	[Epididymis-specific alpha-mannosidase precursor (EC 3.2.1.24), (Mannosidase alpha class 2B member 2).]	Go Component not found
Q9Y2G7	Zinc finger protein 30 homolog (Zfp-30) (Zinc finger protein 745).	Go Component not found
Q9Y2K6	[Ubiquitin carboxyl-terminal hydrolase 20 (EC 3.1.2.15) (Ubiquitin, thioesterase 20) (Ubiquitin-specific-processing protease 20), (Deubiquitinating enzyme 20).]	Go Component not found
Q9Y2P7	Zinc finger protein 256 (Bone marrow zinc finger 3) (BMZF-3).	Go Component not found
Q9Y2X7	[ARF GTPase-activating protein GIT1 (G protein-coupled receptor kinase-, interactor 1) (GRK-interacting protein 1) (Cool-associated and, tyrosine-phosphorylated protein 1) (Cat-1).]	Go Component not found
Q9Y2Z2	Protein MTO1 homolog, mitochondrial precursor.	Go Component not found
Q9Y376	Calcium-binding protein 39 (Protein Mo25).	Go Component not found
Q9Y2V3	[Retinal homeobox protein Rx (Retina and anterior neural fold homeobox, protein).]	Go Component not found
A2RU54	Homeobox protein HMX2 (Homeobox protein H6 family member 2).	Go Component not found
O00124	[UBX domain-containing protein 6 (Reproduction 8 protein) (Protein Rep-, 8).]	Go Component not found
O00142	Thymidine kinase 2, mitochondrial precursor (EC 2.7.1.21) (Mt-TK).	Go Component not found

O00187	[Mannan-binding lectin serine protease 2 precursor (EC 3.4.21.104), (Mannose-binding protein-associated serine protease 2) (MASP-2) (MBL-, associated serine protease 2) [Contains: Mannan-binding lectin serine, protease 2 A chain; Mannan-binding lectin serine protease 2 B chain].]	Go Component not found
O00198	[Activator of apoptosis harakiri (Neuronal death protein DP5) (BH3-, interacting domain-containing protein 3).]	Go Component not found
O00295	[Tubby-related protein 2 (Tubby-like protein 2) (Cancer/testis antigen, 65) (CT65).]	Go Component not found
O00327	[Aryl hydrocarbon receptor nuclear translocator-like protein 1 (Brain, and muscle ARNT-like 1) (Member of PAS protein 3) (Basic-helix-loop-, helix-PAS protein MOP3) (bHLH-PAS protein JAP3).]	Go Component not found
O00458	[Interferon-related developmental regulator 1 (Nerve growth factor-, inducible protein PC4).]	Go Component not found
O00506	[Serine/threonine-protein kinase 25 (EC 2.7.11.1) (Sterile 20/oxidant, stress-response kinase 1) (Ste20/oxidant stress response kinase 1), (SOK-1) (Ste20-like kinase).]	Go Component not found
O00754	[Lysosomal alpha-mannosidase precursor (EC 3.2.1.24) (Mannosidase,, alpha B) (Lysosomal acid alpha-mannosidase) (Laman) (Mannosidase alpha, class 2B member 1) [Contains: Lysosomal alpha-mannosidase A peptide;, Lysosomal alpha-mannosidase B peptide; Lysosomal alpha-mannosidase C, peptide; Lysosomal alpha-mannosidase D peptide; Lysosomal alpha-, mannosidase E peptide].]	Go Component not found
O00763	[Acetyl-CoA carboxylase 2 (EC 6.4.1.2) (ACC-beta) [Includes: Biotin, carboxylase (EC 6.3.4.14)].]	Go Component not found
O14581	Olfactory receptor 7A17.	Go Component not found
O14609	Testis-specific XK-related protein, Y-linked.	Go Component not found
O14646	[Chromodomain-helicase-DNA-binding protein 1 (EC 3.6.1.-) (ATP-, dependent helicase CHD1) (CHD-1).]	Go Component not found
O14683	Tumor protein p53-inducible protein 11 (p53-induced gene 11 protein).	Go Component not found
O14717	[tRNA (cytosine-5-)-methyltransferase (EC 2.1.1.29) (DNA (cytosine-5)-, methyltransferase-like protein 2) (Dnmt2) (DNA methyltransferase, homolog HsaIIP) (DNA MTase homolog HsaIIP) (M.HsaIIP) (PuMet).]	Go Component not found
O14737	[Programmed cell death protein 5 (Protein TFAR19) (TF-1 cell apoptosis-, related protein 19).]	Go Component not found
O14828	[Secretory carrier-associated membrane protein 3 (Secretory carrier, membrane protein 3).]	Go Component not found
O14879	[Interferon-induced protein with tetratricopeptide repeats 3 (IFIT-3), (IFIT-4) (Interferon-induced 60 kDa protein) (IFI-60K) (ISG-60), (CIG49) (Retinoic acid-induced gene G protein) (RIG-G).]	Go Component not found
O14896	Interferon regulatory factor 6 (IRF-6).	Go Component not found
O14957	[Ubiquinol-cytochrome c reductase complex 6.4 kDa protein (EC 1.10.2.2), (Complex III subunit XI).]	Go Component not found
O15015	Zinc finger protein 646.	Go Component not found
O15040	Uncharacterized protein KIAA0329/KIAA0297.	Go Component not found
O15055	[Period circadian protein homolog 2 (Circadian clock protein PERIOD 2), (hPER2).]	Go Component not found
O15091	Uncharacterized protein KIAA0391.	Go Component not found
O15127	[Secretory carrier-associated membrane protein 2 (Secretory carrier, membrane protein 2).]	Go Component not found
O15266	[Short stature homeobox protein (Short stature homeobox-containing, protein) (Pseudoautosomal homeobox-containing osteogenic protein).]	Go Component not found
O15273	Telethonin (Titin cap protein).	Go Component not found
O15350	[Tumor protein p73 (p53-like transcription factor) (p53-related, protein).]	Go Component not found
O15370	SOX-12 protein (SOX-22 protein).	Go Component not found
O15479	[Melanoma-associated antigen B2 (MAGE-B2 antigen) (DSS-AHC critical, interval MAGE superfamily 6) (DAM6) (MAGE XP-2) (Cancer/testis antigen, 3.2) (CT3.2).]	Go Component not found

O15528	[25-hydroxyvitamin D-1 alpha hydroxylase, mitochondrial precursor, (EC 1.14.13.13) (Cytochrome P450 subfamily XXVIIB polypeptide 1), (Cytochrome p450 27B1) (Calcidiol 1-monooxygenase) (25-OHD-1 alpha-, hydroxylase) (25-hydroxyvitamin D(3) 1-alpha-hydroxylase) (VD3 1A, hydroxylase) (P450C1 alpha) (P450VD1-alpha).]	Go Component not found
O15541	RING finger protein 113A (Zinc finger protein 183).	Go Component not found
O15550	[Ubiquitously transcribed X chromosome tetratricopeptide repeat protein, (Ubiquitously transcribed TPR protein on the X chromosome).]	Go Component not found
O43175	D-3-phosphoglycerate dehydrogenase (EC 1.1.1.95) (3-PGDH).	Go Component not found
O43186	Cone-rod homeobox protein.	Go Component not found
O43196	MutS protein homolog 5.	Go Component not found
O43248	Homeobox protein Hox-C11.	Go Component not found
O43295	[SLIT-ROBO Rho GTPase-activating protein 3 (srGAP3) (srGAP2) (WAVE-, associated Rac GTPase-activating protein) (WRP) (Mental disorder-, associated GAP) (Rho GTPase-activating protein 14).]	Go Component not found
O43306	[Adenylate cyclase type 6 (EC 4.6.1.1) (Adenylate cyclase type VI) (ATP, pyrophosphate-lyase 6) (Adenylyl cyclase 6) (Ca(2+)-inhibitable, adenylyl cyclase).]	Go Component not found
O43313	Uncharacterized protein KIAA0431.	Go Component not found
O43474	[Krueppel-like factor 4 (Epithelial zinc finger protein EZF) (Gut-, enriched krueppel-like factor).]	Go Component not found
O43541	[Mothers against decapentaplegic homolog 6 (SMAD 6) (Mothers against, DPP homolog 6) (Smad6) (hSMAD6).]	Go Component not found
O43592	Exportin-T (tRNA exportin) (Exportin(tRNA)).	Go Component not found
O43734	[Adapter protein CIKS (Connection to IKK and SAPK/JNK) (TRAF3-, interacting protein 2) (Nuclear factor NF-kappa-B activator 1) (ACT1).]	Go Component not found
O43795	Myosin-Ib (Myosin I alpha) (MMI-alpha) (MMIa) (MYH-1c).	Go Component not found
O43822	Uncharacterized protein C21orf2 (C21orf-HUMF09G8.5) (YF5/A2).	Go Component not found
O43909	[Exostosin-like 3 (EC 2.4.1.223) (Glucuronyl-galactosyl-proteoglycan 4-, alpha-N-acetylglucosaminyltransferase) (Putative tumor suppressor, protein EXTL3) (Multiple exostosis-like protein 3) (Hereditary, multiple exostoses gene isolog) (EXT-related protein 1).]	Go Component not found
O43921	[Ephrin-A2 precursor (EPH-related receptor tyrosine kinase ligand 6), (LERK-6) (HEK7-ligand) (HEK7-L).]	Go Component not found
O60232	Sjoegren syndrome/scleroderma autoantigen 1 (Autoantigen p27).	Go Component not found
O60299	Uncharacterized protein KIAA0552.	Go Component not found
O60361	[Putative nucleoside diphosphate kinase (EC 2.7.4.6) (NDK) (NDP, kinase).]	Go Component not found
O60658	[High affinity cAMP-specific and IBMX-insensitive 3',5'-cyclic, phosphodiesterase 8A (EC 3.1.4.17).]	Go Component not found
O60676	[Cystatin-8 precursor (Cystatin-related epididymal spermatogenic, protein).]	Go Component not found
O60733	[85 kDa calcium-independent phospholipase A2 (EC 3.1.1.4) (iPLA2) (CaI-, PLA2) (Group VI phospholipase A2) (GVI PLA2).]	Go Component not found
O60810	PRAME family member 4.	Go Component not found
O60841	[Eukaryotic translation initiation factor 5B (eIF-5B) (Translation, initiation factor IF-2).]	Go Component not found
O60941	Dystrobrevin beta (Beta-dystrobrevin) (DTN-B).	Go Component not found
O75185	[Calcium-transporting ATPase type 2C member 2 (EC 3.6.3.8) (ATPase, 2C2).]	Go Component not found
O75317	[Ubiquitin carboxyl-terminal hydrolase 12 (EC 3.1.2.15) (Ubiquitin, thioesterase 12) (Ubiquitin-specific-processing protease 12), (Deubiquitinating enzyme 12) (Ubiquitin-hydrolyzing enzyme 1).]	Go Component not found
O75330	[Hyaluronan mediated motility receptor (Intracellular hyaluronic acid-, binding protein) (Receptor for hyaluronan-mediated motility) (CD168, antigen).]	Go Component not found

O75363	[Breast carcinoma amplified sequence 1 (Novel amplified in breast, cancer 1) (Amplified and overexpressed in breast cancer).]	Go Component not found
O75376	Nuclear receptor corepressor 1 (N-CoR1) (N-CoR).	Go Component not found
O75437	[Zinc finger protein 254 (Bone marrow zinc finger 5) (BMZF-5), (Hematopoietic cell-derived zinc finger protein 1) (HD-ZNF1) (Zinc, finger protein 539) (Zinc finger protein 91-like).]	Go Component not found
O75493	[Carbonic anhydrase-related protein 11 precursor (CA-XI) (CARP XI) (CA-, RP XI) (Carbonic anhydrase-related protein 2) (CARP-2) (CA-RP II).]	Go Component not found
O75558	Syntaxin-11.	Go Component not found
O75601	[Caspase-13 precursor (EC 3.4.22.-) (CASP-13) (Evolutionary related, interleukin-1-beta-converting enzyme) (ERICE) [Contains: Caspase-13, subunit 1; Caspase-13 subunit 2].]	Go Component not found
O75616	[GTP-binding protein era homolog (hERA) (ERA-W) (Conserved ERA-like, GTPase) (CEGA).]	Go Component not found
O75695	Protein XRP2.	Go Component not found
O75844	[CAAX prenyl protease 1 homolog (EC 3.4.24.84) (Prenyl protein-specific, endoprotease 1) (Farnesylated proteins-converting enzyme 1) (FACE-1), (Zinc metalloproteinase Ste24 homolog).]	Go Component not found
O75891	[10-formyltetrahydrofolate dehydrogenase (EC 1.5.1.6) (10-FTHFDH), (Aldehyde dehydrogenase family 1 member L1).]	Go Component not found
O75909	Cyclin-K.	Go Component not found
P07902	[Galactose-1-phosphate uridylyltransferase (EC 2.7.7.12) (Gal-1-P, uridylyltransferase) (UDP-glucose--hexose-1-phosphate, uridylyltransferase).]	Go Component not found
P07947	[Proto-oncogene tyrosine-protein kinase Yes (EC 2.7.10.2) (p61-Yes) (c-, Yes).]	Go Component not found
P08134	Rho-related GTP-binding protein RhoC precursor (H9).	Go Component not found
P08631	[Tyrosine-protein kinase HCK (EC 2.7.10.2) (p59-HCK/p60-HCK), (Hemopoietic cell kinase).]	Go Component not found
P08684	[Cytochrome P450 3A4 (EC 1.14.13.67) (Quinine 3-monooxygenase), (CYPIIIA4) (Nifedipine oxidase) (Taurochenodeoxycholate 6-alpha-, hydroxylase) (EC 1.14.13.97) (NF-25) (P450-PCN1).]	Go Component not found
P08754	Guanine nucleotide-binding protein G(k) subunit alpha (G(i) alpha-3).	Go Component not found
P09488	[Glutathione S-transferase Mu 1 (EC 2.5.1.18) (GSTM1-1) (GST class-mu, 1) (GSTM1a-1a) (GSTM1b-1b) (HB subunit 4) (GTH4).]	Go Component not found
P09871	[Complement C1s subcomponent precursor (EC 3.4.21.42) (C1 esterase), [Contains: Complement C1s subcomponent heavy chain; Complement C1s, subcomponent light chain].]	Go Component not found
P09913	[Interferon-induced protein with tetratricopeptide repeats 2 (IFIT-2), (Interferon-induced 54 kDa protein) (IFI-54K) (ISG-54 K).]	Go Component not found
P09960	[Leukotriene A-4 hydrolase (EC 3.3.2.6) (LTA-4 hydrolase) (Leukotriene, A(4) hydrolase).]	Go Component not found
P0C0L5	[Complement C4-B precursor (Basic complement C4) [Contains: Complement, C4 beta chain; Complement C4-B alpha chain; C4a anaphylatoxin; C4b-B;, C4d-B; Complement C4 gamma chain].]	Go Component not found
P10244	Myb-related protein B (B-Myb).	Go Component not found
P10301	Ras-related protein R-Ras precursor (p23).	Go Component not found
P10635	[Cytochrome P450 2D6 (EC 1.14.14.1) (CYPIID6) (P450-DB1) (Debrisoquine, 4-hydroxylase).]	Go Component not found
P10721	[Mast/stem cell growth factor receptor precursor (EC 2.7.10.1) (SCFR), (Proto-oncogene tyrosine-protein kinase Kit) (c-kit) (CD117 antigen).]	Go Component not found
P10826	[Retinoic acid receptor beta (RAR-beta) (RAR-epsilon) (HBV-activated, protein).]	Go Component not found
P11473	Vitamin D3 receptor (VDR) (1,25-dihydroxyvitamin D3 receptor).	Go Component not found
P11498	[Pyruvate carboxylase, mitochondrial precursor (EC 6.4.1.1) (Pyruvic, carboxylase) (PCB).]	Go Component not found
P11686	[Pulmonary surfactant-associated protein C precursor (SP-C) (SP5), (Pulmonary surfactant-associated proteolipid SPL(Val)).]	Go Component not found

P11766	[Alcohol dehydrogenase class-3 (EC 1.1.1.1) (Alcohol dehydrogenase, class-III) (Alcohol dehydrogenase class chi chain) (S-, (hydroxymethyl)glutathione dehydrogenase) (EC 1.1.1.284) (Glutathione-, dependent formaldehyde dehydrogenase) (FDH).]	Go Component not found
P11844	Gamma-crystallin A (Gamma-A-crystallin) (Gamma-crystallin 5).	Go Component not found
P12104	Fatty acid-binding protein, intestinal (I-FABP) (FABPI).	Go Component not found
P12643	Bone morphogenetic protein 2 precursor (BMP-2) (BMP-2A).	Go Component not found
P12955	[Xaa-Pro dipeptidase (EC 3.4.13.9) (X-Pro dipeptidase) (Proline, dipeptidase) (Prolidase) (Imidodipeptidase).]	Go Component not found
P13056	Orphan nuclear receptor TR2 (Testicular receptor 2).	Go Component not found
P13349	Myogenic factor 5 (Myf-5).	Go Component not found
P13497	[Bone morphogenetic protein 1 precursor (EC 3.4.24.19) (BMP-1), (Procollagen C-proteinase) (PCP) (Mammalian tolloid protein) (mTld).]	Go Component not found
P13612	[Integrin alpha-4 precursor (Integrin alpha-IV) (VLA-4) (CD49d, antigen).]	Go Component not found
P13682	Zinc finger protein 35 (Zinc finger protein HF.10).	Go Component not found
P13798	[Acylamino-acid-releasing enzyme (EC 3.4.19.1) (AARE) (Acyl-peptide, hydrolase) (APH) (Acylaminoacyl-peptidase) (Oxidized protein, hydrolase) (OPH).]	Go Component not found
P13928	[Annexin A8 (Annexin-8) (Annexin VIII) (Vascular anticoagulant-beta), (VAC-beta).]	Go Component not found
P13984	[Transcription initiation factor IIF subunit beta (EC 3.6.1.-) (TFIIF-, beta) (ATP-dependent helicase GTF2F2) (General transcription factor, IIF subunit 2) (Transcription initiation factor RAP30).]	Go Component not found
P15309	Prostatic acid phosphatase precursor (EC 3.1.3.2).	Go Component not found
P15336	[Cyclic AMP-dependent transcription factor ATF-2 (Activating, transcription factor 2) (cAMP response element-binding protein CRE-, BP1) (HB16).]	Go Component not found
P15498	Proto-oncogene vav.	Go Component not found
P15538	[Cytochrome P450 11B1, mitochondrial precursor (EC 1.14.15.4) (CYPXIB1), (P450C11) (P-450c11) (Steroid 11-beta-hydroxylase).]	Go Component not found
P15621	[Zinc finger protein 44 (Zinc finger protein KOX7) (Gonadotropin-, inducible transcription repressor 2) (GIOT-2) (Zinc finger protein, 58).]	Go Component not found
P15882	[N-chimaerin (NC) (N-chimerin) (Alpha-chimerin) (A-chimaerin) (Rho, GTPase-activating protein 2).]	Go Component not found
P16050	[Arachidonate 15-lipoxygenase (EC 1.13.11.33) (Arachidonate omega-6, lipoxygenase) (15-LOX).]	Go Component not found
P16112	[Aggrecan core protein precursor (Cartilage-specific proteoglycan core, protein) (CSPCP) (Chondroitin sulfate proteoglycan core protein 1), [Contains: Aggrecan core protein 2].]	Go Component not found
P16885	[1-phosphatidylinositol-4,5-bisphosphate phosphodiesterase gamma-2, (EC 3.1.4.11) (Phosphoinositide phospholipase C) (PLC-gamma-2), (Phospholipase C-gamma-2) (PLC-IV).]	Go Component not found
P17020	Zinc finger protein 16 (Zinc finger protein KOX9).	Go Component not found
P17040	[Zinc finger and SCAN domain-containing protein 20 (Zinc finger protein, 31) (Zinc finger protein 360) (Zinc finger protein KOX29).]	Go Component not found
P17342	[Atrial natriuretic peptide clearance receptor precursor (ANP-C), (ANPRC) (NPR-C) (Atrial natriuretic peptide C-type receptor).]	Go Component not found
P17812	[CTP synthase 1 (EC 6.3.4.2) (UTP--ammonia ligase 1) (CTP synthetase, 1).]	Go Component not found
P18440	[Arylamine N-acetyltransferase 1 (EC 2.3.1.5) (Arylamide acetylase 1), (Monomorphic arylamine N-acetyltransferase) (MNAT) (N-, acetyltransferase type 1) (NAT-1).]	Go Component not found
P18846	[Cyclic AMP-dependent transcription factor ATF-1 (Activating, transcription factor 1) (TREB36 protein).]	Go Component not found
P20061	Transcobalamin-1 precursor (Transcobalamin I) (TCI) (TC I).	Go Component not found

P20292	[Arachidonate 5-lipoxygenase-activating protein (FLAP) (MK-886-binding, protein).]	Go Component not found
P20810	Calpastatin (Calpain inhibitor) (Sperm BS-17 component).	Go Component not found
P20839	[Inosine-5'-monophosphate dehydrogenase 1 (EC 1.1.1.205) (IMP, dehydrogenase 1) (IMPDH-I) (IMPD 1).]	Go Component not found
P20853	Cytochrome P450 2A7 (EC 1.14.14.1) (CYPIIA7) (P450-IIA4).	Go Component not found
P21281	[Vacuolar ATP synthase subunit B, brain isoform (EC 3.6.3.14) (V-ATPase, subunit B 2) (Vacuolar proton pump subunit B 2) (Endomembrane proton, pump 58 kDa subunit) (HO57).]	Go Component not found
P21397	[Amine oxidase [flavin-containing] A (EC 1.4.3.4) (Monoamine oxidase, type A) (MAO-A).]	Go Component not found
P42226	Signal transducer and activator of transcription 6 (IL-4 Stat).	Go Component not found
P42285	[Superkiller viralicidic activity 2-like 2 (EC 3.6.1.-) (ATP-dependent, helicase SKIV2L2).]	Go Component not found
P42338	[Phosphatidylinositol-4,5-bisphosphate 3-kinase catalytic subunit beta, isoform (EC 2.7.1.153) (PI3-kinase p110 subunit beta) (PtdIns-3-kinase, p110) (PI3K) (PI3Kbeta).]	Go Component not found
P42357	Histidine ammonia-lyase (EC 4.3.1.3) (Histidase).	Go Component not found
P42575	[Caspase-2 precursor (EC 3.4.22.55) (CASP-2) (ICH-1 protease) (NEDD2, protein) (Neural precursor cell expressed developmentally down-, regulated protein 2) (NEDD-2) [Contains: Caspase-2 subunit p18;, Caspase-2 subunit p13; Caspase-2 subunit p12].]	Go Component not found
P42679	[Megakaryocyte-associated tyrosine-protein kinase (EC 2.7.10.2), (Tyrosine-protein kinase CTK) (Protein kinase HYL) (Hematopoietic, consensus tyrosine-lacking kinase).]	Go Component not found
P42696	RNA-binding protein 34 (RNA-binding motif protein 34).	Go Component not found
P42772	[Cyclin-dependent kinase 4 inhibitor B (p14-INK4b) (p15-INK4b), (p15INK4B) (Multiple tumor suppressor 2) (MTS2).]	Go Component not found
P43146	[Netrin receptor DCC precursor (Tumor suppressor protein DCC), (Colorectal cancer suppressor).]	Go Component not found
P43304	[Glycerol-3-phosphate dehydrogenase, mitochondrial precursor, (EC 1.1.99.5) (GPD-M) (GPDH-M) (mtGPD).]	Go Component not found
P43353	Aldehyde dehydrogenase 3B1 (EC 1.2.1.5) (Aldehyde dehydrogenase 7).	Go Component not found
P43360	[Melanoma-associated antigen 6 (MAGE-6 antigen) (MAGE3B) (Cancer/testis, antigen 1.6) (CT1.6).]	Go Component not found
P43366	[Melanoma-associated antigen B1 (MAGE-B1 antigen) (MAGE-XP antigen), (DSS-AHC critical interval MAGE superfamily 10) (DAM10) (Cancer/testis, antigen 3.1) (CT3.1).]	Go Component not found
P43490	[Nicotinamide phosphoribosyltransferase (EC 2.4.2.12) (NAmPRTase), (Nampt) (Pre-B cell-enhancing factor) (Pre-B-cell colony-enhancing, factor 1) (Visfatin).]	Go Component not found
P45985	[Dual specificity mitogen-activated protein kinase kinase 4, (EC 2.7.12.2) (MAP kinase kinase 4) (JNK-activating kinase 1) (c-Jun, N-terminal kinase kinase 1) (JNKK) (SAPK/ERK kinase 1) (SEK1).]	Go Component not found
P46020	[Phosphorylase b kinase regulatory subunit alpha, skeletal muscle, isoform (Phosphorylase kinase alpha M subunit).]	Go Component not found
P46379	[Large proline-rich protein BAT3 (HLA-B-associated transcript 3), (Protein G3).]	Go Component not found
P46597	[Hydroxyindole O-methyltransferase (EC 2.1.1.4) (HIOMT), (Acetylserotonin O-methyltransferase) (ASMT).]	Go Component not found
P46926	[Glucosamine-6-phosphate isomerase (EC 3.5.99.6) (Glucosamine-6-, phosphate deaminase) (GNPDA) (GlcN6P deaminase) (Oscillin).]	Go Component not found
P46976	Glycogenin-1 (EC 2.4.1.186).	Go Component not found
P47736	Rap1 GTPase-activating protein 1 (Rap1GAP).	Go Component not found
P47874	Olfactory marker protein (Olfactory neuronal-specific protein).	Go Component not found
P47989	[Xanthine dehydrogenase/oxidase [Includes: Xanthine dehydrogenase, (EC 1.17.1.4) (XD); Xanthine oxidase (EC 1.17.3.2) (XO) (Xanthine, oxidoreductase)].]	Go Component not found

P48052	Carboxypeptidase A2 precursor (EC 3.4.17.15).	Go Component not found
P48506	[Glutamate--cysteine ligase catalytic subunit (EC 6.3.2.2) (Gamma-, glutamylcysteine synthetase) (Gamma-ECS) (GCS heavy chain).]	Go Component not found
P48634	Large proline-rich protein BAT2 (HLA-B-associated transcript 2).	Go Component not found
P48651	[Phosphatidylserine synthase 1 (EC 2.7.8.-) (PtdSer synthase 1) (PSS-1), (Serine-exchange enzyme I).]	Go Component not found
P48735	[Isocitrate dehydrogenase [NADP], mitochondrial precursor (EC 1.1.1.42), (Oxalosuccinate decarboxylase) (IDH) (NADP(+)-specific ICDH) (IDP), (ICD-M).]	Go Component not found
P48764	[Sodium/hydrogen exchanger 3 (Na(+)/H(+) exchanger 3) (NHE-3) (Solute, carrier family 9 member 3).]	Go Component not found
P49591	[Seryl-tRNA synthetase, cytoplasmic (EC 6.1.1.11) (Seryl-tRNA(Ser/Sec), synthetase) (Serine--tRNA ligase) (SerRS).]	Go Component not found
P49642	[DNA primase small subunit (EC 2.7.7.-) (DNA primase 49 kDa subunit), (p49).]	Go Component not found
P49703	[ADP-ribosylation factor-like protein 4D (ADP-ribosylation factor-like, protein 4L).]	Go Component not found
P49716	[CCAAT/enhancer-binding protein delta (C/EBP delta) (Nuclear factor NF-, IL6-beta) (NF-IL6-beta).]	Go Component not found
P49746	Thrombospondin-3 precursor.	Go Component not found
P49754	Vacuolar protein sorting-associated protein 41 homolog (S53).	Go Component not found
P49760	[Dual specificity protein kinase CLK2 (EC 2.7.12.1) (CDC-like kinase, 2).]	Go Component not found
P49888	[Estrogen sulfotransferase (EC 2.8.2.4) (Sulfotransferase, estrogen-, preferring) (EST-1).]	Go Component not found
P50479	[PDZ and LIM domain protein 4 (LIM protein RIL) (Reversion-induced LIM, protein).]	Go Component not found
P51168	[Amiloride-sensitive sodium channel subunit beta (Epithelial Na(+), channel subunit beta) (Beta-ENaC) (Nonvoltage-gated sodium channel 1, subunit beta) (SCNEB) (Beta-NaCH) (ENaCB).]	Go Component not found
P51589	[Cytochrome P450 2J2 (EC 1.14.14.1) (CYPIIJ2) (Arachidonic acid, epoxygenase).]	Go Component not found
P51692	Signal transducer and activator of transcription 5B.	Go Component not found
P51786	Zinc finger protein 157 (Zinc finger protein 22) (HZF22).	Go Component not found
P51813	[Cytoplasmic tyrosine-protein kinase BMX (EC 2.7.10.2) (Bone marrow, tyrosine kinase gene in chromosome X protein) (Epithelial and, endothelial tyrosine kinase) (ETK) (NTK38).]	Go Component not found
P51816	[AF4/FMR2 family member 2 (Fragile X mental retardation 2 protein), (Protein FMR-2) (FMR2P) (Protein Ox19) (Fragile X E mental retardation, syndrome protein).]	Go Component not found
P51861	Cerebellar degeneration-related antigen 1 (CDR34).	Go Component not found
P51959	Cyclin-G1 (Cyclin-G).	Go Component not found
P52429	[Diacylglycerol kinase epsilon (EC 2.7.1.107) (Diglyceride kinase, epsilon) (DGK-epsilon) (DAG kinase epsilon).]	Go Component not found
P52736	Zinc finger protein 133 (Zinc finger protein 150).	Go Component not found
P52746	Zinc finger protein 142 (HA4654).	Go Component not found
P52788	[Spermine synthase (EC 2.5.1.22) (Spermidine aminopropyltransferase), (SPMSY).]	Go Component not found
P52824	[Diacylglycerol kinase theta (EC 2.7.1.107) (Diglyceride kinase theta), (DGK-theta) (DAG kinase theta).]	Go Component not found
P52945	[Pancreas/duodenum homeobox protein 1 (PDX-1) (Insulin promoter factor, 1) (IPF-1) (Islet/duodenum homeobox-1) (IDX-1) (Somatostatin-, transactivating factor 1) (STF-1) (Insulin upstream factor 1) (IUF-1), (Glucose-sensitive factor) (GSF).]	Go Component not found
P53004	[Biliverdin reductase A precursor (EC 1.3.1.24) (Biliverdin-IX alpha-, reductase) (BVR A).]	Go Component not found
P53804	[Tetratricopeptide repeat protein 3 (TPR repeat protein 3) (TPR repeat, protein D) (RING finger protein 105).]	Go Component not found
P54793	Arylsulfatase F precursor (EC 3.1.6.-) (ASF).	Go Component not found

P54855	[UDP-glucuronosyltransferase 2B15 precursor (EC 2.4.1.17) (UDPGT), (UDPGTh-3) (HLUG4).]	Go Component not found
P54920	[Alpha-soluble NSF attachment protein (SNAP-alpha) (N-ethylmaleimide-, sensitive factor attachment protein, alpha).]	Go Component not found
P55039	Developmentally-regulated GTP-binding protein 2 (DRG 2).	Go Component not found
P55081	Microfibrillar-associated protein 1.	Go Component not found
P55107	[Bone morphogenetic protein 3b precursor (BMP-3b), (Growth/differentiation factor 10) (GDF-10) (Bone-inducing protein), (BIP).]	Go Component not found
P55199	[RNA polymerase II elongation factor ELL (Eleven-nineteen lysine-rich, leukemia protein).]	Go Component not found
P55290	[Cadherin-13 precursor (Truncated cadherin) (T-cadherin) (T-cad) (Heart, cadherin) (H-cadherin) (P105).]	Go Component not found
P55735	Protein SEC13 homolog (SEC13-related protein) (SEC13-like protein 1).	Go Component not found
P55809	[Succinyl-CoA:3-ketoacid-coenzyme A transferase 1, mitochondrial, precursor (EC 2.8.3.5) (Somatic-type succinyl CoA:3-oxoacid CoA-, transferase) (Scot-S).]	Go Component not found
P56277	[Protein p8 MTCP-1 (Mature T-cell proliferation-1 type A) (MTCP-1 type, A) (p8MTCP1).]	Go Component not found
P56378	6.8 kDa mitochondrial proteolipid.	Go Component not found
P56539	Caveolin-3 (M-caveolin).	Go Component not found
P56693	Transcription factor SOX-10.	Go Component not found
P56847	Protein TNG2.	Go Component not found
P57056	Putative uncharacterized protein C21orf32.	Go Component not found
P57073	Transcription factor SOX-8.	Go Component not found
P57082	T-box transcription factor TBX4 (T-box protein 4).	Go Component not found
P57679	Ellis-van Creveld syndrome protein (DWF-1).	Go Component not found
P57735	Ras-related protein Rab-25 (CATX-8).	Go Component not found
P57764	Gasdermin domain-containing protein 1.	Go Component not found
Q13449	Limbic system-associated membrane protein precursor (LSAMP).	Go Component not found
Q13562	Neurogenic differentiation factor 1 (NeuroD1) (NeuroD).	Go Component not found
Q13591	Semaphorin-5A precursor (Semaphorin F) (Sema F).	Go Component not found
Q13617	Cullin-2 (CUL-2).	Go Component not found
Q13620	Cullin-4B (CUL-4B).	Go Component not found
Q13683	[Integrin alpha-7 precursor [Contains: Integrin alpha-7 heavy chain;, Integrin alpha-7 light chain].]	Go Component not found
Q13751	[Laminin subunit beta-3 precursor (Laminin 5 beta 3) (Laminin B1k, chain) (Kalinin B1 chain).]	Go Component not found
Q13888	[TFIIH basal transcription factor complex p44 subunit (Basic, transcription factor 2 44 kDa subunit) (BTF2-p44) (General, transcription factor IIH polypeptide 2).]	Go Component not found
Q13976	[cGMP-dependent protein kinase 1, alpha isozyme (EC 2.7.11.12) (CGK 1, alpha) (cGKI-alpha).]	Go Component not found
Q14011	[Cold-inducible RNA-binding protein (Glycine-rich RNA-binding protein, CIRP) (A18 hnRNP).]	Go Component not found
Q14094	Cyclin-I.	Go Component not found
Q14141	Septin-6.	Go Component not found
Q14147	[Probable ATP-dependent RNA helicase DHX34 (EC 3.6.1.-) (DEAH box, protein 34).]	Go Component not found
Q14155	[Rho guanine nucleotide exchange factor 7 (PAK-interacting exchange, factor beta) (Beta-Pix) (COOL-1) (p85).]	Go Component not found
Q14186	[Transcription factor Dp-1 (E2F dimerization partner 1) (DRTF1-, polypeptide 1) (DRTF1).]	Go Component not found

Q14344	Guanine nucleotide-binding protein alpha-13 subunit (G alpha-13).	Go Component not found
Q14500	[ATP-sensitive inward rectifier potassium channel 12 (Potassium, channel, inwardly rectifying subfamily J member 12) (Inward rectifier, K(+) channel Kir2.2) (Kir2.2v) (IRK2).]	Go Component not found
Q14541	Hepatocyte nuclear factor 4-gamma (HNF-4-gamma).	Go Component not found
Q14582	[Max-interacting transcriptional repressor MAD4 (Max-associated protein, 4) (MAX dimerization protein 4).]	Go Component not found
Q14586	Zinc finger protein 267 (Zinc finger protein 2) (HZF2).	Go Component not found
Q14669	[Probable E3 ubiquitin-protein ligase TRIP12 (EC 6.3.2.-) (Thyroid, receptor-interacting protein 12) (TRIP-12).]	Go Component not found
Q14689	Disco-interacting protein 2 homolog A.	Go Component not found
Q14721	[Potassium voltage-gated channel subfamily B member 1 (Voltage-gated, potassium channel subunit Kv2.1) (h-DRK1).]	Go Component not found
Q15031	[Probable leucyl-tRNA synthetase, mitochondrial precursor (EC 6.1.1.4), (Leucine--tRNA ligase) (LeuRS).]	Go Component not found
Q15040	Josephin-1 (Josephin domain-containing 1).	Go Component not found
Q15050	Ribosome biogenesis regulatory protein homolog.	Go Component not found
Q15116	[Programmed cell death protein 1 precursor (Protein PD-1) (hPD-1), (CD279 antigen).]	Go Component not found
Q15131	[Cell division protein kinase 10 (EC 2.7.11.22) (Serine/threonine-, protein kinase PISSLRE).]	Go Component not found
Q15269	Periodic tryptophan protein 2 homolog.	Go Component not found
Q15319	[POU domain, class 4, transcription factor 3 (Brain-specific, homeobox/POU domain protein 3C) (Brn-3C).]	Go Component not found
Q15375	[Ephrin type-A receptor 7 precursor (EC 2.7.10.1) (Tyrosine-protein, kinase receptor EHK-3) (EPH homology kinase 3) (Receptor protein-, tyrosine kinase HEK11).]	Go Component not found
Q15475	Homeobox protein SIX1 (Sine oculis homeobox homolog 1).	Go Component not found
Q15561	[Transcriptional enhancer factor TEF-3 (TEA domain family member 4), (TEAD-4) (Transcription factor RTEF-1).]	Go Component not found
Q15744	CCAAT/enhancer-binding protein epsilon (C/EBP epsilon).	Go Component not found
Q15759	[Mitogen-activated protein kinase 11 (EC 2.7.11.24) (Mitogen-activated, protein kinase p38 beta) (MAP kinase p38 beta) (p38b) (p38-2) (Stress-, activated protein kinase 2).]	Go Component not found
Q15776	[Zinc finger protein 192 (LD5-1) (Zinc finger protein with KRAB and, SCAN domains 8).]	Go Component not found
Q15818	Neuronal pentraxin-1 precursor (NP1) (Neuronal pentraxin I) (NP-I).	Go Component not found
Q15884	Uncharacterized protein C9orf61 (Protein X123).	Go Component not found
Q16342	[Programmed cell death protein 2 (Zinc finger protein Rp-8) (Zinc, finger MYND domain-containing protein 7).]	Go Component not found
Q16513	[Serine/threonine-protein kinase N2 (EC 2.7.11.13) (Protein kinase C-, like 2) (Protein-kinase C-related kinase 2).]	Go Component not found
Q16587	Zinc finger protein 74 (hZNF7).	Go Component not found
Q16600	Zinc finger protein 239 (Zinc finger protein MOK-2) (HOK-2).	Go Component not found
Q16678	Cytochrome P450 1B1 (EC 1.14.14.1) (CYPIB1).	Go Component not found
Q16763	[Ubiquitin-conjugating enzyme E2 S (EC 6.3.2.19) (Ubiquitin-protein, ligase S) (Ubiquitin carrier protein S) (Ubiquitin-conjugating enzyme, E2-24 kDa) (E2-EPF5).]	Go Component not found
Q16881	[Thioredoxin reductase 1, cytoplasmic precursor (EC 1.8.1.9) (TR), (TR1).]	Go Component not found
Q6P597	Kinesin light chain 3 (kinesin light chain 2) (KLC2-like).	Go Component not found
Q92466	[DNA damage-binding protein 2 (Damage-specific DNA-binding protein 2), (DDB p48 subunit) (DDBb) (UV-damaged DNA-binding protein 2) (UV-DDB, 2).]	Go Component not found

Q92526	[T-complex protein 1 subunit zeta-2 (TCP-1-zeta-2) (CCT-zeta-2) (TCP-1-, zeta-like) (CCT-zeta-like) (Testis-specific Tcp20) (Testis-specific, protein TSA303).]	Go Component not found
Q92543	Sorting nexin-19.	Go Component not found
Q92569	[Phosphatidylinositol 3-kinase regulatory subunit gamma (PI3-kinase p85, subunit gamma) (PtdIns-3-kinase p85-gamma) (p55PIK).]	Go Component not found
Q92609	TBC1 domain family member 5.	Go Component not found
Q92618	Zinc finger protein 516.	Go Component not found
Q92664	Transcription factor IIIA (Factor A) (TFIIIA).	Go Component not found
Q92674	[Centromere protein I (CENP-I) (Interphase centromere complex protein, 19) (Follicle-stimulating hormone primary response protein) (FSH, primary response protein 1) (Leucine-rich primary response protein 1).]	Go Component not found
Q92781	11-cis retinol dehydrogenase (EC 1.1.1.105) (11-cis RDH).	Go Component not found
Q92786	Homeobox prospero-like protein PROX1 (PROX 1).	Go Component not found
Q92828	Coronin-2A (WD repeat-containing protein 2) (IR10).	Go Component not found
Q92833	Protein Jumonji (Jumonji/ARID domain-containing protein 2).	Go Component not found
Q92851	[Caspase-10 precursor (EC 3.4.22.63) (CASP-10) (ICE-like apoptotic, protease 4) (Apoptotic protease Mch-4) (FAS-associated death domain, protein interleukin-1B-converting enzyme 2) (FLICE2) [Contains:, Caspase-10 subunit p23/17; Caspase-10 subunit p12].]	Go Component not found
O75962	[Triple functional domain protein (EC 2.7.11.1) (PTPRF-interacting, protein).]	Go Component not found
O76039	[Cyclin-dependent kinase-like 5 (EC 2.7.11.22) (Serine/threonine-, protein kinase 9).]	Go Component not found
O76074	[cGMP-specific 3',5'-cyclic phosphodiesterase (EC 3.1.4.35) (CGB-PDE), (cGMP-binding cGMP-specific phosphodiesterase).]	Go Component not found
O94808	[Glucosamine--fructose-6-phosphate aminotransferase [isomerizing] 2, (EC 2.6.1.16) (Glutamine:fructose 6 phosphate amidotransferase 2), (Hexosephosphate aminotransferase 2) (D-fructose-6-phosphate, amidotransferase 2) (GFAT 2) (GFAT2).]	Go Component not found
O94822	Zinc finger protein 294 (RING finger protein 160).	Go Component not found
O94933	SLIT and NTRK-like protein 3 precursor.	Go Component not found
O94967	WD repeat-containing protein 47.	Go Component not found
O95006	[Olfactory receptor 2F2 (Olfactory receptor OR7-6) (Olfactory receptor, 7-1) (OR7-1).]	Go Component not found
O95047	Olfactory receptor 2A4 (Olfactory receptor OR6-37).	Go Component not found
O95104	[Splicing factor, arginine/serine-rich 15 (CTD-binding SR-like protein, RA4).]	Go Component not found
O95294	RasGAP-activating-like protein 1.	Go Component not found
O95340	[Bifunctional 3'-phosphoadenosine 5'-phosphosulfate synthetase 2 (PAPS, synthetase 2) (PAPSS 2) (Sulfurylase kinase 2) (SK2) (SK 2) [Includes:, Sulfate adenylyltransferase (EC 2.7.7.4) (Sulfate adenylate, transferase) (SAT) (ATP-sulfurylase); Adenylyl-sulfate kinase, (EC 2.7.1.25) (Adenylylsulfate 3'-phosphotransferase) (APS kinase), (Adenosine-5'-phosphosulfate 3'-phosphotransferase) (3'-, phosphoadenosine-5'-phosphosulfate synthetase)].]	Go Component not found
O95390	[Growth/differentiation factor 11 precursor (GDF-11) (Bone, morphogenetic protein 11).]	Go Component not found
O95416	Transcription factor SOX-14.	Go Component not found
O95450	[ADAMTS-2 precursor (EC 3.4.24.14) (A disintegrin and metalloproteinase, with thrombospondin motifs 2) (ADAM-TS 2) (ADAM-TS2) (Procollagen I/II, amino propeptide-processing enzyme) (Procollagen I N-proteinase) (PC, I-NP) (Procollagen N-endopeptidase) (pNPI).]	Go Component not found
O95498	[Vascular non-inflammatory molecule 2 precursor (Vanin-2), (Glycosylphosphatidyl inositol-anchored protein GPI-80) (Protein FOAP-, 4).]	Go Component not found

O95573	[Long-chain-fatty-acid--CoA ligase 3 (EC 6.2.1.3) (Long-chain acyl-CoA, synthetase 3) (LACS 3).]	Go Component not found
O95622	[Adenylate cyclase type 5 (EC 4.6.1.1) (Adenylate cyclase type V) (ATP, pyrophosphate-lyase 5) (Adenylyl cyclase 5).]	Go Component not found
O95741	Copine-6 (Copine VI) (Neuronal-copine) (N-copine).	Go Component not found
O95865	[N(G),N(G)-dimethylarginine dimethylaminohydrolase 2 (EC 3.5.3.18), (Dimethylargininase-2) (Dimethylarginine dimethylaminohydrolase 2), (DDAHII) (DDAH-2) (S-phase protein) (Protein G6a).]	Go Component not found
O95948	[One cut domain family member 2 (Transcription factor ONECUT-2) (OC-2), (Hepatocyte nuclear factor 6-beta) (HNF-6-beta).]	Go Component not found
P25686	[DnaJ homolog subfamily B member 2 (Heat shock 40 kDa protein 3) (DnaJ, protein homolog 1) (HSJ-1).]	Go Component not found
P00390	[Glutathione reductase, mitochondrial precursor (EC 1.8.1.7) (GR), (GRase).]	Go Component not found
P00739	Haptoglobin-related protein precursor.	Go Component not found
P00746	[Complement factor D precursor (EC 3.4.21.46) (C3 convertase activator), (Properdin factor D) (Adipsin).]	Go Component not found
P00750	[Tissue-type plasminogen activator precursor (EC 3.4.21.68) (tPA) (t-, PA) (t-plasminogen activator) (Alteplase) (Reteplase) [Contains:, Tissue-type plasminogen activator chain A; Tissue-type plasminogen, activator chain B].]	Go Component not found
P00813	Adenosine deaminase (EC 3.5.4.4) (Adenosine aminohydrolase).	Go Component not found
P01024	[Complement C3 precursor [Contains: Complement C3 beta chain;, Complement C3 alpha chain; C3a anaphylatoxin; Complement C3b alpha', chain; Complement C3c alpha' chain fragment 1; Complement C3dg, fragment; Complement C3g fragment; Complement C3d fragment; Complement, C3f fragment; Complement C3c alpha' chain fragment 2].]	Go Component not found
P01286	[Somatoliberin precursor (Growth hormone-releasing factor) (GRF), (Growth hormone-releasing hormone) (GHRH) (Somatocrinin) (Somatorelin), (Sermorelin).]	Go Component not found
P01350	[Gastrin precursor [Contains: Gastrin-71 (Gastrin component I);, Gastrin-52 (G52); Big gastrin (Gastrin-34) (G34) (Gastrin component, II); Gastrin (Gastrin-17) (G17) (Gastrin component III); Gastrin-14, (G14); Gastrin-6 (G6)].]	Go Component not found
P01568	Interferon alpha-21 precursor (Interferon alpha-F) (LeIF F).	Go Component not found
P01842	Ig lambda chain C regions.	Go Component not found
P01912	[HLA class II histocompatibility antigen, DR-1 beta chain precursor, (Clone P2-beta-3).]	Go Component not found
P02452	Collagen alpha-1(I) chain precursor (Alpha-1 type I collagen).	Go Component not found
P02545	Lamin-A/C (70 kDa lamin) (Renal carcinoma antigen NY-REN-32).	Go Component not found
P03956	[Interstitial collagenase precursor (EC 3.4.24.7) (Matrix, metalloproteinase-1) (MMP-1) (Fibroblast collagenase) [Contains: 22, kDa interstitial collagenase; 27 kDa interstitial collagenase].]	Go Component not found
P03992	[HLA class II histocompatibility antigen, DQB1*0602 beta chain, precursor (DQ(5)) (DC-1).]	Go Component not found
P04053	[DNA nucleotidylexotransferase (EC 2.7.7.31) (Terminal addition enzyme), (Terminal deoxynucleotidyltransferase) (Terminal transferase).]	Go Component not found
P05013	[Interferon alpha-6 precursor (Interferon alpha-K) (LeIF K) (Interferon, alpha-54).]	Go Component not found
P05165	[Propionyl-CoA carboxylase alpha chain, mitochondrial precursor, (EC 6.4.1.3) (PCCase subunit alpha) (Propanoyl-CoA:carbon dioxide, ligase subunit alpha).]	Go Component not found
P05813	[Beta-crystallin A3 [Contains: Beta-crystallin A3, isoform A1, Delta4, form; Beta-crystallin A3, isoform A1, Delta7 form; Beta-crystallin A3,, isoform A1, Delta8 form].]	Go Component not found
P06241	[Proto-oncogene tyrosine-protein kinase Fyn (EC 2.7.10.2) (p59-Fyn), (Protooncogene Syn) (SLK).]	Go Component not found
P07101	Tyrosine 3-monooxygenase (EC 1.14.16.2) (Tyrosine 3-hydroxylase) (TH).	Go Component not found
P07203	[Glutathione peroxidase 1 (EC 1.11.1.9) (GSHPx-1) (GPx-1) (Cellular, glutathione peroxidase).]	Go Component not found

P07686	[Beta-hexosaminidase beta chain precursor (EC 3.2.1.52) (N-acetyl-beta-, glucosaminidase) (Beta-N-acetylhexosaminidase) (Hexosaminidase B), (Cervical cancer proto-oncogene 7 protein) (HCC-7) [Contains: Beta-, hexosaminidase beta-B chain; Beta-hexosaminidase beta-A chain].]	Go Component not found
P21695	[Glycerol-3-phosphate dehydrogenase [NAD+], cytoplasmic (EC 1.1.1.8), (GPD-C) (GPDH-C).]	Go Component not found
P21781	[Keratinocyte growth factor precursor (KGF) (Fibroblast growth factor, 7) (FGF-7) (HBGF-7).]	Go Component not found
P21815	[Bone sialoprotein 2 precursor (Bone sialoprotein II) (BSP II) (Cell-, binding sialoprotein) (Integrin-binding sialoprotein).]	Go Component not found
P22004	Bone morphogenetic protein 6 precursor (BMP-6).	Go Component not found
P22087	[rRNA 2'-O-methyltransferase fibrillarin (EC 2.1.1.-) (34 kDa nucleolar, scleroderma antigen).]	Go Component not found
P22223	Cadherin-3 precursor (Placental cadherin) (P-cadherin).	Go Component not found
P22304	[Iduronate 2-sulfatase precursor (EC 3.1.6.13) (Alpha-L-iduronate, sulfate sulfatase) (Idursulfase) [Contains: Iduronate 2-sulfatase 42, kDa chain; Iduronate 2-sulfatase 14 kDa chain].]	Go Component not found
P22314	Ubiquitin-activating enzyme E1 (A1S9 protein).	Go Component not found
P22680	[Cytochrome P450 7A1 (Cholesterol 7-alpha-monooxygenase) (CYPVII), (EC 1.14.13.17) (Cholesterol 7-alpha-hydroxylase).]	Go Component not found
P23229	[Integrin alpha-6 precursor (VLA-6) (CD49f antigen) [Contains: Integrin, alpha-6 heavy chain; Integrin alpha-6 light chain].]	Go Component not found
P23515	Oligodendrocyte-myelin glycoprotein precursor.	Go Component not found
P23759	Paired box protein Pax-7 (HUP1).	Go Component not found
P24592	[Insulin-like growth factor-binding protein 6 precursor (IGFBP-6) (IBP-, 6) (IGF-binding protein 6).]	Go Component not found
P24821	[Tenascin precursor (TN) (Tenascin-C) (TN-C) (Hexabrachion), (Cytotactin) (Neuronectin) (GMEM) (JI) (Myotendinous antigen) (Glioma-, associated-extracellular matrix antigen) (GP 150-225).]	Go Component not found
P80108	[Phosphatidylinositol-glycan-specific phospholipase D precursor, (EC 3.1.4.50) (PI-G PLD) (Glycoprotein phospholipase D) (Glycosyl-, phosphatidylinositol-specific phospholipase D).]	Go Component not found
P80108	[Phosphatidylinositol-glycan-specific phospholipase D precursor, (EC 3.1.4.50) (PI-G PLD) (Glycoprotein phospholipase D) (Glycosyl-, phosphatidylinositol-specific phospholipase D).]	Go Component not found
P80108	[Phosphatidylinositol-glycan-specific phospholipase D precursor, (EC 3.1.4.50) (PI-G PLD) (Glycoprotein phospholipase D) (Glycosyl-, phosphatidylinositol-specific phospholipase D).]	Go Component not found
P80108	[Phosphatidylinositol-glycan-specific phospholipase D precursor, (EC 3.1.4.50) (PI-G PLD) (Glycoprotein phospholipase D) (Glycosyl-, phosphatidylinositol-specific phospholipase D).]	Go Component not found
P80108	[Phosphatidylinositol-glycan-specific phospholipase D precursor, (EC 3.1.4.50) (PI-G PLD) (Glycoprotein phospholipase D) (Glycosyl-, phosphatidylinositol-specific phospholipase D).]	Go Component not found
P80108	[Phosphatidylinositol-glycan-specific phospholipase D precursor, (EC 3.1.4.50) (PI-G PLD) (Glycoprotein phospholipase D) (Glycosyl-, phosphatidylinositol-specific phospholipase D).]	Go Component not found
P80108	[Phosphatidylinositol-glycan-specific phospholipase D precursor, (EC 3.1.4.50) (PI-G PLD) (Glycoprotein phospholipase D) (Glycosyl-, phosphatidylinositol-specific phospholipase D).]	Go Component not found
P80108	[Phosphatidylinositol-glycan-specific phospholipase D precursor, (EC 3.1.4.50) (PI-G PLD) (Glycoprotein phospholipase D) (Glycosyl-, phosphatidylinositol-specific phospholipase D).]	Go Component not found
P81274	G-protein-signaling modulator 2 (Mosaic protein LGN).	Go Component not found
P26371	[Keratin-associated protein 5-9 (Keratin-associated protein 5.9), (Ultrahigh sulfur keratin-associated protein 5.9) (Keratin, cuticle,, ultrahigh sulfur 1) (Keratin, ultra high-sulfur matrix protein A) (UHS, keratin A) (UHS KerA).]	Go Component not found
P26927	[Hepatocyte growth factor-like protein precursor (Macrophage, stimulatory protein) (MSP) (Macrophage-stimulating protein) [Contains:, Hepatocyte growth factor-like protein alpha chain; Hepatocyte growth, factor-like protein beta chain].]	Go Component not found

P26998	Beta-crystallin B3 (Beta-B3 crystallin).	Go Component not found
P27144	[Adenylate kinase isoenzyme 4, mitochondrial (EC 2.7.4.3) (Adenylate, kinase 3-like 1) (ATP-AMP transphosphorylase).]	Go Component not found
P27361	[Mitogen-activated protein kinase 3 (EC 2.7.11.24) (Extracellular, signal-regulated kinase 1) (ERK-1) (Insulin-stimulated MAP2 kinase), (MAP kinase 1) (MAPK 1) (p44-ERK1) (ERT2) (p44-MAPK) (Microtubule-, associated protein 2 kinase).]	Go Component not found
P27708	[CAD protein [Includes: Glutamine-dependent carbamoyl-phosphate, synthase (EC 6.3.5.5); Aspartate carbamoyltransferase (EC 2.1.3.2);, Dihydroorotase (EC 3.5.2.3)].]	Go Component not found
P28161	[Glutathione S-transferase Mu 2 (EC 2.5.1.18) (GSTM2-2) (GST class-mu, 2).]	Go Component not found
P28300	Protein-lysine 6-oxidase precursor (EC 1.4.3.13) (Lysyl oxidase).	Go Component not found
P28332	Alcohol dehydrogenase 6 (EC 1.1.1.1).	Go Component not found
P28715	[DNA-repair protein complementing XP-G cells (Xeroderma pigmentosum, group G-complementing protein) (DNA excision repair protein ERCC-5).]	Go Component not found
P29083	[Transcription initiation factor IIE subunit alpha (TFIIE-alpha), (General transcription factor IIE subunit 1) (General transcription, factor IIE 56 kDa subunit).]	Go Component not found
P30304	[M-phase inducer phosphatase 1 (EC 3.1.3.48) (Dual specificity, phosphatase Cdc25A).]	Go Component not found
P30414	[NK-tumor recognition protein (Natural-killer cells cyclophilin-related, protein) (NK-TR protein).]	Go Component not found
P30505	[HLA class I histocompatibility antigen, Cw-8 alpha chain precursor, (MHC class I antigen Cw*8).]	Go Component not found
P31213	[3-oxo-5-alpha-steroid 4-dehydrogenase 2 (EC 1.3.99.5) (Steroid 5-, alpha-reductase 2) (SR type 2) (5 alpha-SR2) (Type II 5-alpha, reductase).]	Go Component not found
P31271	Homeobox protein Hox-A13 (Hox-1J).	Go Component not found
P31323	cAMP-dependent protein kinase type II-beta regulatory subunit.	Go Component not found
P31689	[DnaJ homolog subfamily A member 1 (Heat shock 40 kDa protein 4) (DnaJ, protein homolog 2) (HDJ-2) (HSJ-2) (HSDJ).]	Go Component not found
P31939	[Bifunctional purine biosynthesis protein PURH [Includes:, Phosphoribosylaminoimidazolecarboxamide formyltransferase (EC 2.1.2.3), (5-aminoimidazole-4-carboxamide ribonucleotide formyltransferase), (AICAR transformylase); IMP cyclohydrolase (EC 3.5.4.10) (Inosinicase), (IMP synthetase) (ATIC)].]	Go Component not found
P32004	Neural cell adhesion molecule L1 precursor (N-CAM L1) (CD171 antigen).	Go Component not found
P32456	[Interferon-induced guanylate-binding protein 2 (GTP-binding protein 2), (Guanine nucleotide-binding protein 2) (GBP-2) (HuGBP-2).]	Go Component not found
P33764	Protein S100-A3 (S100 calcium-binding protein A3) (Protein S-100E).	Go Component not found
P34949	[Mannose-6-phosphate isomerase (EC 5.3.1.8) (Phosphomannose isomerase), (PMI) (Phosphohexomutase).]	Go Component not found
P35221	[Catenin alpha-1 (Cadherin-associated protein) (Alpha E-catenin) (NY-, REN-13 antigen).]	Go Component not found
P35241	Radixin.	Go Component not found
P35442	Thrombospondin-2 precursor.	Go Component not found
P35475	Alpha-L-iduronidase precursor (EC 3.2.1.76).	Go Component not found
P35503	[UDP-glucuronosyltransferase 1-3 precursor (EC 2.4.1.17) (UDP-, glucuronosyltransferase 1A3) (UDPGT) (UGT1*3) (UGT1-03) (UGT1.3) (UGT-, 1C) (UGT1C).]	Go Component not found
P35558	[Phosphoenolpyruvate carboxykinase, cytosolic [GTP] (EC 4.1.1.32), (Phosphoenolpyruvate carboxylase) (PEPCK-C).]	Go Component not found
P35626	[Beta-adrenergic receptor kinase 2 (EC 2.7.11.15) (Beta-ARK-2) (G-, protein-coupled receptor kinase 3).]	Go Component not found
P35711	Transcription factor SOX-5.	Go Component not found

P38935	[DNA-binding protein SMUBP-2 (EC 3.6.1.-) (ATP-dependent helicase, IGHMBP2) (Immunoglobulin mu-binding protein 2) (SMUBP-2) (Glial factor, 1) (GF-1).]	Go Component not found
P39880	Homeobox protein cut-like 1 (CCAAT displacement protein) (CDP).	Go Component not found
P40261	Nicotinamide N-methyltransferase (EC 2.1.1.1).	Go Component not found
P41091	[Eukaryotic translation initiation factor 2 subunit 3 (Eukaryotic, translation initiation factor 2 subunit gamma) (eIF-2-gamma).]	Go Component not found
P41229	[Histone demethylase JARID1C (EC 1.14.11.-) (Jumonji/ARID domain-, containing protein 1C) (Protein SmcX) (Protein Xe169).]	Go Component not found
P58005	Sestrin-3.	Go Component not found
P58166	Inhibin beta E chain precursor (Activin beta-E chain).	Go Component not found
P58335	[Anthrax toxin receptor 2 precursor (Capillary morphogenesis gene 2, protein) (CMG-2).]	Go Component not found
P60983	Glia maturation factor beta (GMF-beta).	Go Component not found
P61024	Cyclin-dependent kinases regulatory subunit 1 (CKS-1).	Go Component not found
P61371	Insulin gene enhancer protein ISL-1 (Islet-1).	Go Component not found
P61769	[Beta-2-microglobulin precursor [Contains: Beta-2-microglobulin form pI, 5.3].]	Go Component not found
P61921	[Hemoglobin subunit gamma-2 (Hemoglobin gamma-2 chain) (Gamma-2-globin), (Hemoglobin gamma-G chain).]	Go Component not found
P61981	14-3-3 protein gamma (Protein kinase C inhibitor protein 1) (KCIP-1).	Go Component not found
P62136	[Serine/threonine-protein phosphatase PP1-alpha catalytic subunit, (EC 3.1.3.16) (PP-1A).]	Go Component not found
P62508	[Estrogen-related receptor gamma (Estrogen receptor-related protein 3), (ERR gamma-2).]	Go Component not found
P62834	[Ras-related protein Rap-1A precursor (GTP-binding protein smg-p21A), (Ras-related protein Krev-1) (C21KG) (G-22K).]	Go Component not found
P69891	[Hemoglobin subunit gamma-1 (Hemoglobin gamma-1 chain) (Gamma-1-globin), (Hemoglobin gamma-A chain) (Hb F Agamma).]	Go Component not found
P78329	[Cytochrome P450 4F2 (EC 1.14.13.30) (CYPIVF2) (Leukotriene-B(4) omega-, hydroxylase) (Leukotriene-B(4) 20-monooxygenase) (Cytochrome P450-LTB-, omega).]	Go Component not found
P78368	Casein kinase I isoform gamma-2 (EC 2.7.11.1) (CKI-gamma 2).	Go Component not found
P78395	[Melanoma antigen preferentially expressed in tumors (Preferentially, expressed antigen of melanoma) (OPA-interacting protein 4) (OIP4).]	Go Component not found
P78426	Homeobox protein Nkx-6.1.	Go Component not found
P78563	[Double-stranded RNA-specific editase 1 (EC 3.5.-.-) (dsRNA adenosine, deaminase) (RNA-editing deaminase 1) (RNA-editing enzyme 1).]	Go Component not found
Q9BZQ6	ER degradation-enhancing alpha-mannosidase-like 3.	Go Component not found
Q9C005	Dpy-30-like protein.	Go Component not found
P82279	Crumbs homolog 1 precursor.	Go Component not found
Q01518	Adenylyl cyclase-associated protein 1 (CAP 1).	Go Component not found
Q01638	Interleukin-1 receptor-like 1 precursor (ST2 protein).	Go Component not found
Q01804	OTU domain-containing protein 4 (HIV-1-induced protein HIN-1).	Go Component not found
Q02040	[Splicing factor, arginine/serine-rich 17A (Protein XE7) (B-lymphocyte, antigen) (721P).]	Go Component not found
Q02750	[Dual specificity mitogen-activated protein kinase kinase 1, (EC 2.7.12.2) (MAP kinase kinase 1) (MAPKK 1) (ERK activator kinase 1), (MAPK/ERK kinase 1) (MEK1).]	Go Component not found
Q02962	Paired box protein Pax-2.	Go Component not found

Q03052	[POU domain, class 3, transcription factor 1 (Octamer-binding, transcription factor 6) (Oct-6) (POU domain transcription factor, SCIP).]	Go Component not found
Q03113	Guanine nucleotide-binding protein alpha-12 subunit (G alpha-12).	Go Component not found
Q03468	[DNA excision repair protein ERCC-6 (EC 3.6.1.-) (ATP-dependent, helicase ERCC6) (Cockayne syndrome protein CSB).]	Go Component not found
Q05086	[Ubiquitin-protein ligase E3A (EC 6.3.2.-) (E6AP ubiquitin-protein, ligase) (Oncogenic protein-associated protein E6-AP) (Human, papillomavirus E6-associated protein) (Renal carcinoma antigen NY-REN-, 54).]	Go Component not found
Q05215	Early growth response protein 4 (EGR-4) (AT133).	Go Component not found
Q05639	[Elongation factor 1-alpha 2 (EF-1-alpha-2) (Elongation factor 1 A-2), (eEF1A-2) (Statin S1).]	Go Component not found
Q05823	[2-5A-dependent ribonuclease (EC 3.1.26.-) (2-5A-dependent RNase), (Ribonuclease L) (RNase L) (Ribonuclease 4).]	Go Component not found
Q06889	Early growth response protein 3 (EGR-3) (Zinc finger protein pilot).	Go Component not found
Q08493	[cAMP-specific 3',5'-cyclic phosphodiesterase 4C (EC 3.1.4.17) (DPDE1), (PDE21).]	Go Component not found
Q10586	[D site-binding protein (Albumin D box-binding protein) (Albumin D-, element-binding protein) (TAXREB302).]	Go Component not found
Q12767	Uncharacterized protein KIAA0195 (Transmembrane protein 94).	Go Component not found
Q12872	[Splicing factor, arginine/serine-rich 8 (Suppressor of white apricot, protein homolog).]	Go Component not found
Q12899	[Tripartite motif-containing protein 26 (Zinc finger protein 173) (Acid, finger protein) (AFP) (RING finger protein 95).]	Go Component not found
Q12923	[Tyrosine-protein phosphatase non-receptor type 13 (EC 3.1.3.48), (Protein-tyrosine phosphatase 1E) (PTP-E1) (hPTPE1) (PTP-BAS), (Protein-tyrosine phosphatase PTPL1) (Fas-associated protein-tyrosine, phosphatase 1) (FAP-1).]	Go Component not found
Q12933	[TNF receptor-associated factor 2 (Tumor necrosis factor type 2, receptor-associated protein 3).]	Go Component not found
Q12979	Active breakpoint cluster region-related protein.	Go Component not found
Q13007	[Interleukin-24 precursor (Suppression of tumorigenicity 16 protein), (Melanoma differentiation-associated gene 7 protein) (MDA-7).]	Go Component not found
Q13075	[Baculoviral IAP repeat-containing protein 1 (Neuronal apoptosis, inhibitory protein).]	Go Component not found
Q13098	[COP9 signalosome complex subunit 1 (Signalosome subunit 1) (SGN1), (JAB1-containing signalosome subunit 1) (G protein pathway suppressor, 1) (Protein GPS1) (Protein MFH).]	Go Component not found
Q13163	[Dual specificity mitogen-activated protein kinase kinase 5, (EC 2.7.12.2) (MAP kinase kinase 5) (MAPKK 5) (MAPK/ERK kinase 5).]	Go Component not found
Q13191	[E3 ubiquitin-protein ligase CBL-B (EC 6.3.2.-) (Signal transduction, protein CBL-B) (SH3-binding protein CBL-B) (Casitas B-lineage lymphoma, proto-oncogene b) (RING finger protein 56).]	Go Component not found
Q13206	[Probable ATP-dependent RNA helicase DDX10 (EC 3.6.1.-) (DEAD box, protein 10).]	Go Component not found
Q13227	G protein pathway suppressor 2 (Protein GPS2).	Go Component not found
Q13237	[cGMP-dependent protein kinase 2 (EC 2.7.11.12) (CGK 2) (cGKII) (Type, II cGMP-dependent protein kinase).]	Go Component not found
Q13330	Metastasis-associated protein MTA1.	Go Component not found
Q13351	[Krueppel-like factor 1 (Erythroid krueppel-like transcription factor), (EKLF) (Erythroid transcription factor).]	Go Component not found
Q13370	[cGMP-inhibited 3',5'-cyclic phosphodiesterase B (EC 3.1.4.17) (Cyclic, GMP-inhibited phosphodiesterase B) (CGI-PDE B) (CGIPDE1) (CGIP1).]	Go Component not found
Q92989	Pre-mRNA cleavage complex II protein Clp1.	Go Component not found
Q92995	[Ubiquitin carboxyl-terminal hydrolase 13 (EC 3.1.2.15) (Ubiquitin, thioesterase 13) (Ubiquitin-specific-processing protease 13), (Deubiquitinating enzyme 13) (Isopeptidase T-3) (ISOT-3).]	Go Component not found
Q93075	TatD DNase domain-containing deoxyribonuclease 2 (EC 3.1.21.-).	Go Component not found
Q93088	Betaine--homocysteine S-methyltransferase 1 (EC 2.1.1.5).	Go Component not found

Q95604	[HLA class I histocompatibility antigen, Cw-17 alpha chain precursor, (MHC class I antigen Cw*17).]	Go Component not found
Q96NI6	[Leucine-rich repeat and fibronectin type-III domain-containing protein, 5 precursor.]	Go Component not found
Q99487	[Platelet-activating factor acetylhydrolase 2, cytoplasmic, (EC 3.1.1.47) (Serine-dependent phospholipase A2) (HSD-PLA2).]	Go Component not found
Q99518	[Dimethylaniline monooxygenase [N-oxide-forming] 2 (EC 1.14.13.8), (Pulmonary flavin-containing monooxygenase 2) (FMO 2) (Dimethylaniline, oxidase 2) (FMO 1B1).]	Go Component not found
Q99574	Neuroserpin precursor (Serpin I1) (Protease inhibitor 12).	Go Component not found
Q99611	[Selenide, water dikinase 2 (EC 2.7.9.3) (Selenophosphate synthetase 2), (Selenium donor protein 2).]	Go Component not found
Q99684	[Zinc finger protein Gfi-1 (Growth factor independent protein 1) (Zinc, finger protein 163).]	Go Component not found
Q99743	[Neuronal PAS domain-containing protein 2 (Neuronal PAS2) (Member of, PAS protein 4) (Basic-helix-loop-helix-PAS protein MOP4).]	Go Component not found
Q99865	[Spindlin-2A (Spindlin-like protein 2A) (SPIN-2A) (SPIN-2) (Protein, DXF34).]	Go Component not found
Q99935	[Proline-rich protein 1 precursor (PRL1) (Basic proline-rich lacrimal, protein).]	Go Component not found
Q99961	[SH3-containing GRB2-like protein 1 (Endophilin-2) (Endophilin-A2) (SH3, domain protein 2B) (Extra eleven-nineteen leukemia fusion gene, protein) (EEN) (EEN fusion partner of MLL).]	Go Component not found
Q9BPZ7	[Stress-activated map kinase-interacting protein 1 (SAPK-interacting, protein 1) (Putative Ras inhibitor JC310).]	Go Component not found
Q9BSY9	UPF0326 protein C1orf121.	Go Component not found
Q9BU70	Nef-associated protein 1 (EC 3.1.2.-) (Thioesterase NAP1).	Go Component not found
Q9BXT8	RING finger protein 17.	Go Component not found
Q9H1E3	Nuclear ubiquitous casein and cyclin-dependent kinases substrate (P1).	Go Component not found
Q9H4B7	Tubulin beta-1 chain.	Go Component not found
Q9H4W6	[Transcription factor COE3 (Early B-cell factor 3) (EBF-3) (Olf-1/EBF-, like 2) (OE-2) (O/E-2).]	Go Component not found
Q9H9Y6	[DNA-directed RNA polymerase I subunit RPA2 (EC 2.7.7.6) (RNA, polymerase I subunit 2) (DNA-directed RNA polymerase I 135 kDa, polypeptide) (RPA135).]	Go Component not found
Q9HCL2	[Glycerol-3-phosphate acyltransferase, mitochondrial precursor, (EC 2.3.1.15) (GPAT).]	Go Component not found
Q9NQ11	Probable cation-transporting ATPase 13A2 (EC 3.6.3.-).	Go Component not found
Q9NQV7	PR domain zinc finger protein 9 (PR domain-containing protein 9).	Go Component not found
Q9NQX3	Gephyrin.	Go Component not found
Q9NR09	[Baculoviral IAP repeat-containing protein 6 (Ubiquitin-conjugating BIR, domain enzyme apollon).]	Go Component not found
Q9NRC9	[Otoraplin precursor (Fibrocyte-derived protein) (Melanoma inhibitory, activity-like protein).]	Go Component not found
Q9NSE2	[Cytokine-inducible SH2-containing protein (CIS) (CIS-1) (Suppressor of, cytokine signaling) (SOCS) (Protein G18).]	Go Component not found
Q9NSV4	Protein diaphanous homolog 3 (Diaphanous-related formin-3) (DRF3).	Go Component not found
Q9NUA8	Zinc finger and BTB domain-containing protein 40.	Go Component not found
Q9NVP1	[ATP-dependent RNA helicase DDX18 (EC 3.6.1.-) (DEAD box protein 18), (Myc-regulated DEAD box protein) (MrDb).]	Go Component not found
Q9NY30	Protein BTG4 (PC3b).	Go Component not found
Q9NY65	Tubulin alpha-8 chain (Alpha-tubulin 8).	Go Component not found
Q9NZ56	Formin-2.	Go Component not found
Q9NZL3	[Zinc finger protein 224 (Zinc finger protein 27) (Zinc finger protein, 233) (Zinc finger protein 255) (Bone marrow zinc finger 2) (BMZF-2), (Zinc finger protein KOX22).]	Go Component not found

Q9P0K8	Forkhead box protein J2 (Fork head homologous X).	Go Component not found
Q9P2G3	Kelch-like protein 14.	Go Component not found
Q9P2K6	Kelch domain-containing protein 5.	Go Component not found
Q9P2Q2	FERM domain-containing protein 4A.	Go Component not found
Q9UBK7	Rab-like protein 2A.	Go Component not found
Q9UBQ6	[Exostosin-like 2 (EC 2.4.1.223) (Glucuronyl-galactosyl-proteoglycan 4-, alpha-N-acetylglucosaminyltransferase) (Alpha-1,4-N-, acetylhexosaminyltransferase EXTL2) (Alpha-GalNAcT EXTL2) (EXT-related, protein 2) [Contains: Processed exostosin-like 2].]	Go Component not found
Q9UBT3	[Dickkopf-related protein 4 precursor (Dkk-4) (Dickkopf-4) (hDkk-4), [Contains: Dickkopf-related protein 4 short form].]	Go Component not found
Q9UBW7	[MYM-type zinc finger protein 2 (Zinc finger protein 198) (Fused in, myeloproliferative disorders protein) (Rearranged in atypical, myeloproliferative disorder protein).]	Go Component not found
Q9UGL9	Protein NICE-1.	Go Component not found
Q9UH64	Susceptibility protein NSG-x.	Go Component not found
Q9UHI8	[ADAMTS-1 precursor (EC 3.4.24.-) (A disintegrin and metalloproteinase, with thrombospondin motifs 1) (ADAM-TS 1) (ADAM-TS1) (METH-1).]	Go Component not found
Q9UHY8	Fasciculation and elongation protein zeta 2 (Zygin-2) (Zygin II).	Go Component not found
Q9UI43	[Putative ribosomal RNA methyltransferase 2 (EC 2.1.1.-) (rRNA, (uridine-2'-O-)-methyltransferase).]	Go Component not found
Q9UIV8	[Serpin B13 (Hurpin) (HaCaT UV-repressible serpin) (Proteinase, inhibitor 13) (Headpin).]	Go Component not found
Q9UJF2	Ras GTPase-activating protein nGAP (RAS protein activator-like 1).	Go Component not found
Q9UK10	Zinc finger protein 225.	Go Component not found
Q9UK13	Zinc finger protein 221.	Go Component not found
Q9UK99	F-box only protein 3.	Go Component not found
Q9UKJ8	[ADAM 21 precursor (EC 3.4.24.-) (A disintegrin and metalloproteinase, domain 21).]	Go Component not found
Q9UKN5	PR domain zinc finger protein 4 (PR domain-containing protein 4).	Go Component not found
Q9UKP5	[ADAMTS-6 precursor (EC 3.4.24.-) (A disintegrin and metalloproteinase, with thrombospondin motifs 6) (ADAM-TS 6) (ADAM-TS6).]	Go Component not found
Q9UL15	[BAG family molecular chaperone regulator 5 (Bcl-2-associated, athanogene 5) (BAG-5).]	Go Component not found
Q9ULC6	[Protein-arginine deiminase type-1 (EC 3.5.3.15) (Protein-arginine, deiminase type I) (Peptidylarginine deiminase I).]	Go Component not found
Q9ULJ3	[Zinc finger protein 295 (Zinc finger and BTB domain-containing protein, 21).]	Go Component not found
Q9ULS5	Transmembrane and coiled-coil domains protein 3.	Go Component not found
Q9ULW8	[Protein-arginine deiminase type-3 (EC 3.5.3.15) (Protein-arginine, deiminase type III) (Peptidylarginine deiminase III).]	Go Component not found
Q9UM07	[Protein-arginine deiminase type-4 (EC 3.5.3.15) (Protein-arginine, deiminase type IV) (Peptidylarginine deiminase IV) (HL-60 PAD).]	Go Component not found
Q9UN75	Protocadherin alpha 12 precursor (PCDH-alpha12).	Go Component not found
Q9UNU6	[Cytochrome P450 8B1 (EC 1.14.13.95) (CYPVIIIB1) (7-alpha-, hydroxycholest-4-en-3-one 12-alpha-hydroxylase) (Sterol 12-alpha-, hydroxylase) (7-alpha-hydroxy-4-cholesten-3-one 12-alpha-hydroxylase).]	Go Component not found
Q9UP79	[ADAMTS-8 precursor (EC 3.4.24.-) (A disintegrin and metalloproteinase, with thrombospondin motifs 8) (ADAM-TS 8) (ADAM-TS8) (METH-2) (METH-, 8).]	Go Component not found
Q9UPG8	Zinc finger protein PLAGL2 (Pleiomorphic adenoma-like protein 2).	Go Component not found
Q9Y236	Oxidative stress induced growth inhibitor 2 (hT41).	Go Component not found

Q9Y281	Cofilin-2 (Cofilin, muscle isoform).	Go Component not found
Q9Y2A7	[Nck-associated protein 1 (NAP 1) (p125Nap1) (Membrane-associated, protein HEM-2).]	Go Component not found
Q9Y388	RNA-binding motif protein, X-linked 2.	Go Component not found
O00139	Kinesin-like protein KIF2A (Kinesin-2) (HK2).	Go Component not found
O00182	Galectin-9 (HOM-HD-21) (Ecalectin).	Go Component not found
O00212	[Rho-related GTP-binding protein RhoD precursor (Rho-related protein, HP1) (RhoHP1).]	Go Component not found
O00408	[cGMP-dependent 3',5'-cyclic phosphodiesterase (EC 3.1.4.17) (Cyclic, GMP-stimulated phosphodiesterase) (CGS-PDE) (cGSPDE).]	Go Component not found
O00459	[Phosphatidylinositol 3-kinase regulatory subunit beta (PI3-kinase p85, subunit beta) (PtdIns-3-kinase p85-beta).]	Go Component not found
O00515	[Ladinin 1 (Lad-1) (120 kDa linear IgA bullous dermatosis antigen) (97, kDa linear IgA bullous dermatosis antigen) (Linear IgA disease antigen, homolog) (LadA).]	Go Component not found
O00744	Protein Wnt-10b precursor (Wnt-12).	Go Component not found
O00755	Protein Wnt-7a precursor.	Go Component not found
O00767	[Acyl-CoA desaturase (EC 1.14.19.1) (Stearoyl-CoA desaturase) (Fatty, acid desaturase) (Delta(9)-desaturase).]	Go Component not found
O14531	[Dihydropyrimidinase-related protein 4 (DRP-4) (Collapsin response, mediator protein 3) (CRMP-3) (UNC33-like phosphoprotein 4) (ULIP4, protein).]	Go Component not found
O14578	[Citron Rho-interacting kinase (EC 2.7.11.1) (CRIK) (Rho-interacting,, serine/threonine-protein kinase 21).]	Go Component not found
O14598	[Testis-specific basic protein Y 1 (Variably charged protein Y) (Basic, charge, Y-linked 1).]	Go Component not found
O14625	[Small-inducible cytokine B11 precursor (CXCL11) (Interferon-inducible, T-cell alpha chemoattractant) (I-TAC) (Interferon-gamma-inducible, protein 9) (IP-9) (H174) (Beta-R1).]	Go Component not found
O14647	[Chromodomain-helicase-DNA-binding protein 2 (EC 3.6.1.-) (ATP-, dependent helicase CHD2) (CHD-2).]	Go Component not found
O14681	Etoposide-induced protein 2.4 (p53-induced gene 8 protein).	Go Component not found
O14732	[Inositol monophosphatase 2 (EC 3.1.3.25) (IMPase 2) (IMP 2) (Inositol-, 1(or 4)-monophosphatase 2) (Myo-inositol monophosphatase A2).]	Go Component not found
O14793	Growth/differentiation factor 8 precursor (GDF-8) (Myostatin).	Go Component not found
O14813	[Paired mesoderm homeobox protein 2A (Paired-like homeobox 2A), (Aristaless homeobox protein homolog) (ARIX1 homeodomain protein).]	Go Component not found
O14829	[Serine/threonine-protein phosphatase with EF-hands 1 (EC 3.1.3.16), (PPEF-1) (Protein phosphatase with EF calcium-binding domain) (PPEF), (Serine/threonine-protein phosphatase 7) (PP7).]	Go Component not found
O14862	Interferon-inducible protein AIM2 (Absent in melanoma 2).	Go Component not found
O14958	Calsequestrin-2 precursor (Calsequestrin, cardiac muscle isoform).	Go Component not found
O14976	Cyclin G-associated kinase (EC 2.7.11.1).	Go Component not found
O15016	Tripartite motif-containing protein 66.	Go Component not found
O15105	[Mothers against decapentaplegic homolog 7 (SMAD 7) (Mothers against, DPP homolog 7) (Smad7) (hSMAD7).]	Go Component not found
O15131	Importin subunit alpha-6 (Karyopherin subunit alpha-5).	Go Component not found
O15178	Brachyury protein (T protein).	Go Component not found
O15197	[Ephrin type-B receptor 6 precursor (Tyrosine-protein kinase-defective, receptor EPH-6) (HEP).]	Go Component not found
O15230	Laminin subunit alpha-5 precursor.	Go Component not found

O15264	[Mitogen-activated protein kinase 13 (EC 2.7.11.24) (Stress-activated, protein kinase 4) (Mitogen-activated protein kinase p38 delta) (MAP, kinase p38 delta).]	Go Component not found
O15269	[Serine palmitoyltransferase 1 (EC 2.3.1.50) (Long chain base, biosynthesis protein 1) (LCB 1) (Serine-palmitoyl-CoA transferase 1), (SPT 1) (SPT1).]	Go Component not found
O15480	Melanoma-associated antigen B3 (MAGE-B3 antigen).	Go Component not found
O15519	[CASP8 and FADD-like apoptosis regulator precursor (Cellular FLICE-like, inhibitory protein) (c-FLIP) (Caspase-eight-related protein) (Casper), (Caspase-like apoptosis regulatory protein) (CLARP) (MACH-related, inducer of toxicity) (MRIT) (Caspase homolog) (CASH) (Inhibitor of, FLICE) (I-FLICE) (FADD-like antiapoptotic molecule 1) (FLAME-1), (Usurpin) [Contains: CASP8 and FADD-like apoptosis regulator subunit, p43; CASP8 and FADD-like apoptosis regulator subunit p12].]	Go Component not found
O15534	[Period circadian protein homolog 1 (Circadian clock protein PERIOD 1), (Circadian pacemaker protein Rigui) (hPER1).]	Go Component not found
O15544	Protein GR6.	Go Component not found
O43151	Uncharacterized protein KIAA0401.	Go Component not found
O43236	[Septin-4 (Peanut-like protein 2) (Brain protein H5) (Cell division, control-related protein 2) (hCDCREL-2) (Bradeion beta) (CE5B3 beta), (Cerebral protein 7).]	Go Component not found
O43301	Heat shock 70 kDa protein 12A.	Go Component not found
O43309	[Zinc finger and SCAN domain-containing protein 12 (Zinc finger protein, 96) (Zinc finger protein 305).]	Go Component not found
O43316	Paired box protein Pax-4.	Go Component not found
O43364	Homeobox protein Hox-A2.	Go Component not found
O43399	Tumor protein D54 (hD54) (Tumor protein D52-like 2).	Go Component not found
O43488	[Aflatoxin B1 aldehyde reductase member 2 (EC 1.-.-.-) (AFB1-AR 1), (Aldoketoreductase 7).]	Go Component not found
O43548	[Protein-glutamine gamma-glutamyltransferase 5 (EC 2.3.2.13), (Transglutaminase-5) (TGase 5) (Transglutaminase X) (TGase X) (TGX), (TG(X)).]	Go Component not found
O43566	Regulator of G-protein signaling 14 (RGS14).	Go Component not found
O43665	Regulator of G-protein signaling 10 (RGS10).	Go Component not found
O43900	LIM domain only protein 6 (Triple LIM domain protein 6).	Go Component not found
O60229	[Kalirin (Huntingtin-associated protein-interacting protein) (Protein, Duo).]	Go Component not found
O60285	[NUAK family SNF1-like kinase 1 (EC 2.7.11.1) (AMPK-related protein, kinase 5).]	Go Component not found
O60309	Leucine-rich repeat-containing protein 37A.	Go Component not found
O60481	Zinc finger protein ZIC 3 (Zinc finger protein of the cerebellum 3).	Go Component not found
O60496	Docking protein 2 (Downstream of tyrosine kinase 2) (p56(dok-2)).	Go Component not found
O60504	[Vinexin (Sorbin and SH3 domain-containing protein 3) (SH3-containing, adapter molecule 1) (SCAM-1).]	Go Component not found
O60551	[Glycylpeptide N-tetradecanoyltransferase 2 (EC 2.3.1.97) (Peptide N-, myristoyltransferase 2) (Myristoyl-CoA:protein N-myristoyltransferase, 2) (NMT 2) (Type II N-myristoyltransferase).]	Go Component not found
O60662	[Kelch repeat and BTB domain-containing protein 10 (Kelch-related, protein 1) (Kel-like protein 23) (Sarcosin).]	Go Component not found
O60673	DNA polymerase zeta catalytic subunit (EC 2.7.7.7) (hREV3).	Go Component not found
O60711	Leupaxin.	Go Component not found
O60806	[T-box transcription factor TBX19 (T-box protein 19) (T-box factor,, pituitary).]	Go Component not found
O60861	Growth arrest-specific protein 7 (GAS-7).	Go Component not found
O60885	Bromodomain-containing protein 4 (HUNK1 protein).	Go Component not found

O75038	[1-phosphatidylinositol-4,5-bisphosphate phosphodiesterase eta-2, (EC 3.1.4.11) (Phosphoinositide phospholipase C) (Phospholipase C-eta-, 2) (PLC-eta-2) (Phosphoinositide phospholipase C-like 4), (Phospholipase C-like 4) (Fragment).]	Go Component not found
O75340	[Programmed cell death protein 6 (Apoptosis-linked gene 2 protein), (Probable calcium-binding protein ALG-2).]	Go Component not found
O75364	Pituitary homeobox 3 (Homeobox protein PITX3).	Go Component not found
O75410	[Transforming acidic coiled-coil-containing protein 1 (Taxin 1), (Gastric cancer antigen Ga55).]	Go Component not found
O75439	[Mitochondrial-processing peptidase subunit beta, mitochondrial, precursor (EC 3.4.24.64) (Beta-MPP) (P-52).]	Go Component not found
O75562	Protein HFSE-1.	Go Component not found
O75604	[Ubiquitin carboxyl-terminal hydrolase 2 (EC 3.1.2.15) (Ubiquitin, thioesterase 2) (Ubiquitin-specific-processing protease 2), (Deubiquitinating enzyme 2) (41 kDa ubiquitin-specific protease).]	Go Component not found
O75631	Uroplakin-3A precursor (Uroplakin III) (UPIII).	Go Component not found
O75688	[Protein phosphatase 1B (EC 3.1.3.16) (Protein phosphatase 2C isoform, beta) (PP2C-beta).]	Go Component not found
O75715	[Epididymal secretory glutathione peroxidase precursor (EC 1.11.1.9), (Epididymis-specific glutathione peroxidase-like protein) (EGLP).]	Go Component not found
O75820	Zinc finger protein 189.	Go Component not found
O75881	[Cytochrome P450 7B1 (EC 1.14.13.100) (25-hydroxycholesterol 7-alpha-, hydroxylase) (Oxysterol 7-alpha-hydroxylase).]	Go Component not found
O75897	[Sulfotransferase 1C4 (EC 2.8.2.-) (SULT1C) (Sulfotransferase 1C2), (SULT1C#2).]	Go Component not found
P07864	[L-lactate dehydrogenase C chain (EC 1.1.1.27) (LDH-C) (LDH testis, subunit) (LDH-X) (Cancer/testis antigen 32) (CT32).]	Go Component not found
P08236	Beta-glucuronidase precursor (EC 3.2.1.31) (Beta-G1).	Go Component not found
P09067	Homeobox protein Hox-B5 (Hox-2A) (HHO.C10) (HU-1).	Go Component not found
P09668	[Cathepsin H precursor (EC 3.4.22.16) [Contains: Cathepsin H mini, chain; Cathepsin H heavy chain; Cathepsin H light chain].]	Go Component not found
P09769	[Proto-oncogene tyrosine-protein kinase FGR (EC 2.7.10.2) (P55-FGR) (C-, FGR).]	Go Component not found
P09917	Arachidonate 5-lipoxygenase (EC 1.13.11.34) (5-lipoxygenase) (5-LO).	Go Component not found
P09972	Fructose-bisphosphate aldolase C (EC 4.1.2.13) (Brain-type aldolase).	Go Component not found
P10070	Zinc finger protein GLI2 (Tax helper protein).	Go Component not found
P10074	[Zinc finger and BTB domain-containing protein 48 (Krueppel-related, zinc finger protein 3) (Protein HKR3).]	Go Component not found
P10267	[HERV-K_5q33.3 provirus ancestral Env polyprotein (Envelope, polyprotein) (HERV-K10 envelope protein) (HERV-K107 envelope protein), [Contains: Surface protein (SU); Transmembrane protein (TM)].]	Go Component not found
P10398	[A-Raf proto-oncogene serine/threonine-protein kinase (EC 2.7.11.1) (A-, raf-1) (Proto-oncogene Pks).]	Go Component not found
P10645	[Chromogranin-A precursor (CgA) (Pituitary secretory protein I) (SP-I), [Contains: Vasostatin-1 (Vasostatin I); Vasostatin-2 (Vasostatin II);, EA-92; ES-43; Pancreastatin; SS-18; WA-8; WE-14; LF-19; AL-11; GV-19;, GR-44; ER-37].]	Go Component not found
P10745	[Interphotoreceptor retinoid-binding protein precursor (IRBP), (Interstitial retinol-binding protein).]	Go Component not found
P10827	[Thyroid hormone receptor alpha (C-erbA-alpha) (c-erbA-1) (EAR-7), (EAR7).]	Go Component not found
P11086	[Phenylethanolamine N-methyltransferase (EC 2.1.1.28) (PNMTase), (Noradrenaline N-methyltransferase).]	Go Component not found
P11161	Early growth response protein 2 (EGR-2) (Protein Krox-20) (AT591).	Go Component not found
P11245	[Arylamine N-acetyltransferase 2 (EC 2.3.1.5) (Arylamide acetylase 2), (Polymorphic arylamine N-acetyltransferase) (PNAT) (N-, acetyltransferase type 2) (NAT-2).]	Go Component not found
P11441	Ubiquitin-like protein 4A (Ubiquitin-like protein GDX).	Go Component not found
P11597	[Cholesteryl ester transfer protein precursor (Lipid transfer protein, I).]	Go Component not found

P11801	Serine/threonine-protein kinase H1 (EC 2.7.11.1) (PSK-H1).	Go Component not found
P12525	L-myc-2 protein.	Go Component not found
P12644	Bone morphogenetic protein 4 precursor (BMP-4) (BMP-2B).	Go Component not found
P13073	[Cytochrome c oxidase subunit 4 isoform 1, mitochondrial precursor, (EC 1.9.3.1) (Cytochrome c oxidase subunit IV isoform 1) (COX IV-1), (Cytochrome c oxidase polypeptide IV).]	Go Component not found
P13498	[Cytochrome b-245 light chain (p22 phagocyte B-cytochrome) (Neutrophil, cytochrome b 22 kDa polypeptide) (p22-phox) (p22phox) (Cytochrome, b(558) alpha chain) (Cytochrome b558 subunit alpha) (Superoxide-, generating NADPH oxidase light chain subunit).]	Go Component not found
P13646	[Keratin, type I cytoskeletal 13 (Cytokeratin-13) (CK-13) (Keratin-13), (K13).]	Go Component not found
P13671	Complement component C6 precursor.	Go Component not found
P13716	[Delta-aminolevulinic acid dehydratase (EC 4.2.1.24) (Porphobilinogen, synthase) (ALADH).]	Go Component not found
P14222	[Perforin-1 precursor (P1) (Lymphocyte pore-forming protein) (PFP), (Cytolysin).]	Go Component not found
P14550	[Alcohol dehydrogenase [NADP+] (EC 1.1.1.2) (Aldehyde reductase) (Aldo-, keto reductase family 1 member A1).]	Go Component not found
P15086	[Carboxypeptidase B precursor (EC 3.4.17.2) (Pancreas-specific protein), (PASP).]	Go Component not found
P15622	Zinc finger protein 250 (Zinc finger protein 647).	Go Component not found
P15918	[V(D)J recombination-activating protein 1 (RAG-1) (RING finger protein, 74).]	Go Component not found
P16278	[Beta-galactosidase precursor (EC 3.2.1.23) (Lactase) (Acid beta-, galactosidase) (Elastin receptor 1).]	Go Component not found
P16383	[GC-rich sequence DNA-binding factor (GCF) (Transcription factor 9), (TCF-9).]	Go Component not found
P16415	Zinc finger protein ZFP-36.	Go Component not found
P16444	[Dipeptidase 1 precursor (EC 3.4.13.19) (Microsomal dipeptidase) (Renal, dipeptidase) (hRDP) (Dehydropeptidase-I).]	Go Component not found
P16499	[Rod cGMP-specific 3',5'-cyclic phosphodiesterase subunit alpha, (EC 3.1.4.35) (GMP-PDE alpha) (PDE V-B1).]	Go Component not found
P16930	[Fumarylacetoacetase (EC 3.7.1.2) (Fumarylacetoacetate hydrolase), (Beta-diketonase) (FAA).]	Go Component not found
P17021	Zinc finger protein 17 (Zinc finger protein KOX10) (HPF3).	Go Component not found
P17050	[Alpha-N-acetylgalactosaminidase precursor (EC 3.2.1.49) (Alpha-, galactosidase B).]	Go Component not found
P17097	[Zinc finger protein 7 (Zinc finger protein KOX4) (Zinc finger protein, HF.16).]	Go Component not found
P17600	Synapsin-1 (Synapsin I) (Brain protein 4.1).	Go Component not found
P17707	[S-adenosylmethionine decarboxylase proenzyme (EC 4.1.1.50) (AdoMetDC), (SamDC) [Contains: S-adenosylmethionine decarboxylase alpha chain; S-, adenosylmethionine decarboxylase beta chain].]	Go Component not found
P17947	Transcription factor PU.1 (31 kDa-transforming protein).	Go Component not found
P18031	[Tyrosine-protein phosphatase non-receptor type 1 (EC 3.1.3.48), (Protein-tyrosine phosphatase 1B) (PTP-1B).]	Go Component not found
P18583	[SON protein (SON3) (Negative regulatory element-binding protein) (NRE-, binding protein) (DBP-5) (Bax antagonist selected in saccharomyces 1), (BASS1).]	Go Component not found
P18847	[Cyclic AMP-dependent transcription factor ATF-3 (Activating, transcription factor 3).]	Go Component not found
P18887	DNA-repair protein XRCC1 (X-ray repair cross-complementing protein 1).	Go Component not found
P19525	[Interferon-induced, double-stranded RNA-activated protein kinase, (EC 2.7.11.1) (Interferon-inducible RNA-dependent protein kinase), (Eukaryotic translation initiation factor 2-alpha kinase 2) (eIF-2A, protein kinase 2) (Protein kinase RNA-activated) (PKR) (p68 kinase), (P1/eIF-2A protein kinase).]	Go Component not found
P19878	[Neutrophil cytosol factor 2 (NCF-2) (Neutrophil NADPH oxidase factor, 2) (67 kDa neutrophil oxidase factor) (p67-phox) (NOXA2).]	Go Component not found

P20132	L-serine dehydratase (EC 4.3.1.17) (L-serine deaminase).	Go Component not found
P20273	[B-cell receptor CD22 precursor (Sialic acid-binding Ig-like lectin 2), (Siglec-2) (Leu-14) (B-lymphocyte cell adhesion molecule) (BL-CAM).]	Go Component not found
P20848	Alpha-1-antitrypsin-related protein precursor.	Go Component not found
P42331	Rho GTPase-activating protein 25.	Go Component not found
P42658	[Dipeptidyl aminopeptidase-like protein 6 (Dipeptidylpeptidase VI), (Dipeptidylpeptidase 6) (Dipeptidyl peptidase IV-like protein), (Dipeptidyl aminopeptidase-related protein) (DPPX).]	Go Component not found
P42680	Tyrosine-protein kinase Tec (EC 2.7.10.2).	Go Component not found
P42694	Probable helicase with zinc finger domain (EC 3.6.1.-).	Go Component not found
P43026	[Growth/differentiation factor 5 precursor (GDF-5) (Cartilage-derived, morphogenetic protein 1) (CDMP-1) (Radotermin).]	Go Component not found
P43235	[Cathepsin K precursor (EC 3.4.22.38) (Cathepsin O) (Cathepsin X), (Cathepsin O2).]	Go Component not found
P43251	Biotinidase precursor (EC 3.5.1.12).	Go Component not found
P43357	[Melanoma-associated antigen 3 (MAGE-3 antigen) (Antigen MZ2-D), (Cancer/testis antigen 1.3) (CT1.3).]	Go Component not found
P43362	[Melanoma-associated antigen 9 (MAGE-9 antigen) (Cancer/testis antigen, 1.9) (CT1.9).]	Go Component not found
P46013	Antigen KI-67.	Go Component not found
P46109	Crk-like protein.	Go Component not found
P46459	[Vesicle-fusing ATPase (EC 3.6.4.6) (Vesicular-fusion protein NSF) (N-, ethylmaleimide sensitive fusion protein) (NEM-sensitive fusion, protein).]	Go Component not found
P47895	[Aldehyde dehydrogenase 1A3 (EC 1.2.1.5) (Aldehyde dehydrogenase 6), (Retinaldehyde dehydrogenase 3) (RALDH-3).]	Go Component not found
P47974	[Butyrate response factor 2 (Protein TIS11D) (EGF-response factor 2), (ERF-2).]	Go Component not found
P47992	[Lymphotactin precursor (XCL1) (Cytokine SCM-1) (ATAC) (Lymphotaxin), (SCM-1-alpha) (Small-inducible cytokine C1) (XC chemokine ligand 1).]	Go Component not found
P48378	DNA-binding protein RFX2.	Go Component not found
P48637	[Glutathione synthetase (EC 6.3.2.3) (Glutathione synthase) (GSH, synthetase) (GSH-S).]	Go Component not found
P48736	[Phosphatidylinositol-4,5-bisphosphate 3-kinase catalytic subunit gamma, isoform (EC 2.7.1.153) (PI3-kinase p110 subunit gamma) (PtdIns-3-, kinase subunit p110) (PI3K) (PI3Kgamma) (p120-PI3K).]	Go Component not found
P49116	Orphan nuclear receptor TR4 (Orphan nuclear receptor TAK1).	Go Component not found
P49221	[Protein-glutamine gamma-glutamyltransferase 4 (EC 2.3.2.13), (Transglutaminase-4) (TGase-4) (Prostate transglutaminase) (TGP), (TG(P)) (Prostate-specific transglutaminase) (Fibrinoligase).]	Go Component not found
P49459	[Ubiquitin-conjugating enzyme E2 A (EC 6.3.2.19) (Ubiquitin-protein, ligase A) (Ubiquitin carrier protein A) (HR6A) (hHR6A).]	Go Component not found
P49593	[Protein phosphatase 1F (EC 3.1.3.16) (Ca(2+)/calmodulin-dependent, protein kinase phosphatase) (CaM-kinase phosphatase) (CaMKPase), (Partner of PIX 2) (hFEM-2).]	Go Component not found
P49675	[Steroidogenic acute regulatory protein, mitochondrial precursor (StAR), (StARD1).]	Go Component not found
P49750	YLP motif-containing protein 1 (Nuclear protein ZAP3) (ZAP113).	Go Component not found
P49758	Regulator of G-protein signaling 6 (RGS6) (S914).	Go Component not found
P49761	[Dual specificity protein kinase CLK3 (EC 2.7.12.1) (CDC-like kinase, 3).]	Go Component not found
P49798	Regulator of G-protein signaling 4 (RGS4) (RGP4).	Go Component not found
P49862	[Kallikrein-7 precursor (EC 3.4.21.117) (hK7) (Stratum corneum, chymotryptic enzyme) (hSCCE) (Serine protease 6).]	Go Component not found
P49902	[Cytosolic purine 5'-nucleotidase (EC 3.1.3.5) (5'-nucleotidase, cytosolic II).]	Go Component not found

P49917	[DNA ligase 4 (EC 6.5.1.1) (DNA ligase IV) (Polydeoxyribonucleotide, synthase [ATP] 4).]	Go Component not found
P50120	Retinol-binding protein II, cellular (CRBP-II).	Go Component not found
P50213	[Isocitrate dehydrogenase [NAD] subunit alpha, mitochondrial precursor, (EC 1.1.1.41) (Isocitric dehydrogenase) (NAD(+)-specific ICDH).]	Go Component not found
P50851	[Lipopolysaccharide-responsive and beige-like anchor protein (CDC4-like, protein) (Beige-like protein).]	Go Component not found
P51157	Ras-related protein Rab-28 (Rab-26).	Go Component not found
P51449	[Nuclear receptor ROR-gamma (Retinoid-related orphan receptor-gamma), (Nuclear receptor RZR-gamma).]	Go Component not found
P51530	[DNA2-like helicase (EC 3.6.1.-) (DNA replication ATP-dependent, helicase-like homolog).]	Go Component not found
P51648	[Fatty aldehyde dehydrogenase (EC 1.2.1.3) (Aldehyde dehydrogenase,, microsomal) (Aldehyde dehydrogenase family 3 member A2) (Aldehyde, dehydrogenase 10).]	Go Component not found
P51817	[Serine/threonine-protein kinase PRKX (EC 2.7.11.1) (Protein kinase, PKX1).]	Go Component not found
P51878	[Caspase-5 precursor (EC 3.4.22.58) (CASP-5) (ICH-3 protease) (TY, protease) (ICE(rel)-III) [Contains: Caspase-5 subunit p20; Caspase-5, subunit p10].]	Go Component not found
P51948	[CDK-activating kinase assembly factor MAT1 (RING finger protein MAT1), (Menage a trois) (CDK7/cyclin H assembly factor) (p36) (p35) (Cyclin, G1-interacting protein) (RING finger protein 66).]	Go Component not found
P52306	[Rap1 GTPase-GDP dissociation stimulator 1 (SMG P21 stimulatory GDP/GTP, exchange protein) (SMG GDS protein) (Exchange factor smgGDS).]	Go Component not found
P52564	[Dual specificity mitogen-activated protein kinase kinase 6, (EC 2.7.12.2) (MAP kinase kinase 6) (MAPKK 6) (MAPK/ERK kinase 6), (SAPKK3).]	Go Component not found
P52737	Zinc finger protein 136.	Go Component not found
P52743	Zinc finger protein 137.	Go Component not found
P52848	[Bifunctional heparan sulfate N-deacetylase/N-sulfotransferase 1, (EC 2.8.2.8) (Glucosaminyl N-deacetylase/N-sulfotransferase 1) (NDST-, 1) ([Heparan sulfate]-glucosamine N-sulfotransferase 1) (HSNST 1) (N-, heparan sulfate sulfotransferase 1) (N-HSST 1) [Includes: Heparan, sulfate N-deacetylase 1 (EC 3.-.-.-); Heparan sulfate N-, sulfotransferase 1 (EC 2.8.2.-)].]	Go Component not found
P53611	[Geranylgeranyl transferase type-2 subunit beta (EC 2.5.1.60), (Geranylgeranyl transferase type II subunit beta) (Rab, geranylgeranyltransferase subunit beta) (Rab geranyl-, geranyltransferase subunit beta) (Rab GG transferase beta) (Rab GGTase, beta).]	Go Component not found
P54296	[Myomesin-2 (M-protein) (165 kDa titin-associated protein) (165 kDa, connectin-associated protein).]	Go Component not found
P54619	[5'-AMP-activated protein kinase subunit gamma-1 (AMPK gamma-1 chain), (AMPKg).]	Go Component not found
P54687	[Branched-chain-amino-acid aminotransferase, cytosolic (EC 2.6.1.42), (BCAT(c)) (Protein ECA39).]	Go Component not found
P54922	[[Protein ADP-ribosylarginine] hydrolase (EC 3.2.2.19) (ADP-, ribosylarginine hydrolase) (ADP-ribose-L-arginine cleaving enzyme).]	Go Component not found
P55040	[GTP-binding protein GEM (GTP-binding mitogen-induced T-cell protein), (RAS-like protein KIR).]	Go Component not found
P55212	[Caspase-6 precursor (EC 3.4.22.59) (CASP-6) (Apoptotic protease Mch-2), [Contains: Caspase-6 subunit p18; Caspase-6 subunit p11].]	Go Component not found
P55283	Cadherin-4 precursor (Retinal cadherin) (R-cadherin) (R-CAD).	Go Component not found
P55774	[Small-inducible cytokine A18 precursor (CCL18) (Macrophage, inflammatory protein 4) (MIP-4) (Pulmonary and activation-regulated, chemokine) (CC chemokine PARC) (Alternative macrophage activation-, associated CC chemokine 1) (AMAC-1) (Dendritic cell chemokine 1) (DC-, CK1) [Contains: CCL18(1-68); CCL18(3-69); CCL18(4-69)].]	Go Component not found
P55899	[IgG receptor FcRn large subunit p51 precursor (FcRn) (Neonatal Fc, receptor) (IgG Fc fragment receptor transporter alpha chain).]	Go Component not found

P56202	Cathepsin W precursor (EC 3.4.22.-) (Lymphopain).	Go Component not found
P56282	[DNA polymerase epsilon subunit 2 (EC 2.7.7.7) (DNA polymerase II, subunit 2) (DNA polymerase epsilon subunit B).]	Go Component not found
P56545	C-terminal-binding protein 2 (CtBP2).	Go Component not found
P56597	[Nucleoside diphosphate kinase homolog 5 (NDK-H 5) (NDP kinase homolog, 5) (nm23-H5) (Testis-specific nm23 homolog) (Inhibitor of p53-induced, apoptosis-beta) (IPIA-beta).]	Go Component not found
P56696	[Potassium voltage-gated channel subfamily KQT member 4 (Voltage-gated, potassium channel subunit Kv7.4) (Potassium channel subunit alpha, KvLQT4) (KQT-like 4).]	Go Component not found
P56715	[Oxygen-regulated protein 1 (Retinitis pigmentosa RP1 protein), (Retinitis pigmentosa 1 protein).]	Go Component not found
P57052	Putative RNA-binding protein 11 (RNA-binding motif protein 11).	Go Component not found
P57058	[Hormonally up-regulated neu tumor-associated kinase (EC 2.7.11.1), (Serine/threonine-protein kinase MAK-V) (B19).]	Go Component not found
P57103	[Sodium/calcium exchanger 3 precursor (Na(+)/Ca(2+)-exchange protein, 3).]	Go Component not found
P57723	Poly(rC)-binding protein 4 (Alpha-CP4).	Go Component not found
P57738	[T-cell leukemia translocation-altered gene protein (T-cell leukemia, translocation-associated gene protein).]	Go Component not found
P57789	[Potassium channel subfamily K member 10 (Outward rectifying potassium, channel protein TREK-2) (TREK-2 K(+) channel subunit).]	Go Component not found
Q13405	[Mitochondrial 39S ribosomal protein L49 (L49mt) (MRP-L49) (Protein, NOF1) (Neighbor of FAU) (NOF).]	Go Component not found
Q13434	[Makorin-4 (Zinc finger protein 127-Xp) (ZNF127-Xp) (RING finger, protein 64).]	Go Component not found
Q13451	[FK506-binding protein 5 (EC 5.2.1.8) (Peptidyl-prolyl cis-trans, isomerase) (PPIase) (Rotamase) (51 kDa FK506-binding protein) (FKBP-, 51) (54 kDa progesterone receptor-associated immunophilin) (FKBP54), (P54) (FF1 antigen) (HSP90-binding immunophilin) (Androgen-regulated, protein 6).]	Go Component not found
Q13474	Dystrophin-related protein 2.	Go Component not found
Q13490	[Baculoviral IAP repeat-containing protein 2 (Inhibitor of apoptosis, protein 2) (HIAP2) (HIAP-2) (C-IAP1) (TNFR2-TRAF-signaling complex, protein 2) (IAP homolog B) (RING finger protein 48).]	Go Component not found
Q13516	[Oligodendrocyte transcription factor 2 (Oligo2) (Class B basic helix-, loop-helix protein 1) (bHLHB1) (Protein kinase C-binding protein, RACK17) (Protein kinase C-binding protein 2).]	Go Component not found
Q13618	Cullin-3 (CUL-3).	Go Component not found
Q13634	Cadherin-18 precursor (Cadherin-14).	Go Component not found
Q13753	[Laminin subunit gamma-2 precursor (Laminin 5 gamma 2 subunit), (Kalinin/nicein/epiligrin 100 kDa subunit) (Laminin B2t chain) (Cell-, scattering factor 140 kDa subunit) (CSF 140 kDa subunit) (Large, adhesive scatter factor 140 kDa subunit) (Ladsin 140 kDa subunit).]	Go Component not found
Q13886	[Krueppel-like factor 9 (Transcription factor BTEB1) (Basic, transcription element-binding protein 1) (BTE-binding protein 1) (GC-, box-binding protein 1).]	Go Component not found
Q14004	[Cell division cycle 2-like protein kinase 5 (EC 2.7.11.22) (CDC2-, related protein kinase 5) (Cholinesterase-related cell division, controller).]	Go Component not found
Q14012	[Calcium/calmodulin-dependent protein kinase type 1 (EC 2.7.11.17) (CaM, kinase I) (CaM-KI) (CaM kinase I alpha) (CaMKI-alpha).]	Go Component not found
Q14088	Ras-related protein Rab-33A (Small GTP-binding protein S10).	Go Component not found
Q14149	[MORC family CW-type zinc finger protein 3 (Zinc finger CW-type coiled-, coil domain protein 3).]	Go Component not found
Q14157	Ubiquitin-associated protein 2-like (Protein NICE-4).	Go Component not found
Q14165	Uncharacterized protein KIAA0152 precursor.	Go Component not found

Q14188	Transcription factor Dp-2 (E2F dimerization partner 2).	Go Component not found
Q14195	[Dihydropyrimidinase-related protein 3 (DRP-3) (Unc-33-like, phosphoprotein) (ULIP protein) (Collapsin response mediator protein 4), (CRMP-4).]	Go Component not found
Q14206	[Calcipressin-2 (Regulator of calcineurin 2) (Down syndrome candidate, region 1-like 1) (Thyroid hormone-responsive protein ZAKI-4) (Myocyte-, enriched calcineurin-interacting protein 2) (MCIP2).]	Go Component not found
Q14249	Endonuclease G, mitochondrial precursor (EC 3.1.30.-) (Endo G).	Go Component not found
Q14353	Guanidinoacetate N-methyltransferase (EC 2.1.1.2).	Go Component not found
Q14449	Growth factor receptor-bound protein 14 (GRB14 adapter protein).	Go Component not found
Q14584	Zinc finger protein 266 (Zinc finger protein 1) (HZF1).	Go Component not found
Q14588	Zinc finger protein 234 (Zinc finger protein 4) (HZF4).	Go Component not found
Q14651	Plastin-1 (I-plastin) (Intestine-specific plastin).	Go Component not found
Q14681	BTB/POZ domain-containing protein KCTD2.	Go Component not found
Q14694	[Ubiquitin carboxyl-terminal hydrolase 10 (EC 3.1.2.15) (Ubiquitin, thioesterase 10) (Ubiquitin-specific-processing protease 10), (Deubiquitinating enzyme 10).]	Go Component not found
Q14765	Signal transducer and activator of transcription 4.	Go Component not found
Q14814	Myocyte-specific enhancer factor 2D.	Go Component not found
Q14839	[Chromodomain-helicase-DNA-binding protein 4 (EC 3.6.1.-) (ATP-, dependent helicase CHD4) (CHD-4) (Mi-2 autoantigen 218 kDa protein), (Mi2-beta).]	Go Component not found
Q14938	[Nuclear factor 1 X-type (Nuclear factor 1/X) (NF1-X) (NFI-X) (NF-I/X), (CCAAT-box-binding transcription factor) (CTF) (TGGCA-binding, protein).]	Go Component not found
Q15032	R3H domain-containing protein 1.	Go Component not found
Q15051	IQ calmodulin-binding motif-containing protein 1 (Nephrocystin-5).	Go Component not found
Q15124	[Phosphoglucomutase-like protein 5 (Phosphoglucomutase-related protein), (PGM-RP) (Aciculin).]	Go Component not found
Q15165	[Serum paraoxonase/arylesterase 2 (EC 3.1.1.2) (EC 3.1.8.1) (PON 2), (Serum aryldialkylphosphatase 2) (A-esterase 2) (Aromatic esterase 2).]	Go Component not found
Q15283	Ras GTPase-activating protein 2 (GAP1m).	Go Component not found
Q15418	[Ribosomal protein S6 kinase alpha-1 (EC 2.7.11.1) (S6K-alpha 1) (90, kDa ribosomal protein S6 kinase 1) (p90-RSK 1) (Ribosomal S6 kinase 1), (RSK-1) (pp90RSK1) (p90S6K) (MAP kinase-activated protein kinase 1a), (MAPKAPK1A).]	Go Component not found
Q15477	Helicase SKI2W (EC 3.6.1.-) (Helicase-like protein) (HLP).	Go Component not found
Q15506	[Sperm surface protein Sp17 (Sperm autoantigenic protein 17) (Sperm, protein 17) (Sp17-1) (Cancer/testis antigen 22) (CT22).]	Go Component not found
Q15643	[Thyroid receptor-interacting protein 11 (TRIP-11) (Golgi-associated, microtubule-binding protein 210) (GMAP-210) (Trip230) (Clonal, evolution-related gene on chromosome 14).]	Go Component not found
Q15678	[Tyrosine-protein phosphatase non-receptor type 14 (EC 3.1.3.48), (Protein-tyrosine phosphatase pez).]	Go Component not found
Q15697	[Zinc finger protein 174 (AW-1) (Zinc finger and SCAN domain-containing, protein 8).]	Go Component not found
Q15714	[TSC22 domain family protein 1 (Transforming growth factor beta-1-, induced transcript 4 protein) (Regulatory protein TSC-22) (TGFB-, stimulated clone 22 homolog) (Cerebral protein 2).]	Go Component not found
Q15746	[Myosin light chain kinase, smooth muscle (EC 2.7.11.18) (MLCK), (Telokin) (Kinase-related protein) (KRP).]	Go Component not found
Q15811	Intersectin-1 (SH3 domain-containing protein 1A) (SH3P17).	Go Component not found
Q15833	Syntaxin-binding protein 2 (Unc-18 homolog 2) (Unc-18B) (Unc18-2).	Go Component not found

Q15973	Zinc finger protein 124 (HZF-16).	Go Component not found
Q16254	Transcription factor E2F4 (E2F-4).	Go Component not found
Q16533	[snRNA-activating protein complex subunit 1 (SNAPc subunit 1) (snRNA-, activating protein complex 43 kDa subunit) (SNAPc 43 kDa subunit), (Small nuclear RNA-activating complex polypeptide 1) (Proximal, sequence element-binding transcription factor subunit gamma) (PSE-, binding factor subunit gamma) (PTF subunit gamma).]	Go Component not found
Q16589	Cyclin-G2.	Go Component not found
Q16659	[Mitogen-activated protein kinase 6 (EC 2.7.11.24) (Extracellular, signal-regulated kinase 3) (ERK-3) (MAP kinase isoform p97) (p97-, MAPK).]	Go Component not found
Q16671	[Anti-Muellerian hormone type-2 receptor precursor (EC 2.7.11.30), (Anti-Muellerian hormone type II receptor) (AMH type II receptor) (MIS, type II receptor) (MISRII) (MRII).]	Go Component not found
Q16690	[Dual specificity protein phosphatase 5 (EC 3.1.3.48) (EC 3.1.3.16), (Dual specificity protein phosphatase hVH3).]	Go Component not found
Q16719	Kynureninase (EC 3.7.1.3) (L-kynurenine hydrolase).	Go Component not found
Q16769	[Glutaminyl-peptide cyclotransferase precursor (EC 2.3.2.5) (QC), (Glutaminyl-tRNA cyclotransferase) (Glutaminyl cyclase) (Glutamyl, cyclase) (EC).]	Go Component not found
Q29865	[HLA class I histocompatibility antigen, Cw-18 alpha chain precursor, (MHC class I antigen Cw*18).]	Go Component not found
Q6S8J3	Prostate, ovary, testis-expressed protein on chromosome 2 (Fragment).	Go Component not found
Q86U42	[Polyadenylate-binding protein 2 (Poly(A)-binding protein 2) (Poly(A)-, binding protein II) (PABII) (Polyadenylate-binding nuclear protein 1), (Nuclear poly(A)-binding protein 1).]	Go Component not found
Q8TBG9	Synaptoporin.	Go Component not found
Q92539	Lipin-2.	Go Component not found
Q92544	Transmembrane 9 superfamily protein member 4.	Go Component not found
Q92561	[Phytanoyl-CoA hydroxylase-interacting protein (Phytanoyl-CoA, hydroxylase-associated protein 1) (PAHXAP1) (PAHX-AP1).]	Go Component not found
Q92599	Septin-8.	Go Component not found
Q92610	Zinc finger protein 592.	Go Component not found
Q92628	Uncharacterized protein KIAA0232.	Go Component not found
Q92667	[A kinase anchor protein 1, mitochondrial precursor (Protein kinase A-, anchoring protein 1) (PRKA1) (A-kinase anchor protein 149 kDa) (AKAP, 149) (Dual specificity A-kinase-anchoring protein 1) (D-AKAP-1), (Spermatid A-kinase anchor protein 84) (S-AKAP84).]	Go Component not found
Q92696	[Geranylgeranyl transferase type-2 subunit alpha (EC 2.5.1.60), (Geranylgeranyl transferase type II subunit alpha) (Rab, geranylgeranyltransferase subunit alpha) (Rab geranyl-, geranyltransferase subunit alpha) (Rab GG transferase alpha) (Rab, GGTase alpha).]	Go Component not found
Q92733	[Proline-rich protein PRCC (Papillary renal cell carcinoma, translocation-associated gene protein).]	Go Component not found
Q92754	[Transcription factor AP-2 gamma (AP2-gamma) (Activating enhancer-, binding protein 2 gamma) (Transcription factor ERF-1).]	Go Component not found
Q92782	[Zinc finger protein neuro-d4 (D4, zinc and double PHD fingers family, 1).]	Go Component not found
Q92800	Enhancer of zeste homolog 1 (ENX-2).	Go Component not found
Q92820	[Gamma-glutamyl hydrolase precursor (EC 3.4.19.9) (Gamma-Glu-X, carboxypeptidase) (Conjugase) (GH).]	Go Component not found
Q92830	[General control of amino acid synthesis protein 5-like 2 (EC 2.3.1.48), (Histone acetyltransferase GCN5) (hsGCN5) (STAF97).]	Go Component not found
Q92854	[Semaphorin-4D precursor (Leukocyte activation antigen CD100) (BB18), (A8) (GR3).]	Go Component not found
Q92914	[Fibroblast growth factor 11 (FGF-11) (Fibroblast growth factor, homologous factor 3) (FHF-3).]	Go Component not found

O75949	Transmembrane protein 28 (TED protein).	Go Component not found
O75969	[A-kinase anchor protein 3 (Protein kinase A-anchoring protein 3), (PRKA3) (A-kinase anchor protein 110 kDa) (AKAP 110) (Sperm oocyte-, binding protein) (Fibrousheathin-1) (Fibrousheathin I) (Fibrous sheath, protein of 95 kDa) (FSP95) (Cancer/testis antigen 82) (CT82).]	Go Component not found
O76041	Nebulette (Actin-binding Z-disk protein).	Go Component not found
O76094	Signal recognition particle 72 kDa protein (SRP72).	Go Component not found
O94788	[Retinal dehydrogenase 2 (EC 1.2.1.36) (RalDH2) (RALDH 2) (RALDH(II)), (Retinaldehyde-specific dehydrogenase type 2) (Aldehyde dehydrogenase, family 1 member A2).]	Go Component not found
O94810	Regulator of G-protein signaling 11 (RGS11).	Go Component not found
O94823	Probable phospholipid-transporting ATPase VB (EC 3.6.3.1).	Go Component not found
O94876	Transmembrane and coiled-coil domains protein 1.	Go Component not found
O94956	[Solute carrier organic anion transporter family member 2B1 (Solute, carrier family 21 member 9) (Organic anion transporter B) (OATP-B), (Organic anion transporter polypeptide-related protein 2) (OATP-RP2), (OATPRP2).]	Go Component not found
O95007	Olfactory receptor 6B1 (Olfactory receptor 7-3) (OR7-3).	Go Component not found
O95049	[Tight junction protein ZO-3 (Zonula occludens protein 3) (Zona, occludens protein 3) (Tight junction protein 3).]	Go Component not found
O95125	[Zinc finger protein 202 (Zinc finger protein with KRAB and SCAN, domains 10).]	Go Component not found
O95359	[Transforming acidic coiled-coil-containing protein 2 (Anti Zuai-1), (AZU-1).]	Go Component not found
O95393	Bone morphogenetic protein 10 precursor (BMP-10).	Go Component not found
O95479	[GDH/6PGL endoplasmic bifunctional protein precursor [Includes: Glucose, 1-dehydrogenase (EC 1.1.1.47) (Hexose-6-phosphate dehydrogenase); 6-, phosphogluconolactonase (EC 3.1.1.31) (6PGL)].]	Go Component not found
O95521	PRAME family member 1.	Go Component not found
O95716	Ras-related protein Rab-3D.	Go Component not found
O95897	Noelin-2 precursor (Olfactomedin-2).	Go Component not found
O95936	Eomesodermin homolog.	Go Component not found
O95988	[T-cell leukemia/lymphoma protein 1B (Oncogene TCL1B), (Syncytiotrophoblast-specific protein) (SYN-1).]	Go Component not found
O96014	Protein Wnt-11 precursor.	Go Component not found
P25440	Bromodomain-containing protein 2 (Protein RING3) (O27.1.1).	Go Component not found
P00439	[Phenylalanine-4-hydroxylase (EC 1.14.16.1) (PAH) (Phe-4-, monooxygenase).]	Go Component not found
P00540	[Proto-oncogene serine/threonine-protein kinase mos (EC 2.7.11.1) (c-, mos) (Oocyte maturation factor mos).]	Go Component not found
P00751	[Complement factor B precursor (EC 3.4.21.47) (C3/C5 convertase), (Properdin factor B) (Glycine-rich beta glycoprotein) (GBG) (PBF2), [Contains: Complement factor B Ba fragment; Complement factor B Bb, fragment].]	Go Component not found
P01569	[Interferon alpha-5 precursor (Interferon alpha-G) (LeIF G) (Interferon, alpha-61).]	Go Component not found
P01579	Interferon gamma precursor (IFN-gamma) (Immune interferon).	Go Component not found
P01916	HLA class II histocompatibility antigen, SB beta chain (Fragment).	Go Component not found
P02458	[Collagen alpha-1(II) chain precursor (Alpha-1 type II collagen), [Contains: Chondrocalcin].]	Go Component not found
P02689	Myelin P2 protein.	Go Component not found
P02771	[Alpha-fetoprotein precursor (Alpha-fetoglobulin) (Alpha-1-, fetoprotein).]	Go Component not found
P04040	Catalase (EC 1.11.1.6).	Go Component not found

P04062	[Glucosylceramidase precursor (EC 3.2.1.45) (Beta-glucocerebrosidase), (Acid beta-glucosidase) (D-glucosyl-N-acylsphingosine glucohydrolase), (Alglucerase) (Imiglucerase).]	Go Component not found
P04075	[Fructose-bisphosphate aldolase A (EC 4.1.2.13) (Muscle-type aldolase), (Lung cancer antigen NY-LU-1).]	Go Component not found
P04085	[Platelet-derived growth factor A chain precursor (PDGF A-chain), (Platelet-derived growth factor alpha polypeptide) (PDGF-1).]	Go Component not found
P04350	Tubulin beta-4 chain (Tubulin 5 beta).	Go Component not found
P04798	[Cytochrome P450 1A1 (EC 1.14.14.1) (CYPIA1) (P450-P1) (P450 form 6), (P450-C).]	Go Component not found
P04899	[Guanine nucleotide-binding protein G(i), alpha-2 subunit (Adenylate, cyclase-inhibiting G alpha protein).]	Go Component not found
P05019	[Insulin-like growth factor IB precursor (IGF-IB) (Somatomedin-C), (Mechano growth factor) (MGF).]	Go Component not found
P05120	[Plasminogen activator inhibitor 2 precursor (PAI-2) (Placental, plasminogen activator inhibitor) (Monocyte Arg-serpin) (Urokinase, inhibitor).]	Go Component not found
P05162	[Galectin-2 (Beta-galactoside-binding lectin L-14-II) (Lactose-binding, lectin 2) (S-Lac lectin 2) (HL14).]	Go Component not found
P05181	Cytochrome P450 2E1 (EC 1.14.14.1) (CYPIIE1) (P450-J).	Go Component not found
P05534	[HLA class I histocompatibility antigen, A-24 alpha chain precursor, (MHC class I antigen A*24) (Aw-24) (A-9).]	Go Component not found
P05937	[Calbindin (Vitamin D-dependent calcium-binding protein, avian-type), (Calbindin D28) (D-28K).]	Go Component not found
P06737	Glycogen phosphorylase, liver form (EC 2.4.1.1).	Go Component not found
P07315	[Gamma-crystallin C (Gamma-C-crystallin) (Gamma-crystallin 2-1) (Gamma-, crystallin 3).]	Go Component not found
P07360	Complement component C8 gamma chain precursor.	Go Component not found
P07451	[Carbonic anhydrase 3 (EC 4.2.1.1) (Carbonic anhydrase III) (Carbonate, dehydratase III) (CA-III).]	Go Component not found
P07737	Profilin-1 (Profilin I).	Go Component not found
P21506	Zinc finger protein 10 (Zinc finger protein KOX1).	Go Component not found
P22102	[Trifunctional purine biosynthetic protein adenosine-3 [Includes:, Phosphoribosylamine--glycine ligase (EC 6.3.4.13) (GARS) (Glycinamide, ribonucleotide synthetase) (Phosphoribosylglycinamide synthetase);, Phosphoribosylformylglycinamidine cyclo-ligase (EC 6.3.3.1) (AIRS), (Phosphoribosyl-aminoimidazole synthetase) (AIR synthase);, Phosphoribosylglycinamide formyltransferase (EC 2.1.2.2) (GART) (GAR, transformylase) (5'-phosphoribosylglycinamide transformylase)].]	Go Component not found
P22612	[cAMP-dependent protein kinase, gamma-catalytic subunit (EC 2.7.11.11), (PKA C-gamma).]	Go Component not found
P22670	MHC class II regulatory factor RFX1 (RFX) (Enhancer factor C) (EF-C).	Go Component not found
P22736	[Orphan nuclear receptor NR4A1 (Orphan nuclear receptor HMR) (Early, response protein NAK1) (TR3 orphan receptor) (ST-59).]	Go Component not found
P23109	[AMP deaminase 1 (EC 3.5.4.6) (Myoadenylate deaminase) (AMP deaminase, isoform M).]	Go Component not found
P23193	[Transcription elongation factor A protein 1 (Transcription elongation, factor S-II protein 1) (Transcription elongation factor TFIIS.o).]	Go Component not found
P23443	[Ribosomal protein S6 kinase beta-1 (EC 2.7.11.1) (Ribosomal protein S6, kinase I) (S6K) (S6K1) (70 kDa ribosomal protein S6 kinase 1) (p70 S6, kinase alpha) (p70(S6K)-alpha) (p70-S6K) (P70S6K) (p70-alpha).]	Go Component not found
P23469	[Receptor-type tyrosine-protein phosphatase epsilon precursor, (EC 3.1.3.48) (Protein-tyrosine phosphatase epsilon) (R-PTP-epsilon).]	Go Component not found
P23760	Paired box protein Pax-3 (HUP2).	Go Component not found
P23919	Thymidylate kinase (EC 2.7.4.9) (dTMP kinase).	Go Component not found
P24278	[Zinc finger and BTB domain-containing protein 25 (Zinc finger protein, 46) (Zinc finger protein KUP).]	Go Component not found
P80192	[Mitogen-activated protein kinase kinase kinase 9 (EC 2.7.11.25) (Mixed, lineage kinase 1).]	Go Component not found
P26045	[Tyrosine-protein phosphatase non-receptor type 3 (EC 3.1.3.48), (Protein-tyrosine phosphatase H1) (PTP-H1).]	Go Component not found

P26951	[Interleukin-3 receptor alpha chain precursor (IL-3R-alpha) (CD123, antigen).]	Go Component not found
P27448	[MAP/microtubule affinity-regulating kinase 3 (EC 2.7.11.1) (Cdc25C-, associated protein kinase 1) (cTAK1) (C-TAK1) (Serine/threonine, protein kinase p78) (Ser/Thr protein kinase PAR-1) (Protein kinase, STK10).]	Go Component not found
P28039	[Acyloxyacyl hydrolase precursor (EC 3.1.1.77) [Contains: Acyloxyacyl, hydrolase small subunit; Acyloxyacyl hydrolase large subunit].]	Go Component not found
P28069	[Pituitary-specific positive transcription factor 1 (Pit-1) (Growth, hormone factor 1) (GHF-1).]	Go Component not found
P28328	[Peroxisome assembly factor 1 (PAF-1) (Peroxin-2) (Peroxisomal membrane, protein 3) (35 kDa peroxisomal membrane protein) (RING finger protein, 72).]	Go Component not found
P28335	[5-hydroxytryptamine receptor 2C (5-HT-2C) (Serotonin receptor 2C) (5-, HT2C) (5-HTR2C) (5HT-1C).]	Go Component not found
P28347	[Transcriptional enhancer factor TEF-1 (TEA domain family member 1), (TEAD-1) (Protein GT-IIC) (Transcription factor 13) (NTEF-1).]	Go Component not found
P28749	[Retinoblastoma-like protein 1 (107 kDa retinoblastoma-associated, protein) (p107) (PRB1).]	Go Component not found
P28845	[Corticosteroid 11-beta-dehydrogenase isozyme 1 (EC 1.1.1.146) (11-DH), (11-beta-hydroxysteroid dehydrogenase 1) (11-beta-HSD1).]	Go Component not found
P29084	[Transcription initiation factor IIE subunit beta (TFIIE-beta) (General, transcription factor IIE subunit 2).]	Go Component not found
P29401	Transketolase (EC 2.2.1.1) (TK).	Go Component not found
P29466	[Caspase-1 precursor (EC 3.4.22.36) (CASP-1) (Interleukin-1 beta, convertase) (IL-1BC) (IL-1 beta-converting enzyme) (ICE) (Interleukin-, 1 beta-converting enzyme) (p45) [Contains: Caspase-1 subunit p20;, Caspase-1 subunit p10].]	Go Component not found
P29597	Non-receptor tyrosine-protein kinase TYK2 (EC 2.7.10.2).	Go Component not found
P29762	[Cellular retinoic acid-binding protein 1 (Cellular retinoic acid-, binding protein I) (CRABP-I) (Retinoic acid-binding protein I,, cellular).]	Go Component not found
P30086	[Phosphatidylethanolamine-binding protein 1 (PEBP-1) (Prostatic-binding, protein) (HCNPpp) (Neuropolypeptide h3) (Raf kinase inhibitor protein), (RKIP) [Contains: Hippocampal cholinergic neurostimulating peptide, (HCNP)].]	Go Component not found
P30419	[Glycylpeptide N-tetradecanoyltransferase 1 (EC 2.3.1.97) (Peptide N-, myristoyltransferase 1) (Myristoyl-CoA:protein N-myristoyltransferase, 1) (NMT 1) (Type I N-myristoyltransferase).]	Go Component not found
P30464	[HLA class I histocompatibility antigen, B-15 alpha chain precursor, (MHC class I antigen B*15).]	Go Component not found
P30613	[Pyruvate kinase isozymes R/L (EC 2.7.1.40) (R-type/L-type pyruvate, kinase) (Red cell/liver pyruvate kinase) (Pyruvate kinase 1).]	Go Component not found
P30711	[Glutathione S-transferase theta-1 (EC 2.5.1.18) (GST class-theta-1), (Glutathione transferase T1-1).]	Go Component not found
P31274	Homeobox protein Hox-C9 (Hox-3B).	Go Component not found
P31944	[Caspase-14 precursor (EC 3.4.22.-) (CASP-14) [Contains: Caspase-14, subunit 1; Caspase-14 subunit 2].]	Go Component not found
P32314	Forkhead box protein N2 (Human T-cell leukemia virus enhancer factor).	Go Component not found
P32926	Desmoglein-3 precursor (130 kDa pemphigus vulgaris antigen) (PVA).	Go Component not found
P33076	MHC class II transactivator (CIITA).	Go Component not found
P35913	[Rod cGMP-specific 3',5'-cyclic phosphodiesterase subunit beta, precursor (EC 3.1.4.35) (GMP-PDE beta).]	Go Component not found
P36941	[Tumor necrosis factor receptor superfamily member 3 precursor, (Lymphotoxin-beta receptor) (Tumor necrosis factor receptor 2-related, protein) (Tumor necrosis factor C receptor).]	Go Component not found
P36957	[Dihydrolipoyllysine-residue succinyltransferase component of 2-, oxoglutarate dehydrogenase complex, mitochondrial precursor, (EC 2.3.1.61) (Dihydrolipoamide succinyltransferase component of 2-, oxoglutarate dehydrogenase complex) (E2) (E2K).]	Go Component not found
P36980	[Complement factor H-related protein 2 precursor (FHR-2) (H factor-like, protein 2) (H factor-like 3) (DDESK59).]	Go Component not found
P37275	[Zinc finger E-box-binding homeobox 1 (Transcription factor 8) (NIL-2-A, zinc finger protein) (Negative regulator of IL2).]	Go Component not found

P39900	[Macrophage metalloelastase precursor (EC 3.4.24.65) (HME) (Matrix, metalloproteinase-12) (MMP-12) (Macrophage elastase) (ME).]	Go Component not found
P40939	[Trifunctional enzyme subunit alpha, mitochondrial precursor (TP-alpha), (78 kDa gastrin-binding protein) [Includes: Long-chain enoyl-CoA, hydratase (EC 4.2.1.17); Long chain 3-hydroxyacyl-CoA dehydrogenase, (EC 1.1.1.211)].]	Go Component not found
P41218	Myeloid cell nuclear differentiation antigen.	Go Component not found
P58173	[Olfactory receptor 2B6 (Olfactory receptor OR6-4) (Olfactory receptor, 6-31) (OR6-31) (Olfactory receptor 5-40) (OR5-40) (Hs6M1-32).]	Go Component not found
P58397	[ADAMTS-12 precursor (EC 3.4.24.-) (A disintegrin and metalloproteinase, with thrombospondin motifs 12) (ADAM-TS 12) (ADAM-TS12).]	Go Component not found
P61011	Signal recognition particle 54 kDa protein (SRP54).	Go Component not found
P61086	[Ubiquitin-conjugating enzyme E2-25 kDa (EC 6.3.2.19) (Ubiquitin-, protein ligase) (Ubiquitin carrier protein) (E2(25K)) (Huntingtin-, interacting protein 2) (HIP-2).]	Go Component not found
P61244	Protein max (Myc-associated factor X).	Go Component not found
P61513	60S ribosomal protein L37a.	Go Component not found
P62906	[60S ribosomal protein L10a (CSA-19) (Protein NEDD6) (Neural precursor, cell expressed developmentally down-regulated protein 6) (NEDD-6).]	Go Component not found
P63010	[AP-2 complex subunit beta-1 (Adapter-related protein complex 2 beta-1, subunit) (Beta-adaptin) (Plasma membrane adaptor HA2/AP2 adaptin beta, subunit) (Clathrin assembly protein complex 2 beta large chain), (AP105B).]	Go Component not found
P63208	[S-phase kinase-associated protein 1A (Cyclin A/CDK2-associated protein, p19) (p19A) (p19skp1) (RNA polymerase II elongation factor-like, protein) (Organ of Corti protein 2) (OCP-II protein) (OCP-2), (Transcription elongation factor B) (SIII).]	Go Component not found
P67812	[Signal peptidase complex catalytic subunit SEC11A (EC 3.4.-.-) (SEC11, homolog A) (SEC11-like protein 1) (Microsomal signal peptidase 18 kDa, subunit) (SPase 18 kDa subunit) (SPC18) (Endopeptidase SP18).]	Go Component not found
P69892	[Hemoglobin subunit gamma-2 (Hemoglobin gamma-2 chain) (Gamma-2-globin), (Hemoglobin gamma-G chain) (Hb F Ggamma).]	Go Component not found
P78314	SH3 domain-binding protein 2 (3BP-2).	Go Component not found
P78413	[Iroquois-class homeodomain protein IRX-4 (Iroquois homeobox protein 4), (Homeodomain protein IRXA3).]	Go Component not found
P78527	[DNA-dependent protein kinase catalytic subunit (EC 2.7.11.1) (DNA-PK, catalytic subunit) (DNA-PKcs) (DNPK1) (p460).]	Go Component not found
P78560	[Death domain-containing protein CRADD (Caspase and RIP adapter with, death domain) (RIP-associated protein with a death domain).]	Go Component not found
P80075	[Small-inducible cytokine A8 precursor (CCL8) (Monocyte chemotactic, protein 2) (MCP-2) (Monocyte chemoattractant protein 2) (HC14), [Contains: MCP-2(6-76)].]	Go Component not found
Q9BY50	[Signal peptidase complex catalytic subunit SEC11C (EC 3.4.-.-) (SEC11, homolog C) (SEC11-like protein 3) (Microsomal signal peptidase 21 kDa, subunit) (SPase 21 kDa subunit) (SPC21).]	Go Component not found
Q9BZI1	[Iroquois-class homeodomain protein IRX-2 (Iroquois homeobox protein 2), (Homeodomain protein IRXA2).]	Go Component not found
P83105	Probable serine protease HTRA4 precursor (EC 3.4.21.-).	Go Component not found
P84090	Enhancer of rudimentary homolog.	Go Component not found
P98066	[Tumor necrosis factor-inducible protein TSG-6 precursor (TNF-, stimulated gene 6 protein) (Tumor necrosis factor, alpha-induced, protein 6) (Hyaluronate-binding protein).]	Go Component not found
P98160	[Basement membrane-specific heparan sulfate proteoglycan core protein, precursor (HSPG) (Perlecan) (PLC).]	Go Component not found
P98196	[Probable phospholipid-transporting ATPase IH (EC 3.6.3.1) (ATPase, class I type 11A) (ATPase IS).]	Go Component not found
Q00526	Cell division protein kinase 3 (EC 2.7.11.22).	Go Component not found

Q00536	[Serine/threonine-protein kinase PCTAIRE-1 (EC 2.7.11.22) (PCTAIRE-, motif protein kinase 1).]	Go Component not found
Q00722	[1-phosphatidylinositol-4,5-bisphosphate phosphodiesterase beta-2, (EC 3.1.4.11) (Phosphoinositide phospholipase C) (Phospholipase C-, beta-2) (PLC-beta-2).]	Go Component not found
Q00887	[Pregnancy-specific beta-1-glycoprotein 9 precursor (PSBG-9), (Pregnancy-specific glycoprotein 9) (Pregnancy-specific beta-1, glycoprotein B) (PS-beta-B) (PS34) (Pregnancy-specific glycoprotein 7), (PSG7).]	Go Component not found
Q01064	[Calcium/calmodulin-dependent 3',5'-cyclic nucleotide phosphodiesterase, 1B (EC 3.1.4.17) (Cam-PDE 1B) (63 kDa Cam-PDE).]	Go Component not found
Q01118	[Sodium channel protein type 7 subunit alpha (Sodium channel protein, type VII subunit alpha) (Putative voltage-gated sodium channel alpha, subunit Nax) (Sodium channel protein cardiac and skeletal muscle, subunit alpha).]	Go Component not found
Q01433	AMP deaminase 2 (EC 3.5.4.6) (AMP deaminase isoform L).	Go Component not found
Q01524	Defensin-6 precursor (Defensin, alpha 6).	Go Component not found
Q01740	[Dimethylaniline monooxygenase [N-oxide-forming] 1 (EC 1.14.13.8), (Fetal hepatic flavin-containing monooxygenase 1) (FMO 1), (Dimethylaniline oxidase 1).]	Go Component not found
Q01851	[POU domain, class 4, transcription factor 1 (Brain-specific, homeobox/POU domain protein 3A) (Brn-3A) (Oct-T1) (Homeobox/POU domain, protein RDC-1).]	Go Component not found
Q02218	[2-oxoglutarate dehydrogenase E1 component, mitochondrial precursor, (EC 1.2.4.2) (Alpha-ketoglutarate dehydrogenase).]	Go Component not found
Q02575	[Helix-loop-helix protein 1 (HEN1) (Nescient helix loop helix 1) (NSCL-, 1).]	Go Component not found
Q02809	[Procollagen-lysine,2-oxoglutarate 5-dioxygenase 1 precursor, (EC 1.14.11.4) (Lysyl hydroxylase 1) (LH1).]	Go Component not found
Q03013	[Glutathione S-transferase Mu 4 (EC 2.5.1.18) (GSTM4-4) (GST class-mu, 4) (GTS-Mu2).]	Go Component not found
Q04446	[1,4-alpha-glucan-branching enzyme (EC 2.4.1.18) (Glycogen-branching, enzyme) (Brancher enzyme).]	Go Component not found
Q05193	Dynamin-1 (EC 3.6.5.5).	Go Component not found
Q05524	[Alpha-enolase, lung specific (EC 4.2.1.11) (2-phospho-D-glycerate, hydro-lyase) (Non-neural enolase) (NNE) (Phosphopyruvate hydratase), (HLE1).]	Go Component not found
Q06124	[Tyrosine-protein phosphatase non-receptor type 11 (EC 3.1.3.48), (Protein-tyrosine phosphatase 2C) (PTP-2C) (PTP-1D) (SH-PTP3) (SH-, PTP2) (SHP-2) (Shp2).]	Go Component not found
Q06203	[Amidophosphoribosyltransferase precursor (EC 2.4.2.14) (Glutamine, phosphoribosylpyrophosphate amidotransferase) (ATase) (GPAT).]	Go Component not found
Q06278	Aldehyde oxidase (EC 1.2.3.1).	Go Component not found
Q06945	Transcription factor SOX-4.	Go Component not found
Q07889	Son of sevenless homolog 1 (SOS-1).	Go Component not found
Q08623	[Haloacid dehalogenase-like hydrolase domain-containing protein 1A (GS1, protein).]	Go Component not found
Q08881	[Tyrosine-protein kinase ITK/TSK (EC 2.7.10.2) (T-cell-specific kinase), (Tyrosine-protein kinase Lyk) (Kinase EMT).]	Go Component not found
Q10567	[AP-1 complex subunit beta-1 (Adapter-related protein complex 1 beta-1, subunit) (Beta-adaptin 1) (Adaptor protein complex AP-1 beta-1, subunit) (Golgi adaptor HA1/AP1 adaptin beta subunit) (Clathrin, assembly protein complex 1 beta large chain).]	Go Component not found
Q12756	[Kinesin-like protein KIF1A (Axonal transporter of synaptic vesicles), (Microtubule-based motor KIF1A) (UNC-104- and KIF1A-related protein), (hUNC-104).]	Go Component not found
Q12768	Strumpellin.	Go Component not found
Q12772	[Sterol regulatory element-binding protein 2 (SREBP-2) (Sterol, regulatory element-binding transcription factor 2).]	Go Component not found
Q12901	Zinc finger protein 155.	Go Component not found
Q12929	Epidermal growth factor receptor kinase substrate 8.	Go Component not found

Q12988	Heat shock protein beta-3 (HspB3) (Heat shock 17 kDa protein).	Go Component not found
Q13057	[Bifunctional coenzyme A synthase (CoA synthase) (NBP) (POV-2), [Includes: Phosphopantetheine adenylyltransferase (EC 2.7.7.3), (Pantetheine-phosphate adenylyltransferase) (PPAT) (Dephospho-CoA, pyrophosphorylase); Dephospho-CoA kinase (EC 2.7.1.24) (DPCK), (Dephosphocoenzyme A kinase) (DPCOAK)].]	Go Component not found
Q13085	[Acetyl-CoA carboxylase 1 (EC 6.4.1.2) (ACC-alpha) [Includes: Biotin, carboxylase (EC 6.3.4.14)].]	Go Component not found
Q13105	[Zinc finger and BTB domain-containing protein 17 (Zinc finger protein, 60) (Zinc finger protein 151) (Myc-interacting zinc finger protein), (Miz-1).]	Go Component not found
Q13155	Multisynthetase complex auxiliary component p38 (Protein JTV-1).	Go Component not found
Q13228	Selenium-binding protein 1 (56 kDa selenium-binding protein) (SP56).	Go Component not found
Q13243	[Splicing factor, arginine/serine-rich 5 (Pre-mRNA-splicing factor, SRP40) (Delayed-early protein HRS).]	Go Component not found
Q13277	Syntaxin-3.	Go Component not found
Q13361	[Microfibrillar-associated protein 5 precursor (MFAP-5) (Microfibril-, associated glycoprotein 2) (MAGP-2) (MP25).]	Go Component not found
Q93050	[Vacuolar proton translocating ATPase 116 kDa subunit a isoform 1 (V-, ATPase 116 kDa isoform a1) (Clathrin-coated vesicle/synaptic vesicle, proton pump 116 kDa subunit) (Vacuolar proton pump subunit 1), (Vacuolar adenosine triphosphatase subunit Ac116).]	Go Component not found
Q93073	Uncharacterized protein KIAA0256.	Go Component not found
Q96DR5	[Short palate, lung and nasal epithelium carcinoma-associated protein 2, precursor (Parotid secretory protein) (PSP).]	Go Component not found
Q96Q77	[Calcium and integrin-binding family member 3 (Kinase-interacting, protein 3) (KIP 3).]	Go Component not found
Q99594	[Transcriptional enhancer factor TEF-5 (TEA domain family member 3), (TEAD-3) (DTEF-1).]	Go Component not found
Q99687	Homeobox protein Meis3 (Meis1-related protein 2).	Go Component not found
Q99704	Docking protein 1 (Downstream of tyrosine kinase 1) (p62(dok)) (pp62).	Go Component not found
Q99816	Tumor susceptibility gene 101 protein.	Go Component not found
Q99962	[SH3-containing GRB2-like protein 2 (EC 2.3.1.-) (Endophilin-1), (Endophilin-A1) (SH3 domain protein 2A) (EEN-B1).]	Go Component not found
Q99985	Semaphorin-3C precursor (Semaphorin E) (Sema E).	Go Component not found
Q9BWT3	[Poly(A) polymerase gamma (EC 2.7.7.19) (PAP gamma) (Polynucleotide, adenylyltransferase gamma) (SRP RNA 3'-adenylating enzyme) (Neo-, poly(A) polymerase) (Neo-PAP).]	Go Component not found
Q9BXU1	[Serine/threonine-protein kinase 31 (EC 2.7.11.1) (Serine/threonine-, protein kinase NYD-SPK) (Sugen kinase 396).]	Go Component not found
Q9GZR1	[Sentrin-specific protease 6 (EC 3.4.22.-) (Sentrin/SUMO-specific, protease SENP6) (SUMO-1-specific protease 1).]	Go Component not found
Q9GZZ0	Homeobox protein Hox-D1.	Go Component not found
Q9H1J7	Protein Wnt-5b precursor.	Go Component not found
Q9H213	[Melanoma-associated antigen H1 (MAGE-H1 antigen) (Restin) (Apoptosis-, related protein 1) (APR-1).]	Go Component not found
Q9H3T3	Semaphorin-6B precursor (Semaphorin Z) (Sema Z).	Go Component not found
Q9H4G0	Band 4.1-like protein 1 (Neuronal protein 4.1) (4.1N).	Go Component not found
Q9H583	HEAT repeat-containing protein 1 (Protein BAP28).	Go Component not found
Q9HA64	[Ketosamine-3-kinase (EC 2.7.1.-) (Fructosamine-3-kinase-related, protein).]	Go Component not found
Q9HCF6	[Transient receptor potential cation channel subfamily M member 3 (Long, transient receptor potential channel 3) (LTrpC3) (Melastatin-2), (MLSN2).]	Go Component not found
Q9HCM4	Band 4.1-like protein 5.	Go Component not found

Q9NPC8	Homeobox protein SIX2 (Sine oculis homeobox homolog 2).	Go Component not found
Q9NQW5	PR domain zinc finger protein 7 (PR domain-containing protein 7).	Go Component not found
Q9NQX4	Myosin-Vc.	Go Component not found
Q9NS71	[Gastrokine-1 precursor (18 kDa antrum mucosa protein) (AMP-18) (CA11, protein).]	Go Component not found
Q9NTI2	[Probable phospholipid-transporting ATPase IB (EC 3.6.3.1) (ATPase, class I type 8A member 2) (ML-1).]	Go Component not found
Q9NVD3	SET domain-containing protein 4.	Go Component not found
Q9NXJ5	[Pyroglutamyl-peptidase 1 (EC 3.4.19.3) (Pyroglutamyl-peptidase I), (Pyrrolidone-carboxylate peptidase) (5-oxoprolyl-peptidase) (PGP-I).]	Go Component not found
Q9NY33	[Dipeptidyl-peptidase 3 (EC 3.4.14.4) (Dipeptidyl-peptidase III) (DPP, III) (Dipeptidyl aminopeptidase III) (Dipeptidyl arylamidase III).]	Go Component not found
Q9NZB2	UPF0318 protein FAM120A.	Go Component not found
Q9P2G4	Uncharacterized protein KIAA1383.	Go Component not found
Q9P2S2	Neurexin-2-alpha precursor (Neurexin II-alpha).	Go Component not found
Q9UBD5	[Origin recognition complex subunit 3 (Origin recognition complex, subunit Latheo).]	Go Component not found
Q9UBL9	P2X purinoceptor 2 (ATP receptor) (P2X2) (Purinergic receptor).	Go Component not found
Q9UBR1	[Beta-ureidopropionase (EC 3.5.1.6) (Beta-alanine synthase) (N-, carbamoyl-beta-alanine amidohydrolase) (BUP-1).]	Go Component not found
Q9UDY2	[Tight junction protein ZO-2 (Zonula occludens protein 2) (Zona, occludens protein 2) (Tight junction protein 2).]	Go Component not found
Q9UET6	[Putative ribosomal RNA methyltransferase 1 (EC 2.1.1.-) (rRNA, (uridine-2'-O-)-methyltransferase).]	Go Component not found
Q9UGF6	Olfactory receptor 5V1 (Olfactory receptor OR6-26) (Hs6M1-21).	Go Component not found
Q9UGI9	[5'-AMP-activated protein kinase subunit gamma-3 (AMPK gamma-3 chain), (AMPK gamma3).]	Go Component not found
Q9UGM1	[Neuronal acetylcholine receptor subunit alpha-9 precursor (Nicotinic, acetylcholine receptor subunit alpha 9) (NACHR alpha 9).]	Go Component not found
Q9UH03	Neuronal-specific septin-3.	Go Component not found
Q9UHP3	[Ubiquitin carboxyl-terminal hydrolase 25 (EC 3.1.2.15) (Ubiquitin, thioesterase 25) (Ubiquitin-specific-processing protease 25), (Deubiquitinating enzyme 25) (USP on chromosome 21).]	Go Component not found
Q9UIC8	[Leucine carboxyl methyltransferase 1 (EC 2.1.1.-) (Protein-leucine O-, methyltransferase).]	Go Component not found
Q9UJ42	Probable G-protein coupled receptor 160.	Go Component not found
Q9UJW7	Zinc finger protein 229.	Go Component not found
Q9UK11	Zinc finger protein 223.	Go Component not found
Q9UK22	F-box only protein 2.	Go Component not found
Q9UK80	[Ubiquitin carboxyl-terminal hydrolase 21 (EC 3.1.2.15) (Ubiquitin, thioesterase 21) (Ubiquitin-specific-processing protease 21), (Deubiquitinating enzyme 21) (NEDD8-specific protease).]	Go Component not found
Q9UKA4	[A-kinase anchor protein 11 (Protein kinase A-anchoring protein 11), (PRKA11) (A kinase anchor protein 220 kDa) (AKAP 220) (hAKAP220).]	Go Component not found
Q9UKN7	Myosin-XV (Unconventional myosin-15).	Go Component not found
Q9UKW4	Protein vav-3.	Go Component not found
Q9ULD4	Bromodomain and PHD finger-containing protein 3.	Go Component not found
Q9ULJ8	[Neurabin-1 (Neurabin-I) (Neural tissue-specific F-actin-binding, protein I) (Protein phosphatase 1 regulatory subunit 9A).]	Go Component not found
Q9ULV0	Myosin-Vb.	Go Component not found
Q9ULV5	[Heat shock factor protein 4 (HSF 4) (Heat shock transcription factor, 4) (HSTF 4) (hHSF4).]	Go Component not found

Q9ULX5	[Zinc finger protein 179 (Brain finger protein) (RING finger protein, 112).]	Go Component not found
Q9UMQ3	Homeobox protein BarH-like 2.	Go Component not found
Q9UMX6	[Guanylyl cyclase-activating protein 2 (GCAP 2) (Guanylate cyclase, activator 1B).]	Go Component not found
Q9UN79	[SOX-13 protein (Type 1 diabetes autoantigen ICA12) (Islet cell antigen, 12).]	Go Component not found
Q9UPM8	[AP-4 complex subunit epsilon-1 (Adapter-related protein complex 4, subunit epsilon-1) (Epsilon subunit of AP-4) (AP-4 adapter complex, subunit epsilon).]	Go Component not found
Q9UPR5	[Sodium/calcium exchanger 2 precursor (Na(+)/Ca(2+)-exchange protein, 2).]	Go Component not found
Q9UPZ3	[Hermansky-Pudlak syndrome 5 protein (Alpha-integrin-binding protein, 63) (Ruby-eye protein 2 homolog) (Ru2).]	Go Component not found
Q9UQ72	[Pregnancy-specific beta-1-glycoprotein 11 precursor (PSBG-11) (PSBG-, 13).]	Go Component not found
Q9UQQ2	[SH2B adapter protein 3 (Lymphocyte-specific adapter protein Lnk), (Signal transduction protein Lnk) (Lymphocyte adapter protein).]	Go Component not found
Q9Y243	[RAC-gamma serine/threonine-protein kinase (EC 2.7.11.1) (RAC-PK-gamma), (Protein kinase Akt-3) (Protein kinase B, gamma) (PKB gamma) (STK-2).]	Go Component not found
Q9Y259	[Choline/ethanolamine kinase [Includes: Choline kinase beta, (EC 2.7.1.32) (CK); Ethanolamine kinase (EC 2.7.1.82) (EK)].]	Go Component not found
Q9Y287	[Integral membrane protein 2B (Transmembrane protein BRI) [Contains:, ABri/ADan amyloid peptide].]	Go Component not found
Q9Y2D5	[A-kinase anchor protein 2 (Protein kinase A-anchoring protein 2), (PRKA2) (AKAP-2) (AKAP-KL).]	Go Component not found
Q9Y2J0	Rabphilin-3A (Exophilin-1).	Go Component not found
Q9Y2L6	FERM domain-containing protein 4B (GRP1-binding protein GRSP1).	Go Component not found
Q9Y2N3	[Nuclear envelope pore membrane protein POM 121 (Pore membrane protein, of 121 kDa) (P145).]	Go Component not found
Q9Y2Y9	[Krueppel-like factor 13 (Transcription factor BTEB3) (Basic, transcription element-binding protein 3) (BTE-binding protein 3), (RANTES factor of late activated T-lymphocytes 1) (RFLAT-1), (Transcription factor NSLP1) (Novel Sp1-like zinc finger transcription, factor 1).]	Go Component not found
O15258	Protein RER1.	Golgi
O60499	Syntaxin-10 (Syn10).	Golgi
O60763	[General vesicular transport factor p115 (Protein USO1 homolog), (Transcytosis-associated protein) (TAP) (Vesicle-docking protein).]	Golgi
P09958	[Furin precursor (EC 3.4.21.75) (Paired basic amino acid residue, cleaving enzyme) (PACE) (Dibasic-processing enzyme).]	Golgi
P42356	[Phosphatidylinositol 4-kinase alpha (EC 2.7.1.67) (PI4-kinase alpha), (PtdIns-4-kinase alpha) (PI4K-alpha).]	Golgi
P40616	ADP-ribosylation factor-like protein 1.	Golgi
Q08378	[Golgin subfamily A member 3 (Golgin-160) (Golgi complex-associated, protein of 170 kDa) (GCP170).]	Golgi
Q9NQX7	[Integral membrane protein 2C (Transmembrane protein BRI3) (Cerebral, protein 14) [Contains: CT-BRI3].]	Golgi
Q9NWB1	Ataxin-2-binding protein 1.	Golgi
O14662	Syntaxin-16 (Syn16).	Golgi
Q92503	SEC14-like protein 1.	Golgi
Q92538	[Golgi-specific brefeldin A-resistance guanine nucleotide exchange, factor 1 (BFA-resistant GEF 1).]	Golgi
O96013	[Serine/threonine-protein kinase PAK 4 (EC 2.7.11.1) (p21-activated, kinase 4) (PAK-4).]	Golgi
P02751	[Fibronectin precursor (FN) (Cold-insoluble globulin) (CIG) [Contains:, Ugl-Y1; Ugl-Y2; Ugl-Y3].]	Golgi
P22460	[Potassium voltage-gated channel subfamily A member 5 (Voltage-gated, potassium channel subunit Kv1.5) (HK2) (HPCN1).]	Golgi

P31948	[Stress-induced-phosphoprotein 1 (STI1) (Hsc70/Hsp90-organizing, protein) (Hop) (Transformation-sensitive protein IEF SSP 3521) (NY-, REN-11 antigen).]	Golgi
P63092	[Guanine nucleotide-binding protein G(s) subunit alpha isoforms short, (Adenylate cyclase-stimulating G alpha protein).]	Golgi
P98174	[FYVE, RhoGEF and PH domain-containing protein 1 (Faciogenital, dysplasia 1 protein) (Zinc finger FYVE domain-containing protein 3), (Rho/Rac guanine nucleotide exchange factor FGD1) (Rho/Rac GEF).]	Golgi
P98194	[Calcium-transporting ATPase type 2C member 1 (EC 3.6.3.8) (ATPase 2C1), (ATP-dependent Ca(2+) pump PMR1).]	Golgi
Q02818	Nucleobindin-1 precursor (CALNUC).	Golgi
Q11130	[Alpha-(1,3)-fucosyltransferase (EC 2.4.1.-) (Galactoside 3-L-, fucosyltransferase) (Fucosyltransferase 7) (FucT-VII) (Selectin-ligand, synthase).]	Golgi
Q99550	M-phase phosphoprotein 9.	Golgi
Q9Y2D2	[UDP-N-acetylglucosamine transporter (Golgi UDP-GlcNAc transporter), (Solute carrier family 35 member A3).]	Golgi
P42858	Huntingtin (Huntington disease protein) (HD protein).	Golgi
P15144	[Aminopeptidase N (EC 3.4.11.2) (hAPN) (Alanyl aminopeptidase), (Microsomal aminopeptidase) (Aminopeptidase M) (gp150) (Myeloid plasma, membrane glycoprotein CD13) (CD13 antigen).]	Golgi
P49755	[Transmembrane emp24 domain-containing protein 10 precursor, (Transmembrane protein Tmp21) (21 kDa transmembrane-trafficking, protein) (p24delta) (S31III125) (S31I125) (Tmp-21-I).]	Golgi
P53990	[Uncharacterized protein KIAA0174 (Putative MAPK-activating protein, PM28).]	Golgi
Q13439	[Golgin subfamily A member 4 (Trans-Golgi p230) (256 kDa golgin), (Golgin-245) (Protein 72.1).]	Golgi
Q14746	[Conserved oligomeric Golgi complex component 2 (Low density, lipoprotein receptor defect C-complementing protein).]	Golgi
Q15363	[Transmembrane emp24 domain-containing protein 2 precursor (Membrane, protein p24A).]	Golgi
Q16549	[Proprotein convertase subtilisin/kexin type 7 precursor (EC 3.4.21.-), (Proprotein convertase PC7) (Subtilisin/kexin-like protease PC7), (Prohormone convertase PC7) (PC8) (hPC8) (Lymphoma proprotein, convertase).]	Golgi
Q92834	X-linked retinitis pigmentosa GTPase regulator.	Golgi
Q92896	[Golgi apparatus protein 1 precursor (Golgi sialoglycoprotein MG-160), (E-selectin ligand 1) (ESL-1) (Cysteine-rich fibroblast growth factor, receptor) (CFR-1).]	Golgi
O95235	[Kinesin-like protein KIF20A (Rabkinesin-6) (Rab6-interacting kinesin-, like protein) (GG10_2).]	Golgi
P24390	[ER lumen protein retaining receptor 1 (KDEL receptor 1) (KDEL, endoplasmic reticulum protein retention receptor 1) (Putative MAPK-, activating protein PM23).]	Golgi
Q01968	[Inositol polyphosphate 5-phosphatase OCRL-1 (EC 3.1.3.36) (Lowe, oculocerebrorenal syndrome protein).]	Golgi
P15848	[Arylsulfatase B precursor (EC 3.1.6.12) (ASB) (N-acetylgalactosamine-, 4-sulfatase) (G4S).]	Lysosomal
P05164	[Myeloperoxidase precursor (EC 1.11.1.7) (MPO) [Contains: 89 kDa, myeloperoxidase; 84 kDa myeloperoxidase; Myeloperoxidase light chain;, Myeloperoxidase heavy chain].]	Lysosomal
P35125	[Ubiquitin carboxyl-terminal hydrolase 6 (EC 3.1.2.15) (Ubiquitin, thioesterase 6) (Ubiquitin-specific-processing protease 6), (Deubiquitinating enzyme 6) (Proto-oncogene TRE-2).]	Lysosomal
Q13107	[Ubiquitin carboxyl-terminal hydrolase 4 (EC 3.1.2.15) (Ubiquitin, thioesterase 4) (Ubiquitin-specific-processing protease 4), (Deubiquitinating enzyme 4) (Ubiquitous nuclear protein homolog).]	Lysosomal
O14773	[Tripeptidyl-peptidase 1 precursor (EC 3.4.14.9) (Tripeptidyl-peptidase, I) (TPP-1) (TPP-I) (Tripeptidyl aminopeptidase) (Lysosomal pepstatin, insensitive protease) (LPIC) (Cell growth-inhibiting gene 1 protein).]	Lysosomal
P13765	[HLA class II histocompatibility antigen, DO beta chain precursor (MHC, class II antigen DOB).]	Lysosomal

P17900	[Ganglioside GM2 activator precursor (GM2-AP) (Cerebroside sulfate, activator protein) (Shingolipid activator protein 3) (SAP-3), [Contains: Ganglioside GM2 activator isoform short].]	Lysosomal
P98164	[Low-density lipoprotein receptor-related protein 2 precursor (Megalin), (Glycoprotein 330) (gp330).]	Lysosomal
O00115	[Deoxyribonuclease-2-alpha precursor (EC 3.1.22.1) (Deoxyribonuclease, II alpha) (DNase II alpha) (Acid DNase) (Lysosomal DNase II), (R31240_2).]	Lysosomal
P45974	[Ubiquitin carboxyl-terminal hydrolase 5 (EC 3.1.2.15) (Ubiquitin, thioesterase 5) (Ubiquitin-specific-processing protease 5), (Deubiquitinating enzyme 5) (Isopeptidase T).]	Lysosomal
P54803	[Galactocerebrosidase precursor (EC 3.2.1.46) (GALCERase), (Galactosylceramidase) (Galactosylceramide beta-galactosidase), (Galactocerebroside beta-galactosidase).]	Lysosomal
Q9UBX1	Cathepsin F precursor (EC 3.4.22.41) (CATSF).	Lysosomal
Q9Y2T2	[AP-3 complex subunit mu-1 (Adapter-related protein complex 3 mu-1, subunit) (Mu-adaptin 3A) (AP-3 adapter complex mu3A subunit).]	Lysosomal
O14949	[Ubiquinol-cytochrome c reductase complex ubiquinone-binding protein, QP-C (EC 1.10.2.2) (Ubiquinol-cytochrome c reductase complex 9.5 kDa, protein) (Complex III subunit VII).]	Mitochondrial
O43615	[Import inner membrane translocase subunit TIM44, mitochondrial, precursor.]	Mitochondrial
O75489	[NADH dehydrogenase [ubiquinone] iron-sulfur protein 3, mitochondrial, precursor (EC 1.6.5.3) (EC 1.6.99.3) (NADH-ubiquinone oxidoreductase, 30 kDa subunit) (Complex I-30kD) (CI-30kD).]	Mitochondrial
O75570	Peptide chain release factor 1, mitochondrial precursor (MRF-1).	Mitochondrial
O75600	[2-amino-3-ketobutyrate coenzyme A ligase, mitochondrial precursor, (EC 2.3.1.29) (AKB ligase) (Glycine acetyltransferase) (Aminoacetone, synthetase).]	Mitochondrial
P12532	[Creatine kinase, ubiquitous mitochondrial precursor (EC 2.7.3.2) (U-, MtCK) (Mia-CK) (Acidic-type mitochondrial creatine kinase).]	Mitochondrial
P14927	[Ubiquinol-cytochrome c reductase complex 14 kDa protein (EC 1.10.2.2), (Complex III subunit VI) (QP-C).]	Mitochondrial
P19404	[NADH dehydrogenase [ubiquinone] flavoprotein 2, mitochondrial, precursor (EC 1.6.5.3) (EC 1.6.99.3) (NADH-ubiquinone oxidoreductase, 24 kDa subunit).]	Mitochondrial
P46199	[Translation initiation factor IF-2, mitochondrial precursor (IF-2Mt), (IF2(mt)) (IF-2(Mt)).]	Mitochondrial
P47985	[Ubiquinol-cytochrome c reductase iron-sulfur subunit, mitochondrial, precursor (EC 1.10.2.2) (Rieske iron-sulfur protein) (RISP).]	Mitochondrial
P49753	[Acyl-coenzyme A thioesterase 2, mitochondrial precursor (EC 3.1.2.2), (Acyl-CoA thioesterase 2) (Acyl-coenzyme A thioester hydrolase 2a), (Long-chain acyl-CoA thioesterase 2) (ZAP128) (CTE-Ia).]	Mitochondrial
P49821	[NADH dehydrogenase [ubiquinone] flavoprotein 1, mitochondrial, precursor (EC 1.6.5.3) (EC 1.6.99.3) (NADH-ubiquinone oxidoreductase, 51 kDa subunit) (Complex I-51kD) (CI-51kD) (NADH dehydrogenase, flavoprotein 1).]	Mitochondrial
P56134	ATP synthase f chain, mitochondrial (EC 3.6.3.14).	Mitochondrial
Q13423	[NAD(P) transhydrogenase, mitochondrial precursor (EC 1.6.1.2), (Pyridine nucleotide transhydrogenase) (Nicotinamide nucleotide, transhydrogenase).]	Mitochondrial
Q16654	[[Pyruvate dehydrogenase [lipoamide]] kinase isozyme 4, mitochondrial, precursor (EC 2.7.11.2) (Pyruvate dehydrogenase kinase isoform 4).]	Mitochondrial
Q16762	Thiosulfate sulfurtransferase (EC 2.8.1.1) (Rhodanese).	Mitochondrial
Q92523	[Carnitine O-palmitoyltransferase I, muscle isoform (EC 2.3.1.21) (CPT, I) (CPTI-M) (Carnitine palmitoyltransferase 1B) (Carnitine, palmitoyltransferase I-like protein).]	Mitochondrial
O95831	[Apoptosis-inducing factor 1, mitochondrial precursor (EC 1.-.-.-), (Programmed cell death protein 8).]	Mitochondrial
O95847	[Mitochondrial uncoupling protein 4 (UCP 4) (Solute carrier family 25, member 27).]	Mitochondrial

O96000	[NADH dehydrogenase [ubiquinone] 1 beta subcomplex subunit 10, (EC 1.6.5.3) (EC 1.6.99.3) (NADH-ubiquinone oxidoreductase PDSW, subunit) (Complex I-PDSW) (CI-PDSW).]	Mitochondrial
P00480	[Ornithine carbamoyltransferase, mitochondrial precursor (EC 2.1.3.3), (OTCase) (Ornithine transcarbamylase).]	Mitochondrial
P04049	[RAF proto-oncogene serine/threonine-protein kinase (EC 2.7.11.1) (Raf-, 1) (C-RAF) (cRaf).]	Mitochondrial
P22695	[Ubiquinol-cytochrome-c reductase complex core protein 2, mitochondrial, precursor (EC 1.10.2.2) (Core protein II) (Complex III subunit II).]	Mitochondrial
P27338	[Amine oxidase [flavin-containing] B (EC 1.4.3.4) (Monoamine oxidase, type B) (MAO-B).]	Mitochondrial
P36551	[Coproporphyrinogen III oxidase, mitochondrial precursor (EC 1.3.3.3), (Coproporphyrinogenase) (Coprogen oxidase) (COX).]	Mitochondrial
P82914	28S ribosomal protein S15, mitochondrial precursor (S15mt) (MRP-S15).	Mitochondrial
Q02127	[Dihydroorotate dehydrogenase, mitochondrial precursor (EC 1.3.3.1), (Dihydroorotate oxidase) (DHOdehase).]	Mitochondrial
Q10713	[Mitochondrial-processing peptidase subunit alpha, mitochondrial, precursor (EC 3.4.24.64) (Alpha-MPP) (P-55).]	Mitochondrial
Q99623	[Prohibitin-2 (B-cell receptor-associated protein BAP37) (Repressor of, estrogen receptor activity) (D-prohibitin).]	Mitochondrial
Q99798	[Aconitate hydratase, mitochondrial precursor (EC 4.2.1.3) (Citrate, hydro-lyase) (Aconitase).]	Mitochondrial
Q9UI17	[Dimethylglycine dehydrogenase, mitochondrial precursor (EC 1.5.99.2), (ME2GLYDH).]	Mitochondrial
Q9Y2D0	[Carbonic anhydrase 5B, mitochondrial precursor (EC 4.2.1.1) (Carbonic, anhydrase VB) (Carbonate dehydratase VB) (CA-VB).]	Mitochondrial
O00411	[DNA-directed RNA polymerase, mitochondrial precursor (EC 2.7.7.6), (MtRPOL).]	Mitochondrial
O00483	[NADH dehydrogenase [ubiquinone] 1 alpha subcomplex subunit 4, (EC 1.6.5.3) (EC 1.6.99.3) (NADH-ubiquinone oxidoreductase MLRQ, subunit) (Complex I-MLRQ) (CI-MLRQ).]	Mitochondrial
O15235	[28S ribosomal protein S12, mitochondrial precursor (S12mt) (MRP-S12), (MT-RPS12).]	Mitochondrial
O43674	[NADH dehydrogenase [ubiquinone] 1 beta subcomplex subunit 5,, mitochondrial precursor (EC 1.6.5.3) (EC 1.6.99.3) (NADH-ubiquinone, oxidoreductase SGDH subunit) (Complex I-SGDH) (CI-SGDH).]	Mitochondrial
O43837	[Isocitrate dehydrogenase [NAD] subunit beta, mitochondrial precursor, (EC 1.1.1.41) (Isocitric dehydrogenase) (NAD(+)-specific ICDH).]	Mitochondrial
O75306	[NADH dehydrogenase [ubiquinone] iron-sulfur protein 2, mitochondrial, precursor (EC 1.6.5.3) (EC 1.6.99.3) (NADH-ubiquinone oxidoreductase, 49 kDa subunit) (Complex I-49kD) (CI-49kD).]	Mitochondrial
O75390	Citrate synthase, mitochondrial precursor (EC 2.3.3.1).	Mitochondrial
P09622	[Dihydrolipoyl dehydrogenase, mitochondrial precursor (EC 1.8.1.4), (Dihydrolipoamide dehydrogenase) (Glycine cleavage system L protein).]	Mitochondrial
P10515	[Dihydrolipoyllysine-residue acetyltransferase component of pyruvate, dehydrogenase complex, mitochondrial precursor (EC 2.3.1.12) (Pyruvate, dehydrogenase complex E2 subunit) (PDCE2) (E2) (Dihydrolipoamide S-, acetyltransferase component of pyruvate dehydrogenase complex) (PDC-, E2) (70 kDa mitochondrial autoantigen of primary biliary cirrhosis), (PBC) (M2 antigen complex 70 kDa subunit).]	Mitochondrial
P11182	[Lipoamide acyltransferase component of branched-chain alpha-keto acid, dehydrogenase complex, mitochondrial precursor (EC 2.3.1.168), (Dihydrolipoyllysine-residue (2-methylpropanoyl)transferase) (E2), (Dihydrolipoamide branched chain transacylase) (BCKAD E2 subunit).]	Mitochondrial
P11586	[C-1-tetrahydrofolate synthase, cytoplasmic (C1-THF synthase), [Includes: Methylenetetrahydrofolate dehydrogenase (EC 1.5.1.5);, Methenyltetrahydrofolate cyclohydrolase (EC 3.5.4.9);, Formyltetrahydrofolate synthetase (EC 6.3.4.3)].]	Mitochondrial
P12694	[2-oxoisovalerate dehydrogenase subunit alpha, mitochondrial precursor, (EC 1.2.4.4) (Branched-chain alpha-keto acid dehydrogenase E1, component alpha chain) (BCKDH E1-alpha) (BCKDE1A).]	Mitochondrial

P16260	[Grave disease carrier protein (GDC) (Grave disease autoantigen) (GDA), (Mitochondrial solute carrier protein homolog) (Solute carrier family, 25 member 16).]	Mitochondrial
P17568	[NADH dehydrogenase [ubiquinone] 1 beta subcomplex subunit 7, (EC 1.6.5.3) (EC 1.6.99.3) (NADH-ubiquinone oxidoreductase B18, subunit) (Complex I-B18) (CI-B18) (Cell adhesion protein SQM1).]	Mitochondrial
P45880	[Voltage-dependent anion-selective channel protein 2 (VDAC-2) (hVDAC2), (Outer mitochondrial membrane protein porin 2).]	Mitochondrial
P50416	[Carnitine O-palmitoyltransferase I, liver isoform (EC 2.3.1.21) (CPT, I) (CPTI-L) (Carnitine palmitoyltransferase 1A).]	Mitochondrial
P54098	[DNA polymerase subunit gamma-1 (EC 2.7.7.7) (Mitochondrial DNA, polymerase catalytic subunit) (PolG-alpha).]	Mitochondrial
Q14409	[Glycerol kinase, testis specific 1 (EC 2.7.1.30) (ATP:glycerol 3-, phosphotransferase) (Glycerokinase) (GK).]	Mitochondrial
Q15070	[Inner membrane protein OXA1L, mitochondrial precursor (Oxidase, assembly 1-like protein) (OXA1-like protein) (OXA1Hs) (Hsa).]	Mitochondrial
Q15120	[[Pyruvate dehydrogenase [lipoamide]] kinase isozyme 3, mitochondrial, precursor (EC 2.7.11.2) (Pyruvate dehydrogenase kinase isoform 3).]	Mitochondrial
Q16718	[NADH dehydrogenase [ubiquinone] 1 alpha subcomplex subunit 5, (EC 1.6.5.3) (EC 1.6.99.3) (NADH-ubiquinone oxidoreductase 13 kDa-B, subunit) (Complex I-13kD-B) (CI-13kD-B) (Complex I subunit B13).]	Mitochondrial
Q16798	[NADP-dependent malic enzyme, mitochondrial precursor (EC 1.1.1.40), (NADP-ME) (Malic enzyme 3).]	Mitochondrial
Q92731	Estrogen receptor beta (ER-beta).	Mitochondrial
P03905	[NADH-ubiquinone oxidoreductase chain 4 (EC 1.6.5.3) (NADH, dehydrogenase subunit 4).]	Mitochondrial
P06576	ATP synthase subunit beta, mitochondrial precursor (EC 3.6.3.14).	Mitochondrial
P23786	[Carnitine O-palmitoyltransferase 2, mitochondrial precursor, (EC 2.3.1.21) (Carnitine palmitoyltransferase II) (CPT II).]	Mitochondrial
P30049	ATP synthase delta chain, mitochondrial precursor (EC 3.6.3.14).	Mitochondrial
P36969	[Phospholipid hydroperoxide glutathione peroxidase, mitochondrial, precursor (EC 1.11.1.12) (PHGPx) (GPX-4).]	Mitochondrial
P40926	Malate dehydrogenase, mitochondrial precursor (EC 1.1.1.37).	Mitochondrial
Q02338	[D-beta-hydroxybutyrate dehydrogenase, mitochondrial precursor, (EC 1.1.1.30) (BDH) (3-hydroxybutyrate dehydrogenase).]	Mitochondrial
Q07817	Apoptosis regulator Bcl-X (Bcl-2-like 1 protein).	Mitochondrial
Q9UPV9	[Trafficking kinesin-binding protein 1 (106 kDa O-GlcNAc transferase-, interacting protein).]	Mitochondrial
Q9Y305	[Acyl-coenzyme A thioesterase 9 (EC 3.1.2.-) (Acyl-CoA thioesterase 9), (Acyl-CoA thioester hydrolase 9).]	Mitochondrial
O43181	[NADH dehydrogenase [ubiquinone] iron-sulfur protein 4, mitochondrial, precursor (EC 1.6.5.3) (EC 1.6.99.3) (NADH-ubiquinone oxidoreductase, 18 kDa subunit) (Complex I-18 kDa) (CI-18 kDa) (Complex I-AQDQ) (CI-, AQDQ).]	Mitochondrial
O43715	[TP53-regulated inhibitor of apoptosis 1 (p53-inducible cell-survival, factor) (p53CSV) (Protein 15E1.1) (WF-1).]	Mitochondrial
O75251	[NADH dehydrogenase [ubiquinone] iron-sulfur protein 7, mitochondrial, precursor (EC 1.6.5.3) (EC 1.6.99.3) (NADH-ubiquinone oxidoreductase, 20 kDa subunit) (Complex I-20kD) (CI-20kD) (PSST subunit).]	Mitochondrial
O75648	[tRNA (5-methylaminomethyl-2-thiouridylate)-methyltransferase, (EC 2.1.1.61) (Mitochondrial tRNA-specific 2-thiouridylase 1) (MTO2, homolog).]	Mitochondrial
P42765	[3-ketoacyl-CoA thiolase, mitochondrial (EC 2.3.1.16) (Beta-, ketothiolase) (Acetyl-CoA acyltransferase) (Mitochondrial 3-oxoacyl-, CoA thiolase) (T1).]	Mitochondrial
P43897	Elongation factor Ts, mitochondrial precursor (EF-Ts) (EF-TsMt).	Mitochondrial
P49411	Elongation factor Tu, mitochondrial precursor (EF-Tu) (P43).	Mitochondrial
P50440	[Glycine amidinotransferase, mitochondrial precursor (EC 2.1.4.1) (L-, arginine:glycine amidinotransferase) (Transamidinase) (AT).]	Mitochondrial
P52789	[Hexokinase-2 (EC 2.7.1.1) (Hexokinase type II) (HK II) (Muscle form, hexokinase).]	Mitochondrial

P53007	[Tricarboxylate transport protein, mitochondrial precursor (Citrate, transport protein) (CTP) (Tricarboxylate carrier protein) (Solute, carrier family 25 member 1).]	Mitochondrial
P54868	[Hydroxymethylglutaryl-CoA synthase, mitochondrial precursor, (EC 2.3.3.10) (HMG-CoA synthase) (3-hydroxy-3-methylglutaryl coenzyme, A synthase).]	Mitochondrial
P55084	[Trifunctional enzyme subunit beta, mitochondrial precursor (TP-beta), [Includes: 3-ketoacyl-CoA thiolase (EC 2.3.1.16) (Acetyl-CoA, acyltransferase) (Beta-ketothiolase)].]	Mitochondrial
P55851	Mitochondrial uncoupling protein 2 (UCP 2) (UCPH).	Mitochondrial
Q14410	[Glycerol kinase, testis specific 2 (EC 2.7.1.30) (ATP:glycerol 3-, phosphotransferase) (Glycerokinase) (GK).]	Mitochondrial
Q15118	[[Pyruvate dehydrogenase [lipoamide]] kinase isozyme 1, mitochondrial, precursor (EC 2.7.11.2) (Pyruvate dehydrogenase kinase isoform 1).]	Mitochondrial
Q16822	[Phosphoenolpyruvate carboxykinase [GTP], mitochondrial precursor, (EC 4.1.1.32) (Phosphoenolpyruvate carboxylase) (PEPCK-M).]	Mitochondrial
Q92934	[Bcl2 antagonist of cell death (BAD) (Bcl-2-binding component 6) (Bcl-, XL/Bcl-2-associated death promoter) (Bcl-2-like 8 protein).]	Mitochondrial
O76031	[ATP-dependent Clp protease ATP-binding subunit clpX-like,, mitochondrial precursor.]	Mitochondrial
O95299	[NADH dehydrogenase [ubiquinone] 1 alpha subcomplex subunit 10,, mitochondrial precursor (EC 1.6.5.3) (EC 1.6.99.3) (NADH-ubiquinone, oxidoreductase 42 kDa subunit) (Complex I-42kD) (CI-42kD).]	Mitochondrial
P04150	Glucocorticoid receptor (GR).	Mitochondrial
P04181	[Ornithine aminotransferase, mitochondrial precursor (EC 2.6.1.13), (Ornithine--oxo-acid aminotransferase) [Contains: Ornithine, aminotransferase, hepatic form; Ornithine aminotransferase, renal, form].]	Mitochondrial
P22033	[Methylmalonyl-CoA mutase, mitochondrial precursor (EC 5.4.99.2) (MCM), (Methylmalonyl-CoA isomerase).]	Mitochondrial
P26440	[Isovaleryl-CoA dehydrogenase, mitochondrial precursor (EC 1.3.99.10), (IVD).]	Mitochondrial
P31040	[Succinate dehydrogenase [ubiquinone] flavoprotein subunit,, mitochondrial precursor (EC 1.3.5.1) (Fp) (Flavoprotein subunit of, complex II).]	Mitochondrial
P31327	[Carbamoyl-phosphate synthase [ammonia], mitochondrial precursor, (EC 6.3.4.16) (Carbamoyl-phosphate synthetase I) (CPSase I).]	Mitochondrial
P31415	[Calsequestrin-1 precursor (Calsequestrin, skeletal muscle isoform), (Calmitin).]	Mitochondrial
P36776	[Lon protease homolog, mitochondrial precursor (EC 3.4.21.-) (Lon, protease-like protein) (LONP) (Mitochondrial ATP-dependent protease, Lon) (LONHs) (Serine protease 15).]	Mitochondrial
P62072	Mitochondrial import inner membrane translocase subunit Tim10.	Mitochondrial
P78540	[Arginase-2, mitochondrial precursor (EC 3.5.3.1) (Arginase II) (Non-, hepatic arginase) (Kidney-type arginase).]	Mitochondrial
P82650	Mitochondrial 28S ribosomal protein S22 (S22mt) (MRP-S22).	Mitochondrial
Q02252	[Methylmalonate-semialdehyde dehydrogenase [acylating], mitochondrial, precursor (EC 1.2.1.27) (MMSDH) (Malonate-semialdehyde dehydrogenase, [acylating]) (EC 1.2.1.18).]	Mitochondrial
Q99551	[Transcription termination factor, mitochondrial precursor (mTERF), (Mitochondrial transcription termination factor 1).]	Mitochondrial
Q99714	[3-hydroxyacyl-CoA dehydrogenase type-2 (EC 1.1.1.35) (3-hydroxyacyl-, CoA dehydrogenase type II) (Type II HADH) (3-hydroxy-2-methylbutyryl , CoA dehydrogenase) (EC 1.1.1.178) (Endoplasmic reticulum-associated, amyloid beta-peptide-binding protein) (Short-chain type, dehydrogenase/reductase XH98G2).]	Mitochondrial
Q99797	[Mitochondrial intermediate peptidase, mitochondrial precursor, (EC 3.4.24.59) (MIP).]	Mitochondrial
Q99807	[Ubiquinone biosynthesis protein COQ7 homolog (Coenzyme Q biosynthesis, protein 7 homolog) (Timing protein clk-1 homolog).]	Mitochondrial
Q9NYK5	Mitochondrial 39S ribosomal protein L39 (L39mt) (MRP-L39) (MRP-L5).	Mitochondrial

Q9UHK6	[Alpha-methylacyl-CoA racemase (EC 5.1.99.4) (2-methylacyl-CoA, racemase).]	Mitochondrial
Q9UIJ7	[GTP:AMP phosphotransferase mitochondrial (EC 2.7.4.10) (Adenylate, kinase 3) (AK3) (Adenylate kinase 3 alpha-like 1).]	Mitochondrial
Q9Y375	[Complex I intermediate-associated protein 30, mitochondrial precursor, (NADH dehydrogenase [ubiquinone] 1 alpha subcomplex assembly factor, 1).]	Mitochondrial
O14645	[Axonemal dynein light intermediate polypeptide 1 (Inner dynein arm, light chain, axonemal) (hp28).]	Neural
O15554	[Intermediate conductance calcium-activated potassium channel protein 4, (SK4) (KCa4) (IK1) (IKCa1) (Putative Gardos channel).]	Neural
O43526	[Potassium voltage-gated channel subfamily KQT member 2 (Voltage-gated, potassium channel subunit Kv7.2) (Neuroblastoma-specific potassium, channel subunit alpha KvLQT2) (KQT-like 2).]	Neural
O60359	[Voltage-dependent calcium channel gamma-3 subunit (Neuronal voltage-, gated calcium channel gamma-3 subunit).]	Neural
P12036	[Neurofilament heavy polypeptide (NF-H) (Neurofilament triplet H, protein) (200 kDa neurofilament protein).]	Neural
P21359	[Neurofibromin (Neurofibromatosis-related protein NF-1) [Contains:, Neurofibromin truncated].]	Neural
P48547	[Potassium voltage-gated channel subfamily C member 1 (Voltage-gated, potassium channel subunit Kv3.1) (Kv4) (NGK2).]	Neural
P07196	[Neurofilament light polypeptide (NF-L) (Neurofilament triplet L, protein) (68 kDa neurofilament protein).]	Neural
P22459	[Potassium voltage-gated channel subfamily A member 4 (Voltage-gated, potassium channel subunit Kv1.4) (HK1) (HPCN2) (HBK4) (HUKII).]	Neural
Q12809	[Potassium voltage-gated channel subfamily H member 2 (Voltage-gated, potassium channel subunit Kv11.1) (Ether-a-go-go-related gene, potassium channel 1) (H-ERG) (Erg1) (Ether-a-go-go-related protein 1), (Eag-related protein 1) (eag homolog).]	Neural
Q9NY46	[Sodium channel protein type 3 subunit alpha (Sodium channel protein, type III subunit alpha) (Voltage-gated sodium channel subunit alpha, Nav1.3) (Sodium channel protein, brain III subunit alpha) (Voltage-, gated sodium channel subtype III).]	Neural
O43497	[Voltage-dependent T-type calcium channel subunit alpha-1G (Voltage-, gated calcium channel subunit alpha Cav3.1) (Cav3.1c) (NBR13).]	Neural
P54289	[Voltage-dependent calcium channel subunit alpha-2/delta-1 precursor, (Voltage-gated calcium channel subunit alpha-2/delta-1) [Contains:, Voltage-dependent calcium channel subunit alpha-2-1; Voltage-dependent, calcium channel subunit delta-1].]	Neural
O95180	[Voltage-dependent T-type calcium channel subunit alpha-1H (Voltage-, gated calcium channel subunit alpha Cav3.2) (Low-voltage-activated, calcium channel alpha1 3.2 subunit).]	Neural
P07197	[Neurofilament medium polypeptide (NF-M) (Neurofilament triplet M, protein) (160 kDa neurofilament protein) (Neurofilament 3).]	Neural
Q00535	[Cell division protein kinase 5 (EC 2.7.11.22) (Cyclin-dependent kinase, 5) (Tau protein kinase II catalytic subunit) (TPKII catalytic subunit), (Serine/threonine-protein kinase PSSALRE).]	Neural
Q02641	[Voltage-dependent L-type calcium channel subunit beta-1 (CAB1), (Calcium channel voltage-dependent subunit beta 1).]	Neural
O43525	[Potassium voltage-gated channel subfamily KQT member 3 (Voltage-gated, potassium channel subunit Kv7.3) (Potassium channel subunit alpha, KvLQT3) (KQT-like 3).]	Neural
P10636	[Microtubule-associated protein tau (Neurofibrillary tangle protein), (Paired helical filament-tau) (PHF-tau).]	Neural
P51787	[Potassium voltage-gated channel subfamily KQT member 1 (Voltage-gated, potassium channel subunit Kv7.1) (IKs producing slow voltage-gated, potassium channel subunit alpha KvLQT1) (KQT-like 1).]	Neural
Q15078	[Cyclin-dependent kinase 5 activator 1 precursor (CDK5 activator 1), (Cyclin-dependent kinase 5 regulatory subunit 1) (Tau protein kinase, II 23 kDa subunit) (TPKII regulatory subunit) (p23) (p25) (p35), [Contains: Cyclin-dependent kinase 5 activator 1, p35; Cyclin-, dependent kinase 5 activator 1, p25].]	Neural
Q16352	[Alpha-internexin (Alpha-Inx) (66 kDa neurofilament protein), (Neurofilament-66) (NF-66).]	Neural
Q92952	Small conductance calcium-activated potassium channel protein 1 (SK1).	Neural
Q9H2C0	Gigaxonin (Kelch-like protein 16).	Neural

Q9NR82	[Potassium voltage-gated channel subfamily KQT member 5 (Voltage-gated, potassium channel subunit Kv7.5) (Potassium channel subunit alpha, KvLQT5) (KQT-like 5).]	Neural
Q9NYC9	Ciliary dynein heavy chain 9 (Axonemal beta dynein heavy chain 9).	Neural
O00257	[E3 SUMO-protein ligase CBX4 (Chromobox protein homolog 4) (Polycomb 2, homolog) (Pc2) (hPc2).]	Nuclear
O00482	[Orphan nuclear receptor NR5A2 (Alpha-1-fetoprotein transcription, factor) (Hepatocytic transcription factor) (B1-binding factor) (hB1F), (CYP7A promoter-binding factor) (Liver receptor homolog 1) (LRH-1).]	Nuclear
O14682	[Ectoderm-neural cortex protein 1 (ENC-1) (p53-induced gene 10 protein), (Nuclear matrix protein NRP/B).]	Nuclear
O14753	Putative transcription factor Ovo-like 1 (hOvo1).	Nuclear
O14802	[DNA-directed RNA polymerase III subunit RPC1 (EC 2.7.7.6) (RNA, polymerase III subunit C1) (DNA-directed RNA polymerase III subunit A), (DNA-directed RNA polymerase III largest subunit) (RPC155) (C160).]	Nuclear
O15119	T-box transcription factor TBX3 (T-box protein 3).	Nuclear
O15265	Ataxin-7 (Spinocerebellar ataxia type 7 protein).	Nuclear
O43670	Zinc finger protein 207.	Nuclear
O43791	Speckle-type POZ protein (HIB homolog 1) (Roadkill homolog 1).	Nuclear
O43830	Zinc finger protein 73 (Zinc finger protein 186) (hZNF2).	Nuclear
O43929	Origin recognition complex subunit 4.	Nuclear
O60231	[Putative pre-mRNA-splicing factor ATP-dependent RNA helicase DHX16, (EC 3.6.1.-) (DEAH-box protein 16) (ATP-dependent RNA helicase #3).]	Nuclear
O60248	SOX-15 protein (SOX-20 protein) (SOX-12 protein).	Nuclear
O60287	Nucleolar preribosomal-associated protein 1 (Fragment).	Nuclear
O60663	[LIM homeobox transcription factor 1 beta (LIM/homeobox protein LMX1B), (LIM/homeobox protein 1.2) (LMX-1.2).]	Nuclear
O75386	Tubby-related protein 3 (Tubby-like protein 3).	Nuclear
P09884	DNA polymerase alpha catalytic subunit (EC 2.7.7.7).	Nuclear
P10243	Myb-related protein A (A-Myb).	Nuclear
P10828	Thyroid hormone receptor beta-1.	Nuclear
P11308	Transcriptional regulator ERG (Transforming protein ERG).	Nuclear
P11388	[DNA topoisomerase 2-alpha (EC 5.99.1.3) (DNA topoisomerase II, alpha, isozyme).]	Nuclear
P11802	[Cell division protein kinase 4 (EC 2.7.11.22) (Cyclin-dependent kinase, 4) (PSK-J3).]	Nuclear
P13051	Uracil-DNA glycosylase (EC 3.2.2.-) (UDG).	Nuclear
P14653	Homeobox protein Hox-B1 (Hox-2I).	Nuclear
P15923	[Transcription factor E2-alpha (Immunoglobulin enhancer-binding factor, E12/E47) (Transcription factor 3) (TCF-3) (Immunoglobulin, transcription factor 1) (Transcription factor ITF-1) (Kappa-E2-binding, factor).]	Nuclear
P16455	[Methylated-DNA--protein-cysteine methyltransferase (EC 2.1.1.63) (6-O-, methylguanine-DNA methyltransferase) (MGMT) (O-6-methylguanine-DNA-, alkyltransferase).]	Nuclear
P17098	Zinc finger protein 8 (Zinc finger protein HF.18).	Nuclear
P17482	Homeobox protein Hox-B9 (Hox-2E) (Hox-2.5).	Nuclear
P17844	[Probable ATP-dependent RNA helicase DDX5 (EC 3.6.1.-) (DEAD box, protein 5) (RNA helicase p68).]	Nuclear
P41970	[ETS domain-containing protein Elk-3 (ETS-related protein NET) (ETS-, related protein ERP) (SRF accessory protein 2) (SAP-2).]	Nuclear

P42167	[Lamina-associated polypeptide 2, isoforms beta/gamma (Thymopoietin,, isoforms beta/gamma) (TP beta/gamma) (Thymopoietin-related peptide, isoforms beta/gamma) (TPRP isoforms beta/gamma) [Contains:, Thymopoietin (TP) (Splenin); Thymopentin (TP5)].]	Nuclear
P42568	[Protein AF-9 (ALL1 fused gene from chromosome 9 protein), (Myeloid/lymphoid or mixed-lineage leukemia translocated to chromosome, 3 protein) (YEATS domain-containing protein 3).]	Nuclear
P42695	[Condensin-II complex subunit D3 (Non-SMC condensin II complex subunit, D3) (hCAP-D3).]	Nuclear
P43243	Matrin-3.	Nuclear
P43268	[ETS translocation variant 4 (Adenovirus E1A enhancer-binding protein), (E1A-F).]	Nuclear
P43351	DNA repair protein RAD52 homolog.	Nuclear
P46063	ATP-dependent DNA helicase Q1 (EC 3.6.1.-) (DNA-dependent ATPase Q1).	Nuclear
P46736	BRCA1/BRCA2-containing complex subunit 3.	Nuclear
P48382	DNA-binding protein RFX5 (Regulatory factor X subunit 5).	Nuclear
P49137	[MAP kinase-activated protein kinase 2 (EC 2.7.11.1) (MAPK-activated, protein kinase 2) (MAPKAP kinase 2) (MAPKAPK-2) (MK2).]	Nuclear
P49715	CCAAT/enhancer-binding protein alpha (C/EBP alpha).	Nuclear
P49959	[Double-strand break repair protein MRE11A (MRE11 homolog 1) (MRE11, meiotic recombination 11 homolog A).]	Nuclear
P50219	Motor neuron and pancreas homeobox protein 1 (Homeobox protein HB9).	Nuclear
P50402	Emerin.	Nuclear
P51531	[Probable global transcription activator SNF2L2 (EC 3.6.1.-) (ATP-, dependent helicase SMARCA2) (SNF2-alpha) (SWI/SNF-related matrix-, associated actin-dependent regulator of chromatin subfamily A member, 2) (hBRM).]	Nuclear
P51815	Zinc finger protein 75 (Zinc finger protein 82).	Nuclear
P52741	Zinc finger protein 134.	Nuclear
P52744	Zinc finger protein 138.	Nuclear
P53041	[Serine/threonine-protein phosphatase 5 (EC 3.1.3.16) (PP5) (Protein, phosphatase T) (PP-T) (PPT).]	Nuclear
P53567	CCAAT/enhancer-binding protein gamma (C/EBP gamma).	Nuclear
P54278	PMS1 protein homolog 2 (DNA mismatch repair protein PMS2).	Nuclear
P54845	Neural retina-specific leucine zipper protein (NRL) (D14S46E).	Nuclear
P56182	[RRP1-like protein (Protein NNP-1) (Novel nuclear protein 1) (Nucleolar, protein Nop52).]	Nuclear
P56537	[Eukaryotic translation initiation factor 6 (eIF-6) (B4 integrin, interactor) (CAB) (p27(BBP)) (B(2)GCN homolog).]	Nuclear
P56559	[ADP-ribosylation factor-like protein 4C (ADP-ribosylation factor-like, protein 7) (ADP-ribosylation factor-like protein LAK).]	Nuclear
P57071	[PR domain zinc finger protein 15 (PR domain-containing protein 15), (Zinc finger protein 298).]	Nuclear
Q13398	Zinc finger protein 211 (Zinc finger protein C2H2-25).	Nuclear
Q13415	Origin recognition complex subunit 1 (Replication control protein 1).	Nuclear
Q13569	G/T mismatch-specific thymine DNA glycosylase (EC 3.2.2.-).	Nuclear
Q13772	[Nuclear receptor coactivator 4 (NCoA-4) (70 kDa androgen receptor, coactivator) (70 kDa AR-activator) (Ret-activating protein ELE1).]	Nuclear
Q14106	Protein Tob2 (Transducer of erbB-2 2).	Nuclear
Q14161	[ARF GTPase-activating protein GIT2 (G protein-coupled receptor kinase-, interactor 2) (GRK-interacting protein 2) (Cool-interacting tyrosine-, phosphorylated protein 2) (CAT2) (CAT-2).]	Nuclear
Q14191	Werner syndrome ATP-dependent helicase (EC 3.6.1.-).	Nuclear

Q14995	[Orphan nuclear receptor NR1D2 (Rev-erb-beta) (EAR-1R) (Orphan nuclear, hormone receptor BD73).]	Nuclear
Q15059	Bromodomain-containing protein 3 (RING3-like protein).	Nuclear
Q15306	[Interferon regulatory factor 4 (IRF-4) (Lymphocyte-specific interferon, regulatory factor) (LSIRF) (NF-EM5) (Multiple myeloma oncogene 1).]	Nuclear
Q15398	[Disks large-associated protein DLG7 (Discs large homolog 7) (Hepatoma, up-regulated protein) (HURP).]	Nuclear
Q15695	[U2 small nuclear ribonucleoprotein auxiliary factor 35 kDa subunit-, related protein 1 (U2(RNU2) small nuclear RNA auxiliary factor 1-like, 1) (CCCH type zinc finger, RNA-binding motif and serine/arginine rich, protein 1).]	Nuclear
Q15906	[Vacuolar protein sorting-associated protein 72 homolog (Transcription, factor-like 1) (Protein YL-1).]	Nuclear
Q16534	Hepatic leukemia factor.	Nuclear
Q16665	[Hypoxia-inducible factor 1 alpha (HIF-1 alpha) (HIF1 alpha) (ARNT-, interacting protein) (Member of PAS protein 1) (Basic-helix-loop-, helix-PAS protein MOP1).]	Nuclear
O75971	[snRNA-activating protein complex subunit 5 (SNAPc subunit 5) (Small, nuclear RNA-activating complex polypeptide 5) (snRNA-activating, protein complex 19 kDa subunit) (SNAPc 19 kDa subunit).]	Nuclear
O94916	[Nuclear factor of activated T-cells 5 (T-cell transcription factor, NFAT5) (NF-AT5) (Tonicity-responsive enhancer-binding protein) (TonE-, binding protein) (TonEBP).]	Nuclear
O95218	[Zinc finger Ran-binding domain-containing protein 2 (Zinc finger, protein 265) (Zinc finger, splicing).]	Nuclear
P23769	Endothelial transcription factor GATA-2 (GATA-binding protein 2).	Nuclear
P24468	[COUP transcription factor 2 (COUP-TF2) (COUP-TF II) (Apolipoprotein AI, regulatory protein 1) (ARP-1).]	Nuclear
P24928	[DNA-directed RNA polymerase II subunit RPB1 (EC 2.7.7.6) (RNA, polymerase II subunit B1) (DNA-directed RNA polymerase II subunit A), (DNA-directed RNA polymerase III largest subunit).]	Nuclear
P27694	[Replication protein A 70 kDa DNA-binding subunit (RP-A) (RF-A), (Replication factor-A protein 1) (Single-stranded DNA-binding protein), (p70).]	Nuclear
P28370	[Probable global transcription activator SNF2L1 (EC 3.6.1.-), (Nucleosome-remodeling factor subunit SNF2L) (ATP-dependent helicase, SMARCA1) (SWI/SNF-related matrix-associated actin-dependent regulator, of chromatin subfamily A member 1).]	Nuclear
P31629	[Human immunodeficiency virus type I enhancer-binding protein 2 (HIV-, EP2) (MHC-binding protein 2) (MBP-2).]	Nuclear
P32242	Homeobox protein OTX1 (Orthodenticle homolog 1).	Nuclear
P35226	[Polycomb complex protein BMI-1 (Polycomb group RING finger protein 4), (RING finger protein 51).]	Nuclear
P35251	[Replication factor C subunit 1 (Replication factor C large subunit), (RF-C 140 kDa subunit) (Activator 1 140 kDa subunit) (Activator 1, large subunit) (A1 140 kDa subunit) (DNA-binding protein PO-GA).]	Nuclear
P35659	Protein DEK.	Nuclear
P39748	[Flap endonuclease 1 (EC 3.1.-.-) (Flap structure-specific endonuclease, 1) (FEN-1) (Maturation factor 1) (MF1) (hFEN-1) (DNase IV).]	Nuclear
P63162	[Small nuclear ribonucleoprotein-associated protein N (snRNP-N) (Sm, protein N) (Sm-N) (SmN) (Sm-D) (Tissue-specific-splicing protein).]	Nuclear
P83870	[PHD finger-like domain-containing protein 5A (PHD finger-like domain, protein 5A) (Splicing factor 3B-associated 14 kDa protein) (SF3b14b).]	Nuclear
P98169	Zinc finger X-linked protein ZXDB.	Nuclear
Q01167	[Forkhead box protein K2 (Interleukin enhancer-binding factor 1), (Cellular transcription factor ILF-1) (FOXK1).]	Nuclear
Q01780	[Exosome component 10 (Polymyositis/scleroderma autoantigen 2), (Autoantigen PM/Scl 2) (Polymyositis/scleroderma autoantigen 100 kDa), (PM/Scl-100) (P100 polymyositis-scleroderma overlap syndrome-, associated autoantigen).]	Nuclear

Q01954	Zinc finger protein basonuclin-1.	Nuclear
Q03112	Ecotropic virus integration site 1 protein homolog (EVI-1).	Nuclear
Q03701	[CCAAT/enhancer-binding protein zeta (CCAAT-box-binding transcription, factor) (CCAAT-binding factor) (CBF).]	Nuclear
Q04743	[Homeobox protein EMX2 (Empty spiracles homolog 2) (Empty spiracles-, like protein 2).]	Nuclear
Q05516	[Zinc finger and BTB domain-containing protein 16 (Zinc finger protein, PLZF) (Promyelocytic leukemia zinc finger protein) (Zinc finger, protein 145).]	Nuclear
Q12950	[Forkhead box protein D4 (Forkhead-related protein FKHL9) (Forkhead-, related transcription factor 5) (FREAC-5) (Myeloid factor-alpha).]	Nuclear
Q13127	[RE1-silencing transcription factor (Neural-restrictive silencer, factor) (X2 box repressor).]	Nuclear
Q13283	[Ras GTPase-activating protein-binding protein 1 (EC 3.6.1.-) (G3BP-1), (ATP-dependent DNA helicase VIII) (HDH-VIII) (GAP SH3 domain-binding, protein 1).]	Nuclear
Q92988	Homeobox protein DLX-4 (DLX-7) (DLX-8) (Beta protein 1).	Nuclear
Q99676	Zinc finger protein 184.	Nuclear
Q9UBC3	[DNA (cytosine-5)-methyltransferase 3B (EC 2.1.1.37) (Dnmt3b) (DNA, methyltransferase HsaIIIB) (DNA MTase HsaIIIB) (M.HsaIIIB).]	Nuclear
Q9UBU8	[Mortality factor 4-like protein 1 (MORF-related gene 15 protein), (Transcription factor-like protein MRG15) (MSL3-1 protein).]	Nuclear
Q9UHC1	DNA mismatch repair protein Mlh3 (MutL protein homolog 3).	Nuclear
Q9UKS7	Zinc finger protein Helios (Ikaros family zinc finger protein 2).	Nuclear
Q9UL36	Zinc finger protein 236.	Nuclear
Q9UL59	[Zinc finger protein 214 (BWSCR2-associated zinc finger protein 1) (BAZ, 1).]	Nuclear
Q9ULV3	Cip1-interacting zinc finger protein (Nuclear protein NP94).	Nuclear
Q9ULW0	[Targeting protein for Xklp2 (Restricted expression proliferation-, associated protein 100) (p100) (Differentially expressed in cancerous, and non-cancerous lung cells 2) (DIL-2) (Protein fls353), (Hepatocellular carcinoma-associated antigen 519).]	Nuclear
Q9Y2L8	[Zinc finger protein with KRAB and SCAN domains 5 (Zinc finger protein, 95 homolog) (Zfp-95).]	Nuclear
Q9Y333	[U6 snRNA-associated Sm-like protein LSm2 (snRNP core Sm-like protein, Sm-x5) (Small nuclear ribonuclear protein D homolog) (Protein G7b).]	Nuclear
O00258	Tryptophan-rich protein (Congenital heart disease 5 protein).	Nuclear
O00358	[Forkhead box protein E1 (Thyroid transcription factor 2) (TTF-2), (Forkhead-related protein FKHL15).]	Nuclear
O14628	Zinc finger protein 195.	Nuclear
O14981	[TATA-binding protein-associated factor 172 (EC 3.6.1.-) (ATP-dependent, helicase BTAF1) (TBP-associated factor 172) (TAF-172) (TAF(II)170) (B-, TFIID transcription factor-associated 170 kDa subunit).]	Nuclear
O15117	[FYN-binding protein (FYN-T-binding protein) (FYB-120/130) (p120/p130), (SLP-76-associated phosphoprotein) (SLAP-130) (Adhesion and, degranulation promoting adaptor protein) (ADAP).]	Nuclear
O15297	[Protein phosphatase 1D (EC 3.1.3.16) (Protein phosphatase 2C isoform, delta) (PP2C-delta) (p53-induced protein phosphatase 1) (Protein, phosphatase magnesium-dependent 1 delta).]	Nuclear
O43345	Zinc finger protein 208.	Nuclear
O43390	Heterogeneous nuclear ribonucleoprotein R (hnRNP R).	Nuclear
O43623	[Zinc finger protein SLUG (Neural crest transcription factor Slug), (Snail homolog 2).]	Nuclear
O43707	[Alpha-actinin-4 (Non-muscle alpha-actinin 4) (F-actin cross-linking, protein).]	Nuclear

O60583	Cyclin-T2 (CycT2).	Nuclear
O75346	[Zinc finger protein 253 (Bone marrow zinc finger 1) (BMZF-1) (Zinc, finger protein 411).]	Nuclear
O75417	DNA polymerase theta (EC 2.7.7.7) (DNA polymerase eta).	Nuclear
O75478	Transcriptional adapter 2-like (ADA2-like protein).	Nuclear
P10073	[Zinc finger and SCAN domain-containing protein 22 (Zinc finger protein, 50) (Krueppel-related zinc finger protein 2) (Protein HKR2).]	Nuclear
P15036	Protein C-ets-2.	Nuclear
P17026	Zinc finger protein 22 (Zinc finger protein KOX15) (Krox-26 protein).	Nuclear
P17036	[Zinc finger protein 3 (Zinc finger protein KOX25) (Zinc finger protein, HF.12) (HZF3.1).]	Nuclear
P17980	[26S protease regulatory subunit 6A (Proteasome 26S subunit ATPase 3), (Tat-binding protein 1) (TBP-1) (Proteasome subunit P50).]	Nuclear
P18858	[DNA ligase 1 (EC 6.5.1.1) (DNA ligase I) (Polydeoxyribonucleotide, synthase [ATP] 1).]	Nuclear
P19544	Wilms tumor protein (WT33).	Nuclear
P42685	[Tyrosine-protein kinase FRK (EC 2.7.10.2) (FYN-related kinase), (Nuclear tyrosine protein kinase RAK).]	Nuclear
P43694	Transcription factor GATA-4 (GATA-binding factor 4).	Nuclear
P46087	[Putative RNA methyltransferase NOL1 (EC 2.1.1.-) (Proliferating-cell, nucleolar antigen p120) (Proliferation-associated nucleolar protein, p120).]	Nuclear
P48431	Transcription factor SOX-2.	Nuclear
P48552	[Nuclear receptor-interacting protein 1 (Nuclear factor RIP140), (Receptor-interacting protein 140).]	Nuclear
P49910	[Zinc finger protein 165 (LD65) (Zinc finger and SCAN domain-containing, protein 7) (Cancer/testis antigen 53) (CT53).]	Nuclear
P49916	[DNA ligase 3 (EC 6.5.1.1) (DNA ligase III) (Polydeoxyribonucleotide, synthase [ATP] 3).]	Nuclear
P51508	Zinc finger protein 81 (HFZ20).	Nuclear
P51522	[Zinc finger protein 83 (Zinc finger protein 816B) (Zinc finger protein, HPF1).]	Nuclear
P51532	[Probable global transcription activator SNF2L4 (EC 3.6.1.-) (ATP-, dependent helicase SMARCA4) (SNF2-beta) (BRG-1 protein) (Mitotic, growth and transcription activator) (Brahma protein homolog 1), (SWI/SNF-related matrix-associated actin-dependent regulator of, chromatin subfamily A member 4).]	Nuclear
P51826	[AF4/FMR2 family member 3 (Protein LAF-4) (Lymphoid nuclear protein, related to AF4).]	Nuclear
P51946	Cyclin-H (MO15-associated protein) (p37) (p34).	Nuclear
P52298	[Nuclear cap-binding protein subunit 2 (20 kDa nuclear cap-binding, protein) (NCBP 20 kDa subunit) (CBP20) (NCBP-interacting protein 1), (NIP1) (Cell proliferation-inducing gene 55 protein).]	Nuclear
P52742	Zinc finger protein 135 (Zinc finger protein 61).	Nuclear
P54727	[UV excision repair protein RAD23 homolog B (hHR23B) (XP-C repair-, complementing complex 58 kDa protein) (p58).]	Nuclear
P55895	V(D)J recombination-activating protein 2 (RAG-2).	Nuclear
Q13416	Origin recognition complex subunit 2.	Nuclear
Q13428	Treacle protein (Treacher Collins syndrome protein).	Nuclear
Q13472	DNA topoisomerase 3-alpha (EC 5.99.1.2) (DNA topoisomerase III alpha).	Nuclear
Q13526	[Peptidyl-prolyl cis-trans isomerase NIMA-interacting 1 (EC 5.2.1.8), (Rotamase Pin1) (PPIase Pin1).]	Nuclear
Q13574	[Diacylglycerol kinase zeta (EC 2.7.1.107) (Diglyceride kinase zeta), (DGK-zeta) (DAG kinase zeta).]	Nuclear

Q13627	[Dual specificity tyrosine-phosphorylation-regulated kinase 1A, (EC 2.7.12.1) (Protein kinase minibrain homolog) (MNBH) (HP86) (Dual, specificity YAK1-related kinase) (hMNB).]	Nuclear
Q13823	Nucleolar GTP-binding protein 2 (Autoantigen NGP-1).	Nuclear
Q14192	[Four and a half LIM domains protein 2 (FHL-2) (Skeletal muscle LIM-, protein 3) (SLIM 3) (LIM domain protein DRAL).]	Nuclear
Q14693	Lipin-1.	Nuclear
Q14929	Zinc finger protein 169.	Nuclear
Q15014	[Mortality factor 4-like protein 2 (MORF-related gene X protein), (Transcription factor-like protein MRGX) (MSL3-2 protein).]	Nuclear
Q15054	DNA polymerase subunit delta-3 (DNA polymerase subunit delta p66).	Nuclear
Q15349	[Ribosomal protein S6 kinase alpha-2 (EC 2.7.11.1) (S6K-alpha 2) (90, kDa ribosomal protein S6 kinase 2) (p90-RSK 2) (Ribosomal S6 kinase 3), (RSK-3) (pp90RSK3) (MAP kinase-activated protein kinase 1c), (MAPKAPK1C).]	Nuclear
Q15393	[Splicing factor 3B subunit 3 (Spliceosome-associated protein 130) (SAP, 130) (SF3b130) (Pre-mRNA-splicing factor SF3b 130 kDa subunit), (STAF130).]	Nuclear
Q15633	[TAR RNA-binding protein 2 (Trans-activation-responsive RNA-binding, protein).]	Nuclear
Q16181	Septin-7 (CDC10 protein homolog).	Nuclear
Q16384	[Protein SSX1 (Synovial sarcoma, X breakpoint 1) (Cancer/testis antigen, 5.1) (CT5.1).]	Nuclear
Q16527	[Cysteine and glycine-rich protein 2 (Cysteine-rich protein 2) (CRP2), (Smooth muscle cell LIM protein) (SmLIM) (LIM domain only protein 5).]	Nuclear
Q16629	Splicing factor, arginine/serine-rich 7 (Splicing factor 9G8).	Nuclear
Q16666	[Gamma-interferon-inducible protein Ifi-16 (Interferon-inducible, myeloid differentiation transcriptional activator) (IFI 16).]	Nuclear
Q8IXQ6	[Poly [ADP-ribose] polymerase 9 (EC 2.4.2.30) (PARP-9) (B aggressive, lymphoma protein).]	Nuclear
Q92794	[Histone acetyltransferase MYST3 (EC 2.3.1.48) (EC 2.3.1.-) (MYST, protein 3) (MOZ, YBF2/SAS3, SAS2 and TIP60 protein 3) (Runt-related, transcription factor-binding protein 2) (Monocytic leukemia zinc, finger protein) (Zinc finger protein 220).]	Nuclear
Q92949	[Forkhead box protein J1 (Forkhead-related protein FKHL13) (Hepatocyte, nuclear factor 3 forkhead homolog 4) (HFH-4).]	Nuclear
O94782	[Ubiquitin carboxyl-terminal hydrolase 1 (EC 3.1.2.15) (Ubiquitin, thioesterase 1) (Ubiquitin-specific-processing protease 1), (Deubiquitinating enzyme 1) (hUBP).]	Nuclear
O95696	Bromodomain-containing protein 1 (BR140-like protein).	Nuclear
O95935	T-box transcription factor TBX18 (T-box protein 18).	Nuclear
O95985	[DNA topoisomerase 3-beta-1 (EC 5.99.1.2) (DNA topoisomerase III beta-, 1).]	Nuclear
P21580	[Tumor necrosis factor, alpha-induced protein 3 (EC 3.-.-.-) (Putative, DNA-binding protein A20) (Zinc finger protein A20).]	Nuclear
P22626	Heterogeneous nuclear ribonucleoproteins A2/B1 (hnRNP A2 / hnRNP B1).	Nuclear
P23025	[DNA-repair protein complementing XP-A cells (Xeroderma pigmentosum, group A-complementing protein).]	Nuclear
P25205	[DNA replication licensing factor MCM3 (DNA polymerase alpha, holoenzyme-associated protein P1) (RLF subunit beta) (P102 protein), (P1-MCM3).]	Nuclear
P28340	[DNA polymerase delta catalytic subunit (EC 2.7.7.7) (DNA polymerase, subunit delta p125).]	Nuclear
P29375	[Histone demethylase JARID1A (EC 1.14.11.-) (Jumonji/ARID domain-, containing protein 1A) (Retinoblastoma-binding protein 2) (RBBP-2).]	Nuclear
P33991	DNA replication licensing factor MCM4 (CDC21 homolog) (P1-CDC21).	Nuclear
P35274	Zinc finger protein 125 (HZF-3) (Fragment).	Nuclear
P38432	Coilin (p80).	Nuclear

P40424	[Pre-B-cell leukemia transcription factor 1 (Homeobox protein PBX1), (Homeobox protein PRL).]	Nuclear
P40692	DNA mismatch repair protein Mlh1 (MutL protein homolog 1).	Nuclear
P41182	[B-cell lymphoma 6 protein (BCL-6) (Zinc finger protein 51) (LAZ-3, protein) (BCL-5) (Zinc finger and BTB domain-containing protein 27).]	Nuclear
P62314	[Small nuclear ribonucleoprotein Sm D1 (snRNP core protein D1) (Sm-D1), (Sm-D autoantigen).]	Nuclear
P62988	Ubiquitin.	Nuclear
Q9BZE4	[Nucleolar GTP-binding protein 1 (Chronic renal failure gene protein), (GTP-binding protein NGB).]	Nuclear
Q00839	[Heterogeneous nuclear ribonucleoprotein U (hnRNP U) (Scaffold, attachment factor A) (SAF-A) (p120) (pp120).]	Nuclear
Q00987	[E3 ubiquitin-protein ligase Mdm2 (EC 6.3.2.-) (p53-binding protein, Mdm2) (Oncoprotein Mdm2) (Double minute 2 protein) (Hdm2).]	Nuclear
Q01196	[Runt-related transcription factor 1 (Core-binding factor, alpha 2, subunit) (CBF-alpha 2) (Acute myeloid leukemia 1 protein) (Oncogene, AML-1) (Polyomavirus enhancer-binding protein 2 alpha B subunit), (PEBP2-alpha B) (PEA2-alpha B) (SL3-3 enhancer factor 1 alpha B, subunit) (SL3/AKV core-binding factor alpha B subunit).]	Nuclear
Q01538	[Myelin transcription factor 1 (MyT1) (MyTI) (Proteolipid protein-, binding protein) (PLPB1).]	Nuclear
Q01850	[Cerebellar degeneration-related protein 2 (Paraneoplastic cerebellar, degeneration-associated antigen) (Major Yo paraneoplastic antigen).]	Nuclear
Q03181	[Peroxisome proliferator-activated receptor delta (PPAR-delta) (PPAR-, beta) (Nuclear hormone receptor 1) (NUC1) (NUCI).]	Nuclear
Q03828	Homeobox even-skipped homolog protein 2 (EVX-2).	Nuclear
Q04727	Transducin-like enhancer protein 4.	Nuclear
Q05481	Zinc finger protein 91 (Zinc finger protein HTF10) (HPF7).	Nuclear
Q05519	[Splicing factor arginine/serine-rich 11 (Arginine-rich 54 kDa nuclear, protein) (p54).]	Nuclear
Q06265	[Exosome complex exonuclease RRP45 (EC 3.1.13.-) (Exosome component 9), (Polymyositis/scleroderma autoantigen 1) (Autoantigen PM/Scl 1), (Polymyositis/scleroderma autoantigen 75 kDa) (PM/Scl-75) (P75, polymyositis-scleroderma overlap syndrome-associated autoantigen).]	Nuclear
Q06730	[Zinc finger protein 33A (Zinc finger protein 11A) (Zinc finger protein, KOX31) (Zinc finger and ZAK-associated protein with KRAB domain), (ZZaPK).]	Nuclear
Q07869	Peroxisome proliferator-activated receptor alpha (PPAR-alpha).	Nuclear
Q09028	[Histone-binding protein RBBP4 (Retinoblastoma-binding protein 4), (RBBP-4) (Retinoblastoma-binding protein p48) (Chromatin assembly, factor 1 subunit C) (CAF-1 subunit C) (Chromatin assembly factor I p48, subunit) (CAF-I 48 kDa subunit) (CAF-I p48) (Nucleosome-remodeling, factor subunit RBAP48).]	Nuclear
Q09666	[Neuroblast differentiation-associated protein AHNAK (Desmoyokin), (Fragments).]	Nuclear
Q12951	[Forkhead box protein I1 (Forkhead-related protein FKHL10) (Forkhead-, related transcription factor 6) (FREAC-6) (Hepatocyte nuclear factor 3, forkhead homolog 3) (HNF-3/fork-head homolog 3) (HFH-3).]	Nuclear
Q13049	[Tripartite motif-containing protein 32 (EC 6.3.2.-) (Zinc finger, protein HT2A) (72 kDa Tat-interacting protein).]	Nuclear
Q13115	[Dual specificity protein phosphatase 4 (EC 3.1.3.48) (EC 3.1.3.16), (Mitogen-activated protein kinase phosphatase 2) (MAP kinase, phosphatase 2) (MKP-2) (Dual specificity protein phosphatase hVH2).]	Nuclear
Q13268	[Dehydrogenase/reductase SDR family member 2 (EC 1.1.-.-) (HEP27, protein) (Protein D).]	Nuclear
Q13285	[Steroidogenic factor 1 (STF-1) (SF-1) (Adrenal 4-binding protein), (Steroid hormone receptor Ad4BP) (Fushi tarazu factor homolog 1).]	Nuclear
Q99081	[Transcription factor 12 (Transcription factor HTF-4) (E-box-binding, protein) (DNA-binding protein HTF4).]	Nuclear

Q99708	[Retinoblastoma-binding protein 8 (RBBP-8) (CtBP-interacting protein), (CtIP) (Retinoblastoma-interacting protein and myosin-like) (RIM).]	Nuclear
Q99801	Homeobox protein Nkx-3.1.	Nuclear
Q9HB58	[Sp110 nuclear body protein (Speckled 110 kDa) (Transcriptional, coactivator Sp110) (Interferon-induced protein 41/75).]	Nuclear
Q9NYV4	[Cell division cycle 2-related protein kinase 7 (EC 2.7.11.22) (CDC2-, related protein kinase 7) (Cdc2-related kinase, arginine/serine-rich), (CrkRS).]	Nuclear
Q9UDV7	Zinc finger protein 282 (HTLV-I U5RE-binding protein 1) (HUB-1).	Nuclear
Q9UEE5	[Serine/threonine-protein kinase 17A (EC 2.7.11.1) (DAP kinase-related, apoptosis-inducing protein kinase 1).]	Nuclear
Q9UGU5	High mobility group protein 2-like 1 (Protein HMGBCG).	Nuclear
Q9UMN6	[WW domain-binding protein 7 (Myeloid/lymphoid or mixed-lineage, leukemia protein 4) (Trithorax homolog 2).]	Nuclear
Q9UPN9	[E3 ubiquitin-protein ligase TRIM33 (EC 6.3.2.-) (Tripartite motif-, containing protein 33) (Transcription intermediary factor 1-gamma), (TIF1-gamma) (Ectodermin homolog) (RET-fused gene 7 protein) (Protein, Rfg7).]	Nuclear
Q9Y2P8	RNA 3'-terminal phosphate cyclase-like protein.	Nuclear
Q9Y2T1	[Axin-2 (Axis inhibition protein 2) (Conductin) (Axin-like protein), (Axil).]	Nuclear
Q9Y2U8	Inner nuclear membrane protein Man1 (LEM domain-containing protein 3).	Nuclear
Q9Y2X9	[Zinc finger protein 281 (Zinc finger DNA-binding protein 99), (Transcription factor ZBP-99) (GC-box-binding zinc finger protein 1).]	Nuclear
O00567	Nucleolar protein 5A (Nucleolar protein Nop56).	Nuclear
O14901	[Krueppel-like factor 11 (Transforming growth factor-beta-inducible, early growth response protein 2) (TGFB-inducible early growth response, protein 2) (TIEG-2).]	Nuclear
O15164	[Transcription intermediary factor 1-alpha (TIF1-alpha) (Tripartite, motif-containing protein 24) (RING finger protein 82).]	Nuclear
O15353	Forkhead box protein N1 (Winged-helix transcription factor nude).	Nuclear
O15457	MutS protein homolog 4.	Nuclear
O43251	[RNA-binding protein 9 (RNA-binding motif protein 9), (Hexaribonucleotide-binding protein 2) (Repressor of tamoxifen, transcriptional activity).]	Nuclear
O43435	[T-box transcription factor TBX1 (T-box protein 1) (Testis-specific T-, box protein).]	Nuclear
O43502	DNA repair protein RAD51 homolog 3 (R51H3) (RAD51-like protein 2).	Nuclear
O43593	Protein hairless.	Nuclear
O43781	[Dual specificity tyrosine-phosphorylation-regulated kinase 3, (EC 2.7.12.1) (Regulatory erythroid kinase) (REDK).]	Nuclear
O43823	A-kinase anchor protein 8 (A-kinase anchor protein 95 kDa) (AKAP 95).	Nuclear
O43913	Origin recognition complex subunit 5.	Nuclear
O60315	[Zinc finger E-box-binding homeobox 2 (Zinc finger homeobox protein 1b), (Smad-interacting protein 1) (SMADIP1).]	Nuclear
O60832	[H/ACA ribonucleoprotein complex subunit 4 (EC 5.4.99.-) (Dyskerin), (Nucleolar protein family A member 4) (snoRNP protein DKC1) (Nopp140-, associated protein of 57 kDa) (Nucleolar protein NAP57) (CBF5, homolog).]	Nuclear
O60942	[mRNA-capping enzyme (HCE) (HCAP1) [Includes: Polynucleotide 5'-, triphosphatase (EC 3.1.3.33) (mRNA 5'-triphosphatase) (TPase); mRNA, guanylyltransferase (EC 2.7.7.50) (GTP--RNA guanylyltransferase), (GTase)].]	Nuclear
O75419	CDC45-related protein (PORC-PI-1) (Cdc45).	Nuclear
P09629	Homeobox protein Hox-B7 (Hox-2C) (HHO.C1).	Nuclear
P09874	[Poly [ADP-ribose] polymerase 1 (EC 2.4.2.30) (PARP-1) (ADPRT) (NAD(+), ADP-ribosyltransferase 1) (Poly[ADP-ribose] synthetase 1).]	Nuclear

P11387	DNA topoisomerase 1 (EC 5.99.1.2) (DNA topoisomerase I).	Nuclear
P11474	[Steroid hormone receptor ERR1 (Estrogen-related receptor, alpha) (ERR-, alpha) (Estrogen receptor-like 1).]	Nuclear
P14635	G2/mitotic-specific cyclin-B1.	Nuclear
P15822	[Zinc finger protein 40 (Human immunodeficiency virus type I enhancer-, binding protein 1) (HIV-EP1) (Major histocompatibility complex-binding, protein 1) (MBP-1) (Positive regulatory domain II-binding factor 1), (PRDII-BF1).]	Nuclear
P15927	[Replication protein A 32 kDa subunit (RP-A) (RF-A) (Replication, factor-A protein 2) (p32) (p34).]	Nuclear
P16220	cAMP response element-binding protein (CREB).	Nuclear
P17029	[Zinc finger protein with KRAB and SCAN domains 1 (Zinc finger protein, 36) (Zinc finger protein KOX18).]	Nuclear
P17038	[Zinc finger protein 43 (Zinc protein HTF6) (Zinc finger protein, KOX27).]	Nuclear
P17480	[Nucleolar transcription factor 1 (Upstream-binding factor 1) (UBF-1), (Autoantigen NOR-90).]	Nuclear
P19388	[DNA-directed RNA polymerases I, II, and III subunit RPABC1 (RNA, polymerases I, II, and III subunit ABC1) (DNA-directed RNA polymerase, II subunit E) (RPB5) (DNA-directed RNA polymerase II 23 kDa, polypeptide) (XAP4).]	Nuclear
P42166	[Lamina-associated polypeptide 2 isoform alpha (Thymopoietin isoform, alpha) (TP alpha) (Thymopoietin-related peptide isoform alpha) (TPRP, isoform alpha) [Contains: Thymopoietin (TP) (Splenin); Thymopentin, (TP5)].]	Nuclear
P48436	Transcription factor SOX-9.	Nuclear
P49321	Nuclear autoantigenic sperm protein (NASP).	Nuclear
P49711	[Transcriptional repressor CTCF (CCCTC-binding factor) (CTCFL paralog), (11-zinc finger protein).]	Nuclear
P51814	Zinc finger protein 41.	Nuclear
P52756	[RNA-binding protein 5 (RNA-binding motif protein 5) (Tumor suppressor, LUCA15) (Protein G15) (Renal carcinoma antigen NY-REN-9).]	Nuclear
P53805	[Calcipressin-1 (Regulator of calcineurin 1) (Down syndrome critical, region protein 1) (Myocyte-enriched calcineurin-interacting protein 1), (MCIP1) (Adapt78).]	Nuclear
P54198	Protein HIRA (TUP1-like enhancer of split protein 1).	Nuclear
P54274	[Telomeric repeat-binding factor 1 (TTAGGG repeat-binding factor 1), (NIMA-interacting protein 2) (Telomeric protein Pin2/TRF1).]	Nuclear
P55201	[Peregrin (Bromodomain and PHD finger-containing protein 1) (BR140, protein).]	Nuclear
Q13595	Transformer-2 protein homolog (TRA-2 alpha).	Nuclear
Q13838	[Spliceosome RNA helicase BAT1 (EC 3.6.1.-) (DEAD box protein UAP56), (56 kDa U2AF65-associated protein) (ATP-dependent RNA helicase p47), (HLA-B-associated transcript-1).]	Nuclear
Q13891	[Transcription factor BTF3 homolog 2 (Basic transcription factor 3-like, 2).]	Nuclear
Q14103	[Heterogeneous nuclear ribonucleoprotein D0 (hnRNP D0) (AU-rich element, RNA-binding protein 1).]	Nuclear
Q14690	RRP5 protein homolog (Programmed cell death protein 11).	Nuclear
Q15233	[Non-POU domain-containing octamer-binding protein (NonO protein) (54, kDa nuclear RNA- and DNA-binding protein) (p54(nrb)) (p54nrb) (55 kDa, nuclear protein) (NMT55) (DNA-binding p52/p100 complex, 52 kDa, subunit).]	Nuclear
Q15326	[Zinc finger MYND domain-containing protein 11 (Adenovirus 5 E1A-, binding protein) (BS69 protein).]	Nuclear
Q15562	[Transcriptional enhancer factor TEF-4 (TEA domain family member 2), (TEAD-2).]	Nuclear
Q15596	[Nuclear receptor coactivator 2 (NCoA-2) (Transcriptional intermediary, factor 2).]	Nuclear
Q92481	[Transcription factor AP-2 beta (AP2-beta) (Activating enhancer-binding, protein 2 beta).]	Nuclear
Q92570	[Orphan nuclear receptor NR4A3 (Nuclear hormone receptor NOR-1), (Neuron-derived orphan receptor 1) (Mitogen-induced nuclear orphan, receptor).]	Nuclear

Q92620	[Pre-mRNA-splicing factor ATP-dependent RNA helicase PRP16 (EC 3.6.1.-), (ATP-dependent RNA helicase DHX38) (DEAH box protein 38).]	Nuclear
Q92791	Nucleolar autoantigen No55.	Nuclear
Q92841	[Probable ATP-dependent RNA helicase DDX17 (EC 3.6.1.-) (DEAD box, protein 17) (RNA-dependent helicase p72) (DEAD box protein p72).]	Nuclear
Q92879	[CUG-BP- and ETR-3-like factor 1 (CELF-1) (Bruno-like protein 2) (RNA-, binding protein BRUNOL-2) (CUG triplet repeat RNA-binding protein 1), (CUG-BP1) (Deadenylation factor CUG-BP) (50 kDa nuclear polyadenylated, RNA-binding protein) (Embryo deadenylation element-binding protein, homolog) (EDEN-BP homolog).]	Nuclear
O95602	[DNA-directed RNA polymerase I subunit RPA1 (EC 2.7.7.6) (DNA-directed, RNA polymerase I largest subunit) (RNA polymerase I 194 kDa subunit), (RPA194) (A190).]	Nuclear
O95751	Protein LDOC1 (Leucine zipper protein down-regulated in cancer cells).	Nuclear
P25786	[Proteasome subunit alpha type-1 (EC 3.4.25.1) (Proteasome component, C2) (Macropain subunit C2) (Multicatalytic endopeptidase complex, subunit C2) (Proteasome nu chain) (30 kDa prosomal protein) (PROS-30).]	Nuclear
P01100	[Proto-oncogene protein c-fos (Cellular oncogene fos) (G0/G1 switch, regulatory protein 7).]	Nuclear
P22415	Upstream stimulatory factor 1 (Major late transcription factor 1).	Nuclear
P24864	G1/S-specific cyclin-E1.	Nuclear
P26358	[DNA (cytosine-5)-methyltransferase 1 (EC 2.1.1.37) (Dnmt1) (DNA, methyltransferase HsaI) (DNA MTase HsaI) (MCMT) (M.HsaI).]	Nuclear
P26599	[Polypyrimidine tract-binding protein 1 (PTB) (Heterogeneous nuclear, ribonucleoprotein I) (hnRNP I) (57 kDa RNA-binding protein PPTB-1).]	Nuclear
P31269	Homeobox protein Hox-A9 (Hox-1G).	Nuclear
P33240	[Cleavage stimulation factor 64 kDa subunit (CSTF 64 kDa subunit) (CF-1, 64 kDa subunit) (CstF-64).]	Nuclear
P33992	DNA replication licensing factor MCM5 (CDC46 homolog) (P1-CDC46).	Nuclear
P35275	Zinc finger protein 126 (HZF-2) (Fragment).	Nuclear
P35637	[RNA-binding protein FUS (Oncogene FUS) (Oncogene TLS) (Translocated in, liposarcoma protein) (POMp75) (75 kDa DNA-pairing protein).]	Nuclear
P38159	[Heterogeneous nuclear ribonucleoprotein G (hnRNP G) (RNA-binding motif, protein, X chromosome) (Glycoprotein p43) [Contains: Processed, heterogeneous nuclear ribonucleoprotein G].]	Nuclear
P58012	Forkhead box protein L2.	Nuclear
P61326	Protein mago nashi homolog.	Nuclear
P62316	Small nuclear ribonucleoprotein Sm D2 (snRNP core protein D2) (Sm-D2).	Nuclear
P78332	[RNA-binding protein 6 (RNA-binding motif protein 6) (RNA-binding, protein DEF-3) (Lung cancer antigen NY-LU-12) (Protein G16).]	Nuclear
P98168	Zinc finger X-linked protein ZXDA.	Nuclear
P98175	[RNA-binding protein 10 (RNA-binding motif protein 10) (G patch domain-, containing protein 9).]	Nuclear
Q02078	[Myocyte-specific enhancer factor 2A (Serum response factor-like, protein 1).]	Nuclear
Q03111	Protein ENL (YEATS domain-containing protein 1).	Nuclear
Q03164	Zinc finger protein HRX (ALL-1) (Trithorax-like protein).	Nuclear
Q03924	[Zinc finger protein 117 (Zinc finger protein HPF9) (Provirus-linked, krueppel) (h-PLK).]	Nuclear
Q04741	[Homeobox protein EMX1 (Empty spiracles homolog 1) (Empty spiracles-, like protein 1).]	Nuclear
Q06587	[E3 ubiquitin-protein ligase RING1 (EC 6.3.2.-) (Polycomb complex, protein RING1) (RING finger protein 1).]	Nuclear

Q06732	[Zinc finger protein 33B (Zinc finger protein 11B) (Zinc finger protein, KOX2).]	Nuclear
Q07864	[DNA polymerase epsilon catalytic subunit A (EC 2.7.7.7) (DNA, polymerase II subunit A).]	Nuclear
Q09161	[Nuclear cap-binding protein subunit 1 (80 kDa nuclear cap-binding, protein) (NCBP 80 kDa subunit) (CBP80).]	Nuclear
Q12873	[Chromodomain-helicase-DNA-binding protein 3 (EC 3.6.1.-) (ATP-, dependent helicase CHD3) (CHD-3) (Mi-2 autoantigen 240 kDa protein), (Mi2-alpha) (Zinc finger helicase) (hZFH).]	Nuclear
Q12952	[Forkhead box protein L1 (Forkhead-related protein FKHL11) (Forkhead-, related transcription factor 7) (FREAC-7).]	Nuclear
Q13023	[A-kinase anchor protein 6 (Protein kinase A-anchoring protein 6), (PRKA6) (A-kinase anchor protein 100 kDa) (AKAP 100) (mAKAP).]	Nuclear
Q13185	[Chromobox protein homolog 3 (Heterochromatin protein 1 homolog gamma), (HP1 gamma) (Modifier 2 protein) (HECH).]	Nuclear
Q13216	[DNA excision repair protein ERCC-8 (Cockayne syndrome WD repeat, protein CSA).]	Nuclear
Q13342	[Nuclear body protein SP140 (Nuclear autoantigen Sp-140) (Speckled 140, kDa) (LYSp100 protein) (Lymphoid-restricted homolog of Sp100).]	Nuclear
Q93009	[Ubiquitin carboxyl-terminal hydrolase 7 (EC 3.1.2.15) (Ubiquitin, thioesterase 7) (Ubiquitin-specific-processing protease 7), (Deubiquitinating enzyme 7) (Herpesvirus-associated ubiquitin-specific, protease).]	Nuclear
Q99490	[Centaurin-gamma 1 (ARF-GAP with GTP-binding protein-like, ankyrin, repeat and pleckstrin homology domains 2) (AGAP-2), (Phosphatidylinositol-3-kinase enhancer) (PIKE) (GTP-binding and, GTPase-activating protein 2) (GGAP2).]	Nuclear
Q99543	[DnaJ homolog subfamily C member 2 (Zuotin-related factor 1) (M-phase, phosphoprotein 11).]	Nuclear
Q9H161	Homeobox protein aristaless-like 4.	Nuclear
Q9NRM2	Zinc finger protein 277.	Nuclear
Q9NSI6	[Bromodomain and WD repeat-containing protein 1 (WD repeat-containing, protein 9).]	Nuclear
Q9P2Y4	Zinc finger protein 219.	Nuclear
Q9UBP0	Spastin.	Nuclear
Q9UKK3	[Poly [ADP-ribose] polymerase 4 (EC 2.4.2.30) (PARP-4) (Vault poly(ADP-, ribose) polymerase) (VPARP) (193 kDa vault protein) (PARP-, related/IalphaI-related H5/proline-rich) (PH5P).]	Nuclear
Q9UL17	[T-box transcription factor TBX21 (T-box protein 21) (Transcription, factor TBLYM) (T-cell-specific T-box transcription factor T-bet).]	Nuclear
Q9UL58	[Zinc finger protein 215 (BWSCR2-associated zinc finger protein 2) (BAZ, 2) (Zinc finger protein with KRAB and SCAN domains 11).]	Nuclear
Q9UNX4	WD repeat-containing protein 3.	Nuclear
Q9Y2G3	[Probable phospholipid-transporting ATPase IF (EC 3.6.3.1) (ATPase, class I type 11B) (ATPase IR).]	Nuclear
Q9Y2X3	Nucleolar protein 5 (Nucleolar protein NOP5) (NOP58).	Nuclear
O00505	Importin subunit alpha-3 (Karyopherin subunit alpha-3) (SRP1-gamma).	Nuclear Membrane
P57740	[Nuclear pore complex protein Nup107 (Nucleoporin Nup107) (107 kDa, nucleoporin).]	Nuclear Membrane
Q12769	[Nuclear pore complex protein Nup160 (Nucleoporin Nup160) (160 kDa, nucleoporin).]	Nuclear Membrane
Q9BTX1	Nucleoporin NDC1 (hNDC1) (Transmembrane protein 48).	Nuclear Membrane
Q9H2T7	Ran-binding protein 17.	Nuclear Membrane
P49792	[E3 SUMO-protein ligase RanBP2 (Ran-binding protein 2) (Nuclear pore, complex protein Nup358) (Nucleoporin Nup358) (358 kDa nucleoporin), (p270).]	Nuclear Membrane
P37802	Transgelin-2 (SM22-alpha homolog).	Nuclear Membrane

P63165	[Small ubiquitin-related modifier 1 precursor (SUMO-1) (Sentrin), (Ubiquitin-like protein SMT3C) (SMT3 homolog 3) (Ubiquitin-homology, domain protein PIC1) (Ubiquitin-like protein UBL1) (GAP-modifying, protein 1) (GMP1).]	Nuclear Membrane
P52594	[Nucleoporin-like protein RIP (HIV-1 Rev-binding protein) (Rev-, interacting protein) (Rev/Rex activation domain-binding protein).]	Nuclear Membrane
P52948	[Nuclear pore complex protein Nup98-Nup96 precursor [Contains: Nuclear, pore complex protein Nup98 (Nucleoporin Nup98) (98 kDa nucleoporin);, Nuclear pore complex protein Nup96 (Nucleoporin Nup96) (96 kDa, nucleoporin)].]	Nuclear Membrane
O15182	Centrin-3.	Other Filament or Tubule
O43182	[Rho GTPase-activating protein 6 (Rho-type GTPase-activating protein, RhoGAPX-1).]	Other Filament or Tubule
O43303	Centrosomal protein of 110 kDa (Cep110 protein).	Other Filament or Tubule
O43903	Growth arrest-specific protein 2 (GAS-2).	Other Filament or Tubule
P08729	[Keratin, type II cytoskeletal 7 (Cytokeratin-7) (CK-7) (Keratin-7), (K7) (Sarcolectin).]	Other Filament or Tubule
P08779	[Keratin, type I cytoskeletal 16 (Cytokeratin-16) (CK-16) (Keratin-16), (K16).]	Other Filament or Tubule
P13647	[Keratin, type II cytoskeletal 5 (Cytokeratin-5) (CK-5) (Keratin-5), (K5) (58 kDa cytokeratin).]	Other Filament or Tubule
P14136	Glial fibrillary acidic protein (GFAP).	Other Filament or Tubule
P17661	Desmin.	Other Filament or Tubule
P19012	[Keratin, type I cytoskeletal 15 (Cytokeratin-15) (CK-15) (Keratin-15), (K15).]	Other Filament or Tubule
P46940	Ras GTPase-activating-like protein IQGAP1 (p195).	Other Filament or Tubule
P48681	Nestin.	Other Filament or Tubule
Q14008	[Cytoskeleton-associated protein 5 (Colonic and hepatic tumor over-, expressed protein) (Ch-TOG protein).]	Other Filament or Tubule
P02533	[Keratin, type I cytoskeletal 14 (Cytokeratin-14) (CK-14) (Keratin-14), (K14).]	Other Filament or Tubule
P05787	[Keratin, type II cytoskeletal 8 (Cytokeratin-8) (CK-8) (Keratin-8), (K8).]	Other Filament or Tubule
P40121	Macrophage-capping protein (Actin regulatory protein CAP-G).	Other Filament or Tubule
P41219	Peripherin.	Other Filament or Tubule
Q04695	[Keratin, type I cytoskeletal 17 (Cytokeratin-17) (CK-17) (Keratin-17), (K17) (39.1).]	Other Filament or Tubule
Q99996	[A-kinase anchor protein 9 (Protein kinase A-anchoring protein 9), (PRKA9) (A-kinase anchor protein 450 kDa) (AKAP 450) (A-kinase anchor, protein 350 kDa) (AKAP 350) (hgAKAP 350) (AKAP 120-like protein), (Protein hyperion) (Protein yotiao) (Centrosome- and Golgi-localized, PKN-associated protein) (CG-NAP).]	Other Filament or Tubule
P13645	[Keratin, type I cytoskeletal 10 (Cytokeratin-10) (CK-10) (Keratin-10), (K10).]	Other Filament or Tubule
P19013	[Keratin, type II cytoskeletal 4 (Cytokeratin-4) (CK-4) (Keratin-4), (K4).]	Other Filament or Tubule
Q13515	[Phakinin (Beaded filament structural protein 2) (Lens fiber cell, beaded filament protein CP 49) (CP49) (49 kDa cytoskeletal protein), (CP 47) (CP47) (Lens intermediate filament-like light) (LIFL-L).]	Other Filament or Tubule
P04264	[Keratin, type II cytoskeletal 1 (Cytokeratin-1) (CK-1) (Keratin-1), (K1) (67 kDa cytokeratin) (Hair alpha protein).]	Other Filament or Tubule
P30260	Cell division cycle protein 27 homolog (CDC27Hs) (H-NUC).	Other Filament or Tubule
P35609	[Alpha-actinin-2 (Alpha-actinin skeletal muscle isoform 2) (F-actin, cross-linking protein).]	Other Filament or Tubule
P68133	Actin, alpha skeletal muscle (Alpha-actin-1).	Other Filament or Tubule
O15392	[Baculoviral IAP repeat-containing protein 5 (Apoptosis inhibitor, survivin) (Apoptosis inhibitor 4).]	Other Filament or Tubule
P12035	[Keratin, type II cytoskeletal 3 (Cytokeratin-3) (CK-3) (Keratin-3), (K3) (65 kDa cytokeratin).]	Other Filament or Tubule
P15311	Ezrin (p81) (Cytovillin) (Villin-2).	Other Filament or Tubule
P20700	Lamin-B1.	Other Filament or Tubule

P47756	F-actin-capping protein subunit beta (CapZ beta).	Other Filament or Tubule
P52179	[Myomesin-1 (190 kDa titin-associated protein) (190 kDa connectin-, associated protein).]	Other Filament or Tubule
P06753	Tropomyosin alpha-3 chain (Tropomyosin-3) (Tropomyosin gamma) (hTM5).	Other Filament or Tubule
P23258	[Tubulin gamma-1 chain (Gamma-1-tubulin) (Gamma-tubulin complex, component 1) (GCP-1).]	Other Filament or Tubule
P41208	Centrin-2 (Caltractin isoform 1).	Other Filament or Tubule
Q01546	[Keratin, type II cytoskeletal 2 oral (Cytokeratin-2P) (K2P) (CK 2P), (Keratin-76).]	Other Filament or Tubule
Q08043	[Alpha-actinin-3 (Alpha-actinin skeletal muscle isoform 3) (F-actin, cross-linking protein).]	Other Filament or Tubule
Q9UJT1	Tubulin delta chain (Delta-tubulin).	Other Filament or Tubule
O00213	[Amyloid beta A4 precursor protein-binding family B member 1 (Fe65, protein).]	Other Go Component
O43684	Mitotic checkpoint protein BUB3.	Other Go Component
O60240	Perilipin (PERI) (Lipid droplet-associated protein).	Other Go Component
O60508	[Pre-mRNA-processing factor 17 (PRP17 homolog) (hPRP17) (Cell division, cycle 40 homolog) (EH-binding protein 3) (Ehb3).]	Other Go Component
P08575	[Leukocyte common antigen precursor (EC 3.1.3.48) (L-CA) (T200) (CD45, antigen).]	Other Go Component
P08621	[U1 small nuclear ribonucleoprotein 70 kDa (U1 snRNP 70 kDa) (snRNP70), (U1-70K).]	Other Go Component
P09012	[U1 small nuclear ribonucleoprotein A (U1 snRNP protein A) (U1A, protein) (U1-A).]	Other Go Component
P10144	[Granzyme B precursor (EC 3.4.21.79) (T-cell serine protease 1-3E), (Cytotoxic T-lymphocyte proteinase 2) (Lymphocyte protease) (SECT), (Granzyme-2) (Cathepsin G-like 1) (CTSGL1) (CTLA-1) (Fragmentin-2), (Human lymphocyte protein) (HLP) (C11).]	Other Go Component
P11532	Dystrophin.	Other Go Component
P12109	Collagen alpha-1(VI) chain precursor.	Other Go Component
P15088	[Mast cell carboxypeptidase A precursor (EC 3.4.17.1) (MC-CPA), (Carboxypeptidase A3).]	Other Go Component
P20849	Collagen alpha-1(IX) chain precursor.	Other Go Component
P43681	Neuronal acetylcholine receptor subunit alpha-4 precursor.	Other Go Component
P51884	[Lumican precursor (Keratan sulfate proteoglycan lumican) (KSPG, lumican).]	Other Go Component
Q13435	[Splicing factor 3B subunit 2 (Spliceosome-associated protein 145) (SAP, 145) (SF3b150) (Pre-mRNA-splicing factor SF3b 145 kDa subunit).]	Other Go Component
Q14562	[ATP-dependent RNA helicase DHX8 (EC 3.6.1.-) (DEAH box protein 8) (RNA, helicase HRH1).]	Other Go Component
Q16363	Laminin subunit alpha-4 precursor.	Other Go Component
O75955	Flotillin-1.	Other Go Component
O96017	Serine/threonine-protein kinase Chk2 (EC 2.7.11.1) (Cds1).	Other Go Component
P00441	Superoxide dismutase [Cu-Zn] (EC 1.15.1.1).	Other Go Component
P01619	Ig kappa chain V-III region B6.	Other Go Component
P02461	Collagen alpha-1(III) chain precursor.	Other Go Component
P22303	Acetylcholinesterase precursor (EC 3.1.1.7) (AChE).	Other Go Component
P30532	Neuronal acetylcholine receptor subunit alpha-5 precursor.	Other Go Component
P32927	[Cytokine receptor common beta chain precursor (GM-CSF/IL-3/IL-5, receptor common beta-chain) (CD131 antigen) (CDw131).]	Other Go Component
P33981	[Dual specificity protein kinase TTK (EC 2.7.12.1) (Phosphotyrosine, picked threonine-protein kinase) (PYT).]	Other Go Component

P98073	[Enteropeptidase precursor (EC 3.4.21.9) (Enterokinase) (Serine, protease 7) [Contains: Enteropeptidase non-catalytic heavy chain;, Enteropeptidase catalytic light chain].]	Other Go Component
Q05586	[Glutamate [NMDA] receptor subunit zeta-1 precursor (N-methyl-D-, aspartate receptor subunit NR1).]	Other Go Component
Q10570	[Cleavage and polyadenylation specificity factor subunit 1 (Cleavage, and polyadenylation specificity factor 160 kDa subunit) (CPSF 160 kDa, subunit).]	Other Go Component
Q13257	[Mitotic spindle assembly checkpoint protein MAD2A (MAD2-like 1), (HsMAD2).]	Other Go Component
Q99715	Collagen alpha-1(XII) chain precursor.	Other Go Component
Q9H1Y0	Autophagy protein 5 (APG5-like) (Apoptosis-specific protein).	Other Go Component
O43143	[Putative pre-mRNA-splicing factor ATP-dependent RNA helicase DHX15, (EC 3.6.1.-) (DEAH box protein 15) (ATP-dependent RNA helicase #46).]	Other Go Component
O43561	[Linker for activation of T-cells family member 1 (36 kDa phospho-, tyrosine adapter protein) (pp36) (p36-38).]	Other Go Component
O60313	[Dynamin-like 120 kDa protein, mitochondrial precursor (Optic atrophy, protein 1) [Contains: Dynamin-like 120 kDa protein, form S1].]	Other Go Component
O75643	[U5 small nuclear ribonucleoprotein 200 kDa helicase (EC 3.6.1.-) (U5, snRNP-specific 200 kDa protein) (U5-200KD) (Activating signal, cointegrator 1 complex subunit 3-like 1) (BRR2 homolog).]	Other Go Component
O75683	Surfeit locus protein 6.	Other Go Component
P09661	U2 small nuclear ribonucleoprotein A' (U2 snRNP-A').	Other Go Component
P12814	[Alpha-actinin-1 (Alpha-actinin cytoskeletal isoform) (Non-muscle, alpha-actinin-1) (F-actin cross-linking protein).]	Other Go Component
P15924	Desmoplakin (DP) (250/210 kDa paraneoplastic pemphigus antigen).	Other Go Component
P18206	Vinculin (Metavinculin).	Other Go Component
P55268	Laminin subunit beta-2 precursor (S-laminin) (Laminin B1s chain).	Other Go Component
Q14031	Collagen alpha-6(IV) chain precursor.	Other Go Component
Q14807	[Kinesin-like protein KIF22 (Kinesin-like DNA-binding protein), (Kinesin-like protein 4).]	Other Go Component
Q15696	[U2 small nuclear ribonucleoprotein auxiliary factor 35 kDa subunit-, related protein 2 (U2(RNU2) small nuclear RNA auxiliary factor 1-like, 2) (CCCH type zinc finger, RNA-binding motif and serine/arginine rich, protein 2) (Renal carcinoma antigen NY-REN-20).]	Other Go Component
Q15942	Zyxin (Zyxin-2).	Other Go Component
Q7RTV0	[PHD finger-like domain-containing protein 5A (PHD finger-like domain, protein 5A) (Splicing factor 3B-associated 14 kDa protein) (SF3b14b).]	Other Go Component
Q92817	[Envoplakin (210 kDa paraneoplastic pemphigus antigen) (p210) (210 kDa, cornified envelope precursor protein).]	Other Go Component
P01157	Growth-modulating peptide.	Other Go Component
P04083	[Annexin A1 (Annexin-1) (Annexin I) (Lipocortin I) (Calpactin II), (Chromobindin-9) (p35) (Phospholipase A2 inhibitory protein).]	Other Go Component
P05198	[Eukaryotic translation initiation factor 2 subunit 1 (Eukaryotic, translation initiation factor 2 subunit alpha) (eIF-2-alpha) (EIF-, 2alpha) (EIF-2A).]	Other Go Component
P05997	Collagen alpha-2(V) chain precursor.	Other Go Component
P22735	[Protein-glutamine gamma-glutamyltransferase K (EC 2.3.2.13), (Transglutaminase K) (TGase K) (TGK) (TG(K)) (Transglutaminase-1), (Epidermal TGase).]	Other Go Component
P29400	Collagen alpha-5(IV) chain precursor.	Other Go Component
P35663	Cylicin-1 (Cylicin I) (Multiple-band polypeptide I).	Other Go Component
P39059	[Collagen alpha-1(XV) chain precursor [Contains: Endostatin, (Endostatin-XV) (Restin) (Related to endostatin)].]	Other Go Component

P63279	[SUMO-conjugating enzyme UBC9 (EC 6.3.2.-) (SUMO-protein ligase), (Ubiquitin-conjugating enzyme E2 I) (Ubiquitin-protein ligase I), (Ubiquitin carrier protein I) (Ubiquitin carrier protein 9) (p18).]	Other Go Component
P82932	Mitochondrial 28S ribosomal protein S6 (S6mt) (MRP-S6).	Other Go Component
P98082	Disabled homolog 2 (Differentially-expressed protein 2) (DOC-2).	Other Go Component
Q01955	[Collagen alpha-3(IV) chain precursor (Goodpasture antigen) [Contains:, Tumstatin].]	Other Go Component
Q07001	Acetylcholine receptor subunit delta precursor.	Other Go Component
Q07092	Collagen alpha-1(XVI) chain precursor.	Other Go Component
Q9NZU7	Calcium-binding protein 1 (CaBP1) (Calbrain).	Other Go Component
Q9UJT0	Tubulin epsilon chain (Epsilon-tubulin).	Other Go Component
Q9Y2I7	[FYVE finger-containing phosphoinositide kinase (EC 2.7.1.68) (1-, phosphatidylinositol-4-phosphate 5-kinase) (Phosphatidylinositol-3-, phosphate 5-kinase type III) (PIP5K) (PtdIns(4)P-5-kinase) (PIKfyve), (p235).]	Other Go Component
O00255	Menin.	Other Go Component
O43683	[Mitotic checkpoint serine/threonine-protein kinase BUB1 (EC 2.7.11.1), (hBUB1) (BUB1A).]	Other Go Component
O75533	[Splicing factor 3B subunit 1 (Spliceosome-associated protein 155) (SAP, 155) (SF3b155) (Pre-mRNA-splicing factor SF3b 155 kDa subunit).]	Other Go Component
P08572	Collagen alpha-2(IV) chain precursor [Contains: Canstatin].	Other Go Component
P12107	Collagen alpha-1(XI) chain precursor.	Other Go Component
P12111	Collagen alpha-3(VI) chain precursor.	Other Go Component
P13942	Collagen alpha-2(XI) chain precursor.	Other Go Component
P19021	[Peptidyl-glycine alpha-amidating monooxygenase precursor (PAM), [Includes: Peptidylglycine alpha-hydroxylating monooxygenase, (EC 1.14.17.3) (PHM); Peptidyl-alpha-hydroxyglycine alpha-amidating, lyase (EC 4.3.2.5) (Peptidylamidoglycolate lyase) (PAL)].]	Other Go Component
P20908	Collagen alpha-1(V) chain precursor.	Other Go Component
P42566	[Epidermal growth factor receptor substrate 15 (Protein Eps15) (AF-1p, protein).]	Other Go Component
P53420	Collagen alpha-4(IV) chain precursor.	Other Go Component
P55344	Lens fiber membrane intrinsic protein (MP18) (MP19) (MP20).	Other Go Component
P56856	Claudin-18.	Other Go Component
Q13946	[High affinity cAMP-specific 3',5'-cyclic phosphodiesterase 7A, (EC 3.1.4.17) (HCP1) (TM22).]	Other Go Component
Q14993	Collagen alpha-1(XIX) chain precursor (Collagen alpha-1(Y) chain).	Other Go Component
Q15459	[Splicing factor 3 subunit 1 (Spliceosome-associated protein 114) (SAP, 114) (SF3a120).]	Other Go Component
Q16643	Drebrin (Developmentally-regulated brain protein).	Other Go Component
P01303	[Neuropeptide Y precursor [Contains: Neuropeptide Y (Neuropeptide, tyrosine) (NPY); C-flanking peptide of NPY (CPON)].]	Other Go Component
P02549	Spectrin alpha chain, erythrocyte (Erythroid alpha-spectrin).	Other Go Component
P05408	[Neuroendocrine protein 7B2 precursor (Secretogranin-5) (Secretogranin, V) (Secretory granule endocrine protein I) (Pituitary polypeptide), [Contains: N-terminal peptide; C-terminal peptide].]	Other Go Component
P23497	[Nuclear autoantigen Sp-100 (Speckled 100 kDa) (Nuclear dot-associated, Sp100 protein) (Lysp100b).]	Other Go Component
P24534	Elongation factor 1-beta (EF-1-beta).	Other Go Component
P27658	Collagen alpha-1(VIII) chain precursor (Endothelial collagen).	Other Go Component

P36544	Neuronal acetylcholine receptor subunit alpha-7 precursor.	Other Go Component
P39060	Collagen alpha-1(XVIII) chain precursor [Contains: Endostatin].	Other Go Component
P40429	60S ribosomal protein L13a (23 kDa highly basic protein).	Other Go Component
P60662	[Myosin light polypeptide 6 (Smooth muscle and nonmuscle myosin light, chain alkali 6) (Myosin light chain alkali 3) (Myosin light chain 3), (MLC-3) (LC17).]	Other Go Component
P78369	Claudin-10 (OSP-like protein).	Other Go Component
P98088	[Mucin-5AC (Mucin-5 subtype AC, tracheobronchial) (Tracheobronchial, mucin) (TBM) (Major airway glycoprotein) (Fragment).]	Other Go Component
Q00610	Clathrin heavy chain 1 (CLH-17).	Other Go Component
Q02413	[Desmoglein-1 precursor (Desmosomal glycoprotein 1) (DG1) (DGI), (Pemphigus foliaceus antigen).]	Other Go Component
Q03188	[Centromere protein C 1 (CENP-C) (Centromere autoantigen C) (Interphase, centromere complex protein 7).]	Other Go Component
Q03692	Collagen alpha-1(X) chain precursor.	Other Go Component
Q05901	Neuronal acetylcholine receptor subunit beta-3 precursor.	Other Go Component
Q9UI46	[Dynein intermediate chain 1, axonemal (Axonemal dynein intermediate, chain 1).]	Other Go Component
P51811	[Membrane transport protein XK (Kx antigen) (Kell complex 37 kDa, component) (XK-related protein 1).]	Other Membrane
O00341	[Excitatory amino acid transporter 5 (Solute carrier family 1 member 7), (Retinal glutamate transporter).]	Other Membrane
O00451	[GDNF family receptor alpha-2 precursor (GFR-alpha-2) (Neurturin, receptor alpha) (NTNR-alpha) (NRTNR-alpha) (TGF-beta-related, neurotrophic factor receptor 2) (GDNF receptor beta) (GDNFR-beta) (RET, ligand 2).]	Other Membrane
O00559	[Receptor-binding cancer antigen expressed on SiSo cells (Cancer-, associated surface antigen RCAS1) (Estrogen receptor-binding fragment-, associated gene 9 protein).]	Other Membrane
O15165	Uncharacterized protein C18orf1.	Other Membrane
O15439	[Multidrug resistance-associated protein 4 (ATP-binding cassette sub-, family C member 4) (MRP/cMOAT-related ABC transporter) (Multi-specific, organic anion transporter-B) (MOAT-B).]	Other Membrane
O15482	Testis-specific protein TEX28.	Other Membrane
O43462	[Membrane-bound transcription factor site-2 protease (EC 3.4.24.85), (S2P endopeptidase) (Site-2 protease) (Sterol regulatory element-, binding proteins intramembrane protease).]	Other Membrane
O60312	[Probable phospholipid-transporting ATPase VA (EC 3.6.3.1) (ATPVA), (Aminophospholipid translocase VA).]	Other Membrane
O60840	[Voltage-dependent L-type calcium channel subunit alpha-1F (Voltage-, gated calcium channel subunit alpha Cav1.4).]	Other Membrane
P10643	Complement component C7 precursor.	Other Membrane
P10696	[Alkaline phosphatase, placental-like precursor (EC 3.1.3.1) (Alkaline, phosphatase Nagao isozyme) (Germ cell alkaline phosphatase) (GCAP), (PLAP-like) (ALP-1).]	Other Membrane
P13761	[HLA class II histocompatibility antigen, DRB1-7 beta chain precursor, (MHC class I antigen DRB1*7) (DR-7) (DR7).]	Other Membrane
P46531	[Neurogenic locus notch homolog protein 1 precursor (Notch 1) (hN1), (Translocation-associated notch protein TAN-1) [Contains: Notch 1, extracellular truncation; Notch 1 intracellular domain].]	Other Membrane
P48029	[Sodium- and chloride-dependent creatine transporter 1 (CT1) (Creatine, transporter 1) (Solute carrier family 6 member 8).]	Other Membrane
P50542	[Peroxisomal targeting signal 1 receptor (Peroxisome receptor 1), (Peroxisomal C-terminal targeting signal import receptor) (PTS1-BP), (Peroxin-5) (PTS1 receptor).]	Other Membrane
P54756	[Ephrin type-A receptor 5 precursor (EC 2.7.10.1) (Tyrosine-protein, kinase receptor EHK-1) (EPH homology kinase 1) (Receptor protein-, tyrosine kinase HEK7).]	Other Membrane

P56377	[AP-1 complex subunit sigma-2 (Adapter-related protein complex 1 sigma-, 1B subunit) (Sigma-adaptin 1B) (Adaptor protein complex AP-1 sigma-1B, subunit) (Golgi adaptor HA1/AP1 adaptin sigma-1B subunit) (Clathrin, assembly protein complex 1 sigma-1B small chain) (Sigma 1B subunit of, AP-1 clathrin).]	Other Membrane
Q14330	[N-arachidonyl glycine receptor (NAGly receptor) (G-protein coupled, receptor 18).]	Other Membrane
Q14940	[Sodium/hydrogen exchanger 5 (Na(+)/H(+) exchanger 5) (NHE-5) (Solute, carrier family 9 member 5).]	Other Membrane
Q15286	Ras-related protein Rab-35 (Rab-1C) (GTP-binding protein RAY).	Other Membrane
Q15391	[P2Y purinoceptor 14 (P2Y14) (UDP-glucose receptor) (G-protein coupled, receptor 105).]	Other Membrane
Q15849	Urea transporter, kidney.	Other Membrane
Q16478	[Glutamate receptor, ionotropic kainate 5 precursor (Glutamate receptor, KA-2) (KA2) (Excitatory amino acid receptor 2) (EAA2).]	Other Membrane
Q16787	[Laminin subunit alpha-3 precursor (Epiligrin 170 kDa subunit) (E170), (Nicein subunit alpha).]	Other Membrane
Q16864	[Vacuolar ATP synthase subunit F (EC 3.6.3.14) (V-ATPase subunit F), (Vacuolar proton pump subunit F) (V-ATPase 14 kDa subunit).]	Other Membrane
Q6UWL6	[Kin of IRRE-like protein 2 precursor (Kin of irregular chiasm-like, protein 2) (Nephrin-like protein 3).]	Other Membrane
P01860	Ig gamma-3 chain C region (Heavy chain disease protein) (HDC).	Other Membrane
P03989	[HLA class I histocompatibility antigen, B-27 alpha chain precursor, (MHC class I antigen B*27).]	Other Membrane
P04233	[HLA class II histocompatibility antigen gamma chain (HLA-DR antigens-, associated invariant chain) (Ia antigen-associated invariant chain), (Ii) (p33) (CD74 antigen).]	Other Membrane
P04920	[Anion exchange protein 2 (Non-erythroid band 3-like protein) (AE2, anion exchanger) (Solute carrier family 4 member 2) (BND3L).]	Other Membrane
P07358	[Complement component C8 beta chain precursor (Complement component 8, subunit beta).]	Other Membrane
P22748	[Carbonic anhydrase 4 precursor (EC 4.2.1.1) (Carbonic anhydrase IV), (Carbonate dehydratase IV) (CA-IV).]	Other Membrane
P25067	Collagen alpha-2(VIII) chain precursor (Endothelial collagen).	Other Membrane
P26992	Ciliary neurotrophic factor receptor alpha precursor (CNTFR alpha).	Other Membrane
P31995	[Low affinity immunoglobulin gamma Fc region receptor II-c precursor, (Fc-gamma RII-c) (FcRII-c) (IgG Fc receptor II-c) (Fc-gamma-RIIc), (CD32 antigen) (CDw32).]	Other Membrane
P35410	Mas-related G-protein coupled receptor MRG (MAS-R) (MAS1-like).	Other Membrane
P60763	Ras-related C3 botulinum toxin substrate 3 precursor (p21-Rac3).	Other Membrane
Q00973	[Beta-1,4 N-acetylgalactosaminyltransferase 1 (EC 2.4.1.92) ((N-, acetylneuraminyl)-galactosylglucosylceramide) (GM2/GD2 synthase), (GalNAc-T).]	Other Membrane
Q13061	Triadin.	Other Membrane
Q99848	[Probable rRNA-processing protein EBP2 (EBNA1-binding protein 2), (Nucleolar protein p40).]	Other Membrane
Q9GZZ6	[Neuronal acetylcholine receptor subunit alpha-10 precursor (Nicotinic, acetylcholine receptor subunit alpha 10) (NACHR alpha 10).]	Other Membrane
Q9NRS4	[Transmembrane protease, serine 4 (EC 3.4.21.-) (Membrane-type serine, protease 2) (MT-SP2).]	Other Membrane
Q9NZW5	MAGUK p55 subfamily member 6 (Veli-associated MAGUK 1) (VAM-1).	Other Membrane
O00623	[Peroxisome assembly protein 12 (Peroxin-12) (Peroxisome assembly, factor 3) (PAF-3).]	Other Membrane
O15259	Nephrocystin-1 (Juvenile nephronophthisis 1 protein).	Other Membrane
O15394	Neural cell adhesion molecule 2 precursor (N-CAM 2).	Other Membrane
O43427	[Acidic fibroblast growth factor intracellular-binding protein (aFGF, intracellular-binding protein) (FGF-1 intracellular-binding protein).]	Other Membrane

O60241	Brain-specific angiogenesis inhibitor 2 precursor.	Other Membrane
O60779	[Thiamine transporter 1 (ThTr-1) (ThTr1) (Thiamine carrier 1) (TC1), (Solute carrier family 19 member 2).]	Other Membrane
O75144	[ICOS ligand precursor (B7 homolog 2) (B7-H2) (B7-like protein Gl50), (B7-related protein 1) (B7RP-1) (CD275 antigen).]	Other Membrane
P08240	[Signal recognition particle receptor subunit alpha (SR-alpha) (Docking, protein alpha) (DP-alpha).]	Other Membrane
P08922	[Proto-oncogene tyrosine-protein kinase ROS precursor (EC 2.7.10.1) (c-, ros-1).]	Other Membrane
P12830	[Epithelial cadherin precursor (E-cadherin) (Uvomorulin) (Cadherin-1), (CAM 120/80) (CD324 antigen) [Contains: E-Cad/CTF1; E-Cad/CTF2; E-, Cad/CTF3].]	Other Membrane
P13591	[Neural cell adhesion molecule 1, 140 kDa isoform precursor (N-CAM 140), (NCAM-140) (CD56 antigen).]	Other Membrane
P21217	[Galactoside 3(4)-L-fucosyltransferase (EC 2.4.1.65) (Blood group Lewis, alpha-4-fucosyltransferase) (Lewis FT) (Fucosyltransferase 3) (FucT-, III).]	Other Membrane
P42081	[T-lymphocyte activation antigen CD86 precursor (Activation B7-2, antigen) (CTLA-4 counter-receptor B7.2) (B70) (FUN-1) (BU63).]	Other Membrane
P43003	[Excitatory amino acid transporter 1 (Solute carrier family 1 member 3), (Sodium-dependent glutamate/aspartate transporter 1) (GLAST-1).]	Other Membrane
P43220	[Glucagon-like peptide 1 receptor precursor (GLP-1 receptor) (GLP-1-R), (GLP-1R).]	Other Membrane
P43631	[Killer cell immunoglobulin-like receptor 2DS2 precursor (MHC class I, NK cell receptor) (Natural killer-associated transcript 5) (NKAT-5), (p58 natural killer cell receptor clone CL-49) (p58 NK receptor) (NK, receptor 183 ActI) (CD158j antigen).]	Other Membrane
P48065	[Sodium- and chloride-dependent betaine transporter (Na(+)/Cl(-), betaine/GABA transporter) (BGT-1).]	Other Membrane
P51805	Plexin-A3 precursor (Plexin-4) (Semaphorin receptor SEX).	Other Membrane
P51843	[Nuclear receptor 0B1 (Nuclear receptor DAX-1) (DSS-AHC critical region, on the X chromosome protein 1).]	Other Membrane
P53985	[Monocarboxylate transporter 1 (MCT 1) (Solute carrier family 16 member, 1).]	Other Membrane
P54829	[Tyrosine-protein phosphatase non-receptor type 5 (EC 3.1.3.48), (Protein-tyrosine phosphatase striatum-enriched) (STEP) (Neural-, specific protein-tyrosine phosphatase).]	Other Membrane
P56159	[GDNF family receptor alpha-1 precursor (GFR-alpha-1) (GDNF receptor, alpha) (GDNFR-alpha) (TGF-beta-related neurotrophic factor receptor 1), (RET ligand 1).]	Other Membrane
Q13505	Metaxin-1.	Other Membrane
Q13795	[ADP-ribosylation factor-related protein 1 (ARF-related protein 1), (ARP).]	Other Membrane
Q14210	Lymphocyte antigen 6D precursor (Ly-6D) (E48 antigen).	Other Membrane
Q15491	Neuregulin-1, sensory and motor neuron-derived factor isoform.	Other Membrane
Q16739	[Ceramide glucosyltransferase (EC 2.4.1.80) (Glucosylceramide synthase), (GCS) (UDP-glucose:N-acylsphingosine D-glucosyltransferase) (UDP-, glucose ceramide glucosyltransferase) (GLCT-1).]	Other Membrane
Q92911	[Sodium/iodide cotransporter (Na(+)/I(-) cotransporter) (Sodium-iodide, symporter) (Na(+)/I(-)-symporter).]	Other Membrane
Q92968	Peroxisomal membrane protein PEX13 (Peroxin-13).	Other Membrane
O76024	Wolframin.	Other Membrane
P01857	Ig gamma-1 chain C region.	Other Membrane
P01861	Ig gamma-4 chain C region.	Other Membrane
P01919	[HLA class II histocompatibility antigen, DQ(W1.1) beta chain precursor, (DQB1*0501).]	Other Membrane

P22413	[Ectonucleotide pyrophosphatase/phosphodiesterase family member 1 (E-, NPP 1) (Phosphodiesterase I/nucleotide pyrophosphatase 1) (Plasma-cell, membrane glycoprotein PC-1) [Includes: Alkaline phosphodiesterase I, (EC 3.1.4.1); Nucleotide pyrophosphatase (EC 3.6.1.9) (NPPase)].]	Other Membrane
P25089	FMLP-related receptor II (FMLP-R-II) (Formylpeptide receptor-like 2).	Other Membrane
P26439	[3 beta-hydroxysteroid dehydrogenase/Delta 5-->4-isomerase type 2 (3-, beta-HSD II) [Includes: 3-beta-hydroxy-Delta(5)-steroid dehydrogenase, (EC 1.1.1.145) (3-beta-hydroxy-5-ene steroid dehydrogenase), (Progesterone reductase); Steroid Delta-isomerase (EC 5.3.3.1) (Delta-, 5-3-ketosteroid isomerase)].]	Other Membrane
P26442	[Autocrine motility factor receptor precursor, isoform 1 (AMF, receptor).]	Other Membrane
P29350	[Tyrosine-protein phosphatase non-receptor type 6 (EC 3.1.3.48), (Protein-tyrosine phosphatase 1C) (PTP-1C) (Hematopoietic cell, protein-tyrosine phosphatase) (SH-PTP1) (Protein-tyrosine phosphatase, SHP-1).]	Other Membrane
P29728	[2'-5'-oligoadenylate synthetase 2 (EC 2.7.7.-) ((2-5')oligo(A), synthetase 2) (2-5A synthetase 2) (p69 OAS / p71 OAS) (p69OAS /, p71OAS).]	Other Membrane
P30533	[Alpha-2-macroglobulin receptor-associated protein precursor (Alpha-2-, MRAP) (Low density lipoprotein receptor-related protein-associated, protein 1) (RAP).]	Other Membrane
P35555	Fibrillin-1 precursor.	Other Membrane
P37173	[TGF-beta receptor type-2 precursor (EC 2.7.11.30) (TGF-beta receptor, type II) (TGFR-2) (TGF-beta type II receptor) (Transforming growth, factor-beta receptor type II) (TbetaR-II).]	Other Membrane
P41439	Folate receptor gamma precursor (FR-gamma) (Folate receptor 3).	Other Membrane
P98155	[Very low-density lipoprotein receptor precursor (VLDL receptor) (VLDL-, R).]	Other Membrane
Q02388	[Collagen alpha-1(VII) chain precursor (Long-chain collagen) (LC, collagen).]	Other Membrane
Q03001	[Bullous pemphigoid antigen 1, isoforms 1/2/3/4/5/8 (230 kDa bullous, pemphigoid antigen) (BPA) (Hemidesmosomal plaque protein) (Dystonia, musculorum protein) (Dystonin) (Fragment).]	Other Membrane
Q06481	[Amyloid-like protein 2 precursor (Amyloid protein homolog) (APPH), (CDEI box-binding protein) (CDEBP).]	Other Membrane
Q08828	[Adenylate cyclase type 1 (EC 4.6.1.1) (Adenylate cyclase type I) (ATP, pyrophosphate-lyase 1) (Adenylyl cyclase 1) (Ca(2+)/calmodulin-, activated adenylyl cyclase).]	Other Membrane
Q12846	Syntaxin-4 (Renal carcinoma antigen NY-REN-31).	Other Membrane
Q93070	[Ecto-ADP-ribosyltransferase 4 precursor (EC 2.4.2.31) (NAD(P)(+)--, arginine ADP-ribosyltransferase 4) (Mono(ADP-ribosyl)transferase 4), (Dombrock blood group carrier molecule) (CD297 antigen).]	Other Membrane
Q99643	[Succinate dehydrogenase cytochrome b560 subunit, mitochondrial, precursor (Integral membrane protein CII-3) (QPs1) (QPs-1) (Succinate, dehydrogenase complex subunit C) (Succinate-ubiquinone oxidoreductase, cytochrome B large subunit) (CYBL).]	Other Membrane
Q99717	[Mothers against decapentaplegic homolog 5 (SMAD 5) (Mothers against, DPP homolog 5) (Smad5) (hSmad5) (JV5-1).]	Other Membrane
Q9GZQ6	[Neuropeptide FF receptor 1 (G-protein coupled receptor 147) (RFamide-, related peptide receptor OT7T022).]	Other Membrane
Q9H251	Cadherin-23 precursor (Otocadherin).	Other Membrane
Q9H3N8	[Histamine H4 receptor (HH4R) (GPRv53) (G-protein coupled receptor 105), (SP9144) (AXOR35).]	Other Membrane
Q9NRJ7	Protocadherin beta 16 precursor (PCDH-beta16) (Protocadherin 3X).	Other Membrane
Q9NYJ7	Delta-like protein 3 precursor (Drosophila Delta homolog 3).	Other Membrane
Q9UEY8	Gamma-adducin (Adducin-like protein 70).	Other Membrane
Q9UKF2	[ADAM 30 precursor (EC 3.4.24.-) (A disintegrin and metalloproteinase, domain 30).]	Other Membrane
Q9ULB4	Cadherin-9 precursor.	Other Membrane
Q9Y345	[Sodium- and chloride-dependent glycine transporter 2 (GlyT2) (GlyT-2), (Solute carrier family 6 member 5).]	Other Membrane

P13726	[Tissue factor precursor (TF) (Coagulation factor III) (Thromboplastin), (CD142 antigen).]	Other Membrane
O14684	[Prostaglandin E synthase (EC 5.3.99.3) (Microsomal glutathione S-, transferase 1-like 1) (MGST1-L1) (p53-induced gene 12 protein).]	Other Membrane
O14983	[Sarcoplasmic/endoplasmic reticulum calcium ATPase 1 (EC 3.6.3.8), (Calcium pump 1) (SERCA1) (SR Ca(2+)-ATPase 1) (Calcium-transporting, ATPase sarcoplasmic reticulum type, fast twitch skeletal muscle, isoform) (Endoplasmic reticulum class 1/2 Ca(2+) ATPase).]	Other Membrane
O43747	[AP-1 complex subunit gamma-1 (Adapter-related protein complex 1, subunit gamma-1) (Gamma-adaptin) (Adaptor protein complex AP-1 gamma-1, subunit) (Golgi adaptor HA1/AP1 adaptin subunit gamma-1) (Clathrin, assembly protein complex 1 gamma-1 large chain).]	Other Membrane
O60242	Brain-specific angiogenesis inhibitor 3 precursor.	Other Membrane
O60609	GDNF family receptor alpha-3 precursor (GFR-alpha-3).	Other Membrane
O60683	[Peroxisome assembly protein 10 (Peroxin-10) (Peroxisome biogenesis, factor 10) (RING finger protein 69).]	Other Membrane
O75311	Glycine receptor subunit alpha-3 precursor.	Other Membrane
O75381	[Peroxisomal membrane protein PEX14 (Peroxin-14) (Peroxisomal membrane, anchor protein PEX14) (PTS1 receptor docking protein).]	Other Membrane
P07911	Uromodulin precursor (Tamm-Horsfall urinary glycoprotein) (THP).	Other Membrane
P09603	[Macrophage colony-stimulating factor 1 precursor (CSF-1) (MCSF) (M-, CSF) (Lanimostim) [Contains: Processed macrophage colony-stimulating, factor 1].]	Other Membrane
P12821	[Angiotensin-converting enzyme, somatic isoform precursor (EC 3.4.15.1), (Dipeptidyl carboxypeptidase I) (Kininase II) (CD143 antigen), [Contains: Angiotensin-converting enzyme, somatic isoform, soluble, form].]	Other Membrane
P12956	[ATP-dependent DNA helicase 2 subunit 1 (ATP-dependent DNA helicase II, 70 kDa subunit) (Lupus Ku autoantigen protein p70) (Ku70) (70 kDa, subunit of Ku antigen) (Thyroid-lupus autoantigen) (TLAA) (CTC box-, binding factor 75 kDa subunit) (CTCBF) (CTC75) (DNA-repair protein, XRCC6).]	Other Membrane
P13987	[CD59 glycoprotein precursor (Membrane attack complex inhibition, factor) (MACIF) (MAC-inhibitory protein) (MAC-IP) (Protectin) (MEM43, antigen) (Membrane inhibitor of reactive lysis) (MIRL) (20 kDa, homologous restriction factor) (HRF-20) (HRF20) (1F5 antigen).]	Other Membrane
P19634	[Sodium/hydrogen exchanger 1 (Na(+)/H(+) exchanger 1) (NHE-1) (Solute, carrier family 9 member 1) (Na(+)/H(+) antiporter, amiloride-, sensitive) (APNH).]	Other Membrane
P20023	[Complement receptor type 2 precursor (Cr2) (Complement C3d receptor), (Epstein-Barr virus receptor) (EBV receptor) (CD21 antigen).]	Other Membrane
P42345	[FKBP12-rapamycin complex-associated protein (FK506-binding protein 12-, rapamycin complex-associated protein 1) (Rapamycin target protein), (RAPT1) (Mammalian target of rapamycin) (mTOR).]	Other Membrane
P42892	Endothelin-converting enzyme 1 (EC 3.4.24.71) (ECE-1).	Other Membrane
P48066	Sodium- and chloride-dependent GABA transporter 3.	Other Membrane
P48546	[Gastric inhibitory polypeptide receptor precursor (GIP-R) (Glucose-, dependent insulinotropic polypeptide receptor).]	Other Membrane
P48553	[Transmembrane protein 1 (Epilepsy holoprosencephaly candidate 1, protein) (EHOC-1) (GT334 protein).]	Other Membrane
P51693	Amyloid-like protein 1 precursor (APLP) (APLP-1) [Contains: C30].	Other Membrane
P51797	Chloride channel protein 6 (ClC-6).	Other Membrane
P51809	[Synaptobrevin-like protein 1 (Tetanus insensitive VAMP) (Ti-VAMP) (Ti-, VAMP/VAMP7).]	Other Membrane

P56180	[Putative tyrosine-protein phosphatase TPTE (EC 3.1.3.48), (Transmembrane phosphatase with tensin homology) (Protein BJ-HCC-5), (Cancer/testis antigen 44) (CT44).]	Other Membrane
Q13621	[Solute carrier family 12 member 1 (Bumetanide-sensitive sodium-, (potassium)-chloride cotransporter 2) (Kidney-specific Na-K-Cl, symporter).]	Other Membrane
Q14112	Nidogen-2 precursor (NID-2) (Osteonidogen).	Other Membrane
Q92185	[Alpha-N-acetylneuraminide alpha-2,8-sialyltransferase (EC 2.4.99.8), (Ganglioside GD3 synthase) (Ganglioside GT3 synthase) (Alpha-2,8-, sialyltransferase 8A) (ST8Sia I).]	Other Membrane
O95248	Myotubularin-related protein 5 (SET-binding factor 1) (Sbf1).	Other Membrane
O95838	[Glucagon-like peptide 2 receptor precursor (GLP-2 receptor) (GLP-2-R), (GLP-2R).]	Other Membrane
O95859	[Tetraspanin-12 (Tspan-12) (Transmembrane 4 superfamily member 12), (Tetraspan NET-2).]	Other Membrane
P01859	Ig gamma-2 chain C region.	Other Membrane
P01871	Ig mu chain C region.	Other Membrane
P24043	[Laminin subunit alpha-2 precursor (Laminin M chain) (Merosin heavy, chain).]	Other Membrane
P25090	[FMLP-related receptor I (FMLP-R-I) (Lipoxin A4 receptor) (LXA4, receptor) (Formyl peptide receptor-like 1) (RFP) (HM63).]	Other Membrane
P33897	[ATP-binding cassette sub-family D member 1 (Adrenoleukodystrophy, protein) (ALDP).]	Other Membrane
P35498	[Sodium channel protein type 1 subunit alpha (Sodium channel protein, type I subunit alpha) (Voltage-gated sodium channel subunit alpha, Nav1.1) (Sodium channel protein, brain I subunit alpha).]	Other Membrane
P36894	[Bone morphogenetic protein receptor type IA precursor (EC 2.7.11.30), (Serine/threonine-protein kinase receptor R5) (SKR5) (Activin, receptor-like kinase 3) (ALK-3) (CD292 antigen).]	Other Membrane
P38435	[Vitamin K-dependent gamma-carboxylase (EC 6.4.-.-) (Gamma-glutamyl, carboxylase) (Vitamin K gamma glutamyl carboxylase).]	Other Membrane
P40145	[Adenylate cyclase type 8 (EC 4.6.1.1) (Adenylate cyclase type VIII), (ATP pyrophosphate-lyase 8) (Adenylyl cyclase 8) (Ca(2+)/calmodulin-, activated adenylyl cyclase).]	Other Membrane
Q02643	[Growth hormone-releasing hormone receptor precursor (GHRH receptor), (GRF receptor) (GRFR).]	Other Membrane
Q06430	[N-acetyllactosaminide beta-1,6-N-acetylglucosaminyl-transferase, (EC 2.4.1.150) (N-acetylglucosaminyltransferase) (I-branching enzyme), (IGNT).]	Other Membrane
Q08462	[Adenylate cyclase type 2 (EC 4.6.1.1) (Adenylate cyclase type II) (ATP, pyrophosphate-lyase 2) (Adenylyl cyclase 2).]	Other Membrane
Q10588	[ADP-ribosyl cyclase 2 precursor (EC 3.2.2.5) (Cyclic ADP-ribose, hydrolase 2) (cADPr hydrolase 2) (Bone marrow stromal antigen 1) (BST-, 1) (CD157 antigen).]	Other Membrane
Q12802	[A-kinase anchor protein 13 (AKAP 13) (Protein kinase A-anchoring, protein 13) (Breast cancer nuclear receptor-binding auxiliary protein), (Human thyroid-anchoring protein 31) (Guanine nucleotide exchange, factor Lbc) (AKAP-Lbc) (LBC oncogene) (P47) (Lymphoid blast crisis, oncogene) (Non-oncogenic Rho GTPase-specific GTP exchange factor).]	Other Membrane
Q12860	[Contactin-1 precursor (Neural cell surface protein F3) (Glycoprotein, gp135).]	Other Membrane
Q99747	[Gamma-soluble NSF attachment protein (SNAP-gamma) (N-ethylmaleimide-, sensitive factor attachment protein, gamma).]	Other Membrane
Q99835	Smoothened homolog precursor (SMO) (Gx protein).	Other Membrane
Q9P241	Probable phospholipid-transporting ATPase VD (EC 3.6.3.1) (ATPVD).	Other Membrane
Q9UKP6	Urotensin II receptor (UR-II-R) (G-protein coupled receptor 14).	Other Membrane
Q9ULB5	Cadherin-7 precursor.	Other Membrane
Q9Y2Q0	[Probable phospholipid-transporting ATPase IA (EC 3.6.3.1) (Chromaffin, granule ATPase II) (ATPase class I type 8A member 1).]	Other Membrane
P11171	Protein 4.1 (Band 4.1) (P4.1) (EPB4.1) (4.1R).	Plasma Membrane

O00155	Probable G-protein coupled receptor 25.	Plasma Membrane
O00238	[Bone morphogenetic protein receptor type IB precursor (EC 2.7.11.30), (CDw293 antigen).]	Plasma Membrane
O14894	[Transmembrane 4 L6 family member 5 (Tetraspan transmembrane protein, L6H).]	Plasma Membrane
O15393	[Transmembrane protease, serine 2 precursor (EC 3.4.21.-) (Serine, protease 10) [Contains: Transmembrane protease, serine 2 non-catalytic, chain; Transmembrane protease, serine 2 catalytic chain].]	Plasma Membrane
O15547	[P2X purinoceptor 6 (ATP receptor) (P2X6) (Purinergic receptor) (P2XM), (Purinergic receptor P2X-like 1).]	Plasma Membrane
O43193	Motilin receptor (G-protein coupled receptor 38).	Plasma Membrane
O43490	[Prominin-1 precursor (Prominin-like protein 1) (Antigen AC133) (CD133, antigen).]	Plasma Membrane
O43556	Epsilon-sarcoglycan precursor (Epsilon-SG).	Plasma Membrane
O43868	[Sodium/nucleoside cotransporter 2 (Na(+)/nucleoside cotransporter 2), (Sodium-coupled nucleoside transporter 2) (Concentrative nucleoside, transporter 2) (CNT 2) (hCNT2) (Sodium/purine nucleoside co-, transporter) (SPNT).]	Plasma Membrane
O75899	[Gamma-aminobutyric acid type B receptor subunit 2 precursor (GABA-B, receptor 2) (GABA-B-R2) (Gb2) (GABABR2) (G-protein coupled receptor, 51) (HG20).]	Plasma Membrane
P08172	Muscarinic acetylcholine receptor M2.	Plasma Membrane
P08588	[Beta-1 adrenergic receptor (Beta-1 adrenoceptor) (Beta-1, adrenoreceptor).]	Plasma Membrane
P08913	[Alpha-2A adrenergic receptor (Alpha-2A adrenoceptor) (Alpha-2A, adrenoreceptor) (Alpha-2AAR) (Alpha-2 adrenergic receptor subtype, C10).]	Plasma Membrane
P09693	[T-cell surface glycoprotein CD3 gamma chain precursor (T-cell receptor, T3 gamma chain).]	Plasma Membrane
P09848	[Lactase-phlorizin hydrolase precursor (Lactase-glycosylceramidase), [Includes: Lactase (EC 3.2.1.108); Phlorizin hydrolase (EC 3.2.1.62)].]	Plasma Membrane
P10747	T-cell-specific surface glycoprotein CD28 precursor (TP44).	Plasma Membrane
P11021	[78 kDa glucose-regulated protein precursor (GRP 78) (Heat shock 70 kDa, protein 5) (Immunoglobulin heavy chain-binding protein) (BiP), (Endoplasmic reticulum lumenal Ca(2+)-binding protein grp78).]	Plasma Membrane
P13224	[Platelet glycoprotein Ib beta chain precursor (GP-Ib beta) (GPIbB), (GPIb-beta) (Antigen CD42b-beta) (CD42c antigen).]	Plasma Membrane
P13866	[Sodium/glucose cotransporter 1 (Na(+)/glucose cotransporter 1) (High, affinity sodium-glucose cotransporter).]	Plasma Membrane
P13945	[Beta-3 adrenergic receptor (Beta-3 adrenoceptor) (Beta-3, adrenoreceptor).]	Plasma Membrane
P14384	Carboxypeptidase M precursor (EC 3.4.17.12) (CPM).	Plasma Membrane
P15328	[Folate receptor alpha precursor (FR-alpha) (Folate receptor 1) (Folate, receptor, adult) (Adult folate-binding protein) (FBP) (Ovarian tumor-, associated antigen MOv18) (KB cells FBP).]	Plasma Membrane
P15529	[Membrane cofactor protein precursor (Trophoblast leukocyte common, antigen) (TLX) (CD46 antigen).]	Plasma Membrane
P16070	[CD44 antigen precursor (Phagocytic glycoprotein I) (PGP-1) (HUTCH-I), (Extracellular matrix receptor-III) (ECMR-III) (GP90 lymphocyte, homing/adhesion receptor) (Hermes antigen) (Hyaluronate receptor), (Heparan sulfate proteoglycan) (Epican) (CDw44).]	Plasma Membrane
P16234	[Alpha-type platelet-derived growth factor receptor precursor, (EC 2.7.10.1) (PDGF-R-alpha) (CD140a antigen).]	Plasma Membrane
P16284	[Platelet endothelial cell adhesion molecule precursor (PECAM-1), (EndoCAM) (GPIIA') (CD31 antigen).]	Plasma Membrane
P16870	[Carboxypeptidase E precursor (EC 3.4.17.10) (CPE) (Carboxypeptidase H), (CPH) (Enkephalin convertase) (Prohormone-processing, carboxypeptidase).]	Plasma Membrane
P18433	[Receptor-type tyrosine-protein phosphatase alpha precursor, (EC 3.1.3.48) (Protein-tyrosine phosphatase alpha) (R-PTP-alpha).]	Plasma Membrane
P18827	Syndecan-1 precursor (SYND1) (CD138 antigen).	Plasma Membrane

P20039	[HLA class II histocompatibility antigen, DRB1-11 beta chain precursor, (MHC class I antigen DRB1*11) (DR-5) (DR5) (DRw11).]	Plasma Membrane
P20594	[Atrial natriuretic peptide receptor B precursor (ANP-B) (ANPRB) (GC-B), (Guanylate cyclase B) (EC 4.6.1.2) (NPR-B) (Atrial natriuretic peptide, B-type receptor).]	Plasma Membrane
P20701	[Integrin alpha-L precursor (Leukocyte adhesion glycoprotein LFA-1, alpha chain) (LFA-1A) (Leukocyte function-associated molecule 1 alpha, chain) (CD11a antigen).]	Plasma Membrane
P21439	[Multidrug resistance protein 3 (EC 3.6.3.44) (ATP-binding cassette, sub-family B member 4) (P-glycoprotein 3).]	Plasma Membrane
P42262	[Glutamate receptor 2 precursor (GluR-2) (GluR-B) (GluR-K2) (Glutamate, receptor ionotropic, AMPA 2) (AMPA-selective glutamate receptor 2).]	Plasma Membrane
P42702	[Leukemia inhibitory factor receptor precursor (LIF receptor) (LIF-R), (CD118 antigen).]	Plasma Membrane
P43121	[Cell surface glycoprotein MUC18 precursor (Melanoma-associated antigen, MUC18) (Melanoma cell adhesion molecule) (Melanoma-associated antigen, A32) (S-endo 1 endothelial-associated antigen) (Cell surface, glycoprotein P1H12) (CD146 antigen).]	Plasma Membrane
P43630	[Killer cell immunoglobulin-like receptor 3DL2 precursor (MHC class I, NK cell receptor) (Natural killer-associated transcript 4) (NKAT-4), (p70 natural killer cell receptor clone CL-5) (CD158k antigen).]	Plasma Membrane
P47872	Secretin receptor precursor (SCT-R).	Plasma Membrane
P48169	[Gamma-aminobutyric acid receptor subunit alpha-4 precursor (GABA(A), receptor subunit alpha-4).]	Plasma Membrane
P48995	Short transient receptor potential channel 1 (TrpC1) (TRP-1 protein).	Plasma Membrane
P49683	[Prolactin-releasing peptide receptor (PrRP receptor) (PrRPR) (G-, protein coupled receptor 10) (hGR3).]	Plasma Membrane
P51572	[B-cell receptor-associated protein 31 (BCR-associated protein Bap31), (p28 Bap31) (Protein CDM) (6C6-AG tumor-associated antigen).]	Plasma Membrane
P51790	Chloride channel protein 3 (ClC-3).	Plasma Membrane
P51800	Chloride channel protein ClC-Ka (Chloride channel Ka) (ClC-K1).	Plasma Membrane
P51841	[Retinal guanylyl cyclase 2 precursor (EC 4.6.1.2) (Guanylate cyclase, 2F, retinal) (RETGC-2) (Rod outer segment membrane guanylate cyclase, 2) (ROS-GC2) (Guanylate cyclase F) (GC-F).]	Plasma Membrane
P54764	[Ephrin type-A receptor 4 precursor (EC 2.7.10.1) (Tyrosine-protein, kinase receptor SEK) (Receptor protein-tyrosine kinase HEK8), (Tyrosine-protein kinase TYRO1).]	Plasma Membrane
P54826	Growth arrest-specific protein 1 precursor (GAS-1).	Plasma Membrane
P55011	[Solute carrier family 12 member 2 (Bumetanide-sensitive sodium-, (potassium)-chloride cotransporter 1) (Basolateral Na-K-Cl symporter).]	Plasma Membrane
P55087	[Aquaporin-4 (AQP-4) (WCH4) (Mercurial-insensitive water channel), (MIWC).]	Plasma Membrane
Q13488	[Vacuolar proton translocating ATPase 116 kDa subunit a isoform 3 (V-, ATPase 116 kDa isoform a3) (Osteoclastic proton pump 116 kDa subunit), (OC-116 kDa) (OC116) (T-cell immune regulator 1) (T-cell immune, response cDNA7 protein) (TIRC7).]	Plasma Membrane
Q13508	[Ecto-ADP-ribosyltransferase 3 precursor (EC 2.4.2.31) (NAD(P)(+)--, arginine ADP-ribosyltransferase 3) (Mono(ADP-ribosyl)transferase 3).]	Plasma Membrane
Q13635	Protein patched homolog 1 (PTC1) (PTC).	Plasma Membrane
Q13797	Integrin alpha-9 precursor (Integrin alpha-RLC).	Plasma Membrane
Q13972	[Guanine nucleotide-releasing protein (GNRP) (Ras-specific nucleotide, exchange factor CDC25) (Ras-specific guanine nucleotide-releasing, factor).]	Plasma Membrane
Q14246	[EGF-like module-containing mucin-like hormone receptor-like 1, precursor (Cell surface glycoprotein EMR1) (EMR1 hormone receptor).]	Plasma Membrane

Q14254	Flotillin-2 (Epidermal surface antigen) (ESA).	Plasma Membrane
Q14392	[Leucine-rich repeat-containing protein 32 precursor (GARP protein), (Garpin) (Glycoprotein A repetitions predominant).]	Plasma Membrane
Q14831	Metabotropic glutamate receptor 7 precursor (mGluR7).	Plasma Membrane
Q15262	[Receptor-type tyrosine-protein phosphatase kappa precursor, (EC 3.1.3.48) (Protein-tyrosine phosphatase kappa) (R-PTP-kappa).]	Plasma Membrane
Q15465	[Sonic hedgehog protein precursor (SHH) (HHG-1) [Contains: Sonic, hedgehog protein N-product; Sonic hedgehog protein C-product].]	Plasma Membrane
Q16099	[Glutamate receptor, ionotropic kainate 4 precursor (Glutamate receptor, KA-1) (KA1) (Excitatory amino acid receptor 1) (EAA1).]	Plasma Membrane
Q16720	[Plasma membrane calcium-transporting ATPase 3 (EC 3.6.3.8) (PMCA3), (Plasma membrane calcium pump isoform 3) (Plasma membrane calcium, ATPase isoform 3).]	Plasma Membrane
Q92633	Lysophosphatidic acid receptor Edg-2 (LPA receptor 1) (LPA-1).	Plasma Membrane
Q92673	[Sortilin-related receptor precursor (Sorting protein-related receptor, containing LDLR class A repeats) (SorLA) (SorLA-1) (Low-density, lipoprotein receptor relative with 11 ligand-binding repeats) (LDLR, relative with 11 ligand-binding repeats) (LR11).]	Plasma Membrane
Q92729	[Receptor-type tyrosine-protein phosphatase U precursor (EC 3.1.3.48), (R-PTP-U) (Protein-tyrosine phosphatase J) (PTP-J) (Pancreatic, carcinoma phosphatase 2) (PCP-2).]	Plasma Membrane
Q92736	[Ryanodine receptor 2 (Cardiac muscle-type ryanodine receptor) (RyR2), (RYR-2) (Cardiac muscle ryanodine receptor-calcium release channel), (hRYR-2).]	Plasma Membrane
Q92859	Neogenin precursor.	Plasma Membrane
Q92887	[Canalicular multispecific organic anion transporter 1 (ATP-binding, cassette sub-family C member 2) (Multidrug resistance-associated, protein 2) (Canalicular multidrug resistance protein).]	Plasma Membrane
Q92959	[Solute carrier organic anion transporter family member 2A1 (Solute, carrier family 21 member 2) (Prostaglandin transporter) (PGT).]	Plasma Membrane
O76090	Bestrophin-1 (Vitelliform macular dystrophy protein 2) (TU15B).	Plasma Membrane
O94759	[Transient receptor potential cation channel subfamily M member 2, (EC 3.6.1.13) (Long transient receptor potential channel 2) (LTrpC2), (LTrpC-2) (Transient receptor potential channel 7) (TrpC7) (Estrogen-, responsive element-associated gene 1 protein).]	Plasma Membrane
O95279	[Potassium channel subfamily K member 5 (Acid-sensitive potassium, channel protein TASK-2) (TWIK-related acid-sensitive K(+) channel 2).]	Plasma Membrane
O95971	CD160 antigen precursor (Natural killer cell receptor BY55).	Plasma Membrane
P26006	[Integrin alpha-3 precursor (Galactoprotein B3) (GAPB3) (VLA-3 alpha, chain) (FRP-2) (CD49c antigen) [Contains: Integrin alpha-3 heavy, chain; Integrin alpha-3 light chain].]	Plasma Membrane
P01589	[Interleukin-2 receptor alpha chain precursor (IL-2 receptor alpha, subunit) (IL-2-RA) (IL2-RA) (p55) (TAC antigen) (CD25 antigen).]	Plasma Membrane
P05187	[Alkaline phosphatase, placental type precursor (EC 3.1.3.1) (PLAP-1), (Alkaline phosphatase Regan isozyme).]	Plasma Membrane
P05362	[Intercellular adhesion molecule 1 precursor (ICAM-1) (Major group, rhinovirus receptor) (CD54 antigen).]	Plasma Membrane
P05981	[Serine protease hepsin (EC 3.4.21.106) (Transmembrane protease, serine, 1) [Contains: Serine protease hepsin non-catalytic chain; Serine, protease hepsin catalytic chain].]	Plasma Membrane
P06213	[Insulin receptor precursor (EC 2.7.10.1) (IR) (CD220 antigen), [Contains: Insulin receptor subunit alpha; Insulin receptor subunit, beta].]	Plasma Membrane
P06756	[Integrin alpha-V precursor (Vitronectin receptor subunit alpha) (CD51, antigen) [Contains: Integrin alpha-V heavy chain; Integrin alpha-V, light chain].]	Plasma Membrane
P07202	Thyroid peroxidase precursor (EC 1.11.1.8) (TPO).	Plasma Membrane

P21757	[Macrophage scavenger receptor types I and II (Macrophage acetylated, LDL receptor I and II) (Scavenger receptor class A member 1) (CD204, antigen).]	Plasma Membrane
P21854	B-cell differentiation antigen CD72 (Lyb-2).	Plasma Membrane
P23276	Kell blood group glycoprotein (EC 3.4.24.-) (CD238 antigen).	Plasma Membrane
P23416	Glycine receptor subunit alpha-2 precursor.	Plasma Membrane
P23470	[Receptor-type tyrosine-protein phosphatase gamma precursor, (EC 3.1.3.48) (Protein-tyrosine phosphatase gamma) (R-PTP-gamma).]	Plasma Membrane
P24046	[Gamma-aminobutyric acid receptor subunit rho-1 precursor (GABA(A), receptor subunit rho-1).]	Plasma Membrane
P24588	[A-kinase anchor protein 5 (A-kinase anchor protein 79 kDa) (AKAP 79), (cAMP-dependent protein kinase regulatory subunit II high affinity-, binding protein) (H21).]	Plasma Membrane
P28290	[Sperm-specific antigen 2 (Cleavage signal-1 protein) (CS-1) (Ki-ras-, induced actin-interacting protein).]	Plasma Membrane
P28336	[Neuromedin-B receptor (NMB-R) (Neuromedin-B-preferring bombesin, receptor).]	Plasma Membrane
P28827	[Receptor-type tyrosine-protein phosphatase mu precursor (EC 3.1.3.48), (Protein-tyrosine phosphatase mu) (R-PTP-mu).]	Plasma Membrane
P29320	[Ephrin type-A receptor 3 precursor (EC 2.7.10.1) (Tyrosine-protein, kinase receptor ETK1) (HEK) (HEK4) (Tyrosine-protein kinase TYRO4).]	Plasma Membrane
P30301	Lens fiber major intrinsic protein (Aquaporin-0) (MIP26) (MP26).	Plasma Membrane
P31785	[Cytokine receptor common gamma chain precursor (Gamma-C) (Interleukin-, 2 receptor gamma chain) (IL-2R gamma chain) (p64) (CD132 antigen).]	Plasma Membrane
P35499	[Sodium channel protein type 4 subunit alpha (Sodium channel protein, type IV subunit alpha) (Voltage-gated sodium channel subunit alpha, Nav1.4) (Sodium channel protein skeletal muscle subunit alpha) (SkM1).]	Plasma Membrane
P35523	[Chloride channel protein, skeletal muscle (Chloride channel protein 1), (ClC-1).]	Plasma Membrane
P35916	[Vascular endothelial growth factor receptor 3 precursor (EC 2.7.10.1), (VEGFR-3) (Tyrosine-protein kinase receptor FLT4).]	Plasma Membrane
P36021	[Monocarboxylate transporter 8 (MCT 8) (MCT 7) (Solute carrier family, 16 member 2) (X-linked PEST-containing transporter).]	Plasma Membrane
P36896	[Activin receptor type-1B precursor (EC 2.7.11.30) (ACTR-IB), (Serine/threonine-protein kinase receptor R2) (SKR2) (Activin, receptor-like kinase 4) (ALK-4).]	Plasma Membrane
P37023	[Serine/threonine-protein kinase receptor R3 precursor (EC 2.7.11.30), (SKR3) (Activin receptor-like kinase 1) (ALK-1) (TGF-B superfamily, receptor type I) (TSR-I).]	Plasma Membrane
P38646	[Stress-70 protein, mitochondrial precursor (75 kDa glucose-regulated, protein) (GRP 75) (Heat shock 70 kDa protein 9) (Peptide-binding, protein 74) (PBP74) (Mortalin) (MOT).]	Plasma Membrane
P40197	Platelet glycoprotein V precursor (GPV) (CD42D antigen).	Plasma Membrane
P40259	[B-cell antigen receptor complex-associated protein beta-chain, precursor (B-cell-specific glycoprotein B29) (Immunoglobulin-, associated B29 protein) (IG-beta) (CD79b antigen).]	Plasma Membrane
P41594	Metabotropic glutamate receptor 5 precursor (mGluR5).	Plasma Membrane
P63027	Vesicle-associated membrane protein 2 (VAMP-2) (Synaptobrevin-2).	Plasma Membrane
P78325	[ADAM 8 precursor (EC 3.4.24.-) (A disintegrin and metalloproteinase, domain 8) (Cell surface antigen MS2) (CD156a antigen) (CD156).]	Plasma Membrane
P78334	[Gamma-aminobutyric acid receptor subunit epsilon precursor (GABA(A), receptor subunit epsilon).]	Plasma Membrane
P78536	[ADAM 17 precursor (EC 3.4.24.86) (A disintegrin and metalloproteinase, domain 17) (TNF-alpha-converting enzyme) (TNF-alpha convertase) (Snake, venom-like protease) (CD156b antigen).]	Plasma Membrane

P78562	[Phosphate-regulating neutral endopeptidase (EC 3.4.24.-), (Metalloendopeptidase homolog PEX) (X-linked hypophosphatemia protein), (HYP) (Vitamin D-resistant hypophosphatemic rickets protein).]	Plasma Membrane
P98161	[Polycystin-1 precursor (Autosomal dominant polycystic kidney disease, protein 1).]	Plasma Membrane
P98172	[Ephrin-B1 precursor (EPH-related receptor tyrosine kinase ligand 2), (LERK-2) (ELK ligand) (ELK-L).]	Plasma Membrane
Q03405	[Urokinase plasminogen activator surface receptor precursor (uPAR) (U-, PAR) (Monocyte activation antigen Mo3) (CD87 antigen).]	Plasma Membrane
Q04609	[Glutamate carboxypeptidase 2 (EC 3.4.17.21) (Glutamate, carboxypeptidase II) (Membrane glutamate carboxypeptidase) (mGCP) (N-, acetylated-alpha-linked acidic dipeptidase I) (NAALADase I), (Pteroylpoly-gamma-glutamate carboxypeptidase) (Folylpoly-gamma-, glutamate carboxypeptidase) (FGCP) (Folate hydrolase 1) (Prostate-, specific membrane antigen) (PSMA) (PSM).]	Plasma Membrane
Q05940	[Synaptic vesicular amine transporter (Monoamine transporter), (Vesicular amine transporter 2) (VAT2) (Solute carrier family 18, member 2).]	Plasma Membrane
Q07075	[Glutamyl aminopeptidase (EC 3.4.11.7) (EAP) (Aminopeptidase A) (APA), (Differentiation antigen gp160) (CD249 antigen).]	Plasma Membrane
Q12879	[Glutamate [NMDA] receptor subunit epsilon-1 precursor (N-methyl D-, aspartate receptor subtype 2A) (NR2A) (NMDAR2A) (hNR2A).]	Plasma Membrane
Q12913	[Receptor-type tyrosine-protein phosphatase eta precursor (EC 3.1.3.48), (Protein-tyrosine phosphatase eta) (R-PTP-eta) (HPTP eta) (Protein-, tyrosine phosphatase receptor type J) (Density-enhanced phosphatase 1), (DEP-1) (CD148 antigen).]	Plasma Membrane
Q13003	[Glutamate receptor, ionotropic kainate 3 precursor (Glutamate receptor, 7) (GluR-7) (GluR7) (Excitatory amino acid receptor 5) (EAA5).]	Plasma Membrane
Q13224	[Glutamate [NMDA] receptor subunit epsilon-2 precursor (N-methyl D-, aspartate receptor subtype 2B) (NR2B) (NMDAR2B) (N-methyl-D-aspartate, receptor subunit 3) (NR3) (hNR3).]	Plasma Membrane
Q13368	MAGUK p55 subfamily member 3 (Protein MPP3) (Discs large homolog 3).	Plasma Membrane
Q93086	P2X purinoceptor 5 (ATP receptor) (P2X5) (Purinergic receptor).	Plasma Membrane
Q99075	[Proheparin-binding EGF-like growth factor precursor [Contains:, Heparin-binding EGF-like growth factor (HB-EGF) (HBEGF) (Diphtheria, toxin receptor) (DT-R)].]	Plasma Membrane
Q99965	[ADAM 2 precursor (A disintegrin and metalloproteinase domain 2), (Fertilin subunit beta) (PH-30) (PH30) (Cancer/testis antigen 15), (CT15).]	Plasma Membrane
Q9UBN1	[Voltage-dependent calcium channel gamma-4 subunit (Neuronal voltage-, gated calcium channel gamma-4 subunit).]	Plasma Membrane
Q9UJS0	[Calcium-binding mitochondrial carrier protein Aralar2 (Mitochondrial, aspartate glutamate carrier 2) (Solute carrier family 25 member 13), (Citrin).]	Plasma Membrane
Q9UKX5	Integrin alpha-11 precursor.	Plasma Membrane
Q9ULB1	Neurexin-1-alpha precursor (Neurexin I-alpha).	Plasma Membrane
Q9ULZ9	[Matrix metalloproteinase-17 precursor (EC 3.4.24.-) (MMP-17), (Membrane-type matrix metalloproteinase 4) (MT-MMP 4) (Membrane-type-4, matrix metalloproteinase) (MT4-MMP).]	Plasma Membrane
Q9Y2T5	Probable G-protein coupled receptor 52.	Plasma Membrane
P05067	[Amyloid beta A4 protein precursor (APP) (ABPP) (Alzheimer disease, amyloid protein) (Cerebral vascular amyloid peptide) (CVAP) (Protease, nexin-II) (PN-II) (APPI) (PreA4) [Contains: Soluble APP-alpha (S-APP-, alpha); Soluble APP-beta (S-APP-beta); C99; Beta-amyloid protein 42, (Beta-APP42); Beta-amyloid protein 40 (Beta-APP40); C83; P3(42);, P3(40); Gamma-CTF(59) (Gamma-secretase C-terminal fragment 59), (Amyloid intracellular domain 59) (AID(59)) (AICD-59); Gamma-CTF(57), (Gamma-secretase C-terminal fragment 57) (Amyloid intracellular domain, 57) (AID(57)) (AICD-57); Gamma-CTF(50) (Gamma-secretase C-terminal, fragment 50) (Amyloid intracellular domain 50) (AID(50)) (AICD-50);, C31].]	Plasma Membrane
O00159	Myosin-Ic (Myosin I beta) (MMI-beta) (MMIb).	Plasma Membrane

O00219	[Hyaluronan synthase 3 (EC 2.4.1.212) (Hyaluronate synthase 3), (Hyaluronic acid synthase 3) (HA synthase 3).]	Plasma Membrane
O00337	[Sodium/nucleoside cotransporter 1 (Na(+)/nucleoside cotransporter 1), (Sodium-coupled nucleoside transporter 1) (Concentrative nucleoside, transporter 1) (CNT 1) (hCNT1).]	Plasma Membrane
O00526	Uroplakin-2 precursor (UP 2) (Uroplakin II) (UPII).	Plasma Membrane
O14520	Aquaporin-7 (AQP-7) (Aquaporin-7-like) (Aquaporin adipose) (AQPap).	Plasma Membrane
O14807	Ras-related protein M-Ras precursor (Ras-related protein R-Ras3).	Plasma Membrane
O14843	Free fatty acid receptor 3 (G-protein coupled receptor 41).	Plasma Membrane
O15075	[Serine/threonine-protein kinase DCLK1 (EC 2.7.11.1) (Doublecortin-like, and CAM kinase-like 1) (Doublecortin-like kinase 1).]	Plasma Membrane
O15162	[Phospholipid scramblase 1 (PL scramblase 1) (Ca(2+)-dependent, phospholipid scramblase 1) (Erythrocyte phospholipid scramblase), (MmTRA1b).]	Plasma Membrane
O15374	[Monocarboxylate transporter 5 (MCT 5) (MCT 4) (Solute carrier family, 16 member 4).]	Plasma Membrane
O15440	[Multidrug resistance-associated protein 5 (ATP-binding cassette sub-, family C member 5) (Multi-specific organic anion transporter-C) (MOAT-, C) (pABC11) (SMRP).]	Plasma Membrane
O43520	[Probable phospholipid-transporting ATPase IC (EC 3.6.3.1) (Familial, intrahepatic cholestasis type 1) (ATPase class I type 8B member 1).]	Plasma Membrane
O43613	Orexin receptor type 1 (Ox1r) (Hypocretin receptor type 1).	Plasma Membrane
O60469	Down syndrome cell adhesion molecule precursor (CHD2).	Plasma Membrane
O60503	[Adenylate cyclase type 9 (EC 4.6.1.1) (Adenylate cyclase type IX) (ATP, pyrophosphate-lyase 9) (Adenylyl cyclase 9).]	Plasma Membrane
O75578	Integrin alpha-10 precursor.	Plasma Membrane
O75787	[Renin receptor precursor (Renin/prorenin receptor) (ATPase H(+)-, transporting lysosomal accessory protein 2) (ATPase H(+)-transporting, lysosomal-interacting protein 2) (Vacuolar ATP synthase membrane, sector-associated protein M8-9) (V-ATPase M8.9 subunit) (ATP6M8-9), (N14F) (ER-localized type I transmembrane adaptor) (Embryonic liver, differentiation factor 10).]	Plasma Membrane
P08069	[Insulin-like growth factor 1 receptor precursor (EC 2.7.10.1), (Insulin-like growth factor I receptor) (IGF-I receptor) (CD221, antigen) [Contains: Insulin-like growth factor 1 receptor alpha chain;, Insulin-like growth factor 1 receptor beta chain].]	Plasma Membrane
P08173	Muscarinic acetylcholine receptor M4.	Plasma Membrane
P08195	[4F2 cell-surface antigen heavy chain (4F2hc) (Lymphocyte activation, antigen 4F2 large subunit) (4F2 heavy chain antigen) (CD98 antigen).]	Plasma Membrane
P08571	[Monocyte differentiation antigen CD14 precursor (Myeloid cell-specific, leucine-rich glycoprotein) [Contains: Monocyte differentiation antigen, CD14, urinary form; Monocyte differentiation antigen CD14, membrane-, bound form].]	Plasma Membrane
P08581	[Hepatocyte growth factor receptor precursor (EC 2.7.10.1) (HGF, receptor) (Scatter factor receptor) (SF receptor) (HGF/SF receptor), (Met proto-oncogene tyrosine kinase) (c-Met).]	Plasma Membrane
P11142	Heat shock cognate 71 kDa protein (Heat shock 70 kDa protein 8).	Plasma Membrane
P11362	[Basic fibroblast growth factor receptor 1 precursor (EC 2.7.10.1), (FGFR-1) (bFGF-R) (Fms-like tyrosine kinase 2) (c-fgr) (CD331, antigen).]	Plasma Membrane
P14416	D(2) dopamine receptor (Dopamine D2 receptor).	Plasma Membrane
P14616	[Insulin receptor-related protein precursor (EC 2.7.10.1) (IRR) (IR-, related receptor) [Contains: Insulin receptor-related protein alpha, chain; Insulin receptor-related protein beta chain].]	Plasma Membrane
P14867	[Gamma-aminobutyric acid receptor subunit alpha-1 precursor (GABA(A), receptor subunit alpha-1).]	Plasma Membrane
P15812	T-cell surface glycoprotein CD1e precursor (CD1e antigen) (R2G1).	Plasma Membrane
P16157	Ankyrin-1 (Erythrocyte ankyrin) (Ankyrin-R).	Plasma Membrane

P16471	Prolactin receptor precursor (PRL-R).	Plasma Membrane
P17081	[Rho-related GTP-binding protein RhoQ precursor (Ras-related GTP-, binding protein TC10).]	Plasma Membrane
P17181	Interferon-alpha/beta receptor alpha chain precursor (IFN-alpha-REC).	Plasma Membrane
P17677	[Neuromodulin (Axonal membrane protein GAP-43) (Growth-associated, protein 43) (PP46) (Neural phosphoprotein B-50).]	Plasma Membrane
P18089	[Alpha-2B adrenergic receptor (Alpha-2B adrenoceptor) (Alpha-2B, adrenoreceptor) (Alpha-2 adrenergic receptor subtype C2).]	Plasma Membrane
P18564	Integrin beta-6 precursor.	Plasma Membrane
P20020	[Plasma membrane calcium-transporting ATPase 1 (EC 3.6.3.8) (PMCA1), (Plasma membrane calcium pump isoform 1) (Plasma membrane calcium, ATPase isoform 1).]	Plasma Membrane
P20648	[Potassium-transporting ATPase alpha chain 1 (EC 3.6.3.10) (Proton, pump) (Gastric H(+)/K(+) ATPase subunit alpha).]	Plasma Membrane
P47900	P2Y purinoceptor 1 (ATP receptor) (P2Y1) (Purinergic receptor).	Plasma Membrane
P49019	[Nicotinic acid receptor 2 (G-protein coupled receptor 109B) (G-protein, coupled receptor HM74) (G-protein coupled receptor HM74B).]	Plasma Membrane
P49286	Melatonin receptor type 1B (Mel-1B-R) (Mel1b melatonin receptor).	Plasma Membrane
P49757	Protein numb homolog (h-Numb) (Protein S171).	Plasma Membrane
P49961	[Ectonucleoside triphosphate diphosphohydrolase 1 (EC 3.6.1.5) (NTPDase, 1) (Ecto-ATP diphosphohydrolase) (ATPDase) (Lymphoid cell activation, antigen) (Ecto-apyrase) (CD39 antigen).]	Plasma Membrane
P50281	[Matrix metalloproteinase-14 precursor (EC 3.4.24.80) (MMP-14), (Membrane-type matrix metalloproteinase 1) (MT-MMP 1) (MTMMP1), (Membrane-type-1 matrix metalloproteinase) (MT1-MMP) (MT1MMP) (MMP-, X1).]	Plasma Membrane
P51575	P2X purinoceptor 1 (ATP receptor) (P2X1) (Purinergic receptor).	Plasma Membrane
P51795	Chloride channel protein 5 (ClC-5).	Plasma Membrane
P52569	[Low affinity cationic amino acid transporter 2 (CAT-2) (CAT2) (Solute, carrier family 7 member 2).]	Plasma Membrane
P53708	[Integrin alpha-8 precursor [Contains: Integrin alpha-8 heavy chain;, Integrin alpha-8 light chain].]	Plasma Membrane
P54652	Heat shock-related 70 kDa protein 2 (Heat shock 70 kDa protein 2).	Plasma Membrane
P54760	[Ephrin type-B receptor 4 precursor (EC 2.7.10.1) (Tyrosine-protein, kinase receptor HTK) (Tyrosine-protein kinase TYRO11).]	Plasma Membrane
P55064	Aquaporin-5 (AQP-5).	Plasma Membrane
P56199	[Integrin alpha-1 precursor (Laminin and collagen receptor) (VLA-1), (CD49a antigen).]	Plasma Membrane
P56589	[Peroxisomal biogenesis factor 3 (Peroxin-3) (Peroxisomal assembly, protein PEX3).]	Plasma Membrane
Q13873	[Bone morphogenetic protein receptor type-2 precursor (EC 2.7.11.30), (Bone morphogenetic protein receptor type II) (BMP type II receptor), (BMPR-II).]	Plasma Membrane
Q14108	[Lysosome membrane protein 2 (Lysosome membrane protein II) (LIMP II), (Scavenger receptor class B member 2) (85 kDa lysosomal membrane, sialoglycoprotein) (LGP85) (CD36 antigen-like 2).]	Plasma Membrane
Q14126	Desmoglein-2 precursor (HDGC).	Plasma Membrane
Q14444	[Caprin-1 (Cytoplasmic activation- and proliferation-associated protein, 1) (GPI-anchored membrane protein 1) (GPI-anchored protein p137), (p137GPI) (Membrane component chromosome 11 surface marker 1).]	Plasma Membrane
Q14644	[Ras GTPase-activating protein 3 (GAP1(IP4BP)) (Ins P4-binding, protein).]	Plasma Membrane
Q14957	[Glutamate [NMDA] receptor subunit epsilon-3 precursor (N-methyl D-, aspartate receptor subtype 2C) (NR2C) (NMDAR2C).]	Plasma Membrane
Q14982	[Opioid-binding protein/cell adhesion molecule precursor (OBCAM), (Opioid-binding cell adhesion molecule) (OPCML).]	Plasma Membrane
Q15149	Plectin-1 (PLTN) (PCN) (Hemidesmosomal protein 1) (HD1) (Plectin-11).	Plasma Membrane
Q15700	[Disks large homolog 2 (Postsynaptic density protein PSD-93) (Channel-, associated protein of synapse-110) (Chapsyn-110).]	Plasma Membrane

Q15722	[Leukotriene B4 receptor 1 (LTB4-R 1) (P2Y purinoceptor 7) (P2Y7), (Chemoattractant receptor-like 1) (G-protein coupled receptor 16).]	Plasma Membrane
Q16832	[Discoidin domain-containing receptor 2 precursor (EC 2.7.10.1), (Discoidin domain receptor 2) (Receptor protein-tyrosine kinase TKT), (Tyrosine-protein kinase TYRO10) (Neurotrophic tyrosine kinase,, receptor-related 3) (CD167b antigen).]	Plasma Membrane
Q92839	[Hyaluronan synthase 1 (EC 2.4.1.212) (Hyaluronate synthase 1), (Hyaluronic acid synthase 1) (HA synthase 1) (HuHAS1).]	Plasma Membrane
Q92932	[Receptor-type tyrosine-protein phosphatase N2 precursor (EC 3.1.3.48), (R-PTP-N2) (Islet cell autoantigen-related protein) (ICAAR) (IAR), (Phogrin).]	Plasma Membrane
O94907	[Dickkopf-related protein 1 precursor (Dkk-1) (Dickkopf-1) (hDkk-1), (SK).]	Plasma Membrane
O95255	[Multidrug resistance-associated protein 6 (ATP-binding cassette sub-, family C member 6) (Anthracycline resistance-associated protein), (Multi-specific organic anion transporter-E) (MOAT-E).]	Plasma Membrane
O95858	[Tetraspanin-15 (Tspan-15) (Transmembrane 4 superfamily member 15), (Tetraspan NET-7).]	Plasma Membrane
P25929	Neuropeptide Y receptor type 1 (NPY1-R).	Plasma Membrane
P26010	Integrin beta-7 precursor.	Plasma Membrane
P01213	[Beta-neoendorphin-dynorphin precursor (Proenkephalin B), (Preprodynorphin) [Contains: Alpha-neoendorphin; Beta-neoendorphin;, Big dynorphin (Big Dyn); Dynorphin A(1-17) (Dynorphin A) (Dyn-A17);, Dynorphin A(1-13); Dynorphin A(1-8); Leu-enkephalin; Rimorphin, (Dynorphin B) (Dyn-B) (Dynorphin B(1-13)); Leumorphin (Dynorphin B-, 29)].]	Plasma Membrane
P01733	T-cell receptor beta chain V region YT35 precursor.	Plasma Membrane
P02748	[Complement component C9 precursor [Contains: Complement component C9a;, Complement component C9b].]	Plasma Membrane
P07333	[Macrophage colony-stimulating factor 1 receptor precursor, (EC 2.7.10.1) (CSF-1-R) (Fms proto-oncogene) (c-fms) (CD115 antigen).]	Plasma Membrane
P07359	[Platelet glycoprotein Ib alpha chain precursor (Glycoprotein Ibalpha), (GP-Ib alpha) (GPIbA) (GPIb-alpha) (Antigen CD42b-alpha) (CD42b, antigen) [Contains: Glycocalicin].]	Plasma Membrane
P21452	[Substance-K receptor (SKR) (Neurokinin A receptor) (NK-2 receptor), (NK-2R) (Tachykinin receptor 2).]	Plasma Membrane
P21860	[Receptor tyrosine-protein kinase erbB-3 precursor (EC 2.7.10.1) (c-, erbB3) (Tyrosine kinase-type cell surface receptor HER3).]	Plasma Membrane
P23467	[Receptor-type tyrosine-protein phosphatase beta precursor, (EC 3.1.3.48) (Protein-tyrosine phosphatase beta) (R-PTP-beta).]	Plasma Membrane
P23471	[Receptor-type tyrosine-protein phosphatase zeta precursor, (EC 3.1.3.48) (R-PTP-zeta).]	Plasma Membrane
P24071	[Immunoglobulin alpha Fc receptor precursor (IgA Fc receptor) (CD89, antigen).]	Plasma Membrane
P24530	[Endothelin B receptor precursor (ET-B) (Endothelin receptor Non-, selective type).]	Plasma Membrane
P28476	[Gamma-aminobutyric acid receptor subunit rho-2 precursor (GABA(A), receptor subunit rho-2).]	Plasma Membrane
P29279	[Connective tissue growth factor precursor (Hypertrophic chondrocyte-, specific protein 24).]	Plasma Membrane
P29973	[cGMP-gated cation channel alpha 1 (CNG channel alpha 1) (CNG-1) (CNG1), (Cyclic nucleotide-gated channel alpha 1) (Cyclic nucleotide-gated, channel, photoreceptor) (Cyclic nucleotide-gated cation channel 1), (Rod photoreceptor cGMP-gated channel subunit alpha).]	Plasma Membrane
P30460	[HLA class I histocompatibility antigen, B-8 alpha chain precursor (MHC, class I antigen B*8).]	Plasma Membrane
P32238	[Cholecystokinin receptor type A (CCK-A receptor) (CCK-AR), (Cholecystokinin-1 receptor) (CCK1-R).]	Plasma Membrane
P32248	[C-C chemokine receptor type 7 precursor (C-C CKR-7) (CC-CKR-7) (CCR-7), (MIP-3 beta receptor) (EBV-induced G-protein coupled receptor 1), (EBI1) (BLR2) (CD197 antigen) (CDw197).]	Plasma Membrane
P34903	[Gamma-aminobutyric acid receptor subunit alpha-3 precursor (GABA(A), receptor subunit alpha-3).]	Plasma Membrane
P35232	Prohibitin.	Plasma Membrane

P35367	Histamine H1 receptor.	Plasma Membrane
P35968	[Vascular endothelial growth factor receptor 2 precursor (EC 2.7.10.1), (VEGFR-2) (Kinase insert domain receptor) (Protein-tyrosine kinase, receptor Flk-1) (CD309 antigen).]	Plasma Membrane
P36888	[FL cytokine receptor precursor (EC 2.7.10.1) (Tyrosine-protein kinase, receptor FLT3) (Stem cell tyrosine kinase 1) (STK-1) (CD135 antigen).]	Plasma Membrane
P36897	[TGF-beta receptor type-1 precursor (EC 2.7.11.30) (TGF-beta receptor, type I) (TGFR-1) (TGF-beta type I receptor) (Transforming growth, factor-beta receptor type I) (TbetaR-I) (Serine/threonine-protein, kinase receptor R4) (SKR4) (Activin receptor-like kinase 5) (ALK-5).]	Plasma Membrane
P38567	[Hyaluronidase PH-20 precursor (EC 3.2.1.35) (Hyal-PH20) (Sperm surface, protein PH-20) (Sperm adhesion molecule 1).]	Plasma Membrane
P40199	[Carcinoembryonic antigen-related cell adhesion molecule 6 precursor, (Normal cross-reacting antigen) (Non-specific crossreacting antigen), (CD66c antigen).]	Plasma Membrane
P41217	OX-2 membrane glycoprotein precursor (CD200 antigen).	Plasma Membrane
P78310	[Coxsackievirus and adenovirus receptor precursor (Coxsackievirus B-, adenovirus receptor) (hCAR) (CVB3-binding protein) (HCVADR).]	Plasma Membrane
P78539	Sushi repeat-containing protein SRPX precursor.	Plasma Membrane
Q00341	[Vigilin (High density lipoprotein-binding protein) (HDL-binding, protein).]	Plasma Membrane
Q02094	[Rhesus blood group-associated glycoprotein (Rhesus blood group-, associated ammonia channel) (Erythrocyte plasma membrane 50 kDa, glycoprotein) (Rh50A) (CD241 antigen).]	Plasma Membrane
Q02161	[Blood group Rh(D) polypeptide (Rhesus D antigen) (RHXIII) (Rh, polypeptide 2) (RhPII) (CD240D antigen).]	Plasma Membrane
Q02246	[Contactin-2 precursor (Axonin-1) (Axonal glycoprotein TAG-1), (Transient axonal glycoprotein 1) (TAX-1).]	Plasma Membrane
Q06418	[Tyrosine-protein kinase receptor TYRO3 precursor (EC 2.7.10.1), (Tyrosine-protein kinase RSE) (Tyrosine-protein kinase SKY) (Tyrosine-, protein kinase DTK) (Protein-tyrosine kinase byk).]	Plasma Membrane
Q07954	[Prolow-density lipoprotein receptor-related protein 1 precursor (LRP), (Alpha-2-macroglobulin receptor) (A2MR) (Apolipoprotein E receptor), (APOER) (CD91 antigen) [Contains: Low-density lipoprotein receptor-, related protein 1 85 kDa subunit (LRP-85); Low-density lipoprotein, receptor-related protein 1 515 kDa subunit (LRP-515); Low-density, lipoprotein receptor-related protein 1 intracellular domain (LRPICD)].]	Plasma Membrane
Q08289	[Voltage-dependent L-type calcium channel subunit beta-2 (CAB2), (Calcium channel voltage-dependent subunit beta 2) (Lambert-Eaton, myasthenic syndrome antigen B) (MYSB).]	Plasma Membrane
Q08554	Desmocollin-1 precursor (Desmosomal glycoprotein 2/3) (DG2/DG3).	Plasma Membrane
Q99677	[Lysophosphatidic acid receptor 4 (LPA receptor 4) (LPA-4) (P2Y, purinoceptor 9) (P2Y9) (Purinergic receptor 9) (G-protein coupled, receptor 23) (P2Y5-like receptor).]	Plasma Membrane
Q99795	Cell surface A33 antigen precursor (Glycoprotein A33).	Plasma Membrane
Q9H158	Protocadherin alpha C1 precursor (PCDH-alpha-C1).	Plasma Membrane
Q9H1Y3	Opsin-3 (Encephalopsin) (Panopsin).	Plasma Membrane
Q9NPA2	[Matrix metalloproteinase-25 precursor (EC 3.4.24.-) (MMP-25), (Membrane-type matrix metalloproteinase 6) (MT-MMP 6) (Membrane-type-6, matrix metalloproteinase) (MT6-MMP) (Leukolysin).]	Plasma Membrane
Q9UBC5	[Myosin-Ia (Brush border myosin I) (BBM-I) (BBMI) (Myosin I heavy, chain) (MIHC).]	Plasma Membrane
Q9UII2	ATPase inhibitor, mitochondrial precursor.	Plasma Membrane
Q9UL52	[Transmembrane protease, serine 11E precursor (EC 3.4.21.-) (Serine, protease DESC1) [Contains: Transmembrane protease, serine 11E non-, catalytic chain; Transmembrane protease, serine 11E catalytic chain].]	Plasma Membrane

Q9UL62	Short transient receptor potential channel 5 (TrpC5) (Htrp-5) (Htrp5).	Plasma Membrane
Q9UN88	[Gamma-aminobutyric acid receptor subunit theta precursor (GABA(A), receptor subunit theta).]	Plasma Membrane
Q9UQ49	[Sialidase-3 (EC 3.2.1.18) (Membrane sialidase) (Ganglioside sialidase), (N-acetyl-alpha-neuraminidase 3).]	Plasma Membrane
Q9Y256	[CAAX prenyl protease 2 (EC 3.4.22.-) (Prenyl protein-specific, endoprotease 2) (Farnesylated proteins-converting enzyme 2) (FACE-2), (hRCE1).]	Plasma Membrane
O00222	Metabotropic glutamate receptor 8 precursor (mGluR8).	Plasma Membrane
O00624	[Sodium-dependent phosphate transport protein 3 (Sodium/phosphate, cotransporter 3) (Na(+)/PI cotransporter 3) (Solute carrier family 17, member 2).]	Plasma Membrane
O14764	[Gamma-aminobutyric acid receptor subunit delta precursor (GABA(A), receptor subunit delta).]	Plasma Membrane
O14939	[Phospholipase D2 (EC 3.1.4.4) (PLD 2) (Choline phosphatase 2), (Phosphatidylcholine-hydrolyzing phospholipase D2) (PLD1C) (hPLD2).]	Plasma Membrane
O15078	[Centrosomal protein Cep290 (Nephrocystin-6) (Tumor antigen se2-2), (Cancer/testis antigen 87) (CT87).]	Plasma Membrane
O15303	Metabotropic glutamate receptor 6 precursor (mGluR6).	Plasma Membrane
O15438	[Canalicular multispecific organic anion transporter 2 (ATP-binding, cassette sub-family C member 3) (Multidrug resistance-associated, protein 3) (Multi-specific organic anion transporter-D) (MOAT-D).]	Plasma Membrane
O15529	Putative G-protein coupled receptor 42.	Plasma Membrane
O43187	Interleukin-1 receptor-associated kinase-like 2 (IRAK-2).	Plasma Membrane
O43614	Orexin receptor type 2 (Ox2r) (Hypocretin receptor type 2).	Plasma Membrane
O43808	[Peroxisomal membrane protein PMP34 (34 kDa peroxisomal membrane, protein) (Solute carrier family 25 member 17).]	Plasma Membrane
O43847	[Nardilysin precursor (EC 3.4.24.61) (N-arginine dibasic convertase), (NRD convertase) (NRD-C).]	Plasma Membrane
O60266	[Adenylate cyclase type 3 (EC 4.6.1.1) (Adenylate cyclase type III), (ATP pyrophosphate-lyase 3) (Adenylyl cyclase 3) (AC-III) (AC3), (Adenylate cyclase, olfactive type).]	Plasma Membrane
O75323	Protein NipSnap2 (Glioblastoma amplified sequence).	Plasma Membrane
O75487	Glypican-4 precursor (K-glypican).	Plasma Membrane
P08183	[Multidrug resistance protein 1 (EC 3.6.3.44) (ATP-binding cassette, sub-family B member 1) (P-glycoprotein 1) (CD243 antigen).]	Plasma Membrane
P08473	[Neprilysin (EC 3.4.24.11) (Neutral endopeptidase) (NEP), (Enkephalinase) (Neutral endopeptidase 24.11) (Atriopeptidase) (Common, acute lymphocytic leukemia antigen) (CALLA) (CD10 antigen).]	Plasma Membrane
P08514	[Integrin alpha-IIb precursor (Platelet membrane glycoprotein IIb), (GPalpha IIb) (GPIIb) (CD41 antigen) [Contains: Integrin alpha-IIb, heavy chain; Integrin alpha-IIb light chain, form 1; Integrin alpha-, IIb light chain, form 2].]	Plasma Membrane
P08582	[Melanotransferrin precursor (Melanoma-associated antigen p97) (CD228, antigen).]	Plasma Membrane
P08637	[Low affinity immunoglobulin gamma Fc region receptor III-A precursor, (IgG Fc receptor III-2) (Fc-gamma RIII-alpha) (Fc-gamma RIIIa), (FcRIIIa) (Fc-gamma RIII) (FcRIII) (FcR-10) (CD16a antigen).]	Plasma Membrane
P09326	[CD48 antigen precursor (B-lymphocyte activation marker BLAST-1) (BCM1, surface antigen) (Leukocyte antigen MEM-102) (TCT.1).]	Plasma Membrane
P10586	[Receptor-type tyrosine-protein phosphatase F precursor (EC 3.1.3.48), (LAR protein) (Leukocyte antigen related).]	Plasma Membrane
P10912	[Growth hormone receptor precursor (GH receptor) (Somatotropin, receptor) [Contains: Growth hormone-binding protein (GH-binding, protein) (GHBP) (Serum-binding protein)].]	Plasma Membrane
P11215	[Integrin alpha-M precursor (Cell surface glycoprotein MAC-1 subunit, alpha) (CR-3 alpha chain) (Leukocyte adhesion receptor MO1), (Neutrophil adherence receptor) (CD11b antigen).]	Plasma Membrane
P11279	[Lysosome-associated membrane glycoprotein 1 precursor (LAMP-1) (CD107a, antigen).]	Plasma Membrane

P13569	[Cystic fibrosis transmembrane conductance regulator (CFTR) (cAMP-, dependent chloride channel) (ATP-binding cassette transporter sub-, family C member 7).]	Plasma Membrane
P13598	Intercellular adhesion molecule 2 precursor (ICAM-2) (CD102 antigen).	Plasma Membrane
P13746	[HLA class I histocompatibility antigen, A-11 alpha chain precursor, (MHC class I antigen A*11).]	Plasma Membrane
P14778	[Interleukin-1 receptor type I precursor (IL-1R-1) (IL-1RT1) (IL-1R-, alpha) (p80) (CD121a antigen).]	Plasma Membrane
P15391	[B-lymphocyte antigen CD19 precursor (Differentiation antigen CD19) (B-, lymphocyte surface antigen B4) (Leu-12).]	Plasma Membrane
P16066	[Atrial natriuretic peptide receptor A precursor (ANP-A) (ANPRA) (GC-A), (Guanylate cyclase) (EC 4.6.1.2) (NPR-A) (Atrial natriuretic peptide, A-type receptor).]	Plasma Membrane
P16144	Integrin beta-4 precursor (GP150) (CD104 antigen).	Plasma Membrane
P16671	[Platelet glycoprotein 4 (Platelet glycoprotein IV) (GPIV), (Glycoprotein IIIb) (GPIIIB) (Leukocyte differentiation antigen CD36), (CD36 antigen) (PAS IV) (PAS-4 protein) (Platelet collagen receptor), (Fatty acid translocase) (FAT) (Thrombospondin receptor).]	Plasma Membrane
P17301	[Integrin alpha-2 precursor (Platelet membrane glycoprotein Ia) (GPIa), (Collagen receptor) (VLA-2 alpha chain) (CD49b antigen).]	Plasma Membrane
P17813	Endoglin precursor (CD105 antigen).	Plasma Membrane
P17927	[Complement receptor type 1 precursor (C3b/C4b receptor) (CD35, antigen).]	Plasma Membrane
P20309	Muscarinic acetylcholine receptor M3.	Plasma Membrane
P41968	Melanocortin receptor 3 (MC3-R).	Plasma Membrane
P42261	[Glutamate receptor 1 precursor (GluR-1) (GluR-A) (GluR-K1) (Glutamate, receptor ionotropic, AMPA 1) (AMPA-selective glutamate receptor 1).]	Plasma Membrane
P42701	[Interleukin-12 receptor beta-1 chain precursor (IL-12R-beta1), (Interleukin-12 receptor beta) (IL-12 receptor beta component) (IL-, 12RB1) (CD212 antigen).]	Plasma Membrane
P43626	[Killer cell immunoglobulin-like receptor 2DL1 precursor (MHC class I, NK cell receptor) (Natural killer-associated transcript 1) (NKAT-1), (p58 natural killer cell receptor clones CL-42/47.11) (p58 NK, receptor) (p58.1 MHC class-I-specific NK receptor) (CD158a antigen).]	Plasma Membrane
P46098	[5-hydroxytryptamine receptor 3 precursor (5-HT-3) (Serotonin-gated ion, channel receptor) (5-HT3R).]	Plasma Membrane
P48058	[Glutamate receptor 4 precursor (GluR-4) (GluR4) (GluR-D) (Glutamate, receptor ionotropic, AMPA 4) (AMPA-selective glutamate receptor 4).]	Plasma Membrane
P48167	[Glycine receptor subunit beta precursor (Glycine receptor 58 kDa, subunit).]	Plasma Membrane
P48664	[Excitatory amino acid transporter 4 (Solute carrier family 1 member 6), (Sodium-dependent glutamate/aspartate transporter).]	Plasma Membrane
P48960	[CD97 antigen precursor (Leukocyte antigen CD97) [Contains: CD97, antigen subunit alpha; CD97 antigen subunit beta].]	Plasma Membrane
P50395	[Rab GDP dissociation inhibitor beta (Rab GDI beta) (Guanosine, diphosphate dissociation inhibitor 2) (GDI-2).]	Plasma Membrane
P51170	[Amiloride-sensitive sodium channel subunit gamma (Epithelial Na(+), channel subunit gamma) (Gamma-ENaC) (Nonvoltage-gated sodium channel 1, subunit gamma) (SCNEG) (Gamma-NaCH).]	Plasma Membrane
P51512	[Matrix metalloproteinase-16 precursor (EC 3.4.24.-) (MMP-16), (Membrane-type matrix metalloproteinase 3) (MT-MMP 3) (MTMMP3), (Membrane-type-3 matrix metalloproteinase) (MT3-MMP) (MT3MMP) (MMP-, X2).]	Plasma Membrane
P51582	P2Y purinoceptor 4 (P2Y4) (Uridine nucleotide receptor) (UNR) (P2P).	Plasma Membrane
P51677	[C-C chemokine receptor type 3 (C-C CKR-3) (CC-CKR-3) (CCR-3) (CCR3), (CKR3) (Eosinophil eotaxin receptor) (CD193 antigen).]	Plasma Membrane
P51828	[Adenylate cyclase type 7 (EC 4.6.1.1) (Adenylate cyclase type VII), (ATP pyrophosphate-lyase 7) (Adenylyl cyclase 7).]	Plasma Membrane
P54753	[Ephrin type-B receptor 3 precursor (EC 2.7.10.1) (Tyrosine-protein, kinase receptor HEK-2) (Tyrosine-protein kinase TYRO6).]	Plasma Membrane
P54762	[Ephrin type-B receptor 1 precursor (EC 2.7.10.1) (Tyrosine-protein, kinase receptor EPH-2) (NET) (HEK6) (ELK).]	Plasma Membrane

P55196	Afadin (Protein AF-6).	Plasma Membrane
P55291	Cadherin-15 precursor (Muscle cadherin) (M-cadherin) (Cadherin-14).	Plasma Membrane
Q13507	Short transient receptor potential channel 3 (TrpC3) (Htrp-3) (Htrp3).	Plasma Membrane
Q13705	[Activin receptor type-2B precursor (EC 2.7.11.30) (Activin receptor, type IIB) (ACTR-IIB).]	Plasma Membrane
Q13796	Protein Shroom2 (Apical-like protein) (Protein APXL).	Plasma Membrane
Q14517	[Cadherin-related tumor suppressor homolog precursor (Protein fat, homolog).]	Plasma Membrane
Q14574	Desmocollin-3 precursor (Desmocollin-4) (HT-CP).	Plasma Membrane
Q15109	[Advanced glycosylation end product-specific receptor precursor, (Receptor for advanced glycosylation end products).]	Plasma Membrane
Q15303	[Receptor tyrosine-protein kinase erbB-4 precursor (EC 2.7.10.1), (p180erbB4) (Tyrosine kinase-type cell surface receptor HER4).]	Plasma Membrane
Q15390	[Mitochondrial fission regulator 1 (Chondrocyte protein with a poly-, proline region).]	Plasma Membrane
Q15760	Probable G-protein coupled receptor 19 (GPR-NGA).	Plasma Membrane
Q16445	[Gamma-aminobutyric acid receptor subunit alpha-6 precursor (GABA(A), receptor subunit alpha-6).]	Plasma Membrane
Q16602	[Calcitonin gene-related peptide type 1 receptor precursor (CGRP type 1, receptor) (Calcitonin receptor-like receptor).]	Plasma Membrane
Q16625	Occludin.	Plasma Membrane
Q16653	Myelin-oligodendrocyte glycoprotein precursor.	Plasma Membrane
Q16849	[Receptor-type tyrosine-protein phosphatase-like N precursor (R-PTP-N), (PTP IA-2) (Islet cell antigen 512) (ICA 512) (Islet cell autoantigen, 3).]	Plasma Membrane
O95258	[Brain mitochondrial carrier protein 1 (BMCP-1) (Mitochondrial, uncoupling protein 5) (UCP 5) (Solute carrier family 25 member 14).]	Plasma Membrane
P01848	T-cell receptor alpha chain C region.	Plasma Membrane
P03979	T-cell receptor gamma chain V region PT-gamma-1/2 precursor.	Plasma Membrane
P04435	T-cell receptor beta chain V region CTL-L17 precursor.	Plasma Membrane
P05107	[Integrin beta-2 precursor (Cell surface adhesion glycoproteins LFA-, 1/CR3/p150,95 subunit beta) (Complement receptor C3 subunit beta), (CD18 antigen).]	Plasma Membrane
P06340	[HLA class II histocompatibility antigen, DO alpha chain precursor (MHC, class II antigen DOA) (MHC DZ alpha) (MHC DN-alpha).]	Plasma Membrane
P07510	Acetylcholine receptor subunit gamma precursor.	Plasma Membrane
P21709	[Ephrin type-A receptor 1 precursor (EC 2.7.10.1) (Tyrosine-protein, kinase receptor EPH).]	Plasma Membrane
P21802	[Fibroblast growth factor receptor 2 precursor (EC 2.7.10.1) (FGFR-2), (Keratinocyte growth factor receptor 2) (CD332 antigen).]	Plasma Membrane
P21817	[Ryanodine receptor 1 (Skeletal muscle-type ryanodine receptor) (RyR1), (RYR-1) (Skeletal muscle calcium release channel).]	Plasma Membrane
P22681	[E3 ubiquitin-protein ligase CBL (EC 6.3.2.-) (Signal transduction, protein CBL) (Proto-oncogene c-CBL) (Casitas B-lineage lymphoma proto-, oncogene) (RING finger protein 55).]	Plasma Membrane
P22897	Macrophage mannose receptor 1 precursor (MMR) (CD206 antigen).	Plasma Membrane
P23415	[Glycine receptor subunit alpha-1 precursor (Glycine receptor 48 kDa, subunit) (Glycine receptor strychnine-binding subunit).]	Plasma Membrane
P23634	[Plasma membrane calcium-transporting ATPase 4 (EC 3.6.3.8) (PMCA4), (Plasma membrane calcium pump isoform 4) (Plasma membrane calcium, ATPase isoform 4) (Matrix-remodeling-associated protein 1).]	Plasma Membrane
P28223	[5-hydroxytryptamine receptor 2A (5-HT-2A) (Serotonin receptor 2A) (5-, HT-2).]	Plasma Membrane
P28566	[5-hydroxytryptamine receptor 1E (5-HT-1E) (Serotonin receptor 1E) (5-, HT1E) (S31).]	Plasma Membrane
P29317	[Ephrin type-A receptor 2 precursor (EC 2.7.10.1) (Tyrosine-protein, kinase receptor ECK) (Epithelial cell kinase).]	Plasma Membrane

P29353	[SHC-transforming protein 1 (SH2 domain protein C1) (Src homology 2, domain-containing-transforming protein C1).]	Plasma Membrane
P29376	[Leukocyte tyrosine kinase receptor precursor (EC 2.7.10.1) (Protein, tyrosine kinase 1).]	Plasma Membrane
P30273	[High affinity immunoglobulin epsilon receptor subunit gamma precursor, (FceRI gamma) (IgE Fc receptor subunit gamma) (Fc-epsilon RI-gamma).]	Plasma Membrane
P30530	[Tyrosine-protein kinase receptor UFO precursor (EC 2.7.10.1) (AXL, oncogene).]	Plasma Membrane
P30954	[Olfactory receptor 10J1 (Olfactory receptor OR1-26) (Olfactory, receptor-like protein HGMP07J).]	Plasma Membrane
P32241	[Vasoactive intestinal polypeptide receptor 1 precursor (VIP-R-1), (Pituitary adenylate cyclase-activating polypeptide type II receptor), (PACAP type II receptor) (PACAP-R-2).]	Plasma Membrane
P32745	Somatostatin receptor type 3 (SS3R) (SSR-28).	Plasma Membrane
P33527	[Multidrug resistance-associated protein 1 (ATP-binding cassette sub-, family C member 1) (Leukotriene C(4) transporter) (LTC4 transporter).]	Plasma Membrane
P34910	[EVI2B protein precursor (Ecotropic viral integration site 2B protein, homolog) (EVI-2B).]	Plasma Membrane
P34981	[Thyrotropin-releasing hormone receptor (TRH-R) (Thyroliberin, receptor).]	Plasma Membrane
P35368	[Alpha-1B adrenergic receptor (Alpha 1B-adrenoceptor) (Alpha 1B-, adrenoreceptor).]	Plasma Membrane
P35670	[Copper-transporting ATPase 2 (EC 3.6.3.4) (Copper pump 2) (Wilson, disease-associated protein) [Contains: WND/140 kDa].]	Plasma Membrane
P36383	Gap junction alpha-7 protein (Connexin-45) (Cx45).	Plasma Membrane
P38570	[Integrin alpha-E precursor (Mucosal lymphocyte 1 antigen) (HML-1, antigen) (Integrin alpha-IEL) (CD103 antigen) [Contains: Integrin, alpha-E light chain; Integrin alpha-E heavy chain].]	Plasma Membrane
P40200	[T-cell surface protein tactile precursor (T cell-activated increased, late expression protein) (CD96 antigen).]	Plasma Membrane
P40238	[Thrombopoietin receptor precursor (TPO-R) (Myeloproliferative leukemia, protein) (C-mpl) (CD110 antigen).]	Plasma Membrane
P41143	Delta-type opioid receptor (DOR-1).	Plasma Membrane
P41231	[P2Y purinoceptor 2 (P2Y2) (P2U purinoceptor 1) (P2U1) (ATP receptor), (Purinergic receptor).]	Plasma Membrane
P41440	[Folate transporter 1 (Solute carrier family 19 member 1) (Placental, folate transporter) (FOLT) (Reduced folate carrier protein) (RFC), (Intestinal folate carrier) (IFC-1).]	Plasma Membrane
P60893	[Probable G-protein coupled receptor 85 (Super conserved receptor, expressed in brain 2).]	Plasma Membrane
Q00013	[55 kDa erythrocyte membrane protein (p55) (Membrane protein,, palmitoylated 1).]	Plasma Membrane
Q02763	[Angiopoietin-1 receptor precursor (EC 2.7.10.1) (Tyrosine-protein, kinase receptor TIE-2) (hTIE2) (Tyrosine-protein kinase receptor TEK), (p140 TEK) (Tunica interna endothelial cell kinase) (CD202b antigen).]	Plasma Membrane
Q02846	[Retinal guanylyl cyclase 1 precursor (EC 4.6.1.2) (Guanylate cyclase, 2D, retinal) (RETGC-1) (Rod outer segment membrane guanylate cyclase), (ROS-GC).]	Plasma Membrane
Q02978	[Mitochondrial 2-oxoglutarate/malate carrier protein (OGCP) (Solute, carrier family 25 member 11).]	Plasma Membrane
Q04656	[Copper-transporting ATPase 1 (EC 3.6.3.4) (Copper pump 1) (Menkes, disease-associated protein).]	Plasma Membrane
Q07157	[Tight junction protein ZO-1 (Zonula occludens protein 1) (Zona, occludens protein 1) (Tight junction protein 1).]	Plasma Membrane
Q12816	Trophinin (MAGE-D3 antigen).	Plasma Membrane
Q13255	Metabotropic glutamate receptor 1 precursor (mGluR1).	Plasma Membrane
Q99250	[Sodium channel protein type 2 subunit alpha (Sodium channel protein, type II subunit alpha) (Voltage-gated sodium channel subunit alpha, Nav1.2) (Sodium channel protein, brain II subunit alpha) (HBSC II).]	Plasma Membrane
Q99665	[Interleukin-12 receptor beta-2 chain precursor (IL-12 receptor beta-2), (IL-12R-beta2).]	Plasma Membrane
Q99680	Probable G-protein coupled receptor 22.	Plasma Membrane

Q99719	[Septin-5 (Peanut-like protein 1) (Cell division control-related, protein 1) (CDCrel-1).]	Plasma Membrane
Q99884	[Sodium-dependent proline transporter (Solute carrier family 6 member, 7).]	Plasma Membrane
Q9NRY6	[Phospholipid scramblase 3 (PL scramblase 3) (Ca(2+)-dependent, phospholipid scramblase 3).]	Plasma Membrane
Q9UHE8	[Metalloreductase STEAP1 (EC 1.16.1.-) (Six-transmembrane epithelial, antigen of prostate 1).]	Plasma Membrane
Q9UKF5	[ADAM 29 precursor (A disintegrin and metalloproteinase domain 29), (Cancer/testis antigen 73) (CT73).]	Plasma Membrane
Q9UKU6	[Thyrotropin-releasing hormone-degrading ectoenzyme (EC 3.4.19.6) (TRH-, degrading ectoenzyme) (TRH-DE) (TRH-specific aminopeptidase), (Thyroliberinase) (Pyroglutamyl-peptidase II) (PAP-II).]	Plasma Membrane
Q9UPC5	Probable G-protein coupled receptor 34.	Plasma Membrane
P05455	[Lupus La protein (Sjoegren syndrome type B antigen) (SS-B) (La, ribonucleoprotein) (La autoantigen).]	Protein Complex
O14818	[Proteasome subunit alpha type-7 (EC 3.4.25.1) (Proteasome subunit RC6-, 1) (Proteasome subunit XAPC7).]	Protein Complex
O15145	[Actin-related protein 2/3 complex subunit 3 (ARP2/3 complex 21 kDa, subunit) (p21-ARC).]	Protein Complex
O15372	[Eukaryotic translation initiation factor 3 subunit 3 (eIF-3 gamma), (eIF3 p40 subunit) (eIF3h).]	Protein Complex
O75426	F-box only protein 24.	Protein Complex
O75817	[Ribonuclease P protein subunit p20 (EC 3.1.26.5) (RNaseP protein p20), (hPOP7).]	Protein Complex
P07942	Laminin subunit beta-1 precursor (Laminin B1 chain).	Protein Complex
P08476	[Inhibin beta A chain precursor (Activin beta-A chain) (Erythroid, differentiation protein) (EDF).]	Protein Complex
P12829	[Myosin light polypeptide 4 (Myosin light chain 1, embryonic, muscle/atrial isoform) (Myosin light chain alkali, GT-1 isoform).]	Protein Complex
P13489	[Ribonuclease inhibitor (Ribonuclease/angiogenin inhibitor 1) (RAI), (Placental ribonuclease inhibitor) (RNase inhibitor) (RI).]	Protein Complex
P17302	[Gap junction alpha-1 protein (Connexin-43) (Cx43) (Gap junction 43 kDa, heart protein).]	Protein Complex
P46019	[Phosphorylase b kinase regulatory subunit alpha, liver isoform, (Phosphorylase kinase alpha L subunit).]	Protein Complex
P51587	[Breast cancer type 2 susceptibility protein (Fanconi anemia group D1, protein).]	Protein Complex
P54707	[Potassium-transporting ATPase alpha chain 2 (EC 3.6.3.10) (Proton, pump) (Non-gastric H(+)/K(+) ATPase subunit alpha).]	Protein Complex
P55884	[Eukaryotic translation initiation factor 3 subunit 9 (eIF-3-eta) (eIF3, p116) (eIF3 p110) (eIF3b) (Prt1 homolog) (hPrt1).]	Protein Complex
Q13454	Tumor suppressor candidate 3 (Protein N33).	Protein Complex
Q14152	[Eukaryotic translation initiation factor 3 subunit 10 (eIF-3-theta), (eIF3 p167) (eIF3 p180) (eIF3 p185) (eIF3a).]	Protein Complex
P01730	[T-cell surface glycoprotein CD4 precursor (T-cell surface antigen, T4/Leu-3).]	Protein Complex
P02675	Fibrinogen beta chain precursor [Contains: Fibrinopeptide B].	Protein Complex
P04843	[Dolichyl-diphosphooligosaccharide--protein glycosyltransferase 67 kDa, subunit precursor (EC 2.4.1.119) (Ribophorin I) (RPN-I).]	Protein Complex
P05538	HLA class II histocompatibility antigen, DX beta chain precursor.	Protein Complex
P23921	[Ribonucleoside-diphosphate reductase large subunit (EC 1.17.4.1), (Ribonucleoside-diphosphate reductase subunit M1) (Ribonucleotide, reductase large chain).]	Protein Complex
P28065	[Proteasome subunit beta type-9 precursor (EC 3.4.25.1) (Proteasome, subunit beta-1i) (Proteasome chain 7) (Macropain chain 7), (Multicatalytic endopeptidase complex chain 7) (RING12 protein) (Low, molecular mass protein 2).]	Protein Complex
P28072	[Proteasome subunit beta type-6 precursor (EC 3.4.25.1) (Proteasome, delta chain) (Macropain delta chain) (Multicatalytic endopeptidase, complex delta chain) (Proteasome subunit Y).]	Protein Complex
P29459	[Interleukin-12 subunit alpha precursor (IL-12A) (IL-12 subunit p35), (Cytotoxic lymphocyte maturation factor 35 kDa subunit) (CLMF p35) (NK, cell stimulatory factor chain 1) (NKSF1).]	Protein Complex
P31321	cAMP-dependent protein kinase type I-beta regulatory subunit.	Protein Complex

P38398	Breast cancer type 1 susceptibility protein (RING finger protein 53).	Protein Complex
P60660	[Myosin light polypeptide 6 (Smooth muscle and nonmuscle myosin light, chain alkali 6) (Myosin light chain alkali 3) (Myosin light chain 3), (MLC-3) (LC17).]	Protein Complex
P61353	60S ribosomal protein L27.	Protein Complex
P61966	[AP-1 complex subunit sigma-1A (Adapter-related protein complex 1, sigma-1A subunit) (Sigma-adaptin 1A) (Adaptor protein complex AP-1, sigma-1A subunit) (Golgi adaptor HA1/AP1 adaptin sigma-1A subunit), (Clathrin assembly protein complex 1 sigma-1A small chain) (Clathrin, coat assembly protein AP19) (HA1 19 kDa subunit) (Sigma 1a subunit of, AP-1 clathrin).]	Protein Complex
P62829	60S ribosomal protein L23 (Ribosomal protein L17).	Protein Complex
P68036	[Ubiquitin-conjugating enzyme E2 L3 (EC 6.3.2.19) (Ubiquitin-protein, ligase L3) (Ubiquitin carrier protein L3) (UbcH7) (E2-F1) (L-UBC).]	Protein Complex
P68871	[Hemoglobin subunit beta (Hemoglobin beta chain) (Beta-globin), [Contains: LVV-hemorphin-7].]	Protein Complex
P69905	Hemoglobin subunit alpha (Hemoglobin alpha chain) (Alpha-globin).	Protein Complex
P78552	[Interleukin-13 receptor alpha-1 chain precursor (IL-13R-alpha-1) (IL-, 13RA-1) (Cancer/testis antigen 19) (CT19) (CD213a1 antigen).]	Protein Complex
Q12840	[Kinesin heavy chain isoform 5A (Neuronal kinesin heavy chain) (NKHC), (Kinesin heavy chain neuron-specific 1).]	Protein Complex
Q13326	[Gamma-sarcoglycan (Gamma-SG) (35 kDa dystrophin-associated, glycoprotein) (35DAG).]	Protein Complex
Q13347	[Eukaryotic translation initiation factor 3 subunit 2 (eIF-3-beta), (eIF3 p36) (eIF3i) (TGF-beta receptor-interacting protein 1) (TRIP-1).]	Protein Complex
Q92993	[Histone acetyltransferase HTATIP (EC 2.3.1.48) (EC 2.3.1.-) (60 kDa, Tat interactive protein) (Tip60) (HIV-1 Tat interactive protein), (cPLA(2)-interacting protein).]	Protein Complex
Q93074	[Mediator of RNA polymerase II transcription subunit 12 (Thyroid, hormone receptor-associated protein complex 230 kDa component), (Trap230) (Activator-recruited cofactor 240 kDa component) (ARC240), (CAG repeat protein 45) (OPA-containing protein) (Trinucleotide, repeat-containing gene 11 protein).]	Protein Complex
Q99728	BRCA1-associated RING domain protein 1 (BARD-1).	Protein Complex
Q99759	[Mitogen-activated protein kinase kinase kinase 3 (EC 2.7.11.25), (MAPK/ERK kinase kinase 3) (MEK kinase 3) (MEKK 3).]	Protein Complex
Q9UK97	[F-box only protein 9 (Cross-immune reaction antigen 1) (Renal, carcinoma antigen NY-REN-57).]	Protein Complex
Q9UKB1	[F-box/WD repeat-containing protein 11 (F-box/WD repeat-containing, protein 1B) (F-box and WD repeats protein beta-TrCP2).]	Protein Complex
Q9UL18	[Eukaryotic translation initiation factor 2C 1 (eIF2C 1) (eIF-2C 1), (Argonaute-1) (Putative RNA-binding protein Q99).]	Protein Complex
O00566	[U3 small nucleolar ribonucleoprotein protein MPP10 (M phase, phosphoprotein 10).]	Protein Complex
O15169	Axin-1 (Axis inhibition protein 1) (hAxin).	Protein Complex
O15511	[Actin-related protein 2/3 complex subunit 5 (ARP2/3 complex 16 kDa, subunit) (p16-ARC).]	Protein Complex
O60282	[Kinesin heavy chain isoform 5C (Kinesin heavy chain neuron-specific, 2).]	Protein Complex
O75818	[Ribonuclease P protein subunit p40 (EC 3.1.26.5) (RNaseP protein p40), (RNase P subunit 1).]	Protein Complex
P07992	DNA excision repair protein ERCC-1.	Protein Complex
P10644	[cAMP-dependent protein kinase type I-alpha regulatory subunit (Tissue-, specific extinguisher 1) (TSE1).]	Protein Complex
P11047	Laminin subunit gamma-1 precursor (Laminin B2 chain).	Protein Complex
P11277	Spectrin beta chain, erythrocyte (Beta-I spectrin).	Protein Complex
P13533	[Myosin-6 (Myosin heavy chain 6) (Myosin heavy chain, cardiac muscle, alpha isoform) (MyHC-alpha).]	Protein Complex

P19474	[52 kDa Ro protein (Sjoegren syndrome type A antigen) (SS-A) (Ro(SS-A)), (52 kDa ribonucleoprotein autoantigen Ro/SS-A) (Tripartite motif-, containing protein 21) (RING finger protein 81).]	Protein Complex
P20585	[DNA mismatch repair protein Msh3 (Divergent upstream protein) (DUP), (Mismatch repair protein 1) (MRP1).]	Protein Complex
P43246	DNA mismatch repair protein Msh2 (MutS protein homolog 2).	Protein Complex
P45379	Troponin T, cardiac muscle (TnTc) (Cardiac muscle troponin T) (cTnT).	Protein Complex
P51617	Interleukin-1 receptor-associated kinase 1 (EC 2.7.11.1) (IRAK-1).	Protein Complex
P51665	[26S proteasome non-ATPase regulatory subunit 7 (26S proteasome, regulatory subunit rpn8) (26S proteasome regulatory subunit S12), (Proteasome subunit p40) (Mov34 protein homolog).]	Protein Complex
P52701	[DNA mismatch repair protein MSH6 (MutS-alpha 160 kDa subunit) (G/T, mismatch-binding protein) (GTBP) (GTMBP) (p160).]	Protein Complex
P53396	[ATP-citrate synthase (EC 2.3.3.8) (ATP-citrate (pro-S-)-lyase), (Citrate cleavage enzyme).]	Protein Complex
Q92889	[DNA repair endonuclease XPF (EC 3.1.-.-) (DNA excision repair protein, ERCC-4) (DNA-repair protein complementing XP-F cells) (Xeroderma, pigmentosum group F-complementing protein).]	Protein Complex
Q92901	60S ribosomal protein L3-like.	Protein Complex
O95782	[AP-2 complex subunit alpha-1 (Adapter-related protein complex 2 alpha-, 1 subunit) (Alpha-adaptin A) (Adaptor protein complex AP-2 alpha-1, subunit) (Clathrin assembly protein complex 2 alpha-A large chain), (100 kDa coated vesicle protein A) (Plasma membrane adaptor HA2/AP2, adaptin alpha A subunit).]	Protein Complex
P25391	Laminin subunit alpha-1 precursor (Laminin A chain).	Protein Complex
P02679	Fibrinogen gamma chain precursor.	Protein Complex
P04844	[Dolichyl-diphosphooligosaccharide--protein glycosyltransferase 63 kDa, subunit precursor (EC 2.4.1.119) (Ribophorin II) (RPN-II) (RIBIIR).]	Protein Complex
P23434	Glycine cleavage system H protein, mitochondrial precursor.	Protein Complex
P23588	Eukaryotic translation initiation factor 4B (eIF-4B).	Protein Complex
P24386	[Rab proteins geranylgeranyltransferase component A 1 (Rab escort, protein 1) (REP-1) (Choroideraemia protein) (TCD protein).]	Protein Complex
P27986	[Phosphatidylinositol 3-kinase regulatory subunit alpha (PI3-kinase p85, subunit alpha) (PtdIns-3-kinase p85-alpha) (PI3K).]	Protein Complex
P28066	[Proteasome subunit alpha type-5 (EC 3.4.25.1) (Proteasome zeta chain), (Macropain zeta chain) (Multicatalytic endopeptidase complex zeta, chain).]	Protein Complex
P30679	[Guanine nucleotide-binding protein alpha-15 subunit (G alpha-15) (G, alpha-16).]	Protein Complex
P32969	60S ribosomal protein L9.	Protein Complex
P33176	Kinesin heavy chain (Ubiquitous kinesin heavy chain) (UKHC).	Protein Complex
P36543	[Vacuolar ATP synthase subunit E 1 (EC 3.6.3.14) (V-ATPase subunit E 1), (Vacuolar proton pump subunit E 1) (V-ATPase 31 kDa subunit) (P31).]	Protein Complex
P60228	[Eukaryotic translation initiation factor 3 subunit 6 (eIF-3 p48), (eIF3e) (Viral integration site protein INT-6 homolog).]	Protein Complex
P60661	[Myosin light polypeptide 6 (Myosin light chain alkali 3) (Myosin light, chain 3) (MLC-3) (LC17).]	Protein Complex
P60842	[Eukaryotic initiation factor 4A-I (EC 3.6.1.-) (ATP-dependent RNA, helicase eIF4A-1) (eIF4A-I) (eIF-4A-I).]	Protein Complex
P61313	60S ribosomal protein L15.	Protein Complex
P78345	Ribonuclease P protein subunit p38 (EC 3.1.26.5) (RNaseP protein p38).	Protein Complex
Q00653	[Nuclear factor NF-kappa-B p100 subunit (DNA-binding factor KBF2), (H2TF1) (Lymphocyte translocation chromosome 10) (Oncogene Lyt-10), (Lyt10) [Contains: Nuclear factor NF-kappa-B p52 subunit].]	Protein Complex
Q04637	[Eukaryotic translation initiation factor 4 gamma 1 (eIF-4-gamma 1), (eIF-4G1) (eIF-4G 1) (p220).]	Protein Complex

Q13144	[Translation initiation factor eIF-2B subunit epsilon (eIF-2B GDP-GTP, exchange factor subunit epsilon).]	Protein Complex
Q99436	[Proteasome subunit beta type-7 precursor (EC 3.4.25.1) (Proteasome, subunit Z) (Macropain chain Z) (Multicatalytic endopeptidase complex, chain Z).]	Protein Complex
Q9Y2L1	[Exosome complex exonuclease RRP44 (EC 3.1.13.-) (Ribosomal RNA-, processing protein 44) (DIS3 protein homolog).]	Protein Complex
O00160	Myosin-If (Myosin-Ie).	Protein Complex
O00329	[Phosphatidylinositol-4,5-bisphosphate 3-kinase catalytic subunit delta, isoform (EC 2.7.1.153) (PI3-kinase p110 subunit delta) (PtdIns-3-, kinase p110) (PI3K) (p110delta).]	Protein Complex
O14775	[Guanine nucleotide-binding protein subunit beta-5 (Transducin beta, chain 5) (Gbeta5).]	Protein Complex
O15066	[Kinesin-like protein KIF3B (Microtubule plus end-directed kinesin, motor 3B) (HH0048).]	Protein Complex
O15371	[Eukaryotic translation initiation factor 3 subunit 7 (eIF-3-zeta), (eIF3 p66) (eIF3d).]	Protein Complex
O75593	[Forkhead box protein H1 (Forkhead activin signal transducer 1) (Fast-, 1) (hFAST-1) (Forkhead activin signal transducer 2) (Fast-2).]	Protein Complex
P10155	[60 kDa SS-A/Ro ribonucleoprotein (60 kDa Ro protein) (60 kDa, ribonucleoprotein Ro) (RoRNP) (Ro 60 kDa autoantigen) (TROVE domain, family member 2) (Sjoegren syndrome type A antigen) (SS-A) (Sjoegren, syndrome antigen A2).]	Protein Complex
P12883	[Myosin-7 (Myosin heavy chain 7) (Myosin heavy chain, cardiac muscle, beta isoform) (MyHC-beta) (Myosin heavy chain slow isoform) (MyHC-, slow).]	Protein Complex
P19087	[Guanine nucleotide-binding protein G(t), alpha-2 subunit (Transducin, alpha-2 chain).]	Protein Complex
P46977	[Dolichyl-diphosphooligosaccharide--protein glycosyltransferase subunit, STT3A (EC 2.4.1.119) (Oligosaccharyl transferase subunit STT3A) (STT3-, A) (B5) (Integral membrane protein 1) (TMC).]	Protein Complex
P49720	[Proteasome subunit beta type-3 (EC 3.4.25.1) (Proteasome theta chain), (Proteasome chain 13) (Proteasome component C10-II).]	Protein Complex
P52732	[Kinesin-like protein KIF11 (Kinesin-related motor protein Eg5), (Kinesin-like spindle protein HKSP) (Thyroid receptor-interacting, protein 5) (TRIP-5) (Kinesin-like protein 1).]	Protein Complex
Q14232	[Translation initiation factor eIF-2B subunit alpha (eIF-2B GDP-GTP, exchange factor subunit alpha).]	Protein Complex
Q14314	Fibroleukin precursor (Fibrinogen-like protein 2) (pT49).	Protein Complex
Q15008	[26S proteasome non-ATPase regulatory subunit 6 (26S proteasome, regulatory subunit S10) (p42A) (Proteasome regulatory particle subunit, p44S10) (Phosphonoformate immuno-associated protein 4) (Breast cancer-, associated protein SGA-113M).]	Protein Complex
Q15056	[Eukaryotic translation initiation factor 4H (eIF-4H) (Williams-Beuren, syndrome chromosome region 1 protein).]	Protein Complex
Q15904	[Vacuolar ATP synthase subunit S1 precursor (EC 3.6.3.14) (V-ATPase, subunit S1) (V-ATPase S1 accessory protein) (V-ATPase Ac45 subunit), (Protein XAP-3).]	Protein Complex
Q8IUD2	[ELKS/RAB6-interacting/CAST family member 1 (RAB6-interacting protein, 2) (ERC protein 1).]	Protein Complex
O94913	[Pre-mRNA cleavage complex 2 protein Pcf11 (Pre-mRNA cleavage complex, II protein Pcf11) (Fragment).]	Protein Complex
O94973	[AP-2 complex subunit alpha-2 (Adapter-related protein complex 2 alpha-, 2 subunit) (Alpha-adaptin C) (Adaptor protein complex AP-2 alpha-2, subunit) (Clathrin assembly protein complex 2 alpha-C large chain), (100 kDa coated vesicle protein C) (Plasma membrane adaptor HA2/AP2, adaptin alpha C subunit) (Huntingtin-interacting protein HYPJ).]	Protein Complex
P02042	Hemoglobin subunit delta (Hemoglobin delta chain) (Delta-globin).	Protein Complex
P02671	Fibrinogen alpha chain precursor [Contains: Fibrinopeptide A].	Protein Complex
P02746	Complement C1q subcomponent subunit B precursor.	Protein Complex
P02794	[Ferritin heavy chain (EC 1.16.3.1) (Ferritin H subunit) (Cell, proliferation-inducing gene 15 protein).]	Protein Complex
P03950	Angiogenin precursor (EC 3.1.27.-) (Ribonuclease 5) (RNase 5).	Protein Complex

P06730	[Eukaryotic translation initiation factor 4E (eIF4E) (eIF-4E) (mRNA, cap-binding protein) (eIF-4F 25 kDa subunit).]	Protein Complex
P25054	Adenomatous polyposis coli protein (Protein APC).	Protein Complex
P35222	Catenin beta-1 (Beta-catenin).	Protein Complex
P35749	[Myosin-11 (Myosin heavy chain 11) (Myosin heavy chain, smooth muscle, isoform) (SMMHC).]	Protein Complex
P40306	[Proteasome subunit beta type-10 precursor (EC 3.4.25.1) (Proteasome, subunit beta-2i) (Proteasome MECl-1) (Macropain subunit MECl-1), (Multicatalytic endopeptidase complex subunit MECl-1).]	Protein Complex
P61803	[Dolichyl-diphosphooligosaccharide--protein glycosyltransferase subunit, DAD1 (EC 2.4.1.119) (Oligosaccharyl transferase subunit DAD1), (Defender against cell death 1) (DAD-1).]	Protein Complex
P62191	[26S protease regulatory subunit 4 (P26s4) (Proteasome 26S subunit, ATPase 1).]	Protein Complex
Q02108	[Guanylate cyclase soluble subunit alpha-3 (EC 4.6.1.2) (GCS-alpha-3), (Soluble guanylate cyclase large subunit) (GCS-alpha-1).]	Protein Complex
Q04771	[Activin receptor type-1 precursor (EC 2.7.11.30) (Activin receptor, type I) (ACTR-I) (Serine/threonine-protein kinase receptor R1) (SKR1), (Activin receptor-like kinase 2) (ALK-2) (TGF-B superfamily receptor, type I) (TSR-I).]	Protein Complex
Q08334	[Interleukin-10 receptor beta chain precursor (IL-10R-B) (IL-10R2), (Cytokine receptor family 2 member 4) (Cytokine receptor class-II, member 4) (CRF2-4) (CDw210b antigen).]	Protein Complex
Q99460	[26S proteasome non-ATPase regulatory subunit 1 (26S proteasome, regulatory subunit RPN2) (26S proteasome regulatory subunit S1) (26S, proteasome subunit p112).]	Protein Complex
Q99575	Ribonucleases P/MRP protein subunit POP1 (EC 3.1.26.5) (hPOP1).	Protein Complex
Q99613	[Eukaryotic translation initiation factor 3 subunit 8 (eIF3 p110), (eIF3c).]	Protein Complex
Q9NRD0	F-box only protein 8 (F-box/SEC7 protein FBS).	Protein Complex
Q9UI10	[Translation initiation factor eIF-2B subunit delta (eIF-2B GDP-GTP, exchange factor subunit delta).]	Protein Complex
Q15544	[Transcription initiation factor TFIID subunit 11 (Transcription, initiation factor TFIID 28 kDa subunit) (TAF(II)28) (TAFII-28), (TAFII28) (TFIID subunit p30-beta).]	Transcription
Q16594	[Transcription initiation factor TFIID subunit 9 (Transcription, initiation factor TFIID 31 kDa subunit) (TAFII-31) (TAFII-32), (TAFII32) (STAF31/32).]	Transcription
P21675	[Transcription initiation factor TFIID subunit 1 (EC 2.7.11.1), (Transcription initiation factor TFIID 250 kDa subunit) (TAF(II)250), (TAFII-250) (TAFII250) (TBP-associated factor 250 kDa) (p250) (Cell, cycle gene 1 protein).]	Transcription
P29374	[AT-rich interactive domain-containing protein 4A (ARID domain-, containing protein 4A) (Retinoblastoma-binding protein 1) (RBBP-1).]	Transcription
P20226	[TATA-box-binding protein (TATA-box factor) (TATA-binding factor) (TATA, sequence-binding protein) (Transcription initiation factor TFIID TBP, subunit).]	Transcription
P49848	[Transcription initiation factor TFIID subunit 6 (Transcription, initiation factor TFIID 70 kDa subunit) (TAF(II)70) (TAFII-70) (TAFII-, 80) (TAFII80).]	Transcription
P50750	[Cell division protein kinase 9 (EC 2.7.11.22) (EC 2.7.11.23) (Cyclin-, dependent kinase 9) (Serine/threonine-protein kinase PITALRE) (C-2K), (Cell division cycle 2-like protein kinase 4).]	Transcription
Q15406	[Orphan nuclear receptor NR6A1 (Germ cell nuclear factor) (GCNF), (hGCNF) (Retinoid receptor-related testis-specific receptor) (RTR), (hRTR).]	Transcription
Q15542	[Transcription initiation factor TFIID subunit 5 (Transcription, initiation factor TFIID 100 kDa subunit) (TAF(II)100) (TAFII-100), (TAFII100).]	Transcription
Q15796	[Mothers against decapentaplegic homolog 2 (SMAD 2) (Mothers against, DPP homolog 2) (Mad-related protein 2) (hMAD-2) (JV18-1) (hSMAD2).]	Transcription

Q99814	[Endothelial PAS domain-containing protein 1 (EPAS-1) (Member of PAS, protein 2) (Basic-helix-loop-helix-PAS protein MOP2) (Hypoxia-, inducible factor 2 alpha) (HIF-2 alpha) (HIF2 alpha) (HIF-1 alpha-like, factor) (HLF).]	Transcription
O00268	[Transcription initiation factor TFIID subunit 4 (TBP-associated factor, 4) (Transcription initiation factor TFIID 135 kDa subunit), (TAF(II)135) (TAFII-135) (TAFII135) (TAFII-130) (TAFII130).]	Transcription
O00472	RNA polymerase II elongation factor ELL2.	Transcription
Q13461	[Forkhead box protein E3 (Forkhead-related protein FKHL12) (Forkhead-, related transcription factor 8) (FREAC-8).]	Transcription
Q15543	[Transcription initiation factor TFIID subunit 13 (Transcription, initiation factor TFIID 18 kDa subunit) (TAF(II)18) (TAFII-18), (TAFII18).]	Transcription
Q16514	[Transcription initiation factor TFIID subunit 12 (Transcription, initiation factor TFIID 20/15 kDa subunits) (TAFII-20/TAFII-15), (TAFII20/TAFII15).]	Transcription
P35269	[Transcription initiation factor IIF subunit alpha (EC 2.7.11.1), (TFIIF-alpha) (General transcription factor IIF subunit 1), (Transcription initiation factor RAP74) (General transcription factor, IIF polypeptide 1 74 kDa subunit protein).]	Transcription
Q9NVW2	[RING finger protein 12 (LIM domain-interacting RING finger protein), (RING finger LIM domain-binding protein) (R-LIM) (Renal carcinoma, antigen NY-REN-43).]	Transcription
O00116	[Alkyldihydroxyacetonephosphate synthase, peroxisomal precursor, (EC 2.5.1.26) (Alkyl-DHAP synthase) (Alkylglycerone-phosphate, synthase) (Aging-associated gene 5 protein).]	Vesicle
O14579	Coatomer subunit epsilon (Epsilon-coat protein) (Epsilon-COP).	Vesicle
O14994	Synapsin-3 (Synapsin III).	Vesicle
P11717	[Cation-independent mannose-6-phosphate receptor precursor (CI Man-6-P, receptor) (CI-MPR) (M6PR) (Insulin-like growth factor 2 receptor), (Insulin-like growth factor II receptor) (IGF-II receptor) (M6P/IGF2, receptor) (M6P/IGF2R) (300 kDa mannose 6-phosphate receptor) (MPR 300), (MPR300) (CD222 antigen).]	Vesicle
P19801	[Amiloride-sensitive amine oxidase [copper-containing] precursor, (EC 1.4.3.6) (Diamine oxidase) (DAO) (Amiloride-binding protein) (ABP), (Histaminase) (Kidney amine oxidase) (KAO).]	Vesicle
P51659	[Peroxisomal multifunctional enzyme type 2 (MFE-2) (D-bifunctional, protein) (DBP) (17-beta-hydroxysteroid dehydrogenase 4) (17-beta-HSD, 4) (D-3-hydroxyacyl-CoA dehydratase) (EC 4.2.1.107) (3-alpha,7-, alpha,12-alpha-trihydroxy-5-beta-cholest-24-enoyl-CoA hydratase) (3-, hydroxyacyl-CoA dehydrogenase) (EC 1.1.1.35).]	Vesicle
P51681	[C-C chemokine receptor type 5 (C-C CKR-5) (CC-CKR-5) (CCR-5) (CCR5), (HIV-1 fusion coreceptor) (CHEMR13) (CD195 antigen).]	Vesicle
P53621	[Coatomer subunit alpha (Alpha-coat protein) (Alpha-COP) (HEPCOP) (HEP-, COP) [Contains: Xenin (Xenopsin-related peptide); Proxenin].]	Vesicle
P53992	Protein transport protein Sec24C (SEC24-related protein C).	Vesicle
Q15036	Sorting nexin-17.	Vesicle
Q15126	Phosphomevalonate kinase (EC 2.7.4.2) (PMKase).	Vesicle
O95486	Protein transport protein Sec24A (SEC24-related protein A) (Fragment).	Vesicle
P21579	Synaptotagmin-1 (Synaptotagmin I) (SytI) (p65).	Vesicle
P35606	Coatomer subunit beta' (Beta'-coat protein) (Beta'-COP) (p102).	Vesicle
Q9UBF2	Coatomer subunit gamma-2 (Gamma-2-coat protein) (Gamma-2-COP).	Vesicle
O15228	[Dihydroxyacetone phosphate acyltransferase (EC 2.3.1.42) (DHAP-AT), (DAP-AT) (Glycerone-phosphate O-acyltransferase) (Acyl-, CoA:dihydroxyacetonephosphateacyltransferase).]	Vesicle
O43933	[Peroxisome biogenesis factor 1 (Peroxin-1) (Peroxisome biogenesis, disorder protein 1).]	Vesicle

O60333	Kinesin-like protein KIF1B (Klp).	Vesicle
O60664	[Mannose-6-phosphate receptor-binding protein 1 (Cargo selection, protein TIP47) (47 kDa mannose 6-phosphate receptor-binding protein), (47 kDa MPR-binding protein) (Placental protein 17) (PP17).]	Vesicle
P20930	Filaggrin.	Vesicle
Q13907	[Isopentenyl-diphosphate Delta-isomerase 1 (EC 5.3.3.2) (IPP isomerase, 1) (Isopentenyl pyrophosphate isomerase 1) (IPPI1).]	Vesicle
Q14833	Metabotropic glutamate receptor 4 precursor (mGluR4).	Vesicle
O94855	Protein transport protein Sec24D (SEC24-related protein D).	Vesicle
P00533	[Epidermal growth factor receptor precursor (EC 2.7.10.1) (Receptor, tyrosine-protein kinase ErbB-1).]	Vesicle
P02786	[Transferrin receptor protein 1 (TfR1) (TR) (TfR) (Trfr) (CD71 antigen), (T9) (p90) [Contains: Transferrin receptor protein 1, serum form, (sTfR)].]	Vesicle
P27544	LAG1 longevity assurance homolog 1 (UOG-1 protein) (Protein LAG1).	Vesicle
P31512	[Dimethylaniline monooxygenase [N-oxide-forming] 4 (EC 1.14.13.8), (Hepatic flavin-containing monooxygenase 4) (FMO 4) (Dimethylaniline, oxidase 4).]	Vesicle
P32856	Syntaxin-2 (Epimorphin).	Vesicle
P33261	[Cytochrome P450 2C19 (EC 1.14.13.80) ((R)-limonene 6-monooxygenase), (EC 1.14.13.48) ((S)-limonene 6-monooxygenase) (EC 1.14.13.49) ((S)-, limonene 7-monooxygenase) (CYPIIC19) (P450-11A) (Mephenytoin 4-, hydroxylase) (CYPIIC17) (P450-254C).]	Vesicle
P37058	[Testosterone 17-beta-dehydrogenase 3 (EC 1.1.1.64) (17-beta-HSD 3), (Testicular 17-beta-hydroxysteroid dehydrogenase).]	Vesicle
Q08426	[Peroxisomal bifunctional enzyme (PBE) (PBFE) [Includes: Enoyl-CoA, hydratase/3,2-trans-enoyl-CoA isomerase (EC 5.3.3.8) (EC 4.2.1.17); 3-, hydroxyacyl-CoA dehydrogenase (EC 1.1.1.35)].]	Vesicle
Q99424	[Acyl-coenzyme A oxidase 2, peroxisomal (EC 1.17.99.3) (3-alpha,7-, alpha,12-alpha-trihydroxy-5-beta-cholestanoyl-CoA 24-hydroxylase) (3-, alpha,7-alpha,12-alpha-trihydroxy-5-beta-cholestanoyl-CoA oxidase), (Trihydroxycoprostanoyl-CoA oxidase) (THCCox) (THCA-CoA oxidase).]	Vesicle
Q99698	Lysosomal-trafficking regulator (Beige homolog).	Vesicle
Q9NYQ2	[Hydroxyacid oxidase 2 (EC 1.1.3.15) (HAOX2) ((S)-2-hydroxy-acid, oxidase, peroxisomal) (Medium chain alpha-hydroxy acid oxidase), (Medium-chain L-2-hydroxy acid oxidase).]	Vesicle
Q9UKU0	[Long-chain-fatty-acid--CoA ligase 6 (EC 6.2.1.3) (Long-chain acyl-CoA, synthetase 6) (LACS 6).]	Vesicle
Q9UL25	Ras-related protein Rab-21.	Vesicle
O15254	[Acyl-coenzyme A oxidase 3, peroxisomal (EC 1.3.3.6) (Pristanoyl-CoA, oxidase) (Branched-chain acyl-CoA oxidase) (BRCACox).]	Vesicle
O60344	Endothelin-converting enzyme 2 (EC 3.4.24.71) (ECE-2).	Vesicle
O75146	[Huntingtin-interacting protein 1-related protein (Hip1-related) (Hip, 12).]	Vesicle
O75351	[Vacuolar protein sorting-associating protein 4B (Suppressor of K(+), transport growth defect 1) (Protein SKD1).]	Vesicle
P13473	[Lysosome-associated membrane glycoprotein 2 precursor (LAMP-2) (CD107b, antigen).]	Vesicle
P18825	[Alpha-2C adrenergic receptor (Alpha-2C adrenoceptor) (Alpha-2C, adrenoreceptor) (Alpha-2 adrenergic receptor subtype C4).]	Vesicle
P19224	[UDP-glucuronosyltransferase 1-6 precursor (EC 2.4.1.17) (UDP-, glucuronosyltransferase 1A6) (UDPGT) (UGT1*6) (UGT1-06) (UGT1.6) (UGT-, 1F) (UGT1F) (Phenol-metabolizing UDP-glucuronosyltransferase).]	Vesicle
Q92902	Hermansky-Pudlak syndrome 1 protein.	Vesicle
O95405	[Zinc finger FYVE domain-containing protein 9 (Mothers against, decapentaplegic homolog-interacting protein) (Madh-interacting, protein) (Smad anchor for receptor activation) (Receptor activation, anchor) (hSARA) (Novel serine protease) (NSP).]	Vesicle

O95822	Malonyl-CoA decarboxylase, mitochondrial precursor (EC 4.1.1.9) (MCD).	Vesicle
P02787	[Serotransferrin precursor (Transferrin) (Siderophilin) (Beta-1-metal-, binding globulin).]	Vesicle
P06133	[UDP-glucuronosyltransferase 2B4 precursor (EC 2.4.1.17) (UDPGT), (Hyodeoxycholic acid) (HLUG25) (UDPGTh-1).]	Vesicle
P22307	[Non-specific lipid-transfer protein (EC 2.3.1.176) (Propanoyl-CoA C-, acyltransferase) (NSL-TP) (Sterol carrier protein 2) (SCP-2) (Sterol, carrier protein X) (SCP-X) (SCP-chi) (SCPX).]	Vesicle
P31513	[Dimethylaniline monooxygenase [N-oxide-forming] 3 (EC 1.14.13.8), (Hepatic flavin-containing monooxygenase 3) (FMO 3) (Dimethylaniline, oxidase 3) (FMO form 2) (FMO II).]	Vesicle
P35247	[Pulmonary surfactant-associated protein D precursor (SP-D) (PSP-D), (Lung surfactant protein D).]	Vesicle
P35504	[UDP-glucuronosyltransferase 1-5 precursor (EC 2.4.1.17) (UDP-, glucuronosyltransferase 1A5) (UDPGT) (UGT1*5) (UGT1-05) (UGT1.5) (UGT-, 1E) (UGT1E).]	Vesicle
Q02928	[Cytochrome P450 4A11 precursor (EC 1.14.15.3) (CYPIVA11) (Fatty acid, omega-hydroxylase) (P-450 HK omega) (Lauric acid omega-hydroxylase), (CYP4AII) (P450-HL-omega).]	Vesicle
Q13393	[Phospholipase D1 (EC 3.1.4.4) (PLD 1) (Choline phosphatase 1), (Phosphatidylcholine-hydrolyzing phospholipase D1) (hPLD1).]	Vesicle
Q99523	[Sortilin precursor (Neurotensin receptor 3) (NTR3) (NT3) (Glycoprotein, 95) (Gp95) (100 kDa NT receptor).]	Vesicle
Q9NYQ3	[Hydroxyacid oxidase 2 (EC 1.1.3.15) (HAOX2) ((S)-2-hydroxy-acid, oxidase, peroxisomal) (Long chain alpha-hydroxy acid oxidase) (Long-, chain L-2-hydroxy acid oxidase).]	Vesicle
Q9UJM8	Hydroxyacid oxidase 1 (EC 1.1.3.15) (HAOX1) (Glycolate oxidase) (GOX).	Vesicle
Q9UL26	Ras-related protein Rab-22A (Rab-22).	Vesicle
