# Supplementary material for: The morphology and biochemistry of nanostructures provide evidence for synthesis and signaling functions in human cerebrospinal fluid
Source: Cerebrospinal Fluid Res. 2009 Sep 7;6:10. doi: 10.1186/1743-8454-6-10 (PMC2746175; doi:10.1186/1743-8454-6-10)
Supplement: Additional file 2 — CSF proteins from the P3-enriched pellet and the S3 supernatant. CSF proteins identified from a single, high resolution shotgun sequencing run of two sets of P3 and S3 samples from one participant collected on two independent occasions (sample #s 3 & 3' in Table 1). [file 1743-8454-6-10-S2.pdf]

Supplementary Table 2. CSF Proteins from the P3-enriched pellet and the S3 supernatant.

CSF samples from one participant, collected on two separate occasions (Visit 1 and 2), were separated into P3 and S3 fractions, as described in Methods. The two P3 and S3 samples were treated with trypsin, carboxymethylated, and subjected to a single high resolution LC-MS/MS analysis. Proteins are ordered by P3 followed by S3 of Data 3, then P3 followed by S3 of Data 4. Within each sample, they are ordered by the protein GO component assignment in alphabetical order

P3 of Data 3, visit 1		
Uniprot Number	Protein Name	GO Component CATEGORY
O14924	Regulator of G-protein signaling 12 (RGS12).	Chromosomal
Q8NI77	Kinesin-like protein KIF18A.	Cytoskeletal
Q99469	[SH3 and cysteine-rich domain-containing protein (SRC homology 3 and, cysteine-rich domain protein).]	Cytosolic
Q9NZ09	[Ubiquitin-associated protein 1 (UBAP) (Nasopharyngeal carcinoma-, associated gene 20 protein).]	Cytosolic
O75912	[Diacylglycerol kinase iota (EC 2.7.1.107) (Diglyceride kinase iota), (DGK-iota) (DAG kinase iota).]	Cytosolic
P00492	[Hypoxanthine-guanine phosphoribosyltransferase (EC 2.4.2.8) (HGPRT), (HGPRTase).]	Cytosolic
P12272	[Parathyroid hormone-related protein precursor (PTH-rP) (PTHrP), [Contains: PTHrP[1-36]; PTHrP[38-94]; Osteostatin (PTHrP[107-139])].]	Cytosolic
Q5K4E3	[Polyserase-2 precursor (EC 3.4.21.-) (Polyserine protease 2) (Serine, protease 36).]	Cytosolic
Q9P2V4	[Leucine-rich repeat, immunoglobulin-like domain and transmembrane, domain-containing protein 1 precursor (Leucine-rich repeat-containing, protein 21) (Photoreceptor-associated LRR superfamily protein), (Retina-specific protein PAL).]	ER
P01009	[Alpha-1-antitrypsin precursor (Alpha-1 protease inhibitor) (Alpha-1-, antiproteinase).]	Extracellular
P01770	Ig heavy chain V-III region NIE.	Extracellular
P41222	[Prostaglandin-H2 D-isomerase precursor (EC 5.3.99.2) (Lipocalin-type, prostaglandin-D synthase) (Glutathione-independent PGD synthetase), (Prostaglandin-D2 synthase) (PGD2 synthase) (PGDS2) (PGDS) (Beta-trace, protein) (Cerebrin-28).]	Extracellular
P11487	[INT-2 proto-oncogene protein precursor (Fibroblast growth factor 3), (FGF-3) (HBGF-3).]	Extracellular
P02768	Serum albumin precursor.	Extracellular
P01023	Alpha-2-macroglobulin precursor (Alpha-2-M).	Extracellular
Q12979	Active breakpoint cluster region-related protein.	Go Component not found
Q9NQ18	CAP-binding protein complex interacting protein 1 (Fragment).	Go Component not found
Q76MJ5	[Serine/threonine-protein kinase/endoribonuclease IRE2 precursor, (Inositol-requiring protein 2) (hIRE2p) (IRE1b) (Ire1-beta), (Endoplasmic reticulum-to-nucleus signaling 2) [Includes:, Serine/threonine-protein kinase (EC 2.7.11.1); Endoribonuclease, (EC 3.1.26.-)].]	Go Component not found
Q8N1F7	[Nuclear pore complex protein Nup93 (Nucleoporin Nup93) (93 kDa, nucleoporin).]	Go Component not found
O43586	[Proline-serine-threonine phosphatase-interacting protein 1 (PEST, phosphatase-interacting protein 1) (CD2-binding protein 1) (H-PIP).]	Go Component not found

Q86X06	BRD1 protein.	Go Component not found
Q9NX55	Huntingtin-interacting protein HYPK (Huntingtin yeast partner K).	Go Component not found
Q12955	Ankyrin-3 (ANK-3) (Ankyrin-G).	Go Component not found
Q5TBS2	[OTTHUMP00000018460 (Heterogeneous nuclear ribonucleoprotein A1-like,, isoform CRA_b).]	Go Component not found
O60811	PRAME family member 2.	Go Component not found
Q5JT25	Ras-related protein Rab-41.	Go Component not found
Q5VZP5	Inactive dual specificity phosphatase 27.	Go Component not found
Q6NVZ9	LOC729176 protein (Fragment).	Go Component not found
Q8N201	Integrator complex subunit 1 (Int1).	Go Component not found
Q86U17	Serpin A11 precursor.	Go Component not found
Q8IYJ1	Copine-9 (Copine IX).	Go Component not found
Q8WYC2	Dystrophin (Fragment).	Go Component not found
Q9BY14	[Testis-expressed protein 101 precursor (Scleroderma-associated, autoantigen) (Cell surface receptor NYD-SP8) (Spermatogenesis-related, gene protein).]	Go Component not found
Q96A35	39S ribosomal protein L24, mitochondrial precursor (L24mt) (MRP-L24).	Go Component not found
P13762	HLA class II histocompatibility antigen, DR-W53 beta chain precursor.	Go Component not found
Q13201	[Multimerin-1 precursor (Endothelial cell multimerin 1) (EMILIN-4), (Elastin microfibril interface located protein 4) (Elastin microfibril, interfacer 4).]	Go Component not found
P59541	Taste receptor type 2 member 47 (T2R47).	Go Component not found
P14543	Nidogen-1 precursor (Entactin).	Go Component not found
Q6MZQ6	Putative uncharacterized protein DKFZp686G11190.	Go Component not found
Q6PK57	LOC554175 protein.	Go Component not found
Q6VB91	COBW domain-containing protein 3.	Go Component not found
Q5SZL2	[Coiled-coil domain-containing protein C6orf204 (Serologically defined, breast cancer antigen NY-BR-15).]	Go Component not found
Q96CF2	[Charged multivesicular body protein 4c (Chromatin-modifying protein, 4c) (CHMP4c) (Vacuolar protein-sorting-associated protein 7-3) (SNF7-, 3) (hSnf7-3) (SNF7 homolog associated with Alix 3).]	Go Component not found
Q9H3T8	Strawberry notch homolog 1 (Protein MOP-3).	Go Component not found
Q9HBQ9	Putative uncharacterized protein.	Go Component not found
Q8NGX2	Olfactory receptor 2T35 (Olfactory receptor OR1-66).	Go Component not found
Q6U7Q0	[Zinc finger protein 322A (Zinc finger protein 388) (Zinc finger, protein 489).]	Go Component not found
Q6P1Q0	[LETM1 domain-containing protein 1 (Cervical cancer 1 proto-oncogene, protein p40) (Cervical cancer proto-oncogene 2 protein) (HCRR-2).]	Go Component not found
Q8N1L7	CDNA FLJ40039 fis, clone SYNOV2000397.	Go Component not found
Q8N8Z0	Putative uncharacterized protein C12orf27.	Go Component not found
Q5TCY1	Tau-tubulin kinase 1 (EC 2.7.11.1) (Brain-derived tau kinase).	Go Component not found
Q9BS16	[Centromere protein K (CENP-K) (Interphase centromere complex protein, 37) (Protein AF-5alpha) (p33).]	Go Component not found

Q01740	[Dimethylaniline monooxygenase [N-oxide-forming] 1 (EC 1.14.13.8), (Fetal hepatic flavin-containing monooxygenase 1) (FMO 1), (Dimethylaniline oxidase 1).]	Go Component not found
O14744	[Protein arginine N-methyltransferase 5 (EC 2.1.1.125) (EC 2.1.1.-), (Shk1 kinase-binding protein 1 homolog) (SKB1Hs) (Jak-binding protein, 1) (72 kDa ICln-binding protein).]	Go Component not found
O43557	[Tumor necrosis factor ligand superfamily member 14 (Herpesvirus entry, mediator-ligand) (HVEM-L) (CD258 antigen) [Contains: Tumor necrosis, factor ligand superfamily member 14, membrane form; Tumor necrosis, factor ligand superfamily member 14, soluble form].]	Go Component not found
P12955	[Xaa-Pro dipeptidase (EC 3.4.13.9) (X-Pro dipeptidase) (Proline, dipeptidase) (Prolidase) (Imidodipeptidase).]	Go Component not found
P01034	[Cystatin-C precursor (Cystatin-3) (Neuroendocrine basic polypeptide), (Gamma-trace) (Post-gamma-globulin).]	Go Component not found
Q59FU7	G protein-coupled receptor 156 variant (Fragment).	Go Component not found
P15173	Myogenin (Myogenic factor 4) (Myf-4).	Go Component not found
Q99798	[Aconitate hydratase, mitochondrial precursor (EC 4.2.1.3) (Citrate, hydro-lyase) (Aconitase).]	Mitochondrial
Q9HCC0	[Methylcrotonoyl-CoA carboxylase beta chain, mitochondrial precursor, (EC 6.4.1.4) (3-methylcrotonyl-CoA carboxylase 2) (MCCase subunit, beta) (3-methylcrotonyl-CoA:carbon dioxide ligase subunit beta) (3-, methylcrotonyl-CoA carboxylase non-biotin-containing subunit).]	Mitochondrial
Q9UDR5	[Alpha-aminoadipic semialdehyde synthase, mitochondrial precursor, (LKR/SDH) [Includes: Lysine ketoglutarate reductase (EC 1.5.1.8) (LOR), (LKR); Saccharopine dehydrogenase (EC 1.5.1.9) (SDH)].]	Mitochondrial
Q9NY61	[Protein AATF (Apoptosis-antagonizing transcription factor) (Rb-binding, protein Che-1).]	Nuclear
Q9HCY0	Putative transcription factor-like nuclear regulator.	Nuclear
Q9H0E7	[Ubiquitin carboxyl-terminal hydrolase 44 (EC 3.1.2.15) (Ubiquitin, thioesterase 44) (Ubiquitin-specific-processing protease 44), (Deubiquitinating enzyme 44).]	Nuclear
Q7Z3Y9	Keratin-26 (Type I inner root sheath-specific keratin-K25irs2).	Other Filament or Tubule
Q86VF7	Nebulin-related-anchoring protein (N-RAP).	Other Go Component
P20908	Collagen alpha-1(V) chain precursor.	Other Go Component
Q6ZP25	CDNA FLJ26676 fis, clone MPG03726.	Other Go Component
Q6ZP91	CDNA FLJ26213 fis, clone ADG07906.	Other Go Component
Q8N845	CDNA FLJ40023 fis, clone STOMA2007680.	Other Go Component
Q6NVV1	Putative uncharacterized protein.	Other Go Component
Q6ZT89	CDNA FLJ44862 fis, clone BRALZ2010842.	Other Membrane
Q9BZJ6	Probable G-protein coupled receptor 63 (PSP24-beta) (PSP24-2).	Other Membrane
Q93070	[Ecto-ADP-ribosyltransferase 4 precursor (EC 2.4.2.31) (NAD(P)(+)--, arginine ADP-ribosyltransferase 4) (Mono(ADP-ribosyl)transferase 4), (Dombrock blood group carrier molecule) (CD297 antigen).]	Other Membrane
Q9UPU3	VPS10 domain-containing receptor SorCS3 precursor.	Other Membrane
O14817	[Tetraspanin-4 (Tspan-4) (Transmembrane 4 superfamily member 7) (Novel, antigen 2) (NAG-2).]	Plasma Membrane
Q14517	[Cadherin-related tumor suppressor homolog precursor (Protein fat, homolog).]	Plasma Membrane
Q8N126	[Cell adhesion molecule 3 precursor (Immunoglobulin superfamily member, 4B) (Nectin-like protein 1) (TSLC1-like protein 1) (Synaptic cell, adhesion molecule 3) (Brain immunoglobulin receptor).]	Plasma Membrane
O75899	[Gamma-aminobutyric acid type B receptor subunit 2 precursor (GABA-B, receptor 2) (GABA-B-R2) (Gb2) (GABABR2) (G-protein coupled receptor, 51) (HG20).]	Plasma Membrane

O15169	Axin-1 (Axis inhibition protein 1) (hAxin).	Protein Complex
Q14714	[Sarcospan (K-ras oncogene-associated protein) (Kirsten-ras-associated, protein).]	Protein Complex
Q14493	[Histone RNA hairpin-binding protein (Histone stem-loop-binding, protein).]	Protein Complex
P02787	[Serotransferrin precursor (Transferrin) (Siderophilin) (Beta-1-metal-, binding globulin).]	Vesicle

S3 of Data 3, visit 1		
**Uniprot Number**	**Protein Name**	**GO Component CATEGORY**
Q14565	Meiotic recombination protein DMC1/LIM15 homolog.	Chromosomal
Q9NQS7	Inner centromere protein.	Chromosomal
O43602	[Neuronal migration protein doublecortin (Lissencephalin-X) (Lis-X), (Doublin).]	Cytoskeletal
Q14118	[Dystroglycan precursor (Dystrophin-associated glycoprotein 1), [Contains: Alpha-dystroglycan (Alpha-DG); Beta-dystroglycan (Beta-, DG)].]	Cytoskeletal
O95425	Supervillin (Archvillin) (p205/p250).	Cytoskeletal
Q15813	Tubulin-specific chaperone E (Tubulin-folding cofactor E).	Cytoskeletal
P06396	[Gelsolin precursor (Actin-depolymerizing factor) (ADF) (Brevin), (AGEL).]	Cytoskeletal
Q07283	Trichohyalin.	Cytoskeletal
Q5HCH8	Myosin-VB (Myosin-7B) (Fragment).	Cytoskeletal
Q9C0H6	Kelch-like protein 4.	Cytoskeletal
Q9Y2M5	[Kelch-like protein 20 (Kelch-like ECT2-interacting protein) (Kelch-, like protein X).]	Cytoskeletal
Q9UDT6	[CAP-Gly domain-containing linker protein 2 (Cytoplasmic linker protein, 2) (Cytoplasmic linker protein 115) (CLIP-115) (Williams-Beuren, syndrome chromosomal region 4 protein).]	Cytoskeletal
Q9NYZ3	G2 and S phase-expressed protein 1 (B99 homolog).	Cytoskeletal
P02730	[Band 3 anion transport protein (Anion exchange protein 1) (AE 1), (Solute carrier family 4 member 1) (CD233 antigen).]	Cytoskeletal
Q684P5	[GTPase activating Rap/RanGAP domain-like protein 4 (Rap1 GTPase-, activating protein 2b).]	Cytosolic
Q8WWN8	[Centaurin-delta 3 (Cnt-d3) (Arf-GAP, Rho-GAP, ankyrin repeat and, pleckstrin homology domain-containing protein 3).]	Cytosolic
Q9Y408	Putative uncharacterized protein DKFZp566D133 (Fragment).	Cytosolic
Q14164	[Inhibitor of nuclear factor kappa-B kinase subunit epsilon, (EC 2.7.11.10) (I kappa-B kinase epsilon) (IkBKE) (IKK-epsilon) (IKK-, E) (Inducible I kappa-B kinase) (IKK-i).]	Cytosolic
Q92876	[Kallikrein-6 precursor (EC 3.4.21.-) (Protease M) (Neurosin) (Zyme), (SP59) (Serine protease 9) (Serine protease 18).]	Cytosolic
Q9BQ85	[Zinc finger protein 655 (Zinc finger protein 655, isoform CRA_b) (Vav-, 1 interacting Kruppel-like protein) (Putative uncharacterized protein, VIK).]	Cytosolic
P00966	[Argininosuccinate synthase (EC 6.3.4.5) (Citrulline--aspartate, ligase).]	Cytosolic
Q86US8	[Telomerase-binding protein EST1A (EC 3.1.-.-) (Ever shorter telomeres, 1A) (Telomerase subunit EST1A) (EST1-like protein A) (Smg-6 homolog), (hSmg5/7a).]	Cytosolic
Q6IE81	Protein Jade-1 (PHD finger protein 17).	Cytosolic
Q9Y314	Nitric oxide synthase-interacting protein (eNOS-interacting protein).	Cytosolic
P49454	[Centromere protein F (Kinetochore protein CENP-F) (Mitosin) (AH, antigen).]	Cytosolic
P37198	Nuclear pore glycoprotein p62 (62 kDa nucleoporin).	Cytosolic
P54136	[Arginyl-tRNA synthetase, cytoplasmic (EC 6.1.1.19) (Arginine--tRNA, ligase) (ArgRS).]	Cytosolic
O14787	Transportin-2 (Karyopherin beta-2b).	Cytosolic
Q8NAJ6	[CDNA FLJ35251 fis, clone PROST2003635, weakly similar to, MULTIFUNCTIONAL AMINOACYL-TRNA SYNTHETASE.]	Cytosolic
Q9C0D4	KIAA1729 protein (Fragment).	Cytosolic

Q12830	[Nucleosome-remodeling factor subunit BPTF (Bromodomain and PHD finger-, containing transcription factor) (Fetal Alzheimer antigen) (Fetal Alz-, 50 clone 1 protein).]	Cytosolic
P57075	[Suppressor of T-cell receptor signaling 2 (STS-2) (Cbl-interacting, protein 4) (CLIP4) (T-cell ubiquitin ligand) (TULA).]	Cytosolic
O75312	Zinc finger protein ZPR1 (Zinc finger protein 259).	Cytosolic
P68402	[Platelet-activating factor acetylhydrolase IB subunit beta, (EC 3.1.1.47) (PAF acetylhydrolase 30 kDa subunit) (PAF-AH 30 kDa, subunit) (PAF-AH subunit beta) (PAFAH subunit beta).]	Cytosolic
Q96A65	Exocyst complex component 4 (Exocyst complex component Sec8).	Cytosolic
Q12799	T-complex protein 10A homolog.	Cytosolic
Q8N632	Sphingosine kinase 1 (Sphingosine kinase 1, isoform CRA_b).	Cytosolic
P05771	Protein kinase C beta type (EC 2.7.11.13) (PKC-beta) (PKC-B).	Cytosolic
Q13217	[DnaJ homolog subfamily C member 3 (Interferon-induced, double-stranded, RNA-activated protein kinase inhibitor) (Protein kinase inhibitor p58), (Protein kinase inhibitor of 58 kDa).]	Cytosolic
Q9NQ66	[1-phosphatidylinositol-4,5-bisphosphate phosphodiesterase beta-1, (EC 3.1.4.11) (Phosphoinositide phospholipase C) (Phospholipase C-, beta-1) (PLC-beta-1) (PLC-I) (PLC-154).]	Cytosolic
P35228	[Nitric oxide synthase, inducible (EC 1.14.13.39) (NOS type II), (Inducible NO synthase) (Inducible NOS) (iNOS) (Hepatocyte NOS) (HEP-, NOS).]	Cytosolic
P08294	[Extracellular superoxide dismutase [Cu-Zn] precursor (EC 1.15.1.1), (EC-SOD).]	Cytosolic
Q9BYN0	Sulfiredoxin-1 (EC 1.8.98.2).	Cytosolic
P11413	Glucose-6-phosphate 1-dehydrogenase (EC 1.1.1.49) (G6PD).	Cytosolic
P02649	Apolipoprotein E precursor (Apo-E).	Cytosolic
P29074	[Tyrosine-protein phosphatase non-receptor type 4 (EC 3.1.3.48), (Protein-tyrosine phosphatase MEG1) (PTPase-MEG1) (MEG).]	Cytosolic
Q9UNH7	Sorting nexin-6 (TRAF4-associated factor 2).	Cytosolic
Q15652	[Probable JmjC domain-containing histone demethylation protein 2C, (EC 1.14.11.-) (Jumonji domain-containing protein 1C) (Thyroid, receptor-interacting protein 8) (TRIP-8).]	Cytosolic
Q9UN86	[Ras GTPase-activating protein-binding protein 2 (G3BP-2) (GAP SH3, domain-binding protein 2).]	Cytosolic
P08708	40S ribosomal protein S17.	Cytosolic
Q9BUP3	[Oxidoreductase HTATIP2 (EC 1.1.1.-) (HIV-1 TAT-interactive protein 2), (30 kDa HIV-1 TAT-interacting protein).]	Cytosolic
P13667	[Protein disulfide-isomerase A4 precursor (EC 5.3.4.1) (Protein ERp-72), (ERp72).]	ER
P56557	Transmembrane protein 50B (HCV p7-trans-regulated protein 3).	ER
P20774	Mimecan precursor (Osteoglycin) (Osteoinductive factor) (OIF).	ER
Q99442	Translocation protein SEC62 (Translocation protein 1) (TP-1) (hTP-1).	ER
P02647	[Apolipoprotein A-I precursor (Apo-AI) (ApoA-I) [Contains:, Apolipoprotein A-I(1-242)].]	Extracellular
P04003	[C4b-binding protein alpha chain precursor (C4bp) (Proline-rich, protein) (PRP).]	Extracellular
P01597	Ig kappa chain V-I region DEE.	Extracellular
P01620	Ig kappa chain V-III region SIE.	Extracellular
P80748	Ig lambda chain V-III region LOI.	Extracellular
Q99435	[Protein kinase C-binding protein NELL2 precursor (NEL-like protein 2), (Nel-related protein 2).]	Extracellular
P01009	[Alpha-1-antitrypsin precursor (Alpha-1 protease inhibitor) (Alpha-1-, antiproteinase).]	Extracellular
P01042	[Kininogen-1 precursor (Alpha-2-thiol proteinase inhibitor) [Contains:, Kininogen-1 heavy chain; Bradykinin (Kallidin I); Lysyl-bradykinin, (Kallidin II); Kininogen-1 light chain; Low molecular weight growth-, promoting factor].]	Extracellular
P01606	Ig kappa chain V-I region OU.	Extracellular
P02774	[Vitamin D-binding protein precursor (DBP) (Group-specific component), (Gc-globulin) (VDB).]	Extracellular

P02763	Alpha-1-acid glycoprotein 1 precursor (AGP 1) (Orosomucoid-1) (OMD 1).	Extracellular
P02790	Hemopexin precursor (Beta-1B-glycoprotein).	Extracellular
P01766	Ig heavy chain V-III region BRO.	Extracellular
P01593	Ig kappa chain V-I region AG.	Extracellular
P10451	[Osteopontin precursor (Bone sialoprotein 1) (Secreted phosphoprotein, 1) (SPP-1) (Urinary stone protein) (Nephropontin) (Uropontin).]	Extracellular
P04004	[Vitronectin precursor (Serum-spreading factor) (S-protein) (V75), [Contains: Vitronectin V65 subunit; Vitronectin V10 subunit;, Somatomedin-B].]	Extracellular
P19652	Alpha-1-acid glycoprotein 2 precursor (AGP 2) (Orosomucoid-2) (OMD 2).	Extracellular
P02750	Leucine-rich alpha-2-glycoprotein precursor (LRG).	Extracellular
P02766	Transthyretin precursor (Prealbumin) (TBPA) (TTR) (ATTR).	Extracellular
P08603	Complement factor H precursor (H factor 1).	Extracellular
P04196	[Histidine-rich glycoprotein precursor (Histidine-proline-rich, glycoprotein) (HPRG).]	Extracellular
P04217	Alpha-1B-glycoprotein precursor (Alpha-1-B glycoprotein).	Extracellular
P43652	Afamin precursor (Alpha-albumin) (Alpha-Alb).	Extracellular
P02749	[Beta-2-glycoprotein 1 precursor (Beta-2-glycoprotein I), (Apolipoprotein H) (Apo-H) (B2GPI) (Beta(2)GPI) (Activated protein C-, binding protein) (APC inhibitor) (Anticardiolipin cofactor).]	Extracellular
P02747	Complement C1q subcomponent subunit C precursor.	Extracellular
P10909	[Clusterin precursor (Complement-associated protein SP-40,40), (Complement cytolysis inhibitor) (CLI) (NA1/NA2) (Apolipoprotein J), (Apo-J) (Testosterone-repressed prostate message 2) (TRPM-2), [Contains: Clusterin beta chain (ApoJalpha) (Complement cytolysis, inhibitor a chain); Clusterin alpha chain (ApoJbeta) (Complement, cytolysis inhibitor b chain)].]	Extracellular
Q9UBP4	Dickkopf-related protein 3 precursor (Dkk-3) (Dickkopf-3) (hDkk-3).	Extracellular
O00587	[Beta-1,3-N-acetylglucosaminyltransferase manic fringe (EC 2.4.1.222), (O-fucosylpeptide 3-beta-N-acetylglucosaminyltransferase).]	Extracellular
P04279	[Semenogelin-1 precursor (Semenogelin I) (SGI) [Contains: Alpha-, inhibin-92; Alpha-inhibin-31; Seminal basic protein].]	Extracellular
P02765	[Alpha-2-HS-glycoprotein precursor (Fetuin-A) (Alpha-2-Z-globulin) (Ba-, alpha-2-glycoprotein) [Contains: Alpha-2-HS-glycoprotein chain A;, Alpha-2-HS-glycoprotein chain B].]	Extracellular
P41222	[Prostaglandin-H2 D-isomerase precursor (EC 5.3.99.2) (Lipocalin-type, prostaglandin-D synthase) (Glutathione-independent PGD synthetase), (Prostaglandin-D2 synthase) (PGD2 synthase) (PGDS2) (PGDS) (Beta-trace, protein) (Cerebrin-28).]	Extracellular
O00584	Ribonuclease T2 precursor (EC 3.1.27.-) (Ribonuclease 6).	Extracellular
P00450	Ceruloplasmin precursor (EC 1.16.3.1) (Ferroxidase).	Extracellular
P07711	[Cathepsin L precursor (EC 3.4.22.15) (Major excreted protein) (MEP), [Contains: Cathepsin L heavy chain; Cathepsin L light chain].]	Extracellular
Q53F31	[Vitamin D-binding protein variant (Group-specific component) (Vitamin, D binding protein) (Fragment).]	Extracellular
P07602	[Proactivator polypeptide precursor [Contains: Saposin-A (Protein A);, Saposin-B-Val; Saposin-B (Sphingolipid activator protein 1) (SAP-1), (Cerebroside sulfate activator) (CSAct) (Dispersin) (Sulfatide/GM1, activator); Saposin-C (Co-beta-glucosidase) (A1 activator), (Glucosylceramidase activator) (Sphingolipid activator protein 2), (SAP-2); Saposin-D (Protein C) (Component C)].]	Extracellular
P13521	[Secretogranin-2 precursor (Secretogranin II) (SgII) (Chromogranin-C), [Contains: Secretoneurin (SN)].]	Extracellular
P02768	Serum albumin precursor.	Extracellular
P01011	[Alpha-1-antichymotrypsin precursor (ACT) (Cell growth-inhibiting gene, 24/25 protein) [Contains: Alpha-1-antichymotrypsin His-Pro-less].]	Extracellular
P06727	Apolipoprotein A-IV precursor (Apo-AIV) (ApoA-IV).	Extracellular
Q92520	Protein FAM3C precursor (Protein GS3786).	Extracellular

P07477	[Trypsin-1 precursor (EC 3.4.21.4) (Trypsin I) (Cationic trypsinogen), (Serine protease 1).]	Extracellular
Q8IUK7	ALB protein.	Extracellular
P36955	[Pigment epithelium-derived factor precursor (PEDF) (Serpin-F1) (EPC-, 1).]	Extracellular
Q9UHG2	[ProSAAS precursor (pro-SAAS) (Proprotein convertase subtilisin/kexin, type 1 inhibitor) (Proprotein convertase 1 inhibitor) [Contains: KEP;, Big SAAS (b-SAAS); Little SAAS (l-SAAS) (N-proSAAS); Big PEN-LEN (b-, PEN-LEN) (SAAS CT(1-49)); PEN; Little LEN (l-LEN); Big LEN (b-LEN), (SAAS CT(25-40))].]	Extracellular
P01023	Alpha-2-macroglobulin precursor (Alpha-2-M).	Extracellular
P05155	[Plasma protease C1 inhibitor precursor (C1 Inh) (C1Inh) (C1 esterase, inhibitor) (C1-inhibiting factor).]	Extracellular
Q6U2F8	C4A91 (C4A) (Fragment).	Extracellular
P01019	[Angiotensinogen precursor (Serpin A8) [Contains: Angiotensin-1, (Angiotensin I) (Ang I); Angiotensin-2 (Angiotensin II) (Ang II);, Angiotensin-3 (Angiotensin III) (Ang III) (Des-Asp[1]-angiotensin, II)].]	Extracellular
P23142	Fibulin-1 precursor.	Extracellular
P05090	Apolipoprotein D precursor (Apo-D) (ApoD).	Extracellular
P02652	[Apolipoprotein A-II precursor (Apo-AII) (ApoA-II) [Contains:, Apolipoprotein A-II(1-76)].]	Extracellular
Q9Y272	[Dexamethasone-induced Ras-related protein 1 precursor (Activator of G-, protein signaling 1).]	Go Component not found
O75154	[Rab11 family-interacting protein 3 (Rab11-FIP3) (EF hands-containing, Rab-interacting protein) (Eferin).]	Go Component not found
P30154	[Serine/threonine-protein phosphatase 2A 65 kDa regulatory subunit A, beta isoform (PP2A, subunit A, PR65-beta isoform) (PP2A, subunit A,, R1-beta isoform).]	Go Component not found
Q16671	[Anti-Muellerian hormone type-2 receptor precursor (EC 2.7.11.30), (Anti-Muellerian hormone type II receptor) (AMH type II receptor) (MIS, type II receptor) (MISRII) (MRII).]	Go Component not found
O96020	G1/S-specific cyclin-E2.	Go Component not found
P00751	[Complement factor B precursor (EC 3.4.21.47) (C3/C5 convertase), (Properdin factor B) (Glycine-rich beta glycoprotein) (GBG) (PBF2), [Contains: Complement factor B Ba fragment; Complement factor B Bb, fragment].]	Go Component not found
Q92828	Coronin-2A (WD repeat-containing protein 2) (IR10).	Go Component not found
Q01804	OTU domain-containing protein 4 (HIV-1-induced protein HIN-1).	Go Component not found
Q16270	[Insulin-like growth factor-binding protein 7 precursor (IGFBP-7) (IBP-, 7) (IGF-binding protein 7) (MAC25 protein) (Prostacyclin-stimulating, factor) (PGI2-stimulating factor) (IGFBP-rP1).]	Go Component not found
O14974	[Protein phosphatase 1 regulatory subunit 12A (Myosin phosphatase-, targeting subunit 1) (Myosin phosphatase target subunit 1) (Protein, phosphatase myosin-binding subunit).]	Go Component not found
O15047	[Histone-lysine N-methyltransferase, H3 lysine-4 specific SET1, (EC 2.1.1.43) (Set1/Ash2 histone methyltransferase complex subunit, SET1) (SET domain-containing protein 1A).]	Go Component not found
Q58HN0	Programmed cell death 2 isoform 1.	Go Component not found
Q8NF20	FLJ00382 protein (Fragment).	Go Component not found
Q9NSN6	Putative uncharacterized protein DKFZp761O0113.	Go Component not found
Q8NE71	[ATP-binding cassette sub-family F member 1 (ATP-binding cassette 50), (TNF-alpha-stimulated ABC protein).]	Go Component not found
Q9NZN5	[Rho guanine nucleotide exchange factor 12 (Leukemia-associated, RhoGEF).]	Go Component not found
P05937	[Calbindin (Vitamin D-dependent calcium-binding protein, avian-type), (Calbindin D28) (D-28K).]	Go Component not found
P46013	Antigen KI-67.	Go Component not found
P01842	Ig lambda chain C regions.	Go Component not found
Q8WTW4	[Tumor suppressor candidate 4 (NPR2-like) (Gene 21 protein) (G21, protein).]	Go Component not found
Q8N720	Zinc finger protein 655 (Vav-interacting Krueppel-like protein).	Go Component not found

Q8N1V2	[WD repeat-containing protein 16 (WD40-repeat protein up-regulated in, HCC).]	Go Component not found
Q9Y4W2	LAS1-like protein.	Go Component not found
Q8NB16	Mixed lineage kinase domain-like protein.	Go Component not found
Q8TDL7	Spermatogenesis associated factor.	Go Component not found
Q96BE9	CDK4 protein (Cyclin-dependent kinase 4, isoform CRA_c).	Go Component not found
P61769	[Beta-2-microglobulin precursor [Contains: Beta-2-microglobulin form pI, 5.3].]	Go Component not found
O00512	B-cell lymphoma 9 protein (Bcl-9) (Legless homolog).	Go Component not found
Q13045	Protein flightless-1 homolog.	Go Component not found
P35527	[Keratin, type I cytoskeletal 9 (Cytokeratin-9) (CK-9) (Keratin-9), (K9).]	Go Component not found
P42679	[Megakaryocyte-associated tyrosine-protein kinase (EC 2.7.10.2), (Tyrosine-protein kinase CTK) (Protein kinase HYL) (Hematopoietic, consensus tyrosine-lacking kinase).]	Go Component not found
Q5JUW7	[Discs, large homolog 3 (Neuroendocrine-dlg, Drosophila) (Discs, large, homolog 3 (Neuroendocrine-dlg, Drosophila), isoform CRA_d).]	Go Component not found
Q96AJ1	Clusterin-associated protein 1.	Go Component not found
Q6NUP7	KIAA1622 (KIAA1622, isoform CRA_a).	Go Component not found
Q8N774	CDNA FLJ25966 fis, clone TST05207.	Go Component not found
Q9H0J4	Glutamine-rich protein 2.	Go Component not found
Q7Z2E3	[Aprataxin (EC 3.-.-.-) (Forkhead-associated domain histidine triad-, like protein) (FHA-HIT).]	Go Component not found
P38935	[DNA-binding protein SMUBP-2 (EC 3.6.1.-) (ATP-dependent helicase, IGHMBP2) (Immunoglobulin mu-binding protein 2) (SMUBP-2) (Glial factor, 1) (GF-1).]	Go Component not found
Q5VZL5	Zinc finger MYM-type protein 4 (Zinc finger protein 262).	Go Component not found
O00172	Line-1 reverse transcriptase (Fragment).	Go Component not found
Q9H040	[Putative uncharacterized protein DKFZp547N043 (Chromosome 1 open, reading frame 124) (CDNA FLJ14707 fis, clone NT2RP3000599).]	Go Component not found
P51816	[AF4/FMR2 family member 2 (Fragile X mental retardation 2 protein), (Protein FMR-2) (FMR2P) (Protein Ox19) (Fragile X E mental retardation, syndrome protein).]	Go Component not found
Q96NS5	Ankyrin repeat and SOCS box protein 16 (ASB-16).	Go Component not found
P59510	[ADAMTS-20 precursor (EC 3.4.24.-) (A disintegrin and metalloproteinase, with thrombospondin motifs 20) (ADAM-TS 20) (ADAM-TS20).]	Go Component not found
P19022	[Cadherin-2 precursor (Neural cadherin) (N-cadherin) (CD325 antigen), (CDw325).]	Go Component not found
Q9P209	Centrosomal protein of 72 kDa (Cep72 protein).	Go Component not found
Q8WUY3	Prune homolog 2.	Go Component not found
P10645	[Chromogranin-A precursor (CgA) (Pituitary secretory protein I) (SP-I), [Contains: Vasostatin-1 (Vasostatin I); Vasostatin-2 (Vasostatin II);, EA-92; ES-43; Pancreastatin; SS-18; WA-8; WE-14; LF-19; AL-11; GV-19;, GR-44; ER-37].]	Go Component not found
Q96M20	Uncharacterized protein C20orf152.	Go Component not found
Q8TBR7	Protein FAM57A (CT120 protein).	Go Component not found
Q9P121	Neurotrimin precursor (hNT).	Go Component not found
Q8NDF8	[PAP-associated domain-containing protein 5 (EC 2.7.7.-), (Topoisomerase-related function protein 4-2) (TRF4-2).]	Go Component not found
P16499	[Rod cGMP-specific 3',5'-cyclic phosphodiesterase subunit alpha, (EC 3.1.4.35) (GMP-PDE alpha) (PDE V-B1).]	Go Component not found
P17257	UPF0484 protein C1orf90 homolog (Protein SEC).	Go Component not found

O15389	[Sialic acid-binding Ig-like lectin 5 precursor (Siglec-5) (Obesity-, binding protein 2) (OB-binding protein 2) (OB-BP2) (CD33 antigen-like, 2) (CD170 antigen).]	Go Component not found
Q96P15	Serpin B11.	Go Component not found
P59817	[Suppressor of hairy wing homolog 1 (3'OY11.1) (Zinc finger protein, 280).]	Go Component not found
O95932	[Protein-glutamine gamma-glutamyltransferase 6 (EC 2.3.2.13), (Transglutaminase-3-like) (TGase-3-like) (Transglutaminase Y).]	Go Component not found
Q96QT4	[Transient receptor potential cation channel subfamily M member 7, (EC 2.7.11.1) (Long transient receptor potential channel 7) (LTrpC7), (Channel-kinase 1).]	Go Component not found
Q9ULK2	Ataxin-7-like protein 1.	Go Component not found
Q9P0L1	[Zinc finger protein 167 (Zinc finger protein 64) (Zinc finger protein, 448) (ZFP) (Zinc finger protein with KRAB and SCAN domains 7).]	Go Component not found
Q9NYT6	Zinc finger protein 226.	Go Component not found
Q15786	Testis calpastatin.	Go Component not found
Q5EFE6	Anti-RhD monoclonal T125 kappa light chain precursor.	Go Component not found
Q5HYI6	Putative uncharacterized protein DKFZp313O2236 (Fragment).	Go Component not found
Q9ULE6	Paladin.	Go Component not found
Q5JWS2	ERGIC and golgi 3 (Fragment).	Go Component not found
Q9Y2I6	Ninein-like protein.	Go Component not found
Q4LE39	[AT-rich interactive domain-containing protein 4B (ARID domain-, containing protein 4B) (Histone deacetylase complex subunit SAP180), (180 kDa Sin3-associated polypeptide) (Sin3-associated polypeptide, p180) (Retinoblastoma-binding protein 1-like 1) (Breast cancer-, associated antigen BRCAA1).]	Go Component not found
Q68CQ1	Uncharacterized protein C1orf175.	Go Component not found
Q9H3W5	[Leucine-rich repeat neuronal protein 3 precursor (Neuronal leucine-, rich repeat protein 3) (NLRR-3).]	Go Component not found
Q7L3V2	Uncharacterized protein C22orf29.	Go Component not found
Q6N030	Putative uncharacterized protein DKFZp686I15212.	Go Component not found
Q6P1N9	[TatD DNAse domain-containing protein 1 (Hepatocarcinoma high, expression protein).]	Go Component not found
Q4G0Z9	Uncharacterized protein C8orf45.	Go Component not found
Q6PIU1	[Potassium voltage-gated channel subfamily V member 1 (Voltage-gated, potassium channel subunit Kv8.1) (Neuronal potassium channel alpha, subunit HNKA).]	Go Component not found
Q6UTX4	Putative uncharacterized protein.	Go Component not found
Q6ZNH7	[CDNA FLJ16074 fis, clone LIVER2000247, weakly similar to RENAL, SODIUM/DICARBOXYLATE COTRANSPORTER.]	Go Component not found
Q6ZNS0	CDNA FLJ27256 fis, clone SYN09689.	Go Component not found
Q6ZTQ6	CDNA FLJ44356 fis, clone TRACH3006889.	Go Component not found
Q6ZUT9	CDNA FLJ43333 fis, clone NT2RI3006376.	Go Component not found
Q6ZVL3	[CDNA FLJ42424 fis, clone BLADE2004089, moderately similar to Mus, musculus PDZ domain actin binding protein Shroom mRNA. (Fragment).]	Go Component not found
Q14C87	[Transmembrane protein 132D precursor (Mature oligodendrocytes, transmembrane protein) (Mature OL transmembrane protein).]	Go Component not found
Q7Z766	LOC492303 protein.	Go Component not found
Q86YA3	Uncharacterized protein C4orf21.	Go Component not found
Q8IUW3	[Spermatogenesis-associated protein 2-like protein (SPATA2-like, protein).]	Go Component not found
Q9Y4B5	Uncharacterized protein KIAA0802.	Go Component not found
Q8IYM2	Schlafen family member 12.	Go Component not found
Q8N0V7	CDNA FLJ40969 fis, clone UTERU2012688 (CDNA FLJ40984 fis).	Go Component not found

Q8N4V6	TMEM181 protein (Fragment).	Go Component not found
Q5T124	[UBX domain-containing protein 5 (Socius) (Colorectal tumor-associated, antigen COA-1).]	Go Component not found
Q8NAS4	[CDNA FLJ34863 fis, clone NT2NE2014013, moderately similar to, SEMAPHORIN 3B.]	Go Component not found
Q8TBR0	PDE4DIP protein.	Go Component not found
Q969F2	Protein naked cuticle homolog 2 (hNkd2) (Naked-2).	Go Component not found
Q96ET8	Protein FAM18B2.	Go Component not found
Q96JD0	Amyloid lambda 6 light chain variable region SAR (Fragment).	Go Component not found
Q96LR2	Uncharacterized protein C1orf190.	Go Component not found
Q9BRD0	BUD13 homolog.	Go Component not found
Q9BSH9	MGC31957 protein.	Go Component not found
Q96JA3	[Pleckstrin homology domain-containing family A member 8, (Phosphoinositol 4-phosphate adapter protein 2) (Phosphatidylinositol-, four-phosphate adapter protein 2) (hFAPP2) (Serologically defined, breast cancer antigen NY-BR-86).]	Go Component not found
Q9H024	Putative uncharacterized protein DKFZp547J036 (ELAVL3 protein).	Go Component not found
Q9H3S5	[GPI mannosyltransferase 1 (EC 2.4.1.-) (GPI mannosyltransferase I), (GPI-MT-I) (Phosphatidylinositol-glycan biosynthesis class M protein), (PIG-M).]	Go Component not found
Q9H799	CDNA: FLJ21126 fis, clone CAS06183. (Fragment).	Go Component not found
Q8TC05	Nuclear protein MDM1.	Go Component not found
Q9ULS2	KIAA1148 protein (Fragment).	Go Component not found
Q2M389	Uncharacterized protein KIAA1033.	Go Component not found
Q9Y6L7	Tolloid-like protein 2 precursor (EC 3.4.24.-).	Go Component not found
Q30134	[HLA class II histocompatibility antigen, DRB1-8 beta chain precursor, (MHC class I antigen DRB1*8) (DR-8) (DR8) (DRw8).]	Go Component not found
Q8WWM7	[Ataxin-2-like protein (Ataxin-2 domain protein) (Ataxin-2-related, protein).]	Go Component not found
Q96P47	[Centaurin-gamma 3 (ARF-GAP with GTP-binding protein-like, ankyrin, repeat and pleckstrin homology domains 3) (AGAP-3) (MR1-interacting, protein) (MRIP-1) (CRAM-associated GTPase) (CRAG).]	Go Component not found
Q5VXU9	Uncharacterized protein C9orf84.	Go Component not found
Q7Z5M8	Abhydrolase domain-containing protein 12B.	Go Component not found
P0C0L4	[Complement C4-A precursor (Acidic complement C4) [Contains: Complement, C4 beta chain; Complement C4-A alpha chain; C4a anaphylatoxin; C4b-A;, C4d-A; Complement C4 gamma chain].]	Go Component not found
Q96KN2	[Beta-Ala-His dipeptidase precursor (EC 3.4.13.20) (Carnosine, dipeptidase 1) (CNDP dipeptidase 1) (Serum carnosinase) (Glutamate, carboxypeptidase-like protein 2).]	Go Component not found
Q9BZ29	[Dedicator of cytokinesis protein 9 (Cdc42 guanine nucleotide exchange, factor zizimin-1).]	Go Component not found
P18065	[Insulin-like growth factor-binding protein 2 precursor (IGFBP-2) (IBP-, 2) (IGF-binding protein 2).]	Go Component not found
Q9H3R0	[JmjC domain-containing histone demethylation protein 3C (EC 1.14.11.-), (Jumonji domain-containing protein 2C) (Gene amplified in squamous, cell carcinoma 1 protein) (GASC-1 protein).]	Go Component not found
Q96PQ7	Kelch-like protein 5.	Go Component not found
Q7Z553	[MAM domain-containing glycosylphosphatidylinositol anchor protein 2, precursor (MAM domain-containing protein 1).]	Go Component not found
Q08431	[Lactadherin precursor (Milk fat globule-EGF factor 8) (MFG-E8) (HMFG), (Breast epithelial antigen BA46) (MFGM) [Contains: Lactadherin short, form; Medin].]	Go Component not found
Q86W25	[NACHT, LRR and PYD domains-containing protein 13 (Nucleotide-binding, oligomerization domain protein 14).]	Go Component not found
Q9UGF6	Olfactory receptor 5V1 (Olfactory receptor OR6-26) (Hs6M1-21).	Go Component not found
Q8N3C7	CAP-Gly domain-containing linker protein 4 (Restin-like protein 2).	Go Component not found

Q9BYH1	Seizure 6-like protein precursor.	Go Component not found
Q14515	[SPARC-like protein 1 precursor (High endothelial venule protein), (Hevin) (MAST 9).]	Go Component not found
Q13277	Syntaxin-3.	Go Component not found
Q96BZ9	TBC1 domain family member 20.	Go Component not found
Q9HCL3	Zinc finger protein 14 homolog (Zfp-14) (Zinc finger protein 531).	Go Component not found
O15014	Zinc finger protein 609.	Go Component not found
Q4VC05	B-cell CLL/lymphoma 7 protein family member A.	Go Component not found
Q56UN5	[SPS1/STE20-related protein kinase YSK4 (EC 2.7.11.1) (Regulated in, COPD, protein kinase).]	Go Component not found
Q5CZ79	Ankyrin repeat domain-containing protein 20B (Fragment).	Go Component not found
Q5H9L2	[Transcription elongation factor A protein-like 5 (TCEA-like protein 5), (Transcription elongation factor S-II protein-like 5).]	Go Component not found
Q86UY6	N-acetyltransferase 11 (EC 2.3.1.-).	Go Component not found
Q5JUR7	Uncharacterized protein C13orf27.	Go Component not found
Q8NEY1	[Neuron navigator 1 (Steerin-1) (Pore membrane and/or filament-, interacting-like protein 3) (Unc-53 homolog 1) (unc53H1).]	Go Component not found
Q5T2T0	Novel protein (Fragment).	Go Component not found
Q5T7N3	Ankyrin repeat domain-containing protein 38.	Go Component not found
Q5VTR2	[E3 ubiquitin-protein ligase BRE1A (EC 6.3.2.-) (BRE1-A) (hBRE1) (RING, finger protein 20).]	Go Component not found
Q5VWN6	Uncharacterized protein C10orf18.	Go Component not found
Q63HM9	[Phosphatidylinositol-specific phospholipase C X domain-containing, protein 3 (PI-PLC X domain-containing protein 3).]	Go Component not found
Q68CJ6	Uncharacterized protein C8orf80.	Go Component not found
Q68CZ6	Uncharacterized protein C4orf15.	Go Component not found
Q6ZXV5	Transmembrane and TPR repeat-containing protein 3 (Protein SMILE).	Go Component not found
Q6MZQ6	Putative uncharacterized protein DKFZp686G11190.	Go Component not found
Q6N095	Putative uncharacterized protein DKFZp686K03196.	Go Component not found
Q9NS62	[Thrombospondin type-1 domain-containing protein 1 precursor, (Transmembrane molecule with thrombospondin module).]	Go Component not found
Q6P6B1	Uncharacterized protein C8orf47.	Go Component not found
Q6SPF0	Atherin (Sterile alpha motif domain-containing protein 1).	Go Component not found
Q6UWM7	[Lactase-like protein precursor (Klotho/lactase-phlorizin hydrolase-, related protein).]	Go Component not found
Q6ZMZ3	Nesprin-3.	Go Component not found
Q6ZNI8	Uncharacterized protein C1orf84 (Fragment).	Go Component not found
Q6ZRH3	CDNA FLJ46360 fis, clone TESTI4049863.	Go Component not found
Q6ZU14	CDNA FLJ44063 fis, clone TESTI4035637.	Go Component not found
Q6ZV48	CDNA FLJ42992 fis, clone BRTHA2010608.	Go Component not found
Q5THJ4	Vacuolar protein sorting-associated protein 13D.	Go Component not found
Q7Z6G3	[EF-hand calcium-binding protein 2 (Neuronal calcium-binding protein 2), (Synaptotagmin-interacting protein 2) (Stip-2).]	Go Component not found
Q86TG9	COL12A1 protein.	Go Component not found
Q8IVL0	[Neuron navigator 3 (Steerin-3) (Pore membrane and/or filament-, interacting-like protein 1) (Unc-53 homolog 3) (unc53H3).]	Go Component not found
Q8IWB8	CCR4-NOT transcription complex, subunit 1.	Go Component not found
Q8IYR2	SET and MYND domain-containing protein 4.	Go Component not found
Q8N1K5	Uncharacterized protein C6orf190.	Go Component not found
Q8N412	[Chromosome 4 open reading frame 37 (Putative uncharacterized protein, MGC46496).]	Go Component not found

Q96MF7	[E3 SUMO-protein ligase NSE2 (EC 6.-.-.-) (Non-structural maintenance, of chromosomes element 2 homolog) (Non-SMC element 2 homolog), (hMMS21).]	Go Component not found
Q8N7Q3	Zinc finger protein 676.	Go Component not found
Q8N9X4	[CDNA FLJ36112 fis, clone TESTI2022023, weakly similar to Bos taurus, Reissner's fiber glycoprotein I mRNA.]	Go Component not found
Q8NFL6	Amphiphysin I variant CT4 (Fragment).	Go Component not found
Q8WZ27	Putative uncharacterized protein.	Go Component not found
Q96E03	Melanoma antigen family A, 2B.	Go Component not found
A6BM72	[Multiple epidermal growth factor-like domains 11 precursor (Multiple, EGF-like-domains 11).]	Go Component not found
Q96LS8	Uncharacterized protein C2orf48.	Go Component not found
Q96N23	Uncharacterized protein C12orf55 (Fragment).	Go Component not found
Q99908	BB1 protein.	Go Component not found
Q9BQ86	C14orf131 protein.	Go Component not found
Q9BRN9	[TM2 domain-containing protein 3 precursor (Beta-amyloid-binding, protein-like protein 2) (BBP-like protein 2).]	Go Component not found
Q9BSK1	Zinc finger protein 577.	Go Component not found
Q9BUR4	WD repeat-containing protein 79.	Go Component not found
Q9H609	Zinc finger protein 576.	Go Component not found
Q9H8H0	Nucleolar protein 11.	Go Component not found
Q9NRU5	X 001 protein.	Go Component not found
Q5VX71	Sushi domain-containing protein 4 precursor.	Go Component not found
Q9NX71	CDNA FLJ20402 fis, clone KAT00919.	Go Component not found
Q8TCC3	39S ribosomal protein L30, mitochondrial precursor (L30mt) (MRP-L30).	Go Component not found
Q9UL88	Myosin-reactive immunoglobulin heavy chain variable region (Fragment).	Go Component not found
Q9UNZ4	NPD002.	Go Component not found
Q9BX69	Caspase recruitment domain-containing protein 6.	Go Component not found
Q5T5N4	Uncharacterized protein C6orf118.	Go Component not found
Q96E09	Protein FAM122A.	Go Component not found
P0C0L5	[Complement C4-B precursor (Basic complement C4) [Contains: Complement, C4 beta chain; Complement C4-B alpha chain; C4a anaphylatoxin; C4b-B;, C4d-B; Complement C4 gamma chain].]	Go Component not found
O94985	Calsyntenin-1 precursor.	Go Component not found
Q13769	[THO complex subunit 5 homolog (NF2/meningioma region protein pK1.3), (Placental protein 39.2).]	Go Component not found
Q9BTC0	[Death-inducer obliterator 1 (DIO-1) (Death-associated transcription, factor 1) (DATF-1) (hDido1).]	Go Component not found
P24592	[Insulin-like growth factor-binding protein 6 precursor (IGFBP-6) (IBP-, 6) (IGF-binding protein 6).]	Go Component not found
P27352	Gastric intrinsic factor precursor (Intrinsic factor) (IF) (INF).	Go Component not found
Q8N7A1	Kelch domain-containing protein 1.	Go Component not found
Q13449	Limbic system-associated membrane protein precursor (LSAMP).	Go Component not found
Q14149	[MORC family CW-type zinc finger protein 3 (Zinc finger CW-type coiled-, coil domain protein 3).]	Go Component not found
Q8NC67	[Neuropilin and tolloid-like protein 2 precursor (Brain-specific, transmembrane protein containing 2 CUB and 1 LDL-receptor class A, domains protein 2).]	Go Component not found
P05120	[Plasminogen activator inhibitor 2 precursor (PAI-2) (Placental, plasminogen activator inhibitor) (Monocyte Arg-serpin) (Urokinase, inhibitor).]	Go Component not found
Q9Y5G9	Protocadherin gamma A4 precursor (PCDH-gamma-A4).	Go Component not found
Q15032	R3H domain-containing protein 1.	Go Component not found

Q96EP0	[RING finger protein 31 (Zinc in-between-RING-finger ubiquitin-, associated domain protein).]	Go Component not found
Q9NY91	[Low affinity sodium-glucose cotransporter (Sodium/glucose, cotransporter 3) (Na(+)/glucose cotransporter 3) (Solute carrier, family 5 member 4).]	Go Component not found
Q8WXD2	Secretogranin-3 precursor (Secretogranin III) (SgIII).	Go Component not found
O43166	[Signal-induced proliferation-associated 1-like protein 1 (High-risk, human papilloma viruses E6 oncoproteins targeted protein 1) (E6-, targeted protein 1).]	Go Component not found
O94991	[SLIT and NTRK-like protein 5 precursor (Leucine-rich repeat-containing, protein 11).]	Go Component not found
Q96BD0	[Solute carrier organic anion transporter family member 4A1 (Solute, carrier family 21 member 12) (Sodium-independent organic anion, transporter E) (Organic anion-transporting polypeptide E) (OATP-E), (Colon organic anion transporter) (Organic anion transporter, polypeptide-related protein 1) (OATP-RP1) (OATPRP1) (POAT).]	Go Component not found
Q8TDD2	Transcription factor Sp7 (Zinc finger protein osterix).	Go Component not found
O00186	[Syntaxin-binding protein 3 (Unc-18 homolog 3) (Unc-18C) (Unc-18-3), (Platelet Sec1 protein) (PSP).]	Go Component not found
Q96G75	RMD5 homolog B.	Go Component not found
Q9NWZ5	Uridine/cytidine kinase-like 1 (EC 2.7.1.48).	Go Component not found
Q8NB66	Protein unc-13 homolog C (Munc13-3).	Go Component not found
Q9UK10	Zinc finger protein 225.	Go Component not found
Q9BY12	[S phase cyclin A-associated protein in the ER (Zinc finger protein, 291).]	Go Component not found
P60852	[Zona pellucida sperm-binding protein 1 precursor (Zona pellucida, glycoprotein 1) (Zp-1) [Contains: Processed zona pellucida sperm-, binding protein 1].]	Go Component not found
O75373	Zinc finger protein (Fragment).	Go Component not found
Q5CZ94	Putative uncharacterized protein DKFZp781M0386.	Go Component not found
Q5EBL2	Zinc finger protein 628.	Go Component not found
Q5JSF6	Novel protein (Fragment).	Go Component not found
Q5JY77	G-protein coupled receptor-associated sorting protein 1 (GASP-1).	Go Component not found
Q495T6	[Membrane metallo-endopeptidase-like 1 (EC 3.4.24.11) (Membrane, metallo-endopeptidase-like 2) (Neprilysin-2) (Neprilysin II) (NL2), (NEPII) (NEP2(m)) [Contains: Membrane metallo-endopeptidase-like 1,, soluble form (Neprilysin-2 secreted) (NEP2(s))].]	Go Component not found
Q5T5A4	Uncharacterized protein C1orf194.	Go Component not found
Q8NC56	LEM domain-containing protein 2 (hLEM2).	Go Component not found
Q5VU18	[Coiled-coil domain containing 19 (Coiled-coil domain containing 19,, isoform CRA_b).]	Go Component not found
Q68CN4	Putative uncharacterized protein DKFZp686E23209.	Go Component not found
Q68D85	Putative uncharacterized protein DKFZp686I21167.	Go Component not found
Q6MZV6	Putative uncharacterized protein DKFZp686L19235.	Go Component not found
Q9P2N6	[Uncharacterized protein KIAA1310 (Serum inhibited-related protein), (Testis development protein PRTD).]	Go Component not found
Q6UWL2	Sushi domain-containing protein 1 precursor.	Go Component not found
Q6UXR4	Serpin A13 precursor.	Go Component not found
Q6ZN93	CDNA FLJ16316 fis, clone SPLEN2033490.	Go Component not found
Q6ZRZ6	CDNA FLJ45951 fis, clone PLACE7008766.	Go Component not found
Q7Z2X4	[PTB-containing, cubilin and LRP1-interacting protein (P-CLI1), (Phosphotyrosine interaction domain-containing protein 1) (Protein, NYGGF4).]	Go Component not found
Q70CQ4	[Ubiquitin carboxyl-terminal hydrolase 31 (EC 3.1.2.15) (Ubiquitin, thioesterase 31) (Ubiquitin-specific-processing protease 31), (Deubiquitinating enzyme 31).]	Go Component not found
O95622	[Adenylate cyclase type 5 (EC 4.6.1.1) (Adenylate cyclase type V) (ATP, pyrophosphate-lyase 5) (Adenylyl cyclase 5).]	Go Component not found

Q7Z6J2	[General receptor for phosphoinositides 1-associated scaffold protein, (GRP1-associated scaffold protein).]	Go Component not found
Q86X02	[Cerebellar degeneration-related protein 2-like (Paraneoplastic 62 kDa, antigen).]	Go Component not found
Q8IWG1	WD repeat-containing protein 63 (Testis development protein NYD-SP29).	Go Component not found
Q8IZ40	REST corepressor 2.	Go Component not found
Q8N2E2	[CDNA PSEC0224 fis, clone HEMBA1005703, weakly similar to UROMODULIN, (HCG1744959).]	Go Component not found
Q9C0J1	[UDP-GlcNAc:betaGal beta-1,3-N-acetylglucosaminyltransferase 4, (EC 2.4.1.-) (Beta3Gn-T4) (BGnT-4) (Beta-1,3-N-, acetylglucosaminyltransferase-4).]	Go Component not found
Q8N7T0	[CDNA FLJ40392 fis, clone TESTI2036913 (HCG1820408, isoform CRA_a), (DPY19L2P2 protein).]	Go Component not found
Q8N9J9	CDNA FLJ37013 fis, clone BRACE2010171 (Hypothetical LOC440337).	Go Component not found
Q96QD5	DEP domain-containing protein 7 (Protein TR2/D15).	Go Component not found
Q96DN5	WD repeat-containing protein 67.	Go Component not found
Q8WUN7	[Ubiquitin domain-containing protein 2 (Dendritic cell-derived, ubiquitin-like protein) (DC-UbP) (Ubiquitin-like protein SB72).]	Go Component not found
Q8WZ28	[Putative uncharacterized protein (Putative uncharacterized protein, MGC3123).]	Go Component not found
Q96BZ3	HAGHL protein (Hydroxyacylglutathione hydrolase-like, isoform CRA_h).	Go Component not found
Q96ES7	Coiled-coil domain-containing protein 101.	Go Component not found
Q96HQ4	DUSP3 protein.	Go Component not found
Q96M85	Putative uncharacterized protein FLJ32756.	Go Component not found
Q99993	WUGSC:H_2G3A.1 protein.	Go Component not found
Q9BR77	Coiled-coil domain-containing protein 77.	Go Component not found
Q9BSH5	Haloacid dehalogenase-like hydrolase domain-containing protein 3.	Go Component not found
Q9BTF7	Calcium binding and coiled-coil domain 2.	Go Component not found
Q9H814	[RNA U small nuclear RNA export adapter protein (Phosphorylated adapter, RNA export protein).]	Go Component not found
Q5TAA0	Tetratricopeptide repeat protein 22 (TPR repeat protein 22).	Go Component not found
Q9UBS9	Protein C1orf9 precursor.	Go Component not found
Q9UDW8	WUGSC:H_DJ0747G18.3 protein (VGF nerve growth factor inducible).	Go Component not found
Q9ULH9	KIAA1241 protein (Fragment).	Go Component not found
Q6MZW2	Follistatin-related protein 4 precursor (Follistatin-like 4).	Go Component not found
Q6ZRS2	[Helicase SRCAP (EC 3.6.1.-) (Snf2-related CBP activator) (Domino, homolog 2).]	Go Component not found
P05129	Protein kinase C gamma type (EC 2.7.11.13) (PKC-gamma).	Go Component not found
Q5VWK9	[Membrane associated guanylate kinase, WW and PDZ domain containing 3, (Membrane associated guanylate kinase, WW and PDZ domain containing 3,, isoform CRA_b).]	Go Component not found
Q96MA6	Putative adenylate kinase-like protein C9orf98 (EC 2.7.4.3).	Go Component not found
Q9UKA4	[A-kinase anchor protein 11 (Protein kinase A-anchoring protein 11), (PRKA11) (A kinase anchor protein 220 kDa) (AKAP 220) (hAKAP220).]	Go Component not found
P52824	[Diacylglycerol kinase theta (EC 2.7.1.107) (Diglyceride kinase theta), (DGK-theta) (DAG kinase theta).]	Go Component not found
Q9UDY2	[Tight junction protein ZO-2 (Zonula occludens protein 2) (Zona, occludens protein 2) (Tight junction protein 2).]	Go Component not found
Q5JZ02	TP53 regulating kinase.	Go Component not found
Q13315	[Serine-protein kinase ATM (EC 2.7.11.1) (Ataxia telangiectasia, mutated) (A-T, mutated).]	Go Component not found
P35558	[Phosphoenolpyruvate carboxykinase, cytosolic [GTP] (EC 4.1.1.32), (Phosphoenolpyruvate carboxylase) (PEPCK-C).]	Go Component not found
P12931	[Proto-oncogene tyrosine-protein kinase Src (EC 2.7.10.2) (p60-Src) (c-, Src) (pp60c-src).]	Go Component not found
Q53HR2	Acyl-Coenzyme A dehydrogenase, very long chain variant (Fragment).	Go Component not found

Q68DD2	[Cytosolic phospholipase A2 zeta (EC 3.1.1.4) (cPLA2-zeta), (Phospholipase A2 group IVF).]	Go Component not found
P16885	[1-phosphatidylinositol-4,5-bisphosphate phosphodiesterase gamma-2, (EC 3.1.4.11) (Phosphoinositide phospholipase C) (PLC-gamma-2), (Phospholipase C-gamma-2) (PLC-IV).]	Go Component not found
Q93088	Betaine--homocysteine S-methyltransferase 1 (EC 2.1.1.5).	Go Component not found
Q96T66	[Nicotinamide mononucleotide adenylyltransferase 3 (EC 2.7.7.1) (NMN, adenylyltransferase 3).]	Go Component not found
Q5T013	[Putative hydroxypyruvate isomerase (EC 5.3.1.22) (Endothelial cell, apoptosis protein E-CE1).]	Go Component not found
Q5TD12	[Serine palmitoyltransferase, long chain base subunit 2-like, (Aminotransferase 2) (Fragment).]	Go Component not found
Q8N8Q3	[CDNA FLJ39025 fis, clone NT2RP7004559, weakly similar to ENDONUCLEASE, C1F12.06C (EC 3.1.-.-) (Putative uncharacterized protein FLJ35220).]	Go Component not found
Q9Y2C4	Endonuclease G-like 1 (EC 3.1.30.-) (Endo G-like).	Go Component not found
O94788	[Retinal dehydrogenase 2 (EC 1.2.1.36) (RalDH2) (RALDH 2) (RALDH(II)), (Retinaldehyde-specific dehydrogenase type 2) (Aldehyde dehydrogenase, family 1 member A2).]	Go Component not found
P55073	[Type III iodothyronine deiodinase (EC 1.97.1.11) (Type-III 5'-, deiodinase) (DIOIII) (Type 3 DI) (5DIII).]	Go Component not found
Q504Q3	[PAB-dependent poly(A)-specific ribonuclease subunit 2 (EC 3.1.13.4), (hPan2) (Inactive ubiquitin carboxyl-terminal hydrolase 52).]	Go Component not found
Q58A53	[UDP-N-acetyl-alpha-D-galactosamine:polypeptide N-, acetylgalactosaminyltransferase 16.]	Go Component not found
Q6NXE8	Protein-tyrosine phosphatase domain-containing protein 1.	Go Component not found
Q8IVL5	[Prolyl 3-hydroxylase 2 precursor (EC 1.14.11.7) (Leprecan-like protein, 1) (Myxoid liposarcoma-associated protein 4).]	Go Component not found
Q9C0C9	[Ubiquitin-conjugating enzyme E2 O (EC 6.3.2.19) (Ubiquitin-protein, ligase O) (Ubiquitin carrier protein O) (Ubiquitin-conjugating enzyme, E2 of 230 kDa) (E2-230K).]	Go Component not found
P22466	[Galanin precursor [Contains: Galanin; Galanin message-associated, peptide (GMAP)].]	Go Component not found
O43451	[Maltase-glucoamylase, intestinal [Includes: Maltase (EC 3.2.1.20), (Alpha-glucosidase); Glucoamylase (EC 3.2.1.3) (Glucan 1,4-alpha-, glucosidase)].]	Go Component not found
P05060	[Secretogranin-1 precursor (Secretogranin I) (SgI) (Chromogranin-B), (CgB) [Contains: GAWK peptide; CCB peptide].]	Go Component not found
Q9UBU7	Protein DBF4 homolog (Activator of S phase Kinase).	Go Component not found
Q14449	Growth factor receptor-bound protein 14 (GRB14 adapter protein).	Go Component not found
O60256	[Phosphoribosyl pyrophosphate synthetase-associated protein 2 (PRPP, synthetase-associated protein 2) (41 kDa phosphoribosypyrophosphate, synthetase-associated protein) (PAP41).]	Go Component not found
Q14524	[Sodium channel protein type 5 subunit alpha (Sodium channel protein, type V subunit alpha) (Voltage-gated sodium channel subunit alpha, Nav1.5) (Sodium channel protein cardiac muscle subunit alpha) (HH1).]	Go Component not found
Q9Y302	[Protein GPR89 (Putative MAPK-activating protein PM01) (Putative NF-, kappa-B-activating protein 90).]	Go Component not found
Q8NBF2	NHL repeat-containing protein 2.	Go Component not found
Q15464	SH2 domain-containing adapter protein B.	Go Component not found
P49441	Inositol polyphosphate 1-phosphatase (EC 3.1.3.57) (IPPase) (IPP).	Go Component not found
P01034	[Cystatin-C precursor (Cystatin-3) (Neuroendocrine basic polypeptide), (Gamma-trace) (Post-gamma-globulin).]	Go Component not found
P52888	Thimet oligopeptidase (EC 3.4.24.15) (Endopeptidase 24.15) (MP78).	Go Component not found
Q7Z5K1	Leukocyte-derived arginine aminopeptidase long form variant.	Go Component not found
P08185	[Corticosteroid-binding globulin precursor (CBG) (Transcortin) (Serpin, A6).]	Go Component not found
Q9Y6I4	[Ubiquitin carboxyl-terminal hydrolase 3 (EC 3.1.2.15) (Ubiquitin, thioesterase 3) (Ubiquitin-specific-processing protease 3), (Deubiquitinating enzyme 3).]	Go Component not found
Q7KZ97	Antithrombin III variant.	Go Component not found
Q9UBX7	[Kallikrein-11 precursor (EC 3.4.21.-) (hK11) (Hippostasin) (Trypsin-, like protease) (Serine protease 20) [Contains: Kallikrein-11 inactive, chain 1; Kallikrein-11 inactive chain 2].]	Go Component not found

P01024	[Complement C3 precursor [Contains: Complement C3 beta chain;, Complement C3 alpha chain; C3a anaphylatoxin; Complement C3b alpha', chain; Complement C3c alpha' chain fragment 1; Complement C3dg, fragment; Complement C3g fragment; Complement C3d fragment; Complement, C3f fragment; Complement C3c alpha' chain fragment 2].]	Go Component not found
Q9UP38	Frizzled-1 precursor (Fz-1) (hFz1) (FzE1).	Go Component not found
Q9NZT2	[Opioid growth factor receptor (OGFr) (Zeta-type opioid receptor) (7-60, protein).]	Go Component not found
Q12986	[Transcriptional repressor NF-X1 (EC 6.3.2.-) (Nuclear transcription, factor, X box-binding, 1).]	Go Component not found
O95416	Transcription factor SOX-14.	Go Component not found
O14709	[Zinc finger protein 197 (ZnF20) (Zinc finger protein with KRAB and, SCAN domains 9).]	Go Component not found
Q9H0U4	Ras-related protein Rab-1B.	Golgi
P02751	[Fibronectin precursor (FN) (Cold-insoluble globulin) (CIG) [Contains:, Ugl-Y1; Ugl-Y2; Ugl-Y3].]	Golgi
P45844	[ATP-binding cassette sub-family G member 1 (White protein homolog), (ATP-binding cassette transporter 8).]	Golgi
O00461	[Golgi integral membrane protein 4 (Golgi phosphoprotein 4) (Golgi, integral membrane protein, cis) (GIMPc) (Golgi-localized, phosphoprotein of 130 kDa) (Golgi phosphoprotein of 130 kDa).]	Golgi
O14795	Protein unc-13 homolog B (Munc13-2) (munc13).	Golgi
Q9H2K0	[Translation initiation factor IF-3, mitochondrial precursor (IF-3Mt), (IF3(mt)) (IF-3(Mt)) (IF3mt).]	Mitochondrial
P54098	[DNA polymerase subunit gamma-1 (EC 2.7.7.7) (Mitochondrial DNA, polymerase catalytic subunit) (PolG-alpha).]	Mitochondrial
Q9Y2Z9	Ubiquinone biosynthesis monooxygenase COQ6 (EC 1.14.13.-).	Mitochondrial
P49748	[Very long-chain specific acyl-CoA dehydrogenase, mitochondrial, precursor (EC 1.3.99.-) (VLCAD).]	Mitochondrial
P54289	[Voltage-dependent calcium channel subunit alpha-2/delta-1 precursor, (Voltage-gated calcium channel subunit alpha-2/delta-1) [Contains:, Voltage-dependent calcium channel subunit alpha-2-1; Voltage-dependent, calcium channel subunit delta-1].]	Neural
P07197	[Neurofilament medium polypeptide (NF-M) (Neurofilament triplet M, protein) (160 kDa neurofilament protein) (Neurofilament 3).]	Neural
Q9H6Z4	Ran-binding protein 3 (RanBP3).	Nuclear
O95071	[E3 ubiquitin-protein ligase EDD1 (EC 6.3.2.-) (Hyperplastic discs, protein homolog) (hHYD) (Progestin-induced protein).]	Nuclear
P11387	DNA topoisomerase 1 (EC 5.99.1.2) (DNA topoisomerase I).	Nuclear
Q9HCK8	[Chromodomain-helicase-DNA-binding protein 8 (EC 3.6.1.-) (ATP-, dependent helicase CHD8) (CHD-8) (Helicase with SNF2 domain 1).]	Nuclear
Q9NX07	tRNA selenocysteine-associated protein 1 (SECp43).	Nuclear
P80217	Interferon-induced 35 kDa protein (IFP 35).	Nuclear
Q96T60	[Bifunctional polynucleotide phosphatase/kinase (Polynucleotide kinase-, 3'-phosphatase) (DNA 5'-kinase/3'-phosphatase) [Includes:, Polynucleotide 3'-phosphatase (EC 3.1.3.32) (2'(3')-polynucleotidase);, Polynucleotide 5'-hydroxyl-kinase (EC 2.7.1.78)].]	Nuclear
P22626	Heterogeneous nuclear ribonucleoproteins A2/B1 (hnRNP A2 / hnRNP B1).	Nuclear
Q92565	[Rap guanine nucleotide exchange factor 5 (Guanine nucleotide exchange, factor for Rap1) (Related to Epac) (Repac) (M-Ras-regulated Rap GEF), (MR-GEF).]	Nuclear
Q6ZNQ5	[CDNA FLJ27339 fis, clone TMS09615, highly similar to TFIIH basal, transcription factor complex helicase subunit (EC 3.6.1.-).]	Nuclear
Q7L590	Protein MCM10 homolog (HsMCM10).	Nuclear
Q7Z401	C-myc promoter-binding protein (DENN domain-containing protein 4A).	Nuclear
P09884	DNA polymerase alpha catalytic subunit (EC 2.7.7.7).	Nuclear
Q15424	[Scaffold attachment factor B1 (SAB-B1) (SAF-B) (HSP27 estrogen, response element-TATA box-binding protein) (HSP27 ERE-TATA-binding, protein).]	Nuclear
P51523	Zinc finger protein 84 (Zinc finger protein HPF2).	Nuclear
Q9NXJ9	CDNA FLJ20203 fis, clone COLF1334.	Nuclear
Q9Y2X3	Nucleolar protein 5 (Nucleolar protein NOP5) (NOP58).	Nuclear

O60287	Nucleolar pre-ribosomal-associated protein 1 (Protein URB1).	Nuclear
Q92698	[DNA repair and recombination protein RAD54-like (EC 3.6.1.-) (RAD54, homolog) (hRAD54) (hHR54).]	Nuclear
Q15772	[Striated muscle preferentially expressed protein kinase (EC 2.7.11.1), (Aortic preferentially expressed protein 1) (APEG-1).]	Nuclear
Q05481	Zinc finger protein 91 (Zinc finger protein HTF10) (HPF7).	Nuclear
Q9NYW8	RB-associated KRAB zinc finger protein (RB-associated KRAB repressor).	Nuclear
Q01954	Zinc finger protein basonuclin-1.	Nuclear
Q9UL36	Zinc finger protein 236.	Nuclear
Q03924	[Zinc finger protein 117 (Zinc finger protein HPF9) (Provirus-linked, krueppel) (h-PLK).]	Nuclear
P04264	[Keratin, type II cytoskeletal 1 (Cytokeratin-1) (CK-1) (Keratin-1), (K1) (67 kDa cytokeratin) (Hair alpha protein).]	Other Filament or Tubule
P53350	[Serine/threonine-protein kinase PLK1 (EC 2.7.11.21) (Polo-like kinase, 1) (PLK-1) (Serine/threonine-protein kinase 13) (STPK13).]	Other Filament or Tubule
Q08043	[Alpha-actinin-3 (Alpha-actinin skeletal muscle isoform 3) (F-actin, cross-linking protein).]	Other Filament or Tubule
Q9NS87	[Kinesin-like protein KIF15 (Kinesin-like protein 2) (Serologically, defined breast cancer antigen NY-BR-62).]	Other Filament or Tubule
Q6P2Q9	[Pre-mRNA-processing-splicing factor 8 (Splicing factor Prp8) (PRP8, homolog) (220 kDa U5 snRNP-specific protein) (p220).]	Other Go Component
Q86VF7	Nebulin-related-anchoring protein (N-RAP).	Other Go Component
Q05707	Collagen alpha-1(XIV) chain precursor (Undulin).	Other Go Component
Q9UI47	Catenin alpha-3 (Alpha T-catenin) (Cadherin-associated protein).	Other Go Component
P12109	Collagen alpha-1(VI) chain precursor.	Other Go Component
P16118	[6-phosphofructo-2-kinase/fructose-2,6-biphosphatase 1 (6PF-2-K/Fru-, 2,6-P2ASE liver isozyme) [Includes: 6-phosphofructo-2-kinase, (EC 2.7.1.105); Fructose-2,6-bisphosphatase (EC 3.1.3.46)].]	Other Go Component
Q6ZQW4	CDNA FLJ46848 fis, clone UTERU3005422.	Other Go Component
Q6ZP02	CDNA FLJ26785 fis, clone PRS04357.	Other Go Component
Q8N8V9	CDNA FLJ38783 fis, clone LIVER2001191.	Other Go Component
P01619	Ig kappa chain V-III region B6.	Other Go Component
Q6ZP44	CDNA FLJ26520 fis, clone KDN08032.	Other Go Component
Q6ZVA4	[CDNA FLJ42828 fis, clone BRCAN2017442, weakly similar to Homo sapiens, sarcosine dehydrogenase (SARDH).]	Other Go Component
Q5PR22	COL5A2 protein.	Other Go Component
P78363	[Retinal-specific ATP-binding cassette transporter (ATP-binding, cassette sub-family A member 4) (RIM ABC transporter) (RIM protein), (RmP) (Stargardt disease protein).]	Other Membrane
Q9NYZ4	[Sialic acid-binding Ig-like lectin 8 precursor (Siglec-8), (Sialoadhesin family member 2) (SAF-2) (CD329 antigen) (CDw329).]	Other Membrane
P51693	Amyloid-like protein 1 precursor (APLP) (APLP-1) [Contains: C30].	Other Membrane
Q7Z443	Polycystic kidney disease 1-like protein 3.	Other Membrane
Q07666	[KH domain-containing, RNA-binding, signal transduction-associated, protein 1 (p21 Ras GTPase-activating protein-associated p62) (GAP-, associated tyrosine phosphoprotein p62) (Src-associated in mitosis 68, kDa protein) (Sam68) (p68).]	Other Membrane
O00533	[Neural cell adhesion molecule L1-like protein precursor (Close homolog, of L1).]	Other Membrane
Q5XKQ4	Alpha-2-glycoprotein 1, zinc-binding.	Other Membrane
Q6NVV9	ADAM5P protein.	Other Membrane
Q8N7D8	CDNA FLJ25756 fis, clone TST06241.	Other Membrane
Q07065	Cytoskeleton-associated protein 4 (63 kDa membrane protein) (p63).	Other Membrane
Q12860	[Contactin-1 precursor (Neural cell surface protein F3) (Glycoprotein, gp135).]	Other Membrane
P29475	[Nitric-oxide synthase, brain (EC 1.14.13.39) (NOS type I) (Neuronal, NOS) (N-NOS) (nNOS) (Constitutive NOS) (NC-NOS) (bNOS).]	Other Membrane

P35498	[Sodium channel protein type 1 subunit alpha (Sodium channel protein, type I subunit alpha) (Voltage-gated sodium channel subunit alpha, Nav1.1) (Sodium channel protein, brain I subunit alpha).]	Other Membrane
Q6ZMB0	[UDP-GlcNAc:betaGal beta-1,3-N-acetylglucosaminyltransferase 6, (EC 2.4.1.-) (Beta3Gn-T6) (BGnT-6) (Beta-1,3-N-, acetylglucosaminyltransferase-6) (Core 3 synthase).]	Other Membrane
O75976	[Carboxypeptidase D precursor (EC 3.4.17.22) (Metallocarboxypeptidase, D) (gp180).]	Other Membrane
Q9Y3Q0	[N-acetylated-alpha-linked acidic dipeptidase 2 (EC 3.4.17.21) (N-, acetylated-alpha-linked acidic dipeptidase II) (NAALADase II).]	Other Membrane
Q15391	[P2Y purinoceptor 14 (P2Y14) (UDP-glucose receptor) (G-protein coupled, receptor 105).]	Other Membrane
P98155	[Very low-density lipoprotein receptor precursor (VLDL receptor) (VLDL-, R).]	Other Membrane
Q9BXR5	Toll-like receptor 10 precursor (CD290 antigen).	Other Membrane
Q6IEU2	Olfactory receptor OR14-38.	Other Membrane
P16144	Integrin beta-4 precursor (GP150) (CD104 antigen).	Plasma Membrane
Q12918	[Killer cell lectin-like receptor subfamily B member 1 (Natural killer, cell surface protein P1A) (HNKR-P1a) (NKR-P1A) (C-type lectin domain, family 5 member B) (CD161 antigen).]	Plasma Membrane
Q9Y2J2	[Band 4.1-like protein 3 (4.1B) (Differentially expressed in, adenocarcinoma of the lung protein 1) (DAL-1).]	Plasma Membrane
P11362	[Basic fibroblast growth factor receptor 1 precursor (EC 2.7.10.1), (FGFR-1) (bFGF-R) (Fms-like tyrosine kinase 2) (c-fgr) (CD331, antigen).]	Plasma Membrane
Q92823	[Neuronal cell adhesion molecule precursor (Nr-CAM) (NgCAM-related cell, adhesion molecule) (Ng-CAM-related) (hBravo).]	Plasma Membrane
Q8WTV0	[Scavenger receptor class B member 1 (SRB1) (SR-BI) (CD36 antigen-like, 1) (CD36 and LIMPII analogous 1) (CLA-1) (Collagen type I receptor,, thrombospondin receptor-like 1).]	Plasma Membrane
O95456	[Down syndrome critical region protein 2 (Leucine-rich protein C21-, LRP).]	Plasma Membrane
Q92859	Neogenin precursor.	Plasma Membrane
Q9Y5I3	Protocadherin alpha 1 precursor (PCDH-alpha1).	Plasma Membrane
O75323	Protein NipSnap2 (Glioblastoma amplified sequence).	Plasma Membrane
O60243	Heparan-sulfate 6-O-sulfotransferase 1 (EC 2.8.2.-) (HS6ST-1).	Plasma Membrane
O95151	Calcium activated chloride channel family member 1.	Plasma Membrane
Q8NER1	[Transient receptor potential cation channel subfamily V member 1, (TrpV1) (osm-9-like TRP channel 1) (OTRPC1) (Vanilloid receptor 1), (Capsaicin receptor).]	Plasma Membrane
Q13822	[Ectonucleotide pyrophosphatase/phosphodiesterase family member 2, precursor (EC 3.1.4.39) (E-NPP 2) (Extracellular lysophospholipase D), (LysoPLD) (Autotaxin).]	Plasma Membrane
P21860	[Receptor tyrosine-protein kinase erbB-3 precursor (EC 2.7.10.1) (c-, erbB3) (Tyrosine kinase-type cell surface receptor HER3).]	Plasma Membrane
P30968	Gonadotropin-releasing hormone receptor (GnRH receptor) (GnRH-R).	Plasma Membrane
P24046	[Gamma-aminobutyric acid receptor subunit rho-1 precursor (GABA(A), receptor subunit rho-1).]	Plasma Membrane
P29320	[Ephrin type-A receptor 3 precursor (EC 2.7.10.1) (Tyrosine-protein, kinase receptor ETK1) (HEK) (HEK4) (Tyrosine-protein kinase TYRO4).]	Plasma Membrane
P17181	Interferon-alpha/beta receptor alpha chain precursor (IFN-alpha-REC).	Plasma Membrane
Q08357	Solute carrier family 20 member 2 (Gibbon leukemia virus receptor 2).	Plasma Membrane
Q96IY1	Kinetochore-associated protein NSL1 homolog.	Protein Complex
P68871	[Hemoglobin subunit beta (Hemoglobin beta chain) (Beta-globin), [Contains: LVV-hemorphin-7].]	Protein Complex
Q99460	[26S proteasome non-ATPase regulatory subunit 1 (26S proteasome, regulatory subunit RPN2) (26S proteasome regulatory subunit S1) (26S, proteasome subunit p112).]	Protein Complex

Q53F97	Alpha 2 globin variant (Fragment).	Protein Complex
P61289	[Proteasome activator complex subunit 3 (Proteasome activator 28, subunit gamma) (PA28gamma) (PA28g) (Activator of multicatalytic, protease subunit 3) (11S regulator complex subunit gamma) (REG-gamma), (Ki nuclear autoantigen).]	Protein Complex
P02675	Fibrinogen beta chain precursor [Contains: Fibrinopeptide B].	Protein Complex
P78552	[Interleukin-13 receptor alpha-1 chain precursor (IL-13R-alpha-1) (IL-, 13RA-1) (Cancer/testis antigen 19) (CT19) (CD213a1 antigen).]	Protein Complex
Q96DR0	[CDNA FLJ30536 fis, clone BRAWH2001166, weakly similar to BETA-, GALACTOSIDASE (EC 3.2.1.23).]	Protein Complex
Q96PK6	[RNA-binding protein 14 (RNA-binding motif protein 14) (RRM-containing, coactivator activator/modulator) (Synaptotagmin-interacting protein), (SYT-interacting protein).]	Protein Complex
P29374	[AT-rich interactive domain-containing protein 4A (ARID domain-, containing protein 4A) (Retinoblastoma-binding protein 1) (RBBP-1).]	Transcription
Q9GZX5	[Zinc finger protein 350 (Zinc finger protein ZBRK1) (Zinc finger and, BRCA1-interacting protein with a KRAB domain 1) (KRAB zinc finger, protein ZFQR).]	Transcription
O00268	[Transcription initiation factor TFIID subunit 4 (TBP-associated factor, 4) (Transcription initiation factor TFIID 135 kDa subunit), (TAF(II)135) (TAFII-135) (TAFII135) (TAFII-130) (TAFII130) (RNA, polymerase II TBP-associated factor subunit C).]	Transcription
Q02410	[Amyloid beta A4 precursor protein-binding family A member 1 (Neuron-, specific X11 protein) (Neuronal Munc18-1-interacting protein 1) (Mint-, 1) (Adapter protein X11alpha).]	Vesicle
P06133	[UDP-glucuronosyltransferase 2B4 precursor (EC 2.4.1.17) (UDPGT), (Hyodeoxycholic acid) (HLUG25) (UDPGTh-1).]	Vesicle
O43601	Putative uncharacterized protein (Fragment).	Vesicle
P02787	[Serotransferrin precursor (Transferrin) (Siderophilin) (Beta-1-metal-, binding globulin).]	Vesicle
Q9BY49	[Peroxisomal trans-2-enoyl-CoA reductase (EC 1.3.1.38) (TERP) (HPDHase), (pVI-ARL) (2,4-dienoyl-CoA reductase-related protein) (DCR-RP).]	Vesicle
Q9UJM8	Hydroxyacid oxidase 1 (EC 1.1.3.15) (HAOX1) (Glycolate oxidase) (GOX).	Vesicle
Q8WZA1	[Protein O-linked-mannose beta-1,2-N-acetylglucosaminyltransferase 1, (EC 2.4.1.-) (POMGnT1) (UDP-GlcNAc:alpha-D-mannoside beta-1,2-N-, acetylglucosaminyltransferase I.2) (GnT I.2).]	Vesicle

P3 of Data 4, visit 2		
**Uniprot Number**	**Protein Name**	**GO Component CATEGORY**
Q9BYG3	[MKI67 FHA domain-interacting nucleolar phosphoprotein (Nucleolar, protein interacting with the FHA domain of pKI-67) (hNIFK) (Nucleolar, phosphoprotein Nopp34).]	Chromosomal
P27816	Microtubule-associated protein 4 (MAP 4).	Cytoskeletal
P02649	Apolipoprotein E precursor (Apo-E).	Cytosolic
P21127	[PITSLRE serine/threonine-protein kinase CDC2L1 (EC 2.7.11.22), (Galactosyltransferase-associated protein kinase p58/GTA) (Cell, division cycle 2-like protein kinase 1) (CLK-1) (CDK11) (p58 CLK-1).]	Cytosolic
Q9Y217	Myotubularin-related protein 6 (EC 3.1.3.-).	Cytosolic
P18124	60S ribosomal protein L7.	Cytosolic
Q9H190	Syntenin-2 (Syndecan-binding protein 2).	Cytosolic
Q5VWL4	[Phospholipase C, epsilon 1 (Phospholipase C, epsilon 1, isoform, CRA_a).]	Cytosolic
Q86VW2	RhoA/RAC/CDC42 exchange factor (RAC/CDC42/Rho exchange factor GEFT).	Cytosolic
Q9H902	Receptor expression-enhancing protein 1.	Cytosolic
Q9NPP4	[NLR family CARD domain-containing protein 4 (Caspase recruitment, domain-containing protein 12) (Ice protease-activating factor) (Ipaf), (CARD, LRR, and NACHT-containing protein) (Clan protein).]	Cytosolic
O15379	Histone deacetylase 3 (HD3) (RPD3-2) (SMAP45).	Cytosolic
Q8WXG6	[MAP kinase-activating death domain protein (Differentially expressed, in normal and neoplastic cells) (Insulinoma glucagonoma clone 20), (Rab3 GDP/GTP exchange factor).]	Cytosolic
P22760	Arylacetamide deacetylase (EC 3.1.1.-) (AADAC).	ER
P01009	[Alpha-1-antitrypsin precursor (Alpha-1 protease inhibitor) (Alpha-1-, antiproteinase).]	Extracellular
P19827	[Inter-alpha-trypsin inhibitor heavy chain H1 precursor (ITI heavy, chain H1) (Inter-alpha-inhibitor heavy chain 1) (Inter-alpha-trypsin, inhibitor complex component III) (Serum-derived hyaluronan-associated, protein) (SHAP).]	Extracellular
Q08380	[Galectin-3-binding protein precursor (Lectin galactoside-binding, soluble 3-binding protein) (Mac-2-binding protein) (Mac-2 BP) (MAC2BP), (Tumor-associated antigen 90K).]	Extracellular
P20062	Transcobalamin-2 precursor (Transcobalamin II) (TCII) (TC II).	Extracellular
P01023	Alpha-2-macroglobulin precursor (Alpha-2-M).	Extracellular
P10909	[Clusterin precursor (Complement-associated protein SP-40,40), (Complement cytolysis inhibitor) (CLI) (NA1/NA2) (Apolipoprotein J), (Apo-J) (Testosterone-repressed prostate message 2) (TRPM-2), [Contains: Clusterin beta chain (ApoJalpha) (Complement cytolysis, inhibitor a chain); Clusterin alpha chain (ApoJbeta) (Complement, cytolysis inhibitor b chain)].]	Extracellular
P02768	Serum albumin precursor.	Extracellular
P41222	[Prostaglandin-H2 D-isomerase precursor (EC 5.3.99.2) (Lipocalin-type, prostaglandin-D synthase) (Glutathione-independent PGD synthetase), (Prostaglandin-D2 synthase) (PGD2 synthase) (PGDS2) (PGDS) (Beta-trace, protein) (Cerebrin-28).]	Extracellular
Q8IUK7	ALB protein.	Extracellular
Q9Y4X0	AMME syndrome candidate gene 1 protein.	Go Component not found
Q5SR76	Uncharacterized protein C10orf93.	Go Component not found
P01034	[Cystatin-C precursor (Cystatin-3) (Neuroendocrine basic polypeptide), (Gamma-trace) (Post-gamma-globulin).]	Go Component not found
Q13642	[Four and a half LIM domains protein 1 (FHL-1) (Skeletal muscle LIM-, protein 1) (SLIM 1) (SLIM).]	Go Component not found
P15173	Myogenin (Myogenic factor 4) (Myf-4).	Go Component not found

Q8NGI7	Olfactory receptor 10V1.	Go Component not found
Q99447	[Ethanolamine-phosphate cytidylyltransferase (EC 2.7.7.14), (Phosphorylethanolamine transferase) (CTP:phosphoethanolamine, cytidylyltransferase).]	Go Component not found
Q7Z2W7	[Transient receptor potential cation channel subfamily M member 8, (Transient receptor potential-p8) (Trp-p8) (Long transient receptor, potential channel 6) (LTrpC6).]	Go Component not found
O95498	[Vascular non-inflammatory molecule 2 precursor (Vanin-2), (Glycosylphosphatidyl inositol-anchored protein GPI-80) (Protein FOAP-, 4).]	Go Component not found
Q53FE4	Uncharacterized protein C4orf17.	Go Component not found
Q5GH70	XK-related protein 9.	Go Component not found
Q5HYI3	Putative uncharacterized protein DKFZp686N1815.	Go Component not found
Q5TCW5	Chromosome 9 open reading frame 127.	Go Component not found
Q6N066	[Putative uncharacterized protein DKFZp686D14128 (Kelch repeat and BTB, (POZ) domain containing 3, isoform CRA_b) (Kelch repeat and BTB (POZ), domain containing 3).]	Go Component not found
Q6ZSP9	CDNA FLJ45310 fis, clone BRHIP3004774.	Go Component not found
Q8N0W7	[Fragile X mental retardation 1 neighbor protein (Sarcoma antigen NY-, SAR-35) (Cancer/testis antigen 37) (CT37).]	Go Component not found
Q8NAI6	CDNA FLJ35314 fis, clone PROST2010606.	Go Component not found
Q9H3S5	[GPI mannosyltransferase 1 (EC 2.4.1.-) (GPI mannosyltransferase I), (GPI-MT-I) (Phosphatidylinositol-glycan biosynthesis class M protein), (PIG-M).]	Go Component not found
Q9NQW1	[Protein transport protein Sec31B (SEC31-related protein B) (SEC31B-1), (SEC31-like 2).]	Go Component not found
Q9UBY2	Neurochondrin-2 (Neurochondrin, isoform CRA_b).	Go Component not found
Q9Y602	[Cysteine sulfinic acid decarboxylase-related protein 1 (Cysteine, sulfinic acid decarboxylase, isoform CRA_c).]	Go Component not found
Q15389	Angiopoietin-1 precursor (ANG-1).	Go Component not found
Q9Y574	Ankyrin repeat and SOCS box protein 4 (ASB-4).	Go Component not found
Q8WVQ1	[Soluble calcium-activated nucleotidase 1 (EC 3.6.1.6) (SCAN-1), (Apyrase homolog) (Putative NF-kappa-B-activating protein 107), (Putative MAPK-activating protein PM09).]	Go Component not found
Q8NB25	Uncharacterized protein C6orf60.	Go Component not found
O94964	Uncharacterized protein C20orf117.	Go Component not found
O75829	[Chondromodulin-1 precursor (Chondromodulin-I) (ChM-I) (Leukocyte cell-, derived chemotaxin 1) [Contains: Chondrosurfactant protein (CH-SP)].]	Go Component not found
P09917	Arachidonate 5-lipoxygenase (EC 1.13.11.34) (5-lipoxygenase) (5-LO).	Go Component not found
Q13164	[Mitogen-activated protein kinase 7 (EC 2.7.11.24) (Extracellular, signal-regulated kinase 5) (ERK-5) (ERK4) (BMK1 kinase).]	Go Component not found
P02144	Myoglobin.	Go Component not found
Q8TAT5	Endonuclease VIII-like 3 (Nei-like 3) (DNA glycosylase FPG2).	Go Component not found
Q9BZF1	[Oxysterol-binding protein-related protein 8 (OSBP-related protein 8), (ORP-8).]	Go Component not found
Q92733	[Proline-rich protein PRCC (Papillary renal cell carcinoma, translocation-associated gene protein).]	Go Component not found
Q9BVG9	[Phosphatidylserine synthase 2 (EC 2.7.8.-) (PtdSer synthase 2) (PSS-2), (Serine-exchange enzyme II).]	Go Component not found
Q9BQ04	[RNA-binding protein 4B (RNA-binding motif protein 4B) (RNA-binding, protein 30) (RNA-binding motif protein 30).]	Go Component not found
P51692	Signal transducer and activator of transcription 5B.	Go Component not found
P59817	[Suppressor of hairy wing homolog 1 (3'OY11.1) (Zinc finger protein, 280).]	Go Component not found
P98066	[Tumor necrosis factor-inducible protein TSG-6 precursor (TNF-, stimulated gene 6 protein) (Tumor necrosis factor, alpha-induced, protein 6) (Hyaluronate-binding protein).]	Go Component not found

Q53TQ3	Putative uncharacterized protein FLJ20309.	Go Component not found
Q5H966	Sex comb on midleg-like 1 (Drosophila) (Fragment).	Go Component not found
Q9BZQ6	ER degradation-enhancing alpha-mannosidase-like 3.	Go Component not found
Q5VY27	Centrosomal protein 55kDa (Fragment).	Go Component not found
Q8WUA7	TBC1 domain family member 22A.	Go Component not found
Q9ULI0	ATPase family AAA domain-containing protein 2B.	Go Component not found
Q8NAN4	CDNA FLJ35074 fis, clone PLACE6001118 (HCG1979341, isoform CRA_a).	Go Component not found
Q8WWU5	TCP11b protein.	Go Component not found
Q9UBZ4	[DNA-(apurinic or apyrimidinic site) lyase 2 (EC 4.2.99.18) (Apurinic-, apyrimidinic endonuclease 2) (AP endonuclease 2) (APEX nuclease 2), (APEX nuclease-like 2) (AP endonuclease XTH2).]	Go Component not found
P35527	[Keratin, type I cytoskeletal 9 (Cytokeratin-9) (CK-9) (Keratin-9), (K9).]	Go Component not found
O00182	Galectin-9 (HOM-HD-21) (Ecalectin).	Go Component not found
O95631	Netrin-1 precursor.	Go Component not found
Q9Y6F1	[Poly [ADP-ribose] polymerase 3 (EC 2.4.2.30) (PARP-3) (NAD(+) ADP-, ribosyltransferase 3) (Poly[ADP-ribose] synthetase 3) (pADPRT-3), (hPARP-3) (IRT1).]	Go Component not found
Q6IQ26	Rab6-interacting protein 1 (Rab6IP1).	Go Component not found
Q9UQ26	[Regulating synaptic membrane exocytosis protein 2 (Rab3-interacting, molecule 2) (RIM 2).]	Go Component not found
O75529	[TAF5-like RNA polymerase II p300/CBP-associated factor-associated, factor 65 kDa subunit 5L (PCAF-associated factor 65 beta) (PAF65-, beta).]	Go Component not found
P49638	Alpha-tocopherol transfer protein (Alpha-TTP).	Go Component not found
Q59FN2	P21-activated kinase 2 variant (Fragment).	Go Component not found
Q5HY98	Zinc finger protein 766.	Go Component not found
Q9NSV4	Protein diaphanous homolog 3 (Diaphanous-related formin-3) (DRF3).	Go Component not found
Q8IX12	[Cell division cycle and apoptosis regulator protein 1 (Cell cycle and, apoptosis regulatory protein 1) (CARP-1) (Death inducer with SAP, domain).]	Go Component not found
Q5VZE5	Protein MAK10 homolog (Embryonic growth-associated protein homolog).	Go Component not found
Q6MZQ6	Putative uncharacterized protein DKFZp686G11190.	Go Component not found
Q6ZUY1	CDNA FLJ43215 fis, clone FEBRA2021908.	Go Component not found
Q8N9I5	CDNA FLJ37092 fis, clone BRACE2017934.	Go Component not found
Q96D53	Uncharacterized aarF domain-containing protein kinase 4 (EC 2.7.11.-).	Go Component not found
Q92600	Protein RCD1 homolog.	Go Component not found
Q8IZ40	REST corepressor 2.	Go Component not found
Q9H903	[CDNA FLJ13105 fis, clone NT2RP3002351, weakly similar to Human NAD-, dependent methylene tetrahydrofolate dehydrogenase cyclohydrolase, (EC 1.5.1.15).]	Go Component not found
Q6NUN0	[Acyl-coenzyme A synthetase ACSM5, mitochondrial precursor, (EC 6.2.1.2).]	Go Component not found
Q9UF92	Putative uncharacterized protein DKFZp434K098 (Fragment).	Go Component not found
P02751	[Fibronectin precursor (FN) (Cold-insoluble globulin) (CIG) [Contains:, Ugl-Y1; Ugl-Y2; Ugl-Y3].]	Golgi
Q9Y3R5	Protein dopey-2.	Golgi
Q6UWV6	[Ectonucleotide pyrophosphatase/phosphodiesterase family member 7, precursor (EC 3.1.4.12) (E-NPP7) (NPP-7) (Alkaline sphingomyelin, phosphodiesterase) (Intestinal alkaline sphingomyelinase) (Alk-SMase).]	Golgi

O14662	Syntaxin-16 (Syn16).	Golgi
Q01085	Nucleolysin TIAR (TIA-1-related protein).	Lysosomal
O00411	[DNA-directed RNA polymerase, mitochondrial precursor (EC 2.7.7.6), (MtRPOL).]	Mitochondrial
Q15878	[Voltage-dependent R-type calcium channel subunit alpha-1E (Voltage-, gated calcium channel subunit alpha Cav2.3) (Calcium channel, L type,, alpha-1 polypeptide, isoform 6) (Brain calcium channel II) (BII).]	Neural
O15554	[Intermediate conductance calcium-activated potassium channel protein 4, (SK4) (KCa4) (IK1) (IKCa1) (Putative Gardos channel).]	Neural
Q9NR82	[Potassium voltage-gated channel subfamily KQT member 5 (Voltage-gated, potassium channel subunit Kv7.5) (Potassium channel subunit alpha, KvLQT5) (KQT-like 5).]	Neural
Q92698	[DNA repair and recombination protein RAD54-like (EC 3.6.1.-) (RAD54, homolog) (hRAD54) (hHR54).]	Nuclear
Q8WUN3	Zinc finger protein 252 (Fragment).	Nuclear
Q9P287	BRCA2 and CDKN1A-interacting protein (Protein TOK-1).	Nuclear
O00148	[ATP-dependent RNA helicase DDX39 (EC 3.6.1.-) (DEAD box protein 39), (Nuclear RNA helicase URH49).]	Nuclear
P17032	Zinc finger protein 37A (Zinc finger protein KOX21).	Nuclear
P02533	[Keratin, type I cytoskeletal 14 (Cytokeratin-14) (CK-14) (Keratin-14), (K14).]	Other Filament or Tubule
P35908	[Keratin, type II cytoskeletal 2 epidermal (Cytokeratin-2e) (K2e) (CK, 2e) (keratin-2).]	Other Filament or Tubule
P04264	[Keratin, type II cytoskeletal 1 (Cytokeratin-1) (CK-1) (Keratin-1), (K1) (67 kDa cytokeratin) (Hair alpha protein).]	Other Filament or Tubule
P08779	[Keratin, type I cytoskeletal 16 (Cytokeratin-16) (CK-16) (Keratin-16), (K16).]	Other Filament or Tubule
P04264	[Keratin, type II cytoskeletal 1 (Cytokeratin-1) (CK-1) (Keratin-1), (K1) (67 kDa cytokeratin) (Hair alpha protein).]	Other Filament or Tubule
P13645	[Keratin, type I cytoskeletal 10 (Cytokeratin-10) (CK-10) (Keratin-10), (K10).]	Other Filament or Tubule
P02538	[Keratin, type II cytoskeletal 6A (Cytokeratin-6A) (CK 6A) (K6a, keratin) (Cytokeratin-6D) (CK 6D).]	Other Filament or Tubule
Q8NEV4	Myosin IIIA (EC 2.7.11.1).	Other Filament or Tubule
P01616	Ig kappa chain V-II region MIL.	Other Go Component
Q6LBZ2	Histone H4 gene (Fragment).	Other Go Component
Q6NWN0	Putative uncharacterized protein FLJ12684.	Other Go Component
Q96LT2	CDNA FLJ25086 fis, clone CBL08645.	Other Go Component
Q9UI62	PRO0457.	Other Go Component
Q93033	[Immunoglobulin superfamily member 2 precursor (Glu-Trp-Ile EWI motif-, containing protein 101) (EWI-101) (Cell surface glycoprotein V7), (CD101 antigen).]	Other Membrane
Q9UFR6	Putative uncharacterized protein DKFZp434B0335 (Fragment).	Other Membrane
O43603	Galanin receptor type 2 (GAL2-R) (GALR2).	Other Membrane
Q9UHN6	Transmembrane protein 2.	Other Membrane
Q9NS84	[Carbohydrate sulfotransferase 7 (EC 2.8.2.17) (EC 2.8.2.-), (Chondroitin 6-sulfotransferase 2) (C6ST-2) (N-acetylglucosamine 6-O-, sulfotransferase 1) (GlcNAc6ST-4) (Galactose/N-acetylglucosamine/N-, acetylglucosamine 6-O-sulfotransferase 5) (GST-5).]	Other Membrane
Q6ZMK5	FLJ00322 protein (Fragment).	Other Membrane
Q14831	Metabotropic glutamate receptor 7 precursor (mGluR7).	Plasma Membrane
P49190	Parathyroid hormone receptor precursor (PTH2 receptor).	Plasma Membrane
Q9Y493	Zonadhesin precursor.	Plasma Membrane
P16066	[Atrial natriuretic peptide receptor A precursor (ANP-A) (ANPRA) (GC-A), (Guanylate cyclase) (EC 4.6.1.2) (NPR-A) (Atrial natriuretic peptide, A-type receptor).]	Plasma Membrane
P20648	[Potassium-transporting ATPase alpha chain 1 (EC 3.6.3.10) (Proton, pump) (Gastric H(+)/K(+) ATPase subunit alpha).]	Plasma Membrane
Q14953	[Killer cell immunoglobulin-like receptor 2DS5 precursor (MHC class I, NK cell receptor) (Natural killer-associated transcript 9) (NKAT-9), (CD158g antigen).]	Plasma Membrane

Q9UHW9	[Solute carrier family 12 member 6 (Electroneutral potassium-chloride, cotransporter 3) (K-Cl cotransporter 3).]	Plasma Membrane
P02671	Fibrinogen alpha chain precursor [Contains: Fibrinopeptide A].	Protein Complex
Q9NU98	OTTHUMP00000016594.	Protein Complex
P02675	Fibrinogen beta chain precursor [Contains: Fibrinopeptide B].	Protein Complex
O43752	Syntaxin-6.	Vesicle
P02787	[Serotransferrin precursor (Transferrin) (Siderophilin) (Beta-1-metal-, binding globulin).]	Vesicle

S3 of Data 4, visit 2		
Uniprot Number	Protein Name	GO Component CATEGORY
O60264	[SWI/SNF-related matrix-associated actin-dependent regulator of, chromatin subfamily A member 5 (EC 3.6.1.-) (SWI/SNF-related matrix-, associated actin-dependent regulator of chromatin A5) (Sucrose, nonfermenting protein 2 homolog) (hSNF2H).]	Chromosomal
O14777	[Kinetochore protein Hec1 (HsHec1) (Kinetochore-associated protein 2), (Highly expressed in cancer protein) (Retinoblastoma-associated, protein HEC).]	Chromosomal
P06396	[Gelsolin precursor (Actin-depolymerizing factor) (ADF) (Brevin), (AGEL).]	Cytoskeletal
Q6H8Q1	[Actin-binding LIM protein 2 (Actin-binding LIM protein family member, 2) (abLIM-2).]	Cytoskeletal
P58107	Epiplakin (450 kDa epidermal antigen).	Cytoskeletal
Q9UBN4	[Short transient receptor potential channel 4 (TrpC4) (Trp-related, protein 4) (hTrp-4) (hTrp4).]	Cytoskeletal
O43602	[Neuronal migration protein doublecortin (Lissencephalin-X) (Lis-X), (Doublin).]	Cytoskeletal
Q15813	Tubulin-specific chaperone E (Tubulin-folding cofactor E).	Cytoskeletal
P19338	Nucleolin (Protein C23).	Cytoskeletal
Q9C0H9	[p130Cas-associated protein (p140Cap) (SNAP-25-interacting protein), (SNIP).]	Cytoskeletal
Q08495	Dematin (Erythrocyte membrane protein band 4.9).	Cytoskeletal
Q14244	[Ensconsin (Microtubule-associated protein 7) (Epithelial microtubule-, associated protein of 115 kDa) (E-MAP-115).]	Cytoskeletal
O95433	Activator of 90 kDa heat shock protein ATPase homolog 1 (AHA1) (p38).	Cytosolic
Q9Y408	Putative uncharacterized protein DKFZp566D133 (Fragment).	Cytosolic
P41240	[Tyrosine-protein kinase CSK (EC 2.7.10.2) (C-SRC kinase) (Protein-, tyrosine kinase CYL).]	Cytosolic
Q14684	RRP1-like protein B.	Cytosolic
P37198	Nuclear pore glycoprotein p62 (62 kDa nucleoporin).	Cytosolic
Q8WYL5	[Protein phosphatase Slingshot homolog 1 (EC 3.1.3.48) (EC 3.1.3.16), (SSH-1L) (hSSH-1L).]	Cytosolic
P27037	[Activin receptor type-2A precursor (EC 2.7.11.30) (Activin receptor, type IIA) (ACTR-IIA) (ACTRIIA).]	Cytosolic
P49454	[Centromere protein F (Kinetochore protein CENP-F) (Mitosin) (AH, antigen).]	Cytosolic
Q00597	Fanconi anemia group C protein (Protein FACC).	Cytosolic
Q13418	[Integrin-linked protein kinase (EC 2.7.11.1) (ILK-1) (ILK-2) (59 kDa, serine/threonine-protein kinase) (p59ILK).]	Cytosolic
Q86UZ3	Zinc finger protein 513 (Putative uncharacterized protein FLJ32203).	Cytosolic
Q92876	[Kallikrein-6 precursor (EC 3.4.21.-) (Protease M) (Neurosin) (Zyme), (SP59) (Serine protease 9) (Serine protease 18).]	Cytosolic
Q9UPN3	[Microtubule-actin cross-linking factor 1, isoforms 1/2/3/5 (Actin, cross-linking family protein 7) (Macrophin-1) (Trabeculin-alpha) (620, kDa actin-binding protein) (ABP620).]	Cytosolic
P26639	[Threonyl-tRNA synthetase, cytoplasmic (EC 6.1.1.3) (Threonine--tRNA, ligase) (ThrRS).]	Cytosolic
P22105	Tenascin-X precursor (TN-X) (Hexabrachion-like protein).	Cytosolic
Q16851	[UTP--glucose-1-phosphate uridylyltransferase (EC 2.7.7.9) (UDP-glucose, pyrophosphorylase) (UDPGP) (UGPase).]	Cytosolic
Q12830	[Nucleosome-remodeling factor subunit BPTF (Bromodomain and PHD finger-, containing transcription factor) (Fetal Alzheimer antigen) (Fetal Alz-, 50 clone 1 protein).]	Cytosolic
P57075	[Suppressor of T-cell receptor signaling 2 (STS-2) (Cbl-interacting, protein 4) (CLIP4) (T-cell ubiquitin ligand) (TULA).]	Cytosolic

Q9P2Y5	UV radiation resistance-associated gene protein (p63).	Cytosolic
P68402	[Platelet-activating factor acetylhydrolase IB subunit beta, (EC 3.1.1.47) (PAF acetylhydrolase 30 kDa subunit) (PAF-AH 30 kDa, subunit) (PAF-AH subunit beta) (PAFAH subunit beta).]	Cytosolic
O95164	[Ubiquitin-like protein 3 precursor (Membrane-anchored ubiquitin-fold, protein) (MUB) (HsMUB) (Protein HCG-1).]	Cytosolic
Q9ULU8	[Calcium-dependent secretion activator 1 (Calcium-dependent activator, protein for secretion 1) (CAPS-1).]	Cytosolic
Q13217	[DnaJ homolog subfamily C member 3 (Interferon-induced, double-stranded, RNA-activated protein kinase inhibitor) (Protein kinase inhibitor p58), (Protein kinase inhibitor of 58 kDa).]	Cytosolic
Q9NQ66	[1-phosphatidylinositol-4,5-bisphosphate phosphodiesterase beta-1, (EC 3.1.4.11) (Phosphoinositide phospholipase C) (Phospholipase C-, beta-1) (PLC-beta-1) (PLC-I) (PLC-154).]	Cytosolic
P48507	[Glutamate--cysteine ligase regulatory subunit (EC 6.3.2.2) (Gamma-, glutamylcysteine synthetase regulatory subunit) (Gamma-ECS regulatory, subunit) (GCS light chain) (Glutamate--cysteine ligase modifier, subunit).]	Cytosolic
O15067	[Phosphoribosylformylglycinamidine synthase (EC 6.3.5.3) (FGAM, synthase) (FGAMS) (Formylglycinamide ribotide amidotransferase), (FGARAT) (Formylglycinamide ribotide synthetase).]	Cytosolic
Q99259	[Glutamate decarboxylase 1 (EC 4.1.1.15) (Glutamate decarboxylase 67, kDa isoform) (GAD-67) (67 kDa glutamic acid decarboxylase).]	Cytosolic
Q9UMR2	[ATP-dependent RNA helicase DDX19B (EC 3.6.1.-) (DEAD box protein 19B), (DEAD box RNA helicase DEAD5).]	Cytosolic
P11413	Glucose-6-phosphate 1-dehydrogenase (EC 1.1.1.49) (G6PD).	Cytosolic
O14772	[Fucose-1-phosphate guanylyltransferase (EC 2.7.7.30) (GDP-L-fucose, pyrophosphorylase) (GDP-L-fucose diphosphorylase).]	Cytosolic
Q6AZ96	Src homology 3 domain-containing guanine nucleotide exchange factor.	Cytosolic
Q9NVM4	Protein arginine N-methyltransferase 7 (EC 2.1.1.-).	Cytosolic
P02649	Apolipoprotein E precursor (Apo-E).	Cytosolic
Q9UN86	[Ras GTPase-activating protein-binding protein 2 (G3BP-2) (GAP SH3, domain-binding protein 2).]	Cytosolic
Q15652	[Probable JmjC domain-containing histone demethylation protein 2C, (EC 1.14.11.-) (Jumonji domain-containing protein 1C) (Thyroid, receptor-interacting protein 8) (TRIP-8).]	Cytosolic
P51003	[Poly(A) polymerase alpha (EC 2.7.7.19) (PAP) (Polynucleotide, adenylyltransferase alpha).]	Cytosolic
Q66K31	TRMU protein.	Cytosolic
Q92766	[RAS-responsive element-binding protein 1 (RREB-1) (Raf-responsive zinc, finger protein LZ321) (Zinc finger motif-enhancer binding-protein 1), (Zep-1) (Finger protein in nuclear bodies).]	Cytosolic
Q93063	[Exostosin-2 (EC 2.4.1.224) (EC 2.4.1.225) (Glucuronosyl-N-, acetylglucosaminyl-proteoglycan/N-acetylglucosaminyl-proteoglycan 4-, alpha-N-acetylglucosaminyltransferase) (Putative tumor suppressor, protein EXT2) (Multiple exostoses protein 2).]	ER
Q86T76	Putative uncharacterized protein DKFZp451O0317.	ER
P20774	Mimecan precursor (Osteoglycin) (Osteoinductive factor) (OIF).	ER
Q8TEQ8	[GPI ethanolamine phosphate transferase 3 (EC 2.-.-.-), (Phosphatidylinositol-glycan biosynthesis class O protein) (PIG-O).]	ER
P11511	[Cytochrome P450 19A1 (EC 1.14.14.1) (Aromatase) (CYPXIX) (Estrogen, synthetase) (P-450AROM).]	ER
P02763	Alpha-1-acid glycoprotein 1 precursor (AGP 1) (Orosomucoid-1) (OMD 1).	Extracellular
P02647	[Apolipoprotein A-I precursor (Apo-AI) (ApoA-I) [Contains:, Apolipoprotein A-I(1-242)].]	Extracellular
P01042	[Kininogen-1 precursor (Alpha-2-thiol proteinase inhibitor) [Contains:, Kininogen-1 heavy chain; Bradykinin (Kallidin I); Lysyl-bradykinin, (Kallidin II); Kininogen-1 light chain; Low molecular weight growth-, promoting factor].]	Extracellular
P35030	[Trypsin-3 precursor (EC 3.4.21.4) (Trypsin III) (Brain trypsinogen), (Mesotrypsinogen) (Trypsin IV) (Serine protease 3) (Serine protease, 4).]	Extracellular

P01009	[Alpha-1-antitrypsin precursor (Alpha-1 protease inhibitor) (Alpha-1-, antiproteinase).]	Extracellular
Q99435	[Protein kinase C-binding protein NELL2 precursor (NEL-like protein 2), (Nel-related protein 2).]	Extracellular
P02774	[Vitamin D-binding protein precursor (DBP) (Group-specific component), (Gc-globulin) (VDB).]	Extracellular
P02790	Hemopexin precursor (Beta-1B-glycoprotein).	Extracellular
P01620	Ig kappa chain V-III region SIE.	Extracellular
P10451	[Osteopontin precursor (Bone sialoprotein 1) (Secreted phosphoprotein, 1) (SPP-1) (Urinary stone protein) (Nephropontin) (Uropontin).]	Extracellular
P09486	[SPARC precursor (Secreted protein acidic and rich in cysteine), (Osteonectin) (ON) (Basement-membrane protein 40) (BM-40).]	Extracellular
P35443	Thrombospondin-4 precursor.	Extracellular
P04004	[Vitronectin precursor (Serum-spreading factor) (S-protein) (V75), [Contains: Vitronectin V65 subunit; Vitronectin V10 subunit;, Somatomedin-B].]	Extracellular
P08603	Complement factor H precursor (H factor 1).	Extracellular
P10909	[Clusterin precursor (Complement-associated protein SP-40,40), (Complement cytolysis inhibitor) (CLI) (NA1/NA2) (Apolipoprotein J), (Apo-J) (Testosterone-repressed prostate message 2) (TRPM-2), [Contains: Clusterin beta chain (ApoJalpha) (Complement cytolysis, inhibitor a chain); Clusterin alpha chain (ApoJbeta) (Complement, cytolysis inhibitor b chain)].]	Extracellular
P04196	[Histidine-rich glycoprotein precursor (Histidine-proline-rich, glycoprotein) (HPRG).]	Extracellular
P05452	[Tetranectin precursor (TN) (C-type lectin domain family 3 member B), (Plasminogen kringle 4-binding protein).]	Extracellular
P02766	Transthyretin precursor (Prealbumin) (TBPA) (TTR) (ATTR).	Extracellular
P43652	Afamin precursor (Alpha-albumin) (Alpha-Alb).	Extracellular
Q9UBP4	Dickkopf-related protein 3 precursor (Dkk-3) (Dickkopf-3) (hDkk-3).	Extracellular
P55058	Phospholipid transfer protein precursor (Lipid transfer protein II).	Extracellular
P04217	Alpha-1B-glycoprotein precursor (Alpha-1-B glycoprotein).	Extracellular
P00738	[Haptoglobin precursor [Contains: Haptoglobin alpha chain; Haptoglobin, beta chain].]	Extracellular
P02765	[Alpha-2-HS-glycoprotein precursor (Fetuin-A) (Alpha-2-Z-globulin) (Ba-, alpha-2-glycoprotein) [Contains: Alpha-2-HS-glycoprotein chain A;, Alpha-2-HS-glycoprotein chain B].]	Extracellular
P41222	[Prostaglandin-H2 D-isomerase precursor (EC 5.3.99.2) (Lipocalin-type, prostaglandin-D synthase) (Glutathione-independent PGD synthetase), (Prostaglandin-D2 synthase) (PGD2 synthase) (PGDS2) (PGDS) (Beta-trace, protein) (Cerebrin-28).]	Extracellular
P00450	Ceruloplasmin precursor (EC 1.16.3.1) (Ferroxidase).	Extracellular
O00584	Ribonuclease T2 precursor (EC 3.1.27.-) (Ribonuclease 6).	Extracellular
Q92520	Protein FAM3C precursor (Protein GS3786).	Extracellular
P13521	[Secretogranin-2 precursor (Secretogranin II) (SgII) (Chromogranin-C), [Contains: Secretoneurin (SN)].]	Extracellular
P02768	Serum albumin precursor.	Extracellular
Q8IUK7	ALB protein.	Extracellular
P01011	[Alpha-1-antichymotrypsin precursor (ACT) (Cell growth-inhibiting gene, 24/25 protein) [Contains: Alpha-1-antichymotrypsin His-Pro-less].]	Extracellular
P06727	Apolipoprotein A-IV precursor (Apo-AIV) (ApoA-IV).	Extracellular
P07477	[Trypsin-1 precursor (EC 3.4.21.4) (Trypsin I) (Cationic trypsinogen), (Serine protease 1).]	Extracellular
P01023	Alpha-2-macroglobulin precursor (Alpha-2-M).	Extracellular

P05155	[Plasma protease C1 inhibitor precursor (C1 Inh) (C1Inh) (C1 esterase, inhibitor) (C1-inhibiting factor).]	Extracellular
Q9UHG2	[ProSAAS precursor (pro-SAAS) (Proprotein convertase subtilisin/kexin, type 1 inhibitor) (Proprotein convertase 1 inhibitor) [Contains: KEP;, Big SAAS (b-SAAS); Little SAAS (l-SAAS) (N-proSAAS); Big PEN-LEN (b-, PEN-LEN) (SAAS CT(1-49)); PEN; Little LEN (l-LEN); Big LEN (b-LEN), (SAAS CT(25-40))].]	Extracellular
Q5JNX2	Complement component 4A (Rodgers blood group).	Extracellular
P36955	[Pigment epithelium-derived factor precursor (PEDF) (Serpin-F1) (EPC-, 1).]	Extracellular
P01019	[Angiotensinogen precursor (Serpin A8) [Contains: Angiotensin-1, (Angiotensin I) (Ang I); Angiotensin-2 (Angiotensin II) (Ang II);, Angiotensin-3 (Angiotensin III) (Ang III) (Des-Asp[1]-angiotensin, II)].]	Extracellular
P23142	Fibulin-1 precursor.	Extracellular
Q9NRM1	Enamelin precursor.	Extracellular
P05090	Apolipoprotein D precursor (Apo-D) (ApoD).	Extracellular
P02652	[Apolipoprotein A-II precursor (Apo-AII) (ApoA-II) [Contains:, Apolipoprotein A-II(1-76)].]	Extracellular
Q9Y272	[Dexamethasone-induced Ras-related protein 1 precursor (Activator of G-, protein signaling 1).]	Go Component not found
P61769	[Beta-2-microglobulin precursor [Contains: Beta-2-microglobulin form pI, 5.3].]	Go Component not found
O14867	[Transcription regulator protein BACH1 (BTB and CNC homolog 1), (HA2303).]	Go Component not found
P01842	Ig lambda chain C regions.	Go Component not found
O15479	[Melanoma-associated antigen B2 (MAGE-B2 antigen) (DSS-AHC critical, interval MAGE superfamily 6) (DAM6) (MAGE XP-2) (Cancer/testis antigen, 3.2) (CT3.2).]	Go Component not found
P00558	[Phosphoglycerate kinase 1 (EC 2.7.2.3) (Primer recognition protein 2), (PRP 2) (Cell migration-inducing gene 10 protein).]	Go Component not found
P35813	[Protein phosphatase 1A (EC 3.1.3.16) (Protein phosphatase 2C isoform, alpha) (PP2C-alpha) (IA).]	Go Component not found
P45378	[Troponin T, fast skeletal muscle (TnTf) (Fast skeletal muscle troponin, T) (fTnT) (Beta TnTF).]	Go Component not found
Q6NZY4	Zinc finger CCHC domain-containing protein 8.	Go Component not found
Q58FF3	Heat shock protein 94b.	Go Component not found
Q8TDL7	Spermatogenesis associated factor.	Go Component not found
Q96RK0	Protein capicua homolog.	Go Component not found
Q13619	Cullin-4A (CUL-4A).	Go Component not found
P42679	[Megakaryocyte-associated tyrosine-protein kinase (EC 2.7.10.2), (Tyrosine-protein kinase CTK) (Protein kinase HYL) (Hematopoietic, consensus tyrosine-lacking kinase).]	Go Component not found
P98066	[Tumor necrosis factor-inducible protein TSG-6 precursor (TNF-, stimulated gene 6 protein) (Tumor necrosis factor, alpha-induced, protein 6) (Hyaluronate-binding protein).]	Go Component not found
P52735	Protein vav-2.	Go Component not found
Q8N720	Zinc finger protein 655 (Vav-interacting Krueppel-like protein).	Go Component not found
Q8N1V2	[WD repeat-containing protein 16 (WD40-repeat protein up-regulated in, HCC).]	Go Component not found
Q5JUW7	[Discs, large homolog 3 (Neuroendocrine-dlg, Drosophila) (Discs, large, homolog 3 (Neuroendocrine-dlg, Drosophila), isoform CRA_d).]	Go Component not found
Q5PRF9	Sterile alpha motif domain-containing protein 4B.	Go Component not found
Q5VZK9	Leucine-rich repeat-containing protein 16.	Go Component not found
Q8N8A2	Ankyrin repeat domain-containing protein 44.	Go Component not found

Q9BWU1	[Cell division cycle 2-like protein kinase 6 (EC 2.7.11.22) (CDC2-, related protein kinase 6) (Death-preventing kinase) (Cyclin-dependent, kinase 11).]	Go Component not found
P00751	[Complement factor B precursor (EC 3.4.21.47) (C3/C5 convertase), (Properdin factor B) (Glycine-rich beta glycoprotein) (GBG) (PBF2), [Contains: Complement factor B Ba fragment; Complement factor B Bb, fragment].]	Go Component not found
Q15418	[Ribosomal protein S6 kinase alpha-1 (EC 2.7.11.1) (S6K-alpha 1) (90, kDa ribosomal protein S6 kinase 1) (p90-RSK 1) (Ribosomal S6 kinase 1), (RSK-1) (pp90RSK1) (p90S6K) (MAP kinase-activated protein kinase 1a), (MAPKAPK1A).]	Go Component not found
Q14134	[Tripartite motif-containing protein 29 (Ataxia-telangiectasia group D-, associated protein).]	Go Component not found
Q5JPN5	LON peptidase N-terminal domain and ring finger 3 (Fragment).	Go Component not found
Q9HBH9	[MAP kinase-interacting serine/threonine-protein kinase 2 (EC 2.7.11.1), (MAP kinase signal-integrating kinase 2) (Mnk2).]	Go Component not found
Q6NUP7	KIAA1622 (KIAA1622, isoform CRA_a).	Go Component not found
Q6ZTD5	[CDNA FLJ44762 fis, clone BRACE3031743, moderately similar to Homo, sapiens bruno-like 4, RNA binding protein.]	Go Component not found
Q96BE9	CDK4 protein (Cyclin-dependent kinase 4, isoform CRA_c).	Go Component not found
Q9Y5L5	Lens epithelial cell protein LEP503.	Go Component not found
O00172	Line-1 reverse transcriptase (Fragment).	Go Component not found
Q5VZL5	Zinc finger MYM-type protein 4 (Zinc finger protein 262).	Go Component not found
Q9BXJ4	[Complement C1q tumor necrosis factor-related protein 3 precursor, (Secretory protein CORS26).]	Go Component not found
O60543	[Cell death activator CIDE-A (Cell death-inducing DFFA-like effector, A).]	Go Component not found
P0C0L4	[Complement C4-A precursor (Acidic complement C4) [Contains: Complement, C4 beta chain; Complement C4-A alpha chain; C4a anaphylatoxin; C4b-A;, C4d-A; Complement C4 gamma chain].]	Go Component not found
Q9NVD3	SET domain-containing protein 4.	Go Component not found
Q9UII4	[Probable E3 ubiquitin-protein ligase HERC5 (EC 6.3.2.-) (HECT domain, and RCC1-like domain-containing protein 5) (Cyclin-E-binding protein, 1).]	Go Component not found
Q96PQ7	Kelch-like protein 5.	Go Component not found
O14513	Nck-associated protein 5 (NAP-5) (Peripheral clock protein).	Go Component not found
Q7RTW8	Otoancorin precursor.	Go Component not found
Q9Y365	[PCTP-like protein (PCTP-L) (StAR-related lipid transfer protein 10), (StARD10) (START domain-containing protein 10) (Serologically defined, colon cancer antigen 28) (Antigen NY-CO-28).]	Go Component not found
Q96EP0	[RING finger protein 31 (Zinc in-between-RING-finger ubiquitin-, associated domain protein).]	Go Component not found
Q9NY91	[Low affinity sodium-glucose cotransporter (Sodium/glucose, cotransporter 3) (Na(+)/glucose cotransporter 3) (Solute carrier, family 5 member 4).]	Go Component not found
Q13242	[Splicing factor, arginine/serine-rich 9 (Pre-mRNA-splicing factor, SRp30C).]	Go Component not found
Q9UHJ3	Scm-like with four MBT domains protein 1 (Renal ubiquitous protein 1).	Go Component not found
Q14515	[SPARC-like protein 1 precursor (High endothelial venule protein), (Hevin) (MAST 9).]	Go Component not found
P59817	[Suppressor of hairy wing homolog 1 (3'OY11.1) (Zinc finger protein, 280).]	Go Component not found
Q9Y6A5	Transforming acidic coiled-coil-containing protein 3 (ERIC-1).	Go Component not found
Q8TBP0	TBC1 domain family member 16.	Go Component not found
Q9UPW8	Protein unc-13 homolog A (Munc13-1).	Go Component not found
O95498	[Vascular non-inflammatory molecule 2 precursor (Vanin-2), (Glycosylphosphatidyl inositol-anchored protein GPI-80) (Protein FOAP-, 4).]	Go Component not found

P62699	Protein yippee-like 5.	Go Component not found
Q9NYT6	Zinc finger protein 226.	Go Component not found
Q8N8Z8	Zinc finger protein 441.	Go Component not found
P60852	[Zona pellucida sperm-binding protein 1 precursor (Zona pellucida, glycoprotein 1) (Zp-1) [Contains: Processed zona pellucida sperm-, binding protein 1].]	Go Component not found
O15014	Zinc finger protein 609.	Go Component not found
Q7Z398	Zinc finger protein 550.	Go Component not found
Q9BQC3	[Diphthamide biosynthesis protein 2 (DPH2 homolog) (HsDph2), (Diphthamide biosynthesis protein 2 homolog-like 2) (DPH2-like 2).]	Go Component not found
O75199	Putative uncharacterized protein A-761H5.4.	Go Component not found
Q53EV4	Leucine-rich repeat-containing protein 23 (Leucine-rich protein B7).	Go Component not found
Q5HYI6	Putative uncharacterized protein DKFZp313O2236 (Fragment).	Go Component not found
Q8NEY1	[Neuron navigator 1 (Steerin-1) (Pore membrane and/or filament-, interacting-like protein 3) (Unc-53 homolog 1) (unc53H1).]	Go Component not found
Q5T447	[Probable E3 ubiquitin-protein ligase HECTD3 (HECT domain-containing, protein 3).]	Go Component not found
Q5T7N3	Ankyrin repeat domain-containing protein 38.	Go Component not found
Q5TGZ2	[Neuroblastoma, suppression of tumorigenicity 1 (Neuroblastoma,, suppression of tumorigenicity 1, isoform CRA_a).]	Go Component not found
Q5VWN6	Uncharacterized protein C10orf18.	Go Component not found
Q68CN4	Putative uncharacterized protein DKFZp686E23209.	Go Component not found
Q9P2P5	[E3 ubiquitin-protein ligase HECW2 (EC 6.3.2.-) (HECT, C2 and WW, domain-containing protein 2) (NEDD4-like E3 ubiquitin-protein ligase, 2).]	Go Component not found
Q6IPR3	tRNA wybutosine-synthesizing protein 3 homolog (EC 2.1.1.-).	Go Component not found
Q6MZV6	Putative uncharacterized protein DKFZp686L19235.	Go Component not found
Q6N095	Putative uncharacterized protein DKFZp686K03196.	Go Component not found
Q6P5U3	CMYA5 protein (Fragment).	Go Component not found
Q6PIU2	Arylacetamide deacetylase-like 1 (EC 3.1.1.-).	Go Component not found
Q6UW49	[Sperm equatorial segment protein 1 precursor (SP-ESP) (Equatorial, segment protein) (ESP) (Glycosylated 38 kDa sperm protein C-7/8).]	Go Component not found
Q6ZN84	Coiled-coil domain-containing protein 81.	Go Component not found
Q6ZNY1	CDNA FLJ26894 fis, clone RCT00440.	Go Component not found
Q6ZRF7	Zinc finger protein 818.	Go Component not found
Q6ZSA8	CDNA FLJ45684 fis, clone FCBBF3005160.	Go Component not found
Q6ZTG8	CDNA FLJ44670 fis, clone BRACE3005903.	Go Component not found
Q6ZUT9	CDNA FLJ43333 fis, clone NT2RI3006376.	Go Component not found
Q70CQ4	[Ubiquitin carboxyl-terminal hydrolase 31 (EC 3.1.2.15) (Ubiquitin, thioesterase 31) (Ubiquitin-specific-processing protease 31), (Deubiquitinating enzyme 31).]	Go Component not found
Q9H2L5	Ras association domain-containing protein 4.	Go Component not found
Q149N8	[E3 ubiquitin-protein ligase SHPRH (EC 6.3.2.-) (EC 3.6.1.-) (SNF2,, histone-linker, PHD and RING finger domain-containing helicase).]	Go Component not found
Q8IZC4	Pleckstrin homology domain-containing family K member 1 (Rhotekin-2).	Go Component not found

Q8N1K5	Uncharacterized protein C6orf190.	Go Component not found
Q96MF7	[E3 SUMO-protein ligase NSE2 (EC 6.-.-.-) (Non-structural maintenance, of chromosomes element 2 homolog) (Non-SMC element 2 homolog), (hMMS21).]	Go Component not found
Q8N7W6	CDNA FLJ40265 fis, clone TESTI2026291.	Go Component not found
Q8NAA3	CDNA FLJ35700 fis, clone SPLEN2019985.	Go Component not found
Q8NAT4	CDNA FLJ34815 fis, clone NT2NE2007786.	Go Component not found
Q8NBZ9	Putative uncharacterized protein C1orf118.	Go Component not found
Q8TD25	Seizure 6 homolog.	Go Component not found
Q96BZ3	HAGHL protein (Hydroxyacylglutathione hydrolase-like, isoform CRA_h).	Go Component not found
Q96JI6	KIAA1841 protein (Fragment).	Go Component not found
Q9BTE6	Alanyl-tRNA synthetase domain-containing protein 1.	Go Component not found
Q9H3S5	[GPI mannosyltransferase 1 (EC 2.4.1.-) (GPI mannosyltransferase I), (GPI-MT-I) (Phosphatidylinositol-glycan biosynthesis class M protein), (PIG-M).]	Go Component not found
Q86TU7	SET domain-containing protein 3.	Go Component not found
Q9H8H0	Nucleolar protein 11.	Go Component not found
Q9HD47	MOG1 isoform A (RAN guanine nucleotide release factor, isoform CRA_a).	Go Component not found
Q9NUJ7	[Phosphatidylinositol-specific phospholipase C X domain-containing, protein 1 (PI-PLC X domain-containing protein 1).]	Go Component not found
P59510	[ADAMTS-20 precursor (EC 3.4.24.-) (A disintegrin and metalloproteinase, with thrombospondin motifs 20) (ADAM-TS 20) (ADAM-TS20).]	Go Component not found
Q9H2I8	Uncharacterized protein C10orf11.	Go Component not found
P10645	[Chromogranin-A precursor (CgA) (Pituitary secretory protein I) (SP-I), [Contains: Vasostatin-1 (Vasostatin I); Vasostatin-2 (Vasostatin II);, EA-92; ES-43; Pancreastatin; SS-18; WA-8; WE-14; LF-19; AL-11; GV-19;, GR-44; ER-37].]	Go Component not found
P0C0L5	[Complement C4-B precursor (Basic complement C4) [Contains: Complement, C4 beta chain; Complement C4-B alpha chain; C4a anaphylatoxin; C4b-B;, C4d-B; Complement C4 gamma chain].]	Go Component not found
Q96KN2	[Beta-Ala-His dipeptidase precursor (EC 3.4.13.20) (Carnosine, dipeptidase 1) (CNDP dipeptidase 1) (Serum carnosinase) (Glutamate, carboxypeptidase-like protein 2).]	Go Component not found
O94985	Calsyntenin-1 precursor.	Go Component not found
Q9HCS5	Band 4.1-like protein 4A (Protein NBL4).	Go Component not found
Q8TBR7	Protein FAM57A (CT120 protein).	Go Component not found
Q5T200	Zinc finger CCCH domain-containing protein 13.	Go Component not found
Q9P2N7	Kelch-like protein 13 (BTB and kelch domain-containing protein 2).	Go Component not found
Q96PG2	Membrane-spanning 4-domains subfamily A member 10.	Go Component not found
Q8NEM0	Microcephalin.	Go Component not found
Q8NC67	[Neuropilin and tolloid-like protein 2 precursor (Brain-specific, transmembrane protein containing 2 CUB and 1 LDL-receptor class A, domains protein 2).]	Go Component not found
Q9P121	Neurotrimin precursor (hNT).	Go Component not found
Q9H668	Oligonucleotide/oligosaccharide-binding fold-containing protein 1.	Go Component not found
Q9UGF6	Olfactory receptor 5V1 (Olfactory receptor OR6-26) (Hs6M1-21).	Go Component not found
P16499	[Rod cGMP-specific 3',5'-cyclic phosphodiesterase subunit alpha, (EC 3.1.4.35) (GMP-PDE alpha) (PDE V-B1).]	Go Component not found

P33764	Protein S100-A3 (S100 calcium-binding protein A3) (Protein S-100E).	Go Component not found
Q8WVM8	[Sec1 family domain-containing protein 1 (Syntaxin-binding protein 1-, like 2) (Sly1p).]	Go Component not found
Q8WXD2	Secretogranin-3 precursor (Secretogranin III) (SgIII).	Go Component not found
Q9P2P6	[StAR-related lipid transfer protein 9 (StARD9) (START domain-, containing protein 9).]	Go Component not found
Q8NG68	Tubulin--tyrosine ligase (EC 6.3.2.25) (TTL).	Go Component not found
O00744	Protein Wnt-10b precursor (Wnt-12).	Go Component not found
Q9NUA8	Zinc finger and BTB domain-containing protein 40.	Go Component not found
Q14588	Zinc finger protein 234 (Zinc finger protein 4) (HZF4).	Go Component not found
O60687	Sushi repeat-containing protein SRPX2 precursor.	Go Component not found
Q8N370	[Large neutral amino acids transporter small subunit 4 (L-type amino L-, acid transporter 4) (Solute carrier family 43 member 2).]	Go Component not found
Q15818	Neuronal pentraxin-1 precursor (NP1) (Neuronal pentraxin I) (NP-I).	Go Component not found
Q5SYE7	NHS-like protein 1.	Go Component not found
Q5T4S7	[Zinc finger UBR1-type protein 1 (Retinoblastoma-associated factor of, 600 kDa) (600 kDa retinoblastoma protein-associated factor) (RBAF600), (p600).]	Go Component not found
Q5RHP9	Uncharacterized protein C1orf173.	Go Component not found
Q6DHV5	Putative uncharacterized protein C10orf130.	Go Component not found
Q6IQ55	Tau-tubulin kinase 2 (EC 2.7.11.1).	Go Component not found
Q6MZX7	Putative uncharacterized protein DKFZp686M24218.	Go Component not found
Q6NSJ5	Leucine-rich repeat-containing protein 8E.	Go Component not found
Q6PI47	BTB/POZ domain-containing protein KCTD18.	Go Component not found
Q6S9Z5	Zinc finger protein 474.	Go Component not found
Q6UXZ0	[Transmembrane and immunoglobulin domain-containing protein 1, precursor.]	Go Component not found
Q6ZMP0	Thrombospondin type-1 domain-containing protein 4 (Fragment).	Go Component not found
Q6ZRG3	CDNA FLJ46373 fis, clone TESTI4052132.	Go Component not found
Q9ULJ7	Ankyrin repeat domain-containing protein 50.	Go Component not found
Q4AC94	C2 domain-containing protein 3.	Go Component not found
Q86SE5	[RNA-binding Raly-like protein (hRALYL) (Heterogeneous nuclear, ribonucleoprotein C-like 3) (hnRNP core protein C-like 3).]	Go Component not found
Q86XE5	[Dihydrodipicolinate synthase-like, mitochondrial precursor, (EC 4.-.-.-) (DHDPS-like protein).]	Go Component not found
Q7LBC6	[JmjC domain-containing histone demethylation protein 2B (EC 1.14.11.-), (Jumonji domain-containing protein 1B) (Nuclear protein 5qNCA).]	Go Component not found
Q7Z6J2	[General receptor for phosphoinositides 1-associated scaffold protein, (GRP1-associated scaffold protein).]	Go Component not found
Q8TC20	[Cancer-associated gene 1 protein (CAGE-1) (Cancer/testis antigen 3), (CT3).]	Go Component not found
Q5T8A7	Protein KIAA0649.	Go Component not found
O43147	[Small G protein signaling modulator 2 (RUN and TBC1 domain-containing, protein 1).]	Go Component not found
Q4G0P3	Hydrocephalus-inducing protein homolog.	Go Component not found
Q8N4P6	Leucine-rich repeat-containing protein C10orf92.	Go Component not found
Q8N5U1	Putative uncharacterized protein MGC35295.	Go Component not found

Q8NB62	CDNA FLJ34189 fis, clone FCBBF3017535.	Go Component not found
Q8IVL1	[Neuron navigator 2 (EC 3.6.1.-) (Helicase APC down-regulated 1), (Retinoic acid inducible in neuroblastoma 1) (Steerin-2) (Pore, membrane and/or filament-interacting-like protein 2) (Unc-53 homolog, 2) (unc53H2).]	Go Component not found
Q8TER2	FLJ00131 protein (Fragment).	Go Component not found
Q969T7	Cytosolic 5'-nucleotidase III-like protein.	Go Component not found
Q96ET8	Protein FAM18B2.	Go Component not found
Q96RR1	[Twinkle protein, mitochondrial precursor (EC 3.6.1.-) (T7 gp4-like, protein with intramitochondrial nucleoid localization) (T7-like, mitochondrial DNA helicase) (Progressive external ophthalmoplegia 1, protein).]	Go Component not found
Q99993	WUGSC:H_2G3A.1 protein.	Go Component not found
Q9BTF7	Calcium binding and coiled-coil domain 2.	Go Component not found
Q9NYV6	[RNA polymerase I-specific transcription initiation factor RRN3, (Transcription initiation factor IA) (TIF-IA).]	Go Component not found
Q9H958	CDNA FLJ12990 fis, clone NT2RP3000092.	Go Component not found
Q9NWL6	[CDNA FLJ20752 fis, clone HEP02921 (Asparagine synthetase domain, containing 1, isoform CRA_a) (Asparagine synthetase domain containing, 1) (HCV NS3-transactivated protein 1).]	Go Component not found
Q8TCC3	39S ribosomal protein L30, mitochondrial precursor (L30mt) (MRP-L30).	Go Component not found
Q6MZW2	Follistatin-related protein 4 precursor (Follistatin-like 4).	Go Component not found
P62736	[Actin, aortic smooth muscle (Alpha-actin-2) (Cell growth-inhibiting, gene 46 protein).]	Go Component not found
P61109	Kidney androgen-regulated protein precursor (KAP) (ARP).	Go Component not found
Q8WXK4	Ankyrin repeat and SOCS box protein 12 (ASB-12).	Go Component not found
Q8WXX7	Autism susceptibility gene 2 protein.	Go Component not found
P34820	[Bone morphogenetic protein 8B precursor (BMP-8B) (BMP-8) (Osteogenic, protein 2) (OP-2).]	Go Component not found
Q9P209	Centrosomal protein of 72 kDa (Cep72 protein).	Go Component not found
Q5VXU9	Uncharacterized protein C9orf84.	Go Component not found
Q96M20	Uncharacterized protein C20orf152.	Go Component not found
Q8WUP2	[Filamin-binding LIM protein 1 (FBLP-1) (Mitogen-inducible 2-, interacting protein) (MIG2-interacting protein) (Migfilin).]	Go Component not found
P18065	[Insulin-like growth factor-binding protein 2 precursor (IGFBP-2) (IBP-, 2) (IGF-binding protein 2).]	Go Component not found
Q6TDP4	Kelch-like protein 17 (Actinfilin).	Go Component not found
Q13449	Limbic system-associated membrane protein precursor (LSAMP).	Go Component not found
Q9H4F8	[SPARC-related modular calcium-binding protein 1 precursor (Secreted, modular calcium-binding protein 1) (SMOC-1).]	Go Component not found
Q07617	[Sperm-associated antigen 1 (Infertility-related sperm protein Spag-1), (HSD-3.8).]	Go Component not found
Q7Z410	[Transmembrane protease, serine 9 (EC 3.4.21.-) (Polyserase-1), (Polyserase-I) (Polyserine protease 1) [Contains: Serase-1; Serase-2;, Serase-3].]	Go Component not found
Q8IZJ1	[Netrin receptor UNC5B precursor (Protein unc-5 homolog B) (Unc-5, homolog 2) (p53-regulated receptor for death and life protein 1).]	Go Component not found
Q5GH73	XK-related protein 6.	Go Component not found
Q7Z2W4	[Zinc finger CCCH type antiviral protein 1 (Zinc finger CCCH domain-, containing protein 2).]	Go Component not found
Q9UK10	Zinc finger protein 225.	Go Component not found

Q9BY12	[S phase cyclin A-associated protein in the ER (Zinc finger protein, 291).]	Go Component not found
O43532	RIG-like 7-1.	Go Component not found
Q5CZ94	Putative uncharacterized protein DKFZp781M0386.	Go Component not found
Q5EFE6	Anti-RhD monoclonal T125 kappa light chain precursor.	Go Component not found
Q7Z2Y5	Nik-related protein kinase (EC 2.7.11.1).	Go Component not found
Q5JYT8	Novel protein (Fragment).	Go Component not found
Q5T0D9	Protein FAM79A.	Go Component not found
Q5TF21	Uncharacterized protein C6orf174 precursor.	Go Component not found
Q5VVM6	Novel protein.	Go Component not found
A0AV02	Solute carrier family 12 member 8 (Cation-chloride cotransporter 9).	Go Component not found
Q9Y4C1	[JmjC domain-containing histone demethylation protein 2A (EC 1.14.11.-), (Jumonji domain-containing protein 1A).]	Go Component not found
Q8NGI3	Olfactory receptor 56B1 (Olfactory receptor OR11-65).	Go Component not found
Q6MZQ6	Putative uncharacterized protein DKFZp686G11190.	Go Component not found
Q6N030	Putative uncharacterized protein DKFZp686I15212.	Go Component not found
Q6P280	Zinc finger protein 529.	Go Component not found
Q6PIU1	[Potassium voltage-gated channel subfamily V member 1 (Voltage-gated, potassium channel subunit Kv8.1) (Neuronal potassium channel alpha, subunit HNKA).]	Go Component not found
Q6TDU7	Lung adenoma susceptibility 1-like protein (CASC1 protein).	Go Component not found
Q6ZMX3	CDNA FLJ16614 fis, clone TESTI4013365.	Go Component not found
Q6ZNQ8	CDNA FLJ27323 fis, clone TMS07977.	Go Component not found
Q6ZRT8	[CDNA FLJ46113 fis, clone TESTI2036285, highly similar to Rattus, norvegicus ubiquitin C (Ubc).]	Go Component not found
Q7Z4H8	KDEL motif-containing protein 2 precursor.	Go Component not found
Q6ZWJ8	Cysteine-rich BMP regulator 2.	Go Component not found
Q76B61	SCO-spondin homolog (Fragment).	Go Component not found
A4D1E1	Zinc finger protein 804B.	Go Component not found
Q86TP7	LAMB4 protein (Fragment).	Go Component not found
Q86Y25	[Zinc finger protein 354C (Kidney, ischemia, and developmentally-, regulated protein 3) (hKID3).]	Go Component not found
O14526	FCH domain only protein 1.	Go Component not found
Q8IZ40	REST corepressor 2.	Go Component not found
Q8N0V7	CDNA FLJ40969 fis, clone UTERU2012688 (CDNA FLJ40984 fis).	Go Component not found
Q8N4V6	TMEM181 protein (Fragment).	Go Component not found
Q8N7Q3	Zinc finger protein 676.	Go Component not found
Q8NAS2	CDNA FLJ34873 fis, clone NT2NE2014950.	Go Component not found
Q8NBX4	CDNA FLJ90687 fis, clone PLACE1006093.	Go Component not found
Q8TCD6	Pyridoxal phosphate phosphatase PHOSPHO2 (EC 3.1.3.74).	Go Component not found
Q96J66	[ATP-binding cassette transporter sub-family C member 11 (Multidrug, resistance-associated protein 8).]	Go Component not found
Q8WY69	PP4534.	Go Component not found

Q96HQ4	DUSP3 protein.	Go Component not found
Q5TGI0	Uncharacterized protein C6orf168.	Go Component not found
Q86UV5	[Ubiquitin carboxyl-terminal hydrolase 48 (EC 3.1.2.15) (Ubiquitin, thioesterase 48) (Ubiquitin-specific-processing protease 48), (Deubiquitinating enzyme 48).]	Go Component not found
Q6P2H3	Coiled-coil domain-containing protein 21.	Go Component not found
Q9HBL8	NmrA-like family domain-containing protein 1.	Go Component not found
Q9NWS3	CDNA FLJ20639 fis, clone KAT02950.	Go Component not found
Q9UDW8	WUGSC:H_DJ0747G18.3 protein (VGF nerve growth factor inducible).	Go Component not found
Q9UKA4	[A-kinase anchor protein 11 (Protein kinase A-anchoring protein 11), (PRKA11) (A kinase anchor protein 220 kDa) (AKAP 220) (hAKAP220).]	Go Component not found
P52824	[Diacylglycerol kinase theta (EC 2.7.1.107) (Diglyceride kinase theta), (DGK-theta) (DAG kinase theta).]	Go Component not found
Q9NS73	[MAP3K12-binding inhibitory protein 1 (MAPK upstream kinase-binding, inhibitory protein) (MUK-binding inhibitory protein).]	Go Component not found
P45983	[Mitogen-activated protein kinase 8 (EC 2.7.11.24) (Stress-activated, protein kinase JNK1) (c-Jun N-terminal kinase 1) (JNK-46).]	Go Component not found
Q99886	Receptor tyrosine kinase (Fragment).	Go Component not found
P05129	Protein kinase C gamma type (EC 2.7.11.13) (PKC-gamma).	Go Component not found
P42680	Tyrosine-protein kinase Tec (EC 2.7.10.2).	Go Component not found
Q96MA6	Putative adenylate kinase-like protein C9orf98 (EC 2.7.4.3).	Go Component not found
Q13237	[cGMP-dependent protein kinase 2 (EC 2.7.11.12) (CGK 2) (cGKII) (Type, II cGMP-dependent protein kinase).]	Go Component not found
Q68DD2	[Cytosolic phospholipase A2 zeta (EC 3.1.1.4) (cPLA2-zeta), (Phospholipase A2 group IVF).]	Go Component not found
Q96AD5	[Patatin-like phospholipase domain-containing protein 2 (EC 3.1.1.3), (Adipose triglyceride lipase) (Desnutrin) (Transport-secretion protein, 2) (TTS2) (TTS2.2) (Calcium-independent phospholipase A2) (IPLA2-zeta), (Pigment epithelium-derived factor).]	Go Component not found
Q06278	Aldehyde oxidase (EC 1.2.3.1).	Go Component not found
Q9HB55	Cytochrome P450 3A43 (EC 1.14.14.1).	Go Component not found
P19623	[Spermidine synthase (EC 2.5.1.16) (Putrescine aminopropyltransferase), (SPDSY).]	Go Component not found
Q5JS39	Tubulin tyrosine ligase-like family, member 11.	Go Component not found
Q5KTR3	Flavoprotein oxidoreductase.	Go Component not found
P04075	[Fructose-bisphosphate aldolase A (EC 4.1.2.13) (Muscle-type aldolase), (Lung cancer antigen NY-LU-1).]	Go Component not found
Q93088	Betaine--homocysteine S-methyltransferase 1 (EC 2.1.1.5).	Go Component not found
Q5TD12	[Serine palmitoyltransferase, long chain base subunit 2-like, (Aminotransferase 2) (Fragment).]	Go Component not found
Q9Y2P4	[Long-chain fatty acid transport protein 6 (Fatty acid transport, protein 6) (FATP-6) (Very long-chain acyl-CoA synthetase homolog 1), (VLCSH1) (hVLCS-H1) (Fatty-acid-coenzyme A ligase, very long-chain 2), (Solute carrier family 27 member 6).]	Go Component not found
Q9C0C9	[Ubiquitin-conjugating enzyme E2 O (EC 6.3.2.19) (Ubiquitin-protein, ligase O) (Ubiquitin carrier protein O) (Ubiquitin-conjugating enzyme, E2 of 230 kDa) (E2-230K).]	Go Component not found
P08319	[Alcohol dehydrogenase 4 (EC 1.1.1.1) (Alcohol dehydrogenase class II, pi chain).]	Go Component not found
Q9Y600	[Cysteine sulfinic acid decarboxylase (EC 4.1.1.29) (Sulfinoalanine, decarboxylase) (Cysteine-sulfinate decarboxylase).]	Go Component not found
Q5T013	[Putative hydroxypyruvate isomerase (EC 5.3.1.22) (Endothelial cell, apoptosis protein E-CE1).]	Go Component not found
Q9Y302	[Protein GPR89 (Putative MAPK-activating protein PM01) (Putative NF-, kappa-B-activating protein 90).]	Go Component not found
Q9H3M0	[Potassium voltage-gated channel subfamily F member 1 (Voltage-gated, potassium channel subunit Kv5.1) (kH1).]	Go Component not found

Q8NFN6	RGS3 isoform RGS3S.	Go Component not found
P53675	Clathrin heavy chain 2 (CLH-22).	Go Component not found
Q93034	[Cullin-5 (CUL-5) (Vasopressin-activated calcium-mobilizing receptor), (VACM-1).]	Go Component not found
Q9UKY4	[Protein O-mannosyl-transferase 2 (EC 2.4.1.109) (Dolichyl-phosphate-, mannose--protein mannosyltransferase 2).]	Go Component not found
P05060	[Secretogranin-1 precursor (Secretogranin I) (SgI) (Chromogranin-B), (CgB) [Contains: GAWK peptide; CCB peptide].]	Go Component not found
Q14524	[Sodium channel protein type 5 subunit alpha (Sodium channel protein, type V subunit alpha) (Voltage-gated sodium channel subunit alpha, Nav1.5) (Sodium channel protein cardiac muscle subunit alpha) (HH1).]	Go Component not found
O15240	Neurosecretory protein VGF precursor.	Go Component not found
Q15678	[Tyrosine-protein phosphatase non-receptor type 14 (EC 3.1.3.48), (Protein-tyrosine phosphatase pez).]	Go Component not found
Q9NRR6	[72 kDa inositol polyphosphate 5-phosphatase (EC 3.1.3.36), (Phosphatidylinositol-4,5-bisphosphate 5-phosphatase), (Phosphatidylinositol polyphosphate 5-phosphatase type IV).]	Go Component not found
P01034	[Cystatin-C precursor (Cystatin-3) (Neuroendocrine basic polypeptide), (Gamma-trace) (Post-gamma-globulin).]	Go Component not found
Q6HA08	[Astacin-like metalloendopeptidase precursor (EC 3.4.-.-) (Oocyte, astacin) (Ovastacin).]	Go Component not found
Q7KZ97	Antithrombin III variant.	Go Component not found
Q9UBX7	[Kallikrein-11 precursor (EC 3.4.21.-) (hK11) (Hippostasin) (Trypsin-, like protease) (Serine protease 20) [Contains: Kallikrein-11 inactive, chain 1; Kallikrein-11 inactive chain 2].]	Go Component not found
Q8IZJ3	C3 and PZP-like alpha-2-macroglobulin domain-containing protein 8.	Go Component not found
P01024	[Complement C3 precursor [Contains: Complement C3 beta chain;, Complement C3 alpha chain; C3a anaphylatoxin; Complement C3b alpha', chain; Complement C3c alpha' chain fragment 1; Complement C3dg, fragment; Complement C3g fragment; Complement C3d fragment; Complement, C3f fragment; Complement C3c alpha' chain fragment 2].]	Go Component not found
Q9NZT2	[Opioid growth factor receptor (OGFr) (Zeta-type opioid receptor) (7-60, protein).]	Go Component not found
Q15247	33.1 kDa protein.	Go Component not found
O15309	Endogenous retroviral sequence K(C4) 2 (Fragment).	Go Component not found
Q6ZPD6	CDNA FLJ25992 fis, clone DMC03508.	Go Component not found
Q9UN79	[SOX-13 protein (Type 1 diabetes autoantigen ICA12) (Islet cell antigen, 12).]	Go Component not found
Q12986	[Transcriptional repressor NF-X1 (EC 6.3.2.-) (Nuclear transcription, factor, X box-binding, 1).]	Go Component not found
Q8N950	CDNA FLJ38364 fis, clone FEBRA2000909 (HCG1978990).	Go Component not found
P78381	[UDP-galactose translocator (UDP-galactose transporter) (UGT) (UDP-Gal-, Tr) (Solute carrier family 35 member A2).]	Go Component not found
P02751	[Fibronectin precursor (FN) (Cold-insoluble globulin) (CIG) [Contains:, Ugl-Y1; Ugl-Y2; Ugl-Y3].]	Golgi
Q7Z3U7	Protein MON2 homolog (Protein SF21).	Golgi
Q9UBV7	[Beta-1,4-galactosyltransferase 7 (EC 2.4.1.-) (Beta-1,4-GalTase 7), (Beta4Gal-T7) (b4Gal-T7) (UDP-galactose:beta-N-acetylglucosamine beta-, 1,4-galactosyltransferase 7) (UDP-Gal:beta-GlcNAc beta-1,4-, galactosyltransferase 7) [Includes: Xylosylprotein 4-beta-, galactosyltransferase (EC 2.4.1.133) (UDP-galactose:beta-xylose beta-, 1,4-galactosyltransferase) (Xylosylprotein beta-1,4-, galactosyltransferase) (XGPT) (Proteoglycan UDP-galactose:beta-xylose, beta1,4-galactosyltransferase I) (XGalT-1)].]	Golgi
O14795	Protein unc-13 homolog B (Munc13-2) (munc13).	Golgi
Q9Y6M9	[NADH dehydrogenase [ubiquinone] 1 beta subcomplex subunit 9, (EC 1.6.5.3) (EC 1.6.99.3) (NADH-ubiquinone oxidoreductase B22, subunit) (Complex I-B22) (CI-B22).]	Mitochondrial

P47985	[Cytochrome b-c1 complex subunit Rieske, mitochondrial precursor, (EC 1.10.2.2) (Ubiquinol-cytochrome c reductase iron-sulfur subunit), (Rieske iron-sulfur protein) (RISP) (Complex III subunit 5) [Contains:, Cytochrome b-c1 complex subunit 11 (Ubiquinol-cytochrome c reductase 8, kDa protein) (Complex III subunit IX)].]	Mitochondrial
Q969V5	[RING finger protein C1orf166 (Putative NF-kappa-B-activating protein, 266).]	Mitochondrial
Q9H936	[Mitochondrial glutamate carrier 1 (GC-1) (Glutamate/H(+) symporter 1), (Solute carrier family 25 member 22).]	Mitochondrial
P48547	[Potassium voltage-gated channel subfamily C member 1 (Voltage-gated, potassium channel subunit Kv3.1) (Kv4) (NGK2).]	Neural
O95180	[Voltage-dependent T-type calcium channel subunit alpha-1H (Voltage-, gated calcium channel subunit alpha Cav3.2) (Low-voltage-activated, calcium channel alpha1 3.2 subunit).]	Neural
Q9UNX9	[ATP-sensitive inward rectifier potassium channel 14 (Potassium, channel, inwardly rectifying subfamily J member 14) (Inward rectifier, K(+) channel Kir2.4) (IRK4).]	Neural
Q02641	[Voltage-dependent L-type calcium channel subunit beta-1 (CAB1), (Calcium channel voltage-dependent subunit beta 1).]	Neural
Q9H6Z4	Ran-binding protein 3 (RanBP3).	Nuclear
Q9UQ88	[PITSLRE serine/threonine-protein kinase CDC2L2 (EC 2.7.11.22), (Galactosyltransferase-associated protein kinase p58/GTA) (Cell, division cycle 2-like protein kinase 2) (CDK11).]	Nuclear
P38159	[Heterogeneous nuclear ribonucleoprotein G (hnRNP G) (RNA-binding motif, protein, X chromosome) (Glycoprotein p43) [Contains: Processed, heterogeneous nuclear ribonucleoprotein G].]	Nuclear
Q96ST3	[Paired amphipathic helix protein Sin3a (Transcriptional corepressor, Sin3a) (Histone deacetylase complex subunit Sin3a).]	Nuclear
Q68CN7	Putative uncharacterized protein DKFZp781I1252.	Nuclear
Q6ZNQ5	[CDNA FLJ27339 fis, clone TMS09615, highly similar to TFIIH basal, transcription factor complex helicase subunit (EC 3.6.1.-).]	Nuclear
Q6ZP55	[CDNA FLJ26482 fis, clone KDN05127 (Putative uncharacterized protein, DKFZp686G16228).]	Nuclear
Q9UIS9	[Methyl-CpG-binding domain protein 1 (Methyl-CpG-binding protein MBD1), (Protein containing methyl-CpG-binding domain 1).]	Nuclear
Q14980	Nuclear mitotic apparatus protein 1 (NuMA protein) (SP-H antigen).	Nuclear
P22626	Heterogeneous nuclear ribonucleoproteins A2/B1 (hnRNP A2 / hnRNP B1).	Nuclear
P55771	Paired box protein Pax-9.	Nuclear
P15822	[Zinc finger protein 40 (Human immunodeficiency virus type I enhancer-, binding protein 1) (HIV-EP1) (Major histocompatibility complex-binding, protein 1) (MBP-1) (Positive regulatory domain II-binding factor 1), (PRDII-BF1).]	Nuclear
Q86VE0	Putative uncharacterized protein LOC339344.	Nuclear
Q96SI9	Spermatid perinuclear RNA-binding protein.	Nuclear
Q9UNH5	[Dual specificity protein phosphatase CDC14A (EC 3.1.3.48), (EC 3.1.3.16) (CDC14 cell division cycle 14 homolog A).]	Nuclear
Q14192	[Four and a half LIM domains protein 2 (FHL-2) (Skeletal muscle LIM-, protein 3) (SLIM 3) (LIM domain protein DRAL).]	Nuclear
P48552	[Nuclear receptor-interacting protein 1 (Nuclear factor RIP140), (Receptor-interacting protein 140).]	Nuclear
Q13342	[Nuclear body protein SP140 (Nuclear autoantigen Sp-140) (Speckled 140, kDa) (LYSp100 protein) (Lymphoid-restricted homolog of Sp100).]	Nuclear
Q9NPG3	Ubinuclein (Ubiquitously expressed nuclear protein) (VT4).	Nuclear
O95373	Importin-7 (Imp7) (Ran-binding protein 7) (RanBP7).	Nuclear Membrane
Q08043	[Alpha-actinin-3 (Alpha-actinin skeletal muscle isoform 3) (F-actin, cross-linking protein).]	Other Filament or Tubule
P41208	Centrin-2 (Caltractin isoform 1).	Other Filament or Tubule
Q9NZQ3	[SH3 adapter protein SPIN90 (NCK-interacting protein with SH3 domain), (SH3 protein interacting with Nck, 90 kDa) (VacA-interacting protein,, 54 kDa) (VIP54) (AF3p21) (Diaphanous protein-interacting protein), (Dia-interacting protein 1) (DIP-1).]	Other Filament or Tubule

P04264	[Keratin, type II cytoskeletal 1 (Cytokeratin-1) (CK-1) (Keratin-1), (K1) (67 kDa cytokeratin) (Hair alpha protein).]	Other Filament or Tubule
Q15029	[116 kDa U5 small nuclear ribonucleoprotein component (U5 snRNP-, specific protein, 116 kDa) (U5-116 kDa) (Elongation factor Tu GTP-, binding domain protein 2) (hSNU114).]	Other Go Component
Q92870	[Amyloid beta A4 precursor protein-binding family B member 2 (Fe65-like, protein).]	Other Go Component
P42566	[Epidermal growth factor receptor substrate 15 (Protein Eps15) (AF-1p, protein).]	Other Go Component
Q15517	Corneodesmosin precursor (S protein).	Other Go Component
P01619	Ig kappa chain V-III region B6.	Other Go Component
Q9P241	Probable phospholipid-transporting ATPase VD (EC 3.6.3.1) (ATPVD).	Other Membrane
P29323	[Ephrin type-B receptor 2 precursor (EC 2.7.10.1) (Tyrosine-protein, kinase receptor EPH-3) (DRT) (Receptor protein-tyrosine kinase HEK5), (ERK) (Tyrosine-protein kinase TYRO5) (Renal carcinoma antigen NY-REN-, 47).]	Other Membrane
P04220	Ig mu heavy chain disease protein (BOT).	Other Membrane
O00631	Sarcolipin.	Other Membrane
Q7LFX5	[N-acetylgalactosamine 4-sulfate 6-O-sulfotransferase (EC 2.8.2.33), (GalNAc4S-6ST) (B-cell RAG-associated gene protein) (hBRAG).]	Other Membrane
P51693	Amyloid-like protein 1 precursor (APLP) (APLP-1) [Contains: C30].	Other Membrane
Q93045	Stathmin-2 (Protein SCG10) (Superior cervical ganglion-10 protein).	Other Membrane
Q9NP80	[Calcium-independent phospholipase A2-gamma (EC 3.1.1.5) (Intracellular, membrane-associated calcium-independent phospholipase A2 gamma), (iPLA2-gamma) (Patatin-like phospholipase domain-containing protein, 8).]	Other Membrane
Q86UD5	[NHEDC2 protein (Putative uncharacterized protein LOC133308) (CDNA, FLJ23984 fis, clone HEP22543).]	Other Membrane
P13726	[Tissue factor precursor (TF) (Coagulation factor III) (Thromboplastin), (CD142 antigen).]	Other Membrane
Q07065	Cytoskeleton-associated protein 4 (63 kDa membrane protein) (p63).	Other Membrane
O60883	[Endothelin B receptor-like protein 2 precursor (ETBR-LP-2) (G-protein, coupled receptor 37-like 1).]	Other Membrane
Q96PD7	[Diacylglycerol O-acyltransferase 2 (EC 2.3.1.20) (Diglyceride, acyltransferase 2).]	Other Membrane
P29475	[Nitric-oxide synthase, brain (EC 1.14.13.39) (NOS type I) (Neuronal, NOS) (N-NOS) (nNOS) (Constitutive NOS) (NC-NOS) (bNOS).]	Other Membrane
Q8WZA2	[Rap guanine nucleotide exchange factor 4 (cAMP-regulated guanine, nucleotide exchange factor II) (cAMP-GEFII) (Exchange factor directly, activated by cAMP 2) (Epac 2).]	Other Membrane
Q6ZMB0	[UDP-GlcNAc:betaGal beta-1,3-N-acetylglucosaminyltransferase 6, (EC 2.4.1.-) (Beta3Gn-T6) (BGnT-6) (Beta-1,3-N-, acetylglucosaminyltransferase-6) (Core 3 synthase).]	Other Membrane
Q96RY8	[Putative chloride channel protein 7 (Chloride channel 7, isoform, CRA_b).]	Other Membrane
O75976	[Carboxypeptidase D precursor (EC 3.4.17.22) (Metallocarboxypeptidase, D) (gp180).]	Other Membrane
Q14868	Effector cell protease receptor 1 (Fragment).	Other Membrane
Q5EGP2	G protein-coupled receptor 112.	Other Membrane
Q15391	[P2Y purinoceptor 14 (P2Y14) (UDP-glucose receptor) (G-protein coupled, receptor 105).]	Other Membrane
Q86UC7	Solute carrier family 5 (Sodium/glucose cotransporter), member 12.	Other Membrane
Q13621	[Solute carrier family 12 member 1 (Bumetanide-sensitive sodium-, (potassium)-chloride cotransporter 2) (Kidney-specific Na-K-Cl, symporter).]	Other Membrane
O95342	Bile salt export pump (ATP-binding cassette sub-family B member 11).	Plasma Membrane

Q08345	[Epithelial discoidin domain-containing receptor 1 precursor, (EC 2.7.10.1) (Epithelial discoidin domain receptor 1) (Tyrosine, kinase DDR) (Discoidin receptor tyrosine kinase) (Tyrosine-protein, kinase CAK) (Cell adhesion kinase) (TRK E) (Protein-tyrosine kinase, RTK 6) (HGK2) (CD167a antigen).]	Plasma Membrane
Q9Y2J2	[Band 4.1-like protein 3 (4.1B) (Differentially expressed in, adenocarcinoma of the lung protein 1) (DAL-1).]	Plasma Membrane
P48995	Short transient receptor potential channel 1 (TrpC1) (TRP-1 protein).	Plasma Membrane
P05067	[Amyloid beta A4 protein precursor (APP) (ABPP) (Alzheimer disease, amyloid protein) (Cerebral vascular amyloid peptide) (CVAP) (Protease, nexin-II) (PN-II) (APPI) (PreA4) [Contains: Soluble APP-alpha (S-APP-, alpha); Soluble APP-beta (S-APP-beta); C99; Beta-amyloid protein 42, (Beta-APP42); Beta-amyloid protein 40 (Beta-APP40); C83; P3(42);, P3(40); Gamma-CTF(59) (Gamma-secretase C-terminal fragment 59), (Amyloid intracellular domain 59) (AID(59)) (AICD-59); Gamma-CTF(57), (Gamma-secretase C-terminal fragment 57) (Amyloid intracellular domain, 57) (AID(57)) (AICD-57); Gamma-CTF(50) (Gamma-secretase C-terminal, fragment 50) (Amyloid intracellular domain 50) (AID(50)) (AICD-50);, C31].]	Plasma Membrane
P13569	[Cystic fibrosis transmembrane conductance regulator (CFTR) (cAMP-, dependent chloride channel) (ATP-binding cassette transporter sub-, family C member 7).]	Plasma Membrane
P11362	[Basic fibroblast growth factor receptor 1 precursor (EC 2.7.10.1), (FGFR-1) (bFGF-R) (Fms-like tyrosine kinase 2) (c-fgr) (CD331, antigen).]	Plasma Membrane
P38646	[Stress-70 protein, mitochondrial precursor (75 kDa glucose-regulated, protein) (GRP 75) (Heat shock 70 kDa protein 9) (Peptide-binding, protein 74) (PBP74) (Mortalin) (MOT).]	Plasma Membrane
P05556	[Integrin beta-1 precursor (Fibronectin receptor subunit beta), (Integrin VLA-4 subunit beta) (CD29 antigen).]	Plasma Membrane
Q92823	[Neuronal cell adhesion molecule precursor (Nr-CAM) (NgCAM-related cell, adhesion molecule) (Ng-CAM-related) (hBravo).]	Plasma Membrane
Q13332	[Receptor-type tyrosine-protein phosphatase S precursor (EC 3.1.3.48), (R-PTP-S) (Protein-tyrosine phosphatase sigma) (R-PTP-sigma).]	Plasma Membrane
Q07157	[Tight junction protein ZO-1 (Zonula occludens protein 1) (Zona, occludens protein 1) (Tight junction protein 1).]	Plasma Membrane
O60469	Down syndrome cell adhesion molecule precursor (CHD2).	Plasma Membrane
Q92859	Neogenin precursor.	Plasma Membrane
Q9Y5I3	Protocadherin alpha 1 precursor (PCDH-alpha1).	Plasma Membrane
Q9UHC3	[Amiloride-sensitive cation channel 3 (Acid-sensing ion channel 3), (ASIC3) (hASIC3) (Testis sodium channel 1) (hTNaC1).]	Plasma Membrane
Q13822	[Ectonucleotide pyrophosphatase/phosphodiesterase family member 2, precursor (EC 3.1.4.39) (E-NPP 2) (Extracellular lysophospholipase D), (LysoPLD) (Autotaxin).]	Plasma Membrane
P08473	[Neprilysin (EC 3.4.24.11) (Neutral endopeptidase) (NEP), (Enkephalinase) (Neutral endopeptidase 24.11) (Atriopeptidase) (Common, acute lymphocytic leukemia antigen) (CALLA) (CD10 antigen).]	Plasma Membrane
P78562	[Phosphate-regulating neutral endopeptidase (EC 3.4.24.-), (Metalloendopeptidase homolog PEX) (X-linked hypophosphatemia protein), (HYP) (Vitamin D-resistant hypophosphatemic rickets protein).]	Plasma Membrane
P08571	[Monocyte differentiation antigen CD14 precursor (Myeloid cell-specific, leucine-rich glycoprotein) [Contains: Monocyte differentiation antigen, CD14, urinary form; Monocyte differentiation antigen CD14, membrane-, bound form].]	Plasma Membrane
Q14982	[Opioid-binding protein/cell adhesion molecule precursor (OBCAM), (Opioid-binding cell adhesion molecule) (OPCML).]	Plasma Membrane
Q99665	[Interleukin-12 receptor beta-2 chain precursor (IL-12 receptor beta-2), (IL-12R-beta2).]	Plasma Membrane
Q9NPB9	[C-C chemokine receptor type 11 (C-C CKR-11) (CC-CKR-11) (CCR-11) (CC, chemokine receptor-like 1) (CCRL1) (CCX CKR).]	Plasma Membrane
P29320	[Ephrin type-A receptor 3 precursor (EC 2.7.10.1) (Tyrosine-protein, kinase receptor ETK1) (HEK) (HEK4) (Tyrosine-protein kinase TYRO4).]	Plasma Membrane
O60486	[Plexin-C1 precursor (Virus-encoded semaphorin protein receptor) (CD232, antigen).]	Plasma Membrane

O15244	Solute carrier family 22 member 2 (Organic cation transporter OCT2).	Plasma Membrane
Q9BVA0	[Katanin p80 WD40-containing subunit B1 (Katanin p80 subunit B1) (p80, katanin).]	Protein Complex
P25054	Adenomatous polyposis coli protein (Protein APC).	Protein Complex
P68871	[Hemoglobin subunit beta (Hemoglobin beta chain) (Beta-globin), [Contains: LVV-hemorphin-7].]	Protein Complex
Q99460	[26S proteasome non-ATPase regulatory subunit 1 (26S proteasome, regulatory subunit RPN2) (26S proteasome regulatory subunit S1) (26S, proteasome subunit p112).]	Protein Complex
Q14139	Ubiquitin conjugation factor E4 A.	Protein Complex
Q53F97	Alpha 2 globin variant (Fragment).	Protein Complex
Q9UKX3	[Myosin-13 (Myosin heavy chain 13) (Myosin heavy chain, skeletal, muscle, extraocular) (MyHC-eo).]	Protein Complex
P61289	[Proteasome activator complex subunit 3 (Proteasome activator 28, subunit gamma) (PA28gamma) (PA28g) (Activator of multicatalytic, protease subunit 3) (11S regulator complex subunit gamma) (REG-gamma), (Ki nuclear autoantigen).]	Protein Complex
O60934	[Nibrin (Nijmegen breakage syndrome protein 1) (Cell cycle regulatory, protein p95).]	Protein Complex
Q9BX26	[Synaptonemal complex protein 2 (SCP-2) (Synaptonemal complex lateral, element protein) (hsSCP2).]	Protein Complex
P02675	Fibrinogen beta chain precursor [Contains: Fibrinopeptide B].	Protein Complex
Q6P9B9	Integrator complex subunit 5 (Int5).	Protein Complex
P52732	[Kinesin-like protein KIF11 (Kinesin-related motor protein Eg5), (Kinesin-like spindle protein HKSP) (Thyroid receptor-interacting, protein 5) (TRIP-5) (Kinesin-like protein 1).]	Protein Complex
Q9NP71	[Williams-Beuren syndrome chromosomal region 14 protein (WS basic-, helix-loop-helix leucine zipper protein) (WS-bHLH) (Mlx interactor), (MLX-interacting protein-like).]	Transcription
P21675	[Transcription initiation factor TFIID subunit 1 (EC 2.7.11.1), (Transcription initiation factor TFIID 250 kDa subunit) (TAF(II)250), (TAFII-250) (TAFII250) (TBP-associated factor 250 kDa) (p250) (Cell, cycle gene 1 protein).]	Transcription
Q6P1X5	[Transcription initiation factor TFIID subunit 2 (Transcription, initiation factor TFIID 150 kDa subunit) (TBP-associated factor 150, kDa) (TAFII-150) (TAFII150) (150 kDa cofactor of initiator function), (RNA polymerase II TBP-associated factor subunit B).]	Transcription
O00268	[Transcription initiation factor TFIID subunit 4 (TBP-associated factor, 4) (Transcription initiation factor TFIID 135 kDa subunit), (TAF(II)135) (TAFII-135) (TAFII135) (TAFII-130) (TAFII130) (RNA, polymerase II TBP-associated factor subunit C).]	Transcription
Q13485	[Mothers against decapentaplegic homolog 4 (SMAD 4) (Mothers against, DPP homolog 4) (Deletion target in pancreatic carcinoma 4) (hSMAD4).]	Transcription
P53992	Protein transport protein Sec24C (SEC24-related protein C).	Vesicle
P02787	[Serotransferrin precursor (Transferrin) (Siderophilin) (Beta-1-metal-, binding globulin).]	Vesicle
O60656	[UDP-glucuronosyltransferase 1-9 precursor (EC 2.4.1.17) (UDP-, glucuronosyltransferase 1A9) (UDPGT) (UGT1*9) (UGT1-9) (UGT1.9) (UGT-, 1I) (UGT1I) (lugP4).]	Vesicle
